# A highly enantioselective intramolecular 1,3-dipolar cycloaddition yields novel pseudo-natural product inhibitors of the Hedgehog signalling pathway†

**DOI:** 10.1039/d3sc01240a

**Published:** 2023-06-21

**Authors:** Jie Liu, Ruirui Zhang, Shubhadip Mallick, Sohan Patil, Chantal Wientjens, Jana Flegel, Anna Krupp, Carsten Strohmann, Corentin Grassin, Christian Merten, Axel Pahl, Michael Grigalunas, Herbert Waldmann

**Affiliations:** a Department of Chemical Biology, Max Planck Institute of Molecular Physiology Otto-Hahn-Street 11 44227 Dortmund Germany Herbert.waldmann@mpi-dortmund.mpg.de; b Faculty of Chemistry, Chemical Biology, Technical University Dortmund Otto-Hahn-Street 6 44221 Dortmund Germany; c Faculty of Chemistry, Inorganic Chemistry, Technical University Dortmund Otto-Hahn-Street 6 44221 Dortmund Germany; d Faculty of Chemistry and Biochemistry, Organic Chemistry II, Ruhr University Bochum University-Street 150 44801 Bochum Germany; e Compound Management and Screening Center Otto-Hahn-Street 11 44227 Dortmund Germany

## Abstract

*De novo* combination of natural product (NP) fragments by means of efficient, complexity- and stereogenic character-generating transformations to yield pseudo-natural products (PNPs) may explore novel biologically relevant chemical space. Pyrrolidine- and tetrahydroquinoline fragments rarely occur in combination in nature, such that PNPs that embody both fragments might represent novel NP-inspired chemical matter endowed with bioactivity. We describe the synthesis of pyrrolo[3,2-*c*]quinolines by means of a highly enantioselective intramolecular *exo*-1,3-dipolar cycloaddition catalysed by the AgOAc/(*S*)-DMBiphep complex. The cycloadditions proceeded in excellent yields (up to 98%) and with very high enantioselectivity (up to 99% ee). Investigation of the resulting PNP collection in cell-based assays monitoring different biological programmes led to the discovery of a structurally novel and potent inhibitor of the Hedgehog signalling pathway that targets the Smoothened protein.

## Abstract 2

Synthesis of pyrrolo[3,2-*c*]quinoline pseudo-natural products by means of a highly enantioselective intramolecular 1,3-dipolar cycloaddition yielded a novel Hedgehog signalling inhibitor chemotype that targets the Smoothened protein.

## Graphical abstract

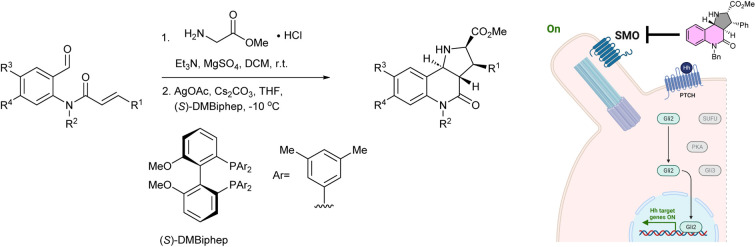

## Introduction

Compound classes endowed with pronounced bioactivity are often inspired by natural product (NP) structure and have high stereogenic content, which positively correlates with performance in drug discovery.^1^ To identify such novel NP-inspired bioactive matter, principles including pseudo-natural product (PNP) design,^2^ biology-oriented synthesis (BIOS)^3^ and the complexity-to-diversity (CtD) approach^4^ have been developed as guiding strategies. In PNP design and synthesis, NP fragments are combined in novel arrangements not found in nature by means of efficient, complexity- and stereogenic character-generating transformations, and for such syntheses enantioselective 1,3-dipolar cycloadditions have proven to be of high value.^2*d*,5^

**Fig. 1:**
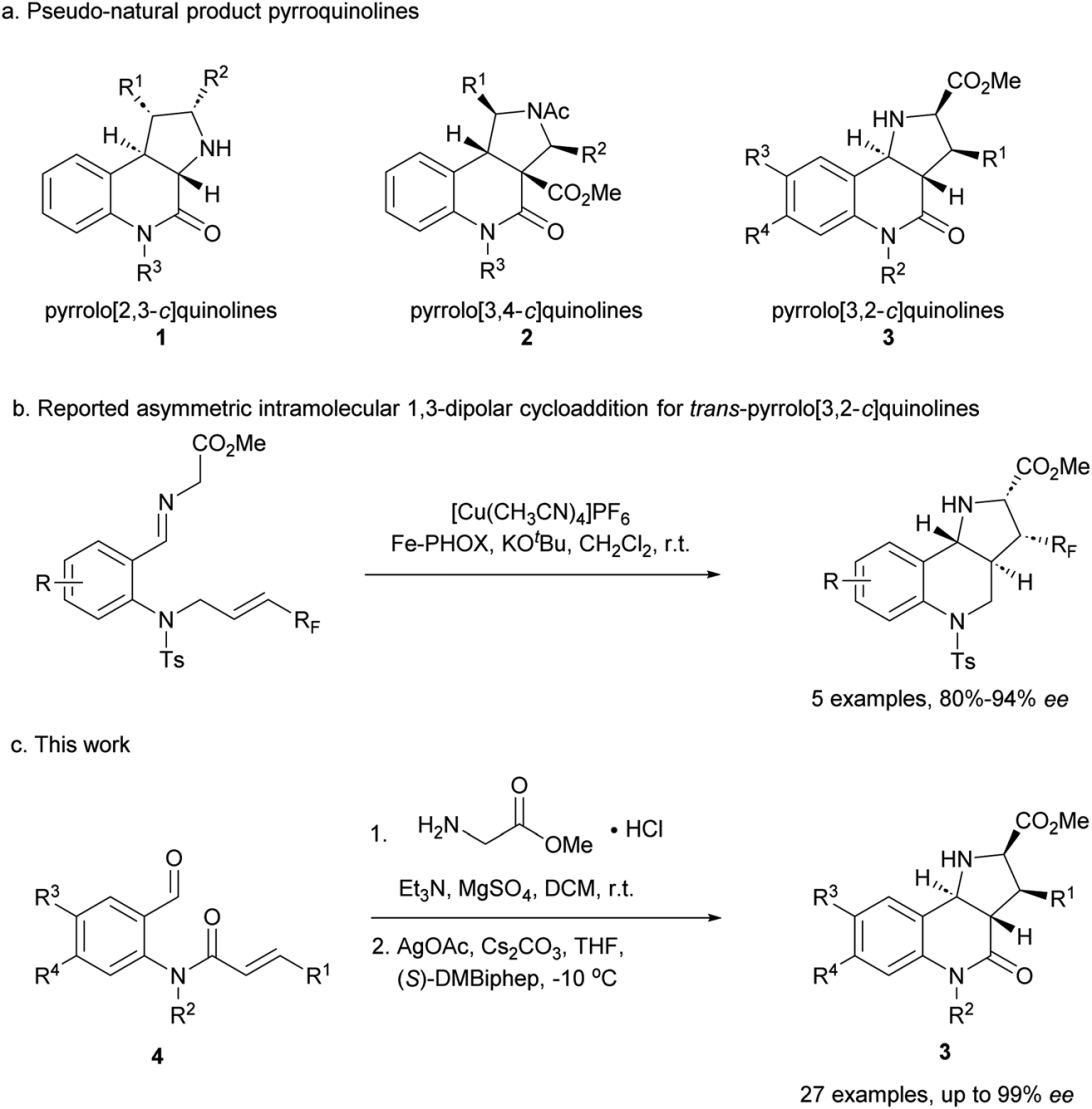
Asymmetric synthesis of pyrrolo[3,2-*c*]quinolines. (a) Reported pyrroquinolines with different connectivities. (b) Reported asymmetric synthesis of *trans*-pyrrolo[3,2-*c*]quinolines. (c) This work.

The pyrrolidine-derived and tetrahydroquinoline alkaloid-derived fragments frequently occur individually in NPs, but rarely in combination,^6^ such that PNPs that embody both fragments might represent novel NP-inspired chemical matter that is endowed with bioactivity. We have recently developed asymmetric syntheses of pyrrolo[2,3-*c*]quinolines 1 (ref. 7) and pyrrolo[3,4-*c*]quinolines 2 (ref. 8) and shown that the combination of pyrrolidine and tetrahydroquinoline fragments in different arrangements and with different connectivities that are not found in nature yields chemically and biologically diverse PNP classes.^9^ However, the regioisomeric pyrrolo[3,2-*c*]quinolines 3 (Fig. 1a) were only accessible in racemic^9,10^ and/or *cis* form.^11^ In principle, this scaffold would be available by means of an enantioselective intramolecular 1,3-dipolar cycloaddition, but such transformations have rarely been described. Recently, del Pozo and Adrio *et al.* reported a catalytic enantioselective intramolecular 1,3-dipolar cycloaddition to afford *trans*-configured fluorinated pyrroloquinolines (Fig. 1b), but with limited exploration of substrate scope.^12^

We have now developed a new enantioselective intramolecular 1,3-dipolar cycloaddition catalysed by a chiral AgOAc/DMBiphep complex that yields *trans*-pyrrolo[3,2-*c*]quinolines in high yields and with excellent enantioselectivity (Fig. 1c). Investigation of these pseudo-NPs in different biological assays identified a novel inhibitor of Hedgehog signalling targeting the protein Smoothened (SMO).

## Results and discussion

In order to identify favourable reaction conditions, aldehyde 4a was condensed with glycine methyl ester and the resulting iminoester was subjected to intramolecular cycloaddition without further purification in the presence of different chiral metal complexes (Table 1). Initial solvent screening revealed that the transformations proceeded best in THF (Table S1†). When Segphos was used as ligand (L1) and AgOAc as metal salt, the cycloadduct was isolated with 79% ee (Table 1, entry 1). Increasing steric hindrance of the substituents on Segphos did not improve the enantioselectivity (L2 and L3, Table 1, entries 2 and 3). When the biaryl substituent was changed, only DMBiphep L5 with moderate steric demand gave a slight increase of enantioselectivity to 80% ee (Table 1, entry 5). Further increasing or decreasing the steric demand of the substituents led to lower enantioselectivity (Table 1, entries 4–10). Changing the metal catalyst from AgOAc to either a Cu(i) or a Cu(ii) salt led to greatly reduced enantioselectivity (Table 1, entries 11 and 12). Finally, lowering the temperature to −10 °C yielded the desired cycloadduct 3a in 90% ee and in viable yield (Table 1, entry 13).

**Table 1:** Screening of reaction conditions for the intramolecular 1,3-dipolar cycloaddition. Unless otherwise specified, aldehyde (0.05 mmol, 1.0 equiv.), MgSO_4_ (3.0 equiv.) and Et_3_N (3.0 equiv.) were used for iminoester formation. Then, catalyst (10 mol%), ligand (12 mol%), Cs_2_CO_3_ (20 mol%) in THF (1.0 mL). Isolated yield after column chromatography. The ee was determined by chiral HPLC

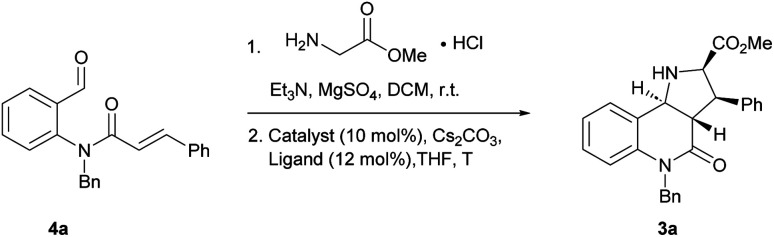					
Entry	Catalyst	Ligand	*T* [°C]	Yield [%]	ee [%]
1	AgOAc	L1	r.t.	90	79
2	AgOAc	L2	r.t.	65	74
3	AgOAc	L3	r.t.	64	5
4	AgOAc	L4	r.t.	80	73
5	AgOAc	L5	r.t.	83	80
6	AgOAc	L6	r.t.	66	17
7	AgOAc	L7	r.t.	85	6
8	AgOAc	L8	r.t.	85	26
9	AgOAc	L9	r.t.	77	71
10	AgOAc	L10	r.t.	85	67
11	[Cu(CH_3_CN)_4_]BF_4_	L5	r.t.	70	51
12	Cu(OAc)_2_	L5	r.t.	50	56
13^a^	AgOAc	L5	−10	67	90
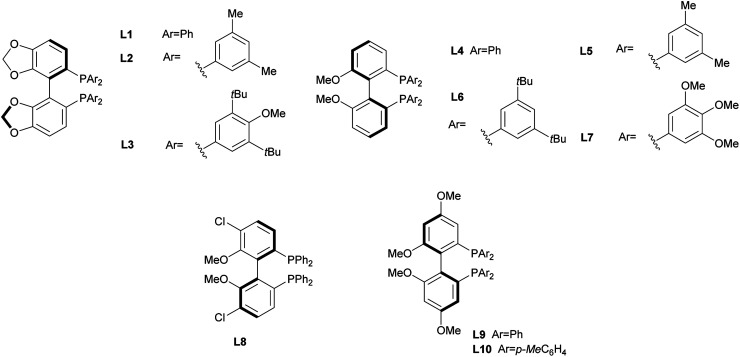					

a The reaction was performed on 0.1 mmol scale.

With favourable conditions identified, the substrate scope of this asymmetric reaction was explored (Table 2). When chloro-substituents were introduced into the phenyl ring of the cinnamic acid unit (R^1^), enantioselectivity was lower regardless of the substitution site (Table 2, entries 2–4). Also, differently functionalised *N*-benzyl substituents or an *N*-methyl group (R^2^) led to a decrease of ee to 79–88% (Table 2, entries 5–8). Conversely, introduction of a substituent *meta* to the aldehyde in starting compound 4 (*i.e.* R^3^), which corresponds to the C6 position in the final quinoline scaffold, increased the enantioselectivity. Diverse substituents with different electronic properties or steric demand were well tolerated and afforded the desired cycloadducts 3 in good yields and with excellent enantioselectivity (Table 2, entries 9–14). A decreased enantioselectivity was observed when the substituents were introduced *para* to the aldehyde in 4a, *i.e.* R^4^ at the C7 position of the quinoline (Table 2, entries 15 and 16). The relative and absolute configuration of the cycloadducts were determined by X-ray crystallography for *rac*-3j and computed vibrational circular dichroism (VCD) spectra^13^ for 3l, respectively, and were assigned by analogy to the other cycloadducts (see Fig. S1† for details).

**Table 2:** Substrate scope for the asymmetric intramolecular 1,3-dipolar cycloaddition

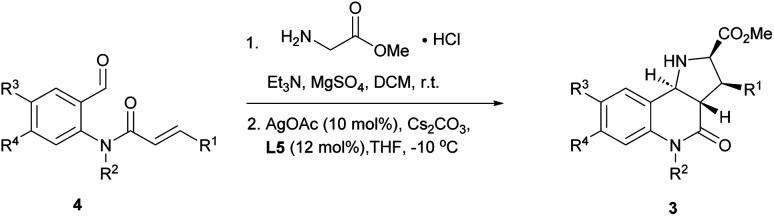							
Entry	Product	R^1^	R^2^	R^3^	R^4^	Yield [%]	ee [%]
1	3a	Ph	Bn	H	H	67	90
2	3b	*p*-ClC_6_H_4_	Bn	H	H	92	85
3	3c	*m*-ClC_6_H_4_	Bn	H	H	81	76
4	3d	*o*-ClC_6_H_4_	Bn	H	H	73	75
5	3e	Ph	*o*-Me benzyl	H	H	62	88
6	3f	Ph	*m*-Br benzyl	H	H	47	85
7	3g	Ph	*p*-Cl benzyl	H	H	59	85
8	3h	Ph	Me	H	H	56	79
9	3i	Ph	Bn	F	H	75	94
10	3j	Ph	Bn	Cl	H	76	97
11	3k	Ph	Bn	Br	H	84	96
12	3l	Ph	Bn	Me	H	98	97
13	3m	Ph	Bn	MeO	H	63	98
14	3n	Ph	Bn	CF_3_O	H	69	96
15	3o	Ph	Bn	H	F	68	83
16	3p	Ph	Bn	H	Br	82	80
17	3q	*p*-ClC_6_H_4_	Bn	Me	H	59	96
18	3r	*m*-ClC_6_H_4_	Bn	Me	H	67	97
19	3s	*o*-ClC_6_H_4_	Bn	Me	H	92	94
20	3t	Ph	*o*-Me benzyl	Br	H	59	97
21	3u	Ph	*o*-Me benzyl	Me	H	52	98
22	3v	Ph	*m*-Br benzyl	F	H	61	98
23	3w	Ph	*m*-Br benzyl	Me	H	65	98
24	3x	Ph	*p*-Cl benzyl	MeO	H	58	99
25	3y	Ph	Me	F	H	66	90
26	3z	Ph	Bn	F	F	70	96
27	3aa	Ph	Bn	–C_4_H_4_–		59	93
28	3bb	*p*-CNC_6_H_4_	Bn	H	H	91	4
29	3cc	Ph	*p*-CN benzyl	H	H	94	88

To further explore the positive impact of the quinoline C6 substituent (R^3^) on enantioselectivity, diverse substituent combinations were investigated. In the presence of a methyl group at R^3^, enantioselectivity improved from 75%–85% to 94%–97% for regioisomeric chlorophenyl substituents at R^1^ (Table 2, entries 2–4 and 17–19). By analogy, excellent ee values were recorded for different substitutions of the benzyl group at R^2^ as long as there are substituents at the final quinoline C6 position (Table 2, entries 20–25). Even when R^2^ is a methyl group, a sterically less demanding substitution with a fluorine atom at the C6 position improved the enantioselectivity from 79% to 90% (Table 2, entries 8 and 25). When both R^3^ and R^4^ were fluorine or represented a fusion with a benzene ring, enantioselectivity was also very high (Table 2, entries 26 and 27).

The reaction was incompatible with a strong electron-withdrawing group at the R^1^ position. Thus, in the case of a *para*-cyanide substituent hardly any enantioselectivity was observed under standard conditions (Table 2, entry 28). However, when the cyanide substituent was at the R^2^ position on the benzyl group, only a minor effect was observed and the reaction proceeded in excellent yield and with good enantioselectivity (Table 2, entry 29).

**Fig. 2:**
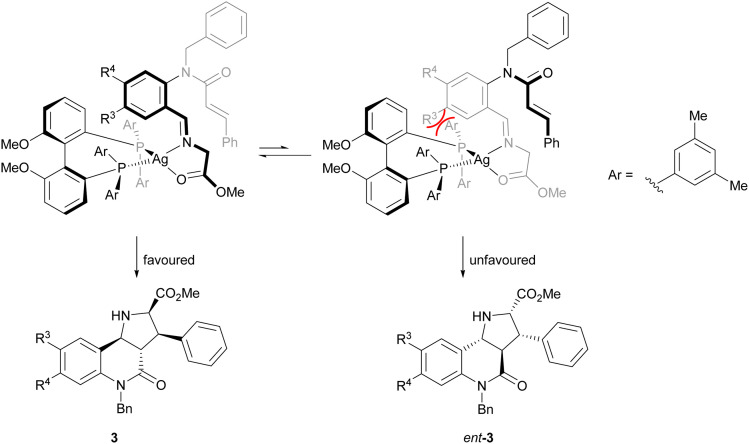
Proposed transition state for the 1,3-dipolar cycloaddition.

The direction of the stereoselection and the pronounced influence of the substituent *meta* to the aldehyde in starting material 4 (R^3^) may be rationalised by the transition state proposed in Fig. 2. The silver ion most likely would be chelated by the bidentate phosphine ligand (*S*)-DMBiphep and the iminoester intermediate, which would be deprotonated to the azomethine ylide by Cs_2_CO_3_. In this transition state, the dipolarophile would preferably approach the 1,3-dipole from the back side to avoid steric repulsion between the substrate backbone and the ligand. In particular, in the presence of a substituent R^3^, the repulsion would be pronounced, thus leading to higher enantioselectivity. In comparison, no improved enantioselectivity was recorded if a substituent R^4^ was introduced because this position does not point towards the ligand. We also note that the intramolecular cycloaddition proceeds with an *exo* approach due to the steric demands and conformational rigidity in the transition state.

In order to investigate whether the cycloadducts 3 are endowed with bioactivity, they were subjected to cell-based assays monitoring different biological programmes, such as oncogenic signalling, autophagy and immunometabolism. Gratifyingly, the compound collection defines a new inhibitor chemotype of signal transduction through the Hedgehog pathway. The Hedgehog (Hh) signalling pathway plays a critical role in the regulation of embryonic development, post-embryonic tissue homeostasis and regeneration in vertebrates.^14^ Excessive activity of the pathway is associated with malignancy, such as medulloblastoma,^15^ basal cell carcinoma^16^ and rhabdomyosarcoma.^17^ Therefore, inhibitors of the Hh signalling pathway have emerged as attractive therapeutic options in oncology.^18^

**Fig. 3:**
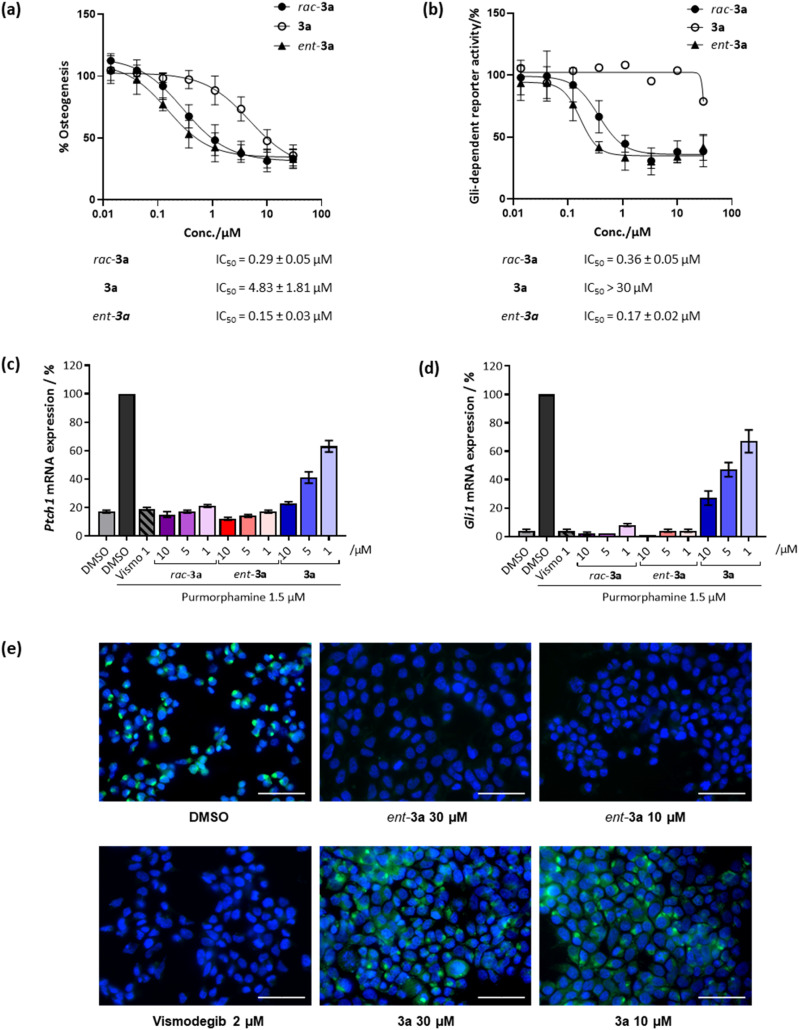
Biological characterisation of selected compounds for Hh pathway inhibition. (a) Osteoblast differentiation assay. C3H10T1/2 cells were treated with 1.5 μM purmorphamine together with DMSO as a control or compounds for 96 h. The activity of alkaline phosphatase was quantified as a measure of Hh pathway activity. Values for cells treated with purmorphamine and DMSO were set to 100%. Data are mean values ± SD (*n* = 3). (b) Gli-responsive reporter gene assay. Shh-LIGHT2 cells were treated with 2 μM purmorphamine together with DMSO as a control or compounds for 48 h. The Gli-responsive firefly luciferase signals were normalised to the signal for *Renilla* luciferase. The value for the purmorphamine/DMSO control was set to 100%. Data are mean values ± SD and are representative of three biological replicates (*n* = 3). (c) and (d) Expression of the Hh target genes *Ptch1* (c) and (d) *Gli1*. C3H10T1/2 cells were incubated with 1.5 μM purmorphamine and DMSO, 1 μM vismodegib (Vismo) or compounds for 96 h prior to RT-qPCR. Data are mean values ± SD (*n* = 3). (e) Smoothened binding assay. HEK293T cells were transfected with a SMO-expressing plasmid and 48 h later cells were fixed and incubated with 5 nM BODIPY-cyclopamine (green) and treated with either DMSO (negative control), vismodegib (2 μM, positive control) or compounds at 30 μM or 10 μM for 4 h. The cells were stained with 4′,6-diamidino-2-phenylindole (DAPI, blue) to visualise the nuclei. Images are overlayed images of DAPI and BODIPY signals, representative of three biological replicates (*n* = 3). Scale bar: 50 μm.

Initially, racemic pseudo-NPs were subjected to a phenotypic Hh-dependent osteoblast differentiation assay to monitor their possible impact on the Hh signalling pathway upon activation by purmorphamine, a pathway agonist binding to the Smoothened protein.^19^ Gratifyingly, most of the compounds showed potent inhibition of Hh signalling (see Table S2†), with the most potent compound *rac*-3a displaying a half-maximal inhibitory concentration (IC_50_) of 0.29 ± 0.05 μM (Fig. 3a). Interestingly, 3a (90% ee) showed an IC_50_ value of 4.83 ± 1.81 μM, while its enantiomer *ent*-3a (90% ee) displayed an approximately 30-fold lower IC_50_ value of 0.15 ± 0.03 μM. This tendency was also observed in the orthogonal Gli-dependent reporter gene assay in Shh-LIGHT2 cells^20^ with an IC_50_ of 0.17 ± 0.02 μM for *ent*-3a while 3a was inactive (IC_50_ > 30 μM, Fig. 3b). Hh target gene expression was explored to validate the inhibition of the Hh pathway. Compound *ent*-3a reduced the expression of Hh target genes *Ptch1* and *Gli1* dose dependently (Fig. 3c and d); at 1 μM, *Ptch1* and *Gli1* gene expression was suppressed by 83% and 96%, respectively. The activity of *ent*-3a was comparable to the Hh pathway inhibitor vismodegib at the same concentration, which is clinically approved for the treatment of basal cell carcinoma.^21^

The Hh pathway is activated by the binding of Hh ligands to the *trans*-membrane receptor Patched1 (Ptch1). Upon binding, cellular internalisation of Ptch1 relieves the Ptch1-mediated inhibition of the Smoothened (SMO) protein, allowing for SMO translocation to the membrane, which triggers a signalling cascade that ultimately activates transcription of Hh target genes such as *Ptch1* and glioma-associated oncogene homolog 1 (*Gli1*).^14^ The protein SMO is a clinically validated therapeutic target and many relevant Hh pathway inhibitors act by SMO antagonism, including vismodegib and cyclopamine, which specifically bind to the heptahelical bundle of SMO leading to pathway inhibition.^22^ To detect whether 3a also binds to SMO, a competitive SMO-binding assay was performed with a BODIPY-labelled derivative of the SMO binder cyclopamine.^19^ HEK293T cells transfected with a SMO-expressing plasmid were incubated with the compounds and BODIPY-cyclopamine at 5 nM. Less potent compound 3a did not decrease BODIPY-related fluorescence (Fig. 3e). In contrast, vismodegib and the potent enantiomer *ent*-3a decreased BODIPY fluorescence, indicating competition with cyclopamine for SMO binding and the displacement of BODIPY-cyclopamine from SMO (Fig. 3e). Thus, strong inhibitory activity exhibited by *ent*-3a on the Hedgehog pathway most likely is the result of direct binding to SMO.

Molecular docking was performed to determine the potential binding mode of *ent*-3a to SMO (Fig. S2†). Seven different crystal structures of human SMO (PDB ID: 4JKV, 4N4W, 4O9R, 4QIM, 4QIN, 5L7I and 5V56) were processed for the modelling. Only structure 5L7I could differentiate well between 3a and *ent*-3a according to the docking scores, which suggests that *ent*-3a may adopt a similar binding mode, as observed for the ligand vismodegib in 5L7I.^23^ The binding pose suggests that *ent*-3a occupies a space closer to the entrance of the pocket, while vismodegib resides deeper inside. For this model, the key interactions are hydrogen bonding between the ester carbonyl oxygen of *ent*-3a to Arg400 and of an amino proton to Gln477 (Fig. S2b†). Additionally, this binding pose suggests a stabilising π-stacking interaction with Phe484. This binding mode supports the substituent preference observed in the structure–activity relationship investigation (Table S2†). Any additional substituents at R^3^ would impair the π–π interaction, and only a small fluorine substitution at R^3^ afforded potency comparable to *rac*-3a (Table S2,† entry 9). Hydrogen bonding between *ent*-3a and SMO Gln477 proves to be vital for the binding, as acetylation of 3a totally abolishes activity (Table S2,† entry 16). The incompatibility of different substituents at R^1^ or R^2^ may be attributed to the rigidity of the scaffold, where a distant substitution leads to conformational change of the entire molecule.

SMO mutations have been associated with Hh pathway-driven oncogenicity and acquired resistance to treatment with drugs, limiting the clinical success of SMO antagonists.^24^ The first and only characterised mechanism of resistance to date is a mutation of Asp473 to a histidine (D473H), observed in a relapsed patient with metastatic medulloblastoma after vismodegib treatment.^24*b*,25^ It is believed that D473H mutation impairs the tight binding of vismodegib through an indirect pathway.^26^ The cation–π interaction between Arg400 and the pyridine ring of vismodegib is a key source of high affinity, where Asp473 forms a tight hydrogen bond with Arg400 (Fig. S2a†). Therefore, D473H mutation can disrupt the orientation of Arg400 for interaction and lead to loss of binding. In the model of SMO binding to *ent*-3a, Arg400 also forms a hydrogen bond with the ester motif, and thus for this compound it is most likely that a similar resistance might evolve. Combination therapies with downstream antagonists may be an option to circumvent such drug resistance.

The pharmacokinetic properties of vismodegib and *ent*-3a were compared by calculating their physicochemical properties using the webtool SwissADME.^27^ Notably, *ent*-3a resides in the physiochemical space predicted for good oral bioavailability (Fig. S3a†), while vismodegib has one violation in the dimension of INSATU (fraction of carbons in the sp^3^ hybridisation) due to its relatively high degree of saturation (Fig. S3b†). Considering the application of SMO inhibitors for medulloblastoma treatment, penetrating the blood–brain barrier (BBB) is another major challenge. While the calculation suggests that vismodegib may not permeate the BBB, for pseudo-NP *ent*-3a the prediction is different (Fig. S3a†), indicating that *ent*-3a may have more favourable properties.

The unsaturated pyrrolo[3,2-*c*]quinoline scaffold was previously reported as a lead chemotype for SMO binding.^28^ Therefore, the aromatised planar pyrrolo[3,2-*c*]quinoline 5a derived from 3a was also tested in the purmorphamine-induced osteogenesis assay. However, a more than five-fold decrease of inhibition was observed (Table S2,† entry 15), indicating that in the case under investigation, an increase in saturation is beneficial among these structurally related chemotypes. This observation may be regarded as an example that an “escape from flatland”^1*a*,*d*^ may lead to more advantageous interactions between related small molecules and their cellular targets.

For comparison of the structural and chemical properties of *ent*-3a with previously described SMO antagonists, *ent*-3a and a reference set of 578 reported SMO antagonists (see ESI† for curation details)^29^ were subjected to cheminformatic analyses computed using the open-source software RDKit.^30^ Molecular shape was evaluated by a principal moments of inertia analysis.^31^ Most of the reported SMO antagonists have a rod-like shape, while *ent*-3a occupies a unique position with a more disc-like topology that is very sparsely populated by the reference set (Fig. S4a†). An NP-likeness score^32^ and a quantitative estimate of drug likeness (QED)^33^ were calculated and visualised in a two-dimensional plot to evaluate fragment compositions and drug-like properties, respectively. *Ent*-3a occupies an unpopulated area that has a high NP-likeness score and high QED score relative to the previously developed SMO antagonists (Fig. S4b†). This indicates that the pseudo-NP may be simultaneously more NP-like and drug-like than other SMO antagonists. Overall, these analyses suggest that the pseudo-NP *ent*-3a is characterised by a unique combination of molecular shape, atom connectivity and chemical properties relative to known SMO antagonists and represents a new chemotype for SMO inhibition.

## Conclusions

In conclusion we have developed a new enantioselective intramolecular 1,3-dipolar cycloaddition for the combination of the pyrrolidine- and tetrahydroquinoline-NP fragments. The highly challenging asymmetric cycloaddition was enabled by the use of a AgOAc/(*S*)-DMBiphep catalyst, resulting in excellent yields and enantioselectivity. Exploration of the substrate scope revealed that enantioselectivity in the cycloaddition is decisively influenced by the site-specific introduction of a substituent. Biological evaluation of the resulting pseudo-NP collection revealed a novel Hh signalling inhibitor, whose activity was highly dependent on absolute configuration. Compound *ent*-3a binds to the SMO protein and, thereby, potently suppresses Hh target gene expression to an extent similar to the clinically approved SMO antagonist vismodegib. Cheminformatic analysis of *ent*-3a and reported SMO antagonists revealed that the pseudo-NP has unique properties and constitutes a new chemotype for SMO inhibition.

## Data availability

All experimental and characterisation data, as well as NMR spectra are available in the ESI.† Crystallographic data for compound *rac*-3j have been deposited in the Cambridge Crystallographic Data Centre under accession number CCDC 2243515.

## Author contributions

J. L. and R. R. Z. contributed equally. J. L. and H. W. conceived and directed the project. J. L. and S. M. performed the organic synthesis. J. L., R. R. Z., S. P., C. W. and J. F. performed the biological evaluation of the compound. A. K. and C. S. conducted the X-ray crystallography. C. G. and C. M. performed the computation of VCD spectra. J. L. performed molecular docking and the prediction of drug-like properties. A. P. and M. G. carried out the cheminformatic analysis. J. L., R. R. Z. and H. W. wrote the paper. All authors discussed the results and commented on the manuscript.

## Conflicts of interest

The authors declare no conflict of interest.

## Supplementary Material

SC-014-D3SC01240A-s001

## References

[cit1] Lovering F., Bikker J., Humblet C. (2009). J. Med. Chem..

[cit2] Karageorgis G., Foley D. J., Laraia L., Waldmann H. (2020). Nat. Chem..

[cit3] Wetzel S., Bon R. S., Kumar K., Waldmann H. (2011). Angew. Chem., Int. Ed..

[cit4] Morrison K. C., Hergenrother P. J. (2014). Nat. Prod. Rep..

[cit5] Adrio J., Carretero J. C. (2019). Chem. Commun..

[cit6] Gao D., Zhou T., Da L.-T., Bruhn T., Guo L.-L., Chen Y.-H., Xu J., Xu M.-J. (2019). Org. Lett..

[cit7] Vidadala S. R., Golz C., Strohmann C., Daniliuc C.-G., Waldmann H. (2015). Angew. Chem., Int. Ed..

[cit8] Liu J., Otte F., Strohmann C., Waldmann H. (2021). Tetrahedron Lett..

[cit9] Liu J., Cremosnik G. S., Otte F., Pahl A., Sievers S., Strohmann C., Waldmann H. (2021). Angew. Chem., Int. Ed..

[cit10] Masse C. E., Ng P. Y., Fukase Y., Sánchez-Roselló M., Shaw J. T. (2006). J. Comb. Chem..

[cit11] Nyerges M., Fejes I., Tõke L. (2002). Synthesis.

[cit12] Cristóbal C., Gaviña D., Alonso I., Ribagorda M., Carretero J. C., del Pozo C., Adrio J. (2022). Chem. Commun..

[cit13] Merten C., Golub T. P., Kreienborg N. M. (2019). J. Org. Chem..

[cit14] Briscoe J., Thérond P. P. (2013). Nat. Rev. Mol. Cell Biol..

[cit15] Raffel C., Jenkins R. B., Frederick L., Hebrink D., Alderete B., Fults D. W., James C. D. (1997). Cancer Res..

[cit16] Xie J., Murone M., Luoh S. M., Ryan A., Gu Q., Zhang C., Bonifas J. M., Lam C. W., Hynes M., Goddard A., Rosenthal A., Epstein Jr E. H., de Sauvage F. J. (1998). Nature.

[cit17] Tostar U., Malm C. J., Meis-Kindblom J. M., Kindblom L.-G., Toftgård R., Undén A. B. (2006). J. Pathol..

[cit18] Peukert S., Miller-Moslin K. (2010). ChemMedChem.

[cit19] Sinha S., Chen J. K. (2006). Nat. Chem. Biol..

[cit20] Taipale J., Chen J. K., Cooper M. K., Wang B., Mann R. K., Milenkovic L., Scott M. P., Beachy P. A. (2000). Nature.

[cit21] Robarge K. D., Brunton S. A., Castanedo G. M., Cui Y., Dina M. S., Goldsmith R., Gould S. E., Guichert O., Gunzner J. L., Halladay J., Jia W., Khojasteh C., Koehler M. F. T., Kotkow K., La H., LaLonde R. L., Lau K., Lee L., Marshall D., Marsters J. C., Murray L. J., Qian C., Rubin L. L., Salphati L., Stanley M. S., Stibbard J. H. A., Sutherlin D. P., Ubhayaker S., Wang S., Wong S., Xie M. (2009). Bioorg. Med. Chem. Lett..

[cit22] Chen J. K., Taipale J., Cooper M. K., Beachy P. A. (2002). Genes Dev..

[cit23] Byrne E. F. X., Sircar R., Miller P. S., Hedger G., Luchetti G., Nachtergaele S., Tully M. D., Mydock-McGrane L., Covey D. F., Rambo R. P., Sansom M. S. P., Newstead S., Rohatgi R., Siebold C. (2016). Nature.

[cit24] Sharpe H. J., Wang W., Hannoush R. N., de Sauvage F. J. (2015). Nat. Chem. Biol..

[cit25] Rudin C. M., Hann C. L., Laterra J., Yauch R. L., Callahan C. A., Fu L., Holcomb T., Stinson J., Gould S. E., Coleman B., LoRusso P. M., Von Hoff D. D., de Sauvage F. J., Low J. A. (2009). N. Engl. J. Med..

[cit26] Sharpe H. J., Pau G., Dijkgraaf G. J., Basset-Seguin N., Modrusan Z., Januario T., Tsui V., Durham A. B., Dlugosz A. A., Haverty P. M., Bourgon R., Tang J. Y., Sarin K. Y., Dirix L., Fisher D. C., Rudin C. M., Sofen H., Migden M. R., Yauch R. L., de Sauvage F. J. (2015). Cancer Cell.

[cit27] Daina A., Michielin O., Zoete V. (2017). Sci. Rep..

[cit28] Ohashi T., Oguro Y., Tanaka T., Shiokawa Z., Shibata S., Sato Y., Yamakawa H., Hattori H., Yamamoto Y., Kondo S., Miyamoto M., Tojo H., Baba A., Sasaki S. (2012). Bioorg. Med. Chem..

[cit29] Grigalunas M., Patil S., Krzyzanowski A., Pahl A., Flegel J., Schölermann B., Xie J., Sievers S., Ziegler S., Waldmann H. (2022). Chem.–Eur. J..

[cit30] RDKit: open-source cheminformatics, http://www.rdkit.org

[cit31] Sauer W. H. B., Schwarz M. K. (2003). J. Chem. Inf. Comput..

[cit32] Ertl P., Roggo S., Schuffenhauer A. (2008). J. Chem. Inf. Model..

[cit33] Bickerton G. R., Paolini G. V., Besnard J., Muresan S., Hopkins A. L. (2012). Nat. Chem..

