# Supplementary material for: A highly enantioselective intramolecular 1,3-dipolar cycloaddition yields novel pseudo-natural product inhibitors of the Hedgehog signalling pathway
Source: Chem Sci. 2023 Jun 21;14(29):7936–43. doi: 10.1039/d3sc01240a (PMC10370549; doi:10.1039/d3sc01240a)
Supplement: SC-014-D3SC01240A-s001 [file SC-014-D3SC01240A-s001.pdf]

Electronic Supplementary Material (ESI) for Chemical Science.
This journal is © The Royal Society of Chemistry 2023

Table of Contents

1. Supplementary figures and tables 2

Table S1. Primary condition screening for intramolecular 1,3-dipolar cycloaddition. 2

Table S2. SAR study of Hedgehog signalling pathway inhibitor. 3

Figure S1. Structural determination of *rac*-**3j** and **3l**. 4

Figure S2. Proposed binding mode of *ent*-**3a** in complex with SMO. 5

Figure S3. Prediction of drug-like properties for *ent*-3a (a) and vismodegib (b). 5

Figure S4. Cheminformatic analysis of *ent*-**3a** and reported SMO antagonists. 6

2. Biology experiment 7

2.1 Cell culture 7

2.2 Hedgehog-dependent osteoblast differentiation assay (ODA) 7

2.3 Gli-responsive reporter-gene assay 8

2.4 Reverse transcription quantitative PCR (RT-qPCR) 8

2.5 Smoothened binding assay 9

3. Structural determination 11

3.1 X-ray crystallography for *rac*-**3j** 11

3.2 Computed VCD spectrum for **3l** 12

4. Chemical synthesis 15

4.1 General information 15

4.2 Experimental details and analytical data 16

4.2.1 Starting material preparation 16

4.2.2 Asymmetric synthesis of pyrrolo[3,2-*c*]quinolines 27

5. Cheminformatic analysis 42

6. Molecular docking for *ent*-3a 67

7. Compound spectra (NMR and chiral HPLC spectra) 68

8. References 158

1. Supplementary figures and tables

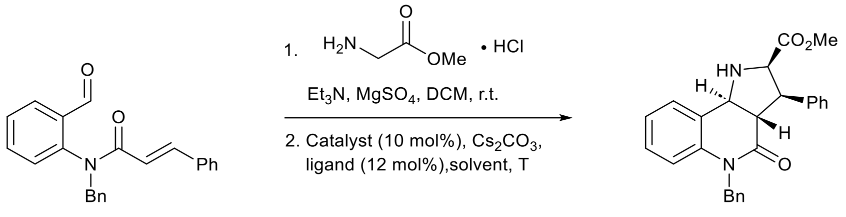

Entry	Catalyst	Ligand	Solvent	T [°C]	Yield[a] [%]	*ee*[b] [%]
1	AgOAc	L1	CH_2Cl_2	r.t.	89	75
2	AgOAc	L1	DCE	r.t.	55	73
3	AgOAc	L1	THF	r.t.	90	79
4	AgOAc	L1	Et_2O	r.t.	81	77
5	AgOAc	L1	PhMe	r.t.	81	73
6	AgOAc	L1	CH_3CN	r.t.	73	69

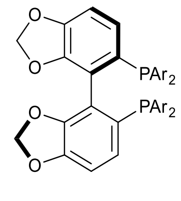

L1 Ar=Ph

Table S1. Primary condition screening for intramolecular 1,3-dipolar cycloaddition.
Unless otherwise specified, aldehyde (0.05 mmol, 1.0 equiv.), $MgSO_4$ (3.0 equiv.) and Et_3N (3.0 equiv.) were used for iminoester formation. Then, catalyst (10 mol%), ligand (12 mol%), Cs_2CO_3 (20 mol%) in solvent (1.0 mL). [a] Isolated yield after column chromatography. [b] The *ee* was determined by chiral HPLC.

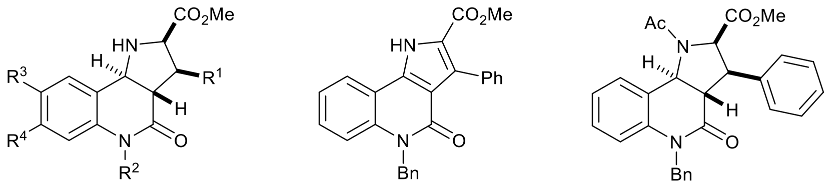

rac - **3** **5a** *rac*-**3a**-Ac

Entry	Compound	R[1]	R[2]	R[3]	R[4]	ODA IC50[a]	Cell viability [%][b]
1	*rac*-**3a**	Ph	Bn	H	H	0.4 ± 0.1 µM	112
2	*rac*-**3b**	*p*-ClC_6H_4	Bn	H	H	4.0 ± 0.5 µM	87
3	*rac*-**3c**	*m*-ClC_6H_4	Bn	H	H	6.6 ± 2.0 µM	94
4	*rac*-**3d**	*o*-ClC_6H_4	Bn	H	H	0.9 ± 0.3 µM	100
5	*rac*-**3e**	Ph	*o*-Me Benzyl	H	H	1.0 ± 0.2 µM	90
6	*rac*-**3f**	Ph	*m*-Br Benzyl	H	H	1.8 ± 0.1 µM	101
7	*rac*-**3g**	Ph	*p*-Cl Benzyl	H	H	1.3 ± 0.2 µM	112
8	*rac*-**3h**	Ph	Me	H	H	inactive	104
9	*rac*-**3i**	Ph	Bn	F	H	0.9 ± 0.3 µM	104
10	*rac*-**3j**	Ph	Bn	Cl	H	1.2 ± 0.2 µM	93
11	*rac*-**3k**	Ph	Bn	Br	H	7.6 ± 0.1 µM	104
12	*rac*-**3l**	Ph	Bn	Me	H	5.0 ± 0.2 µM	98
13	*rac*-**3m**	Ph	Bn	MeO	H	2.9 ± 0.3 µM	97
14	*rac*-**3n**	Ph	Bn	CF_3O	H	2.9 ± 0.5 µM	90
15	5a	-	-	-	-	2.2 ± 0.2 µM	77
16	*rac*-**3a-Ac**	-	-	-	-	inactive	89

Table S2. SAR study of Hedgehog signalling pathway inhibitor.
(a) Half maximum inhibitory concentration in a Hedgehog-dependent osteoblast differentiation assay (ODA) monitoring the reduction of alkaline phosphatase for scaffold **3** and **5** with different substituents. (b) Cell viability as % control with 10 µM compound treated in parallel cell viability assays.

(a)

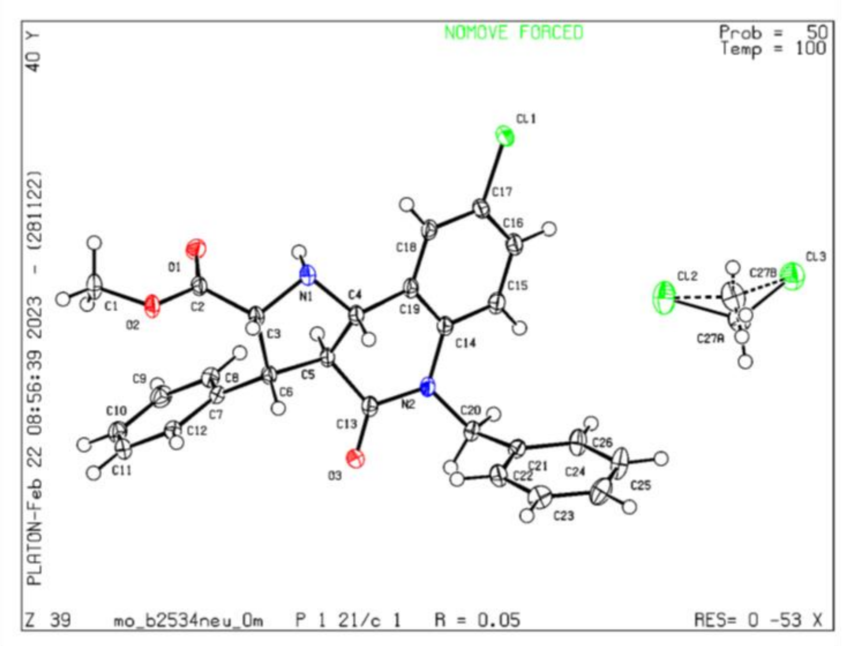

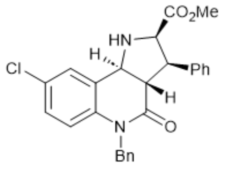

rac-**3j**

(b)

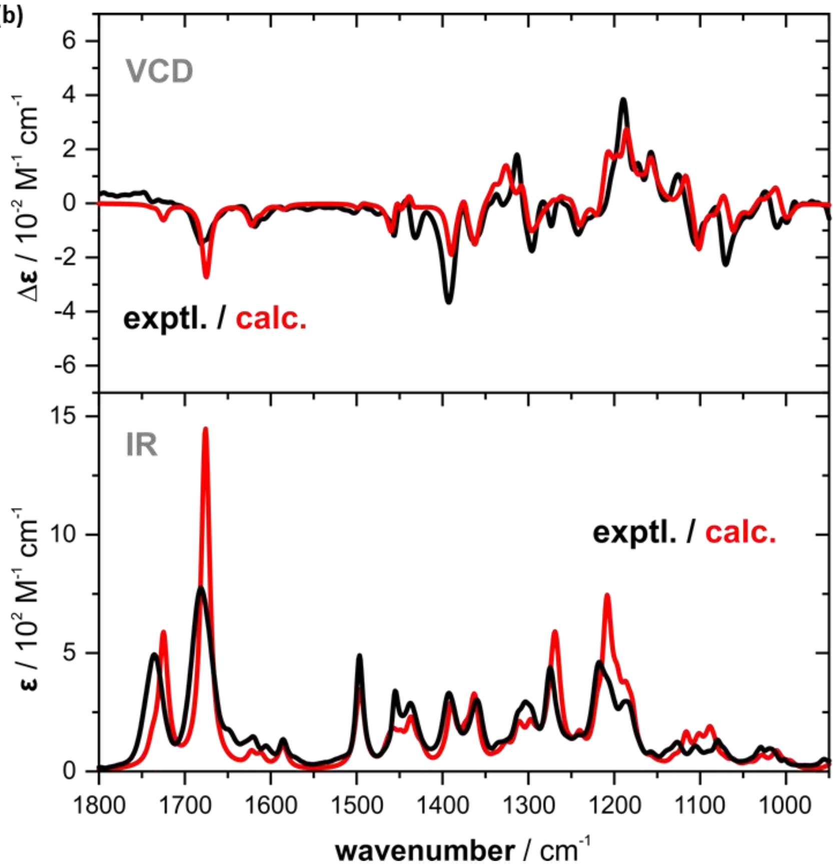

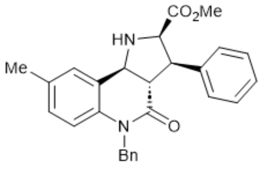

3l

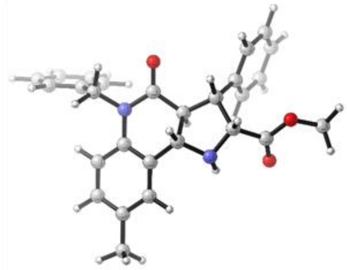

Figure S1. Structural determination of *rac*-**3j** and **3l**.
(a) Crystal structure for *rac*-**3j.** Crystallographic data for compound *rac*-**3j** have been deposited in the Cambridge Crystallographic Data Centre under accession number CCDC 2243515. (b) Comparison of the experimental and computed IR and VCD spectra (left) and lowest energy conformation of **3l** showing the correct stereochemistry.

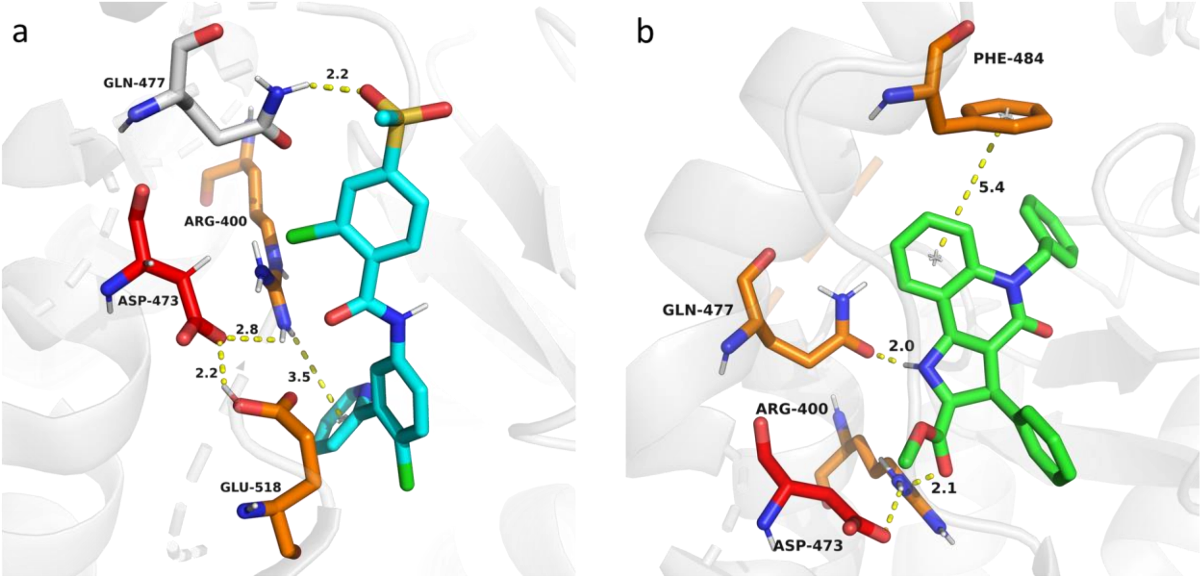

Figure S2. Proposed binding mode of *ent*-**3a** in complex with SMO.
(a) Binding mode of vismodegib in complex with SMO (PDB: 5L7I). (b) Proposed binding mode of *ent*-**3a** in complex with SMO.

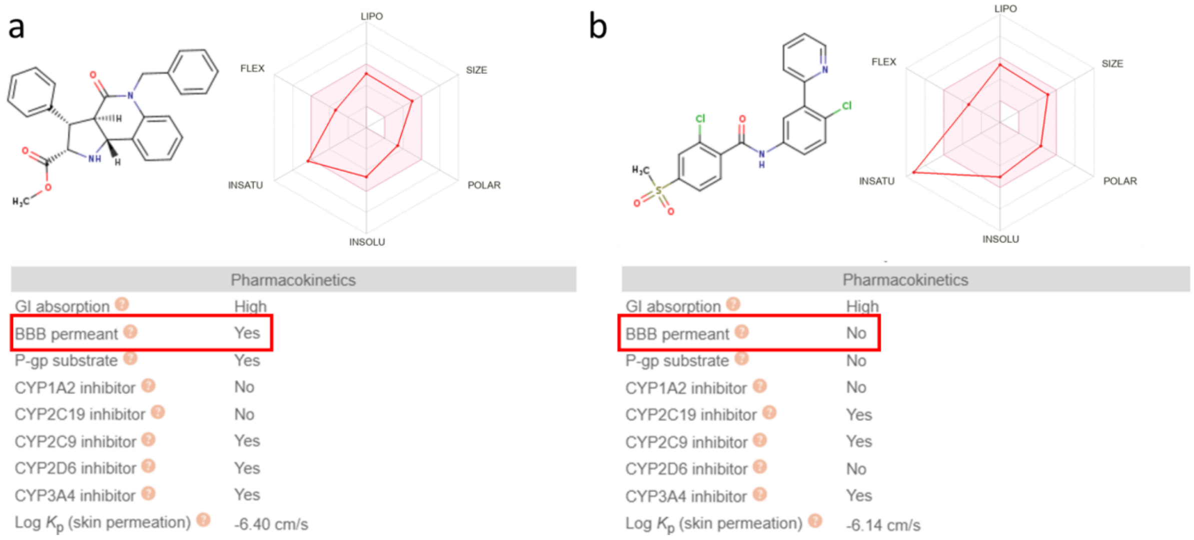

Figure S3. Prediction of drug-like properties for *ent*-**3a** (a) and vismodegib (b).

(a)

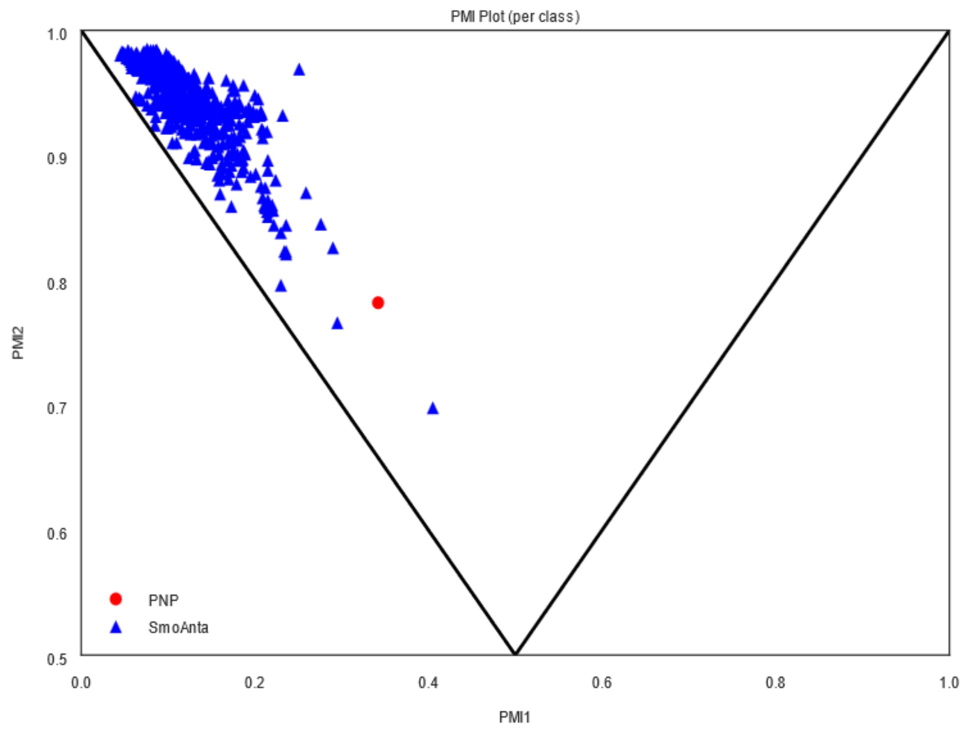

(b)

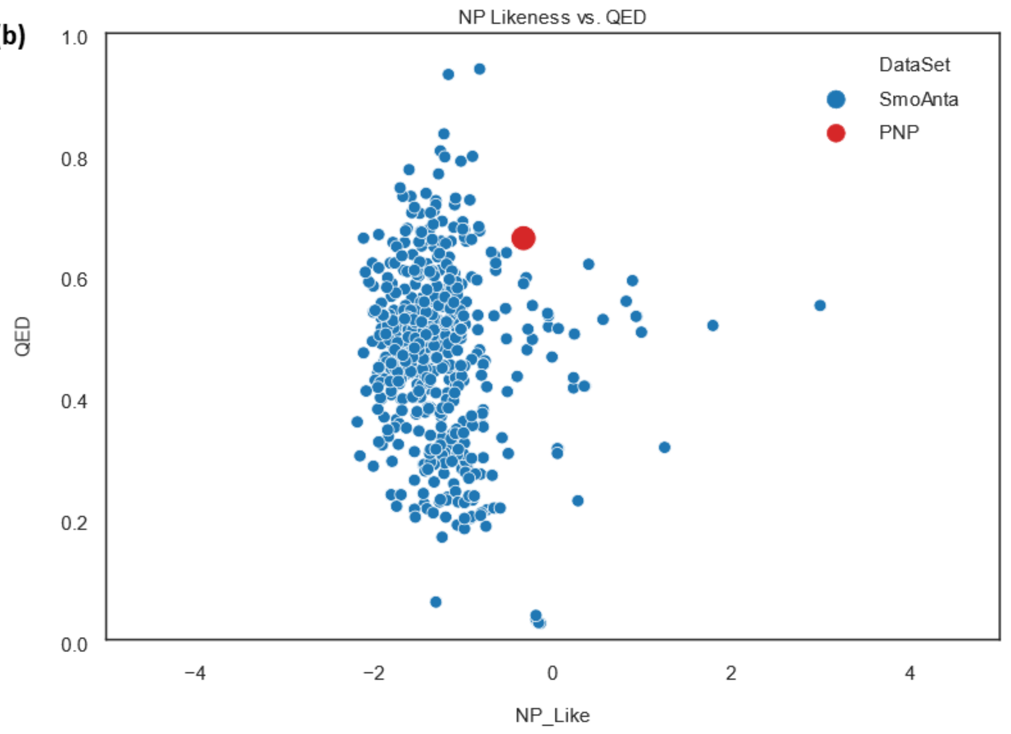

Figure S4. Cheminformatic analysis of *ent*-**3a** and reported SMO antagonists.
(a) Principal moments of inertia (PMI) plot of the shape of the pseudo-natural product (PNP) *ent*-**3a** (red) and reported SMO antagonists (blue). (b) NP-likeness score and quantitative estimation of drug-likeness (QED) for *ent*-**3a** (red) and reported SMO antagonists (blue).

2. Biology experiment

2.1 Cell culture

Experiments with mammalian cells were performed in a sterile environment in cell culture-approved clean benches with sterile equipment and media. All cell lines were cultured in a humidified atmosphere at 37 °C and 5% CO_2. The C3H10T1/2 cell line (ATCC, CCL-226) is a murine mesenchymal stem cell line. The cells were cultured in Dulbecco's Modified Eagle's medium (DMEM with 4.5 g/L glucose, L-glutamine and 3.7 g/L sodium bicarbonate; PAN Biotech, #P04-03550) supplemented with 10% of Foetal Bovine Serum Australian Origin (FBS, CellSera Australia #AU-FBS/PG, heat inactivated), 1 mM sodium pyruvate (PAN, #P04-43100). Shh-LIGHT2[1] cells are murine fibroblast cells (NIH/3T3 cells) which are stably transfected with a GLI-responsive firefly luciferase reporter plasmid[2] and a pRL-TK constitutive *Renilla* luciferase expression vector (Promega). The cells were cultured in Dulbecco's Modified Eagle's medium (DMEM with 4.5 g/L glucose, L-glutamine and 3.7 g/L sodium bicarbonate; PAN Biotech, #P04-03550) supplemented with 10% of Foetal Bovine Serum Australian Origin (FBS, CellSera Australia #AU-FBS/PG, heat inactivated), 1 mM sodium pyruvate (PAN, #P04-43100). Additionally, 400 µg/ml geneticin (Sigma Aldrich, #A1720) and 150 µg/ml zeocin (InvitroGen, #R25001) were added to the medium as selecting agents. The human kidney cell line HEK293T (ATCC, CRL-11268) was cultured in Dulbecco's Modified Eagle's medium (DMEM with 4.5 g/L glucose, L-glutamine and 3.7 g/L sodium bicarbonate; PAN Biotech, #P04-03550) supplemented with 10% of Foetal Bovine Serum (FBS, Fisher Scientific, #10270106), 1 mM sodium pyruvate (PAN, #P04-43100) and 1% MEM-non-essential amino acids (PAN, #P08-32100). Mycoplasma contaminations were checked on a regular basis, and cells were found to be free of contaminations at all times.

2.2 Hedgehog-dependent osteoblast differentiation assay (ODA)

The initial screening for racemic products was performed by the Compound Management and Screening Center (COMAS) in Dortmund, Germany in 384 well format. Eight hundred C3H10T1/2 cells per well were seeded in 25 µl medium (high glucose DMEM, 10% heat inactivated foetal calf serum, 1 mM sodium pyruvate, 6 mM L-glutamine, 100 U/ml penicillin and 0.1 mg/ml streptomycin) and allowed to grow overnight. Compounds were subsequently added to a final concentration of 10 µM using the acoustic nanoliter dispenser ECHO 520 (Beckman). After one hour, 10 µl of purmorphamine in medium were added to a final concentration of 1.5 µM using Multidrop Combi (Thermofisher Scientific) to activate the Hedgehog signalling; control cells did not receive purmorphamine. After four days, the cell culture medium was aspirated using the aspiration function of the Elx405 cell washer (Biotek) and 25 µl of a commercial luminogenic ALK substrate (CDP-Star, Roche) were added. After one hour, luminescence was read. To identify and exclude toxic compounds that also lead to a reduction in the luminescent signal, cell viability measurements were carried out in parallel. The cell viability

assay followed the same workflow, except that only 200 cells per well were seeded. Cell culture medium alone served as control for the cell viability assay. For the measurement of cell viability, 15 µl of CellTiterGlo reagent (Promega) which determines the cellular ATP content were added after aspiration of the medium. Hits were scored as showing at least a 50% reduction in the luminescent signal in the Hh assay, and a minimum of 80% cell viability. Dose response analysis for hit compounds was done using a three-fold dilution curve starting from 10 µM. IC_{50} values were calculated using the Quattro software suite (Quattro Research GmbH).

In manual ODA assay, six thousand C3H10T1/2 cells per well were seeded in white 96-well plates with a clear flat bottom (Greiner Bio-One, # 655098) and incubated in 5% CO_2 at 37 °C overnight. The cells were then treated with 1.5 µM purmorphamine (Cayman Chemical #10009634) and different concentration of the compounds or DMSO (< 0.5%) as a control. The plate was sealed with gas permeable membrane and incubated in 5% CO_2 at 37 °C. After 96 h, the cell culture medium was aspirated and 50 µL per well lysis buffer (100mM Tris pH 9.5, 250 mM NaCl, 25 mM $MgCl_2$ and 1% Triton X-100) containing luminogenic the luminogenic alkaline phosphatase (ALP) substrate CDP-Star (Roche, #11685627001) 1:100 dilution was added and incubated for 1 h at room temperature with gentle shaking in dark. Afterwards the luminescence signal was measured using the Spark® plate reader (Tecan). The alkaline phosphatase activity of cells that were treated with DMSO and purmorphamine was set to 100%. Calculations of the IC_{50} values were conducted using the GraphPad Prism 9 (GraphPad Software, USA).

2.3 Gli-responsive reporter-gene assay

Twenty-five thousand Shh-LIGHT2 cells per well were seeded in 96-well plates (Sarstedt, #83.3924) and incubated in 5% CO_2 at 37 °C overnight. The cells were incubated for 48 h with 2 µM purmorphamine to activate Hh signalling and different concentrations of the compounds or DMSO (< 0.5%) as a control, in serum-reduced medium (0.5% FBS). Then, the Dual-Luciferase Reporter Assay System (Promega, #E1960) was used to detect the expression and activity of firefly and *Renilla* luciferases using the Spark® plate reader (Tecan). The *Renilla* luciferase signal was used to normalise the firefly luciferase signal. The normalised activity ratio of the cell treated with DMSO and purmorphamine was set to 100%. Calculations of the IC_{50} values were conducted using the GraphPad Prism 9 (GraphPad Software, USA).

2.4 Reverse transcription quantitative PCR (RT-qPCR)

Sixty thousand C3H10T1/2 cells were seeded into 12-well plates and incubated at 37 °C in 5% CO_2 for 48 hours to achieve 80% cell confluency. The cells were then treated with 1.5 µM purmorphamine to

activate Hh signalling, and different concentrations of the compounds or DMSO (< 0.5%) as control for 96 h. Afterwards, the total RNA was isolated using the RNAeasy Kit (Qiagen, #74104) including DNase digestion step. The RNA concentration was measured using NanoDrop 2000 (Thermo Scientific). The QuantiTect Reverse Transcription Kit (Qiagen #205313) was used to generate cDNA. The relative mRNA amount of the Hedgehog target genes *Ptch1*[3] and *Gli1*[4] and the reference genes *Gapdh* and *Ap3dl* were assessed using SsoAdvanced™ Universal SYBR® Green Supermix, template cDNA, and primers (custom DNA Oligos, Sigma Aldrich). The SYBR Green signal was detected using the CFX96 Real-Time PCR Detection System (Bio-Rad, Germany) and relative gene expression levels were calculated using the $^{\Delta\Delta}$Ct method[5] with *Gapdh* and *Ap3dl* as reference genes. Gene expression levels in DMSO and purmorphamine-treated samples were set to 100%, whereas *Ptch1* and *Gli1* expression levels in compound-treated samples were related to the respective positive control.

Used primers:

	Forward (5'-3')	Reverse (5'-3')
Ptch1	CTCTGGAGCAGATTTCCAAGG	TGCCGCAGTTCTTTTGAATG
Gli1	CACCGTGGGAGTAAACAGGCCTTCC	CCAGAGCGTTACACACCTGCCCTTC
Gapdh	CAGTGCCAGCCTCGTC	CAATCTCCACTTTGCCACTG
Ap3dl	CAGAGGGCTCATCGGTACAC	GCCGGAAGTCCAACTTCTCA

2.5 Smoothened binding assay

Fifty thousand HEK293T cells per well were seeded on poly-D-lysine-coated coverslips (Neuvitro, 12 mm, #GG-12-1.5-PDL) placed in a 24-well plate and incubated at 37 °C in 5% CO_2 overnight. The cells were transfected with the SMO-expressing plasmid pGEN-mSMO (pGEN-mSmo was a gift from Philip Beachy (Addgene plasmid #37673; http://n2t.net/addgene:37673; (RRID:Addgene_37673))[1] in OptiMEM medium using FuGENE® HD transfection reagent (Promega, #E2311) according to the manufacturer's protocol. Cells were incubated at 37 °C in 5% CO_2 for 48 h. Afterwards the cells were washed once with PBS, fixed with 3.7 % paraformaldehyde in PBS for 10 min at room temperature and subsequently treated with 0.3% Triton X-100 in PBS for 5 min. The fixed cells were washed three times with PBS before treated with the compounds, vismodegib (Selleckchem #1082) and DMSO in DMEM containing 0.5% FBS (assay medium) and 5 nM BODIPY-cyclopamine S26 (Carbosynth Limited, FB18988) for four hours at room temperature in the dark. After that, cover slips were washed with PBS and incubated for 10 min at room temperature with 1 g/ml 4',6 diamidino-2-phenylindole (DAPI, Sigma Aldrich, Roche, #10236276001) in PBS. Cover slips were then washed again with PBS and mounted

onto glass slides using Aqua Polymount (Polysciences). Zeiss Observer Z1 microscope (Carl Zeiss, Germany) was used to acquire the images using a Plan-Apochromat 63x/1.40 Oil DIC M27 objective.

3. Structural determination

3.1 X-ray crystallography for *rac*-**3j**

CCDC No.	2243515
Identification code	mo_B2534neu_0m
Empirical formula	$C_{27}H_{25}Cl_3N_2O_3$
Formula weight	531.84
Temperature/K	100.00
Crystal system	monoclinic
Space group	$P2_1/c$
a/Å	13.9309(8)
b/Å	15.4866(10)
c/Å	11.3531(8)
α/°	90
β/°	93.315(3)
γ/°	90
Volume/Å^3	2445.2(3)
Z	4
ρ_{calc}g/cm^3	1.445
μ/mm^{-1}	0.408
F(000)	1104.0
Crystal size/mm^3	0.244 × 0.232 × 0.217
Radiation	MoKα (λ = 0.71073)
2Θ range for data collection/°	3.936 to 56.758
Index ranges	-18 ≤ h ≤ 18, -20 ≤ k ≤ 20, -15 ≤ l ≤ 15
Reflections collected	47360
Independent reflections	6102 [R_{int} = 0.0731, R_{sigma} = 0.0374]
Data/restraints/parameters	6102/0/331
Goodness-of-fit on F^2	1.058
Final R indexes [I>=2σ (I)]	R_1 = 0.0454, wR_2 = 0.0928
Final R indexes [all data]	R_1 = 0.0635, wR_2 = 0.1028
Largest diff. peak/hole / e Å^{-3}	0.34/-0.41

3.2 Computed VCD spectrum for **3l**

Experimental details. The IR and VCD spectrum were recorded on a Bruker Vertex 70/PMA 50 VCD spectrometer at 4 cm^{-1} spectral resolution by accumulating 32 scans for the IR and ~100000 scans (23 h accumulation time) for VCD. The sample was dissolved in $CDCl_3$ at a concentration of 0.11 M and measured using a BaF_2 IR cell with 100 µm optical path length. Baseline correction of the VCD spectra was done by subtraction of the spectra of the solvent recorded under identical conditions.

Computational details. Deriving the absolute configuration from the experimental spectra requires the computation of IR and VCD spectra. Therefore, a conformational sampling was carried out based on a Monte-Carlo (MC) algorithm on force-field level (MMFF).[6, 7] All so-obtained 12 conformers were subjected to further geometry optimisations at B3LYP/6-31+G(2d,p)/IEFPCM($CHCl_3$) level of theory using Gaussian 09 Rev E.01.[8] For the final comparison with the experiment, the IR and VCD spectra were simulated from the single-conformer spectra using the ΔE_{ZPC}-based Boltzmann weights and by assigning a Lorentzian band shape with half-width at half-height of 6 cm^{-1} to the computed dipole and rotational strength. Only three conformers were found to be notably populated: c1 with 51.2 %, c2 with 32.7 % and c3 with 5.6 %.

Cartesian coordinates of c1 and c2

C1

C	-2.17141200	3.88119800	0.69500700
C	-3.26306300	3.01351300	0.65513900
C	-3.15740900	1.72562400	0.12268500
C	-1.94139800	1.27417800	-0.39353000
C	-0.82816300	2.14367400	-0.36672200
C	-0.95481900	3.41883500	0.17012400
N	-1.82140400	-0.02319600	-0.97961500
C	-0.60109100	-0.66040300	-1.13776500
C	0.55745800	0.11572400	-0.55456100
C	0.43560000	1.59153200	-0.95402000
O	-0.49589600	-1.74454200	-1.69702100
C	1.98792000	-0.26252300	-0.96369300
C	2.71879400	1.17621300	-0.91493300
N	1.71032700	2.19376800	-0.57497100
C	2.62438300	-1.38833000	-0.17369200
C	3.24102100	-2.44591700	-0.85489100
C	3.83939700	-3.49978800	-0.16379300
C	3.83129900	-3.51559700	1.23145200
C	3.21983600	-2.46951900	1.92446200
C	2.62241100	-1.41783800	1.22881000
C	-3.82539600	-1.48292500	-0.48112500
C	-3.22343200	-2.14044400	0.59648300
C	-3.98942600	-2.89846400	1.48342900
C	-5.36878300	-3.01365400	1.30266400
C	-5.97767100	-2.36206700	0.22893800
C	-5.20956900	-1.60099100	-0.65327000
C	-3.01778900	-0.69457100	-1.49977400
C	3.88482200	1.26304000	0.05011100
O	3.81618900	1.73145800	1.16657300
O	4.99997300	0.76000000	-0.48496400
C	6.16789100	0.73538300	0.36362500
C	-2.28002500	5.26652700	1.28471600
H	-4.22081100	3.33593600	1.05308400
H	-4.02602600	1.07977100	0.13107700
H	-0.08662900	4.07172800	0.16949800
H	0.45258500	0.06984100	0.53795700
H	0.36170400	1.64289900	-2.05354500
H	1.97838800	-0.56621000	-2.01312700
H	3.11173300	1.36998000	-1.91647100
H	1.74015900	2.33805800	0.43468500
H	3.24512200	-2.44706100	-1.94104300
H	4.30579600	-4.30936600	-0.71651100
H	4.29204900	-4.33547200	1.77320000
H	3.20442100	-2.47038500	3.00994700
H	2.15790500	-0.61567300	1.79345800
H	-2.15201500	-2.06448300	0.74696000
H	-3.50565000	-3.40140300	2.31494400
H	-5.96339300	-3.60311600	1.99318100
H	-7.05012400	-2.43991500	0.07988100
H	-5.69237000	-1.09104500	-1.48290600
H	-2.66886600	-1.37237400	-2.28249500
H	-3.65131300	0.06050700	-1.97264800
H	6.95194400	0.27962000	-0.23889400
H	6.44395700	1.75180700	0.65113100
H	5.97087000	0.13845200	1.25603200
H	-3.31437800	5.50646600	1.54614400
H	-1.67495300	5.35977600	2.19448700
H	-1.92302200	6.02788200	0.58212300

C2

C	-2.34880900	3.50278600	0.77499500
C	-3.17892300	2.62172400	1.46756300
C	-2.96465000	1.24083800	1.44609700
C	-1.89804600	0.70203400	0.72448300
C	-1.05476900	1.58227900	0.00959700
C	-1.28563600	2.95170200	0.04374100
N	-1.66921600	-0.70806200	0.67019500
C	-0.43460900	-1.24897000	0.34023600
C	0.63342200	-0.20587300	0.10551600
C	0.06012100	0.93055200	-0.74987100
O	-0.24554100	-2.45543400	0.25097400
C	1.91833900	-0.61162400	-0.63105900
C	2.27762300	0.75461400	-1.41197700
N	1.21471300	1.73552900	-1.13681300
C	3.00671800	-1.22362100	0.22761800
C	3.60572100	-2.42588900	-0.17035900
C	4.61129700	-3.01985000	0.59278200
C	5.03894200	-2.41799000	1.77679200
C	4.45126600	-1.22015800	2.18702500
C	3.44563000	-0.63029800	1.42053000
C	-3.92734200	-1.63561200	0.08349700
C	-3.74255300	-1.59552800	-1.30323000
C	-4.83512100	-1.65754800	-2.16803300
C	-6.13073900	-1.76618400	-1.65787200
C	-6.32435600	-1.80942000	-0.27686100
C	-5.22836100	-1.74131700	0.58553000

C	-2.74684700	-1.63494900	1.03694000
C	3.62943800	1.34780500	-1.06620900
O	3.79829100	2.25361600	-0.27779100
O	4.61295700	0.74842800	-1.74177800
C	5.95672200	1.18904600	-1.45066100
C	-2.57664700	4.99469600	0.80066800
H	-4.01614600	3.00989600	2.04020300
H	-3.64139300	0.60204700	1.99757600
H	-0.62582200	3.60449800	-0.52060400
H	0.87851100	0.22182200	1.08725500
H	-0.35513000	0.48848400	-1.67131000
H	1.65857100	-1.34456400	-1.39864500
H	2.29137100	0.51910500	-2.47941400
H	1.51668600	2.31204300	-0.35103600
H	3.27386500	-2.90659300	-1.08609600
H	5.05498300	-3.95444800	0.26393100
H	5.81816100	-2.87856500	2.37569400
H	4.77303800	-0.74230900	3.10715500
H	3.00944500	0.30352900	1.76048700
H	-2.73949300	-1.51557600	-1.71023300
H	-4.67399500	-1.62399600	-3.24106000
H	-6.98074100	-1.81404300	-2.33099100
H	-7.32689800	-1.88955100	0.13162300
H	-5.38815100	-1.77044000	1.66015900
H	-3.08457500	-1.42042400	2.05537100
H	-2.28708100	-2.62451800	1.05514600
H	6.60238000	0.57958800	-2.08088300
H	6.06607100	2.24738800	-1.69552000
H	6.18387300	1.02736800	-0.39525700
H	-3.47577400	5.24855600	1.36888500
H	-1.73017800	5.51880800	1.25988000
H	-2.69268200	5.39727600	-0.21208200

4. Chemical synthesis

4.1 General information

Unless otherwise noted, all commercially available compounds were used as provided without further purifications. Solvents for chromatography were technical grade. Analytical thin-layer chromatography (TLC) was performed on Merck silica gel aluminium plates with F-254 indicator. Compounds were visualised by irradiation with UV light or potassium permanganate staining. Column chromatography was performed using silica gel Merck 60 (particle size 0.040-0.063 mm) or aluminium oxide (activated, neutral, Brockmann I, Sigma-Aldrich).

^{1}H-NMR and ^{13}C-NMR were recorded on a *Bruker DRX400* (400 MHz), *Bruker DRX500* (500 MHz), *INOVA500* (500 MHz) and *Bruker DRX700* (700 MHz) using CD_2Cl_2, $CDCl_3$ or DMSO-*d6* as solvent. Data are reported in the following order: chemical shift (δ) values are reported in ppm with the solvent resonance as internal standard ($CDCl_3$: δ = 7.26 ppm for ^{1}H, δ = 77.16 ppm for ^{13}C); multiplicities are indicated br s (broadened singlet), s (singlet), d (doublet), t (triplet), q (quartet), m (multiplet); coupling constants (*J*) are given in Hertz (Hz).

High resolution mass spectra were recorded on a *LTQ Orbitrap* mass spectrometer coupled to an *Accela HPLC*-System (HPLC column: *Hypersyl GOLD*, 50 mm x 1 mm, particle size 1.9 µm, ionisation method: electron spray ionisation).

Data collection for single crystal X-ray structure analyses was conducted on a Bruker D8 VENTURE area detector diffractometer. The crystal was kept at 100.0 K during data collection. Using Olex2[9], the structures were solved with the ShelXT[10] structure solution program using Intrinsic Phasing and refined with the SHEXL[11] refinement package using Least Squares minimisation.

4.2 Experimental details and analytical data

4.2.1 Starting material preparation

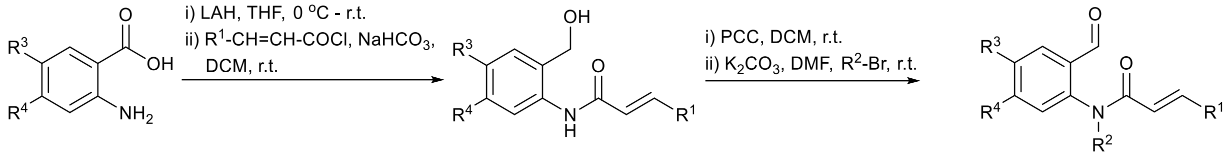

S1 **S2** **4**

General procedure A for the synthesis of 4: Commercially available **S1** (1.0 equiv.) was dissolved in dry THF (0.3 M) and cooled in an ice bath, followed by the addition of LAH (1.5 equiv., 1.0 mol/L in THF). The whole reaction was stirred for 30 min in ice bath before warming to room temperature. $Na_2SO_4{\bullet}10H_2O$ was added in small portions to quench the reaction. Then the reaction mixture was filtered and the filtrate was dried by anhydrous Na_2SO_4. The residue was concentrated and used for the next step without any other purification.

The alcohol from the previous step was dissolved in dry DCM (0.15 M). Then R^1-CH=CH-COCl (1.1 equiv.) was added followed by $NaHCO_3$ (5.0 equiv.). The reaction was stirred overnight and quenched by the addition of water. Then the reaction mixture was extracted by ethyl acetate (30 mL x 3), followed by drying and concentration. The residue was purified by flash column chromatography.

The amide **S2** from the previous step was dissolved in DCM (0.05 M) followed by the addition of PCC (1.50 equiv.) and stirred overnight. The reaction mixture was passed through a short pad of Celite and concentrated. The residue was then dissolved in DMF (0.1 M), followed by the addition of R^2-Br or MeI (2.0 equiv.) and K_2CO_3 (5.0 equiv.). The reaction was stirred overnight before the addition of water. Then the mixture was extracted by ethyl acetate (30 mL x 3), followed by washing, drying and concentration. The residue was purified on column. The yield was calculated based on **S1**.

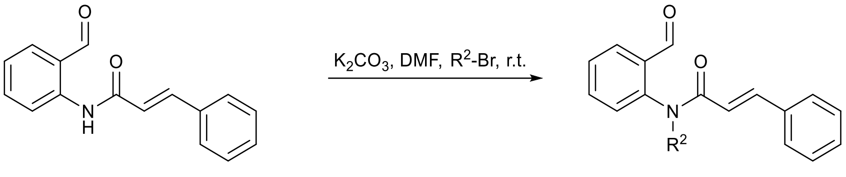

S3 **4**

General procedure B for the synthesis of 4: Aldehyde **S3** was synthesised according to the literature.[12] The compound **S3** was dissolved in DMF (0.1 M), followed by the addition of R^2-Br or MeI (2.0 equiv.) and K_2CO_3 (5.0 equiv.). The reaction was stirred overnight before the addition of water. Then the mixture was extracted by ethyl acetate (30 mL x 3), followed by washing, drying and concentration. The residue was purified on column. The yield was calculated based on **S3**.

***N*-(2-formylphenyl)-*N*-(2-methylbenzyl)cinnamamide (4e)**

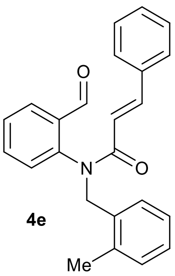

4e

The title product compound **4e** was prepared using general procedure B from aldehyde **S3** (125.6 mg, 0.50 mmol) and isolated by column chromatography (4:1 *n*-pentane:EA) giving a solid (159.3 mg, 0.45 mmol, 90% yield).
^{1}H NMR (400 MHz, CDCl$_3$) δ 9.69 (s, 1H), 7.92 (dd, *J* = 7.7, 1.7 Hz, 1H), 7.80 (d, *J* = 15.4 Hz, 1H), 7.62 (td, *J* = 7.6, 1.8 Hz, 1H), 7.52 (t, *J* = 7.5 Hz, 1H), 7.30 – 7.21 (m, 5H), 7.18 – 7.02 (m, 5H), 6.10 (d, *J* = 15.4 Hz, 1H), 5.20 (d, *J* = 14.2 Hz, 1H), 5.09 (d, *J* = 14.1 Hz, 1H), 2.12 (s, 3H). **^{13}C NMR (101 MHz, CDCl$_3$)** δ 188.8, 165.8, 144.1, 143.6, 137.1, 135.4, 134.8, 134.0, 133.9, 130.9, 130.7, 130.4, 130.1, 129.3, 129.2, 128.8, 128.3, 128.1, 126.3, 117.7, 50.9, 19.2. **HRMS(ESI):** $[M+H]^+$ calcd. $C_{24}H_{22}O_2N$ m/z 356.1645, found 356.1646.

***N*-(3-bromobenzyl)-*N*-(2-formylphenyl)cinnamamide (4f)**

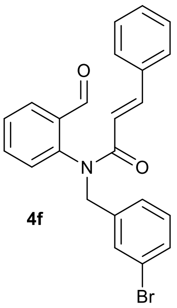

4f

The title product compound **4f** was prepared using general procedure B from aldehyde **S3** (125.6 mg, 0.50 mmol) and isolated by column chromatography (4:1 *n*-pentane:EA) giving a solid (165.5 mg, 0.40 mmol, 79% yield).
^{1}H NMR (400 MHz, CDCl$_3$) δ 9.79 (s, 1H), 7.98 (dd, *J* = 7.7, 1.7 Hz, 1H), 7.78 (d, *J* = 15.4 Hz, 1H), 7.66 (td, *J* = 7.6, 1.7 Hz, 1H), 7.59 – 7.54 (m, 1H), 7.43 – 7.37 (m, 2H), 7.29 – 7.21 (m, 5H), 7.17 – 7.11 (m, 3H), 6.08 (d, *J* = 15.4 Hz, 1H), 5.17 (d, *J* = 14.2 Hz, 1H), 4.85 (d, *J* = 14.2 Hz, 1H). **^{13}C NMR (101 MHz, CDCl$_3$)** δ 189.0, 166.1, 144.5, 143.4, 138.5, 135.5, 134.7, 133.5, 132.4, 131.3, 130.3, 130.3, 130.2, 130.1, 129.3, 128.9, 128.1, 122.7, 117.4, 53.6. **HRMS(ESI):** $[M+H]^+$ calcd. $C_{23}H_{19}O_2NBr$ m/z 420.0594, found 420.0598.

***N*-(4-chlorobenzyl)-*N*-(2-formylphenyl)cinnamamide (4g)**

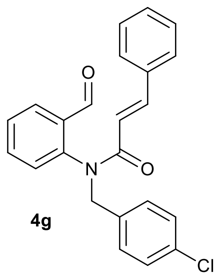

4g

The title product compound **4g** was prepared using general procedure B from aldehyde **S3** (125.6 mg, 0.50 mmol) and isolated by column chromatography (4:1 *n*-pentane:EA) giving a solid (177.5 mg, 0.47 mmol, 94% yield).

^{1}H NMR (400 MHz, $CDCl_3$) δ 9.78 (s, 1H), 7.98 (dd, *J* = 7.7, 1.7 Hz, 1H), 7.77 (d, *J* = 15.4 Hz, 1H), 7.66 (td, *J* = 7.7, 1.7 Hz, 2H), 7.56 (t, *J* = 7.5 Hz, 1H), 7.33 – 7.22 (m, 7H), 7.20 – 7.10 (m, 3H), 6.07 (d, *J* = 15.4, 1H), 5.15 (d, *J* = 14.1 Hz, 1H), 4.89 (d, *J* = 14.1 Hz, 1H). **^{13}C NMR (101 MHz, $CDCl_3$)** δ 189.0, 166.1, 144.4, 143.6, 135.5, 134.7, 134.1, 133.5, 131.0, 130.4, 130.2, 129.9, 129.3, 129.0, 128.9, 128.1, 117.5, 53.5. **HRMS(ESI):** $[M+H]^+$ calcd. $C_{23}H_{19}O_2NCl$ m/z 376.1099, found 376.1101.

***N*-(2-formylphenyl)-*N*-methylcinnamamide (4h)**

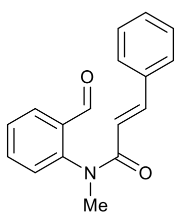

4h

The title product compound **4h** was prepared using general procedure B from aldehyde **S3** (125.6 mg, 0.50 mmol) and isolated by column chromatography (4:1 *n*-pentane:EA) giving a solid (112.2 mg, 0.42 mmol, 85% yield).

^{1}H NMR (400 MHz, $CDCl_3$) δ 10.13 (s, 1H), 8.03 (dd, *J* = 7.7, 1.7 Hz, 1H), 7.76 – 7.69 (m, 2H), 7.57 (t, *J* = 7.6 Hz, 1H), 7.36 – 7.23 (m, 6H), 6.13 (d, *J* = 15.5 Hz, 1H), 3.45 (s, 3H). **^{13}C NMR (101 MHz, $CDCl_3$)** δ 189.3, 166.5, 145.7, 143.5, 135.7, 134.9, 133.0, 130.0, 129.9, 129.6, 129.1, 128.8, 128.1, 117.7, 38.7. **HRMS(ESI):** $[M+H]^+$ calcd. $C_{17}H_{16}O_2N$ m/z 266.1176, found 266.1171.

***N*-benzyl-*N*-(4-fluoro-2-formylphenyl)cinnamamide (4i)**

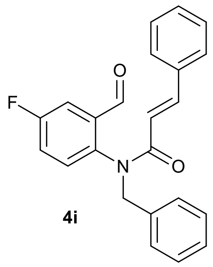

4i

The title product compound **4i** was prepared using general procedure A from 2-amino-5-fluorobenzoic acid (930.8 mg, 6.0 mmol) and isolated by column chromatography (4:1 *n*-pentane:EA) giving a solid (1.121 g, 3.1 mmol, 52% yield).

^{1}H NMR (400 MHz, $CDCl_3$) δ 9.51 (d, *J* = 2.9 Hz, 1H), 7.76 (d, *J* = 15.4 Hz, 1H), 7.62 – 7.50 (m, 1H), 7.34 – 7.10 (m, 12H), 6.03 (d, *J* = 15.4 Hz, 1H), 5.10 (d, *J* = 13.9 Hz, 1H), 4.92 (d, *J* = 13.9 Hz, 1H). **^{13}C NMR (101 MHz, $CDCl_3$)** δ 187.6 (d, *J* = 1.6 Hz), 166.0, 162.1 (d, *J* = 252.2 Hz), 144.6, 139.7 (d, *J* = 3.4 Hz), 135.6, 135.5 (d, *J* = 6.7 Hz), 134.5, 132.3 (d, *J* = 7.8 Hz), 130.2, 129.6, 128.8, 128.3, 128.1, 122.5 (d, *J* = 23.0 Hz), 117.2, 115.3 (d, *J* = 23.5 Hz), 54.2. **^{19}F NMR (377 MHz, $CDCl_3$)** δ -109.7 (tt, *J* = 7.8, 3.8 Hz). **HRMS(ESI):** $[M+H]^+$ calcd. $C_{23}H_{19}O_2NF$ m/z 360.1394, found 360.1395.

N-benzyl-N-(4-chloro-2-formylphenyl)cinnamamide (4j)

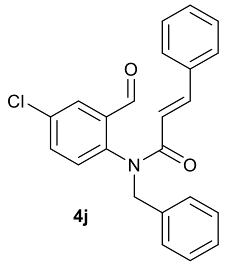

4j

The title product compound **4j** was prepared using general procedure A from 2-amino-5-chlorobenzoic acid (1.029 g, 6.0 mmol) and isolated by column chromatography (4:1 *n*-pentane:EA) giving a solid (90.2 mg, 0.24 mmol, 4% yield). **^{1}H NMR (400 MHz, CDCl$_3$)** δ 9.47 (s, 1H), 7.82 (d, *J* = 2.6 Hz, 1H), 7.72 (d, *J* = 15.3 Hz, 1H), 7.54 (dd, *J* = 8.4, 2.6 Hz, 1H), 7.26 – 7.14 (m, 8H), 7.14 – 7.09 (m, 2H), 7.05 (d, *J* = 8.4 Hz, 1H), 5.98 (d, *J* = 15.4 Hz, 1H), 5.06 (d, *J* = 14.0 Hz, 1H), 4.88 (d, *J* = 14.0 Hz, 1H). **^{13}C NMR (126 MHz, CDCl$_3$)** δ 187.6, 165.9, 144.9, 142.2, 135.7, 135.5, 135.4, 134.9, 134.6, 131.7, 130.3, 129.7, 129.1, 128.9, 128.9, 128.4, 128.2, 117.2, 54.2. **HRMS(ESI):** [M+H]$^+$ calcd. $C_{23}H_{19}O_2NCl$ m/z 376.1099, found 376.1104.

N-benzyl-N-(4-bromo-2-formylphenyl)cinnamamide (4k)

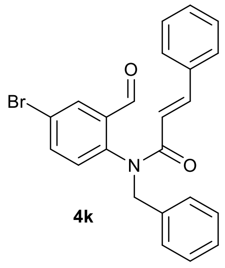

4k

The title product compound **4k** was prepared using general procedure A from 2-amino-5-bromobenzoic acid (1.296 g, 6.0 mmol) and isolated by column chromatography (4:1 *n*-pentane:EA) giving a solid (958.3 mg, 2.3 mmol, 38% yield).

^{1}H NMR (400 MHz, CDCl$_3$) δ 9.52 (s, 1H), 8.03 (d, *J* = 2.4 Hz, 1H), 7.84 – 7.72 (m, 2H), 7.31 – 7.22 (m, 8H), 7.20 – 7.15 (m, 2H), 7.04 (d, *J* = 8.4 Hz, 1H), 6.04 (d, *J* = 15.4 Hz, 1H), 5.11 (d, *J* = 13.9 Hz, 1H), 4.94 (d, *J* = 14.0 Hz, 1H). **^{13}C NMR (101 MHz, CDCl$_3$)** δ 187.5, 165.8, 144.9, 142.7, 138.3, 135.7, 135.1, 134.6, 132.2, 131.9, 130.3, 129.7, 128.9, 128.9, 128.4, 128.2, 123.3, 117.1, 54.2. **HRMS(ESI):** [M+H]$^+$ calcd. $C_{23}H_{19}O_2NBr$ m/z 420.0594, found 420.0599.

N-benzyl-N-(2-formyl-4-methylphenyl)cinnamamide (4l)

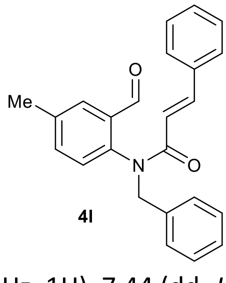

4l

The title product compound **4l** was prepared using general procedure A from 2-amino-5-methylbenzoic acid (1.814 g, 12 mmol) and isolated by column chromatography (4:1 *n*-pentane:EA) giving a solid (341.2 mg, 0.96 mmol, 8% yield).

^{1}H NMR (400 MHz, CDCl$_3$) δ 9.63 (s, 1H), 7.77 (d, *J* = 15.4 Hz, 1H), 7.74 (d, *J* = 2.2 Hz, 1H), 7.44 (dd, *J* = 8.0, 2.2 Hz, 1H), 7.30 – 7.18 (m, 10H), 7.04 (d, *J* = 8.0 Hz, 1H), 6.10 (d, *J* = 15.4 Hz, 1H), 5.07 (d, *J* = 13.9 Hz, 1H), 5.01 (d, *J* = 13.9 Hz, 1H), 2.46 (s, 3H). **^{13}C NMR (101 MHz, CDCl$_3$)** δ 189.3, 166.2, 144.0, 141.3, 139.3, 136.2, 136.2, 134.9, 133.3, 130.1, 130.0, 129.7, 129.6, 128.8, 128.8, 128.2, 128.1, 117.8, 54.2, 21.2. **HRMS(ESI):** [M+H]$^+$ calcd. $C_{24}H_{22}O_2N$ m/z 356.1645, found 356.1647.

***N*-benzyl-*N*-(2-formyl-4-methoxyphenyl)cinnamamide (4m)**

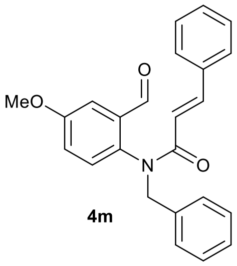

4m

The title product compound **4m** was prepared using general procedure A from 2-amino-5-methoxybenzoic acid (1.000 g, 6.0 mmol) and isolated by column chromatography (2:1 *n*-pentane:EA) giving a solid (1.158 g, 3.1 mmol, 52% yield).

^{1}H NMR (500 MHz, CDCl$_3$) δ 9.56 (s, 1H), 7.78 (d, *J* = 15.5 Hz, 1H), 7.41 – 7.37 (m, 2H), 7.31 – 7.25 (m, 7H), 7.23 – 7.17 (m, 3H), 7.08 (d, *J* = 8.7 Hz, 1H), 6.12 (d, *J* = 15.4 Hz, 1H), 5.13 (d, *J* = 13.8 Hz, 1H), 4.95 (d, *J* = 13.9 Hz, 1H), 3.91 (s, 3H). **^{13}C NMR (126 MHz, CDCl$_3$)** δ 189.0, 166.4, 159.6, 144.1, 136.8, 136.0, 134.8, 134.6, 131.4, 130.1, 129.8, 128.8, 128.8, 128.2, 128.1, 122.7, 117.6, 111.4, 55.9, 54.3. **HRMS(ESI):** [M+H]$^+$ calcd. $C_{24}H_{22}O_3N$ m/z 372.1594, found 372.1595.

***N*-benzyl-*N*-(2-formyl-4-(trifluoromethoxy)phenyl)cinnamamide (4n)**

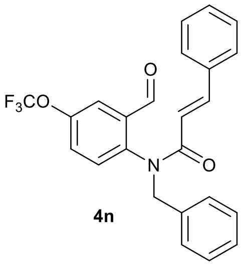

4n

The title product compound **4n** was prepared using general procedure A from 2-amino-5-(trifluoromethoxy)benzoic acid (1.000 g, 4.5 mmol) and isolated by column chromatography (2:1 *n*-pentane:EA) giving a solid (938.0 mg, 2.2 mmol, 49% yield).

^{1}H NMR (500 MHz, CDCl$_3$) δ 9.62 (s, 1H), 7.84 (d, *J* = 15.4 Hz, 1H), 7.80 (d, *J* = 3.0 Hz, 1H), 7.51 (dd, *J* = 8.6, 3.0 Hz, 1H), 7.35 – 7.21 (m, 11H), 6.08 (d, *J* = 15.4 Hz, 1H), 5.17 (d, *J* = 14.1 Hz, 1H), 5.01 (d, *J* = 14.0 Hz, 1H). **^{13}C NMR (126 MHz, CDCl$_3$)** δ 187.4, 165.9, 149.2 (q, *J* = 2.2 Hz), 144.9, 141.9, 135.6, 135.3, 134.5, 132.1, 130.3, 129.6, 128.9, 128.9, 128.4, 128.1, 127.2, 120.5, 120.4 (q, *J* = 259.4 Hz), 117.0, 54.2. **^{19}F NMR (470 MHz, CDCl$_3$)** δ -57.9 (s). **HRMS(ESI):** [M+H]$^+$ calcd. $C_{24}H_{19}O_3NF_3$ m/z 426.1312, found 426.1312.

***N*-benzyl-*N*-(5-fluoro-2-formylphenyl)cinnamamide (4o)**

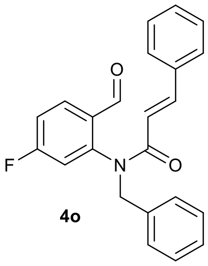

4o

The title product compound **4o** was prepared using general procedure A from 2-amino-4-fluorobenzoic acid (621.0 mg, 4.0 mmol) and isolated by column chromatography (4:1 *n*-pentane:EA) giving a solid (460.0 mg, 1.3 mmol, 32% yield).

^{1}H NMR (500 MHz, CDCl$_3$) δ 9.59 (s, 1H), 7.99 (dd, *J* = 8.8, 6.3 Hz, 1H), 7.83 (d, *J* = 15.4 Hz, 1H), 7.32 – 7.20 (m, 11H), 6.93 (dd, *J* = 8.9, 2.5 Hz, 1H), 6.12 (d, *J* = 15.4 Hz, 1H), 5.17 (d, *J* = 14.1 Hz, 1H), 5.01 (d, *J* = 14.1 Hz, 1H). **^{13}C NMR (126 MHz, CDCl$_3$)** δ 187.3, 166.2 (d, *J* = 259.8 Hz), 165.6, 145.9 (d, *J* = 10.5 Hz), 144.7, 135.6, 134.5, 131.6 (d, *J* = 10.5 Hz), 130.6 (d, *J* = 3.1 Hz), 130.2, 129.4, 128.8, 128.3, 128.0, 117.2 (d, *J* = 21.4 Hz), 117.1, 116.7 (d, *J* = 21.6 Hz), 54.0. **^{19}F NMR**

(470 MHz, $CDCl_3$) δ -99.9 (q, *J* = 7.7 Hz). **HRMS(ESI):** $[M+H]^+$ calcd. $C_{23}H_{19}O_2NF$ m/z 360.1394, found 360.1397.

***N*-benzyl-*N*-(5-bromo-2-formylphenyl)cinnamamide (4p)**

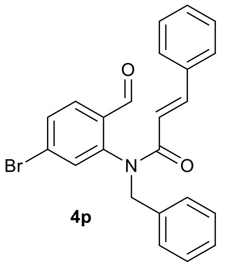

4p

The title product compound **4p** was prepared using general procedure A from 2-amino-4-bromobenzoic acid (864.0 mg, 4.0 mmol) and isolated by column chromatography (4:1 *n*-pentane:EA) giving a solid (285.8 mg, 0.68 mmol, 17% yield).

^{1}H NMR (400 MHz, $CDCl_3$) δ 9.50 (s, 1H), 7.83 – 7.77 (m, 2H), 7.70 – 7.65 (m, 1H), 7.40 (d, *J* = 1.8 Hz, 1H), 7.31 – 7.24 (m, 8H), 7.22 – 7.18 (m, 2H), 6.05 (d, *J* = 15.4 Hz, 1H), 5.27 (d, *J* = 14.0 Hz, 1H), 4.85 (d, *J* = 14.0 Hz, 1H). **^{13}C NMR (126 MHz, $CDCl_3$)** δ 187.9, 165.8, 145.0, 144.8, 135.5, 134.6, 133.1, 132.9, 132.6, 130.3, 130.3, 129.9, 129.7, 128.9, 128.9, 128.5, 128.2, 117.1, 54.2. **HRMS(ESI):** $[M+H]^+$ calcd. $C_{23}H_{19}O_2NBr$ m/z 420.0594, found 420.0595.

***(E)*-*N*-benzyl-3-(4-chlorophenyl)-*N*-(2-formyl-4-methylphenyl)acrylamide (4q)**

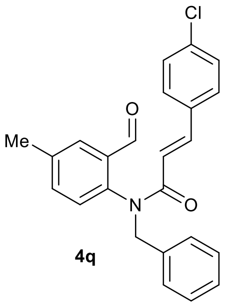

4q

The title product compound **4q** was prepared using general procedure A from 2-amino-5-methylbenzoic acid (1.814 g, 12 mmol) and isolated by column chromatography (4:1 *n*-pentane:EA) giving a solid (421.1 mg, 1.1 mmol, 9% yield). **^{1}H NMR (400 MHz, $CDCl_3$)** δ 9.67 (s, 1H), 7.78 (d, *J* = 1.8 Hz, 1H), 7.74 (d, *J* = 15.5 Hz, 1H), 7.50 (dd, *J* = 8.0, 1.7 Hz, 1H), 7.32 – 7.19 (m, 8H), 7.16 (d, *J* = 7.6 Hz, 1H), 7.09 (d, *J* = 8.0 Hz, 1H), 6.16 (d, *J* = 15.4 Hz, 1H), 5.15 – 5.03 (m, 2H), 2.49 (s, 3H). **^{13}C NMR (101 MHz, $CDCl_3$)** δ 189.1, 165.6, 142.2, 140.8, 139.4, 136.5, 136.2, 135.9, 134.6, 133.1, 129.9, 129.9, 129.8, 129.7, 129.5, 128.6, 128.0, 127.5, 126.3, 119.0, 54.1, 21.1. **HRMS(ESI):** $[M+H]^+$ calcd. $C_{24}H_{21}O_2NCl$ m/z 390.1255, found 390.1262.

***(E)*-*N*-benzyl-3-(3-chlorophenyl)-*N*-(2-formyl-4-methylphenyl)acrylamide (4r)**

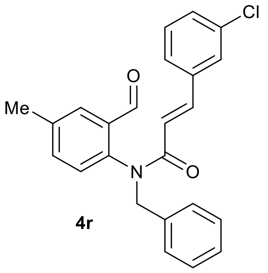

4r

The title product compound **4r** was prepared using general procedure A from 2-amino-5-methylbenzoic acid (1.814 g, 12 mmol) and isolated by column chromatography (4:1 *n*-pentane:EA) giving a solid (374.3 mg, 0.96 mmol, 8% yield).

^{1}H NMR (400 MHz, $CDCl_3$) δ 9.64 (s, 1H), 7.79 – 7.69 (m, 2H), 7.48 (dd, *J* = 8.0, 2.1 Hz, 1H), 7.33 – 7.16 (m, 9H), 7.07 (d, *J* = 8.0 Hz, 1H), 6.10 (d, *J* = 15.4 Hz, 1H), 5.09 (d, *J* = 13.9 Hz, 1H), 5.04 (d, *J* = 13.9 Hz, 1H), 2.48 (s, 3H). **^{13}C NMR (101 MHz, $CDCl_3$)** δ 189.2,

165.8, 142.5, 141.0, 139.4, 136.2, 136.0, 135.8, 133.3, 133.2, 130.0, 129.7, 129.6, 129.2, 129.0, 128.7, 128.1, 118.2, 54.2, 21.2. **HRMS(ESI):** $[M+H]^+$ calcd. $C_{24}H_{21}O_2NCl$ m/z 390.1255, found 390.1266.

***(E)*-*N*-benzyl-3-(2-chlorophenyl)-*N*-(2-formyl-4-methylphenyl)acrylamide (4s)**

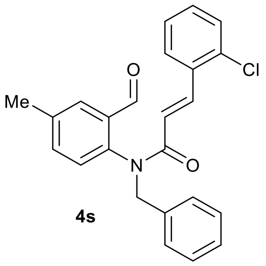

4s

The title product compound **4s** was prepared using general procedure A from 2-amino-5-methylbenzoic acid (1.814 g, 12 mmol) and isolated by column chromatography (4:1 *n*-pentane:EA) giving a solid (374.3 mg, 0.96 mmol, 8% yield).

^{1}H NMR (400 MHz, CDCl$_3$) δ 9.64 (s, 1H), 8.14 (d, *J* = 15.5 Hz, 1H), 7.74 (d, *J* = 1.6 Hz, 1H), 7.46 (dd, *J* = 8.0, 1.7 Hz, 1H), 7.33 (dd, *J* = 8.1, 1.3 Hz, 1H), 7.29 – 7.17 (m, 6H), 7.15 – 7.09 (m, 2H), 7.06 (d, *J* = 8.0 Hz, 1H), 6.13 (d, *J* = 15.5 Hz, 1H), 5.10 (d, *J* = 13.9 Hz, 1H), 5.01 (d, *J* = 13.9 Hz, 1H), 2.45 (s, 3H). **^{13}C NMR (101 MHz, CDCl$_3$)** δ 189.2, 165.6, 141.0, 139.8, 139.4, 136.2, 136.0, 134.9, 133.2, 133.2, 130.6, 130.1, 130.0, 129.6, 129.6, 128.7, 128.1, 127.7, 126.8, 120.5, 54.2, 21.1. **HRMS(ESI):** $[M+H]^+$ calcd. $C_{24}H_{21}O_2NCl$ m/z 390.1255, found 390.1269.

***N*-(4-bromo-2-formylphenyl)-*N*-(2-methylbenzyl)cinnamamide (4t)**

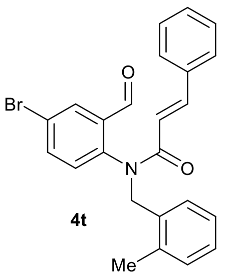

4t

The title product compound **4t** was prepared using general procedure A from 2-amino-5-bromobenzoic acid (1.296 g, 6.0 mmol) and isolated by column chromatography (4:1 *n*-pentane:EA) giving a solid (755.7 mg, 1.7 mmol, 29% yield).

^{1}H NMR (500 MHz, CDCl$_3$) δ 9.56 (s, 1H), 8.02 (d, *J* = 2.4 Hz, 1H), 7.80 (d, *J* = 15.4 Hz, 1H), 7.73 (dd, *J* = 8.3, 2.5 Hz, 1H), 7.34 – 7.25 (m, 5H), 7.19 – 7.14 (m, 1H), 7.13 – 7.00 (m, 4H), 6.06 (d, *J* = 15.4 Hz, 1H), 5.22 (d, *J* = 14.2 Hz, 1H), 5.01 (d, *J* = 14.2 Hz, 1H), 2.15 (s, 3H). **^{13}C NMR (126 MHz, CDCl$_3$)** δ 187.3, 165.7, 144.9, 142.5, 138.2, 137.2, 135.4, 134.6, 133.6, 132.1, 132.1, 131.0, 130.9, 130.3, 128.9, 128.6, 128.2, 126.5, 123.4, 117.2, 50.9, 19.3. **HRMS(ESI):** $[M+H]^+$ calcd. $C_{24}H_{21}O_2NBr$ m/z 434.0750, found 434.0754.

N-(2-formyl-4-methylphenyl)-N-(2-methylbenzyl)cinnamamide (4u)

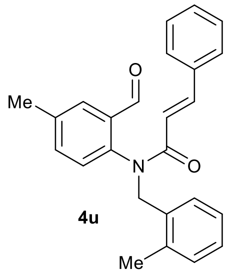

4u

The title product compound **4u** was prepared using general procedure A from 2-amino-5-methylbenzoic acid (1.814 g, 12 mmol) and isolated by column chromatography (4:1 *n*-pentane:EA) giving a solid (399.0 mg, 1.1 mmol, 9% yield).

^{1}H NMR (400 MHz, CDCl$_3$) δ 9.65 (s, 1H), 7.78 (d, *J* = 15.4 Hz, 1H), 7.71 (dd, *J* = 1.9, 1.0 Hz, 1H), 7.41 (dd, *J* = 8.1, 2.2 Hz, 1H), 7.32 – 7.23 (m, 5H), 7.17 – 6.98 (m, 5H), 6.12 (d, *J* = 15.4 Hz, 1H), 5.18 (d, *J* = 14.2 Hz, 1H), 5.06 (d, *J* = 14.1 Hz, 1H), 2.45 (s, 3H), 2.12 (s, 3H). **^{13}C NMR (101 MHz, CDCl$_3$)** δ 189.1, 166.0, 144.0, 141.1, 139.4, 137.2, 136.2, 134.9, 134.1, 133.6, 130.9, 130.6, 130.2, 130.0, 129.5, 128.8, 128.3, 128.1, 126.3, 117.8, 50.9, 21.2, 19.3. **HRMS(ESI):** $[M+H]^+$ calcd. $C_{25}H_{24}O_2N$ m/z 370.1802, found 370.1803.

N-(3-bromobenzyl)-N-(4-fluoro-2-formylphenyl)cinnamamide (4v)

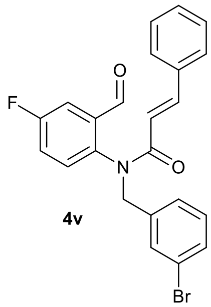

4v

The title product compound **4v** was prepared using general procedure A from 2-amino-5-fluorobenzoic acid (930.8 mg, 6.0 mmol) and isolated by column chromatography (4:1 *n*-pentane:EA) giving a solid (2.051 g, 4.7 mmol, 78% yield).

^{1}H NMR (400 MHz, CDCl$_3$) δ 9.68 (d, *J* = 2.8 Hz, 1H), 7.79 (d, *J* = 15.4 Hz, 1H), 7.64 (dd, *J* = 8.1, 3.1 Hz, 1H), 7.43 – 7.23 (m, 8H), 7.16 – 7.10 (m, 3H), 6.05 (d, *J* = 15.4 Hz, 1H), 5.09 (d, *J* = 14.1 Hz, 1H), 4.88 (d, *J* = 14.1 Hz, 1H). **^{13}C NMR (101 MHz, CDCl$_3$)** δ 187.6, 166.2, 162.3 (d, *J* = 252.6 Hz), 145.0, 139.5 (d, *J* = 3.4 Hz), 138.2, 135.3 (d, *J* = 6.8 Hz), 134.6, 132.5, 132.4 (d, *J* = 7.8 Hz), 131.5, 130.4, 130.4, 128.9, 128.2, 122.9, 122.7 (d, *J* = 23.1 Hz), 116.9, 116.0 (d, *J* = 23.4 Hz), 53.7. **^{19}F NMR (470 MHz, CDCl$_3$)** δ -109.3 (tdd, *J* = 7.6, 4.5, 3.0 Hz). **HRMS(ESI):** $[M+H]^+$ calcd. $C_{23}H_{18}O_2NBrF$ m/z 438.0500, found 438.0503.

N-(3-bromobenzyl)-N-(2-formyl-4-methylphenyl)cinnamamide (4w)

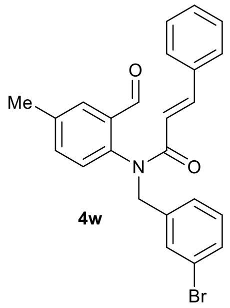

4w

The title product compound **4w** was prepared using general procedure A from 2-amino-5-methylbenzoic acid (1.814 g, 12 mmol) and isolated by column chromatography (4:1 *n*-pentane:EA) giving a solid (469.1 mg, 1.1 mmol, 9% yield).

^{1}H NMR (400 MHz, CDCl$_3$) δ 9.75 (s, 1H), 7.78 (d, *J* = 15.4 Hz, 1H), 7.77 (d, *J* = 2.2 Hz, 1H), 7.45 (dd, *J* = 8.4, 1.6 Hz, 1H), 7.42 – 7.37 (m, 2H), 7.29 – 7.23 (m, 5H), 7.18 – 7.11 (m, 2H), 7.01 (d, *J* = 8.0 Hz, 1H), 6.11 (d, *J* = 15.5 Hz, 1H), 5.14 (d, *J* = 14.1 Hz, 1H), 4.83 (d, *J* = 14.1 Hz, 1H), 2.47 (s, 3H). **^{13}C NMR (101 MHz, CDCl$_3$)** δ 189.2, 166.2, 144.2, 140.8, 139.6, 138.6,

136.3, 134.7, 132.9, 132.4, 131.2, 130.2, 130.0, 128.8, 128.1, 128.1, 122.6, 117.4, 53.5, 21.2. **HRMS(ESI):** $[M+H]^+$ calcd. $C_{24}H_{21}O_2NBr$ m/z 434.0750, found 434.0757.

***N*-(4-chlorobenzyl)-*N*-(2-formyl-4-methoxyphenyl)cinnamamide (4x)**

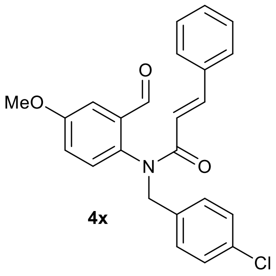

4x

The title product compound **4x** was prepared using general procedure A from 2-amino-5-methoxybenzoic acid (1.003 g, 6.0 mmol) and isolated by column chromatography (2:1 *n*-pentane:EA) giving a solid (1.534 g, 3.8 mmol, 63% yield).

^{1}H NMR (400 MHz, $CDCl_3$) δ 9.67 (s, 1H), 7.76 (d, *J* = 15.5 Hz, 1H), 7.41 (d, *J* = 3.1 Hz, 1H), 7.32 – 7.22 (m, 7H), 7.19 – 7.13 (m, 3H), 7.01 (d, *J* = 8.7 Hz, 1H), 6.10 (d, *J* = 15.4 Hz, 1H), 5.06 (d, *J* = 14.0 Hz, 1H), 4.90 (d, *J* = 14.0 Hz, 1H), 3.90 (s, 3H). **^{13}C NMR (101 MHz, $CDCl_3$)** δ 188.9, 166.5, 159.7, 144.3, 136.5, 134.8, 134.7, 134.3, 134.1, 131.5, 131.1, 130.1, 128.9, 128.8, 128.1, 122.5, 117.5, 112.0, 55.9, 53.7. **HRMS(ESI):** $[M+H]^+$ calcd. $C_{24}H_{21}O_3NCl$ m/z 406.1205, found 406.1207.

***N*-(4-fluoro-2-formylphenyl)-*N*-methylcinnamamide (4y)**

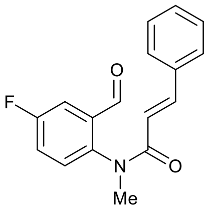

4y

The title product compound **4y** was prepared using general procedure A from 2-amino-5-fluorobenzoic acid (930.8 mg, 6.0 mmol) and isolated by column chromatography (4:1 *n*-pentane:EA) giving a solid (1.257 g, 4.4 mmol, 74% yield).

^{1}H NMR (400 MHz, $CDCl_3$) δ 10.04 (d, *J* = 2.8 Hz, 1H), 7.71 (d, *J* = 15.5 Hz, 1H), 7.67 (dd, *J* = 8.1, 2.7 Hz, 1H), 7.45 – 7.36 (m, 1H), 7.37 – 7.31 (m, 1H), 7.30 – 7.23 (m, 5H), 6.10 (d, *J* = 15.4 Hz, 1H), 3.41 (s, 3H). **^{13}C NMR (101 MHz, $CDCl_3$)** δ 188.0 (d, *J* = 1.6 Hz), 166.4, 162.1 (d, *J* = 252.0 Hz), 143.9, 141.8 (d, *J* = 3.2 Hz), 134.6, 131.5 (d, *J* = 7.9 Hz), 130.1, 128.8, 128.0, 122.8 (d, *J* = 23.1 Hz), 117.2, 115.8 (d, *J* = 23.4 Hz), 38.7. **^{19}F NMR (470 MHz, $CDCl_3$)** δ -109.9 - -110.0 (m). **HRMS(ESI):** $[M+H]^+$ calcd. $C_{17}H_{15}O_2NF$ m/z 284.1081, found 284.1078.

***N*-benzyl-*N*-(4,5-difluoro-2-formylphenyl)cinnamamide (4z)**

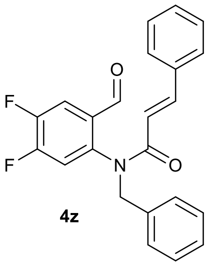

4z

The title product compound **4z** was prepared using general procedure A from 2-amino-4,5-difluorobenzoic acid (519.4 mg, 3.0 mmol) and isolated by column chromatography (4:1 *n*-pentane:EA) giving a solid (452.9 mg, 1.2 mmol, 40% yield).

^{1}H NMR (400 MHz, $CDCl_3$) δ 9.51 (d, *J* = 2.9 Hz, 1H), 7.86 (d, *J* = 15.4 Hz, 1H), 7.77 (dd, *J* = 9.9, 8.6 Hz, 1H), 7.38 – 7.30 (m, 8H), 7.28 – 7.21 (m, 2H), 7.09 (dd, *J* = 10.0, 6.7 Hz, 1H), 6.12 (d, *J* = 15.4 Hz, 1H), 5.24 (d, *J* = 14.0 Hz, 1H), 4.94 (d, *J* = 14.0 Hz, 1H). **^{13}C NMR (101

MHz, CDCl₃) δ 186.3, 165.7, 154.0 (dd, *J* = 262.5, 13.9 Hz), 150.4 (dd, *J* = 255.2, 12.9 Hz), 145.2, 140.7 (dd, *J* = 8.4, 3.4 Hz), 135.3, 134.4, 131.4 (t, *J* = 3.7 Hz), 130.3, 129.5, 128.9, 128.8, 128.5, 128.1, 119.3 (d, *J* = 17.8 Hz), 117.3 (dd, *J* = 18.6, 2.5 Hz), 116.7, 54.1, 53.5. **^{19}F NMR (377 MHz, $CDCl_3$)** δ -123.5 (dt, *J* = 21.7, 9.3 Hz), -133.4 (dddd, *J* = 20.1, 9.5, 6.4, 2.7 Hz). **HRMS(ESI):** $[M+H]^+$ calcd. $C_{23}H_{18}O_2NF_2$ m/z 378.1300, found 378.1302.

***N*-benzyl-*N*-(3-formylnaphthalen-2-yl)cinnamamide (4aa)**

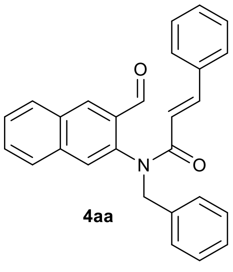

4aa

The title product compound **4aa** was prepared using general procedure A from 3-amino-2-naphthoic acid (748.8 mg, 4.0 mmol) and isolated by column chromatography (4:1 *n*-pentane:EA) giving a solid (469.8 mg, 1.2 mmol, 30% yield).

^{1}H NMR (400 MHz, $CDCl_3$) δ 9.86 (s, 1H), 8.50 (s, 1H), 8.06 (dd, *J* = 7.2, 2.0 Hz, 1H), 7.84 – 7.78 (m, 2H), 7.70 – 7.62 (m, 2H), 7.57 (s, 1H), 7.31 – 7.16 (m, 10H), 6.15 (d, *J* = 15.5 Hz, 1H), 5.28 (d, *J* = 14.0 Hz, 1H), 4.97 (d, *J* = 14.0 Hz, 1H). **^{13}C NMR (101 MHz, $CDCl_3$)** δ 189.3, 166.2, 144.0, 138.6, 136.5, 136.1, 134.9, 133.0, 132.1, 131.1, 130.0, 130.0, 129.9, 129.7, 129.6, 128.8, 128.7, 128.1, 128.1, 128.1, 117.9, 54.3. **HRMS(ESI):** $[M+H]^+$ calcd. $C_{27}H_{22}O_2N$ m/z 392.1645, found 392.1638.

(*E*)-*N*-benzyl-3-(4-cyanophenyl)-*N*-(2-formylphenyl)acrylamide (4bb)

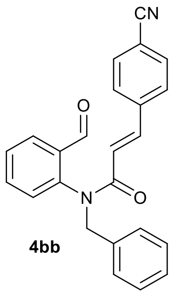

4bb

The compound **4bb** was synthesised according to the literature published procedures.[13]

^{1}H NMR (700 MHz, $CDCl_3$) δ 9.65 (s, 1H), 7.95 (dd, *J* = 7.7, 1.7 Hz, 1H), 7.74 (d, *J* = 15.5 Hz, 1H), 7.67 (td, *J* = 7.6, 1.7 Hz, 1H), 7.56 (t, *J* = 7.6 Hz, 1H), 7.54 – 7.52 (m, 2H), 7.32 – 7.29 (m, 2H), 7.27-7.23 (m, 3H), 7.20-7.17 (m, 2H), 7.16 (d, *J* = 7.9 Hz, 1H), 6.14 (d, *J* = 15.4 Hz, 1H), 5.06 (s, 2H). **^{13}C NMR (176 MHz, $CDCl_3$)** δ 188.9, 165.1, 143.2, 141.7, 139.1, 135.7, 135.6, 133.7, 132.6 , 130.2, 129.9, 129.7, 129.4, 128.8 , 128.4 , 128.3, 121.1, 118.5, 113.1, 54.3. **HRMS(ESI):** $[M+H]^+$ calcd. $C_{24}H_{19}N_2O_2$ m/z 367.1441, found 367.1443.

***N*-(4-cyanobenzyl)-*N*-(2-formylphenyl)cinnamamide (4cc)**

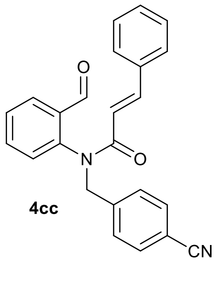

4cc

The title product compound **4cc** was prepared using general procedure B from aldehyde **S3** (125.6 mg, 0.50 mmol) and isolated by column chromatography (4:1 n-pentane:EA) giving a solid (157.4 mg, 0.43 mmol, 86% yield).

^{1}H NMR (400 MHz, $CDCl_3$) δ 9.86 (s, 1H), 8.00 (dd, *J* = 7.7, 1.7 Hz, 1H), 7.78 (d, *J* = 15.4 Hz, 1H), 7.70 – 7.66 (m, 1H), 7.66 – 7.61 (m, 1H), 7.59 (d, *J* = 8.2 Hz, 3H), 7.38

(d, *J* = 8.2 Hz, 2H), 7.32 – 7.27 (m, 3H), 7.25 – 7.23 (m, 1H), 7.11 (dd, *J* = 7.7, 1.2 Hz, 1H), 6.09 (d, *J* = 15.4 Hz, 1H), 5.34 (d, *J* = 14.4 Hz, 1H), 4.83 (d, *J* = 14.4 Hz, 1H). **^{13}C NMR (176 MHz, $CDCl_3$)** δ 188.9, 166.3, 144.7, 143.1, 141.7, 135.6, 134.6, 133.2, 132.6, 130.8, 130.3, 130.3, 130.1, 129.5, 128.9, 128.1, 118.6, 117.0, 112.0, 53.9. **HRMS(ESI):** $[M+H]^+$ calcd. $C_{24}H_{19}O_2N_2$ m/z 367.1441, found 367.1441.

4.2.2 Asymmetric synthesis of pyrrolo[3,2-*c*]quinolines

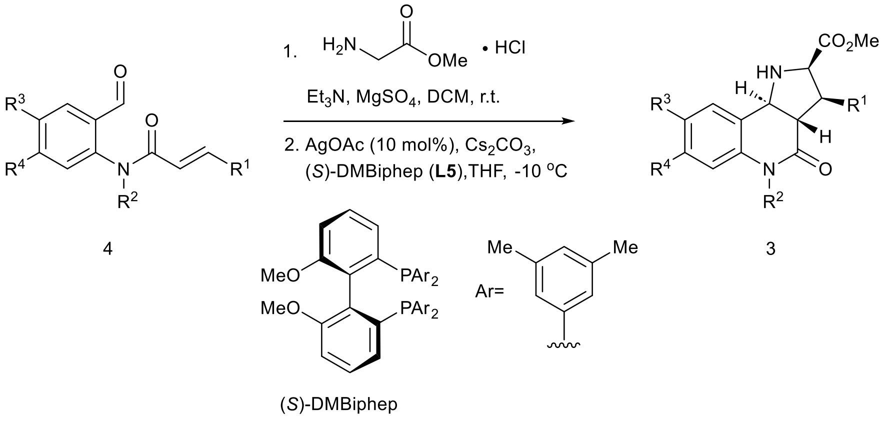

General procedure C: Glycine methyl ester (37.6 mg, 0.30 mmol, 3.0 equiv.) and $MgSO_4$ (36.1 mg, 0.30 mmol, 3.0 equiv.) were stirred in DCM (1.5 mL) followed by the addition of Et_3N (41.7 µL, 0.30 mmol, 3.0 equiv.). The reaction was stirred for 30 min at room temperature before the addition of aldehyde **4** (0.10 mmol, 1 equiv.) in DCM (0.5 mL). The reaction was stirred for 6 h before the addition of saturated NH_4Cl solution (10 mL) to quench the reaction. The mixture was extracted using Et_2O (10 x 3 mL). The organic phase was combined and washed by saturated $NaHCO_3$ solution (10 mL) and brine (10 mL) sequentially, dried by anhydrous Na_2SO_4. The mixture was filtered and concentrated for the next step without any purification.

Then the residue was dissolved in the dry THF (1.0 mL) and cooled in -10 °C bath followed by the addition of a solution of AgOAc (1.7 mg, 0.010 mmol, 0.1 equiv.) and (*S*)-DMBiphep (8.3 mg, 0.012 mmol, 0.12 equiv.) in THF (1.0 mL). Cs_2CO_3 (6.5 mg, 0.020 mmol, 0.2 equiv.) was added to the reaction and stirred until the complete consumption of the iminoester. The solvent was removed under reduced pressure. Compound **4** was purified by silica gel chromatography using *n*-pentane/EA.

Racemic synthesis was conducted in the same condition except the usage of Ph_3P (3.1 mg, 0.012 mmol, 0.12 equiv.) in replacement of the chiral ligand (*S*)-DMBiphep.

Methyl (2*R*,3*R*,3a*S*,9b*S*)-5-benzyl-4-oxo-3-phenyl-2,3,3a,4,5,9b-hexahydro-1*H*-pyrrolo[3,2-*c*]quinoline-2-carboxylate (3a)

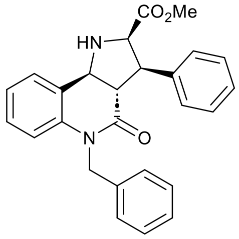

3a

The title product compound **3a** was prepared using general procedure C from **4a** (34.1 mg, 0.10 mmol) and isolated by column chromatography (2:1 *n*-pentane:EA) giving a solid (27.6 mg, 0.067 mmol, 67% yield).

^{1}H NMR, **^{13}C NMR** and **HRMS(ESI)** are identical with the reported ones.[13] **HPLC conditions**: CHIRAPAK IC column, *iso*-propanol / *iso*-hexane = 40/60, flow rate = 0.5 mL min^{-1}, major enantiomer: t_R = 71.5 min; minor enantiomer: t_R = 42.1 min. *ee*=90%. **$[\alpha]_D^{20}$** = -124.7 (c = 0.32, CH_2Cl_2).

Methyl (2*S*,3*S*,3a*R*,9b*R*)-5-benzyl-4-oxo-3-phenyl-2,3,3a,4,5,9b-hexahydro-1*H*-pyrrolo[3,2-*c*]quinoline-2-carboxylate (*ent*-3a)

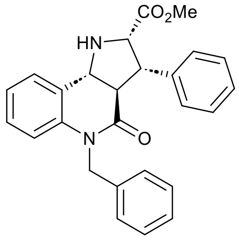

ent-**3a**

The title product compound *ent*-**3a** was prepared using general procedure C from **4a** (34.1 mg, 0.10 mmol) but with (*R*)-DMBiphep and isolated by column chromatography (2:1 *n*-pentane:EA) giving a solid (23.1 mg, 0.056 mmol, 56% yield).

^{1}H NMR, **^{13}C NMR** and **HRMS(ESI)** are identical with the reported ones.[13] **HPLC conditions**: CHIRAPAK IC column, *iso*-propanol / *iso*-hexane = 40/60, flow rate = 0.5 mL min^{-1}, major enantiomer: t_R = 39.6 min; minor enantiomer: t_R = 74.1 min. *ee*=90%. **$[\alpha]_D^{20}$** = +132.3 (c = 0.38, CH_2Cl_2).

Methyl (2*R*,3*R*,3a*S*,9b*S*)-5-benzyl-3-(4-chlorophenyl)-4-oxo-2,3,3a,4,5,9b-hexahydro-1*H*-pyrrolo[3,2-*c*]quinoline-2-carboxylate (3b)

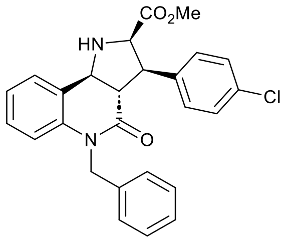

3b

The title product compound **3b** was prepared using general procedure C from **4b** (37.6 mg, 0.10 mmol) and isolated by column chromatography (2:1 *n*-pentane:EA) giving a solid (41.0 mg, 0.092 mmol, 92% yield).

^{1}H NMR, **^{13}C NMR** and **HRMS(ESI)** are identical with the reported ones.[13] **HPLC conditions**: CHIRAPAK IC column, *iso*-propanol / *iso*-hexane = 40/60, flow rate = 0.5 mL min^{-1}, major enantiomer: t_R = 49.6 min; minor enantiomer: t_R = 24.7 min. *ee*=85%. **$[\alpha]_D^{20}$** = -105.6 (c = 0.18, CH_2Cl_2).

Methyl (2*R*,3*R*,3a*S*,9b*S*)-5-benzyl-3-(3-chlorophenyl)-4-oxo-2,3,3a,4,5,9b-hexahydro-1*H*-pyrrolo[3,2-*c*]quinoline-2-carboxylate (3c)

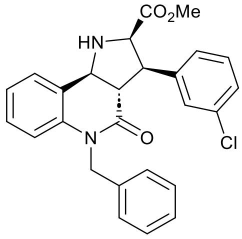

3c

The title product compound **3c** was prepared using general procedure C from **4c** (37.6 mg, 0.10 mmol) and isolated by column chromatography (2:1 *n*-pentane:EA) giving a solid (36.1 mg, 0.081 mmol, 81% yield).

^{1}H NMR, **^{13}C NMR** and **HRMS(ESI)** are identical with the reported ones.[13] **HPLC conditions**: CHIRAPAK IC column, *iso*-propanol / *iso*-hexane = 40/60, flow rate = 0.5 mL min^{-1}, major enantiomer: t_R = 47.3 min; minor enantiomer: t_R = 31.6 min. *ee*=76%. **$[\alpha]_D^{20}$** = -106.0 (c = 0.20, CH_2Cl_2).

Methyl (2*R*,3*R*,3a*S*,9b*S*)-5-benzyl-3-(2-chlorophenyl)-4-oxo-2,3,3a,4,5,9b-hexahydro-1*H*-pyrrolo[3,2-*c*]quinoline-2-carboxylate (3d)

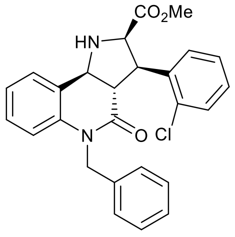

3d

The title product compound **3d** was prepared using general procedure C from **4d** (37.6 mg, 0.10 mmol) and isolated by column chromatography (2:1 *n*-pentane:EA) giving a solid (32.8 mg, 0.073 mmol, 73% yield).

^{1}H NMR, **^{13}C NMR** and **HRMS(ESI)** are identical with the reported ones.[13] **HPLC conditions**: CHIRAPAK IC column, *iso*-propanol / *iso*-hexane = 40/60, flow rate = 0.5 mL min^{-1}, major enantiomer: t_R = 52.8 min; minor enantiomer: t_R = 31.2 min. *ee*=75%. **$[\alpha]_D^{20}$** = -112.2 (c = 0.18, CH_2Cl_2).

Methyl (2*R*,3*R*,3a*S*,9b*S*)-5-(2-methylbenzyl)-4-oxo-3-phenyl-2,3,3a,4,5,9b-hexahydro-1*H*-pyrrolo[3,2-*c*]quinoline-2-carboxylate (3e)

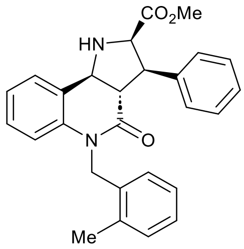

3e

The title product compound **3e** was prepared using general procedure C from **4e** (35.5 mg, 0.10 mmol) and isolated by column chromatography (2:1 *n*-pentane:EA) giving a solid (26.4 mg, 0.062 mmol, 62% yield).

^{1}H NMR (500 MHz, $CDCl_3$) δ 7.48 (dt, *J* = 7.3, 1.5 Hz, 1H), 7.31 – 7.24 (m, 2H), 7.23 – 7.18 (m, 4H), 7.17 – 7.10 (m, 3H), 7.05 (td, *J* = 7.5, 1.6 Hz, 1H), 6.84 (ddd, *J* = 8.1, 3.6, 1.2 Hz, 2H), 5.31 (d, *J* = 16.9 Hz, 1H), 4.84 (d, *J* = 16.9 Hz, 1H), 4.51 (d, *J* = 10.4 Hz, 1H), 4.30 (dt, *J* = 13.6, 1.0 Hz, 1H), 4.07 (t, *J* = 10.7 Hz, 1H), 3.16 – 3.07 (m, 4H), 2.36 (s, 3H). **^{13}C NMR (126 MHz, $CDCl_3$)** δ 172.5, 169.5, 139.8, 138.6, 135.0, 134.2, 130.5, 129.5, 128.5, 128.4, 128.1, 127.3, 127.0, 126.4, 124.9, 123.8, 123.3, 116.3, 66.9, 60.3, 54.4, 51.9, 49.7, 44.7, 19.3. **HRMS(ESI):** $[M+H]^+$ calcd. $C_{27}H_{27}O_3N_2$ m/z 427.2016, found 427.2018. **HPLC conditions**: CHIRAPAK IC

column, *iso*-propanol / *iso*-hexane = 40/60, flow rate = 0.5 mL min^{-1}, major enantiomer: t_R = 58.3 min; minor enantiomer: t_R = 41.5 min. *ee*=88%. **[α]$_D$^{20}** = -145.7 (c = 0.14, CH_2Cl_2).

Methyl (2*R*,3*R*,3a*S*,9b*S*)-5-(3-bromobenzyl)-4-oxo-3-phenyl-2,3,3a,4,5,9b-hexahydro-1*H*-pyrrolo[3,2-*c*]quinoline-2-carboxylate (3f)

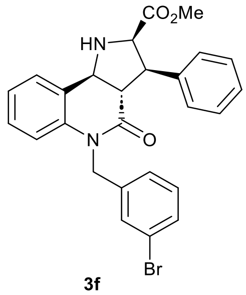

3f

The title product compound **3f** was prepared using general procedure C from **4f** (42.0 mg, 0.10 mmol) and isolated by column chromatography (2:1 *n*-pentane:EA) giving a solid (23.1 mg, 0.047 mmol, 47% yield).

^{1}H NMR (500 MHz, $CDCl_3$) δ 7.46 (dt, *J* = 7.4, 1.5 Hz, 1H), 7.36 – 7.31 (m, 2H), 7.30 – 7.18 (m, 6H), 7.18 – 7.11 (m, 2H), 7.10 – 7.07 (m, 1H), 6.94 (d, *J* = 7.7 Hz, 1H), 5.19 (d, *J* = 16.3 Hz, 1H), 5.00 (d, *J* = 16.4 Hz, 1H), 4.50 (d, *J* = 10.4 Hz, 1H), 4.23 (d, *J* = 13.5 Hz, 1H), 4.07 (t, *J* = 10.7 Hz, 1H), 3.13 (s, 3H), 3.08 (dd, *J* = 13.5, 11.1 Hz, 1H). **^{13}C NMR (126 MHz, $CDCl_3$)** δ 172.5, 169.7, 139.4, 139.2, 138.5, 130.6, 130.4, 129.7, 129.6, 128.5, 128.4, 128.0, 127.3, 125.3, 123.9, 123.5, 123.0, 116.1, 66.8, 60.2, 54.1, 51.9, 49.6, 45.7. **HRMS(ESI):** $[M+H]^+$ calcd. $C_{26}H_{24}O_3N_2Br$ m/z 491.0965, found 491.0960. **HPLC conditions**: CHIRAPAK IC column, *iso*-propanol / *iso*-hexane = 40/60, flow rate = 0.5 mL min^{-1}, major enantiomer: t_R = 58.3 min; minor enantiomer: t_R = 37.6 min. *ee*=85%. **[α]$_D$^{20}** = -76.1 (c = 0.19, CH_2Cl_2).

Methyl (2*R*,3*R*,3a*S*,9b*S*)-5-(4-chlorobenzyl)-4-oxo-3-phenyl-2,3,3a,4,5,9b-hexahydro-1*H*-pyrrolo[3,2-*c*]quinoline-2-carboxylate (3g)

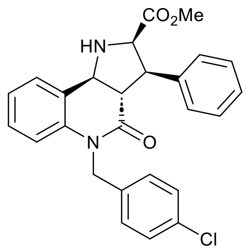

3g

The title product compound **3g** was prepared using general procedure C from **4g** (37.6 mg, 0.10 mmol) and isolated by column chromatography (2:1 *n*-pentane:EA) giving a solid (26.4 mg, 0.059 mmol, 59% yield).

^{1}H NMR (500 MHz, $CDCl_3$) δ 7.47 (d, *J* = 7.4 Hz, 1H), 7.31 – 7.18 (m, 8H), 7.17 – 7.10 (m, 3H), 6.96 (d, *J* = 8.1 Hz, 1H), 5.19 (d, *J* = 16.2 Hz, 1H), 5.00 (d, *J* = 16.2 Hz, 1H), 4.52 (d, *J* = 10.4 Hz, 1H), 4.24 (d, *J* = 13.6 Hz, 1H), 4.07 (t, *J* = 10.7 Hz, 1H), 3.14 (s, 3H), 3.08 (td, *J* = 12.0, 11.1, 1.7 Hz, 1H). **^{13}C NMR (126 MHz, $CDCl_3$)** δ 172.4, 169.5, 139.3, 138.4, 138.4, 135.6, 133.1, 129.0, 128.5, 128.4, 128.2, 128.1, 127.4, 124.0, 123.5, 116.2, 66.8, 60.2, 54.1, 54.1, 51.9, 49.6, 49.5, 45.7. **HRMS(ESI):** $[M+H]^+$ calcd. $C_{26}H_{24}O_3N_2Cl$ m/z 447.1470, found 447.1470. **HPLC conditions**: CHIRAPAK IC column, *iso*-propanol / *iso*-hexane = 40/60, flow rate = 0.5 mL min^{-1}, major enantiomer: t_R = 52.3 min; minor enantiomer: t_R = 34.6 min. *ee*=85%. **[α]$_D$^{20}** = -82.5 (c = 0.16, CH_2Cl_2).

Methyl (2*R*,3*R*,3a*S*,9b*S*)-5-methyl-4-oxo-3-phenyl-2,3,3a,4,5,9b-hexahydro-1*H*-pyrrolo[3,2-*c*]quinoline-2-carboxylate (3h)

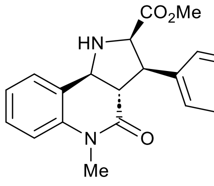

3h

The title product compound **3h** was prepared using general procedure C from **4h** (26.5 mg, 0.10 mmol) and isolated by column chromatography (2:1 *n*-pentane:EA) giving a solid (18.8 mg, 0.056 mmol, 56% yield).

^{1}H NMR (700 MHz, CDCl$_3$) δ 7.48 (d, *J* = 7.4 Hz, 1H), 7.38 (t, *J* = 7.9 Hz, 1H), 7.30 – 7.26 (m, 2H), 7.23 – 7.18 (m, 4H), 7.09 (d, *J* = 8.1 Hz, 1H), 4.49 (d, *J* = 10.4 Hz, 1H), 4.18 (d, *J* = 13.6 Hz, 1H), 4.01 (t, *J* = 10.8 Hz, 1H), 3.36 (s, 3H), 3.14 (s, 3H), 2.94 (dd, *J* = 13.5, 11.2 Hz, 1H). **^{13}C NMR (176 MHz, CDCl$_3$)** δ 172.5, 169.4, 140.4, 138.6, 129.3, 128.4, 128.4, 128.1, 127.2, 123.6, 123.2, 115.5, 66.8, 60.0, 54.2, 51.8, 49.7, 29.8. **HRMS(ESI):** $[M+H]^+$ calcd. $C_{20}H_{21}O_3N_2$ m/z 337.1547, found 337.1546. **HPLC conditions**: CHIRAPAK IC column, *iso*-propanol / *iso*-hexane = 40/60, flow rate = 0.5 mL min^{-1}, major enantiomer: t_R = 58.2 min; minor enantiomer: t_R = 41.4 min. *ee*=79%. **$[\alpha]_D^{20}$** = -143.4 (c = 0.11, CH_2Cl_2).

Methyl (2*R*,3*R*,3a*S*,9b*S*)-5-benzyl-8-fluoro-4-oxo-3-phenyl-2,3,3a,4,5,9b-hexahydro-1*H*-pyrrolo[3,2-*c*]quinoline-2-carboxylate (3i)

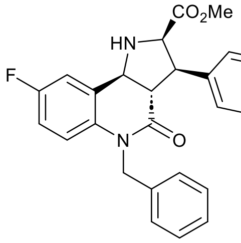

3i

The title product compound **3i** was prepared using general procedure C from **4i** (35.9 mg, 0.10 mmol) and isolated by column chromatography (2:1 *n*-pentane:EA) giving a solid (32.1 mg, 0.075 mmol, 75% yield).

^{1}H NMR (500 MHz, CDCl$_3$) δ 7.33 – 7.27 (m, 4H), 7.24 – 7.12 (m, 7H), 6.96 – 6.84 (m, 2H), 5.19 (d, *J* = 16.1 Hz, 1H), 5.04 (d, *J* = 16.2 Hz, 1H), 4.50 (d, *J* = 10.4 Hz, 1H), 4.24 (d, *J* = 13.5 Hz, 1H), 4.07 (t, *J* = 10.7 Hz, 1H), 3.14 (s, 3H), 3.11 (dd, *J* = 13.6, 11.0 Hz, 1H). **^{13}C NMR (126 MHz, CDCl$_3$)** δ 172.3, 169.2, 159.1 (d, *J* = 245.0 Hz), 138.2, 136.8, 135.6 (d, *J* = 2.8 Hz), 131.6 (d, *J* = 7.6 Hz), 128.9, 128.5, 128.1, 127.5, 127.4, 126.7, 117.8 (d, *J* = 8.1 Hz), 114.52 (d, *J* = 22.5 Hz), 111.2 (d, *J* = 24.4 Hz), 66.7, 59.9, 53.5, 51.9, 49.5, 46.4. **^{19}F NMR (470 MHz, CDCl$_3$)** δ -118.38 (td, *J* = 8.2, 4.8 Hz). **HRMS(ESI):** $[M+H]^+$ calcd. $C_{26}H_{24}O_3N_2F$ m/z 431.1766, found 431.1767. **HPLC conditions**: CHIRAPAK IC column, *iso*-propanol / *iso*-hexane = 40/60, flow rate = 0.5 mL min^{-1}, major enantiomer: t_R = 54.0 min; minor enantiomer: t_R = 32.9 min. *ee*=94%. **$[\alpha]_D^{20}$** = -79.5 (c = 0.20, CH_2Cl_2).

Methyl (2*R*,3*R*,3a*S*,9b*S*)-5-benzyl-8-chloro-4-oxo-3-phenyl-2,3,3a,4,5,9b-hexahydro-1*H*-pyrrolo[3,2-*c*]quinoline-2-carboxylate (3j)

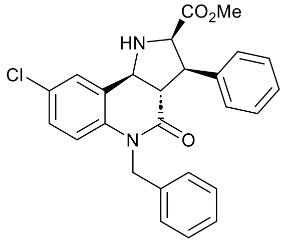

3j

The title product compound **3j** was prepared using general procedure C from **4j** (37.6 mg, 0.10 mmol) and isolated by column chromatography (2:1 *n*-pentane:EA) giving a solid (33.9 mg, 0.076 mmol, 76% yield).

^{1}H NMR (700 MHz, $CDCl_3$) δ 7.44 (dd, *J* = 2.5, 1.2 Hz, 1H), 7.30 – 7.26 (m, 4H), 7.23 – 7.19 (m, 4H), 7.17 (dd, *J* = 8.7, 2.4 Hz, 1H), 7.16 – 7.13 (m, 2H), 6.91 (d, *J* = 8.7 Hz, 1H), 5.15 (d, *J* = 16.3 Hz, 1H), 5.06 (d, *J* = 16.2 Hz, 1H), 4.53 (d, *J* = 10.4 Hz, 1H), 4.26 (d, *J* = 13.7 Hz, 1H), 4.08 (t, *J* = 10.7 Hz, 1H), 3.15 (s, 3H), 3.12 (dd, *J* = 13.6, 11.1 Hz, 1H). **^{13}C NMR (176 MHz, $CDCl_3$)** δ 172.2, 169.1, 138.0, 137.9, 136.5, 131.0, 129.2, 129.0, 128.6, 128.3, 128.1, 127.5, 127.5, 126.7, 123.8, 117.7, 66.5, 59.7, 53.3, 52.0, 49.3, 46.2. **HRMS(ESI):** $[M+H]^+$ calcd. $C_{26}H_{24}O_3N_2Cl$ m/z 447.1470, found 447.1471. **HPLC conditions**: CHIRAPAK IC column, *iso*-propanol / *iso*-hexane = 40/60, flow rate = 0.5 mL min^{-1}, major enantiomer: t_R = 51.7 min; minor enantiomer: t_R = 33.5 min. *ee*=97%. **$[\alpha]_D^{20}$** = -48.1 (c = 0.19, CH_2Cl_2).

Methyl (2*R*,3*R*,3a*S*,9b*S*)-5-benzyl-8-bromo-4-oxo-3-phenyl-2,3,3a,4,5,9b-hexahydro-1*H*-pyrrolo[3,2-*c*]quinoline-2-carboxylate (3k)

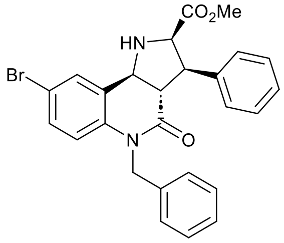

3k

The title product compound **3k** was prepared using general procedure C from **4k** (42.0 mg, 0.1 mmol) and isolated by column chromatography (2:1 *n*-pentane:EA) giving a solid (41.2 mg, 0.084 mmol, 84% yield).

^{1}H NMR (500 MHz, $CDCl_3$) δ 7.57 (dd, *J* = 2.4, 1.3 Hz, 1H), 7.33 – 7.25 (m, 6H), 7.23 – 7.19 (m, 4H), 7.17 – 7.12 (m, 2H), 6.85 (d, *J* = 8.6 Hz, 1H), 5.15 (d, *J* = 16.2 Hz, 1H), 5.06 (d, *J* = 16.2 Hz, 1H), 4.50 (d, *J* = 10.3 Hz, 1H), 4.25 (d, *J* = 13.6 Hz, 1H), 4.06 (t, *J* = 10.7 Hz, 1H), 3.14 (s, 3H), 3.10 (dd, *J* = 13.6, 11.1 Hz, 1H). **^{13}C NMR (126 MHz, $CDCl_3$)** δ 172.3, 169.3, 138.5, 138.2, 136.6, 131.6, 131.1, 129.0, 128.5, 128.1, 127.5, 127.4, 126.7, 126.6, 118.0, 116.7, 66.6, 59.7, 53.4, 51.9, 49.4, 46.2. **HRMS(ESI):** $[M+H]^+$ calcd. $C_{26}H_{24}O_3N_2Br$ m/z 491.0965, found 491.0968. **HPLC conditions**: CHIRAPAK IC column, *iso*-propanol / *iso*-hexane = 40/60, flow rate = 0.5 mL min^{-1}, major enantiomer: t_R = 53.4 min; minor enantiomer: t_R = 34.4 min. *ee*=96%. **$[\alpha]_D^{20}$** = -48.9 (c = 0.13, CH_2Cl_2).

Methyl (2*R*,3*R*,3a*S*,9b*S*)-5-benzyl-8-methyl-4-oxo-3-phenyl-2,3,3a,4,5,9b-hexahydro-1*H*-pyrrolo[3,2-*c*]quinoline-2-carboxylate (3l)

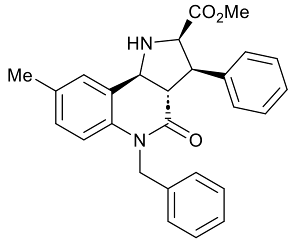

3l

The title product compound **3l** was prepared using general procedure C from **4l** (35.5 mg, 0.10 mmol) and isolated by column chromatography (2:1 *n*-pentane:EA) giving a solid (41.8 mg, 0.098 mmol, 98% yield).

^{1}H NMR (500 MHz, $CDCl_3$) δ 7.30 – 7.24 (m, 5H), 7.23 – 7.15 (m, 6H), 7.01 (d, *J* = 8.3 Hz, 1H), 6.89 (d, *J* = 8.3 Hz, 1H), 5.18 (d, *J* = 16.1 Hz, 1H), 5.04 (d, *J* = 16.2 Hz, 1H), 4.51 (d, *J* = 10.4 Hz, 1H), 4.22 (d, *J* = 13.2 Hz, 1H), 4.08 (t, *J* = 10.8 Hz, 1H), 3.14 (s, 3H), 3.06 (dd, *J* = 13.5, 11.1 Hz, 1H), 2.34 (s, 3H). **^{13}C NMR (126 MHz, $CDCl_3$)** δ 172.4, 169.4, 138.6, 137.1, 137.0, 133.5, 129.3, 128.8, 128.7, 128.5, 128.1, 127.3, 127.3, 126.8, 124.0, 116.3, 66.8, 60.2, 54.3, 51.9, 49.6, 46.2, 20.9. **HRMS(ESI):** $[M+H]^+$ calcd. $C_{27}H_{27}O_3N_2$ m/z 427.2016, found 427.2017. **HPLC conditions**: CHIRAPAK IC column, *iso*-propanol / *iso*-hexane = 40/60, flow rate = 0.5 mL min^{-1}, major enantiomer: t_R = 73.8 min; minor enantiomer: t_R = 41.0 min. *ee*=97%. **$[\alpha]_D^{20}$** = -105.6 (c = 0.17, CH_2Cl_2).

Methyl (2*R*,3*R*,3a*S*,9b*S*)-5-benzyl-8-methoxy-4-oxo-3-phenyl-2,3,3a,4,5,9b-hexahydro-1*H*-pyrrolo[3,2-*c*]quinoline-2-carboxylate (3m)

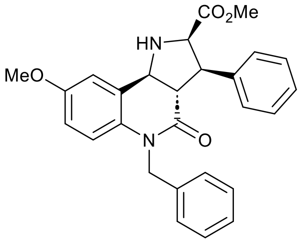

3m

The title product compound **3m** was prepared using general procedure C from **4m** (37.1 mg, 0.10 mmol) and isolated by column chromatography (1:1 *n*-pentane:EA) giving a solid (27.8 g, 0.063 mmol, 63% yield).

^{1}H NMR (400 MHz, $CDCl_3$) δ 7.31 – 7.14 (m, 10H), 7.05 (d, *J* = 2.9 Hz, 1H), 6.92 (d, *J* = 8.9 Hz, 1H), 6.73 (dd, *J* = 8.9, 2.9 Hz, 1H), 5.17 (d, *J* = 16.1 Hz, 1H), 5.02 (d, *J* = 16.1 Hz, 1H), 4.55 (d, *J* = 10.4 Hz, 1H), 4.27 (d, *J* = 13.5 Hz, 1H), 4.10 (t, *J* = 10.8 Hz, 1H), 3.81 (s, 3H), 3.14 (s, 3H), 3.10 (dd, *J* = 13.6, 11.1 Hz, 1H). **^{13}C NMR (101 MHz, $CDCl_3$)** δ 172.1, 168.8, 156.1, 138.2, 137.1, 132.7, 130.3, 128.9, 128.6, 128.1, 127.5, 127.4, 126.8, 117.7, 113.3, 109.4, 66.6, 60.3, 55.8, 53.9, 52.1, 49.4, 46.4. **HRMS(ESI):** $[M+H]^+$ calcd. $C_{27}H_{27}O_4N_2$ m/z 443.1965, found 443.1966. **HPLC conditions**: CHIRAPAK IA column, *iso*-propanol / *iso*-hexane = 7/93, flow rate = 0.5 mL min^{-1}, major enantiomer: t_R = 150.0 min; minor enantiomer: t_R = 131.5 min. *ee*=98%. **$[\alpha]_D^{20}$** = -82.1 (c = 0.11, CH_2Cl_2).

Methyl (2*R*,3*R*,3a*S*,9b*S*)-5-benzyl-4-oxo-3-phenyl-8-(trifluoromethoxy)-2,3,3a,4,5,9b-hexahydro-1*H*-pyrrolo[3,2-*c*]quinoline-2-carboxylate (3n)

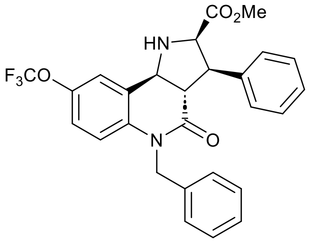

3n

The title product compound **3n** was prepared using general procedure C from **4n** (42.5 mg, 0.10 mmol) and isolated by column chromatography (1:1 *n*-pentane:EA) giving a solid (34.4 mg, 0.069 mmol, 69% yield).

^{1}H NMR (500 MHz, CDCl$_3$) δ 7.34 – 7.26 (m, 5H), 7.25 – 7.19 (m, 4H), 7.18 – 7.14 (m, 2H), 7.07 – 7.04 (m, 1H), 6.98 (d, *J* = 8.9 Hz, 1H), 5.25 (d, *J* = 16.2 Hz, 1H), 5.00 (d, *J* = 16.3 Hz, 1H), 4.50 (d, *J* = 10.3 Hz, 1H), 4.24 (d, *J* = 13.6 Hz, 1H), 4.07 (t, *J* = 10.7 Hz, 1H), 3.14 (s, 3H), 3.13 (dd, *J* = 13.6, 11.0 Hz, 1H). **^{13}C NMR (126 MHz, CDCl$_3$)** δ 172.3, 169.3, 145.0 (q, *J* = 2.0 Hz), 138.2, 138.1, 136.6, 131.4, 129.0, 128.5, 128.1, 127.5, 127.4, 126.7, 120.7, 120.6 (q, *J* = 257.4 Hz), 117.4, 116.8, 66.7, 59.8, 53.4, 51.9, 49.5, 46.5. **^{19}F NMR (470 MHz, CDCl$_3$)** δ -58.0 (s). **HRMS(ESI):** [M+H]$^+$ calcd. $C_{27}H_{24}O_4N_2F_3$ m/z 497.1683, found 497.1686. **HPLC conditions**: CHIRAPAK IC column, *iso*-propanol / *iso*-hexane = 40/60, flow rate = 0.5 mL min^{-1}, major enantiomer: t_R = 26.5 min; minor enantiomer: t_R = 16.6 min. *ee*=96%. **[α]$_D^{20}$** = -51.2 (c = 0.23, CH_2Cl_2).

Methyl (2*R*,3*R*,3a*S*,9b*S*)-5-benzyl-7-fluoro-4-oxo-3-phenyl-2,3,3a,4,5,9b-hexahydro-1*H*-pyrrolo[3,2-*c*]quinoline-2-carboxylate (3o)

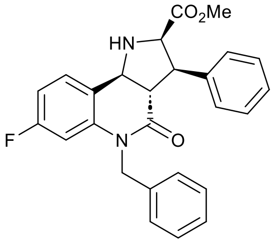

3o

The title product compound **3o** was prepared using general procedure C from **4o** (35.9 mg, 0.10 mmol) and isolated by column chromatography (2:1 *n*-pentane:EA) giving a solid (29.1 mg, 0.068 mmol, 68% yield).

^{1}H NMR (500 MHz, CDCl$_3$) δ 7.41 – 7.37 (m, 1H), 7.31 – 7.26 (m, 4H), 7.24 – 7.19 (m, 4H), 7.19 – 7.15 (m, 2H), 6.81 (td, *J* = 8.3, 2.4 Hz, 1H), 6.74 (dd, *J* = 10.7, 2.4 Hz, 1H), 5.16 (d, *J* = 16.2 Hz, 1H), 5.03 (d, *J* = 16.2 Hz, 1H), 4.50 (d, *J* = 10.3 Hz, 1H), 4.19 (d, *J* = 13.5 Hz, 1H), 4.07 (t, *J* = 10.7 Hz, 1H), 3.13 (s, 3H), 3.07 (dd, *J* = 13.5, 11.0 Hz, 1H). **^{13}C NMR (126 MHz, CDCl$_3$)** δ 172.4, 169.5, 162.7 (d, *J* = 244.8 Hz), 141.0 (d, *J* = 10.3 Hz), 138.4, 136.5, 129.0, 128.5, 128.1, 127.6, 127.4, 126.7, 125.2 (d, *J* = 3.1 Hz), 124.6 (d, *J* = 9.4 Hz), 109.9 (d, *J* = 21.5 Hz), 104.7 (d, *J* = 27.0 Hz), 66.8, 59.8, 54.1, 51.9, 49.6, 46.3. **^{19}F NMR (470 MHz, CDCl$_3$)** δ -112.1 – -112.2 (m). **HRMS(ESI):** [M+H]$^+$ calcd. $C_{26}H_{24}O_3N_2F$ m/z 431.1766, found 431.1767. **HPLC conditions**: CHIRAPAK IC column, *iso*-propanol / *iso*-hexane = 40/60, flow rate = 0.5 mL min^{-1}, major enantiomer: t_R = 42.3 min; minor enantiomer: t_R = 26.7 min. *ee*=83%. **[α]$_D^{20}$** = -126.4 (c = 0.19, CH_2Cl_2).

Methyl (2*R*,3*R*,3a*S*,9b*S*)-5-benzyl-7-bromo-4-oxo-3-phenyl-2,3,3a,4,5,9b-hexahydro-1*H*-pyrrolo[3,2-*c*]quinoline-2-carboxylate (3p)

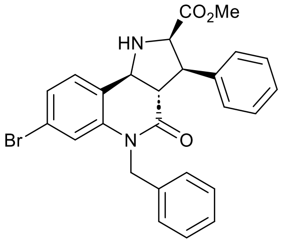

3p

The title product compound **3p** was prepared using general procedure C from **4p** (42.0 mg, 0.10 mmol) and isolated by column chromatography (2:1 *n*-pentane:EA) giving a solid (40.3 mg, 0.082 mmol, 82% yield).

^{1}H NMR (700 MHz, CDCl$_3$) δ 7.31 – 7.26 (m, 5H), 7.26 – 7.15 (m, 8H), 5.13 (d, *J* = 16.2 Hz, 1H), 5.05 (d, *J* = 16.2 Hz, 1H), 4.49 (d, *J* = 10.4 Hz, 1H), 4.17 (d, *J* = 13.5 Hz, 1H), 4.06 (t, *J* = 10.7 Hz, 1H), 3.13 (s, 3H), 3.06 (dd, *J* = 13.6, 11.0 Hz, 1H). **^{13}C NMR (176 MHz, CDCl$_3$)** δ 172.3, 169.4, 140.8, 138.3, 136.4, 129.0, 128.5, 128.5, 128.1, 127.6, 127.4, 126.8, 126.5, 124.8, 121.8, 119.6, 66.7, 59.8, 53.7, 51.9, 49.5, 46.2. **HRMS(ESI):** [M+H]$^+$ calcd. $C_{26}H_{24}O_3N_2{}^{81}Br$ m/z 493.0945, found 493.0947. **HPLC conditions**: CHIRAPAK IC column, *iso*-propanol / *iso*-hexane = 40/60, flow rate = 0.5 mL min^{-1}, major enantiomer: t_R = 46.1 min; minor enantiomer: t_R = 30.0 min. *ee*=80%. **$[\alpha]_D^{20}$** = -95.3 (c = 0.11, CH_2Cl_2).

Methyl (2*R*,3*R*,3a*S*,9b*S*)-5-benzyl-3-(4-chlorophenyl)-8-methyl-4-oxo-2,3,3a,4,5,9b-hexahydro-1*H*-pyrrolo[3,2-*c*]quinoline-2-carboxylate (3q)

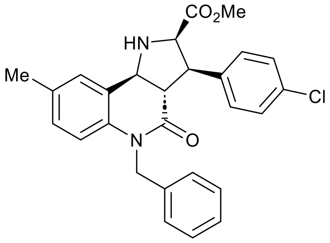

3q

The title product compound **3q** was prepared using general procedure C from **4q** (39.0 mg, 0.10 mmol) and isolated by column chromatography (2:1 *n*-pentane:EA) giving a solid (27.4 mg, 0.059 mmol, 59% yield).

^{1}H NMR (700 MHz, CDCl$_3$) δ 7.29 – 7.25 (m, 3H), 7.23 – 7.18 (m, 4H), 7.17 – 7.14 (m, 2H), 7.09 (d, *J* = 6.9 Hz, 1H), 7.01 (d, *J* = 8.3 Hz, 1H), 6.89 (d, *J* = 8.3 Hz, 1H), 5.17 (d, *J* = 16.2 Hz, 1H), 5.03 (d, *J* = 16.2 Hz, 1H), 4.53 (d, *J* = 10.5 Hz, 1H), 4.24 (d, *J* = 13.5 Hz, 1H), 4.03 (t, *J* = 10.8 Hz, 1H), 3.21 (s, 2H), 3.02 (dd, *J* = 13.5, 11.1 Hz, 1H), 2.32 (s, 3H). **^{13}C NMR (176 MHz, CDCl$_3$)** δ 172.0, 169.0, 140.6, 136.9, 136.8, 134.3, 133.7, 129.8, 128.9, 128.9, 128.8, 128.7, 128.3, 127.6, 127.3, 126.7, 126.3, 124.1, 116.4, 66.5, 60.1, 54.1, 52.2, 49.0, 46.2, 20.9. **HRMS(ESI):** [M+H]$^+$ calcd. $C_{27}H_{26}O_3N_2Cl$ m/z 461.1627, found 461.1630. **HPLC conditions**: CHIRAPAK IC column, *iso*-propanol / *iso*-hexane = 40/60, flow rate = 0.5 mL min^{-1}, major enantiomer: t_R = 55.1 min; minor enantiomer: t_R = 34.9 min. *ee*=96%. **$[\alpha]_D^{20}$** = -110.7 (c = 0.58, CH_2Cl_2).

Methyl (2*R*,3*R*,3a*S*,9b*S*)-5-benzyl-3-(3-chlorophenyl)-8-methyl-4-oxo-2,3,3a,4,5,9b-hexahydro-1*H*-pyrrolo[3,2-*c*]quinoline-2-carboxylate (3r)

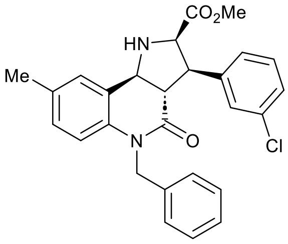

3r

The title product compound **3r** was prepared using general procedure C from **4r** (39.0 mg, 0.10 mmol) and isolated by column chromatography (2:1 *n*-pentane:EA) giving a solid (30.8 mg, 0.067 mmol, 67% yield).

^{1}H NMR (700 MHz, CDCl$_3$) δ 7.29 – 7.25 (m, 5H), 7.23 – 7.20 (m, 1H), 7.19 – 7.14 (m, 4H), 7.02 (d, *J* = 8.3 Hz, 1H), 6.90 (d, *J* = 8.3 Hz, 1H), 5.19 (d, *J* = 16.2 Hz, 1H), 5.04 (d, *J* = 16.2 Hz, 1H), 4.52 (d, *J* = 10.4 Hz, 1H), 4.24 (d, *J* = 13.5 Hz, 1H), 4.06 (t, *J* = 10.7 Hz, 1H), 3.22 (s, 3H), 3.02 (dd, *J* = 13.5, 11.1 Hz, 1H), 2.34 (s, 3H). **^{13}C NMR (176 MHz, CDCl$_3$)** δ 172.2, 169.2, 137.2, 137.0, 136.9, 133.6, 133.2, 129.5, 129.0, 129.0, 128.9, 128.8, 128.8, 128.7, 127.3, 126.7, 124.1, 116.4, 66.5, 60.1, 54.3, 52.0, 48.9, 46.2, 20.9. **HRMS(ESI):** $[M+H]^+$ calcd. $C_{27}H_{26}O_3N_2Cl$ m/z 461.1627, found 461.1630. **HPLC conditions**: CHIRAPAK IC column, *iso*-propanol / *iso*-hexane = 40/60, flow rate = 0.5 mL min^{-1}, major enantiomer: t_R = 56.5 min; minor enantiomer: t_R = 25.4 min. *ee*=97%. **$[\alpha]_D^{20}$** = -103.2 (c = 0.68, CH_2Cl_2).

Methyl (2*R*,3*R*,3a*S*,9b*S*)-5-benzyl-3-(2-chlorophenyl)-8-methyl-4-oxo-2,3,3a,4,5,9b-hexahydro-1*H*-pyrrolo[3,2-*c*]quinoline-2-carboxylate (3s)

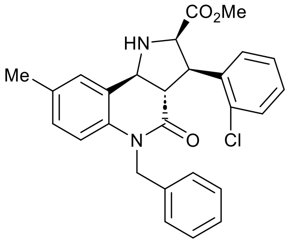

3s

The title product compound **3s** was prepared using general procedure C from **4s** (39.0 mg, 0.10 mmol) and isolated by column chromatography (2:1 *n*-pentane:EA) giving a solid (42.2 mg, 0.092 mmol, 92% yield).

^{1}H NMR (700 MHz, CDCl$_3$) δ 7.43 – 7.40 (m, 1H), 7.32 – 7.26 (m, 3H), 7.24 – 7.14 (m, 5H), 7.12 – 7.06 (m, 1H), 7.03 (d, *J* = 8.3 Hz, 1H), 6.91 (d, *J* = 8.3 Hz, 1H), 5.21 (d, *J* = 16.2 Hz, 1H), 5.07 (d, *J* = 16.2 Hz, 1H), 4.72 (d, *J* = 10.1 Hz, 1H), 4.58 (t, *J* = 10.9 Hz, 1H), 4.35 (d, *J* = 13.4 Hz, 1H), 3.23 (t, *J* = 12.5 Hz, 1H), 3.13 (s, 3H), 2.34 (s, 3H). **^{13}C NMR (176 MHz, CDCl$_3$)** δ 172.7, 168.8, 137.1, 136.9, 135.6, 135.5, 133.6, 129.6, 129.2, 128.8, 128.7, 128.4, 127.3, 127.3, 126.8, 126.8, 124.1, 116.3, 64.4, 59.9, 51.9, 51.3, 46.2, 45.9, 20.9. **HRMS(ESI):** $[M+H]^+$ calcd. $C_{27}H_{26}O_3N_2Cl$ m/z 461.1627, found 461.1625. **HPLC conditions**: CHIRAPAK IC column, *iso*-propanol / *iso*-hexane = 40/60, flow rate = 0.5 mL min^{-1}, major enantiomer: t_R = 56.5 min; minor enantiomer: t_R = 29.0 min. *ee*=94%. **$[\alpha]_D^{20}$** = -131.9 (c = 1.04, CH_2Cl_2).

Methyl (2*R*,3*R*,3a*S*,9b*S*)-8-bromo-5-(2-methylbenzyl)-4-oxo-3-phenyl-2,3,3a,4,5,9b-hexahydro-1*H*-pyrrolo[3,2-*c*]quinoline-2-carboxylate (3t)

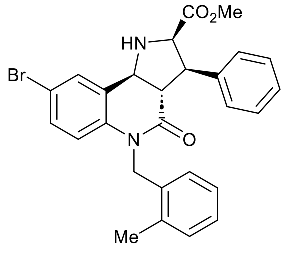

3t

The title product compound **3t** was prepared using general procedure C from **4t** (43.4 mg, 0.10 mmol) and isolated by column chromatography (2:1 *n*-pentane:EA) giving a solid (30.0 mg, 0.059 mmol, 59% yield).

^{1}H NMR (500 MHz, CDCl$_3$) δ 7.60 (dd, *J* = 2.4, 1.2 Hz, 1H), 7.32 – 7.25 (m, 3H), 7.22 – 7.18 (m, 3H), 7.17 – 7.11 (m, 2H), 7.06 (t, *J* = 7.4 Hz, 1H), 6.80 (d, *J* = 7.6 Hz, 1H), 6.68 (d, *J* = 8.7 Hz, 1H), 5.23 (d, *J* = 16.9 Hz, 1H), 4.86 (d, *J* = 16.9 Hz, 1H), 4.50 (d, *J* = 10.3 Hz, 1H), 4.29 (d, *J* = 13.6 Hz, 1H), 4.05 (t, *J* = 10.7 Hz, 1H), 3.15 (s, 3H), 3.13 (dd, *J* = 13.6, 11.1 Hz, 1H), 2.34 (s, 3H). **^{13}C NMR (126 MHz, CDCl$_3$)** δ 172.4, 169.1, 138.8, 138.3, 135.1, 133.8, 131.5, 131.2, 130.6, 128.5, 128.1, 127.4, 127.2, 126.6, 126.5, 125.0, 117.9, 116.7, 66.7, 59.8, 53.6, 51.9, 49.4, 44.6, 19.3. **HRMS(ESI):** $[M+H]^+$ calcd. $C_{27}H_{26}O_3N_2{}^{81}Br$ m/z 507.1101, found 507.1104. **HPLC conditions**: CHIRAPAK IC column, *iso*-propanol / *iso*-hexane = 40/60, flow rate = 0.5 mL min^{-1}, major enantiomer: t_R = 50.4 min; minor enantiomer: t_R = 39.8 min. *ee*=97%. **$[\alpha]_D^{20}$** = -82.0 (c = 0.20, CH_2Cl_2).

Methyl (2*R*,3*R*,3a*S*,9b*S*)-8-methyl-5-(2-methylbenzyl)-4-oxo-3-phenyl-2,3,3a,4,5,9b-hexahydro-1*H*-pyrrolo[3,2-*c*]quinoline-2-carboxylate (3u)

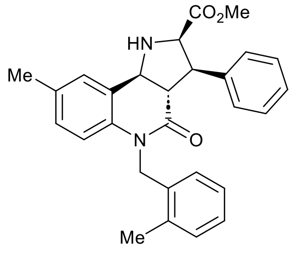

3u

The title product compound **3u** was prepared using general procedure C from **4u** (36.9 mg, 0.10 mmol) and isolated by column chromatography (2:1 *n*-pentane:EA) giving a solid (23.0 mg, 0.052 mmol, 52% yield).

^{1}H NMR (500 MHz, CDCl$_3$) δ 7.32 – 7.25 (m, 3H), 7.23 – 7.18 (m, 3H), 7.17 – 7.10 (m, 2H), 7.06 (t, *J* = 7.5 Hz, 1H), 7.00 (d, *J* = 7.5 Hz, 1H), 6.85 (d, *J* = 7.6 Hz, 1H), 6.73 (d, *J* = 8.2 Hz, 1H), 5.26 (d, *J* = 16.9 Hz, 1H), 4.86 (d, *J* = 16.8 Hz, 1H), 4.51 (d, *J* = 10.4 Hz, 1H), 4.28 (d, *J* = 13.5 Hz, 1H), 4.07 (t, *J* = 10.7 Hz, 1H), 3.15 (s, 3H), 3.10 (dd, *J* = 13.5, 11.1 Hz, 1H), 2.36 (s, 6H for two -CH_3). **^{13}C NMR (126 MHz, CDCl$_3$)** δ 172.5, 169.4, 138.7, 137.3, 135.0, 134.4, 133.5, 130.5, 129.4, 128.7, 128.5, 128.1, 127.2, 127.0, 126.4, 125.1, 124.0, 116.2, 66.9, 60.3, 54.5, 51.8, 49.7, 44.6, 20.9, 19.3. **HRMS(ESI):** $[M+H]^+$ calcd. $C_{28}H_{29}O_3N_2$ m/z 441.2173, found 441.2175. **HPLC conditions**: CHIRAPAK IC column, *iso*-propanol / *iso*-hexane = 40/60, flow rate = 0.5 mL min^{-1}, major enantiomer: t_R = 71.0 min; minor enantiomer: t_R = 45.7 min. *ee*=98%. **$[\alpha]_D^{20}$** = -125.5 (c = 0.27, CH_2Cl_2).

Methyl (2*R*,3*R*,3a*S*,9b*S*)-5-(3-bromobenzyl)-8-fluoro-4-oxo-3-phenyl-2,3,3a,4,5,9b-hexahydro-1*H*-pyrrolo[3,2-*c*]quinoline-2-carboxylate (3v)

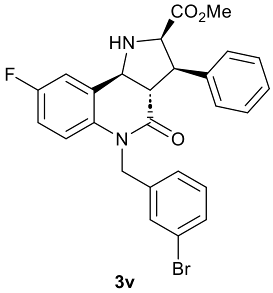

3v

The title product compound **3v** was prepared using general procedure C from **4v** (43.8 mg, 0.10 mmol) and isolated by column chromatography (2:1 *n*-pentane:EA) giving a solid (31.1 mg, 0.061 mmol, 61% yield).

^{1}H NMR (500 MHz, CDCl$_3$) δ 7.35 (d, *J* = 7.9 Hz, 1H), 7.32 – 7.26 (m, 3H), 7.24 – 7.18 (m, 4H), 7.14 (t, *J* = 7.8 Hz, 1H), 7.07 (d, *J* = 7.7 Hz, 1H), 6.94 – 6.86 (m, 2H), 5.14 (d, *J* = 16.3 Hz, 1H), 5.01 (d, *J* = 16.3 Hz, 1H), 4.50 (d, *J* = 10.4 Hz, 1H), 4.23 (d, *J* = 13.6 Hz, 1H), 4.06 (t, *J* = 10.7 Hz, 1H), 3.14 (s, 3H), 3.11 (dd, *J* = 13.6, 11.2 Hz, 1H). **^{13}C NMR (126 MHz, CDCl$_3$)** δ 172.3, 169.2, 159.2 (d, *J* = 245.3 Hz), 139.2, 138.1, 135.3 (d, *J* = 2.9 Hz), 131.8 (d, *J* = 7.5 Hz), 130.7, 130.5, 129.8, 128.6, 128.1, 127.5, 125.3, 123.1, 117.5 (d, *J* = 8.2 Hz), 114.6 (d, *J* = 22.6 Hz), 111.4 (d, *J* = 24.4 Hz), 66.6, 59.8, 53.3, 51.9, 49.5, 45.9. **^{19}F NMR (470 MHz, CDCl$_3$)** δ -118.0 (td, *J* = 8.0, 4.5 Hz). **HRMS(ESI):** $[M+H]^+$ calcd. $C_{26}H_{23}O_3N_2BrF$ m/z 509.0871, found 509.0874. **HPLC conditions**: CHIRAPAK IC column, *iso*-propanol / *iso*-hexane = 40/60, flow rate = 0.5 mL min^{-1}, major enantiomer: t_R = 41.1 min; minor enantiomer: t_R = 26.6 min. *ee*=98%. **$[\alpha]_D^{20}$** = -80.7 (c = 0.25, CH_2Cl_2).

Methyl (2*R*,3*R*,3a*S*,9b*S*)-5-(3-bromobenzyl)-8-methyl-4-oxo-3-phenyl-2,3,3a,4,5,9b-hexahydro-1*H*-pyrrolo[3,2-*c*]quinoline-2-carboxylate (3w)

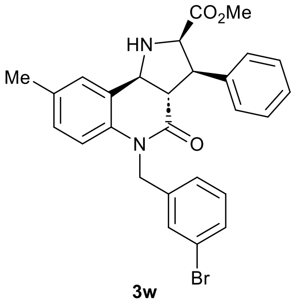

3w

The title product compound **3w** was prepared using general procedure C from **4w** (43.4 mg, 0.10 mmol) and isolated by column chromatography (2:1 *n*-pentane:EA) giving a solid (32.6 mg, 0.065 mmol, 65% yield).

^{1}H NMR (500 MHz, CDCl$_3$) δ 7.35 – 7.31 (m, 2H), 7.30 – 7.26 (m, 3H), 7.23 – 7.18 (m, 3H), 7.12 (t, *J* = 7.6 Hz, 1H), 7.07 (d, *J* = 7.8 Hz, 1H), 7.04 (d, *J* = 8.2 Hz, 1H), 6.84 (d, *J* = 8.2 Hz, 1H), 5.12 (d, *J* = 16.2 Hz, 1H), 5.02 (d, *J* = 16.3 Hz, 1H), 4.53 (d, *J* = 10.4 Hz, 1H), 4.24 (d, *J* = 13.5 Hz, 1H), 4.08 (t, *J* = 10.8 Hz, 1H), 3.14 (s, 3H), 3.08 (dd, *J* = 13.6, 11.1 Hz, 1H), 2.35 (s, 3H). **^{13}C NMR (126 MHz, CDCl$_3$)** δ 172.3, 169.3, 139.5, 138.4, 136.7, 133.8, 130.6, 130.4, 129.8, 129.1, 128.8, 128.5, 128.1, 127.4, 125.4, 124.3, 123.0, 116.1, 66.7, 60.1, 54.0, 51.9, 49.5, 45.6, 20.9. **HRMS(ESI):** $[M+H]^+$ calcd. $C_{27}H_{26}O_3N_2Br$ m/z 505.1121, found 505.1127. **HPLC conditions**: CHIRAPAK IC column, *iso*-propanol / *iso*-hexane = 40/60, flow rate = 0.5 mL min^{-1}, major enantiomer: t_R = 54.9 min; minor enantiomer: t_R = 33.0 min. *ee*=98%. **$[\alpha]_D^{20}$** = -98.1 (c = 0.74, CH_2Cl_2).

Methyl (2*R*,3*R*,3a*S*,9b*S*)-5-(4-chlorobenzyl)-8-methoxy-4-oxo-3-phenyl-2,3,3a,4,5,9b-hexahydro-1*H*-pyrrolo[3,2-*c*]quinoline-2-carboxylate (3x)

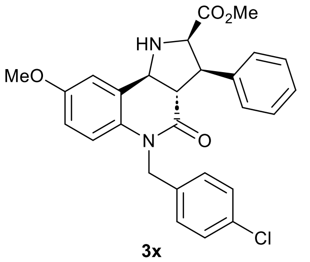

3x

The title product compound **3x** was prepared using general procedure C from **4x** (40.6 mg, 0.10 mmol) and isolated by column chromatography (1:1 *n*-pentane:EA) giving a solid (27.6 mg, 0.058 mmol, 58% yield).

^{1}H NMR (500 MHz, $CDCl_3$) δ 7.31 – 7.26 (m, 2H), 7.26 – 7.18 (m, 5H), 7.13 – 7.10 (m, 2H), 7.03 (dd, *J* = 2.9, 1.2 Hz, 1H), 6.87 (d, *J* = 8.9 Hz, 1H), 6.74 (dd, *J* = 8.9, 2.9 Hz, 1H), 5.12 (d, *J* = 16.1 Hz, 1H), 5.00 (d, *J* = 16.2 Hz, 1H), 4.49 (d, *J* = 10.4 Hz, 1H), 4.19 (d, *J* = 13.5 Hz, 1H), 4.06 (t, *J* = 10.7 Hz, 1H), 3.82 (s, 3H), 3.14 (s, 3H), 3.04 (dd, *J* = 13.5, 11.0 Hz, 1H). **^{13}C NMR (126 MHz, $CDCl_3$)** δ 172.4, 169.2, 156.2, 138.6, 135.8, 133.1, 132.5, 131.1, 129.0, 128.5, 128.3, 128.1, 127.3, 117.4, 113.0, 109.5, 66.8, 60.3, 55.8, 54.4, 51.9, 49.7, 45.8. **HRMS(ESI):** $[M+H]^+$ calcd. $C_{27}H_{26}O_4N_2Cl$ m/z 477.1576, found 477.1581. **HPLC conditions**: CHIRAPAK IC column, *iso*-propanol / *iso*-hexane = 40/60, flow rate = 0.5 mL min^{-1}, major enantiomer: t_R = 25.2 min; minor enantiomer: t_R = 31.8 min. *ee*=99%. **$[\alpha]_D^{20}$** = -87.2 (c = 0.21, CH_2Cl_2).

Methyl (2*R*,3*R*,3a*S*,9b*S*)-8-fluoro-5-methyl-4-oxo-3-phenyl-2,3,3a,4,5,9b-hexahydro-1*H*-pyrrolo[3,2-*c*]quinoline-2-carboxylate (3y)

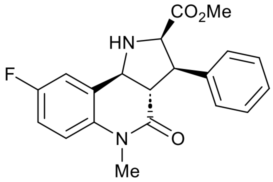

3y

The title product compound **3y** was prepared using general procedure C from **4y** (28.3 mg, 0.10 mmol) and isolated by column chromatography (2:1 *n*-pentane:EA) giving a solid (23.4 mg, 0.066 mmol, 66% yield).

^{1}H NMR (700 MHz, $CDCl_3$) δ 7.28 – 7.23 (m, 3H), 7.22 – 7.19 (m, 1H), 7.19 – 7.16 (m, 2H), 7.07 – 6.98 (m, 2H), 4.53 (d, *J* = 10.5 Hz, 1H), 4.24 (d, *J* = 13.8 Hz, 1H), 4.01 (t, *J* = 10.8 Hz, 1H), 3.32 (s, 3H), 3.13 (s, 3H), 3.00 (dd, *J* = 13.7, 11.2 Hz, 1H). **^{13}C NMR (176 MHz, $CDCl_3$)** δ 171.9, 168.6, 158.9 (d, *J* = 245.0 Hz), 137.9, 136.4 (d, *J* = 2.9 Hz), 130.6 (d, *J* = 7.5 Hz), 128.5, 128.1, 127.5, 116.8 (d, *J* = 8.1 Hz), 114.6 (d, *J* = 22.5 Hz), 111.3 (d, *J* = 24.5 Hz), 66.4, 59.6, 53.0, 52.0, 49.2, 30.1. **^{19}F NMR (470 MHz, $CDCl_3$)** δ -118.7 (td, *J* = 8.0, 4.7 Hz). **HRMS(ESI):** $[M+H]^+$ calcd. $C_{20}H_{20}O_3N_2F$ m/z 355.1453, found 355.1451. **HPLC conditions**: CHIRAPAK IC column, *iso*-propanol / *iso*-hexane = 40/60, flow rate = 0.5 mL min^{-1}, major enantiomer: t_R = 50.9 min; minor enantiomer: t_R = 37.6 min. *ee*=90%. **$[\alpha]_D^{20}$** = -123.0 (c = 0.50, CH_2Cl_2).

Methyl (2*R*,3*R*,3a*S*,9b*S*)-5-benzyl-7,8-difluoro-4-oxo-3-phenyl-2,3,3a,4,5,9b-hexahydro-1*H*-pyrrolo[3,2-*c*]quinoline-2-carboxylate (3z)

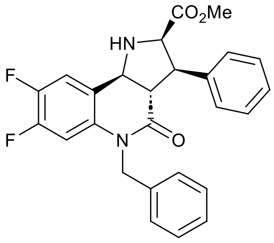

3z

The title product compound **3z** was prepared using general procedure C from **4z** (37.7 mg, 0.10 mmol) and isolated by column chromatography (2:1 *n*-pentane:EA) giving a solid (31.4 mg, 0.070 mmol, 70% yield).

^{1}H NMR (500 MHz, $CDCl_3$) δ 7.37 – 7.27 (m, 5H), 7.25 – 7.20 (m, 4H), 7.17 – 7.13 (m, 2H), 6.83 (dd, *J* = 11.8, 6.6 Hz, 1H), 5.11 (d, *J* = 16.2 Hz, 1H), 5.02 (d, *J* = 16.3 Hz, 1H), 4.56 (d, *J* = 10.4 Hz, 1H), 4.27 (d, *J* = 13.8 Hz, 1H), 4.08 (t, *J* = 10.8 Hz, 1H), 3.22 – 3.16 (m, 1H), 3.14 (s, 3H). **^{13}C NMR (176 MHz, $CDCl_3$)** δ 171.8, 168.9, 149.7 (dd, *J* = 247.1, 13.3 Hz), 146.3 (dd, *J* = 247.0, 12.8 Hz), 137.7, 136.1, 135.8 (dd, *J* = 8.0, 3.0 Hz), 129.1, 128.6, 128.1, 127.7, 127.6, 126.7, 113.2 (d, *J* = 19.8 Hz), 106.7 (d, *J* = 22.4 Hz), 66.4, 59.3, 53.0, 52.1, 49.1, 46.5. **^{19}F NMR (470 MHz, $CDCl_3$)** δ -136.6 (dd, *J* = 21.4, 10.4 Hz), -143.0 (dt, *J* = 21.7, 8.2 Hz). **HRMS(ESI):** $[M+H]^+$ calcd. $C_{26}H_{23}O_3N_2F_2$ m/z 449.1671, found 449.1673. **HPLC conditions**: CHIRAPAK IC column, *iso*-propanol / *iso*-hexane = 40/60, flow rate = 0.5 mL min^{-1}, major enantiomer: t_R = 35.0 min; minor enantiomer: t_R = 22.9 min. *ee*=96%. **$[\alpha]_D^{20}$** = -110.7 (c = 0.65, CH_2Cl_2).

Methyl (2*R*,3*R*,3a*S*,11b*S*)-5-benzyl-4-oxo-3-phenyl-2,3,3a,4,5,11b-hexahydro-1*H*-benzo[*g*]pyrrolo[3,2-*c*]quinoline-2-carboxylate (3aa)

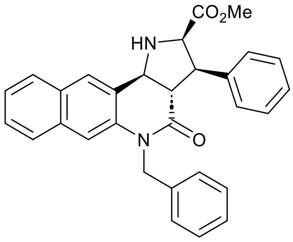

3aa

The title product compound **3aa** was prepared using general procedure C from **4aa** (39.1 mg, 0.10 mmol) and isolated by column chromatography (2:1 *n*-pentane:EA) giving a solid (27.3 mg, 0.059 mmol, 59% yield).

^{1}H NMR (500 MHz, $CDCl_3$) δ 7.90 (s, 1H), 7.85 – 7.80 (m, 1H), 7.70 – 7.63 (m, 1H), 7.47 – 7.41 (m, 2H), 7.37 (s, 1H), 7.32 – 7.26 (m, 4H), 7.25 – 7.18 (m, 6H), 5.32 (d, *J* = 16.1 Hz, 1H), 5.18 (d, *J* = 16.2 Hz, 1H), 4.62 (d, *J* = 10.4 Hz, 1H), 4.43 (d, *J* = 13.0 Hz, 1H), 4.17 (t, *J* = 10.7 Hz, 1H), 3.17 (dd, *J* = 13.1, 11.2 Hz, 1H), 3.16 (s, 3H).**^{13}C NMR (176 MHz, $CDCl_3$)** δ 172.2, 169.1, 138.2, 137.5, 136.9, 133.2, 129.9, 129.0, 128.9, 128.6, 128.1, 127.7, 127.7, 127.5, 127.4, 126.9, 126.8, 125.9, 122.4, 113.8, 66.7, 60.5, 54.1, 52.1, 49.6, 46.8. **HRMS(ESI):** $[M+H]^+$ calcd. $C_{30}H_{27}O_3N_2$ m/z 463.2016, found 463.2019. **HPLC conditions**: CHIRAPAK IC column, *iso*-propanol / *iso*-hexane = 40/60, flow rate = 0.5 mL min^{-1}, major enantiomer: t_R = 83.6 min; minor enantiomer: t_R = 45.4 min. *ee*=93%. **$[\alpha]_D^{20}$** = -10.9 (c = 0.25, CH_2Cl_2).

Methyl (2R,3R,3aS,9bS)-5-benzyl-3-(4-cyanophenyl)-4-oxo-2,3,3a,4,5,9b-hexahydro-1*H*-pyrrolo[3,2-c]quinoline-2-carboxylate (3bb)

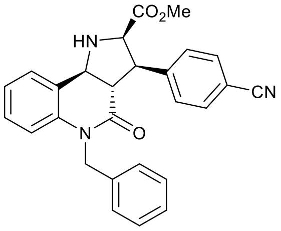

3bb

The title product compound **3bb** was prepared using general procedure C from **4bb** (36.6 mg, 0.10 mmol) and isolated by column chromatography (2:1 *n*-pentane:EA) giving a solid (40.0 mg, 0.091 mmol, 91% yield).

^{1}H NMR, **^{13}C NMR** and **HRMS (ESI)** are identical with the reported ones.[13] **HPLC conditions**: CHIRAPAK IA column, *iso*-propanol / *iso*-hexane = 30/70, flow rate = 0.5 mL min^{-1}, major enantiomer: t_R = 33.3 min; minor enantiomer: t_R = 53.9 min. *ee*=4%. **$[\alpha]_D^{20}$** = -17 (c = 0.2, CH_2Cl_2).

Methyl (2R,3R,3aS,9bS)-5-(4-cyanobenzyl)-4-oxo-3-phenyl-2,3,3a,4,5,9b-hexahydro-1*H*-pyrrolo[3,2-c]quinoline-2-carboxylate (3cc)

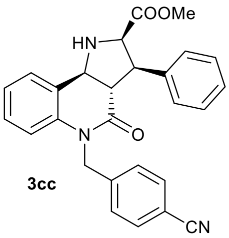

3cc

The title product compound **3cc** was prepared using general procedure C from **4cc** (36.6 mg, 0.10 mmol) and isolated by column chromatography (2:1 *n*-pentane:EA) giving a solid (41.0 mg, 0.094 mmol, 94% yield).

^{1}H NMR (700 MHz, $CDCl_3$) δ 7.59 – 7.53 (m, 3H), 7.30 – 7.29 (m, 1H), 7.28 (d, *J* = 8.0 Hz, 3H), 7.25 – 7.22 (m, 2H), 7.22 – 7.20 (m, 2H), 7.16 (td, *J* = 7.5, 1.0 Hz, 1H), 6.88 (d, *J* = 8.2 Hz, 1H), 5.28 (d, *J* = 16.8 Hz, 1H), 5.04 (d, *J* = 16.7 Hz, 1H), 4.62 (d, *J* = 10.5 Hz, 1H), 4.38 (d, *J* = 13.7 Hz, 1H), 4.11 (t, *J* = 10.8 Hz, 1H), 3.21 – 3.17 (m, 1H), 3.14 (s, 3H). **^{13}C NMR (176 MHz, $CDCl_3$)** δ 171.6, 169.1, 142.5, 138.9, 137.8, 132.8, 128.8, 128.6, 128.3, 128.1, 127.6, 127.4, 124.3, 124.0, 118.7, 115.9, 111.5, 66.4, 59.9, 53.3, 52.2, 49.0, 46.2. **HRMS(ESI):** $[M+H]^+$ calcd. $C_{27}H_{24}O_3N_3$ m/z 438.1812, found 438.1810. **HPLC conditions**: CHIRAPAK IA column, *iso*-propanol / *iso*-hexane = 30/70, flow rate = 0.5 mL min^{-1}, major enantiomer: t_R = 39.6 min; minor enantiomer: t_R = 54.1 min. *ee*=88%. **$[\alpha]_D^{20}$** = -123.5 (c = 0.2, CH_2Cl_2).

5. Cheminformatic analysis

The ChEMBL website was accessed on 12/09/2022 using version ChEMBL 31. Assays that evaluated either function or binding of the SMO homolog from homo sapiens (CHEMBL5971) and the SMO homolog from Mus musculus (CHEMBL6080) were selected. For high confidence, only entries that had "confidence label 9" (direct single protein target assigned) were selected. To exclude SMO agonists, only compounds that had IC_{50} values were used. Furthermore, to exclude compounds that do not have significant potency, entries that had IC_{50} values ≥ 10 µM were excluded. From the resulting entries, duplicate ChEMBL IDs were deleted resulting in 578 unique ChEMBL IDs. A natural product-likeness score, quantitative estimation of drug-likeness, and principal moments of inertia of the pseudo-natural products and SMO antagonists were computed using the open-source software RDKit (Open-source cheminformatics; http://www.rdkit.org.).

A natural product likeness-score (NP_Like) and quantitative estimation of drug-likeness (QED) were calculated for the psuedo-natural product *ent*-**3a** and 578 SMO antagonists (SmoAnta):

No.	Compound ID	Smiles	DataSet	NP_Like	QED
1	*ent*-**3a**	O=C1N(CC2=CC=CC=C2)C3=CC=CC=C3[C@H]4[C@H]1[C@@H](C5=CC=CC=C5)[C@@H](C(OC)=O)N4	PNP	-0.33	0.662
2	CHEMBL4632769	Cc1cc(C)c(-c2ncc[nH]2)cc1NC(=O)c1ccc(OCc2ccccn2)cc1	SmoAnta	-1.73	0.483
3	CHEMBL2160074	C[C@@H]1CN(c2ccc3c(=Nc4ccc(Cl)c(-c5nc(-c6ccccc6)c[nH]5)c4)[nH]ccc3c2)CCO1	SmoAnta	-1.1	0.306
4	CHEMBL2160077	Clc1ccc(N=c2[nH]ccc3cc(N4CCOCC4)ccc23)cc1-c1nc(-c2ccccc2)c[nH]1	SmoAnta	-1.1	0.333
5	CHEMBL2160072	C[C@H]1CN(c2ccc3c(=Nc4ccc(Cl)c(-c5nc(-c6ccccc6)c[nH]5)c4)[nH]ccc3c2)C[C@@H](C)O1	SmoAnta	-0.95	0.282
6	CHEMBL4637222	Cc1cc(C)c(-c2cn(C)cn2)cc1NC(=O)c1ccc(OCc2ccccn2)cc1	SmoAnta	-1.67	0.488
7	CHEMBL2160068	C[C@H]1CN(c2ccc3c(=Nc4ccc(Cl)c(-c5nc(-c6ccccc6)c[nH]5)c4)[nH]ccc3n2)C[C@@H](C)O1	SmoAnta	-1.07	0.305
8	CHEMBL2160078	Cc1ccc(NC(=O)c2ccc(N3CCOCC3)cc2)cc1-c1nc(-c2ccccc2)c[nH]1	SmoAnta	-1.5	0.453
9	CHEMBL2160070	C[C@H]1CN(c2ccc3c(Nc4ccc(Cl)c(-c5nc(-c6ccccc6)c[nH]5)c4)cccc3n2)C[C@@H](C)O1	SmoAnta	-1.33	0.26
10	CHEMBL1083915	Cc1ccc(-c2nnc(N3CCN(C(=O)c4ccccc4)C[C@H]3C)c3ccccc23)cc1	SmoAnta	-1.29	0.468
11	CHEMBL473417	CS(=O)(=O)c1ccc(C(=O)Nc2ccc(Cl)c(-c3ccccn3)c2)c(Cl)c1	SmoAnta	-2.12	0.662
12	CHEMBL2160071	C[C@H]1CN(c2ccc3c(=Nc4ccc(Cl)c(-c5nc(-c6ccccc6)c[nH]5)c4)cc[nH]c3c2)C[C@@H](C)O1	SmoAnta	-1.02	0.282
13	CHEMBL2160079	COc1cc(OC)cc(C(=O)Nc2ccc(C)c(-c3nc(-c4ccccc4)c[nH]3)c2)c1	SmoAnta	-1.06	0.448
14	CHEMBL2160076	Clc1ccc(N=c2[nH]ccc3cc(N4CCCCC4)ccc23)cc1-c1nc(-c2ccccc2)c[nH]1	SmoAnta	-0.97	0.289
15	CHEMBL2160067	C[C@@H]1CN(c2ccc3c(=Nc4ccc(Cl)c(-c5nc(-c6ccccc6)c[nH]5)c4)[nH]ccc3n2)CCO1	SmoAnta	-1.24	0.333
16	CHEMBL1086467	C[C@@H]1CN(C(=O)c2ccccc2)CCN1c1nnc(-c2ccc(Cl)cc2)c2ccccc12	SmoAnta	-1.38	0.432
17	CHEMBL1209455	C[C@@H]1CN(C(=O)c2ccccc2)CCN1c1nnc(-c2ccc(C(F)(F)F)cc2)c2ccncc12	SmoAnta	-1.41	0.413
18	CHEMBL4288592	CN(C(=O)c1ccc(Oc2ccccc2)cc1C(F)(F)F)C1CCN(c2nnc(-c3ccnn3C)c3ccccc23)CC1	SmoAnta	-1.44	0.224
19	CHEMBL497266	CC(C)(N)c1ccc(N2CCN(c3nnc(Cc4ccccc4)c4ccccc34)CC2)nc1	SmoAnta	-1.2	0.505
20	CHEMBL538867	CC1=C2C[C@H]3[C@@H](CC[C@@H]4C[C@H](NS(C)(=O)=O)CC[C@]34C)[C@@H]2CC[C@@]2(C1)O[C@@H]1C[C@H](C)CN[C@H]1[C@H]2C	SmoAnta	1.79	0.518
21	CHEMBL2160073	O=S1(=O)CCN(c2ccc3c(=Nc4ccc(Cl)c(-c5nc(-c6ccccc6)c[nH]5)c4)[nH]ccc3c2)CC1	SmoAnta	-1.13	0.328

22	CHEMBL1084835	C[C@@H]1CN(C(=O)c2ccccc2)CCN1c1nnc(-c2ccccc2)c2ccccc12	SmoAnta	-1.22	0.495
23	CHEMBL1084730	C[C@@H]1CN(C(=O)c2ccccc2)CCN1c1nnc(-c2ccc(C3CC3)cc2)c2ccccc12	SmoAnta	-1.12	0.412
24	CHEMBL2059865	Cc1ccc(-c2ncc[nH]2)cc1NC(=O)c1ccc(OCc2ccccn2)cc1	SmoAnta	-1.81	0.509
25	CHEMBL2059863	Cc1ccc(-c2nc(C(F)(F)F)c[nH]2)cc1NC(=O)c1ccc(OCc2ccccn2)cc1	SmoAnta	-1.7	0.397
26	CHEMBL2142592	CN(C(=O)c1ccc(F)cc1C(F)(F)F)C1CCN(c2nnc(-c3ccnn3C)c3ccccc23)CC1	SmoAnta	-1.75	0.363
27	CHEMBL2059864	Cc1c[nH]c(-c2ccc(C)c(NC(=O)c3ccc(OCc4ccccn4)cc3)c2)n1	SmoAnta	-1.77	0.483
28	CHEMBL2160208	O=C(NC1CCN(Cc2ccc(N3CCC(NC(=O)c4cccc(-c5cccnc5)c4)CC3)nc2)CC1)c1cccc(-c2cccnc2)c1	SmoAnta	-1.2	0.202
29	CHEMBL1084734	C[C@@H]1CN(C(=O)c2ccccc2)CCN1c1nnc(-c2ccc(CO)cc2)c2ccccc12	SmoAnta	-1.01	0.519
30	CHEMBL485870	CC(C)(O)c1ccc(N2CCN(c3nnc(Cc4ccccc4)c4ccccc34)CC2)nc1	SmoAnta	-1.08	0.504
31	CHEMBL1084738	C[C@@H]1CN(C(=O)c2ccccc2)CCN1c1nnc(-c2ccc(C(F)(F)F)cc2)c2ccccc12	SmoAnta	-1.37	0.383
32	CHEMBL1209454	C[C@@H]1CN(C(=O)c2ccccc2)CCN1c1nnc(-c2ccccc2)c2ccncc12	SmoAnta	-1.27	0.511
33	CHEMBL2160069	C[C@H]1CN(c2cnc3c(Nc4ccc(Cl)c(-c5nc(-c6ccccc6)c[nH]5)c4)cccc3n2)C[C@@H](C)O1	SmoAnta	-1.22	0.274
34	CHEMBL4539839	Cc1c(O)n(-c2ccc(Cl)cc2)c(=O)n1Cc1ccc(N=[N+]=[N-])cc1	SmoAnta	-1.16	0.429
35	CHEMBL516246	COc1cc(OC)cc(C(=O)Nc2ccc(Cl)c(-c3nc4cc(N(C)C)ccc4[nH]3)c2)c1	SmoAnta	-1.52	0.421
36	CHEMBL1084736	C[C@@H]1CN(C(=O)c2ccccc2)CCN1c1nnc(-c2ccc(C#N)cc2)c2ccccc12	SmoAnta	-1.46	0.475
37	CHEMBL1083284	Cc1ccc(-c2nnc(N3CCN(C(=O)c4ccccc4)[C@@H](C)C3)c3ccccc23)cc1	SmoAnta	-1.33	0.468
38	CHEMBL1209189	CC(=O)Oc1ccc(-c2nnc(N3CCN(C(=O)c4ccccc4)C[C@H]3C)c3ccccc23)cc1	SmoAnta	-0.99	0.324
39	CHEMBL4278388	CN(C(=O)c1ccc(F)cc1C(F)(F)F)C1CCN(c2ccc(-c3ccnn3C)c3ccccc23)CC1	SmoAnta	-1.55	0.31
40	CHEMBL1083914	C[C@H]1CN(c2nnc(-c3ccc(C(F)(F)F)cc3)c3ccccc23)CCN1C(=O)c1ccccc1	SmoAnta	-1.4	0.383
41	CHEMBL4441310	Cc1ccc(=NCc2ccnn2-c2nnc(N3CCC(C(=O)NC4CCCCC4C)CC3)s2)[nH]c1	SmoAnta	-1.53	0.547
42	CHEMBL1813106	O=C(Nc1cccnc1-c1cccc(Cl)c1)N1CCn2c(c(O)n([C@H]3C[C@@H]3c3ccccc3)c2=O)C1	SmoAnta	-1.09	0.418
43	CHEMBL474507	Cc1csc(CN[C@H]2Cc3ccc(NC(=O)c4cccc(C)c4-c4ccc(C(F)(F)F)cc4)cc3C2)n1	SmoAnta	-1.45	0.289
44	CHEMBL1085503	Cc1ccc(-c2nnc(N3CCN(C(=O)c4ccccc4)CC3)c3ccccc23)cc1	SmoAnta	-1.36	0.498
45	CHEMBL1209190	C[C@@H]1CN(C(=O)c2ccccc2)CCN1c1nnc(-c2ccc(CC(N)=O)cc2)c2ccccc12	SmoAnta	-1.21	0.485
46	CHEMBL2059859	Cc1ccc(-c2nc3ccccc3[nH]2)cc1NC(=O)c1ccc(OCc2ccccn2)cc1	SmoAnta	-1.72	0.356
47	CHEMBL2059866	Cc1ccc(-c2cn(C)cn2)cc1NC(=O)c1ccc(OCc2ccccn2)cc1	SmoAnta	-1.74	0.514
48	CHEMBL561533	CCCC(CCC)C(=O)NCc1ccc2c(cnn2-c2ccccc2OC)c1	SmoAnta	-1.21	0.577
49	CHEMBL2031290	COc1cc(C(=O)N=C(N)Nc2ccc(C)c(NC(=O)c3ccc(-c4ccccc4)cc3)c2)cc(OC)c1OC	SmoAnta	-0.92	0.2
50	CHEMBL2043435	CN1CC[C@@H](NC(=O)Nc2ccc(C(F)(F)F)cc2)CC1c1nc2ccccc2[nH]1	SmoAnta	-1.54	0.586
51	CHEMBL2043437	CN1CC[C@@H](NC(=O)Nc2ccc(C#N)cc2)CC1c1nc2ccccc2[nH]1	SmoAnta	-1.66	0.655
52	CHEMBL2043430	O=C(Nc1cccc(-c2nc3ccccc3[nH]2)c1)c1ccc2c(c1)OCCO2	SmoAnta	-1.46	0.564
53	CHEMBL1086048	C[C@H]1CN(C(=O)c2ccccc2)CCN1c1nnc(-c2ccccc2)c2ccccc12	SmoAnta	-1.22	0.495
54	CHEMBL1086045	C[C@H]1CN(c2nnc(-c3ccccc3)c3ccccc23)CCN1C(=O)c1ccccc1	SmoAnta	-1.26	0.495
55	CHEMBL1084737	C[C@@H]1CN(C(=O)c2ccccc2)CCN1c1nnc(-c2ccc(N(C)C)cc2)c2ccccc12	SmoAnta	-1.35	0.45
56	CHEMBL1084837	O=C(c1ccccc1)N1CCN(c2nnc(-c3ccc(Cl)cc3)c3ccccc23)CC1	SmoAnta	-1.45	0.465
57	CHEMBL3604610	CC(=O)N=c1[nH]c2ccc(-c3cnc(Cl)c(NC(=O)c4ccccc4)c3)cc2s1	SmoAnta	-1.62	0.471
58	CHEMBL471672	Cc1ccc(CN[C@H]2Cc3ccc(NC(=O)c4cccc(C)c4-c4ccc(C(F)(F)F)cc4)cc3C2)o1	SmoAnta	-1.16	0.295
59	CHEMBL1824915	O=C(Nc1ccc(-c2cccc(-c3ccccc3)c2)cc1)c1ccccc1Cl	SmoAnta	-1.18	0.407
60	CHEMBL2059867	Cc1nc(-c2ccc(C)c(NC(=O)c3ccc(OCc4ccccn4)cc3)c2)c[nH]1	SmoAnta	-1.79	0.483
61	CHEMBL1083285	C[C@H]1CN(c2nnc(-c3ccc(Cl)cc3)c3ccccc23)CCN1C(=O)c1ccccc1	SmoAnta	-1.42	0.432
62	CHEMBL1813111	O=C(N=c1cc[nH]n1-c1cccc(F)c1)N1CCn2c(c(O)n([C@H]3C[C@@H]3c3ccccc3)c2=O)C1	SmoAnta	-0.82	0.478
63	CHEMBL474503	Cc1cccc(C(=O)Nc2ccc3c(c2)C[C@@H](NCc2cccs2)C3)c1-c1ccc(C(F)(F)F)cc1	SmoAnta	-1.44	0.289
64	CHEMBL4441529	Cc1ccc(=NCc2ccnn2-c2nnc(N3CCC(C(=O)N[C@H]4CCCC[C@H]4C)CC3)s2)[nH]c1	SmoAnta	-1.53	0.547
65	CHEMBL1084731	CC(C)c1ccc(-c2nnc(N3CCN(C(=O)c4ccccc4)C[C@H]3C)c3ccccc23)cc1	SmoAnta	-1.19	0.396
66	CHEMBL1824916	O=C(Nc1ccc(-c2cc(-c3ccccc3)ccn2)cc1)c1ccccc1Cl	SmoAnta	-1.44	0.447
67	CHEMBL1209314	C[C@@H]1CN(C(=O)c2ccccc2)CCN1c1nnc(-n2ccc3ccccc32)c2ccccc12	SmoAnta	-1.11	0.394
68	CHEMBL473896	Cc1cccc(C(=O)Nc2ccc3c(c2)C[C@@H](NCc2ccccc2)C3)c1-c1ccc(C(F)(F)F)cc1	SmoAnta	-0.98	0.294
69	CHEMBL4567678	Cc1ccc(=NCc2ccnn2-c2nnc(N3CCC(C(=O)N[C@@H]4CCCC[C@@H]4C)CC3)s2)[nH]c1	SmoAnta	-1.53	0.547
70	CHEMBL1209251	C[C@@H]1CN(C(=O)c2ccccc2)CCN1c1nnc(-n2cccc2)c2ccccc12	SmoAnta	-1.29	0.528
71	CHEMBL2059871	Cc1ccc(-c2cc(C(F)(F)F)[nH]n2)cc1NC(=O)c1ccc(OCc2ccccn2)cc1	SmoAnta	-1.92	0.397
72	CHEMBL2059868	Cc1ccc(-c2[nH]cnc2C)cc1NC(=O)c1ccc(OCc2ccccn2)cc1	SmoAnta	-1.63	0.483

73	CHEMBL4472296	C[C@]1(c2ccc(Cl)cc2Cl)OC[C@@H](COc2ccc(N3CCN(c4ccc(NC(=O)NN)cc4)CC3)cc2)O1	SmoAnta	-0.8	0.209
74	CHEMBL1083104	O=C(c1nccs1)N1CCN(c2nnc(-c3ccccc3)c3ccccc23)CC1	SmoAnta	-1.5	0.524
75	CHEMBL3965352	Cc1ccc2c(n1)C(C(C)C(=O)Nc1ccc(Cl)c(-c3nc4ccccc4n3C)c1)CC[C@H]2C	SmoAnta	-0.78	0.353
76	CHEMBL4159710	COc1cc(C(=O)N=C(N)Nc2cccc(NC(=O)c3ccc(-c4ccccc4)cc3)c2)cc(OC)c1OC	SmoAnta	-0.74	0.214
77	CHEMBL1084733	C[C@@H]1CN(C(=O)c2ccccc2)CCN1c1nnc(-c2ccc(C(C)(C)C)cc2)c2ccccc12	SmoAnta	-1.28	0.376
78	CHEMBL3604612	CC(=O)N=c1[nH]c2ccc(-c3cnc(Cl)c(NC(=O)c4cccc(Cl)c4)c3)cc2s1	SmoAnta	-1.8	0.41
79	CHEMBL474893	Cc1cccc(C(=O)Nc2ccc3c(c2)C[C@@H](NCc2nccs2)C3)c1-c1ccc(C(F)(F)F)cc1	SmoAnta	-1.38	0.307
80	CHEMBL519219	OC1(c2ccc(N3CCN(c4nnc(Cc5ccccc5)c5ccccc45)CC3)nc2)CC1	SmoAnta	-0.84	0.511
81	CHEMBL1083739	O=C(c1ccccc1)N1CCN(c2nnc(-c3ccc(F)cc3)c3ccccc23)CC1	SmoAnta	-1.53	0.501
82	CHEMBL1083605	C[C@H]1CN(c2nnc(-c3ccc(F)cc3)c3ccccc23)CCN1C(=O)c1ccccc1	SmoAnta	-1.49	0.473
83	CHEMBL1813105	O=C(Nc1cccnc1-c1ccccc1)N1CCn2c(c(O)n([C@H]3C[C@@H]3c3ccccc3)c2=O)C1	SmoAnta	-0.85	0.471
84	CHEMBL1209381	C[C@@H]1CN(C(=O)c2ccccc2)CCN1c1nnc(-c2ccccc2)c2cnccc12	SmoAnta	-1.26	0.511
85	CHEMBL1209074	C[C@H]1CN(C(=O)c2ccccc2)CCN1c1nnc(-c2ccc(F)cc2)c2ccccc12	SmoAnta	-1.45	0.473
86	CHEMBL2059870	Cc1ccc(-c2c(C)nn(C)c2C)cc1NC(=O)c1ccc(OCc2ccccn2)cc1	SmoAnta	-1.89	0.458
87	CHEMBL1083103	O=C(c1ccco1)N1CCN(c2nnc(-c3ccccc3)c3ccccc23)CC1	SmoAnta	-1.51	0.537
88	CHEMBL1086284	C[C@@H]1CN(c2nnc(-c3ccccc3)c3ccccc23)CCN1C(=O)c1ccccc1	SmoAnta	-1.26	0.495
89	CHEMBL2031277	COc1cc(C(=O)NC(=O)Nc2ccc(C)c(NC(=O)c3ccc(-c4ccccc4)cc3)c2)cc(OC)c1OC	SmoAnta	-1.11	0.257
90	CHEMBL1824904	O=C(Nc1ccc(NC(=O)c2ccccc2Cl)cc1)c1ccccc1	SmoAnta	-1.33	0.707
91	CHEMBL1813114	C[C@@]12CN(C(=O)N=c3cc[nH]n3-c3ccccc3)CCN1C(=O)N([C@H]1C[C@@H]1c1ccccc1)C2=O	SmoAnta	-0.3	0.597
92	CHEMBL2031288	COc1cc(C(=O)N=C(N)Nc2ccc(C)c(NC(=O)c3ccc(-c4ccc(F)cc4)cc3)c2)cc(OC)c1OC	SmoAnta	-1.07	0.189
93	CHEMBL1824905	O=C(Nc1ccc(CC(O)c2ccccc2)cc1)c1ccccc1Cl	SmoAnta	-1.01	0.689
94	CHEMBL514764	Cc1cccc(C(=O)Nc2ccc3c(c2)C[C@@H](NCc2ccco2)C3)c1-c1ccc(C(F)(F)F)cc1	SmoAnta	-1.17	0.313
95	CHEMBL495877	CN(C)Cc1ccc(N2CCN(c3nnc(Cc4ccccc4)c4ccccc34)CC2)nc1	SmoAnta	-1.44	0.453
96	CHEMBL1813109	O=C(N=c1cc[nH]n1-c1ccccc1)N1CCn2c(c(O)n([C@H]3C[C@@H]3c3ccccc3)c2=O)C1	SmoAnta	-0.52	0.496
97	CHEMBL1083738	COc1ccc(-c2nnc(N3CCN(C(=O)c4ccccc4)CC3)c3ccccc23)cc1	SmoAnta	-1.24	0.49
98	CHEMBL1082387	Cc1ccccc1-c1nnc(N2CCN(C(=O)c3ccccc3)CC2)c2ccccc12	SmoAnta	-1.28	0.498
99	CHEMBL1209380	C[C@@H]1CN(C(=O)c2ccccc2)CCN1c1nnc(-c2ccccc2)c2cccnc12	SmoAnta	-1.29	0.511
100	CHEMBL1813107	N#Cc1cccc(-c2ncccc2NC(=O)N2CCn3c(c(O)n([C@H]4C[C@@H]4c4ccccc4)c3=O)C2)c1	SmoAnta	-1.16	0.446
101	CHEMBL1084836	O=C(c1ccccc1)N1CCN(c2nnc(-c3ccc(Cl)c(Cl)c3)c3ccccc23)CC1	SmoAnta	-1.52	0.399
102	CHEMBL497238	C[C@H]1CN(c2ccc(C#N)cn2)CCN1c1nnc(Cc2ccccc2)c2ccccc12	SmoAnta	-1.58	0.493
103	CHEMBL1824909	O=C(Nc1ccc(-c2nc(-c3ccccc3)c(C(F)(F)F)o2)cc1)c1ccccc1Cl	SmoAnta	-1.53	0.372
104	CHEMBL497437	C[C@@H]1CN(c2nnc(Cc3ccccc3)c3ccccc23)CCN1c1ccc(C#N)cn1	SmoAnta	-1.53	0.493
105	CHEMBL520858	CC(O)(CO)c1ccc(N2CCN(c3nnc(Cc4ccccc4)c4ccccc34)CC2)nc1	SmoAnta	-0.91	0.462
106	CHEMBL3818447	Cn1c(-c2cc(N3CCc4sc(C(=O)N5CCOCC5)cc4C3)ccc2Cl)nc2ccccc21	SmoAnta	-1.81	0.404
107	CHEMBL1813110	O=C(N=c1cc[nH]n1-c1cccc(Cl)c1)N1CCn2c(c(O)n([C@H]3C[C@@H]3c3ccccc3)c2=O)C1	SmoAnta	-0.76	0.46
108	CHEMBL4532846	Cc1ccc(NCc2ccnn2-c2nnc(N3CCC(C(=O)NC4CCCCC4C)CC3)s2)cc1	SmoAnta	-1.76	0.502
109	CHEMBL4517976	CC1CCCCC1NC(=O)C1CCN(c2nnc(-n3nccc3CN=c3cccc[nH]3)s2)CC1	SmoAnta	-1.58	0.564
110	CHEMBL1209132	C[C@@H]1CN(C(=O)c2ccccc2)CCN1c1nnc(-c2ccc(C(N)=O)cc2)c2ccccc12	SmoAnta	-1.27	0.51
111	CHEMBL1082791	O=C(c1ccccc1)N1CCN(c2nnc(-c3ccccc3)c3ccccc23)CC1	SmoAnta	-1.29	0.521
112	CHEMBL3741651	O=C(NC(=O)c1ccc(OCc2ccccc2F)cc1)Nc1ccc(Cl)c(-c2nc3ccccc3[nH]2)c1	SmoAnta	-1.81	0.239
113	CHEMBL1813113	C[C@@]12CN(C(=O)Nc3ccccc3-c3ccccc3)CCN1C(=O)N([C@H]1C[C@@H]1c1ccccc1)C2=O	SmoAnta	-0.53	0.546
114	CHEMBL2160075	Clc1ccc(N=c2[nH]ccc3ccccc23)cc1-c1nc(-c2ccccc2)c[nH]1	SmoAnta	-0.78	0.379
115	CHEMBL1083740	COc1cccc(-c2nnc(N3CCN(C(=O)c4ccccc4)CC3)c3ccccc23)c1	SmoAnta	-1.33	0.49
116	CHEMBL495608	N#Cc1ccc(Cc2nnc(N3CCN(c4ccc(C#N)cn4)CC3)c3ccccc23)cc1	SmoAnta	-1.55	0.486
117	CHEMBL496604	N#Cc1ccc(N2CCN(c3nnc(Cc4ccc(Cl)cc4)c4ccccc34)CC2)nc1	SmoAnta	-1.76	0.466
118	CHEMBL2043436	CN1CC[C@@H](NC(=O)Nc2ccc(Cl)cc2)CC1c1nc2ccccc2[nH]1	SmoAnta	-1.57	0.635
119	CHEMBL4848651	CC(O)(CCCCCCOCCOCCOCc1cn(CCOCCOCCOCCOCCOCCS(=O)(=O)c2ccc(C(=O)Nc3ccc(Cl)c(-c4ccccn4)c3)c(Cl)c2)nn1)C1CC[C@H]2[C@@H]3CC=C4C[C@@H](O)CC[C@]4(C)[C@H]3CC[C@]12C	SmoAnta	-0.17	0.028

120	CHEMBL4873360	CC(O)(CCCCCCOCCOCCOCCOCCOCCOCCOCc1cn(CCOCCOCCOCCOCCOCCS(=O)(=O)c2ccc(C(=O)Nc3ccc(Cl)c(-c4ccccn4)c3)c(Cl)c2)nn1)C1CC[C@H]2[C@@H]3CC=C4C[C@@H](O)CC[C@]4(C)[C@H]3CC[C@]12C	SmoAnta	-0.15	0.027
121	CHEMBL4870539	CC(O)(CCCCCCOCCOCCOCCOCCOCCOCCOCCOCCOCc1cn(CCOCCOCCOCCOCCOCCS(=O)(=O)c2ccc(C(=O)Nc3ccc(Cl)c(-c4ccccn4)c3)c(Cl)c2)nn1)C1CC[C@H]2[C@@H]3CC=C4C[C@@H](O)CC[C@]4(C)[C@H]3CC[C@]12C	SmoAnta	-0.14	0.027
122	CHEMBL4858367	CC(O)(CCCCCCOCCOCc1cn(CCOCCOCCOCCOCCS(=O)(=O)c2ccc(C(=O)Nc3ccc(Cl)c(-c4ccccn4)c3)c(Cl)c2)nn1)C1CC[C@H]2[C@@H]3CC=C4C[C@@H](O)CC[C@]4(C)[C@H]3CC[C@]12C	SmoAnta	-0.18	0.031
123	CHEMBL4861427	CC(O)(CCCCCCOCCOCc1cn(CCOCCOCCOCCOCCOCCS(=O)(=O)c2ccc(C(=O)Nc3ccc(Cl)c(-c4ccccn4)c3)c(Cl)c2)nn1)C1CC[C@H]2[C@@H]3CC=C4C[C@@H](O)CC[C@]4(C)[C@H]3CC[C@]12C	SmoAnta	-0.17	0.029
124	CHEMBL4861774	CC(O)(CCCCCCOCCOCc1cn(CCOCCOCCOCCS(=O)(=O)c2ccc(C(=O)Nc3ccc(Cl)c(-c4ccccn4)c3)c(Cl)c2)nn1)C1CC[C@H]2[C@@H]3CC=C4C[C@@H](O)CC[C@]4(C)[C@H]3CC[C@]12C	SmoAnta	-0.19	0.034
125	CHEMBL4861638	CC(O)(CCCCCCOCCOCCOCCOCCOCc1cn(CCOCCOCCOCCOCCOCCS(=O)(=O)c2ccc(C(=O)Nc3ccc(Cl)c(-c4ccccn4)c3)c(Cl)c2)nn1)C1CC[C@H]2[C@@H]3CC=C4C[C@@H](O)CC[C@]4(C)[C@H]3CC[C@]12C	SmoAnta	-0.16	0.028
126	CHEMBL4846473	CC(O)(CCCCCCOCCOCc1cn(CCOCCOCCS(=O)(=O)c2ccc(C(=O)Nc3ccc(Cl)c(-c4ccccn4)c3)c(Cl)c2)nn1)C1CC[C@H]2[C@@H]3CC=C4C[C@@H](O)CC[C@]4(C)[C@H]3CC[C@]12C	SmoAnta	-0.19	0.04
127	CHEMBL1082396	O=C(c1ccccc1)N1CCN(c2nnc(-c3ccccc3Cl)c3ccccc23)CC1	SmoAnta	-1.4	0.465
128	CHEMBL1813096	O=C(Nc1ccccc1-c1ccccc1)N1CCn2c(c(O)n([C@H]3C[C@@H]3c3ccccc3)c2=O)C1	SmoAnta	-0.78	0.454
129	CHEMBL2059875	Cc1ccc(-c2ccccn2)cc1NC(=O)c1ccc(OCc2ccccn2)cc1	SmoAnta	-1.71	0.482
130	CHEMBL1209133	C[C@@H]1CN(C(=O)c2ccccc2)CCN1c1nnc(-c2ccc(N3CCOCC3)cc2)c2ccccc12	SmoAnta	-1.47	0.418
131	CHEMBL1813108	O=C(Nc1cccnc1-c1ccsc1)N1CCn2c(c(O)n([C@H]3C[C@@H]3c3ccccc3)c2=O)C1	SmoAnta	-1.31	0.463
132	CHEMBL2059869	Cc1ccc(-c2cn(C)c(C)n2)cc1NC(=O)c1ccc(OCc2ccccn2)cc1	SmoAnta	-1.92	0.488
133	CHEMBL1084301	O=C(c1cccs1)N1CCN(c2nnc(-c3ccccc3)c3ccccc23)CC1	SmoAnta	-1.78	0.514
134	CHEMBL1209248	C[C@@H]1CN(C(=O)c2ccccc2)CCN1c1nnc(-c2ccncc2)c2ccccc12	SmoAnta	-1.32	0.511
135	CHEMBL1824917	O=C(Nc1ccc(-c2ccnc(-c3ccccc3)n2)cc1)c1ccccc1Cl	SmoAnta	-1.71	0.489
136	CHEMBL1824908	O=C(Nc1ccc(-c2nc(-c3ccccc3)cs2)cc1)c1ccccc1Cl	SmoAnta	-1.96	0.439
137	CHEMBL1824918	O=C(Nc1ccc(-c2cncc(-c3ccccc3)c2)cc1)c1ccccc1Cl	SmoAnta	-1.36	0.447
138	CHEMBL2031263	COc1cc(C(=O)NC(=S)Nc2ccc(C)c(NC(=O)c3ccc(-c4ccc(F)cc4)cc3)c2)cc(OC)c1OC	SmoAnta	-1.41	0.216
139	CHEMBL474301	Cc1cccc(C(=O)Nc2ccc3c(c2)C[C@@H](NC(C)c2ccccc2)C3)c1-c1ccc(C(F)(F)F)cc1	SmoAnta	-0.98	0.276
140	CHEMBL4562943	C#CCOc1ccc(-n2c(O)c(C)n(Cc3ccc(N=[N+]=[N-])cc3)c2=O)cc1	SmoAnta	-1.21	0.308
141	CHEMBL1824919	O=C(Nc1ccc(-c2cccc(-c3ccccc3)n2)cc1)c1ccccc1Cl	SmoAnta	-1.38	0.447
142	CHEMBL2031287	COc1cc(C(=O)N=C(N)Nc2cccc(NC(=O)c3ccc(-c4ccc(F)cc4)cc3)c2)cc(OC)c1OC	SmoAnta	-0.91	0.203
143	CHEMBL4632598	CN(C(=O)c1ccc(F)cc1C(F)(F)F)C1CCN(C(=O)c2ccccc2Nc2ccccc2)CC1	SmoAnta	-1.62	0.441
144	CHEMBL1813095	O=C(Nc1ccccc1-c1ccccc1)N1CCn2c(c(O)n(C3CC3c3ccccc3)c2=O)C1	SmoAnta	-0.78	0.454
145	CHEMBL523255	CC(=O)NCc1ccc(N2CCN(c3nnc(Cc4ccccc4)c4ccccc34)CC2)nc1	SmoAnta	-1.4	0.482
146	CHEMBL2043434	COc1ccc(NC(=O)N[C@@H]2CCN(C)C(c3nc4ccccc4[nH]3)C2)cc1	SmoAnta	-1.33	0.648
147	CHEMBL1824920	O=C(Nc1ccc(-c2nccc(-c3ccccc3)n2)cc1)c1ccccc1Cl	SmoAnta	-1.71	0.489
148	CHEMBL485869	CC(O)c1ccc(N2CCN(c3nnc(Cc4ccccc4)c4ccccc34)CC2)nc1	SmoAnta	-1.06	0.522
149	CHEMBL3126707	CS(=O)(=O)c1ccc(C(=O)Nc2ccc(Cl)c(C(=O)Nc3cccc(O)c3)c2)c(Cl)c1	SmoAnta	-1.53	0.495
150	CHEMBL563928	CCCC(CCC)C(=O)NCc1ccc2c(cnn2-c2ccc(OC)cc2)c1	SmoAnta	-1.2	0.577
151	CHEMBL1824910	O=C(Nc1ccc(-c2ncc(-c3ccccc3)o2)cc1)c1ccccc1Cl	SmoAnta	-1.54	0.482
152	CHEMBL2031268	COc1cc(C(=O)NC(=S)Nc2ccc(C)c(NC(=O)c3ccc(-c4ccccc4)cc3)c2)cc(OC)c1OC	SmoAnta	-1.27	0.227
153	CHEMBL1813104	CS(=O)(=O)c1cc(Cl)c(NC(=O)N2CCn3c(c(O)n([C@H]4C[C@@H]4c4ccccc4)c3=O)C2)c(Cl)c1	SmoAnta	-1.13	0.525
154	CHEMBL2059872	Cc1ccc(-c2ncc(CN3CCOCC3)s2)cc1NC(=O)c1ccc(OCc2ccccn2)cc1	SmoAnta	-2.19	0.359
155	CHEMBL3740447	O=C(NC(=O)c1ccc(OCc2ccc(F)cc2)cc1)Nc1ccc(Cl)c(-c2nc3ccccc3[nH]2)c1	SmoAnta	-1.7	0.239

156	CHEMBL4444651	Cc1ccc(OCc2ccnn2-c2nnc(N3CCC(C(=O)NC4CCCCC4C)CC3)s2)nc1	SmoAnta	-1.92	0.53
157	CHEMBL2043432	CN1CC[C@@H](NC(=O)c2ccc3c(c2)OCCO3)CC1c1nc2ccccc2[nH]1	SmoAnta	-1.1	0.717
158	CHEMBL1824911	Cc1oc(-c2ccc(NC(=O)c3ccccc3Cl)cc2)nc1-c1ccccc1	SmoAnta	-1.56	0.451
159	CHEMBL2059873	Cc1ccc(-c2nccs2)cc1NC(=O)c1ccc(OCc2ccccn2)cc1	SmoAnta	-2.12	0.473
160	CHEMBL2043433	CN1CC[C@@H](NC(=O)Nc2ccc3c(c2)OCCO3)CC1c1nc2ccccc2[nH]1	SmoAnta	-1.41	0.62
161	CHEMBL495875	NC(=O)c1ccc(N2CCN(c3nnc(Cc4ccccc4)c4ccccc34)CC2)nc1	SmoAnta	-1.38	0.53
162	CHEMBL3960082	Cc1ccc2c(n1)C(C(C)C(=O)Nc1ccc(Cl)c(-c3nc4ccccc4[nH]3)c1)CC[C@H]2C	SmoAnta	-0.78	0.351
163	CHEMBL4167751	Cc1ccc(NCc2cccn2-c2nnc(N3CCC(C(=O)NC4CCCCC4C)CC3)s2)cc1	SmoAnta	-1.78	0.476
164	CHEMBL1824921	O=C(Nc1ccc(-c2cc(-c3ccccc3)ncn2)cc1)c1ccccc1Cl	SmoAnta	-1.44	0.489
165	CHEMBL1824906	O=C(Nc1ccc(C=Cc2ccccc2)cc1)c1ccccc1Cl	SmoAnta	-1.1	0.604
166	CHEMBL4558043	Cc1ccc(NCc2ccnn2-c2nnc(N3CCC(C(=O)NC4CCCCC4C)CC3)s2)cn1	SmoAnta	-1.92	0.513
167	CHEMBL3978393	Cc1ccc2c(n1)C(C(C)C(=O)Nc1ccc(Cl)c(-c3nc4ccc(OC(F)(F)F)cc4n3C)c1)CC[C@H]2C	SmoAnta	-0.81	0.273
168	CHEMBL521900	N#Cc1ccc(N2CCN(c3nnc(Cc4cccc(Cl)c4)c4ccccc34)CC2)nc1	SmoAnta	-1.87	0.466
169	CHEMBL451194	Cc1cccc(C(=O)Nc2ccc3c(c2)C[C@@H](NC(c2ccccc2)C(F)(F)F)C3)c1-c1ccc(C(F)(F)F)cc1	SmoAnta	-0.9	0.23
170	CHEMBL3819559	Cn1c(-c2cc(N3CCc4sc(C(=O)Nc5cccnc5)cc4C3)ccc2Cl)nc2ccccc21	SmoAnta	-1.91	0.321
171	CHEMBL1209250	C[C@@H]1CN(C(=O)c2ccccc2)CCN1c1nnc(-c2ccccn2)c2ccccc12	SmoAnta	-1.34	0.511
172	CHEMBL4437383	Cc1ccc(CCc2ccnn2-c2nnc(N3CCC(C(=O)NC4CCCCC4C)CC3)s2)nc1	SmoAnta	-1.67	0.533
173	CHEMBL142972	Cc1cccc(C(=O)Nc2ccc3c(c2)C[C@@H](NCc2ccccn2)C3)c1-c1ccc(C(F)(F)F)cc1	SmoAnta	-1.31	0.314
174	CHEMBL4160906	Cc1ccc(=NCc2cccn2-c2nnc(N3CCC(C(=O)NC4CCCCC4C)CC3)s2)[nH]c1	SmoAnta	-1.53	0.543
175	CHEMBL4441183	Cc1c(O)n(-c2ccc(N=[N+]=[N-])cc2)c(=O)n1Cc1ccccc1	SmoAnta	-0.99	0.451
176	CHEMBL497436	C[C@H]1CN(c2nnc(Cc3ccccc3)c3ccccc23)CCN1c1ccc(C#N)cn1	SmoAnta	-1.53	0.493
177	CHEMBL4586057	Cc1c(O)n(-c2ccc(Cl)cc2)c(=O)n1Cc1ccccc1	SmoAnta	-1.26	0.806
178	CHEMBL2057337	Cc1ccc(-c2ncnc3[nH]cnc23)cc1NC(=O)c1ccc(OCc2ccccn2)cc1	SmoAnta	-1.53	0.404
179	CHEMBL1824912	O=C(Nc1ccc(-c2nc(-c3ccccc3)co2)cc1)c1ccccc1Cl	SmoAnta	-1.53	0.482
180	CHEMBL254129	CC1=C2C[C@H]3[C@@H](CC=C4C[C@@H](O)CC[C@@]43C)[C@@H]2CC[C@]12O[C@@H]1C[C@H](C)CN[C@H]1[C@H]2C	SmoAnta	2.99	0.551
181	CHEMBL1209379	C[C@@H]1CN(C(=O)c2ccccc2)CCN1c1nnc(-c2ccccc2)c2ncccc12	SmoAnta	-1.25	0.511
182	CHEMBL2031289	COc1cc(C(=O)N=C(N)Nc2ccc(Cl)c(NC(=O)c3ccc(-c4ccccc4)cc3)c2)cc(OC)c1OC	SmoAnta	-0.99	0.183
183	CHEMBL562270	CCCC(CCC)C(=O)NCc1ccc2c(cnn2-c2cccc(C)c2)c1	SmoAnta	-1.5	0.6
184	CHEMBL2031283	COc1cc(C(=O)N=C(N)Nc2cccc(-c3cc4ccccc4[nH]3)c2)cc(OC)c1OC	SmoAnta	-0.78	0.3
185	CHEMBL497534	N#Cc1ccc(N2CCN(c3nnc(Cc4ccccc4)c4ccccc34)CC2)nc1	SmoAnta	-1.62	0.513
186	CHEMBL496603	N#Cc1ccc(N2CCN(c3nnc(Cc4ccc(F)cc4)c4ccccc34)CC2)nc1	SmoAnta	-1.83	0.494
187	CHEMBL4593367	CC1CCCCC1NC(=O)C1CCN(c2nnc(-n3nccc3CNc3cccnc3)s2)CC1	SmoAnta	-1.87	0.532
188	CHEMBL4282915	CN(Cc1ccc(F)cc1C(F)(F)F)C1CCN(c2nnc(-c3ccnn3C)c3ccccc23)CC1	SmoAnta	-1.77	0.35
189	CHEMBL3972086	Cc1cc(-c2ncc(CNC(=O)c3ccc4c(c3)[nH]c3ccccc34)cc2F)ccn1	SmoAnta	-1.04	0.43
190	CHEMBL2043438	CN1CC[C@@H](NC(=O)Nc2cccc(C#N)c2)CC1c1nc2ccccc2[nH]1	SmoAnta	-1.79	0.655
191	CHEMBL521558	FC(F)(F)c1ccc(N2CCN(c3nnc(Cc4ccccc4)c4ccccc34)CC2)nc1	SmoAnta	-1.52	0.441
192	CHEMBL1813103	CN(C)C(=O)c1cc(Cl)c(NC(=O)N2CCn3c(c(O)n([C@H]4C[C@@H]4c4ccccc4)c3=O)C2)c(Cl)c1	SmoAnta	-1.15	0.53
193	CHEMBL2031077	COc1cc(C(=O)NC(=O)Nc2ccc(Cl)c(NC(=O)c3ccc(-c4ccccc4)cc3)c2)cc(OC)c1OC	SmoAnta	-1.18	0.235
194	CHEMBL1209249	C[C@@H]1CN(C(=O)c2ccccc2)CCN1c1nnc(-c2cccnc2)c2ccccc12	SmoAnta	-1.41	0.511
195	CHEMBL1824907	O=C(Cc1ccc(NC(=O)c2ccccc2Cl)cc1)c1ccccc1	SmoAnta	-1.25	0.657
196	CHEMBL474302	Cc1cccc(C(=O)Nc2ccc3c(c2)C[C@@H](Nc2ccccc2)C3)c1-c1ccc(C(F)(F)F)cc1	SmoAnta	-1	0.306
197	CHEMBL4447291	Cc1ccc(=NCc2ccnn2-c2nnc(N3CCC(C(=O)NC4CCCCC4C)CC3)s2)[nH]n1	SmoAnta	-1.69	0.543
198	CHEMBL497639	C[C@@H]1CN(c2ccc(C#N)cn2)CCN1c1nnc(Cc2ccccc2)c2ccccc12	SmoAnta	-1.58	0.493
199	CHEMBL3979327	Cc1ccc2c(n1)C(C(C)C(=O)Nc1ccc(Cl)c(-c3nc4cc(OC(F)(F)F)ccc4n3C)c1)CC[C@H]2C	SmoAnta	-0.87	0.273
200	CHEMBL3262641	Cc1cnc(-c2cc(N3CCn4cc(C(=O)Nc5cccc(F)c5)nc4C3)ncc2Cl)c(C)c1	SmoAnta	-1.85	0.447
201	CHEMBL2043431	O=C(N[C@@H]1CCCC(c2nc3ccccc3[nH]2)C1)c1ccc2c(c1)OCCO2	SmoAnta	-1.09	0.728
202	CHEMBL2031098	COc1cc(C(=O)NC(=S)Nc2cccc(NC(=O)c3ccc(-c4ccccc4)cc3)c2)cc(OC)c1OC	SmoAnta	-1.09	0.244
203	CHEMBL1824913	O=C(Nc1ccc(-c2nnc(-c3ccccc3)o2)cc1)c1ccccc1Cl	SmoAnta	-1.5	0.525
204	CHEMBL495876	NS(=O)(=O)c1ccc(N2CCN(c3nnc(Cc4ccccc4)c4ccccc34)CC2)nc1	SmoAnta	-1.54	0.488
205	CHEMBL4450586	CC(=O)c1cccc(C(=O)Nc2ccc(N3CCN(c4ccc(OC[C@@H]5CO[C@](C)(c6ccc(Cl)cc6Cl)O5)cc4)CC3)cc2)c1	SmoAnta	-0.75	0.187
206	CHEMBL1082790	CC(=O)N1CCN(c2nnc(-c3ccccc3)c3ccccc23)CC1	SmoAnta	-1.31	0.724
207	CHEMBL3818034	Cn1c(-c2cc(N3CCc4sc(C(=O)N5CCN(c6ncccn6)CC5)cc4C3)ccc2Cl)nc2ccccc21	SmoAnta	-1.8	0.294

208	CHEMBL4441914	Cc1c(O)n(-c2ccc(Cl)cc2)c(=O)n1CCc1ccccc1	SmoAnta	-1.21	0.796
209	CHEMBL1824922	O=C(Nc1ccc(-c2ccnc(-c3ccccc3)c2)cc1)c1ccccc1Cl	SmoAnta	-1.44	0.447
210	CHEMBL1824914	O=C(Nc1ccc(-c2coc(-c3ccccc3)n2)cc1)c1ccccc1Cl	SmoAnta	-1.53	0.482
211	CHEMBL471881	COc1ccc(CN[C@H]2Cc3ccc(NC(=O)c4cccc(C)c4-c4ccc(C(F)(F)F)cc4)cc3C2)cc1	SmoAnta	-0.94	0.266
212	CHEMBL1082967	C[C@H]1CN(c2nnc(-c3ccncc3)c3ccccc23)CCN1C(=O)c1ccccc1	SmoAnta	-1.36	0.511
213	CHEMBL3949191	Cc1c(Cc2ccccc2)nnc(N2CCC(O)(c3ccc(C(C)(C)O)cn3)CC2)c1C	SmoAnta	-0.52	0.638
214	CHEMBL3966039	Cc1c(-c2ccc(C(F)(F)F)cc2)nnc(N2CCN(c3cnc(C(=O)O)cn3)[C@H](C)C2)c1C	SmoAnta	-1.27	0.609
215	CHEMBL3971008	Cc1c(Cc2ccccc2)nnc(N2CCN(c3cnc(C(O)CO)cn3)[C@H](C)C2)c1C	SmoAnta	-0.64	0.609
216	CHEMBL3965479	Cc1c(Cc2ccccc2)nnc(N2CCN(c3cnc(C(=O)N4CCNC(=O)C4)cn3)[C@H](C)C2)c1 C	SmoAnta	-1.34	0.565
217	CHEMBL3982279	Cc1c(Cc2ccccc2)nnc(N2CCN(C(=O)Nc3ccccc3)CC2)c1C	SmoAnta	-1.31	0.717
218	CHEMBL3953149	Cc1c(C(=O)c2ccccc2)nnc(N2CCN(c3cnc(C(C)(C)O)cn3)[C@H](C)C2)c1C	SmoAnta	-0.91	0.598
219	CHEMBL3965793	COC(=O)c1cnc(N2CCN(c3nnc(Cc4ccncc4)c(C)c3C)C[C@H]2C)cn1	SmoAnta	-1.12	0.562
220	CHEMBL3978301	CC(=O)N1CCN(C(=O)c2cnc(N3CCN(c4nnc(- c5ccc(C(F)(F)F)cc5)c(C)c4C)C[C@H]3C)cn2)CC1	SmoAnta	-1.46	0.461
221	CHEMBL3948598	Cc1c(Cc2ccccc2)nnc(N2CCN(c3ccc(C(F)(F)F)cn3)CC2)c1C	SmoAnta	-1.51	0.617
222	CHEMBL1813102	N#Cc1cc(Cl)c(NC(=O)N2CCn3c(c(O)n([C@H]4C[C@@H]4c4ccccc4)c3=O)C2)c(Cl)c1	SmoAnta	-1.15	0.578
223	CHEMBL3959511	CCCCOC(=O)c1cnc(N2CCN(c3nnc(-c4ccc(C(C)C)cc4)c(C)c3C)CC2)cc1C(F)(F)F	SmoAnta	-1.19	0.23
224	CHEMBL3919707	Cc1c(Cc2ccccc2)nnc(N2CCN(c3cnc(C(=O)N4C[C@H](C)O[C@H](C)C4)cn3)[C@ H](C)C2)c1C	SmoAnta	-1.3	0.511
225	CHEMBL3934557	Cc1c(Cc2ccccc2)nnc(N2CCN(c3cnc(C(=O)N(C)CCO)cn3)[C@H](C)C2)c1C	SmoAnta	-1.3	0.556
226	CHEMBL3891390	Cc1c(Cc2ccccc2)nnc(N2CCN(c3cnc(C(=O)NCCCO)cn3)[C@H](C)C2)c1C	SmoAnta	-1.12	0.478
227	CHEMBL2031245	COc1cc(C(=O)NC(=S)Nc2cccc(NC(=O)c3ccc(-c4ccc(F)cc4)cc3)c2)cc(OC)c1OC	SmoAnta	-1.26	0.231
228	CHEMBL561735	CCCC(CCC)C(=O)NCc1ccc2c(cnn2-c2cccc(OC)c2)c1	SmoAnta	-1.38	0.577
229	CHEMBL3941852	Cc1c(Cc2ccccc2)nnc(N2CCN(c3cnc(C(=O)N4CCN(C(C)C)CC4)cn3)[C@H](C)C2) c1C	SmoAnta	-1.4	0.482
230	CHEMBL3891481	Cc1c(Cc2ccccc2)nnc(N2CCN(c3cnc(C(=O)NCC(O)CO)cn3)[C@H](C)C2)c1C	SmoAnta	-1	0.427
231	CHEMBL3915100	Cc1c(Cc2ccccc2)nnc(N2CCN(c3cnc(C(=O)NC4CCC(O)CC4)cn3)[C@H](C)C2)c1C	SmoAnta	-1.08	0.515
232	CHEMBL3126705	Cc1cccc(NC(=O)c2cc(NC(=O)c3ccc(S(C)(=O)=O)cc3Cl)ccc2Cl)c1C	SmoAnta	-2.02	0.492
233	CHEMBL3986419	COC(=O)c1cnc(N2CCN(c3n[nH]c(=Nc4ccccc4)c(C)c3C)C[C@H]2C)cn1	SmoAnta	-1.16	0.631
234	CHEMBL3927351	Cc1c(-c2ccc(F)c(C#N)c2)nnc(N2CCN(c3ccc(C(F)(F)F)cn3)CC2)c1C	SmoAnta	-2.01	0.54
235	CHEMBL3894764	Cc1c(Cc2ccccc2)nnc(N2CCN(C(=O)Oc3ccccc3)CC2)c1C	SmoAnta	-0.94	0.66
236	CHEMBL3957159	CCCCOC(=O)c1cnc(N2CCN(c3nnc(-c4ccc(F)nc4)c(C)c3C)C[C@H]2C)cc1C(F)(F)F	SmoAnta	-1.24	0.169
237	CHEMBL3965473	Fc1cccc(Cc2nnc(N3CCN(c4ccc(C(F)(F)F)cn4)CC3)c3c2CCC3)c1	SmoAnta	-1.78	0.543
238	CHEMBL3897743	Cc1c(Cc2ccccc2)nnc(N2CCN(c3cnc(C(=O)O)cn3)[C@H](C)C2)c1C	SmoAnta	-0.99	0.676
239	CHEMBL3929514	Cc1c(Cc2ccccc2)nnc(N2CCN(c3cnc(C(=O)N4CCC(CCO)CC4)cn3)[C@H](C)C2)c1 C	SmoAnta	-1.03	0.497
240	CHEMBL3967092	Cc1c(Cc2ccccc2)nnc(N2CCN(c3cnc(C(=O)NCCc4ccccn4)cn3)[C@H](C)C2)c1C	SmoAnta	-1.43	0.375
241	CHEMBL3979837	Cc1c(Cc2ccccc2)nnc(N2CCN(C(=O)NCc3ccccc3)CC2)c1C	SmoAnta	-1.24	0.69
242	CHEMBL3959845	CCCCOC(=O)c1cnc(N2CCN(c3nnc(-c4cccnc4)c(C)c3C)C[C@H]2C)cc1C(F)(F)F	SmoAnta	-1.31	0.304
243	CHEMBL3927593	Cc1c(- c2ccc(C(F)(F)F)cc2)nnc(N2CCN(c3cnc(C(=O)N4CCN(S(C)(=O)=O)CC4)cn3)[C@ H](C)C2)c1C	SmoAnta	-1.72	0.426
244	CHEMBL3912445	Cc1c(Cc2ccccc2)nnc(N2CCN(c3cnc(C(=O)NC4CC4)cn3)[C@H](C)C2)c1C	SmoAnta	-1.35	0.609
245	CHEMBL3955346	Cc1ccccc1Cc1nnc(N2CCN(c3ccc(C(F)(F)F)cn3)CC2)c2c1CCC2	SmoAnta	-1.66	0.578
246	CHEMBL3986486	Cc1c(Cc2ccccc2)nnc(N2CCN(c3cnc(C(=O)NCCN4CCOCC4)cn3)[C@H](C)C2)c1C	SmoAnta	-1.53	0.47
247	CHEMBL3977460	COC(=O)c1cnc(N2CCN(c3nnc(-c4ccc(F)c(C#N)c4)c(C)c3C)C[C@H]2C)cn1	SmoAnta	-1.63	0.542
248	CHEMBL3958236	Cc1c(Cc2ccccc2)nnc(N2CCC(c3nc4cccnc4[nH]3)CC2)c1C	SmoAnta	-1.1	0.554
249	CHEMBL3957247	Cc1c(Cc2ccccc2)nnc(N2CCN(c3cnc(C(=O)N4CCN(C)CC4)cn3)[C@H](C)C2)c1C	SmoAnta	-1.32	0.529
250	CHEMBL3948829	Cc1c(Cc2ccc(F)cc2)nnc(N2CCN(c3ccc(C(C)(C)C)cc3)CC2)c1C	SmoAnta	-1.24	0.549
251	CHEMBL3926840	Cc1c(Cc2ccccc2)nnc(N2CCN(c3cnc(C(=O)NCC(C)O)cn3)[C@H](C)C2)c1C	SmoAnta	-1.1	0.536
252	CHEMBL3973612	FC(F)(F)c1ccc(N2CCN(c3nnc(Cc4ccccc4Cl)c4c3CCC4)CC2)nc1	SmoAnta	-1.83	0.536
253	CHEMBL3909290	Cc1c(N2CCN(c3cnc(C(=O)Nc4ccccc4)cn3)[C@H](C)C2)n[nH]c(=Nc2ccccc2)c1C	SmoAnta	-1.43	0.435
254	CHEMBL3958040	Cc1c(Cc2ccccc2)nnc(C2CCN(c3ccc(C(F)(F)F)cn3)CC2)c1C	SmoAnta	-1.41	0.554
255	CHEMBL3930632	CC(C)(C)c1ccc(N2CCN(c3nnc(Cc4ccc(F)cc4)c4c3CCCC4)CC2)cc1	SmoAnta	-1.24	0.501
256	CHEMBL3906764	COC(=O)c1cnc(N2CCC(c3nnc(Cc4ccccc4)c(C)c3C)CC2)cn1	SmoAnta	-1.02	0.586
257	CHEMBL3905798	Cc1c(Cc2ccccc2)nnc(N2CCN(c3cnc(C(=O)NCCN4CCCC4)cn3)[C@H](C)C2)c1C	SmoAnta	-1.44	0.491

258	CHEMBL3980924	Fc1ccc(Cc2nnc(N3CCN(c4ccc(C(F)(F)F)cn4)CC3)c3c2CCCC3)cc1	SmoAnta	-1.62	0.511
259	CHEMBL3930063	Cc1c(-c2ccncc2)nnc(N2CCN(c3ccc(C(F)(F)F)cn3)CC2)c1C	SmoAnta	-1.75	0.648
260	CHEMBL3952417	CC(=O)c1cnc(N2CCN(c3nnc(Cc4ccccc4)c(C)c3C)C[C@H]2C)nc1C(F)(F)F	SmoAnta	-1.13	0.497
261	CHEMBL3942299	Cc1c(Cc2ccc(F)cc2)nnc(N2CCN(c3ccc(C(F)(F)F)cn3)CC2)c1C	SmoAnta	-1.65	0.551
262	CHEMBL3942727	Cc1c(-c2ccc(F)cc2)nnc(N2CCN(c3ccc(C(F)(F)F)cn3)CC2)c1C	SmoAnta	-1.81	0.566
263	CHEMBL3950710	COC(=O)c1cnc(N2CCN(c3nnc(-c4ccc(OC)cc4)c(C)c3C)CC2)nc1C(F)(F)F	SmoAnta	-1.25	0.484
264	CHEMBL3944996	Fc1ccc(Cc2nnc(N3CCN(c4ccc(C(F)(F)F)cn4)CC3)c3c2CCC3)cc1	SmoAnta	-1.67	0.543
265	CHEMBL3905821	CCCCOC(=O)c1cnc(N2CCN(c3nnc(-c4ccncc4)c(C)c3C)C[C@H]2C)cc1C(F)(F)F	SmoAnta	-1.24	0.304
266	CHEMBL3923355	CCCCOC(=O)c1cnc(N2CCN(c3nnc(-c4cncc(OC)c4)c(C)c3C)CC2)cc1C(F)(F)F	SmoAnta	-1.21	0.291
267	CHEMBL3922446	CC[C@@H]1CN(c2nnc(Cc3ccccc3)c(C)c2C)CCN1c1cnc(C(C)=O)cn1	SmoAnta	-1.01	0.551
268	CHEMBL3927084	Cc1c(Cc2ccc(F)cc2F)nnc(N2CCN(c3ncc(C(C)(C)O)c(C(F)(F)F)n3)[C@H](C)C2)c1 C	SmoAnta	-1.17	0.473
269	CHEMBL3911804	Cc1c(Cc2ccccc2)nnc(C2CCN(c3cnc(C(C)(C)O)cn3)CC2)c1C	SmoAnta	-0.82	0.674
270	CHEMBL3604621	CC(=O)N=c1[nH]c2ccc(-c3cnc(Cl)c(NC(C#N)c4ccccc4)c3)cc2s1	SmoAnta	-1.6	0.439
271	CHEMBL3946402	Cc1c(Cc2ccccc2)nnc(N2CCN(c3ccc(S(=O)(=O)C(F)(F)F)cc3)[C@H](C)C2)c1C	SmoAnta	-1.2	0.502
272	CHEMBL3966631	CCN(CCO)C(=O)c1cnc(N2CCN(c3nnc(Cc4ccccc4)c(C)c3C)C[C@H]2C)cn1	SmoAnta	-1.38	0.516
273	CHEMBL3950687	Cc1c(Cc2ccccc2)nnc(N2CCN(c3cnc(C(=O)NCC(F)(F)F)cn3)[C@H](C)C2)c1C	SmoAnta	-1.48	0.556
274	CHEMBL3903125	Cc1c(Cc2ccccc2)nnc(N2CCN(c3ncc(C(=O)N(C)CCO)c(C(F)(F)F)n3)[C@H](C)C2) c1C	SmoAnta	-1.33	0.485
275	CHEMBL3938550	Cc1c(Cc2ccccc2)nnc(N2CCN(c3cncc(C(N)=O)n3)[C@H](C)C2)c1C	SmoAnta	-1.11	0.68
276	CHEMBL3933708	CCOC(=O)c1ccc(N2CCN(c3nnc(Cc4ccc(F)cc4)c4c3CCCC4)CC2)nc1	SmoAnta	-1.41	0.5
277	CHEMBL3915663	COC(=O)c1cnc(N2CCN(c3nnc(-c4ccc(F)c(Cl)c4)c(C)c3C)CC2)cc1C(F)(F)F	SmoAnta	-1.64	0.349
278	CHEMBL3889599	Cc1c(- c2ccc(C(F)(F)F)cc2)nnc(N2CCN(c3cnc(C(=O)N(C)CCc4ccccc4)cn3)[C@H](C)C2) c1C	SmoAnta	-1.43	0.278
279	CHEMBL3903694	COC(=O)c1cnc(N2CCN(c3nnc(C(=O)c4ccccc4)c(C)c3C)C[C@H]2C)cn1	SmoAnta	-1.09	0.432
280	CHEMBL3953389	COC(=O)c1cnc(N2CCN(c3nnc(-c4cccc(Cl)c4)c(C)c3C)CC2)nc1C(F)(F)F	SmoAnta	-1.54	0.483
281	CHEMBL3971766	COC(=O)c1cnc(N2CCN(c3nnc(Cc4ccccc4)c(C)c3C)C[C@H]2C)cn1	SmoAnta	-1.07	0.569
282	CHEMBL3983876	COC(=O)c1cnc(N2CCN(c3nnc(Cc4ccc(F)cc4)c(C)c3C)C[C@H]2C)nc1C(F)(F)F	SmoAnta	-1.25	0.368
283	CHEMBL3955058	CC(=O)c1cnc(N2CCN(c3nnc(Cc4ccc(F)cc4)c(C)c3C)C[C@H]2C)nc1C(F)(F)F	SmoAnta	-1.26	0.374
284	CHEMBL3931348	Cc1c(Cc2ccccc2)nnc(N2CCN(c3cnc(C(C)O)cn3)[C@H](C)C2)c1C	SmoAnta	-0.83	0.681
285	CHEMBL3922623	Cc1c(Cc2ccccc2)nnc(N2CCN(c3cnc(C(=O)NC(C)(C)CO)cn3)[C@H](C)C2)c1C	SmoAnta	-1.17	0.522
286	CHEMBL3910022	Fc1ccc(Cc2nnc(N3CCN(c4ccc(C(F)(F)F)cn4)CC3)c3c2CCC3)c(F)c1	SmoAnta	-1.85	0.518
287	CHEMBL3906492	Cc1c(N2CCN(c3cnc(C(C)(C)O)cn3)[C@H](C)C2)n[nH]c(=Nc2ccccc2)c1C	SmoAnta	-0.97	0.657
288	CHEMBL3986550	Cc1c(Cc2ccccc2)nnc(N2CCN(c3cnc(C(=O)N4CCOCC4)cn3)[C@H](C)C2)c1C	SmoAnta	-1.43	0.543
289	CHEMBL3937323	Cc1c(Cc2ccccc2)nnc(N2CCN(c3cnc(C(=O)NCC(C)C)cn3)[C@H](C)C2)c1C	SmoAnta	-1.19	0.561
290	CHEMBL3921763	CCOC(=O)c1cnc(N2CCN(c3nnc(Cc4ccccc4)c(C)c3C)C[C@H]2C)nc1C(F)(F)F	SmoAnta	-1.17	0.448
291	CHEMBL3899187	FC(F)(F)c1ccc(N2CCN(c3nnc(Cc4ccccc4)c4c3CCC4)CC2)nc1	SmoAnta	-1.55	0.607
292	CHEMBL3896503	Cc1c(Cc2ccccc2)nnc(N2CCC(c3nc(Cl)c[nH]3)CC2)c1C	SmoAnta	-0.93	0.725
293	CHEMBL3934007	Cc1nc(Cc2nnc(N3CCN(c4ccc(C(F)(F)F)cn4)CC3)c(C)c2C)no1	SmoAnta	-2.02	0.621
294	CHEMBL3892588	Cc1c(Cc2ccccc2)nnc(N2CCC(C#N)(c3ccc(C(C)(C)O)cn3)CC2)c1C	SmoAnta	-0.65	0.633
295	CHEMBL3967151	Cc1nnc(Cc2nnc(N3CCN(c4ccc(C(F)(F)F)cn4)CC3)c(C)c2C)o1	SmoAnta	-1.82	0.621
296	CHEMBL3889851	Cc1c(Cc2ccccc2)nnc(N2CCC(c3nc(C(F)(F)F)c[nH]3)CC2)c1C	SmoAnta	-0.99	0.664
297	CHEMBL3902706	CC(=O)c1cnc(N2CCN(c3nnc(Cc4ccccc4)c4c3CCC4)C[C@H]2C)nc1C(F)(F)F	SmoAnta	-1.17	0.488
298	CHEMBL3939322	Cc1c(- c2ccc(C(F)(F)F)cc2)nnc(N2CCN(c3cnc(C(=O)N4CCC5(CC4)OCCO5)cn3)[C@H](C)C2)c1C	SmoAnta	-1.44	0.437
299	CHEMBL3906243	Cc1c(Cc2ccccc2)nnc(N2CCC(N3Cc4ccccc4C3)CC2)c1C	SmoAnta	-0.69	0.639
300	CHEMBL3944267	Cc1c(Cc2ccccn2)nnc(N2CCN(c3cnc(C(C)(C)O)cn3)[C@H](C)C2)c1C	SmoAnta	-1.14	0.657
301	CHEMBL3961874	Cc1c(Cc2ccccc2)nnc(N2CCC(F)(c3ccc(C(C)(C)O)cc3)CC2)c1C	SmoAnta	-0.33	0.587
302	CHEMBL3925179	CC(=O)c1cnc(N2CCN(c3nnc(Cc4ccccc4)c(C)c3C)C[C@H]2C)cn1	SmoAnta	-1.08	0.59
303	CHEMBL3967114	Cc1c(-c2cccc(Cl)c2)nnc(N2CCN(c3ccc(C(F)(F)F)cn3)CC2)c1C	SmoAnta	-1.92	0.555
304	CHEMBL3983242	COC(=O)c1ncc(N2CCN(c3nnc(Cc4ccccc4)c4c3CCC4)C[C@H]2C)nc1C(F)(F)F	SmoAnta	-1.07	0.477
305	CHEMBL3895940	COC(=O)c1cnc(N2CCN(c3nnc(Cc4ccccn4)c(C)c3C)C[C@H]2C)cn1	SmoAnta	-1.34	0.562
306	CHEMBL3951798	Cc1c(Cc2ccccc2)nnc(N2CCC(N3CCc4ccccc4C3)CC2)c1C	SmoAnta	-0.64	0.621
307	CHEMBL3969729	Cc1c(-c2ccc(C(C)C)cc2)nnc(N2CCN(c3ccc(C(F)(F)F)cn3)CC2)c1C	SmoAnta	-1.57	0.511
308	CHEMBL3898393	CCCCOC(=O)c1cnc(N2CCN(c3nnc(-c4ccc(F)c(C#N)c4)c(C)c3C)CC2)cc1C(F)(F)F	SmoAnta	-1.55	0.215
309	CHEMBL3974732	COc1ccc(-c2nnc(N3CCN(c4ncc(C(C)(C)O)c(C(F)(F)F)n4)CC3)c(C)c2C)cc1	SmoAnta	-0.97	0.557

310	CHEMBL3934045	Cc1c(Cc2ccccc2)nnc(N2CCN(c3cnc(C(=O)CO)cn3)[C@H](C)C2)c1C	SmoAnta	-0.85	0.593
311	CHEMBL3938334	Cc1c(Cc2ccccc2)nnc(N2CCN(c3cnc(C(=O)NCCN4CCN(C)CC4)cn3)[C@H](C)C2)c1C	SmoAnta	-1.45	0.459
312	CHEMBL3944445	COC(=O)c1cnc(N2CCN(c3nnc(Cc4ccccc4)c(C)c3C)C[C@@H]2C)cn1	SmoAnta	-1.07	0.569
313	CHEMBL2043429	CN1CC[C@@H](NC(=O)Nc2ccccc2)CC1c1nc2ccccc2[nH]1	SmoAnta	-1.38	0.677
314	CHEMBL2031273	COc1cc(C(=O)NC(=O)Nc2cccc(NC(=O)c3ccccc3)c2)cc(OC)c1OC	SmoAnta	-1.03	0.504
315	CHEMBL2031291	COc1cc(C(=O)N=C(N)Nc2ccc(C)c(NC(=O)c3cccc(-c4ccccc4)c3)c2)cc(OC)c1OC	SmoAnta	-0.99	0.2
316	CHEMBL1580265	O=C(Nc1ccc(-c2csc(-c3ccccc3)n2)cc1)c1ccccc1Cl	SmoAnta	-1.96	0.439
317	CHEMBL1082713	C[C@H]1CN(c2nnc(-c3ccccn3)c3ccccc23)CCN1C(=O)c1ccccc1	SmoAnta	-1.37	0.511
318	CHEMBL2031281	COc1cc(C(=O)NC(=O)Nc2cccc(NC(=O)c3cc4ccccc4[nH]3)c2)cc(OC)c1OC	SmoAnta	-1.07	0.305
319	CHEMBL474281	Cc1cccc(C(=O)Nc2ccc3c(c2)CC(NCc2ccccn2)C3)c1-c1ccc(C(F)(F)F)cc1	SmoAnta	-1.31	0.314
320	CHEMBL4440172	C[C@@]1(c2ccc(Cl)cc2Cl)OC[C@@H](COc2ccc(N3CCN(c4ccc(NC(=O)NN)cc4)CC3)cc2)O1	SmoAnta	-0.8	0.209
321	CHEMBL551065	CCCC(CCC)C(=O)NCc1ccc2c(cnn2-c2cccc(C)c2C)c1	SmoAnta	-1.32	0.565
322	CHEMBL1615189	CC(=O)N=c1[nH]c2ccc(-c3cnc(Cl)c(NS(=O)(=O)c4ccc(F)cc4)c3)cc2s1	SmoAnta	-1.99	0.428
323	CHEMBL474508	Cc1cccc(C(=O)Nc2ccc3c(c2)C[C@@H](NCc2scnc2C)C3)c1-c1ccc(C(F)(F)F)cc1	SmoAnta	-1.33	0.289
324	CHEMBL4644288	CN(C(=O)c1ccc(F)cc1C(F)(F)F)C1CCN(C(=O)c2ccccc2N=c2cc[nH]n2C)CC1	SmoAnta	-1.47	0.544
325	CHEMBL550190	CCCC(CCC)C(=O)NCc1ccc2c(cnn2-c2ccc(OC)nc2)c1	SmoAnta	-1.52	0.602
326	CHEMBL2031267	COc1cc(C(=O)NC(=S)Nc2ccc(Cl)c(NC(=O)c3ccc(-c4ccccc4)cc3)c2)cc(OC)c1OC	SmoAnta	-1.34	0.209
327	CHEMBL561069	CCCC(CCC)C(=O)NCc1ccc2c(cnn2-c2ccc(F)cc2)c1	SmoAnta	-1.52	0.609
328	CHEMBL3819191	CN1CCN(C(=O)c2cc3c(s2)CCN(c2ccc(Cl)c(-c4nc5ccccc5n4C)c2)C3)CC1	SmoAnta	-1.68	0.397
329	CHEMBL497412	FC(F)(F)c1ccc(N2CCN(c3nnc(Cc4ccccc4)c4occc34)CC2)nc1	SmoAnta	-1.31	0.465
330	CHEMBL480889	COc1cccc(CN(C(=O)CC(C)(C)C)[C@H]2CC(C(=O)N3CCNCC3)N(Cc3ccc4c(c3)OCO4)C2)c1	SmoAnta	-1.05	0.54
331	CHEMBL4588930	Cc1ccc(=NCc2ccnn2-c2nnc(N3CCC(C(=O)Nc4ccccc4C)CC3)s2)[nH]c1	SmoAnta	-1.87	0.431
332	CHEMBL4562342	C[C@@]1(c2ccc(Cl)cc2Cl)OC[C@@H](COc2ccc(N3CCN(c4ccc(-n5cn[nH]c5=O)cc4)CC3)cc2)O1	SmoAnta	-0.57	0.333
333	CHEMBL557114	CCCC(CCC)C(=O)NCc1ccc2c(cnn2-c2cccc3c2CCCC3)c1	SmoAnta	-1.04	0.521
334	CHEMBL3604624	O=C(Nc1cc(-c2ccc3[nH]c(=NCC4CC4)sc3c2)cnc1Cl)c1ccccc1	SmoAnta	-1.31	0.407
335	CHEMBL3604623	O=C(Nc1cc(-c2ccc3[nH]c(=NC4CC4)sc3c2)cnc1Cl)c1cccc(Cl)c1	SmoAnta	-1.52	0.376
336	CHEMBL495620	N#Cc1ccc(N2CCCN(c3nnc(Cc4ccccc4)c4ccccc34)CC2)nc1	SmoAnta	-1.66	0.493
337	CHEMBL3986017	CC(C(=O)Nc1ccc(Cl)c(-c2ccccn2)c1)C1CC[C@@H](C)c2ccc(CN3CCOCC3)nc21	SmoAnta	-1.15	0.453
338	CHEMBL2031295	COc1cc(C(=O)N=C(N)Nc2ccc(Cl)c(C(=O)Nc3ccccc3)c2)cc(OC)c1OC	SmoAnta	-1.12	0.34
339	CHEMBL4572598	C[C@]1(c2ccc(Cl)cc2Cl)OC[C@@H](COc2ccc(N3CCN(c4ccc(NC(=O)c5cccc(O)c5)cc4)CC3)cc2)O1	SmoAnta	-0.63	0.217
340	CHEMBL497217	COC(=O)c1ccc(Cc2nnc(N3CCN(c4ccc(C#N)cn4)CC3)c3ccccc23)cc1	SmoAnta	-1.48	0.414
341	CHEMBL3604616	CC(=O)N=c1[nH]c2ccc(-c3cnc(Cl)c(NC(=O)c4ccc(F)cc4)c3)cc2s1	SmoAnta	-1.84	0.449
342	CHEMBL1813101	O=C(Nc1c(Cl)cc(F)cc1Cl)N1CCn2c(c(O)n([C@H]3C[C@@H]3c3ccccc3)c2=O)C1	SmoAnta	-1.13	0.58
343	CHEMBL2031253	COc1cc(C(=O)NC(=S)Nc2cccc(-c3cc4ccccc4[nH]3)c2)cc(OC)c1OC	SmoAnta	-1.25	0.351
344	CHEMBL3951963	C[C@@H]1CC[C@H]2[C@@](C)(C(=O)Nc3ccc(Cl)c(-c4ccccn4)c3)OC3O[C@@]4(C)CC[C@@H]1[C@]32OO4	SmoAnta	0.89	0.592
345	CHEMBL4473275	Cc1cnc(=NCc2ccnn2-c2nnc(N3CCC(C(=O)NC4CCCCC4C)CC3)s2)[nH]c1	SmoAnta	-1.54	0.543
346	CHEMBL3933195	C[C@@H]1CC[C@H]2[C@@H](C)[C@@H](CC(=O)Nc3ccc(Cl)c(-c4ccccn4)c3)O[C@@H]3O[C@@]4(C)CC[C@@H]1[C@@]23OO4	SmoAnta	0.82	0.558
347	CHEMBL522241	N#Cc1ccc(N2CCN(c3nnc(Cc4ccc(C(F)(F)F)cc4)c4ccccc34)CC2)nc1	SmoAnta	-1.72	0.422
348	CHEMBL498457	OCc1ccc(N2CCN(c3nnc(Cc4ccccc4)c4ccccc34)CC2)nc1	SmoAnta	-1.04	0.543
349	CHEMBL2031294	COc1cc(C(=O)N=C(N)Nc2ccc(C)c(C(=O)Nc3ccccc3)c2)cc(OC)c1OC	SmoAnta	-1	0.36
350	CHEMBL2031258	COc1cc(C(=O)NC(=S)Nc2ccc(Cl)c(NC(=O)c3ccccc3)c2)cc(OC)c1OC	SmoAnta	-1.5	0.404
351	CHEMBL3819380	Cn1c(-c2cc(N3CCc4sc(C(=O)N5CCC(O)CC5)cc4C3)ccc2Cl)nc2ccccc21	SmoAnta	-1.47	0.419
352	CHEMBL3604625	O=C(Nc1cc(-c2ccc3[nH]c(=NCC4CC4)sc3c2)cnc1Cl)c1cccc(Cl)c1	SmoAnta	-1.5	0.344
353	CHEMBL473897	Cc1cccc(C(=O)Nc2ccc3c(c2)C[C@@H](NCc2ccncc2)C3)c1-c1ccc(C(F)(F)F)cc1	SmoAnta	-1.06	0.314
354	CHEMBL4472335	C[C@]1(c2ccc(Cl)cc2Cl)OC[C@@H](COc2ccc(N3CCN(c4ccc(NC(=O)c5cccnc5)cc4)CC3)cc2)O1	SmoAnta	-0.88	0.237
355	CHEMBL2043439	CN1CC[C@@H](NC(=O)Nc2cccnc2)CC1c1nc2ccccc2[nH]1	SmoAnta	-1.63	0.678
356	CHEMBL4447164	C[C@@]1(c2ccc(Cl)cc2Cl)OC[C@@H](COc2ccc(N3CCN(c4ccc(NC(=O)c5cccc(O)c5)cc4)CC3)cc2)O1	SmoAnta	-0.63	0.217
357	CHEMBL561330	CCCC(CCC)C(=O)NCc1ccc2c(cnn2-c2ccccc2C)c1	SmoAnta	-1.35	0.6
358	CHEMBL3262645	Cc1cnc(-c2cc(N3CCn4cc(C(=O)Nc5cccnc5)nc4C3)ncc2Cl)c(C)c1	SmoAnta	-1.74	0.49

359	CHEMBL564222	CCCC(CCC)C(=O)NCc1ccc2c(cnn2-c2ccccc2)c1	SmoAnta	-1.29	0.631
360	CHEMBL454984	Cc1cccc(C(=O)Nc2ccc3c(c2)C[C@@H](NCc2cnc4ccccc4c2)C3)c1-c1ccc(C(F)(F)F)cc1	SmoAnta	-1.06	0.227
361	CHEMBL3604622	O=C(Nc1cc(-c2ccc3[nH]c(=NC4CC4)sc3c2)cnc1Cl)c1ccccc1	SmoAnta	-1.33	0.442
362	CHEMBL4473575	C[C@]1(c2ccc(Cl)cc2Cl)OC[C@@H](COc2ccc(N3CCN(c4ccc(-n5cn[nH]c5=O)cc4)CC3)cc2)O1	SmoAnta	-0.57	0.333
363	CHEMBL1082386	N#Cc1cccc(-c2nnc(N3CCN(C(=O)c4ccccc4)CC3)c3ccccc23)c1	SmoAnta	-1.62	0.499
364	CHEMBL1084241	C[C@H]1CN(c2nnc(-c3cccnc3)c3ccccc23)CCN1C(=O)c1ccccc1	SmoAnta	-1.45	0.511
365	CHEMBL3817897	Cn1c(-c2cc(N3CCc4sc(C(=O)N5CCN(c6ccc(Cl)c(Cl)c6)CC5)cc4C3)ccc2Cl)nc2ccccc21	SmoAnta	-1.54	0.202
366	CHEMBL3967752	Cc1ccc2c(n1)C(C(C)C(=O)Nc1ccc(Cl)c(-c3ccccn3)c1)CC[C@H]2C	SmoAnta	-0.84	0.535
367	CHEMBL2031284	COc1cc(C(=O)N=C(N)Nc2cccc(NC(=O)c3ccc(N4CCOCC4)cc3)c2)cc(OC)c1OC	SmoAnta	-1.13	0.294
368	CHEMBL3819639	Cn1c(-c2cc(N3CCc4sc(C(=O)N5CCN(S(C)(=O)=O)CC5)cc4C3)ccc2Cl)nc2ccccc21	SmoAnta	-1.89	0.367
369	CHEMBL3968327	Cc1ccc2c(n1)C(C(C)C(=O)Nc1ccc(Cl)c(-c3nc(C4CC4)no3)c1)CC[C@H]2C	SmoAnta	-1.09	0.498
370	CHEMBL4525451	Cc1ccc(=NCc2ccnn2-c2nnc(N3CCC(C(=O)N=c4cc[nH]cc4C)CC3)s2)[nH]c1	SmoAnta	-1.42	0.446
371	CHEMBL1813097	O=C(Nc1cccc(Cl)c1Cl)N1CCn2c(c(O)n([C@H]3C[C@@H]3c3ccccc3)c2=O)C1	SmoAnta	-1.13	0.608
372	CHEMBL4276902	CN(C(=O)c1ccc([N+](=O)[O-])cc1)C1CCN(c2nnc(-c3ccnn3C)c3ccccc23)CC1	SmoAnta	-1.73	0.322
373	CHEMBL3818767	Cn1c(-c2cccc(N3CCc4sc(C(=O)N5CCN(c6ncccn6)CC5)cc4C3)c2)nc2ccccc21	SmoAnta	-1.84	0.335
374	CHEMBL4168846	Cc1ccc(NCc2cccn2-c2nnc(N3CCC(C(=O)NC4CCCC4C)CC3)s2)cn1	SmoAnta	-1.94	0.502
375	CHEMBL3924243	C[C@@H]1CC[C@H]2[C@@H](C)[C@@H](CC(=O)Nc3ccc(Cl)c(-c4ccccn4)c3)O[C@@H]3O[C@@]4(C)CC[C@@H]1[C@@]23OO4	SmoAnta	0.99	0.507
376	CHEMBL3980443	Cc1ccc2c(n1)C(C(C)C(=O)Nc1ccc(Cl)c(-c3nc4ccccc4o3)c1)CC[C@H]2C	SmoAnta	-0.9	0.353
377	CHEMBL496014	c1ccc(Cc2nnc(N3CCN(c4ccccn4)CC3)c3ccccc23)cc1	SmoAnta	-1.35	0.536
378	CHEMBL3818148	Cn1c(-c2cccc(N3CCc4sc(C(=O)Nc5cccnc5)cc4C3)c2)nc2ccccc21	SmoAnta	-1.96	0.38
379	CHEMBL3941556	Cc1ccc2c(n1)C(C(C)C(=O)Nc1ccc(Cl)c(-c3nnc(-c4ccnn4C)s3)c1)CC[C@H]2C	SmoAnta	-1.08	0.347
380	CHEMBL4562519	C[C@]1(c2ccc(Cl)cc2Cl)OC[C@@H](COc2ccc(N3CCN(c4ccc([N+](=O)[O-])cc4)CC3)cc2)O1	SmoAnta	-0.68	0.271
381	CHEMBL3262644	Cc1cnc(-c2cc(N3CCn4cc(C(=O)N=c5cccc[nH]5)nc4C3)ncc2Cl)c(C)c1	SmoAnta	-1.35	0.504
382	CHEMBL3262647	Cc1cnc(-c2cc(N3CCn4cc(C(=O)OCC5CC5)nc4C3)ncc2Cl)c(C)c1	SmoAnta	-1.21	0.557
383	CHEMBL3262632	Cc1cnc(-c2cc(N3CCn4cc(C(=O)N5CCCC5)nc4C3)ncc2Cl)c(C)c1	SmoAnta	-1.49	0.623
384	CHEMBL4281406	CN(C(=O)c1ccc(Oc2ccccc2)cc1)C1CCN(c2nnc(-c3ccnn3C)c3ccccc23)CC1	SmoAnta	-1.37	0.293
385	CHEMBL3949509	COC(=O)C(C)c1ccc(C)c2ccc(C(=O)Nc3ccc(Cl)c(-c4ccccn4)c3)nc12	SmoAnta	-1.24	0.382
386	CHEMBL2031084	COc1cc(C(=O)NC(=S)Nc2cccc(NC(=O)c3ccccc3)c2)cc(OC)c1OC	SmoAnta	-1.22	0.452
387	CHEMBL3932585	Cc1ccc2c(n1)C(C(C)C(=O)Nc1ccc(Cl)c(-c3nc4ccccc4s3)c1)CC[C@H]2C	SmoAnta	-1.14	0.331
388	CHEMBL4527095	Cc1ccc(=NCc2ccnn2-c2nnc(N3CCC(C(=O)N=c4[nH]cccc4C)CC3)s2)[nH]c1	SmoAnta	-1.46	0.446
389	CHEMBL4635021	COc1ccccc1Nc1ccccc1C(=O)N1CCC(N(C)C(=O)c2ccc(F)cc2C(F)(F)F)CC1	SmoAnta	-1.48	0.4
390	CHEMBL2031282	COc1cc(C(=O)N=C(N)Nc2cccc(NC(=O)c3ccccc3)c2)cc(OC)c1OC	SmoAnta	-0.79	0.373
391	CHEMBL3971787	CC(C(=O)Nc1ccc(Cl)c(-c2ccccn2)c1)[C@@H]1CC[C@@H](C)[C@@H]2CCC(=O)C[C@@H]21	SmoAnta	-0.31	0.653
392	CHEMBL4290935	COc1cc(OC)c2c(=O)c(-c3ccc(OCc4ccccc4)c(OCc4ccccc4)c3)coc2c1	SmoAnta	0.28	0.229
393	CHEMBL4518172	Cc1ccc(=NCc2ccccc2-c2nnc(N3CCC(C(=O)NC4CCCCC4C)CC3)s2)[nH]c1	SmoAnta	-1.34	0.502
394	CHEMBL3817987	Cn1c(-c2cccc(N3CCc4sc(C(=O)N5CCOCC5)cc4C3)c2)nc2ccccc21	SmoAnta	-1.86	0.458
395	CHEMBL3262639	Cc1cnc(-c2cc(N3CCn4cc(C(=O)Nc5ccccc5)nc4C3)ncc2Cl)c(C)c1	SmoAnta	-1.58	0.471
396	CHEMBL3126692	CS(=O)(=O)c1ccc(C(=O)Nc2ccc(Cl)c(NC(=O)Nc3ccc(C(F)(F)F)cc3Cl)c2)c(Cl)c1	SmoAnta	-2.16	0.303
397	CHEMBL560932	CCCN(CCC)C(=O)NCc1ccc2c(cnn2-c2ccc(F)cc2)c1	SmoAnta	-1.95	0.668
398	CHEMBL4639888	CN(C(=O)c1ccc([N+](=O)[O-])cc1)C1CCN(C(=O)c2ccccc2N=c2cc[nH]n2C)CC1	SmoAnta	-1.45	0.463
399	CHEMBL4462158	C[C@@]1(c2ccc(Cl)cc2Cl)OC[C@@H](COc2ccc(N3CCN(c4ccc([N+](=O)[O-])cc4)CC3)cc2)O1	SmoAnta	-0.68	0.271
400	CHEMBL3818511	Cn1c(-c2cc(N3CCc4sc(C(=O)N5CCCC5)cc4C3)ccc2Cl)nc2ccccc21	SmoAnta	-1.69	0.378
401	CHEMBL3817945	CN1CCN(C(=O)c2cc3c(s2)CCN(c2cccc(-c4nc5ccccc5n4C)c2)C3)CC1	SmoAnta	-1.72	0.448
402	CHEMBL3890559	CC(C(=O)Nc1ccc(Cl)c(-c2ccccn2)c1)[C@@H]1CC[C@@H](C)[C@@H]2Cc3cnn(-c4ccccc4)c3C[C@@H]21	SmoAnta	-0.91	0.299
403	CHEMBL562811	CCCC(C)(C)C(=O)NCc1ccc2c(cnn2-c2ccc(F)cc2)c1	SmoAnta	-1.49	0.703
404	CHEMBL563112	CCCC(CCC)C(=O)NCc1ccc2c(cnn2-c2cccnc2OC)c1	SmoAnta	-1.34	0.602
405	CHEMBL500172	CC(=O)Nc1ccc(CN[C@H]2Cc3ccc(NC(=O)c4cccc(C)c4-c4ccc(C(F)(F)F)cc4)cc3C2)cc1	SmoAnta	-0.99	0.226
406	CHEMBL3262640	Cc1cnc(-c2cc(N3CCn4cc(C(=O)Nc5ccccc5F)nc4C3)ncc2Cl)c(C)c1	SmoAnta	-1.81	0.447

407	CHEMBL3819236	Cn1c(-c2cccc(N3CCc4sc(C(=O)N5CCCC5)cc4C3)c2)nc2ccccc21	SmoAnta	-1.73	0.443
408	CHEMBL552208	CCCC(C)C(=O)NCc1ccc2c(cnn2-c2ccc(F)cc2)c1	SmoAnta	-1.59	0.731
409	CHEMBL2031292	COc1cc(C(=O)N=C(N)Nc2ccc(Cl)c(NC(=O)c3ccccc3)c2)cc(OC)c1OC	SmoAnta	-1.09	0.34
410	CHEMBL3818879	Cn1c(-c2cccc(N3CCc4sc(C(=O)N5CCN(S(C)(=O)=O)CC5)cc4C3)c2)nc2ccccc21	SmoAnta	-1.93	0.399
411	CHEMBL3262646	Cc1cnc(-c2cc(N3CCn4cc(C(=O)OC(C)C)nc4C3)ncc2Cl)c(C)c1	SmoAnta	-1.3	0.583
412	CHEMBL498017	N#Cc1ccc(N2CCC(c3cn(Cc4ccccc4)c4ccccc34)CC2)nc1	SmoAnta	-1.71	0.471
413	CHEMBL4553897	Cc1c(O)n(-c2ccc(Cl)cc2)c(=O)n1CC1CCCCC1	SmoAnta	-1.17	0.932
414	CHEMBL4583477	C[C@]1(c2ccc(Cl)cc2Cl)OC[C@@H](COc2ccc(N3CCN(c4ccc(N)cc4)CC3)cc2)O1	SmoAnta	-0.4	0.434
415	CHEMBL2152380	O=C(CC1CCCCCC(=O)N[C@H](c2ccc(Cl)cc2)COC1=O)N=Cc1ccc(Cl)cc1	SmoAnta	-0.01	0.466
416	CHEMBL4513409	Cc1c[nH]c(=NCc2ccnn2-c2nnc(N3CCC(C(=O)NC4CCCCC4C)CC3)s2)cn1	SmoAnta	-1.59	0.543
417	CHEMBL3262643	Cc1cnc(-c2cc(N3CCn4cc(C(=O)Nc5ccc(F)c(F)c5)nc4C3)ncc2Cl)c(C)c1	SmoAnta	-1.93	0.424
418	CHEMBL496013	c1ccc(Cc2nnc(N3CCN(c4ccccc4)CC3)c3ccccc23)cc1	SmoAnta	-0.98	0.52
419	CHEMBL3818648	Cn1c(-c2cccc(N3CCc4sc(C(=O)N5CCC(O)CC5)cc4C3)c2)nc2ccccc21	SmoAnta	-1.5	0.478
420	CHEMBL3262642	Cc1cnc(-c2cc(N3CCn4cc(C(=O)Nc5ccc(F)cc5)nc4C3)ncc2Cl)c(C)c1	SmoAnta	-1.77	0.447
421	CHEMBL3290331	C[C@@H]1CN(c2nnc(Cc3ccccc3)c3ccc(Cl)cc23)CCN1c1ccc(C#N)cn1	SmoAnta	-1.64	0.438
422	CHEMBL184721	Cc1ccc(NC(=O)c2cccc(N3CCOCC3)c2)cc1NC(=O)c1ccc(OCc2ccccn2)cc1	SmoAnta	-1.95	0.326
423	CHEMBL184712	Cc1ccc(NC(=O)c2cc(F)cc(N3CCCC3)c2)cc1NC(=O)c1ccc(OCc2ccccn2)cc1	SmoAnta	-2.01	0.286
424	CHEMBL3818296	Cn1c(-<br>c2cccc(N3CCc4sc(C(=O)N5CCN(c6ccc(Cl)c(Cl)c6)CC5)cc4C3)c2)nc2ccccc21	SmoAnta	-1.75	0.22
425	CHEMBL4452315	Cc1ccc(=NCc2ccnn2-c2nnc(N3CCC(C(=O)Nc4cccnc4C)CC3)s2)[nH]c1	SmoAnta	-1.7	0.43
426	CHEMBL3977846	CC(C(=O)Nc1ccc(Cl)c(-<br>c2ccccn2)c1)[C@@H]1CC[C@@H](C)[C@@H]2Cc3c(n(C)c4ccccc34)C[C@@H<br>]21	SmoAnta	-0.5	0.307
427	CHEMBL473892	O=C(Nc1ccc2c(c1)CC(NCc1ccccn1)C2)c1ccccc1-c1ccc(C(F)(F)F)cc1	SmoAnta	-1.37	0.337
428	CHEMBL2031080	COc1cc(C(=O)NC(=S)Nc2ccc(Cl)c(C(=O)Nc3ccccc3)c2)cc(OC)c1OC	SmoAnta	-1.53	0.404
429	CHEMBL3262630	Cc1cnc(-c2cc(N3CCn4cc(C(=O)NCC5CC5)nc4C3)ncc2Cl)c(C)c1	SmoAnta	-1.42	0.658
430	CHEMBL4163487	Cc1ccc(NCc2cccn2-c2nnc(N3CCC(C(=O)NC4CCCC4)CC3)s2)cc1	SmoAnta	-1.91	0.538
431	CHEMBL3262636	COC[C@H]1CCCN1C(=O)c1cn2c(n1)CN(c1cc(-c3ncc(C)cc3C)c(Cl)cn1)CC2	SmoAnta	-1.28	0.551
432	CHEMBL4470299	Cc1ccc(=NCc2ccnn2-c2nnc(N3CCC(C(=O)Nc4cnccc4C)CC3)s2)[nH]c1	SmoAnta	-1.75	0.43
433	CHEMBL4539606	Cc1c(O)n(-c2ccc(Cl)cc2)c(=O)n1-c1ccccc1	SmoAnta	-1.03	0.789
434	CHEMBL4632537	COc1ccc(C(=O)N(C)C2CCN(C(=O)c3ccccc3N=c3cc[nH]n3C)CC2)cc1	SmoAnta	-1.2	0.653
435	CHEMBL3976847	Cc1ccc2c(n1)C(C(C)C(=O)Nc1ccc(Cl)c(-c3nnc(-c4ccnn4C)o3)c1)CC[C@H]2C	SmoAnta	-1.17	0.382
436	CHEMBL2031285	COc1cc(C(=O)N=C(N)Nc2cccc(NC(=O)c3cn4ccsc4n3)c2)cc(OC)c1OC	SmoAnta	-1.55	0.263
437	CHEMBL3262654	COCC(=O)Nc1cn2c(n1)CN(c1cc(-c3ncc(C)cc3C)c(Cl)cn1)CC2	SmoAnta	-1.65	0.674
438	CHEMBL1813099	O=C(Nc1cccc(Cl)c1Cl)N1CCn2c(c(O)n([C@H]3C[C@@H]3c3ccccc3)c2=O)C1	SmoAnta	-1.13	0.608
439	CHEMBL4168100	Cc1ccc(NCc2cccn2-c2nnc(N3CCC(C(=O)NC4CCCCC4)CC3)s2)cc1	SmoAnta	-1.87	0.504
440	CHEMBL1813098	O=C(Nc1cccc(Cl)c1Cl)N1CCn2c(c(O)n([C@@H]3C[C@H]3c3ccccc3)c2=O)C1	SmoAnta	-1.13	0.608
441	CHEMBL556427	CCCC(CCC)C(=O)NCc1ccc2c(cnn2-c2cccc(F)c2)c1	SmoAnta	-1.67	0.609
442	CHEMBL3262627	CCOC(=O)c1cn2c(n1)CN(c1cc(-c3ncc(C)cc3C)c(Cl)cn1)CC2	SmoAnta	-1.35	0.608
443	CHEMBL497003	c1ccc(N2CCN(c3nnc(Cc4ccncc4)c4ccccc34)CC2)nc1	SmoAnta	-1.42	0.54
444	CHEMBL550254	CCC(CC)C(=O)NCc1ccc2c(cnn2-c2ccc(F)cc2)c1	SmoAnta	-1.68	0.731
445	CHEMBL497210	FC(F)(F)c1ccc(N2CCN(c3nnc(Cc4ccccc4)c4[nH]cnc34)CC2)nc1	SmoAnta	-1.54	0.523
446	CHEMBL3262648	Cc1cnc(-c2cc(N3CCn4cc(C(=O)OC5CCCC5)nc4C3)ncc2Cl)c(C)c1	SmoAnta	-1	0.534
447	CHEMBL4645447	COc1ccc(Nc2ccccc2C(=O)N2CCC(N(C)C(=O)c3ccc(F)cc3C(F)(F)F)CC2)cc1	SmoAnta	-1.57	0.4
448	CHEMBL474892	Cc1cccc(C(=O)Nc2ccc3c(c2)C[C@H](NCc2nccs2)C3)c1-c1ccc(C(F)(F)F)cc1	SmoAnta	-1.38	0.307
449	CHEMBL3262633	Cc1cnc(-c2cc(N3CCn4cc(C(=O)N5CCOCC5)nc4C3)ncc2Cl)c(C)c1	SmoAnta	-1.63	0.607
450	CHEMBL2031254	COc1cc(C(=O)NC(=S)Nc2cccc(-c3cn4ccccc4n3)c2)cc(OC)c1OC	SmoAnta	-1.96	0.416
451	CHEMBL3604617	CC(=O)N=c1[nH]c2ccc(-c3cnc(Cl)c(NC(=O)c4ccc(C)cc4)c3)cc2s1	SmoAnta	-1.67	0.445
452	CHEMBL4063037	O=C(c1ccccc1)c1c(-c2ccccc2)[nH]c2ccc(Br)cc2c1=O	SmoAnta	-0.29	0.478
453	CHEMBL4284509	CN(C(=O)c1ccc(B(O)O)cc1)C1CCN(c2nnc(-c3ccnn3C)c3ccccc23)CC1	SmoAnta	-1.38	0.425
454	CHEMBL550661	CCCC(CCC)C(=O)NCc1ccc2c(cnn2-c2ccccc2F)c1	SmoAnta	-1.55	0.609
455	CHEMBL2031286	COc1cc(C(=O)N=C(N)Nc2cccc(-n3ccc4ccccc43)c2)cc(OC)c1OC	SmoAnta	-1	0.341
456	CHEMBL563992	CCCC(CCC)C(=O)NCc1ccc2c(cnn2-c2ccc(C)cc2)c1	SmoAnta	-1.33	0.6
457	CHEMBL3262653	CCC(=O)Nc1cn2c(n1)CN(c1cc(-c3ncc(C)cc3C)c(Cl)cn1)CC2	SmoAnta	-1.58	0.704
458	CHEMBL4090942	COc1cccc(C(=O)c2c(-c3ccccc3)[nH]c3ccccc3c2=O)c1	SmoAnta	-0.23	0.551
459	CHEMBL3262651	Cc1cnc(-c2cc(N3CCn4cc(CN5CCOCC5)nc4C3)ncc2Cl)c(C)c1	SmoAnta	-1.77	0.621
460	CHEMBL497819	N#Cc1ccc(N2CCN(c3cnc(Cc4ccccc4)c4ccccc34)CC2)nc1	SmoAnta	-1.41	0.503

461	CHEMBL4471156	C[C@@]1(c2ccc(Cl)cc2Cl)OC[C@@H](COc2ccc(N3CCN(c4ccc(N)cc4)CC3)cc2)O1	SmoAnta	-0.4	0.434
462	CHEMBL3604620	CC(=O)N=c1[nH]c2ccc(-c3cnc(Cl)c(NCc4ccccc4)c3)cc2s1	SmoAnta	-1.57	0.466
463	CHEMBL3262649	Cc1cnc(-c2cc(N3CCn4cc(C(=O)Oc5ccccc5)nc4C3)ncc2Cl)c(C)c1	SmoAnta	-1.26	0.321
464	CHEMBL561995	CCCC(CCC)C(=O)NCc1ccc2c(cnn2-c2c(C)cccc2C)c1	SmoAnta	-1.21	0.565
465	CHEMBL4171049	Cc1oc(-c2cccc(Cl)c2)nc1CN1CCC(C(=O)NC2CCCc3ccccc32)CC1	SmoAnta	-1.79	0.526
466	CHEMBL3955601	Cc1c(Cc2ccccc2)nnc(N2CCC(C)(c3nc4cccnc4[nH]3)CC2)c1C	SmoAnta	-0.66	0.534
467	CHEMBL3960935	Cc1c(-c2ccc(F)cc2)nnc(N2CCN(c3cnc(C(=O)O)cn3)[C@H](C)C2)c1C	SmoAnta	-1.34	0.685
468	CHEMBL3915704	Cc1c(Cc2ccccc2)nnc(N2CCN(Cc3ccccc3)CC2)c1C	SmoAnta	-1.01	0.677
469	CHEMBL3919359	COC(=O)c1cnc(N2CCN(c3nnc(-c4ccccc4-c4ccccc4)c(C)c3C)C[C@H]2C)cn1	SmoAnta	-0.91	0.369
470	CHEMBL3910002	Cc1c(Cc2ccccc2)nnc(N2CCN(c3ncc(C(=O)O)c(C(F)(F)F)n3)[C@H](C)C2)c1C	SmoAnta	-1.07	0.58
471	CHEMBL3981990	COC(=O)c1cnc(N2CCN(c3nnc(-c4ccc(F)c(Cl)c4)c(C)c3C)C[C@H]2C)cn1	SmoAnta	-1.63	0.532
472	CHEMBL3973278	COC(=O)c1cnc(N2CCN(c3nnc(-c4ccc(OC)cc4)c(C)c3C)C[C@H]2C)cn1	SmoAnta	-1.15	0.546
473	CHEMBL3909304	Cc1ccc2c(n1)C(C(C)C(=O)Nc1cccc(-c3ccc(OC(F)(F)F)cc3)c1C)CC[C@H]2C	SmoAnta	-0.51	0.409
474	CHEMBL3967640	COC(=O)c1cnc(N2CCN(c3nnc(-c4ccc(C(F)(F)F)cc4)c(C)c3C)C[C@H]2C)cn1	SmoAnta	-1.33	0.511
475	CHEMBL3970317	COC(=O)c1cnc(N2CCN(c3nnc(-c4ccc(F)cc4)c(C)c3C)C[C@H]2C)cn1	SmoAnta	-1.41	0.577
476	CHEMBL3920191	COC(=O)c1cnc(N2CCN(c3nnc(Cc4ccccc4)c4c3CCC4)C[C@H]2C)cn1	SmoAnta	-1.11	0.556
477	CHEMBL3916441	Cc1c(-c2ccccc2)nnc(N2CCN(c3ccc(C(F)(F)F)cn3)CC2)c1C	SmoAnta	-1.69	0.633
478	CHEMBL4293519	CN(C(=O)c1ccccc1)C1CCN(c2nnc(-c3ccnn3C)c3ccccc23)CC1	SmoAnta	-1.54	0.497
479	CHEMBL366255	Cc1ccc(NC(=O)c2cccc(N(C)C)c2)cc1NC(=O)c1ccc(OCc2ccccn2)cc1	SmoAnta	-1.85	0.346
480	CHEMBL2031256	COc1cc(C(=O)NC(=S)Nc2cccc(-c3cn4ccsc4n3)c2)cc(OC)c1OC	SmoAnta	-2.09	0.41
481	CHEMBL4161601	Cc1ccc(NCc2cccn2-c2nnc(N3CCC(C(=O)NCC(C)C)CC3)s2)cc1	SmoAnta	-1.89	0.535
482	CHEMBL515245	O=C(Nc1ccc2c(c1)CC(NCc1ccccn1)C2)c1ccccc1-c1ccccc1	SmoAnta	-1.24	0.448
483	CHEMBL1813112	C[C@]12CN(C(=O)Nc3ccccc3-c3ccccc3)CCN1C(=O)N([C@H]1C[C@@H]1c1ccccc1)C2=O	SmoAnta	-0.53	0.546
484	CHEMBL559001	CCCC(CCC)C(=O)NCc1ccc2c(cnn2-c2ccc(Cl)cc2)c1	SmoAnta	-1.44	0.557
485	CHEMBL568916	CCCCC(=O)NCc1ccc2c(cnn2-c2ccc(F)cc2)c1	SmoAnta	-1.71	0.745
486	CHEMBL3262637	Cc1cnc(-c2cc(N3CCn4cc(C(=O)N5CCC(O)CC5)nc4C3)ncc2Cl)c(C)c1	SmoAnta	-1.27	0.637
487	CHEMBL1600636	Cc1ccc(NCc2cccn2-c2nnc(N3CCC(C(=O)NC(C)C)CC3)s2)cc1	SmoAnta	-2.01	0.583
488	CHEMBL4175623	O=C(Nc1cccc(N2CCCN(Cc3ccc(F)c(F)c3)C2=O)c1)OCc1ccccc1	SmoAnta	-1.9	0.535
489	CHEMBL4215992	Cc1cc(-c2ncc(CNC(=O)c3ccc4c(c3)[nH]c3ccccc34)cc2C#N)ccn1	SmoAnta	-1.02	0.435
490	CHEMBL551932	CCCN(CCC)C(=O)N(C)Cc1ccc2c(cnn2-c2ccc(F)cc2)c1	SmoAnta	-1.8	0.587
491	CHEMBL3259848	CCNC(=O)c1cn2c(n1)CN(c1cc(-c3ncc(C)cc3C)c(Cl)cn1)CC2	SmoAnta	-1.55	0.713
492	CHEMBL4641704	CN(C(=O)c1ccc(C(F)(F)F)cc1)C1CCN(C(=O)c2ccccc2N=c2cc[nH]n2C)CC1	SmoAnta	-1.38	0.607
493	CHEMBL551873	CCCC(CCC)C(=O)NCc1ccc2c(cnn2-c2ccc(C(C)=O)cc2)c1	SmoAnta	-1.21	0.519
494	CHEMBL497820	N#Cc1ccc(N2CCN(c3ncc(Cc4ccccc4)c4ccccc34)CC2)nc1	SmoAnta	-1.42	0.503
495	CHEMBL4290843	CN(C)c1ccc(C(=O)N(C)C2CCN(c3nnc(-c4ccnn4C)c4ccccc34)CC2)cc1	SmoAnta	-1.59	0.442
496	CHEMBL3262638	Cc1cnc(-c2cc(N3CCn4cc(C(=O)N5CC(C)NC(C)C5)nc4C3)ncc2Cl)c(C)c1	SmoAnta	-1.19	0.619
497	CHEMBL4450803	Cc1c(O)n(-c2c(F)c(F)c(F)c(F)c2F)c(=O)n1Cc1ccccc1	SmoAnta	-0.8	0.436
498	CHEMBL4284830	CN(C(=O)c1ccc(F)cc1)C1CCN(c2nnc(-c3ccnn3C)c3ccccc23)CC1	SmoAnta	-1.77	0.478
499	CHEMBL523431	O=C(O)c1ccc(N2CCN(c3nnc(Cc4ccccc4)c4ccccc34)CC2)nc1	SmoAnta	-1.21	0.523
500	CHEMBL1813100	O=C(Nc1cccc(Cl)c1Cl)N1CCn2c(c(O)n([C@@H]3C[C@H]3c3ccccc3)c2=O)C1	SmoAnta	-1.13	0.608
501	CHEMBL4173721	Cc1ccc(NCc2cccn2-c2nnc(N3CCC(C(=O)NCC4CC4)CC3)s2)cc1	SmoAnta	-1.99	0.543
502	CHEMBL559874	CCCC(CCC)C(=O)NCc1ccc2c(cnn2-c2ccc(C(=O)OC(C)(C)C)cc2)c1	SmoAnta	-1.06	0.418
503	CHEMBL3262652	Cc1cnc(-c2cc(N3CCn4cc(NC(=O)C(F)(F)F)nc4C3)ncc2Cl)c(C)c1	SmoAnta	-1.47	0.649
504	CHEMBL2031079	COc1cc(C(=O)NC(=S)Nc2ccc(C)c(C(=O)Nc3ccccc3)c2)cc(OC)c1OC	SmoAnta	-1.41	0.432
505	CHEMBL497209	FC(F)(F)c1ccc(N2CCN(c3nnc(Cc4ccccc4)c4ccoc34)CC2)nc1	SmoAnta	-1.3	0.465
506	CHEMBL4474558	Cc1ccc(=NCc2c(-c3nnc(N4CCC(C(=O)NC5CCCCC5C)CC4)s3)cnn2C)[nH]c1	SmoAnta	-1.61	0.53
507	CHEMBL4294637	CN(C(=O)c1ccc(OCc2ccccc2)cc1)C1CCN(c2nnc(-c3ccnn3C)c3ccccc23)CC1	SmoAnta	-1.4	0.281
508	CHEMBL557448	CCCC(CCC)C(=O)NCc1ccc2c(cnn2-c2cccnc2)c1	SmoAnta	-1.41	0.66
509	CHEMBL4646599	CC(=O)c1ccc(C(=O)N(C)C2CCN(C(=O)c3ccccc3N=c3cc[nH]n3C)CC2)cc1	SmoAnta	-1.16	0.594
510	CHEMBL4279209	CN(C(=O)c1ccc(N)cc1)C1CCN(c2nnc(-c3ccnn3C)c3ccccc23)CC1	SmoAnta	-1.5	0.488
511	CHEMBL4455409	C[C@]1(c2ccc(Cl)cc2Cl)OC[C@@H](COc2ccc(N3CCN(c4ccc(NC(=O)c5ccccc5O)cc4)CC3)cc2)O1	SmoAnta	-0.66	0.217
512	CHEMBL1086141	C[C@H]1CN(c2nnc(N3CC=CC=N3)c3ccccc23)CCN1C(=O)c1ccccc1	SmoAnta	-0.91	0.66
513	CHEMBL4164165	O=C(Nc1ccc(N2CCN(Cc3ccccc3O)CC2)nc1)c1ccccc1C(F)(F)F	SmoAnta	-1.85	0.597
514	CHEMBL423915	COC(=O)N[C@@H]1Cc2ccc(NC(=O)c3cccc(C)c3-c3ccc(C(F)(F)F)cc3)cc2C1	SmoAnta	-1.03	0.512

515	CHEMBL4290044	COc1cc(OC)c2c(=O)c(-c3ccc(OC)c(OCc4ccc(C(F)(F)F)cc4)c3)coc2c1	SmoAnta	0.06	0.315
516	CHEMBL3262629	CNC(=O)c1cn2c(n1)CN(c1cc(-c3ncc(C)cc3C)c(Cl)cn1)CC2	SmoAnta	-1.42	0.736
517	CHEMBL1084495	C[C@H]1CN(c2nnc(-c3ncco3)c3ccccc23)CCN1C(=O)c1ccccc1	SmoAnta	-1.19	0.524
518	CHEMBL473703	COC(=O)NC1Cc2ccc(NC(=O)c3cccc(C)c3-c3ccc(C(F)(F)F)cc3)cc2C1	SmoAnta	-1.03	0.512
519	CHEMBL473073	O=C(Nc1ccc2c(c1)CC(NCc1ccccn1)C2)c1ccccc1-c1ccc(Cl)cc1	SmoAnta	-1.39	0.381
520	CHEMBL4282560	CN(C(=O)c1ccc(S(=O)(=O)O)cc1)C1CCN(c2nnc(-c3ccnn3C)c3ccccc23)CC1	SmoAnta	-1.5	0.41
521	CHEMBL4282987	CN(C(=O)c1ccc(C=O)cc1)C1CCN(c2nnc(-c3ccnn3C)c3ccccc23)CC1	SmoAnta	-1.37	0.429
522	CHEMBL4285647	COc1cc(OC)c2c(=O)c(-c3ccc(OC)c(OCC=C(C)C)c3)coc2c1	SmoAnta	0.94	0.533
523	CHEMBL564987	CCCC(CCC)C(=O)N(C)Cc1ccc2c(cnn2-c2ccc(F)cc2)c1	SmoAnta	-1.56	0.528
524	CHEMBL4172388	CC1Cc2ccccc2N1C(=O)c1ccc(=O)n(CCC(=O)N2CCN(c3ccccc3)CC2)n1	SmoAnta	-1.76	0.572
525	CHEMBL4289354	CN(C(=O)c1ccc(C(N)=O)cc1)C1CCN(c2nnc(-c3ccnn3C)c3ccccc23)CC1	SmoAnta	-1.55	0.481
526	CHEMBL4866798	[N-]=[N+]=NCCOCCOCCOCCOCCOCCS(=O)(=O)c1ccc(C(=O)Nc2ccc(Cl)c(-c3ccccn3)c2)c(Cl)c1	SmoAnta	-1.31	0.062
527	CHEMBL4543531	C[C@]1(c2ccc(Cl)cc2Cl)OC[C@@H](COc2ccc(N3CCN(c4ccc(NC(=O)c5ccc(O)cc5)cc4)CC3)cc2)O1	SmoAnta	-0.59	0.217
528	CHEMBL365472	Cc1ccc(NC(=O)c2cccc(N(C)C)c2)cc1NC(=O)c1ccc(OC(C)C)cc1	SmoAnta	-1.66	0.529
529	CHEMBL4289775	CN(C(=O)c1ccc(S(N)(=O)=O)cc1)C1CCN(c2nnc(-c3ccnn3C)c3ccccc23)CC1	SmoAnta	-1.74	0.441
530	CHEMBL3604619	CC(=O)N=c1[nH]c2ccc(-c3cnc(Cl)c(NC(=O)Cc4ccccc4)c3)cc2s1	SmoAnta	-1.65	0.459
531	CHEMBL4293248	COc1cc(OC)c2c(=O)c(-c3ccc(OCc4ccc(C(F)(F)F)cc4)c(OC)c3)coc2c1	SmoAnta	0.05	0.315
532	CHEMBL4215124	COc1cc(CNC(=O)c2ccc3c(c2)[nH]c2ccccc23)cnc1-c1ccnc(C)c1	SmoAnta	-0.74	0.417
533	CHEMBL4291101	COc1cc(OC)c2c(=O)c(-c3ccc(OC)c(OC/C=C(\C)CCC=C(C)C)c3)coc2c1	SmoAnta	1.25	0.317
534	CHEMBL4562011	CC(O)c1ccnn1-c1nnc(N2CCC(C(=O)NC3CCCCC3C)CC2)s1	SmoAnta	-1.61	0.775
535	CHEMBL4286606	COc1cc(OC)c2c(=O)c(-c3ccc(OC)c(OCc4ccccc4)c3)coc2c1	SmoAnta	0.36	0.418
536	CHEMBL495579	N#Cc1ccc(C2CCN(c3nnc(Cc4ccccc4)c4ccccc34)CC2)cc1	SmoAnta	-1.03	0.452
537	CHEMBL4467625	Cc1c(O)n(-c2ccc(Cl)cc2)c(=S)n1Cc1ccccc1	SmoAnta	-1.37	0.704
538	CHEMBL4473720	C[C@]1(c2ccc(Cl)cc2Cl)OC[C@@H](COc2ccc(N3CCN(c4ccc(NC(=O)c5ccccn5)cc4)CC3)cc2)O1	SmoAnta	-0.94	0.237
539	CHEMBL2152376	O=C(CC1CCCCCC(=O)N[C@H](Cc2ccccc2)COC1=O)N=Cc1ccc(Cl)cc1	SmoAnta	-0.05	0.516
540	CHEMBL2152370	O=C(C=C1CCCCCC(=O)O[C@H](c2ccccc2)CNC1=O)NCCCC(F)(F)F	SmoAnta	0.23	0.415
541	CHEMBL2152364	CC1CC=CCC(CC(=O)NCc2ccc(Cl)cc2)C(=O)OC[C@@H](c2ccccc2)NC1=O	SmoAnta	0.24	0.504
542	CHEMBL3262631	Cc1cnc(-c2cc(N3CCn4cc(C(=O)N(C)CCO)nc4C3)ncc2Cl)c(C)c1	SmoAnta	-1.5	0.656
543	CHEMBL3262650	Cc1cnc(-c2cc(N3CCn4cc(CO)nc4C3)ncc2Cl)c(C)c1	SmoAnta	-1.28	0.768
544	CHEMBL3604611	CC(=O)N=c1[nH]c2ccc(-c3cnc(Cl)c(NC(=O)c4ccccc4Cl)c3)cc2s1	SmoAnta	-1.81	0.41
545	CHEMBL4171870	Cc1ccc(NCc2cccn2-c2nnc(N3CCC(C(=O)NC4CC4)CC3)s2)cc1	SmoAnta	-2.06	0.59
546	CHEMBL2152381	O=C(CC1CCCCCC(=O)N[C@H](c2ccc(Cl)cn2)COC1=O)N=Cc1ccc(Cl)cc1	SmoAnta	-0.23	0.495
547	CHEMBL4169989	Cc1ccc(NCc2cccn2-c2nnc(N3CCC(C(=O)NCC(C)(C)O)CC3)s2)cc1	SmoAnta	-1.68	0.469
548	CHEMBL4176279	Cc1ccc(NCc2cccn2-c2nnc(N3CCC(C(=O)N4CCCC4)CC3)s2)cc1	SmoAnta	-1.95	0.613
549	CHEMBL4571341	C[C@@]1(c2ccc(Cl)cc2Cl)OC[C@@H](COc2ccc(N3CCN(c4ccc(NC(=O)c5cccnc5)cc4)CC3)cc2)O1	SmoAnta	-0.88	0.237
550	CHEMBL4644469	CN(C(=O)c1ccc(C#N)cc1)C1CCN(C(=O)c2ccccc2N=c2cc[nH]n2C)CC1	SmoAnta	-1.48	0.673
551	CHEMBL557518	CCCC(CCC)C(=O)NCc1ccc2c(cnn2-c2ccncc2)c1	SmoAnta	-1.35	0.66
552	CHEMBL4551454	C[C@H]1CN(c2ccc(Cl)cc2)C(=O)N1Cc1ccccc1	SmoAnta	-1.22	0.834
553	CHEMBL4168517	Cc1ccc(-c2cc3c(N4CCC(C(=O)NC(C)c5ccccc5)CC4)nccn3n2)cc1C	SmoAnta	-1.81	0.456
554	CHEMBL4647112	CN(C(=O)c1ccc(F)cc1C(F)(F)F)C1CCN(C(=O)c2ccccc2Nc2ccc(-c3ccccc3)cc2)CC1	SmoAnta	-1.45	0.241
555	CHEMBL2152375	O=C(CC1CCCCCC(=O)N[C@H](C2CCCCC2)COC1=O)N=Cc1ccc(Cl)cc1	SmoAnta	0.06	0.513
556	CHEMBL2152373	O=C(C=C1CCCCCC(=O)O[C@H](c2ccccc2)CNC1=O)NCc1cccc(Cl)c1	SmoAnta	-0.05	0.534
557	CHEMBL2152379	O=C(CC1CCCCCC(=O)N[C@H](c2ccc(F)cc2)COC1=O)N=Cc1ccc(Cl)cc1	SmoAnta	-0.28	0.512
558	CHEMBL2152357	O=C(CC1CCCCCC(=O)N[C@H](c2ccccc2)COC1=O)N=Cc1ccc(Cl)cc1	SmoAnta	-0.06	0.538
559	CHEMBL3604615	CC(=O)N=c1[nH]c2ccc(-c3cnc(Cl)c(NC(=O)c4cccc(F)c4)c3)cc2s1	SmoAnta	-1.95	0.449
560	CHEMBL142450	COC(=O)N[C@H]1Cc2ccc(NC(=O)c3cccc(C)c3-c3ccc(C(F)(F)F)cc3)cc2C1	SmoAnta	-1.03	0.512
561	CHEMBL4171838	CCNC(=O)C1CCN(c2nnc(-n3cccc3CNc3ccc(C)cc3)s2)CC1	SmoAnta	-2.1	0.606
562	CHEMBL4456292	COc1cccc(C(=O)Nc2ccc(N3CCN(c4ccc(OC[C@@H]5CO[C@](C)(c6ccc(Cl)cc6Cl)O5)cc4)CC3)cc2)c1	SmoAnta	-0.81	0.205
563	CHEMBL2152362	O=C(C=C1CCCC[C@H](Cc2ccccc2)C(=O)N[C@H](c2ccccc2)COC1=O)NCc1ccc(Cl)cc1	SmoAnta	0.05	0.307
564	CHEMBL550191	CCCC(CCC)C(=O)NCc1ccc2c(cnn2-c2ncccn2)c1	SmoAnta	-1.47	0.672
565	CHEMBL4632758	CN(C(=O)c1ccc(F)cc1C(F)(F)F)C1CCN(C(=O)c2ccccc2Nc2ccc(C#N)cc2)CC1	SmoAnta	-1.82	0.426

566	CHEMBL2152371	CC(C)(C)OC(=O)C=C1CCCCCC(=O)N[C@H](c2ccccc2)COC1=O	SmoAnta	0.4	0.619
567	CHEMBL4289866	COc1cc(OC)c2c(=O)c(-c3ccc(OCc4ccccc4)c(OC)c3)coc2c1	SmoAnta	0.35	0.418
568	CHEMBL4279181	COc1cc(OC)c2c(=O)c(-c3ccc(OCC=C(C)C)c(OC)c3)coc2c1	SmoAnta	0.93	0.533
569	CHEMBL4649551	CN(C(=O)c1ccc(F)cc1C(F)(F)F)C1CCN(C(=O)c2ccc[nH]c2=Nc2ccccc2)CC1	SmoAnta	-1.47	0.524
570	CHEMBL4562945	Cc1c(O)n(-c2ccc(Cl)cc2)c(=O)n1Cc1c(F)c(F)c(F)c(F)c1F	SmoAnta	-1.1	0.407
571	CHEMBL4159684	Cc1ccc(NCc2cccn2-c2nnc(N3CCC(C(=O)N4CCC(O)C4)CC3)s2)cc1	SmoAnta	-1.86	0.581
572	CHEMBL2152359	C[C@H]1[C@@H](c2ccccc2)OC(=O)CCCCCC(CC(=O)N=Cc2ccc(Cl)cc2)C(=O)N1<br>C	SmoAnta	0.23	0.432
573	CHEMBL2152361	CC1CC=CCC(CC(=O)NCc2ccc(Cl)cc2)C(=O)OC[C@@H](c2ccccc2)NC1=O	SmoAnta	0.24	0.504
574	CHEMBL2152372	CN1C[C@@H](c2ccccc2)OC(=O)CC/C=C/CC(Cc2ccc(Cl)cc2)C1=O	SmoAnta	0.56	0.528
575	CHEMBL4537455	Cc1c(O)n(C2CCCCC2)c(=O)n1Cc1ccccc1	SmoAnta	-0.82	0.941
576	CHEMBL4582942	Cc1c(O)n(Cc2ccc(Cl)cc2)c(=O)n1Cc1ccccc1	SmoAnta	-0.9	0.797
577	CHEMBL474894	Cc1ccc(-c2ccc(C(F)(F)F)cc2)c(C(=O)Nc2ccc3c(c2)CC(NCc2ccccn2)C3)c1	SmoAnta	-1.37	0.314
578	CHEMBL356310	Cc1cccc(C(=O)Nc2ccc3c(c2)C[C@H](NCc2ccccn2)C3)c1-c1ccc(C(F)(F)F)cc1	SmoAnta	-1.31	0.314
579	CHEMBL4633504	CN(C(=O)c1ccc(F)cc1C(F)(F)F)C1CCN(C(=O)c2ccccc2Nc2ccccc2C#N)CC1	SmoAnta	-1.73	0.426

The 578 SMO antagonists were further curated for a principal moment of inertia (PMI) calculation. Compounds with more than one stereocenter were omitted from the calculation, when they had 1 or more centers of undefined stereochemistry (69 compounds) resulting in 509 SMO antagonists.

The PMI values were calculated using this script:
https://github.com/mpimp-comas/2023_bag_bio_diverse_pnps

No.	Compound ID	Smiles	DataSet	PMI1	PMI2
1	*ent*-**3a**	O=C1N(CC2=CC=CC=C2)C3=CC=CC=C3[C@H]4[C@H]1[C@@H](C5=CC=CC=C5)[<br>C@@H](C(OC)=O)N4	PNP	0.342	0.782
2	CHEMBL4632769	Cc1cc(C)c(-c2ncc[nH]2)cc1NC(=O)c1ccc(OCc2ccccn2)cc1	SmoAnta	0.083	0.954
3	CHEMBL2160074	C[C@@H]1CN(c2ccc3c(=Nc4ccc(Cl)c(-c5nc(-<br>c6ccccc6)c[nH]5)c4)[nH]ccc3c2)CCO1	SmoAnta	0.18	0.896
4	CHEMBL2160077	Clc1ccc(N=c2[nH]ccc3cc(N4CCOCC4)ccc23)cc1-c1nc(-c2ccccc2)c[nH]1	SmoAnta	0.11	0.933
5	CHEMBL2160072	C[C@H]1CN(c2ccc3c(=Nc4ccc(Cl)c(-c5nc(-<br>c6ccccc6)c[nH]5)c4)[nH]ccc3c2)C[C@@H](C)O1	SmoAnta	0.11	0.934
6	CHEMBL4637222	Cc1cc(C)c(-c2cn(C)cn2)cc1NC(=O)c1ccc(OCc2ccccn2)cc1	SmoAnta	0.11	0.925
7	CHEMBL2160068	C[C@H]1CN(c2ccc3c(=Nc4ccc(Cl)c(-c5nc(-<br>c6ccccc6)c[nH]5)c4)[nH]ccc3n2)C[C@@H](C)O1	SmoAnta	0.1	0.95
8	CHEMBL2160078	Cc1ccc(NC(=O)c2ccc(N3CCOCC3)cc2)cc1-c1nc(-c2ccccc2)c[nH]1	SmoAnta	0.081	0.937
9	CHEMBL2160070	C[C@H]1CN(c2ccc3c(Nc4ccc(Cl)c(-c5nc(-<br>c6ccccc6)c[nH]5)c4)cccc3n2)C[C@@H](C)O1	SmoAnta	0.144	0.933
10	CHEMBL1083915	Cc1ccc(-c2nnc(N3CCN(C(=O)c4ccccc4)C[C@H]3C)c3ccccc23)cc1	SmoAnta	0.123	0.942
11	CHEMBL473417	CS(=O)(=O)c1ccc(C(=O)Nc2ccc(Cl)c(-c3ccccn3)c2)c(Cl)c1	SmoAnta	0.129	0.902
12	CHEMBL2160071	C[C@H]1CN(c2ccc3c(=Nc4ccc(Cl)c(-c5nc(-<br>c6ccccc6)c[nH]5)c4)cc[nH]c3c2)C[C@@H](C)O1	SmoAnta	0.105	0.938
13	CHEMBL2160079	COc1cc(OC)cc(C(=O)Nc2ccc(C)c(-c3nc(-c4ccccc4)c[nH]3)c2)c1	SmoAnta	0.148	0.909
14	CHEMBL2160076	Clc1ccc(N=c2[nH]ccc3cc(N4CCCCC4)ccc23)cc1-c1nc(-c2ccccc2)c[nH]1	SmoAnta	0.108	0.936
15	CHEMBL2160067	C[C@@H]1CN(c2ccc3c(=Nc4ccc(Cl)c(-c5nc(-<br>c6ccccc6)c[nH]5)c4)[nH]ccc3n2)CCO1	SmoAnta	0.108	0.937
16	CHEMBL1086467	C[C@@H]1CN(C(=O)c2ccccc2)CCN1c1nnc(-c2ccc(Cl)cc2)c2ccccc12	SmoAnta	0.107	0.951
17	CHEMBL1209455	C[C@@H]1CN(C(=O)c2ccccc2)CCN1c1nnc(-c2ccc(C(F)(F)F)cc2)c2ccncc12	SmoAnta	0.1	0.961
18	CHEMBL4288592	CN(C(=O)c1ccc(Oc2ccccc2)cc1C(F)(F)F)C1CCN(c2nnc(-c3ccnn3C)c3ccccc23)CC1	SmoAnta	0.107	0.965

19	CHEMBL497266	CC(C)(N)c1ccc(N2CCN(c3nnc(Cc4ccccc4)c4ccccc34)CC2)nc1	SmoAnta	0.099	0.967
20	CHEMBL538867	CC1=C2C[C@H]3[C@@H](CC[C@@H]4C[C@H](NS(C)(=O)=O)CC[C@]34C)[C@@H]2CC[C@@]2(C1)O[C@@H]1C[C@H](C)CN[C@H]1[C@H]2C	SmoAnta	0.097	0.968
21	CHEMBL2160073	O=S1(=O)CCN(c2ccc3c(=Nc4ccc(Cl)c(-c5nc(-c6ccccc6)c[nH]5)c4)[nH]ccc3c2)CC1	SmoAnta	0.099	0.945
22	CHEMBL1084835	C[C@@H]1CN(C(=O)c2ccccc2)CCN1c1nnc(-c2ccccc2)c2ccccc12	SmoAnta	0.128	0.943
23	CHEMBL1084730	C[C@@H]1CN(C(=O)c2ccccc2)CCN1c1nnc(-c2ccc(C3CC3)cc2)c2ccccc12	SmoAnta	0.107	0.956
24	CHEMBL2059865	Cc1ccc(-c2ncc[nH]2)cc1NC(=O)c1ccc(OCc2ccccn2)cc1	SmoAnta	0.084	0.941
25	CHEMBL2059863	Cc1ccc(-c2nc(C(F)(F)F)c[nH]2)cc1NC(=O)c1ccc(OCc2ccccn2)cc1	SmoAnta	0.109	0.931
26	CHEMBL2142592	CN(C(=O)c1ccc(F)cc1C(F)(F)F)C1CCN(c2nnc(-c3ccnn3C)c3ccccc23)CC1	SmoAnta	0.11	0.967
27	CHEMBL2059864	Cc1c[nH]c(-c2ccc(C)c(NC(=O)c3ccc(OCc4ccccn4)cc3)c2)n1	SmoAnta	0.087	0.944
28	CHEMBL2160208	O=C(NC1CCN(Cc2ccc(N3CCC(NC(=O)c4cccc(-c5cccnc5)c4)CC3)nc2)CC1)c1cccc(-c2cccnc2)c1	SmoAnta	0.076	0.965
29	CHEMBL1084734	C[C@@H]1CN(C(=O)c2ccccc2)CCN1c1nnc(-c2ccc(CO)cc2)c2ccccc12	SmoAnta	0.111	0.955
30	CHEMBL485870	CC(C)(O)c1ccc(N2CCN(c3nnc(Cc4ccccc4)c4ccccc34)CC2)nc1	SmoAnta	0.098	0.96
31	CHEMBL1084738	C[C@@H]1CN(C(=O)c2ccccc2)CCN1c1nnc(-c2ccc(C(F)(F)F)cc2)c2ccccc12	SmoAnta	0.1	0.962
32	CHEMBL1209454	C[C@@H]1CN(C(=O)c2ccccc2)CCN1c1nnc(-c2ccccc2)c2ccncc12	SmoAnta	0.13	0.948
33	CHEMBL2160069	C[C@H]1CN(c2cnc3c(Nc4ccc(Cl)c(-c5nc(-c6ccccc6)c[nH]5)c4)cccc3n2)C[C@@H](C)O1	SmoAnta	0.161	0.917
34	CHEMBL4539839	Cc1c(O)n(-c2ccc(Cl)cc2)c(=O)n1Cc1ccc(N=[N+]=[N-])cc1	SmoAnta	0.215	0.896
35	CHEMBL516246	COc1cc(OC)cc(C(=O)Nc2ccc(Cl)c(-c3nc4cc(N(C)C)ccc4[nH]3)c2)c1	SmoAnta	0.179	0.877
36	CHEMBL1084736	C[C@@H]1CN(C(=O)c2ccccc2)CCN1c1nnc(-c2ccc(C#N)cc2)c2ccccc12	SmoAnta	0.124	0.941
37	CHEMBL1083284	Cc1ccc(-c2nnc(N3CCN(C(=O)c4ccccc4)[C@@H](C)C3)c3ccccc23)cc1	SmoAnta	0.12	0.944
38	CHEMBL1209189	CC(=O)Oc1ccc(-c2nnc(N3CCN(C(=O)c4ccccc4)C[C@H]3C)c3ccccc23)cc1	SmoAnta	0.096	0.961
39	CHEMBL4278388	CN(C(=O)c1ccc(F)cc1C(F)(F)F)C1CCN(c2ccc(-c3ccnn3C)c3ccccc23)CC1	SmoAnta	0.111	0.962
40	CHEMBL1083914	C[C@H]1CN(c2nnc(-c3ccc(C(F)(F)F)cc3)c3ccccc23)CCN1C(=O)c1ccccc1	SmoAnta	0.102	0.952
41	CHEMBL1813106	O=C(Nc1cccnc1-c1cccc(Cl)c1)N1CCn2c(c(O)n([C@H]3C[C@@H]3c3ccccc3)c2=O)C1	SmoAnta	0.166	0.934
42	CHEMBL474507	Cc1csc(CN[C@H]2Cc3ccc(NC(=O)c4cccc(C)c4-c4ccc(C(F)(F)F)cc4)cc3C2)n1	SmoAnta	0.209	0.866
43	CHEMBL1085503	Cc1ccc(-c2nnc(N3CCN(C(=O)c4ccccc4)CC3)c3ccccc23)cc1	SmoAnta	0.108	0.941
44	CHEMBL1209190	C[C@@H]1CN(C(=O)c2ccccc2)CCN1c1nnc(-c2ccc(CC(N)=O)cc2)c2ccccc12	SmoAnta	0.102	0.955
45	CHEMBL2059859	Cc1ccc(-c2nc3ccccc3[nH]2)cc1NC(=O)c1ccc(OCc2ccccn2)cc1	SmoAnta	0.126	0.917
46	CHEMBL2059866	Cc1ccc(-c2cn(C)cn2)cc1NC(=O)c1ccc(OCc2ccccn2)cc1	SmoAnta	0.097	0.934
47	CHEMBL561533	CCCC(CCC)C(=O)NCc1ccc2c(cnn2-c2ccccc2OC)c1	SmoAnta	0.21	0.919
48	CHEMBL2031290	COc1cc(C(=O)N=C(N)Nc2ccc(C)c(NC(=O)c3ccc(-c4ccccc4)cc3)c2)cc(OC)c1OC	SmoAnta	0.131	0.938
49	CHEMBL2043430	O=C(Nc1cccc(-c2nc3ccccc3[nH]2)c1)c1ccc2c(c1)OCCO2	SmoAnta	0.116	0.921
50	CHEMBL1086048	C[C@H]1CN(C(=O)c2ccccc2)CCN1c1nnc(-c2ccccc2)c2ccccc12	SmoAnta	0.13	0.941
51	CHEMBL1086045	C[C@H]1CN(c2nnc(-c3ccccc3)c3ccccc23)CCN1C(=O)c1ccccc1	SmoAnta	0.133	0.946
52	CHEMBL1084737	C[C@@H]1CN(C(=O)c2ccccc2)CCN1c1nnc(-c2ccc(N(C)C)cc2)c2ccccc12	SmoAnta	0.103	0.957
53	CHEMBL1084837	O=C(c1ccccc1)N1CCN(c2nnc(-c3ccc(Cl)cc3)c3ccccc23)CC1	SmoAnta	0.105	0.939
54	CHEMBL3604610	CC(=O)N=c1[nH]c2ccc(-c3cnc(Cl)c(NC(=O)c4ccccc4)c3)cc2s1	SmoAnta	0.131	0.904
55	CHEMBL471672	Cc1ccc(CN[C@H]2Cc3ccc(NC(=O)c4cccc(C)c4-c4ccc(C(F)(F)F)cc4)cc3C2)o1	SmoAnta	0.219	0.858
56	CHEMBL1824915	O=C(Nc1ccc(-c2cccc(-c3ccccc3)c2)cc1)c1ccccc1Cl	SmoAnta	0.099	0.942
57	CHEMBL2059867	Cc1nc(-c2ccc(C)c(NC(=O)c3ccc(OCc4ccccn4)cc3)c2)c[nH]1	SmoAnta	0.103	0.93
58	CHEMBL1083285	C[C@H]1CN(c2nnc(-c3ccc(Cl)cc3)c3ccccc23)CCN1C(=O)c1ccccc1	SmoAnta	0.115	0.944
59	CHEMBL1813111	O=C(N=c1cc[nH]n1-c1cccc(F)c1)N1CCn2c(c(O)n([C@H]3C[C@@H]3c3ccccc3)c2=O)C1	SmoAnta	0.127	0.932

60	CHEMBL474503	Cc1cccc(C(=O)Nc2ccc3c(c2)C[C@@H](NCc2cccs2)C3)c1-c1ccc(C(F)(F)F)cc1	SmoAnta	0.214	0.855
61	CHEMBL4441529	Cc1ccc(=NCc2ccnn2-c2nnc(N3CCC(C(=O)N[C@H]4CCCC[C@H]4C)CC3)s2)[nH]c1	SmoAnta	0.135	0.94
62	CHEMBL1084731	CC(C)c1ccc(-c2nnc(N3CCN(C(=O)c4ccccc4)C[C@H]3C)c3ccccc23)cc1	SmoAnta	0.107	0.953
63	CHEMBL1824916	O=C(Nc1ccc(-c2cc(-c3ccccc3)ccn2)cc1)c1ccccc1Cl	SmoAnta	0.095	0.94
64	CHEMBL1209314	C[C@@H]1CN(C(=O)c2ccccc2)CCN1c1nnc(-n2ccc3ccccc32)c2ccccc12	SmoAnta	0.137	0.951
65	CHEMBL473896	Cc1cccc(C(=O)Nc2ccc3c(c2)C[C@@H](NCc2ccccc2)C3)c1-c1ccc(C(F)(F)F)cc1	SmoAnta	0.216	0.86
66	CHEMBL4567678	Cc1ccc(=NCc2ccnn2-<br>c2nnc(N3CCC(C(=O)N[C@@H]4CCCC[C@@H]4C)CC3)s2)[nH]c1	SmoAnta	0.136	0.938
67	CHEMBL1209251	C[C@@H]1CN(C(=O)c2ccccc2)CCN1c1nnc(-n2cccc2)c2ccccc12	SmoAnta	0.136	0.935
68	CHEMBL2059871	Cc1ccc(-c2cc(C(F)(F)F)[nH]n2)cc1NC(=O)c1ccc(OCc2ccccn2)cc1	SmoAnta	0.104	0.923
69	CHEMBL2059868	Cc1ccc(-c2[nH]cnc2C)cc1NC(=O)c1ccc(OCc2ccccn2)cc1	SmoAnta	0.1	0.942
70	CHEMBL4472296	C[C@]1(c2ccc(Cl)cc2Cl)OC[C@@H](COc2ccc(N3CCN(c4ccc(NC(=O)NN)cc4)CC3)c<br>c2)O1	SmoAnta	0.074	0.965
71	CHEMBL1083104	O=C(c1nccs1)N1CCN(c2nnc(-c3ccccc3)c3ccccc23)CC1	SmoAnta	0.12	0.929
72	CHEMBL4159710	COc1cc(C(=O)N=C(N)Nc2cccc(NC(=O)c3ccc(-c4ccccc4)cc3)c2)cc(OC)c1OC	SmoAnta	0.116	0.952
73	CHEMBL1084733	C[C@@H]1CN(C(=O)c2ccccc2)CCN1c1nnc(-c2ccc(C(C)(C)C)cc2)c2ccccc12	SmoAnta	0.101	0.96
74	CHEMBL3604612	CC(=O)N=c1[nH]c2ccc(-c3cnc(Cl)c(NC(=O)c4cccc(Cl)c4)c3)cc2s1	SmoAnta	0.151	0.898
75	CHEMBL474893	Cc1cccc(C(=O)Nc2ccc3c(c2)C[C@@H](NCc2nccs2)C3)c1-c1ccc(C(F)(F)F)cc1	SmoAnta	0.212	0.858
76	CHEMBL519219	OC1(c2ccc(N3CCN(c4nnc(Cc5ccccc5)c5ccccc45)CC3)nc2)CC1	SmoAnta	0.099	0.958
77	CHEMBL1083739	O=C(c1ccccc1)N1CCN(c2nnc(-c3ccc(F)cc3)c3ccccc23)CC1	SmoAnta	0.116	0.937
78	CHEMBL1083605	C[C@H]1CN(c2nnc(-c3ccc(F)cc3)c3ccccc23)CCN1C(=O)c1ccccc1	SmoAnta	0.121	0.942
79	CHEMBL1813105	O=C(Nc1cccnc1-c1ccccc1)N1CCn2c(c(O)n([C@H]3C[C@@H]3c3ccccc3)c2=O)C1	SmoAnta	0.134	0.948
80	CHEMBL1209381	C[C@@H]1CN(C(=O)c2ccccc2)CCN1c1nnc(-c2ccccc2)c2cnccc12	SmoAnta	0.129	0.943
81	CHEMBL1209074	C[C@H]1CN(C(=O)c2ccccc2)CCN1c1nnc(-c2ccc(F)cc2)c2ccccc12	SmoAnta	0.113	0.943
82	CHEMBL2059870	Cc1ccc(-c2c(C)nn(C)c2C)cc1NC(=O)c1ccc(OCc2ccccn2)cc1	SmoAnta	0.096	0.951
83	CHEMBL1083103	O=C(c1ccco1)N1CCN(c2nnc(-c3ccccc3)c3ccccc23)CC1	SmoAnta	0.13	0.934
84	CHEMBL1086284	C[C@@H]1CN(c2nnc(-c3ccccc3)c3ccccc23)CCN1C(=O)c1ccccc1	SmoAnta	0.131	0.941
85	CHEMBL2031277	COc1cc(C(=O)NC(=O)Nc2ccc(C)c(NC(=O)c3ccc(-c4ccccc4)cc3)c2)cc(OC)c1OC	SmoAnta	0.122	0.92
86	CHEMBL1824904	O=C(Nc1ccc(NC(=O)c2ccccc2Cl)cc1)c1ccccc1	SmoAnta	0.07	0.962
87	CHEMBL1813114	C[C@@]12CN(C(=O)N=c3cc[nH]n3-<br>c3ccccc3)CCN1C(=O)N([C@H]1C[C@@H]1c1ccccc1)C2=O	SmoAnta	0.149	0.934
88	CHEMBL2031288	COc1cc(C(=O)N=C(N)Nc2ccc(C)c(NC(=O)c3ccc(-c4ccc(F)cc4)cc3)c2)cc(OC)c1OC	SmoAnta	0.114	0.954
89	CHEMBL1824905	O=C(Nc1ccc(CC(O)c2ccccc2)cc1)c1ccccc1Cl	SmoAnta	0.077	0.95
90	CHEMBL514764	Cc1cccc(C(=O)Nc2ccc3c(c2)C[C@@H](NCc2ccco2)C3)c1-c1ccc(C(F)(F)F)cc1	SmoAnta	0.222	0.844
91	CHEMBL495877	CN(C)Cc1ccc(N2CCN(c3nnc(Cc4ccccc4)c4ccccc34)CC2)nc1	SmoAnta	0.107	0.949
92	CHEMBL1813109	O=C(N=c1cc[nH]n1-<br>c1ccccc1)N1CCn2c(c(O)n([C@H]3C[C@@H]3c3ccccc3)c2=O)C1	SmoAnta	0.111	0.932
93	CHEMBL1083738	COc1ccc(-c2nnc(N3CCN(C(=O)c4ccccc4)CC3)c3ccccc23)cc1	SmoAnta	0.108	0.941
94	CHEMBL1082387	Cc1ccccc1-c1nnc(N2CCN(C(=O)c3ccccc3)CC2)c2ccccc12	SmoAnta	0.129	0.946
95	CHEMBL1209380	C[C@@H]1CN(C(=O)c2ccccc2)CCN1c1nnc(-c2ccccc2)c2cccnc12	SmoAnta	0.129	0.941
96	CHEMBL1813107	N#Cc1cccc(-<br>c2ncccc2NC(=O)N2CCn3c(c(O)n([C@H]4C[C@@H]4c4ccccc4)c3=O)C2)c1	SmoAnta	0.161	0.939
97	CHEMBL1084836	O=C(c1ccccc1)N1CCN(c2nnc(-c3ccc(Cl)c(Cl)c3)c3ccccc23)CC1	SmoAnta	0.105	0.951
98	CHEMBL497238	C[C@H]1CN(c2ccc(C#N)cn2)CCN1c1nnc(Cc2ccccc2)c2ccccc12	SmoAnta	0.123	0.962
99	CHEMBL1824909	O=C(Nc1ccc(-c2nc(-c3ccccc3)c(C(F)(F)F)o2)cc1)c1ccccc1Cl	SmoAnta	0.101	0.95
100	CHEMBL497437	C[C@@H]1CN(c2nnc(Cc3ccccc3)c3ccccc23)CCN1c1ccc(C#N)cn1	SmoAnta	0.143	0.953

101	CHEMBL520858	CC(O)(CO)c1ccc(N2CCN(c3nnc(Cc4ccccc4)c4ccccc34)CC2)nc1	SmoAnta	0.094	0.97
102	CHEMBL3818447	Cn1c(-c2cc(N3CCc4sc(C(=O)N5CCOCC5)cc4C3)ccc2Cl)nc2ccccc21	SmoAnta	0.136	0.948
103	CHEMBL1813110	O=C(N=c1cc[nH]n1-c1cccc(Cl)c1)N1CCn2c(c(O)n([C@H]3C[C@@H]3c3ccccc3)c2=O)C1	SmoAnta	0.144	0.913
104	CHEMBL1209132	C[C@@H]1CN(C(=O)c2ccccc2)CCN1c1nnc(-c2ccc(C(N)=O)cc2)c2ccccc12	SmoAnta	0.112	0.948
105	CHEMBL1082791	O=C(c1ccccc1)N1CCN(c2nnc(-c3ccccc3)c3ccccc23)CC1	SmoAnta	0.121	0.937
106	CHEMBL3741651	O=C(NC(=O)c1ccc(OCc2ccccc2F)cc1)Nc1ccc(Cl)c(-c2nc3ccccc3[nH]2)c1	SmoAnta	0.099	0.937
107	CHEMBL1813113	C[C@@]12CN(C(=O)Nc3ccccc3-c3ccccc3)CCN1C(=O)N([C@H]1C[C@@H]1c1ccccc1)C2=O	SmoAnta	0.154	0.94
108	CHEMBL2160075	Clc1ccc(N=c2[nH]ccc3ccccc23)cc1-c1nc(-c2ccccc2)c[nH]1	SmoAnta	0.162	0.893
109	CHEMBL1083740	COc1cccc(-c2nnc(N3CCN(C(=O)c4ccccc4)CC3)c3ccccc23)c1	SmoAnta	0.118	0.943
110	CHEMBL495608	N#Cc1ccc(Cc2nnc(N3CCN(c4ccc(C#N)cn4)CC3)c3ccccc23)cc1	SmoAnta	0.121	0.961
111	CHEMBL496604	N#Cc1ccc(N2CCN(c3nnc(Cc4ccc(Cl)cc4)c4ccccc34)CC2)nc1	SmoAnta	0.126	0.956
112	CHEMBL1082396	O=C(c1ccccc1)N1CCN(c2nnc(-c3ccccc3Cl)c3ccccc23)CC1	SmoAnta	0.138	0.945
113	CHEMBL1813096	O=C(Nc1ccccc1-c1ccccc1)N1CCn2c(c(O)n([C@H]3C[C@@H]3c3ccccc3)c2=O)C1	SmoAnta	0.127	0.952
114	CHEMBL2059875	Cc1ccc(-c2ccccn2)cc1NC(=O)c1ccc(OCc2ccccn2)cc1	SmoAnta	0.107	0.937
115	CHEMBL1209133	C[C@@H]1CN(C(=O)c2ccccc2)CCN1c1nnc(-c2ccc(N3CCOCC3)cc2)c2ccccc12	SmoAnta	0.081	0.967
116	CHEMBL1813108	O=C(Nc1cccnc1-c1ccsc1)N1CCn2c(c(O)n([C@H]3C[C@@H]3c3ccccc3)c2=O)C1	SmoAnta	0.133	0.955
117	CHEMBL2059869	Cc1ccc(-c2cn(C)c(C)n2)cc1NC(=O)c1ccc(OCc2ccccn2)cc1	SmoAnta	0.098	0.922
118	CHEMBL1084301	O=C(c1cccs1)N1CCN(c2nnc(-c3ccccc3)c3ccccc23)CC1	SmoAnta	0.124	0.934
119	CHEMBL1209248	C[C@@H]1CN(C(=O)c2ccccc2)CCN1c1nnc(-c2ccncc2)c2ccccc12	SmoAnta	0.128	0.945
120	CHEMBL1824917	O=C(Nc1ccc(-c2ccnc(-c3ccccc3)n2)cc1)c1ccccc1Cl	SmoAnta	0.105	0.91
121	CHEMBL1824908	O=C(Nc1ccc(-c2nc(-c3ccccc3)cs2)cc1)c1ccccc1Cl	SmoAnta	0.086	0.931
122	CHEMBL1824918	O=C(Nc1ccc(-c2cncc(-c3ccccc3)c2)cc1)c1ccccc1Cl	SmoAnta	0.098	0.942
123	CHEMBL2031263	COc1cc(C(=O)NC(=S)Nc2ccc(C)c(NC(=O)c3ccc(-c4ccc(F)cc4)cc3)c2)cc(OC)c1OC	SmoAnta	0.151	0.916
124	CHEMBL4562943	C#CCOc1ccc(-n2c(O)c(C)n(Cc3ccc(N=[N+]=[N-])cc3)c2=O)cc1	SmoAnta	0.183	0.915
125	CHEMBL1824919	O=C(Nc1ccc(-c2cccc(-c3ccccc3)n2)cc1)c1ccccc1Cl	SmoAnta	0.11	0.91
126	CHEMBL2031287	COc1cc(C(=O)N=C(N)Nc2cccc(NC(=O)c3ccc(-c4ccc(F)cc4)cc3)c2)cc(OC)c1OC	SmoAnta	0.106	0.953
127	CHEMBL4632598	CN(C(=O)c1ccc(F)cc1C(F)(F)F)C1CCN(C(=O)c2ccccc2Nc2ccccc2)CC1	SmoAnta	0.175	0.956
128	CHEMBL523255	CC(=O)NCc1ccc(N2CCN(c3nnc(Cc4ccccc4)c4ccccc34)CC2)nc1	SmoAnta	0.095	0.968
129	CHEMBL1824920	O=C(Nc1ccc(-c2nccc(-c3ccccc3)n2)cc1)c1ccccc1Cl	SmoAnta	0.1	0.922
130	CHEMBL485869	CC(O)c1ccc(N2CCN(c3nnc(Cc4ccccc4)c4ccccc34)CC2)nc1	SmoAnta	0.105	0.955
131	CHEMBL3126707	CS(=O)(=O)c1ccc(C(=O)Nc2ccc(Cl)c(C(=O)Nc3cccc(O)c3)c2)c(Cl)c1	SmoAnta	0.133	0.917
132	CHEMBL563928	CCCC(CCC)C(=O)NCc1ccc2c(cnn2-c2ccc(OC)cc2)c1	SmoAnta	0.186	0.922
133	CHEMBL1824910	O=C(Nc1ccc(-c2ncc(-c3ccccc3)o2)cc1)c1ccccc1Cl	SmoAnta	0.07	0.944
134	CHEMBL2031268	COc1cc(C(=O)NC(=S)Nc2ccc(C)c(NC(=O)c3ccc(-c4ccccc4)cc3)c2)cc(OC)c1OC	SmoAnta	0.159	0.901
135	CHEMBL1813104	CS(=O)(=O)c1cc(Cl)c(NC(=O)N2CCn3c(c(O)n([C@H]4C[C@@H]4c4ccccc4)c3=O)C2)c(Cl)c1	SmoAnta	0.083	0.984
136	CHEMBL2059872	Cc1ccc(-c2ncc(CN3CCOCC3)s2)cc1NC(=O)c1ccc(OCc2ccccn2)cc1	SmoAnta	0.116	0.935
137	CHEMBL3740447	O=C(NC(=O)c1ccc(OCc2ccc(F)cc2)cc1)Nc1ccc(Cl)c(-c2nc3ccccc3[nH]2)c1	SmoAnta	0.093	0.945
138	CHEMBL1824911	Cc1oc(-c2ccc(NC(=O)c3ccccc3Cl)cc2)nc1-c1ccccc1	SmoAnta	0.076	0.94
139	CHEMBL2059873	Cc1ccc(-c2nccs2)cc1NC(=O)c1ccc(OCc2ccccn2)cc1	SmoAnta	0.087	0.942
140	CHEMBL495875	NC(=O)c1ccc(N2CCN(c3nnc(Cc4ccccc4)c4ccccc34)CC2)nc1	SmoAnta	0.102	0.96
141	CHEMBL1824921	O=C(Nc1ccc(-c2cc(-c3ccccc3)ncn2)cc1)c1ccccc1Cl	SmoAnta	0.088	0.94
142	CHEMBL1824906	O=C(Nc1ccc(C=Cc2ccccc2)cc1)c1ccccc1Cl	SmoAnta	0.063	0.947

143	CHEMBL521900	N#Cc1ccc(N2CCN(c3nnc(Cc4cccc(Cl)c4)c4ccccc34)CC2)nc1	SmoAnta	0.132	0.942
144	CHEMBL3819559	Cn1c(-c2cc(N3CCc4sc(C(=O)Nc5cccnc5)cc4C3)ccc2Cl)nc2ccccc21	SmoAnta	0.117	0.967
145	CHEMBL1209250	C[C@@H]1CN(C(=O)c2ccccc2)CCN1c1nnc(-c2ccccn2)c2ccccc12	SmoAnta	0.134	0.941
146	CHEMBL142972	Cc1cccc(C(=O)Nc2ccc3c(c2)C[C@@H](NCc2ccccn2)C3)c1-c1ccc(C(F)(F)F)cc1	SmoAnta	0.22	0.86
147	CHEMBL4441183	Cc1c(O)n(-c2ccc(N=[N+]=[N-])cc2)c(=O)n1Cc1ccccc1	SmoAnta	0.169	0.926
148	CHEMBL497436	C[C@H]1CN(c2nnc(Cc3ccccc3)c3ccccc23)CCN1c1ccc(C#N)cn1	SmoAnta	0.127	0.951
149	CHEMBL4586057	Cc1c(O)n(-c2ccc(Cl)cc2)c(=O)n1Cc1ccccc1	SmoAnta	0.18	0.939
150	CHEMBL2057337	Cc1ccc(-c2ncnc3[nH]cnc23)cc1NC(=O)c1ccc(OCc2ccccn2)cc1	SmoAnta	0.103	0.94
151	CHEMBL1824912	O=C(Nc1ccc(-c2nc(-c3ccccc3)co2)cc1)c1ccccc1Cl	SmoAnta	0.067	0.945
152	CHEMBL254129	CC1=C2C[C@H]3[C@@H](CC=C4C[C@@H](O)CC[C@@]43C)[C@@H]2CC[C@]1 2O[C@@H]1C[C@H](C)CN[C@H]1[C@H]2C	SmoAnta	0.13	0.965
153	CHEMBL1209379	C[C@@H]1CN(C(=O)c2ccccc2)CCN1c1nnc(-c2ccccc2)c2ncccc12	SmoAnta	0.128	0.934
154	CHEMBL2031289	COc1cc(C(=O)N=C(N)Nc2ccc(Cl)c(NC(=O)c3ccc(-c4ccccc4)cc3)c2)cc(OC)c1OC	SmoAnta	0.145	0.954
155	CHEMBL562270	CCCC(CCC)C(=O)NCc1ccc2c(cnn2-c2cccc(C)c2)c1	SmoAnta	0.194	0.931
156	CHEMBL2031283	COc1cc(C(=O)N=C(N)Nc2cccc(-c3cc4ccccc4[nH]3)c2)cc(OC)c1OC	SmoAnta	0.148	0.893
157	CHEMBL497534	N#Cc1ccc(N2CCN(c3nnc(Cc4ccccc4)c4ccccc34)CC2)nc1	SmoAnta	0.112	0.972
158	CHEMBL496603	N#Cc1ccc(N2CCN(c3nnc(Cc4ccc(F)cc4)c4ccccc34)CC2)nc1	SmoAnta	0.116	0.963
159	CHEMBL4282915	CN(Cc1ccc(F)cc1C(F)(F)F)C1CCN(c2nnc(-c3ccnn3C)c3ccccc23)CC1	SmoAnta	0.116	0.946
160	CHEMBL3972086	Cc1cc(-c2ncc(CNC(=O)c3ccc4c(c3)[nH]c3ccccc34)cc2F)ccn1	SmoAnta	0.163	0.931
161	CHEMBL521558	FC(F)(F)c1ccc(N2CCN(c3nnc(Cc4ccccc4)c4ccccc34)CC2)nc1	SmoAnta	0.093	0.962
162	CHEMBL1813103	CN(C)C(=O)c1cc(Cl)c(NC(=O)N2CCn3c(c(O)n([C@H]4C[C@@H]4c4ccccc4)c3=O) C2)c(Cl)c1	SmoAnta	0.08	0.984
163	CHEMBL2031077	COc1cc(C(=O)NC(=O)Nc2ccc(Cl)c(NC(=O)c3ccc(-c4ccccc4)cc3)c2)cc(OC)c1OC	SmoAnta	0.127	0.924
164	CHEMBL1209249	C[C@@H]1CN(C(=O)c2ccccc2)CCN1c1nnc(-c2cccnc2)c2ccccc12	SmoAnta	0.131	0.938
165	CHEMBL1824907	O=C(Cc1ccc(NC(=O)c2ccccc2Cl)cc1)c1ccccc1	SmoAnta	0.076	0.975
166	CHEMBL474302	Cc1cccc(C(=O)Nc2ccc3c(c2)C[C@@H](Nc2ccccc2)C3)c1-c1ccc(C(F)(F)F)cc1	SmoAnta	0.23	0.838
167	CHEMBL497639	C[C@@H]1CN(c2ccc(C#N)cn2)CCN1c1nnc(Cc2ccccc2)c2ccccc12	SmoAnta	0.114	0.964
168	CHEMBL3262641	Cc1cnc(-c2cc(N3CCn4cc(C(=O)Nc5cccc(F)c5)nc4C3)ncc2Cl)c(C)c1	SmoAnta	0.09	0.955
169	CHEMBL2031098	COc1cc(C(=O)NC(=S)Nc2cccc(NC(=O)c3ccc(-c4ccccc4)cc3)c2)cc(OC)c1OC	SmoAnta	0.137	0.919
170	CHEMBL1824913	O=C(Nc1ccc(-c2nnc(-c3ccccc3)o2)cc1)c1ccccc1Cl	SmoAnta	0.067	0.946
171	CHEMBL495876	NS(=O)(=O)c1ccc(N2CCN(c3nnc(Cc4ccccc4)c4ccccc34)CC2)nc1	SmoAnta	0.089	0.964
172	CHEMBL4450586	CC(=O)c1cccc(C(=O)Nc2ccc(N3CCN(c4ccc(OC[C@@H]5CO[C@](C)(c6ccc(Cl)cc6C l)O5)cc4)CC3)cc2)c1	SmoAnta	0.053	0.98
173	CHEMBL1082790	CC(=O)N1CCN(c2nnc(-c3ccccc3)c3ccccc23)CC1	SmoAnta	0.17	0.901
174	CHEMBL3818034	Cn1c(-c2cc(N3CCc4sc(C(=O)N5CCN(c6ncccn6)CC5)cc4C3)ccc2Cl)nc2ccccc21	SmoAnta	0.113	0.95
175	CHEMBL4441914	Cc1c(O)n(-c2ccc(Cl)cc2)c(=O)n1CCc1ccccc1	SmoAnta	0.124	0.898
176	CHEMBL1824922	O=C(Nc1ccc(-c2ccnc(-c3ccccc3)c2)cc1)c1ccccc1Cl	SmoAnta	0.095	0.942
177	CHEMBL1824914	O=C(Nc1ccc(-c2coc(-c3ccccc3)n2)cc1)c1ccccc1Cl	SmoAnta	0.067	0.945
178	CHEMBL471881	COc1ccc(CN[C@H]2Cc3ccc(NC(=O)c4cccc(C)c4-c4ccc(C(F)(F)F)cc4)cc3C2)cc1	SmoAnta	0.195	0.883
179	CHEMBL1082967	C[C@H]1CN(c2nnc(-c3ccncc3)c3ccccc23)CCN1C(=O)c1ccccc1	SmoAnta	0.142	0.933
180	CHEMBL3949191	Cc1c(Cc2ccccc2)nnc(N2CCC(O)(c3ccc(C(C)(C)O)cn3)CC2)c1C	SmoAnta	0.097	0.971
181	CHEMBL3966039	Cc1c(-c2ccc(C(F)(F)F)cc2)nnc(N2CCN(c3cnc(C(=O)O)cn3)[C@H](C)C2)c1C	SmoAnta	0.058	0.982
182	CHEMBL3965479	Cc1c(Cc2ccccc2)nnc(N2CCN(c3cnc(C(=O)N4CCNC(=O)C4)cn3)[C@H](C)C2)c1C	SmoAnta	0.078	0.973
183	CHEMBL3982279	Cc1c(Cc2ccccc2)nnc(N2CCN(C(=O)Nc3ccccc3)CC2)c1C	SmoAnta	0.092	0.974
184	CHEMBL3953149	Cc1c(C(=O)c2ccccc2)nnc(N2CCN(c3cnc(C(C)(C)O)cn3)[C@H](C)C2)c1C	SmoAnta	0.097	0.946

185	CHEMBL3965793	COC(=O)c1cnc(N2CCN(c3nnc(Cc4ccncc4)c(C)c3C)C[C@H]2C)cn1	SmoAnta	0.084	0.977
186	CHEMBL3978301	CC(=O)N1CCN(C(=O)c2cnc(N3CCN(c4nnc(-c5ccc(C(F)(F)F)cc5)c(C)c4C)C[C@H]3C)cn2)CC1	SmoAnta	0.06	0.976
187	CHEMBL3948598	Cc1c(Cc2ccccc2)nnc(N2CCN(c3ccc(C(F)(F)F)cn3)CC2)c1C	SmoAnta	0.074	0.982
188	CHEMBL1813102	N#Cc1cc(Cl)c(NC(=O)N2CCn3c(c(O)n([C@H]4C[C@@H]4c4ccccc4)c3=O)C2)c(Cl)c1	SmoAnta	0.09	0.98
189	CHEMBL3959511	CCCCOC(=O)c1cnc(N2CCN(c3nnc(-c4ccc(C(C)C)cc4)c(C)c3C)CC2)cc1C(F)(F)F	SmoAnta	0.073	0.966
190	CHEMBL3919707	Cc1c(Cc2ccccc2)nnc(N2CCN(c3cnc(C(=O)N4C[C@H](C)O[C@H](C)C4)cn3)[C@H](C)C2)c1C	SmoAnta	0.079	0.978
191	CHEMBL3934557	Cc1c(Cc2ccccc2)nnc(N2CCN(c3cnc(C(=O)N(C)CCO)cn3)[C@H](C)C2)c1C	SmoAnta	0.083	0.977
192	CHEMBL3891390	Cc1c(Cc2ccccc2)nnc(N2CCN(c3cnc(C(=O)NCCCO)cn3)[C@H](C)C2)c1C	SmoAnta	0.079	0.976
193	CHEMBL2031245	COc1cc(C(=O)NC(=S)Nc2cccc(NC(=O)c3ccc(-c4ccc(F)cc4)cc3)c2)cc(OC)c1OC	SmoAnta	0.138	0.919
194	CHEMBL561735	CCCC(CCC)C(=O)NCc1ccc2c(cnn2-c2cccc(OC)c2)c1	SmoAnta	0.199	0.931
195	CHEMBL3941852	Cc1c(Cc2ccccc2)nnc(N2CCN(c3cnc(C(=O)N4CCN(C(C)C)CC4)cn3)[C@H](C)C2)c1C	SmoAnta	0.087	0.969
196	CHEMBL3915100	Cc1c(Cc2ccccc2)nnc(N2CCN(c3cnc(C(=O)NC4CCC(O)CC4)cn3)[C@H](C)C2)c1C	SmoAnta	0.063	0.981
197	CHEMBL3126705	Cc1cccc(NC(=O)c2cc(NC(=O)c3ccc(S(C)(=O)=O)cc3Cl)ccc2Cl)c1C	SmoAnta	0.133	0.897
198	CHEMBL3986419	COC(=O)c1cnc(N2CCN(c3n[nH]c(=Nc4ccccc4)c(C)c3C)C[C@H]2C)cn1	SmoAnta	0.081	0.966
199	CHEMBL3927351	Cc1c(-c2ccc(F)c(C#N)c2)nnc(N2CCN(c3ccc(C(F)(F)F)cn3)CC2)c1C	SmoAnta	0.058	0.979
200	CHEMBL3894764	Cc1c(Cc2ccccc2)nnc(N2CCN(C(=O)Oc3ccccc3)CC2)c1C	SmoAnta	0.096	0.969
201	CHEMBL3957159	CCCCOC(=O)c1cnc(N2CCN(c3nnc(-c4ccc(F)nc4)c(C)c3C)C[C@H]2C)cc1C(F)(F)F	SmoAnta	0.086	0.968
202	CHEMBL3965473	Fc1cccc(Cc2nnc(N3CCN(c4ccc(C(F)(F)F)cn4)CC3)c3c2CCC3)c1	SmoAnta	0.091	0.973
203	CHEMBL3897743	Cc1c(Cc2ccccc2)nnc(N2CCN(c3cnc(C(=O)O)cn3)[C@H](C)C2)c1C	SmoAnta	0.098	0.976
204	CHEMBL3929514	Cc1c(Cc2ccccc2)nnc(N2CCN(c3cnc(C(=O)N4CCC(CCO)CC4)cn3)[C@H](C)C2)c1C	SmoAnta	0.085	0.958
205	CHEMBL3967092	Cc1c(Cc2ccccc2)nnc(N2CCN(c3cnc(C(=O)NCCc4ccccn4)cn3)[C@H](C)C2)c1C	SmoAnta	0.081	0.976
206	CHEMBL3979837	Cc1c(Cc2ccccc2)nnc(N2CCN(C(=O)NCc3ccccc3)CC2)c1C	SmoAnta	0.108	0.971
207	CHEMBL3959845	CCCCOC(=O)c1cnc(N2CCN(c3nnc(-c4cccnc4)c(C)c3C)C[C@H]2C)cc1C(F)(F)F	SmoAnta	0.085	0.967
208	CHEMBL3927593	Cc1c(-c2ccc(C(F)(F)F)cc2)nnc(N2CCN(c3cnc(C(=O)N4CCN(S(C)(=O)=O)CC4)cn3)[C@H](C)C2)c1C	SmoAnta	0.06	0.976
209	CHEMBL3912445	Cc1c(Cc2ccccc2)nnc(N2CCN(c3cnc(C(=O)NC4CC4)cn3)[C@H](C)C2)c1C	SmoAnta	0.076	0.98
210	CHEMBL3955346	Cc1ccccc1Cc1nnc(N2CCN(c3ccc(C(F)(F)F)cn3)CC2)c2c1CCC2	SmoAnta	0.08	0.977
211	CHEMBL3986486	Cc1c(Cc2ccccc2)nnc(N2CCN(c3cnc(C(=O)NCCN4CCOCC4)cn3)[C@H](C)C2)c1C	SmoAnta	0.072	0.981
212	CHEMBL3977460	COC(=O)c1cnc(N2CCN(c3nnc(-c4ccc(F)c(C#N)c4)c(C)c3C)C[C@H]2C)cn1	SmoAnta	0.073	0.964
213	CHEMBL3958236	Cc1c(Cc2ccccc2)nnc(N2CCC(c3nc4cccnc4[nH]3)CC2)c1C	SmoAnta	0.087	0.984
214	CHEMBL3957247	Cc1c(Cc2ccccc2)nnc(N2CCN(c3cnc(C(=O)N4CCN(C)CC4)cn3)[C@H](C)C2)c1C	SmoAnta	0.088	0.963
215	CHEMBL3948829	Cc1c(Cc2ccc(F)cc2)nnc(N2CCN(c3ccc(C(C)(C)C)cc3)CC2)c1C	SmoAnta	0.085	0.975
216	CHEMBL3973612	FC(F)(F)c1ccc(N2CCN(c3nnc(Cc4ccccc4Cl)c4c3CCC4)CC2)nc1	SmoAnta	0.09	0.97
217	CHEMBL3909290	Cc1c(N2CCN(c3cnc(C(=O)Nc4ccccc4)cn3)[C@H](C)C2)n[nH]c(=Nc2ccccc2)c1C	SmoAnta	0.054	0.984
218	CHEMBL3958040	Cc1c(Cc2ccccc2)nnc(C2CCN(c3ccc(C(F)(F)F)cn3)CC2)c1C	SmoAnta	0.072	0.983
219	CHEMBL3930632	CC(C)(C)c1ccc(N2CCN(c3nnc(Cc4ccc(F)cc4)c4c3CCCC4)CC2)cc1	SmoAnta	0.107	0.975
220	CHEMBL3906764	COC(=O)c1cnc(N2CCC(c3nnc(Cc4ccccc4)c(C)c3C)CC2)cn1	SmoAnta	0.072	0.979
221	CHEMBL3905798	Cc1c(Cc2ccccc2)nnc(N2CCN(c3cnc(C(=O)NCCN4CCCC4)cn3)[C@H](C)C2)c1C	SmoAnta	0.074	0.981
222	CHEMBL3980924	Fc1ccc(Cc2nnc(N3CCN(c4ccc(C(F)(F)F)cn4)CC3)c3c2CCCC3)cc1	SmoAnta	0.101	0.973
223	CHEMBL3930063	Cc1c(-c2ccncc2)nnc(N2CCN(c3ccc(C(F)(F)F)cn3)CC2)c1C	SmoAnta	0.055	0.977
224	CHEMBL3952417	CC(=O)c1cnc(N2CCN(c3nnc(Cc4ccccc4)c(C)c3C)C[C@H]2C)nc1C(F)(F)F	SmoAnta	0.106	0.962
225	CHEMBL3942299	Cc1c(Cc2ccc(F)cc2)nnc(N2CCN(c3ccc(C(F)(F)F)cn3)CC2)c1C	SmoAnta	0.076	0.976

226	CHEMBL3942727	Cc1c(-c2ccc(F)cc2)nnc(N2CCN(c3ccc(C(F)(F)F)cn3)CC2)c1C	SmoAnta	0.047	0.983
227	CHEMBL3950710	COC(=O)c1cnc(N2CCN(c3nnc(-c4ccc(OC)cc4)c(C)c3C)CC2)nc1C(F)(F)F	SmoAnta	0.065	0.977
228	CHEMBL3944996	Fc1ccc(Cc2nnc(N3CCN(c4ccc(C(F)(F)F)cn4)CC3)c3c2CCC3)cc1	SmoAnta	0.093	0.974
229	CHEMBL3905821	CCCCOC(=O)c1cnc(N2CCN(c3nnc(-c4ccncc4)c(C)c3C)C[C@H]2C)cc1C(F)(F)F	SmoAnta	0.089	0.969
230	CHEMBL3923355	CCCCOC(=O)c1cnc(N2CCN(c3nnc(-c4cncc(OC)c4)c(C)c3C)CC2)cc1C(F)(F)F	SmoAnta	0.076	0.972
231	CHEMBL3922446	CC[C@@H]1CN(c2nnc(Cc3ccccc3)c(C)c2C)CCN1c1cnc(C(C)=O)cn1	SmoAnta	0.116	0.968
232	CHEMBL3927084	Cc1c(Cc2ccc(F)cc2F)nnc(N2CCN(c3ncc(C(C)(C)O)c(C(F)(F)F)n3)[C@H](C)C2)c1C	SmoAnta	0.108	0.962
233	CHEMBL3911804	Cc1c(Cc2ccccc2)nnc(C2CCN(c3cnc(C(C)(C)O)cn3)CC2)c1C	SmoAnta	0.079	0.976
234	CHEMBL3604621	CC(=O)N=c1[nH]c2ccc(-c3cnc(Cl)c(NC(C#N)c4ccccc4)c3)cc2s1	SmoAnta	0.187	0.903
235	CHEMBL3946402	Cc1c(Cc2ccccc2)nnc(N2CCN(c3ccc(S(=O)(=O)C(F)(F)F)cc3)[C@H](C)C2)c1C	SmoAnta	0.076	0.985
236	CHEMBL3966631	CCN(CCO)C(=O)c1cnc(N2CCN(c3nnc(Cc4ccccc4)c(C)c3C)C[C@H]2C)cn1	SmoAnta	0.084	0.981
237	CHEMBL3950687	Cc1c(Cc2ccccc2)nnc(N2CCN(c3cnc(C(=O)NCC(F)(F)F)cn3)[C@H](C)C2)c1C	SmoAnta	0.072	0.979
238	CHEMBL3903125	Cc1c(Cc2ccccc2)nnc(N2CCN(c3ncc(C(=O)N(C)CCO)c(C(F)(F)F)n3)[C@H](C)C2)c1C	SmoAnta	0.105	0.965
239	CHEMBL3938550	Cc1c(Cc2ccccc2)nnc(N2CCN(c3cncc(C(N)=O)n3)[C@H](C)C2)c1C	SmoAnta	0.132	0.962
240	CHEMBL3933708	CCOC(=O)c1ccc(N2CCN(c3nnc(Cc4ccc(F)cc4)c4c3CCCC4)CC2)nc1	SmoAnta	0.094	0.97
241	CHEMBL3915663	COC(=O)c1cnc(N2CCN(c3nnc(-c4ccc(F)c(Cl)c4)c(C)c3C)CC2)cc1C(F)(F)F	SmoAnta	0.075	0.969
242	CHEMBL3889599	Cc1c(-c2ccc(C(F)(F)F)cc2)nnc(N2CCN(c3cnc(C(=O)N(C)CCc4ccccc4)cn3)[C@H](C)C2)c1C	SmoAnta	0.072	0.973
243	CHEMBL3903694	COC(=O)c1cnc(N2CCN(c3nnc(C(=O)c4ccccc4)c(C)c3C)C[C@H]2C)cn1	SmoAnta	0.071	0.981
244	CHEMBL3953389	COC(=O)c1cnc(N2CCN(c3nnc(-c4cccc(Cl)c4)c(C)c3C)CC2)nc1C(F)(F)F	SmoAnta	0.077	0.979
245	CHEMBL3971766	COC(=O)c1cnc(N2CCN(c3nnc(Cc4ccccc4)c(C)c3C)C[C@H]2C)cn1	SmoAnta	0.085	0.977
246	CHEMBL3983876	COC(=O)c1cnc(N2CCN(c3nnc(Cc4ccc(F)cc4)c(C)c3C)C[C@H]2C)nc1C(F)(F)F	SmoAnta	0.106	0.959
247	CHEMBL3955058	CC(=O)c1cnc(N2CCN(c3nnc(Cc4ccc(F)cc4)c(C)c3C)C[C@H]2C)nc1C(F)(F)F	SmoAnta	0.108	0.959
248	CHEMBL3922623	Cc1c(Cc2ccccc2)nnc(N2CCN(c3cnc(C(=O)NC(C)(C)CO)cn3)[C@H](C)C2)c1C	SmoAnta	0.076	0.982
249	CHEMBL3910022	Fc1ccc(Cc2nnc(N3CCN(c4ccc(C(F)(F)F)cn4)CC3)c3c2CCC3)c(F)c1	SmoAnta	0.092	0.972
250	CHEMBL3906492	Cc1c(N2CCN(c3cnc(C(C)(C)O)cn3)[C@H](C)C2)n[nH]c(=Nc2ccccc2)c1C	SmoAnta	0.076	0.975
251	CHEMBL3986550	Cc1c(Cc2ccccc2)nnc(N2CCN(c3cnc(C(=O)N4CCOCC4)cn3)[C@H](C)C2)c1C	SmoAnta	0.081	0.974
252	CHEMBL3937323	Cc1c(Cc2ccccc2)nnc(N2CCN(c3cnc(C(=O)NCC(C)C)cn3)[C@H](C)C2)c1C	SmoAnta	0.074	0.981
253	CHEMBL3921763	CCOC(=O)c1cnc(N2CCN(c3nnc(Cc4ccccc4)c(C)c3C)C[C@H]2C)nc1C(F)(F)F	SmoAnta	0.094	0.967
254	CHEMBL3899187	FC(F)(F)c1ccc(N2CCN(c3nnc(Cc4ccccc4)c4c3CCC4)CC2)nc1	SmoAnta	0.082	0.973
255	CHEMBL3896503	Cc1c(Cc2ccccc2)nnc(N2CCC(c3nc(Cl)c[nH]3)CC2)c1C	SmoAnta	0.098	0.981
256	CHEMBL3934007	Cc1nc(Cc2nnc(N3CCN(c4ccc(C(F)(F)F)cn4)CC3)c(C)c2C)no1	SmoAnta	0.071	0.979
257	CHEMBL3892588	Cc1c(Cc2ccccc2)nnc(N2CCC(C#N)(c3ccc(C(C)(C)O)cn3)CC2)c1C	SmoAnta	0.111	0.962
258	CHEMBL3967151	Cc1nnc(Cc2nnc(N3CCN(c4ccc(C(F)(F)F)cn4)CC3)c(C)c2C)o1	SmoAnta	0.072	0.981
259	CHEMBL3889851	Cc1c(Cc2ccccc2)nnc(N2CCC(c3nc(C(F)(F)F)c[nH]3)CC2)c1C	SmoAnta	0.088	0.982
260	CHEMBL3902706	CC(=O)c1cnc(N2CCN(c3nnc(Cc4ccccc4)c4c3CCC4)C[C@H]2C)nc1C(F)(F)F	SmoAnta	0.115	0.965
261	CHEMBL3939322	Cc1c(-c2ccc(C(F)(F)F)cc2)nnc(N2CCN(c3cnc(C(=O)N4CCC5(CC4)OCCO5)cn3)[C@H](C)C2)c1C	SmoAnta	0.065	0.968
262	CHEMBL3906243	Cc1c(Cc2ccccc2)nnc(N2CCC(N3Cc4ccccc4C3)CC2)c1C	SmoAnta	0.104	0.976
263	CHEMBL3944267	Cc1c(Cc2ccccn2)nnc(N2CCN(c3cnc(C(C)(C)O)cn3)[C@H](C)C2)c1C	SmoAnta	0.086	0.984
264	CHEMBL3961874	Cc1c(Cc2ccccc2)nnc(N2CCC(F)(c3ccc(C(C)(C)O)cc3)CC2)c1C	SmoAnta	0.089	0.976
265	CHEMBL3925179	CC(=O)c1cnc(N2CCN(c3nnc(Cc4ccccc4)c(C)c3C)C[C@H]2C)cn1	SmoAnta	0.1	0.973
266	CHEMBL3967114	Cc1c(-c2cccc(Cl)c2)nnc(N2CCN(c3ccc(C(F)(F)F)cn3)CC2)c1C	SmoAnta	0.063	0.979

267	CHEMBL3983242	COC(=O)c1ncc(N2CCN(c3nnc(Cc4ccccc4)c4c3CCC4)C[C@H]2C)nc1C(F)(F)F	SmoAnta	0.107	0.962
268	CHEMBL3895940	COC(=O)c1cnc(N2CCN(c3nnc(Cc4ccccn4)c(C)c3C)C[C@H]2C)cn1	SmoAnta	0.089	0.978
269	CHEMBL3951798	Cc1c(Cc2ccccc2)nnc(N2CCC(N3CCc4ccccc4C3)CC2)c1C	SmoAnta	0.1	0.97
270	CHEMBL3969729	Cc1c(-c2ccc(C(C)C)cc2)nnc(N2CCN(c3ccc(C(F)(F)F)cn3)CC2)c1C	SmoAnta	0.05	0.981
271	CHEMBL3898393	CCCCOC(=O)c1cnc(N2CCN(c3nnc(-c4ccc(F)c(C#N)c4)c(C)c3C)CC2)cc1C(F)(F)F	SmoAnta	0.074	0.968
272	CHEMBL3974732	COc1ccc(-c2nnc(N3CCN(c4ncc(C(C)(C)O)c(C(F)(F)F)n4)CC3)c(C)c2C)cc1	SmoAnta	0.066	0.978
273	CHEMBL3934045	Cc1c(Cc2ccccc2)nnc(N2CCN(c3cnc(C(=O)CO)cn3)[C@H](C)C2)c1C	SmoAnta	0.089	0.98
274	CHEMBL3938334	Cc1c(Cc2ccccc2)nnc(N2CCN(c3cnc(C(=O)NCCN4CCN(C)CC4)cn3)[C@H](C)C2)c1C	SmoAnta	0.079	0.981
275	CHEMBL3944445	COC(=O)c1cnc(N2CCN(c3nnc(Cc4ccccc4)c(C)c3C)C[C@@H]2C)cn1	SmoAnta	0.089	0.973
276	CHEMBL2031273	COc1cc(C(=O)NC(=O)Nc2cccc(NC(=O)c3ccccc3)c2)cc(OC)c1OC	SmoAnta	0.128	0.928
277	CHEMBL2031291	COc1cc(C(=O)N=C(N)Nc2ccc(C)c(NC(=O)c3cccc(-c4ccccc4)c3)c2)cc(OC)c1OC	SmoAnta	0.122	0.937
278	CHEMBL1580265	O=C(Nc1ccc(-c2csc(-c3ccccc3)n2)cc1)c1ccccc1Cl	SmoAnta	0.085	0.924
279	CHEMBL1082713	C[C@H]1CN(c2nnc(-c3ccccn3)c3ccccc23)CCN1C(=O)c1ccccc1	SmoAnta	0.13	0.939
280	CHEMBL2031281	COc1cc(C(=O)NC(=O)Nc2cccc(NC(=O)c3cc4ccccc4[nH]3)c2)cc(OC)c1OC	SmoAnta	0.112	0.937
281	CHEMBL474281	Cc1cccc(C(=O)Nc2ccc3c(c2)CC(NCc2ccccn2)C3)c1-c1ccc(C(F)(F)F)cc1	SmoAnta	0.221	0.856
282	CHEMBL4440172	C[C@@]1(c2ccc(Cl)cc2Cl)OC[C@@H](COc2ccc(N3CCN(c4ccc(NC(=O)NN)cc4)CC3)cc2)O1	SmoAnta	0.064	0.97
283	CHEMBL551065	CCCC(CCC)C(=O)NCc1ccc2c(cnn2-c2cccc(C)c2C)c1	SmoAnta	0.192	0.927
284	CHEMBL1615189	CC(=O)N=c1[nH]c2ccc(-c3cnc(Cl)c(NS(=O)(=O)c4ccc(F)cc4)c3)cc2s1	SmoAnta	0.259	0.87
285	CHEMBL474508	Cc1cccc(C(=O)Nc2ccc3c(c2)C[C@@H](NCc2scnc2C)C3)c1-c1ccc(C(F)(F)F)cc1	SmoAnta	0.212	0.874
286	CHEMBL4644288	CN(C(=O)c1ccc(F)cc1C(F)(F)F)C1CCN(C(=O)c2ccccc2N=c2cc[nH]n2C)CC1	SmoAnta	0.182	0.942
287	CHEMBL550190	CCCC(CCC)C(=O)NCc1ccc2c(cnn2-c2ccc(OC)nc2)c1	SmoAnta	0.173	0.928
288	CHEMBL2031267	COc1cc(C(=O)NC(=S)Nc2ccc(Cl)c(NC(=O)c3ccc(-c4ccccc4)cc3)c2)cc(OC)c1OC	SmoAnta	0.207	0.875
289	CHEMBL561069	CCCC(CCC)C(=O)NCc1ccc2c(cnn2-c2ccc(F)cc2)c1	SmoAnta	0.182	0.925
290	CHEMBL3819191	CN1CCN(C(=O)c2cc3c(s2)CCN(c2ccc(Cl)c(-c4nc5ccccc5n4C)c2)C3)CC1	SmoAnta	0.126	0.942
291	CHEMBL497412	FC(F)(F)c1ccc(N2CCN(c3nnc(Cc4ccccc4)c4occc34)CC2)nc1	SmoAnta	0.083	0.97
292	CHEMBL4588930	Cc1ccc(=NCc2ccnn2-c2nnc(N3CCC(C(=O)Nc4ccccc4C)CC3)s2)[nH]c1	SmoAnta	0.136	0.932
293	CHEMBL4562342	C[C@@]1(c2ccc(Cl)cc2Cl)OC[C@@H](COc2ccc(N3CCN(c4ccc(-n5cn[nH]c5=O)cc4)CC3)cc2)O1	SmoAnta	0.058	0.97
294	CHEMBL557114	CCCC(CCC)C(=O)NCc1ccc2c(cnn2-c2cccc3c2CCCC3)c1	SmoAnta	0.205	0.932
295	CHEMBL3604624	O=C(Nc1cc(-c2ccc3[nH]c(=NCC4CC4)sc3c2)cnc1Cl)c1ccccc1	SmoAnta	0.144	0.894
296	CHEMBL3604623	O=C(Nc1cc(-c2ccc3[nH]c(=NC4CC4)sc3c2)cnc1Cl)c1cccc(Cl)c1	SmoAnta	0.168	0.896
297	CHEMBL495620	N#Cc1ccc(N2CCCN(c3nnc(Cc4ccccc4)c4ccccc34)CC2)nc1	SmoAnta	0.13	0.956
298	CHEMBL2031295	COc1cc(C(=O)N=C(N)Nc2ccc(Cl)c(C(=O)Nc3ccccc3)c2)cc(OC)c1OC	SmoAnta	0.163	0.909
299	CHEMBL4572598	C[C@]1(c2ccc(Cl)cc2Cl)OC[C@@H](COc2ccc(N3CCN(c4ccc(NC(=O)c5cccc(O)c5)cc4)CC3)cc2)O1	SmoAnta	0.058	0.976
300	CHEMBL497217	COC(=O)c1ccc(Cc2nnc(N3CCN(c4ccc(C#N)cn4)CC3)c3ccccc23)cc1	SmoAnta	0.129	0.948
301	CHEMBL3604616	CC(=O)N=c1[nH]c2ccc(-c3cnc(Cl)c(NC(=O)c4ccc(F)cc4)c3)cc2s1	SmoAnta	0.159	0.88
302	CHEMBL1813101	O=C(Nc1c(Cl)cc(F)cc1Cl)N1CCn2c(c(O)n([C@H]3C[C@@H]3c3ccccc3)c2=O)C1	SmoAnta	0.101	0.979
303	CHEMBL2031253	COc1cc(C(=O)NC(=S)Nc2cccc(-c3cc4ccccc4[nH]3)c2)cc(OC)c1OC	SmoAnta	0.18	0.912
304	CHEMBL3933195	C[C@@H]1CC[C@H]2[C@@H](C)[C@@H](CC(=O)Nc3ccc(Cl)c(-c4ccccn4)c3)O[C@@H]3O[C@@]4(C)CC[C@@H]1[C@@]23O4	SmoAnta	0.174	0.935
305	CHEMBL522241	N#Cc1ccc(N2CCN(c3nnc(Cc4ccc(C(F)(F)F)cc4)c4ccccc34)CC2)nc1	SmoAnta	0.124	0.956
306	CHEMBL498457	OCc1ccc(N2CCN(c3nnc(Cc4ccccc4)c4ccccc34)CC2)nc1	SmoAnta	0.109	0.957
307	CHEMBL2031294	COc1cc(C(=O)N=C(N)Nc2ccc(C)c(C(=O)Nc3ccccc3)c2)cc(OC)c1OC	SmoAnta	0.158	0.907
308	CHEMBL2031258	COc1cc(C(=O)NC(=S)Nc2ccc(Cl)c(NC(=O)c3ccccc3)c2)cc(OC)c1OC	SmoAnta	0.201	0.885

309	CHEMBL3819380	Cn1c(-c2cc(N3CCc4sc(C(=O)N5CCC(O)CC5)cc4C3)ccc2Cl)nc2ccccc21	SmoAnta	0.131	0.948
310	CHEMBL3604625	O=C(Nc1cc(-c2ccc3[nH]c(=NCC4CC4)sc3c2)cnc1Cl)c1cccc(Cl)c1	SmoAnta	0.141	0.91
311	CHEMBL473897	Cc1cccc(C(=O)Nc2ccc3c(c2)C[C@@H](NCc2ccncc2)C3)c1-c1ccc(C(F)(F)F)cc1	SmoAnta	0.211	0.86
312	CHEMBL4472335	C[C@]1(c2ccc(Cl)cc2Cl)OC[C@@H](COc2ccc(N3CCN(c4ccc(NC(=O)c5cccnc5)cc4)CC3)cc2)O1	SmoAnta	0.049	0.982
313	CHEMBL4447164	C[C@@]1(c2ccc(Cl)cc2Cl)OC[C@@H](COc2ccc(N3CCN(c4ccc(NC(=O)c5cccc(O)c5)cc4)CC3)cc2)O1	SmoAnta	0.045	0.98
314	CHEMBL561330	CCCC(CCC)C(=O)NCc1ccc2c(cnn2-c2ccccc2C)c1	SmoAnta	0.208	0.921
315	CHEMBL3262645	Cc1cnc(-c2cc(N3CCn4cc(C(=O)Nc5cccnc5)nc4C3)ncc2Cl)c(C)c1	SmoAnta	0.092	0.963
316	CHEMBL564222	CCCC(CCC)C(=O)NCc1ccc2c(cnn2-c2ccccc2)c1	SmoAnta	0.202	0.933
317	CHEMBL454984	Cc1cccc(C(=O)Nc2ccc3c(c2)C[C@@H](NCc2cnc4ccccc4c2)C3)c1-c1ccc(C(F)(F)F)cc1	SmoAnta	0.18	0.901
318	CHEMBL3604622	O=C(Nc1cc(-c2ccc3[nH]c(=NC4CC4)sc3c2)cnc1Cl)c1ccccc1	SmoAnta	0.163	0.89
319	CHEMBL4473575	C[C@]1(c2ccc(Cl)cc2Cl)OC[C@@H](COc2ccc(N3CCN(c4ccc(-n5cn[nH]c5=O)cc4)CC3)cc2)O1	SmoAnta	0.064	0.969
320	CHEMBL1082386	N#Cc1cccc(-c2nnc(N3CCN(C(=O)c4ccccc4)CC3)c3ccccc23)c1	SmoAnta	0.127	0.936
321	CHEMBL1084241	C[C@H]1CN(c2nnc(-c3cccnc3)c3ccccc23)CCN1C(=O)c1ccccc1	SmoAnta	0.133	0.941
322	CHEMBL3817897	Cn1c(-c2cc(N3CCc4sc(C(=O)N5CCN(c6ccc(Cl)c(Cl)c6)CC5)cc4C3)ccc2Cl)nc2ccccc21	SmoAnta	0.115	0.951
323	CHEMBL2031284	COc1cc(C(=O)N=C(N)Nc2cccc(NC(=O)c3ccc(N4CCOCC4)cc3)c2)cc(OC)c1OC	SmoAnta	0.106	0.948
324	CHEMBL3819639	Cn1c(-c2cc(N3CCc4sc(C(=O)N5CCN(S(C)(=O)=O)CC5)cc4C3)ccc2Cl)nc2ccccc21	SmoAnta	0.124	0.942
325	CHEMBL4525451	Cc1ccc(=NCc2ccnn2-c2nnc(N3CCC(C(=O)N=c4cc[nH]cc4C)CC3)s2)[nH]c1	SmoAnta	0.135	0.929
326	CHEMBL1813097	O=C(Nc1cccc(Cl)c1Cl)N1CCn2c(c(O)n([C@H]3C[C@@H]3c3ccccc3)c2=O)C1	SmoAnta	0.084	0.97
327	CHEMBL4276902	CN(C(=O)c1ccc([N+](=O)[O-])cc1)C1CCN(c2nnc(-c3ccnn3C)c3ccccc23)CC1	SmoAnta	0.097	0.972
328	CHEMBL3818767	Cn1c(-c2cccc(N3CCc4sc(C(=O)N5CCN(c6ncccn6)CC5)cc4C3)c2)nc2ccccc21	SmoAnta	0.1	0.947
329	CHEMBL3924243	C[C@@H]1CC[C@H]2[C@@H](C)[C@@H](CC(=O)Nc3ccc(Cl)c(-c4ccccn4)c3)O[C@@H]3O[C@@]4(C)CC[C@@H]1[C@@]23OO4	SmoAnta	0.151	0.939
330	CHEMBL496014	c1ccc(Cc2nnc(N3CCN(c4ccccn4)CC3)c3ccccc23)cc1	SmoAnta	0.136	0.946
331	CHEMBL3818148	Cn1c(-c2cccc(N3CCc4sc(C(=O)Nc5cccnc5)cc4C3)c2)nc2ccccc21	SmoAnta	0.106	0.975
332	CHEMBL4562519	C[C@]1(c2ccc(Cl)cc2Cl)OC[C@@H](COc2ccc(N3CCN(c4ccc([N+](=O)[O-])cc4)CC3)cc2)O1	SmoAnta	0.063	0.973
333	CHEMBL3262644	Cc1cnc(-c2cc(N3CCn4cc(C(=O)N=c5cccc[nH]5)nc4C3)ncc2Cl)c(C)c1	SmoAnta	0.116	0.942
334	CHEMBL3262647	Cc1cnc(-c2cc(N3CCn4cc(C(=O)OCC5CC5)nc4C3)ncc2Cl)c(C)c1	SmoAnta	0.125	0.942
335	CHEMBL3262632	Cc1cnc(-c2cc(N3CCn4cc(C(=O)N5CCCC5)nc4C3)ncc2Cl)c(C)c1	SmoAnta	0.12	0.932
336	CHEMBL4281406	CN(C(=O)c1ccc(Oc2ccccc2)cc1)C1CCN(c2nnc(-c3ccnn3C)c3ccccc23)CC1	SmoAnta	0.085	0.974
337	CHEMBL3949509	COC(=O)C(C)c1ccc(C)c2ccc(C(=O)Nc3ccc(Cl)c(-c4ccccn4)c3)nc12	SmoAnta	0.224	0.88
338	CHEMBL2031084	COc1cc(C(=O)NC(=S)Nc2cccc(NC(=O)c3ccccc3)c2)cc(OC)c1OC	SmoAnta	0.133	0.918
339	CHEMBL4527095	Cc1ccc(=NCc2ccnn2-c2nnc(N3CCC(C(=O)N=c4[nH]cccc4C)CC3)s2)[nH]c1	SmoAnta	0.136	0.928
340	CHEMBL4635021	COc1ccccc1Nc1ccccc1C(=O)N1CCC(N(C)C(=O)c2ccc(F)cc2C(F)(F)F)CC1	SmoAnta	0.167	0.96
341	CHEMBL2031282	COc1cc(C(=O)N=C(N)Nc2cccc(NC(=O)c3ccccc3)c2)cc(OC)c1OC	SmoAnta	0.152	0.9
342	CHEMBL4290935	COc1cc(OC)c2c(=O)c(-c3ccc(OCc4ccccc4)c(OCc4ccccc4)c3)coc2c1	SmoAnta	0.29	0.826
343	CHEMBL3817987	Cn1c(-c2cccc(N3CCc4sc(C(=O)N5CCOCC5)cc4C3)c2)nc2ccccc21	SmoAnta	0.114	0.941
344	CHEMBL3262639	Cc1cnc(-c2cc(N3CCn4cc(C(=O)Nc5ccccc5)nc4C3)ncc2Cl)c(C)c1	SmoAnta	0.093	0.957
345	CHEMBL3126692	CS(=O)(=O)c1ccc(C(=O)Nc2ccc(Cl)c(NC(=O)Nc3ccc(C(F)(F)F)cc3Cl)c2)c(Cl)c1	SmoAnta	0.23	0.796
346	CHEMBL560932	CCCN(CCC)C(=O)NCc1ccc2c(cnn2-c2cccc(F)cc2)c1	SmoAnta	0.2	0.948
347	CHEMBL4639888	CN(C(=O)c1ccc([N+](=O)[O-])cc1)C1CCN(C(=O)c2ccccc2N=c2cc[nH]n2C)CC1	SmoAnta	0.155	0.932
348	CHEMBL4462158	C[C@@]1(c2ccc(Cl)cc2Cl)OC[C@@H](COc2ccc(N3CCN(c4ccc([N+](=O)[O-])cc4)CC3)cc2)O1	SmoAnta	0.057	0.975

349	CHEMBL3818511	Cn1c(-c2cc(N3CCc4sc(C(=O)N5CCCC5)cc4C3)ccc2Cl)nc2ccccc21	SmoAnta	0.136	0.942
350	CHEMBL3817945	CN1CCN(C(=O)c2cc3c(s2)CCN(c2cccc(-c4nc5ccccc5n4C)c2)C3)CC1	SmoAnta	0.11	0.954
351	CHEMBL562811	CCCC(C)(C)C(=O)NCc1ccc2c(cnn2-c2ccc(F)cc2)c1	SmoAnta	0.17	0.939
352	CHEMBL563112	CCCC(CCC)C(=O)NCc1ccc2c(cnn2-c2cccnc2OC)c1	SmoAnta	0.208	0.933
353	CHEMBL500172	CC(=O)Nc1ccc(CN[C@H]2Cc3ccc(NC(=O)c4cccc(C)c4-c4ccc(C(F)(F)F)cc4)cc3C2)cc1	SmoAnta	0.17	0.912
354	CHEMBL3262640	Cc1cnc(-c2cc(N3CCn4cc(C(=O)Nc5ccccc5F)nc4C3)ncc2Cl)c(C)c1	SmoAnta	0.092	0.956
355	CHEMBL3819236	Cn1c(-c2cccc(N3CCc4sc(C(=O)N5CCCC5)cc4C3)c2)nc2ccccc21	SmoAnta	0.111	0.961
356	CHEMBL552208	CCCC(C)C(=O)NCc1ccc2c(cnn2-c2ccc(F)cc2)c1	SmoAnta	0.161	0.932
357	CHEMBL2031292	COc1cc(C(=O)N=C(N)Nc2ccc(Cl)c(NC(=O)c3ccccc3)c2)cc(OC)c1OC	SmoAnta	0.19	0.918
358	CHEMBL3818879	Cn1c(-c2cccc(N3CCc4sc(C(=O)N5CCN(S(C)(=O)=O)CC5)cc4C3)c2)nc2ccccc21	SmoAnta	0.104	0.954
359	CHEMBL3262646	Cc1cnc(-c2cc(N3CCn4cc(C(=O)OC(C)C)nc4C3)ncc2Cl)c(C)c1	SmoAnta	0.114	0.945
360	CHEMBL498017	N#Cc1ccc(N2CCC(c3cn(Cc4ccccc4)c4ccccc34)CC2)nc1	SmoAnta	0.134	0.928
361	CHEMBL4553897	Cc1c(O)n(-c2ccc(Cl)cc2)c(=O)n1CC1CCCCC1	SmoAnta	0.178	0.9
362	CHEMBL4583477	C[C@]1(c2ccc(Cl)cc2Cl)OC[C@@H](COc2ccc(N3CCN(c4ccc(N)cc4)CC3)cc2)O1	SmoAnta	0.092	0.96
363	CHEMBL3262643	Cc1cnc(-c2cc(N3CCn4cc(C(=O)Nc5ccc(F)c(F)c5)nc4C3)ncc2Cl)c(C)c1	SmoAnta	0.08	0.961
364	CHEMBL496013	c1ccc(Cc2nnc(N3CCN(c4ccccc4)CC3)c3ccccc23)cc1	SmoAnta	0.134	0.947
365	CHEMBL3818648	Cn1c(-c2cccc(N3CCc4sc(C(=O)N5CCC(O)CC5)cc4C3)c2)nc2ccccc21	SmoAnta	0.114	0.948
366	CHEMBL3262642	Cc1cnc(-c2cc(N3CCn4cc(C(=O)Nc5ccc(F)cc5)nc4C3)ncc2Cl)c(C)c1	SmoAnta	0.091	0.96
367	CHEMBL3290331	C[C@@H]1CN(c2nnc(Cc3ccccc3)c3ccc(Cl)cc23)CCN1c1ccc(C#N)cn1	SmoAnta	0.189	0.901
368	CHEMBL184721	Cc1ccc(NC(=O)c2cccc(N3CCOCC3)c2)cc1NC(=O)c1ccc(OCc2ccccn2)cc1	SmoAnta	0.149	0.926
369	CHEMBL184712	Cc1ccc(NC(=O)c2cc(F)cc(N3CCCC3)c2)cc1NC(=O)c1ccc(OCc2ccccn2)cc1	SmoAnta	0.138	0.933
370	CHEMBL3818296	Cn1c(-c2cccc(N3CCc4sc(C(=O)N5CCN(c6ccc(Cl)c(Cl)c6)CC5)cc4C3)c2)nc2ccccc21	SmoAnta	0.087	0.949
371	CHEMBL4452315	Cc1ccc(=NCc2ccnn2-c2nnc(N3CCC(C(=O)Nc4cccnc4C)CC3)s2)[nH]c1	SmoAnta	0.131	0.937
372	CHEMBL473892	O=C(Nc1ccc2c(c1)CC(NCc1ccccn1)C2)c1ccccc1-c1ccc(C(F)(F)F)cc1	SmoAnta	0.219	0.856
373	CHEMBL2031080	COc1cc(C(=O)NC(=S)Nc2ccc(Cl)c(C(=O)Nc3ccccc3)c2)cc(OC)c1OC	SmoAnta	0.176	0.915
374	CHEMBL3262630	Cc1cnc(-c2cc(N3CCn4cc(C(=O)NCC5CC5)nc4C3)ncc2Cl)c(C)c1	SmoAnta	0.122	0.946
375	CHEMBL4163487	Cc1ccc(NCc2cccn2-c2nnc(N3CCC(C(=O)NC4CCCC4)CC3)s2)cc1	SmoAnta	0.144	0.939
376	CHEMBL3262636	COC[C@H]1CCCN1C(=O)c1cn2c(n1)CN(c1cc(-c3ncc(C)cc3C)c(Cl)cn1)CC2	SmoAnta	0.12	0.958
377	CHEMBL4470299	Cc1ccc(=NCc2ccnn2-c2nnc(N3CCC(C(=O)Nc4cnccc4C)CC3)s2)[nH]c1	SmoAnta	0.128	0.935
378	CHEMBL4539606	Cc1c(O)n(-c2ccc(Cl)cc2)c(=O)n1-c1ccccc1	SmoAnta	0.146	0.895
379	CHEMBL4632537	COc1ccc(C(=O)N(C)C2CCN(C(=O)c3ccccc3N=c3cc[nH]n3C)CC2)cc1	SmoAnta	0.168	0.926
380	CHEMBL2031285	COc1cc(C(=O)N=C(N)Nc2cccc(NC(=O)c3cn4ccsc4n3)c2)cc(OC)c1OC	SmoAnta	0.145	0.921
381	CHEMBL3262654	COCC(=O)Nc1cn2c(n1)CN(c1cc(-c3ncc(C)cc3C)c(Cl)cn1)CC2	SmoAnta	0.136	0.931
382	CHEMBL1813099	O=C(Nc1cccc(Cl)c1Cl)N1CCn2c(c(O)n([C@H]3C[C@@H]3c3ccccc3)c2=O)C1	SmoAnta	0.085	0.975
383	CHEMBL4168100	Cc1ccc(NCc2cccn2-c2nnc(N3CCC(C(=O)NC4CCCCC4)CC3)s2)cc1	SmoAnta	0.139	0.939
384	CHEMBL1813098	O=C(Nc1cccc(Cl)c1Cl)N1CCn2c(c(O)n([C@@H]3C[C@H]3c3ccccc3)c2=O)C1	SmoAnta	0.082	0.98
385	CHEMBL556427	CCCC(CCC)C(=O)NCc1ccc2c(cnn2-c2cccc(F)c2)c1	SmoAnta	0.207	0.935
386	CHEMBL3262627	CCOC(=O)c1cn2c(n1)CN(c1cc(-c3ncc(C)cc3C)c(Cl)cn1)CC2	SmoAnta	0.117	0.94
387	CHEMBL497003	c1ccc(N2CCN(c3nnc(Cc4ccncc4)c4ccccc34)CC2)nc1	SmoAnta	0.133	0.946
388	CHEMBL550254	CCC(CC)C(=O)NCc1ccc2c(cnn2-c2ccc(F)cc2)c1	SmoAnta	0.177	0.952
389	CHEMBL497210	FC(F)(F)c1ccc(N2CCN(c3nnc(Cc4ccccc4)c4[nH]cnc34)CC2)nc1	SmoAnta	0.079	0.978
390	CHEMBL3262648	Cc1cnc(-c2cc(N3CCn4cc(C(=O)OC5CCCC5)nc4C3)ncc2Cl)c(C)c1	SmoAnta	0.105	0.954
391	CHEMBL4645447	COc1ccc(Nc2ccccc2C(=O)N2CCC(N(C)C(=O)c3ccc(F)cc3C(F)(F)F)CC2)cc1	SmoAnta	0.109	0.954

392	CHEMBL474892	Cc1cccc(C(=O)Nc2ccc3c(c2)C[C@H](NCc2nccs2)C3)c1-c1ccc(C(F)(F)F)cc1	SmoAnta	0.215	0.851
393	CHEMBL3262633	Cc1cnc(-c2cc(N3CCn4cc(C(=O)N5CCOCC5)nc4C3)ncc2Cl)c(C)c1	SmoAnta	0.12	0.937
394	CHEMBL2031254	COc1cc(C(=O)NC(=S)Nc2cccc(-c3cn4ccccc4n3)c2)cc(OC)c1OC	SmoAnta	0.156	0.919
395	CHEMBL3604617	CC(=O)N=c1[nH]c2ccc(-c3cnc(Cl)c(NC(=O)c4ccc(C)cc4)c3)cc2s1	SmoAnta	0.157	0.884
396	CHEMBL4063037	O=C(c1ccccc1)c1c(-c2ccccc2)[nH]c2ccc(Br)cc2c1=O	SmoAnta	0.295	0.766
397	CHEMBL4284509	CN(C(=O)c1ccc(B(O)O)cc1)C1CCN(c2nnc(-c3ccnn3C)c3ccccc23)CC1	SmoAnta	0.091	0.973
398	CHEMBL550661	CCCC(CCC)C(=O)NCc1ccc2c(cnn2-c2ccccc2F)c1	SmoAnta	0.202	0.932
399	CHEMBL2031286	COc1cc(C(=O)N=C(N)Nc2cccc(-n3ccc4ccccc43)c2)cc(OC)c1OC	SmoAnta	0.152	0.921
400	CHEMBL563992	CCCC(CCC)C(=O)NCc1ccc2c(cnn2-c2ccc(C)cc2)c1	SmoAnta	0.189	0.935
401	CHEMBL3262653	CCC(=O)Nc1cn2c(n1)CN(c1cc(-c3ncc(C)cc3C)c(Cl)cn1)CC2	SmoAnta	0.118	0.941
402	CHEMBL4090942	COc1cccc(C(=O)c2c(-c3ccccc3)[nH]c3ccccc3c2=O)c1	SmoAnta	0.405	0.698
403	CHEMBL3262651	Cc1cnc(-c2cc(N3CCn4cc(CN5CCOCC5)nc4C3)ncc2Cl)c(C)c1	SmoAnta	0.144	0.945
404	CHEMBL497819	N#Cc1ccc(N2CCN(c3cnc(Cc4ccccc4)c4ccccc34)CC2)nc1	SmoAnta	0.108	0.96
405	CHEMBL4471156	C[C@@]1(c2ccc(Cl)cc2Cl)OC[C@@H](COc2ccc(N3CCN(c4ccc(N)cc4)CC3)cc2)O1	SmoAnta	0.058	0.979
406	CHEMBL3604620	CC(=O)N=c1[nH]c2ccc(-c3cnc(Cl)c(NCc4ccccc4)c3)cc2s1	SmoAnta	0.173	0.859
407	CHEMBL3262649	Cc1cnc(-c2cc(N3CCn4cc(C(=O)Oc5ccccc5)nc4C3)ncc2Cl)c(C)c1	SmoAnta	0.101	0.955
408	CHEMBL561995	CCCC(CCC)C(=O)NCc1ccc2c(cnn2-c2c(C)cccc2C)c1	SmoAnta	0.209	0.914
409	CHEMBL4171049	Cc1oc(-c2cccc(Cl)c2)nc1CN1CCC(C(=O)NC2CCCc3ccccc32)CC1	SmoAnta	0.139	0.93
410	CHEMBL3955601	Cc1c(Cc2ccccc2)nnc(N2CCC(C)(c3nc4cccnc4[nH]3)CC2)c1C	SmoAnta	0.124	0.962
411	CHEMBL3960935	Cc1c(-c2ccc(F)cc2)nnc(N2CCN(c3cnc(C(=O)O)cn3)[C@H](C)C2)c1C	SmoAnta	0.07	0.977
412	CHEMBL3915704	Cc1c(Cc2ccccc2)nnc(N2CCN(Cc3ccccc3)CC2)c1C	SmoAnta	0.142	0.949
413	CHEMBL3919359	COC(=O)c1cnc(N2CCN(c3nnc(-c4ccccc4-c4ccccc4)c(C)c3C)C[C@H]2C)cn1	SmoAnta	0.117	0.956
414	CHEMBL3910002	Cc1c(Cc2ccccc2)nnc(N2CCN(c3ncc(C(=O)O)c(C(F)(F)F)n3)[C@H](C)C2)c1C	SmoAnta	0.104	0.965
415	CHEMBL3981990	COC(=O)c1cnc(N2CCN(c3nnc(-c4ccc(F)c(Cl)c4)c(C)c3C)C[C@H]2C)cn1	SmoAnta	0.069	0.969
416	CHEMBL3973278	COC(=O)c1cnc(N2CCN(c3nnc(-c4ccc(OC)cc4)c(C)c3C)C[C@H]2C)cn1	SmoAnta	0.061	0.976
417	CHEMBL3967640	COC(=O)c1cnc(N2CCN(c3nnc(-c4ccc(C(F)(F)F)cc4)c(C)c3C)C[C@H]2C)cn1	SmoAnta	0.055	0.978
418	CHEMBL3970317	COC(=O)c1cnc(N2CCN(c3nnc(-c4ccc(F)cc4)c(C)c3C)C[C@H]2C)cn1	SmoAnta	0.064	0.982
419	CHEMBL3920191	COC(=O)c1cnc(N2CCN(c3nnc(Cc4ccccc4)c4c3CCC4)C[C@H]2C)cn1	SmoAnta	0.093	0.974
420	CHEMBL3916441	Cc1c(-c2ccccc2)nnc(N2CCN(c3ccc(C(F)(F)F)cn3)CC2)c1C	SmoAnta	0.053	0.98
421	CHEMBL4293519	CN(C(=O)c1ccccc1)C1CCN(c2nnc(-c3ccnn3C)c3ccccc23)CC1	SmoAnta	0.114	0.955
422	CHEMBL366255	Cc1ccc(NC(=O)c2cccc(N(C)C)c2)cc1NC(=O)c1ccc(OCc2ccccn2)cc1	SmoAnta	0.136	0.919
423	CHEMBL2031256	COc1cc(C(=O)NC(=S)Nc2cccc(-c3cn4ccsc4n3)c2)cc(OC)c1OC	SmoAnta	0.165	0.918
424	CHEMBL4161601	Cc1ccc(NCc2cccn2-c2nnc(N3CCC(C(=O)NCC(C)C)CC3)s2)cc1	SmoAnta	0.156	0.929
425	CHEMBL515245	O=C(Nc1ccc2c(c1)CC(NCc1ccccn1)C2)c1ccccc1-c1ccccc1	SmoAnta	0.127	0.936
426	CHEMBL1813112	C[C@]12CN(C(=O)Nc3ccccc3-c3ccccc3)CCN1C(=O)N([C@H]1C[C@@H]1c1ccccc1)C2=O	SmoAnta	0.169	0.93
427	CHEMBL559001	CCCC(CCC)C(=O)NCc1ccc2c(cnn2-c2ccc(Cl)cc2)c1	SmoAnta	0.18	0.934
428	CHEMBL568916	CCCCC(=O)NCc1ccc2c(cnn2-c2ccc(F)cc2)c1	SmoAnta	0.151	0.95
429	CHEMBL3262637	Cc1cnc(-c2cc(N3CCn4cc(C(=O)N5CCC(O)CC5)nc4C3)ncc2Cl)c(C)c1	SmoAnta	0.113	0.939
430	CHEMBL1600636	Cc1ccc(NCc2cccn2-c2nnc(N3CCC(C(=O)NC(C)C)CC3)s2)cc1	SmoAnta	0.173	0.91
431	CHEMBL4175623	O=C(Nc1cccc(N2CCCN(Cc3ccc(F)c(F)c3)C2=O)c1)OCc1ccccc1	SmoAnta	0.169	0.944
432	CHEMBL4215992	Cc1cc(-c2ncc(CNC(=O)c3ccc4c(c3)[nH]c3ccccc34)cc2C#N)ccn1	SmoAnta	0.156	0.942
433	CHEMBL551932	CCCN(CCC)C(=O)N(C)Cc1ccc2c(cnn2-c2ccc(F)cc2)c1	SmoAnta	0.177	0.943
434	CHEMBL3259848	CCNC(=O)c1cn2c(n1)CN(c1cc(-c3ncc(C)cc3C)c(Cl)cn1)CC2	SmoAnta	0.13	0.938

435	CHEMBL4641704	CN(C(=O)c1ccc(C(F)(F)F)cc1)C1CCN(C(=O)c2ccccc2N=c2cc[nH]n2C)CC1	SmoAnta	0.152	0.931
436	CHEMBL551873	CCCC(CCC)C(=O)NCc1ccc2c(cnn2-c2ccc(C(C)=O)cc2)c1	SmoAnta	0.176	0.937
437	CHEMBL497820	N#Cc1ccc(N2CCN(c3ncc(Cc4ccccc4)c4ccccc34)CC2)nc1	SmoAnta	0.109	0.955
438	CHEMBL4290843	CN(C)c1ccc(C(=O)N(C)C2CCN(c3nnc(-c4ccnn4C)c4ccccc34)CC2)cc1	SmoAnta	0.093	0.972
439	CHEMBL4450803	Cc1c(O)n(-c2c(F)c(F)c(F)c(F)c2F)c(=O)n1Cc1ccccc1	SmoAnta	0.251	0.969
440	CHEMBL4284830	CN(C(=O)c1ccc(F)cc1)C1CCN(c2nnc(-c3ccnn3C)c3ccccc23)CC1	SmoAnta	0.098	0.966
441	CHEMBL523431	O=C(O)c1ccc(N2CCN(c3nnc(Cc4ccccc4)c4ccccc34)CC2)nc1	SmoAnta	0.099	0.975
442	CHEMBL1813100	O=C(Nc1cccc(Cl)c1Cl)N1CCn2c(c(O)n([C@@H]3C[C@H]3c3ccccc3)c2=O)C1	SmoAnta	0.084	0.973
443	CHEMBL4173721	Cc1ccc(NCc2cccn2-c2nnc(N3CCC(C(=O)NCC4CC4)CC3)s2)cc1	SmoAnta	0.152	0.93
444	CHEMBL559874	CCCC(CCC)C(=O)NCc1ccc2c(cnn2-c2ccc(C(=O)OC(C)(C)C)cc2)c1	SmoAnta	0.136	0.946
445	CHEMBL3262652	Cc1cnc(-c2cc(N3CCn4cc(NC(=O)C(F)(F)F)nc4C3)ncc2Cl)c(C)c1	SmoAnta	0.096	0.956
446	CHEMBL2031079	COc1cc(C(=O)NC(=S)Nc2ccc(C)c(C(=O)Nc3ccccc3)c2)cc(OC)c1OC	SmoAnta	0.173	0.892
447	CHEMBL497209	FC(F)(F)c1ccc(N2CCN(c3nnc(Cc4ccccc4)c4ccoc34)CC2)nc1	SmoAnta	0.083	0.977
448	CHEMBL4294637	CN(C(=O)c1ccc(OCc2ccccc2)cc1)C1CCN(c2nnc(-c3ccnn3C)c3ccccc23)CC1	SmoAnta	0.113	0.956
449	CHEMBL557448	CCCC(CCC)C(=O)NCc1ccc2c(cnn2-c2cccnc2)c1	SmoAnta	0.194	0.936
450	CHEMBL4646599	CC(=O)c1ccc(C(=O)N(C)C2CCN(C(=O)c3ccccc3N=c3cc[nH]n3C)CC2)cc1	SmoAnta	0.155	0.943
451	CHEMBL4279209	CN(C(=O)c1ccc(N)cc1)C1CCN(c2nnc(-c3ccnn3C)c3ccccc23)CC1	SmoAnta	0.107	0.96
452	CHEMBL4455409	C[C@]1(c2ccc(Cl)cc2Cl)OC[C@@H](COc2ccc(N3CCN(c4ccc(NC(=O)c5ccccc5O)cc4)CC3)cc2)O1	SmoAnta	0.055	0.98
453	CHEMBL1086141	C[C@H]1CN(c2nnc(N3CC=CC=N3)c3ccccc23)CCN1C(=O)c1ccccc1	SmoAnta	0.132	0.942
454	CHEMBL4164165	O=C(Nc1ccc(N2CCN(Cc3ccccc3O)CC2)nc1)c1ccccc1C(F)(F)F	SmoAnta	0.082	0.969
455	CHEMBL423915	COC(=O)N[C@@H]1Cc2ccc(NC(=O)c3cccc(C)c3-c3ccc(C(F)(F)F)cc3)cc2C1	SmoAnta	0.234	0.823
456	CHEMBL4290044	COc1cc(OC)c2c(=O)c(-c3ccc(OC)c(OCc4ccc(C(F)(F)F)cc4)c3)coc2c1	SmoAnta	0.171	0.887
457	CHEMBL3262629	CNC(=O)c1cn2c(n1)CN(c1cc(-c3ncc(C)cc3C)c(Cl)cn1)CC2	SmoAnta	0.122	0.933
458	CHEMBL1084495	C[C@H]1CN(c2nnc(-c3ncco3)c3ccccc23)CCN1C(=O)c1ccccc1	SmoAnta	0.141	0.923
459	CHEMBL473703	COC(=O)NC1Cc2ccc(NC(=O)c3cccc(C)c3-c3ccc(C(F)(F)F)cc3)cc2C1	SmoAnta	0.236	0.823
460	CHEMBL473073	O=C(Nc1ccc2c(c1)CC(NCc1ccccn1)C2)c1ccccc1-c1ccc(Cl)cc1	SmoAnta	0.185	0.887
461	CHEMBL4282560	CN(C(=O)c1ccc(S(=O)(=O)O)cc1)C1CCN(c2nnc(-c3ccnn3C)c3ccccc23)CC1	SmoAnta	0.094	0.973
462	CHEMBL4282987	CN(C(=O)c1ccc(C=O)cc1)C1CCN(c2nnc(-c3ccnn3C)c3ccccc23)CC1	SmoAnta	0.099	0.961
463	CHEMBL4285647	COc1cc(OC)c2c(=O)c(-c3ccc(OC)c(OCC=C(C)C)c3)coc2c1	SmoAnta	0.215	0.888
464	CHEMBL564987	CCCC(CCC)C(=O)N(C)Cc1ccc2c(cnn2-c2ccc(F)cc2)c1	SmoAnta	0.204	0.945
465	CHEMBL4172388	CC1Cc2ccccc2N1C(=O)c1ccc(=O)n(CCC(=O)N2CCN(c3ccccc3)CC2)n1	SmoAnta	0.166	0.924
466	CHEMBL4289354	CN(C(=O)c1ccc(C(N)=O)cc1)C1CCN(c2nnc(-c3ccnn3C)c3ccccc23)CC1	SmoAnta	0.093	0.975
467	CHEMBL4866798	[N-]=[N+]=NCCOCCOCCOCCOCCOCCS(=O)(=O)c1ccc(C(=O)Nc2ccc(Cl)c(-c3ccccn3)c2)c(Cl)c1	SmoAnta	0.154	0.936
468	CHEMBL4543531	C[C@]1(c2ccc(Cl)cc2Cl)OC[C@@H](COc2ccc(N3CCN(c4ccc(NC(=O)c5ccc(O)cc5)cc4)CC3)cc2)O1	SmoAnta	0.057	0.98
469	CHEMBL365472	Cc1ccc(NC(=O)c2cccc(N(C)C)c2)cc1NC(=O)c1ccc(OC(C)C)cc1	SmoAnta	0.151	0.91
470	CHEMBL4289775	CN(C(=O)c1ccc(S(N)(=O)=O)cc1)C1CCN(c2nnc(-c3ccnn3C)c3ccccc23)CC1	SmoAnta	0.102	0.974
471	CHEMBL3604619	CC(=O)N=c1[nH]c2ccc(-c3cnc(Cl)c(NC(=O)Cc4ccccc4)c3)cc2s1	SmoAnta	0.119	0.918
472	CHEMBL4293248	COc1cc(OC)c2c(=O)c(-c3ccc(OCc4ccc(C(F)(F)F)cc4)c(OC)c3)coc2c1	SmoAnta	0.17	0.924
473	CHEMBL4215124	COc1cc(CNC(=O)c2ccc3c(c2)[nH]c2ccccc23)cnc1-c1ccnc(C)c1	SmoAnta	0.179	0.94
474	CHEMBL4291101	COc1cc(OC)c2c(=O)c(-c3ccc(OC)c(OC/C=C(\C)CCC=C(C)C)c3)coc2c1	SmoAnta	0.187	0.89
475	CHEMBL4286606	COc1cc(OC)c2c(=O)c(-c3ccc(OC)c(OCc4ccccc4)c3)coc2c1	SmoAnta	0.215	0.864
476	CHEMBL495579	N#Cc1ccc(C2CCN(c3nnc(Cc4ccccc4)c4ccccc34)CC2)cc1	SmoAnta	0.107	0.97

477	CHEMBL4467625	Cc1c(O)n(-c2ccc(Cl)cc2)c(=S)n1Cc1ccccc1	SmoAnta	0.187	0.956
478	CHEMBL4473720	C[C@]1(c2ccc(Cl)cc2Cl)OC[C@@H](COc2ccc(N3CCN(c4ccc(NC(=O)c5ccccn5)cc4)CC3)cc2)O1	SmoAnta	0.059	0.977
479	CHEMBL2152370	O=C(C=C1CCCCCC(=O)O[C@H](c2ccccc2)CNC1=O)NCCCC(F)(F)F	SmoAnta	0.144	0.941
480	CHEMBL3262631	Cc1cnc(-c2cc(N3CCn4cc(C(=O)N(C)CCO)nc4C3)ncc2Cl)c(C)c1	SmoAnta	0.12	0.947
481	CHEMBL3262650	Cc1cnc(-c2cc(N3CCn4cc(CO)nc4C3)ncc2Cl)c(C)c1	SmoAnta	0.187	0.904
482	CHEMBL3604611	CC(=O)N=c1[nH]c2ccc(-c3cnc(Cl)c(NC(=O)c4ccccc4Cl)c3)cc2s1	SmoAnta	0.16	0.869
483	CHEMBL4171870	Cc1ccc(NCc2cccn2-c2nnc(N3CCC(C(=O)NC4CC4)CC3)s2)cc1	SmoAnta	0.155	0.929
484	CHEMBL4169989	Cc1ccc(NCc2cccn2-c2nnc(N3CCC(C(=O)NCC(C)(C)O)CC3)s2)cc1	SmoAnta	0.152	0.941
485	CHEMBL4176279	Cc1ccc(NCc2cccn2-c2nnc(N3CCC(C(=O)N4CCCC4)CC3)s2)cc1	SmoAnta	0.153	0.923
486	CHEMBL4571341	C[C@@]1(c2ccc(Cl)cc2Cl)OC[C@@H](COc2ccc(N3CCN(c4ccc(NC(=O)c5cccnc5)cc4)CC3)cc2)O1	SmoAnta	0.058	0.973
487	CHEMBL4644469	CN(C(=O)c1ccc(C#N)cc1)C1CCN(C(=O)c2ccccc2N=c2cc[nH]n2C)CC1	SmoAnta	0.182	0.94
488	CHEMBL557518	CCCC(CCC)C(=O)NCc1ccc2c(cnn2-c2ccncc2)c1	SmoAnta	0.198	0.935
489	CHEMBL4551454	C[C@H]1CN(c2ccc(Cl)cc2)C(=O)N1Cc1ccccc1	SmoAnta	0.186	0.897
490	CHEMBL4168517	Cc1ccc(-c2cc3c(N4CCC(C(=O)NC(C)c5ccccc5)CC4)nccn3n2)cc1C	SmoAnta	0.162	0.885
491	CHEMBL4647112	CN(C(=O)c1ccc(F)cc1C(F)(F)F)C1CCN(C(=O)c2ccccc2Nc2ccc(-c3ccccc3)cc2)CC1	SmoAnta	0.099	0.965
492	CHEMBL2152373	O=C(C=C1CCCCCC(=O)O[C@H](c2ccccc2)CNC1=O)NCc1cccc(Cl)c1	SmoAnta	0.214	0.919
493	CHEMBL3604615	CC(=O)N=c1[nH]c2ccc(-c3cnc(Cl)c(NC(=O)c4cccc(F)c4)c3)cc2s1	SmoAnta	0.17	0.881
494	CHEMBL142450	COC(=O)N[C@H]1Cc2ccc(NC(=O)c3cccc(C)c3-c3ccc(C(F)(F)F)cc3)cc2C1	SmoAnta	0.236	0.821
495	CHEMBL4171838	CCNC(=O)C1CCN(c2nnc(-n3cccc3CNc3ccc(C)cc3)s2)CC1	SmoAnta	0.175	0.904
496	CHEMBL4456292	COc1cccc(C(=O)Nc2ccc(N3CCN(c4ccc(OC[C@@H]5CO[C@](C)(c6ccc(Cl)cc6Cl)O5)cc4)CC3)cc2)c1	SmoAnta	0.052	0.978
497	CHEMBL2152362	O=C(C=C1CCCC[C@H](Cc2ccccc2)C(=O)N[C@H](c2ccccc2)COC1=O)NCc1ccc(Cl)cc1	SmoAnta	0.276	0.845
498	CHEMBL550191	CCCC(CCC)C(=O)NCc1ccc2c(cnn2-c2ncccn2)c1	SmoAnta	0.193	0.938
499	CHEMBL4632758	CN(C(=O)c1ccc(F)cc1C(F)(F)F)C1CCN(C(=O)c2ccccc2Nc2ccc(C#N)cc2)CC1	SmoAnta	0.135	0.952
500	CHEMBL2152371	CC(C)(C)OC(=O)C=C1CCCCCC(=O)N[C@H](c2ccccc2)COC1=O	SmoAnta	0.185	0.896
501	CHEMBL4289866	COc1cc(OC)c2c(=O)c(-c3ccc(OCc4ccccc4)c(OC)c3)coc2c1	SmoAnta	0.101	0.942
502	CHEMBL4279181	COc1cc(OC)c2c(=O)c(-c3ccc(OCC=C(C)C)c(OC)c3)coc2c1	SmoAnta	0.14	0.934
503	CHEMBL4649551	CN(C(=O)c1ccc(F)cc1C(F)(F)F)C1CCN(C(=O)c2ccc[nH]c2=Nc2ccccc2)CC1	SmoAnta	0.206	0.932
504	CHEMBL4562945	Cc1c(O)n(-c2ccc(Cl)cc2)c(=O)n1Cc1c(F)c(F)c(F)c(F)c1F	SmoAnta	0.173	0.914
505	CHEMBL4159684	Cc1ccc(NCc2cccn2-c2nnc(N3CCC(C(=O)N4CCC(O)C4)CC3)s2)cc1	SmoAnta	0.145	0.934
506	CHEMBL4537455	Cc1c(O)n(C2CCCCC2)c(=O)n1Cc1ccccc1	SmoAnta	0.232	0.932
507	CHEMBL4582942	Cc1c(O)n(Cc2ccc(Cl)cc2)c(=O)n1Cc1ccccc1	SmoAnta	0.147	0.962
508	CHEMBL474894	Cc1ccc(-c2ccc(C(F)(F)F)cc2)c(C(=O)Nc2ccc3c(c2)CC(NCc2ccccn2)C3)c1	SmoAnta	0.236	0.844
509	CHEMBL356310	Cc1cccc(C(=O)Nc2ccc3c(c2)C[C@H](NCc2ccccn2)C3)c1-c1ccc(C(F)(F)F)cc1	SmoAnta	0.212	0.859
510	CHEMBL4633504	CN(C(=O)c1ccc(F)cc1C(F)(F)F)C1CCN(C(=O)c2ccccc2Nc2ccccc2C#N)CC1	SmoAnta	0.175	0.952

6. Molecular docking for *ent*-3a

Computational docking was performed using the Maestro environment, version 12.7, with the Schrödinger suite of software, release 2021-1 (Schrödinger Inc., USA). The protein structures (PDB ID: 4JKV, 4N4W, 4O9R, 4QIM, 4QIN, 5L7I and 5V56) were prepared for docking using the Protein Preparation Wizard (Schrödinger).[14] The protonation states of amino acids were refined with PROPKA at pH set to 7.0, and applying restrained minimization with an OPLS4 force field, setting the heavy atom convergence to 0.3 Å RMSD. *Ent*-**3a** was docked to the receptor structures using the standard Glide protocol, centering the docking grids around the co-crystallized ligands when possible. The afforded results were assessed visually and with the obtained docking Score values.

Only crystal structure 5L7I could afford reasonable poses, with the docking score of -8.592 for vismodegib (native ligand for 5L7I), -6.380 for active *ent*-**3a** and -2.894 for inactive **3a**. The binding poses of vismodegib or *ent*-**3a** (Figure S2) were visualized using open-source software Pymol.

7. Compound spectra (NMR and chiral HPLC spectra)

¹H NMR Spectrum of **4e** (400 MHz, $CDCl_3$)

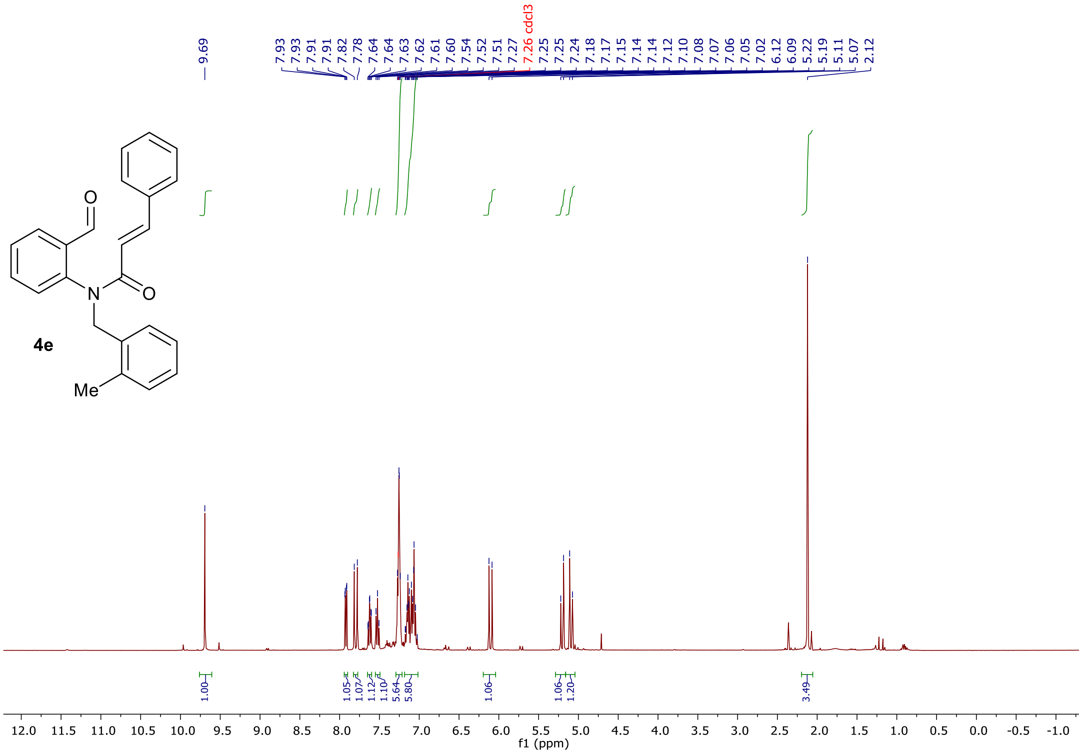

¹³C NMR Spectrum of **4e** (101 MHz, $CDCl_3$)

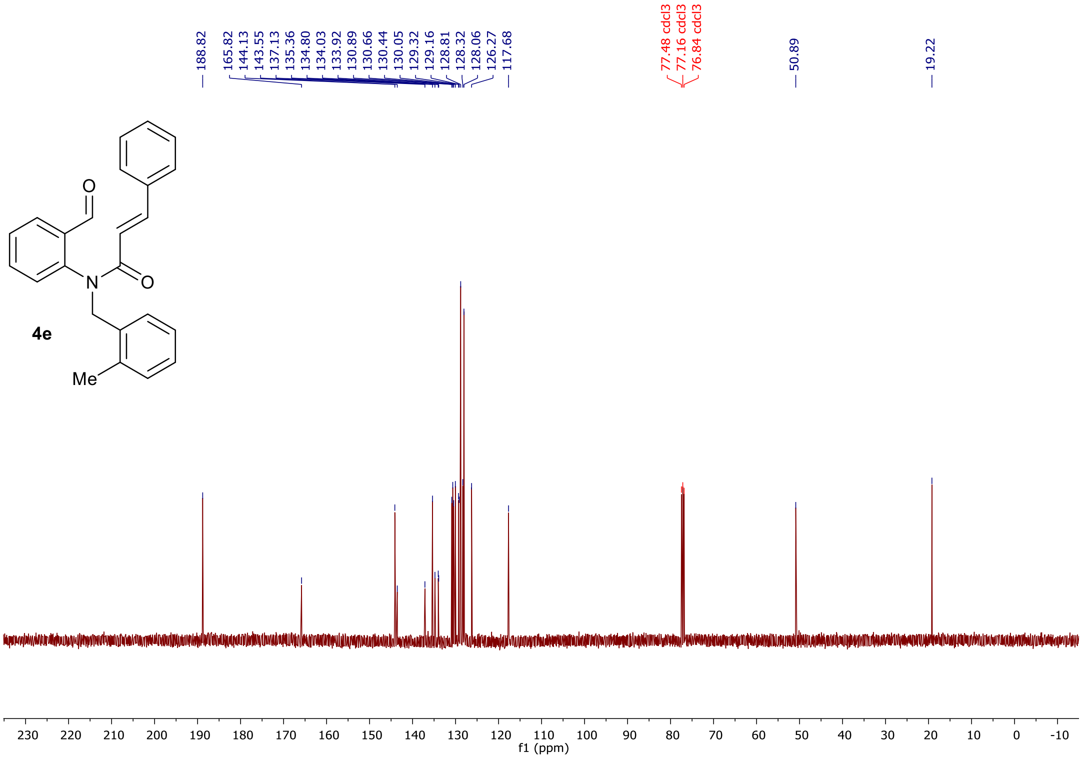

^{1}H NMR Spectrum of **4f** (400 MHz, $CDCl_3$)

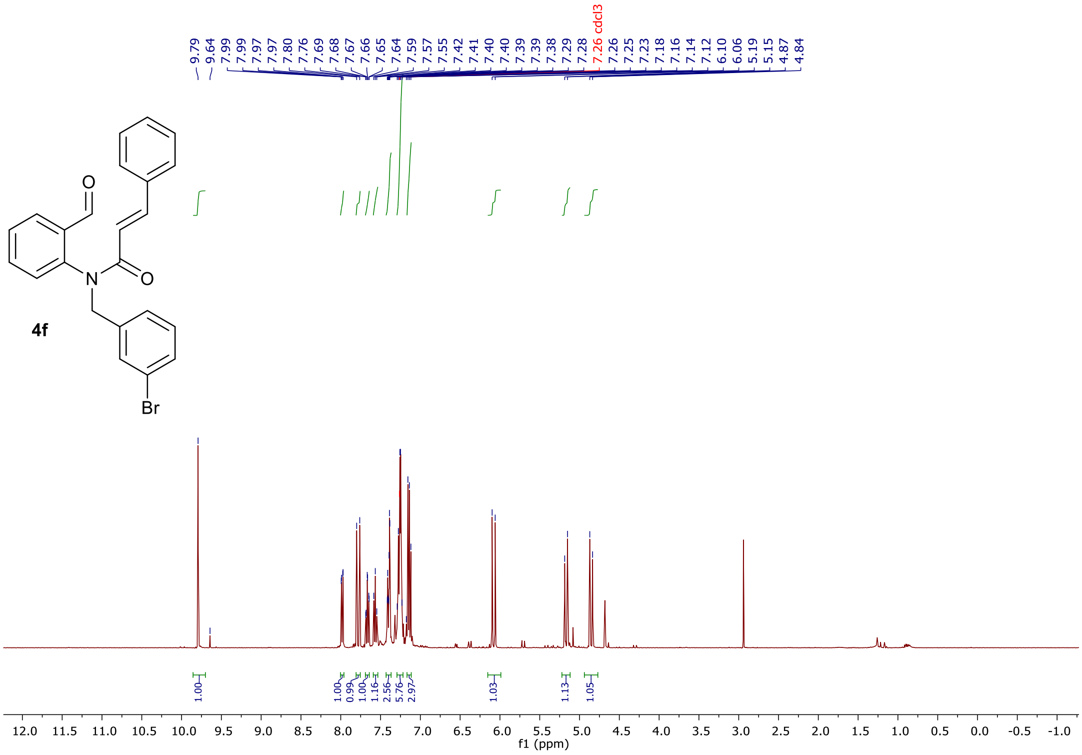

^{13}C NMR Spectrum of **4f** (101 MHz, $CDCl_3$)

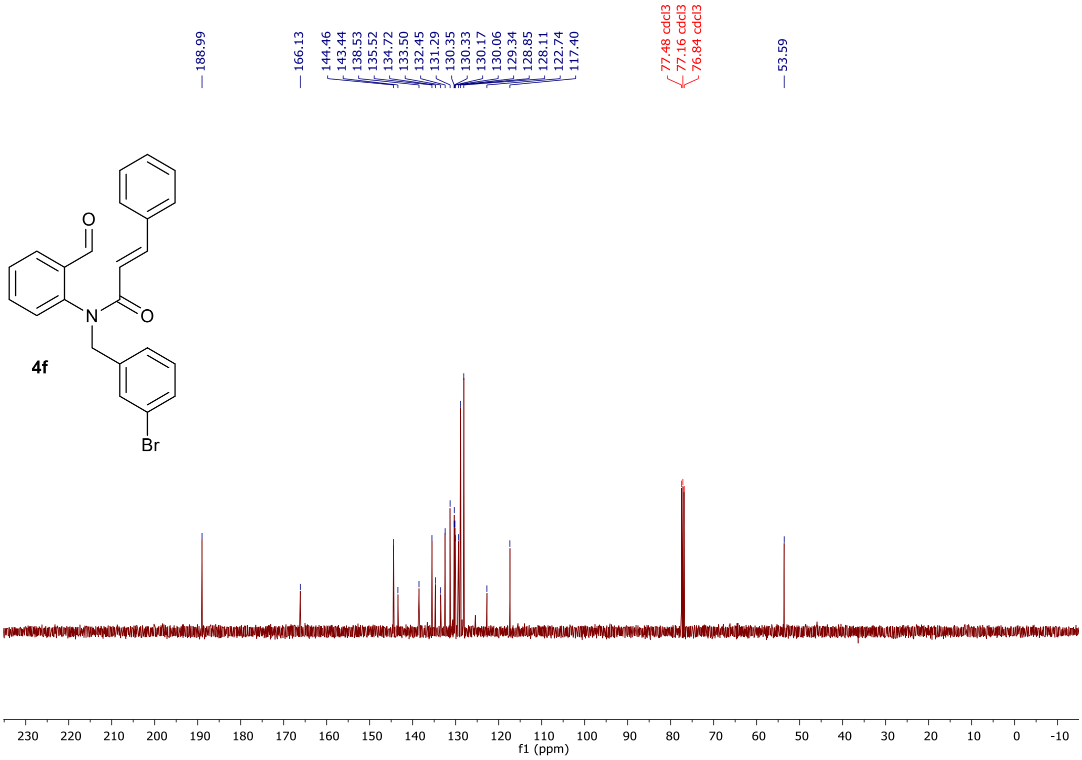

^{1}H NMR Spectrum of **4g** (400 MHz, $CDCl_3$)

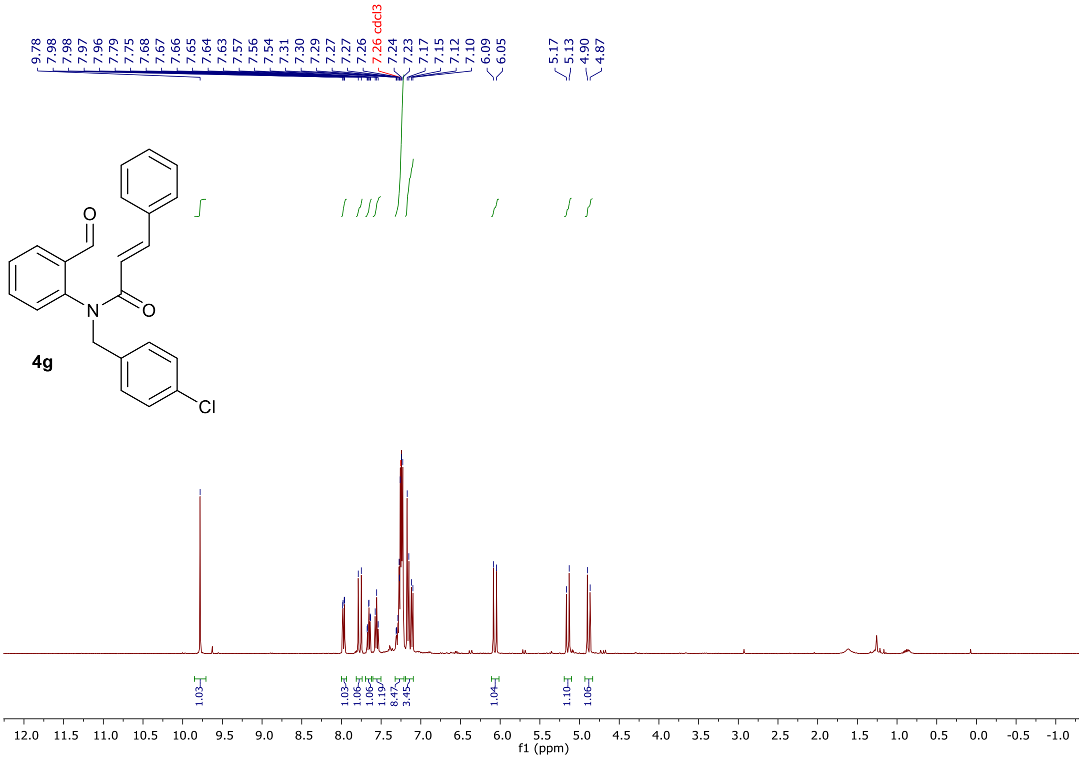

^{13}C NMR Spectrum of **4g** (101 MHz, $CDCl_3$)

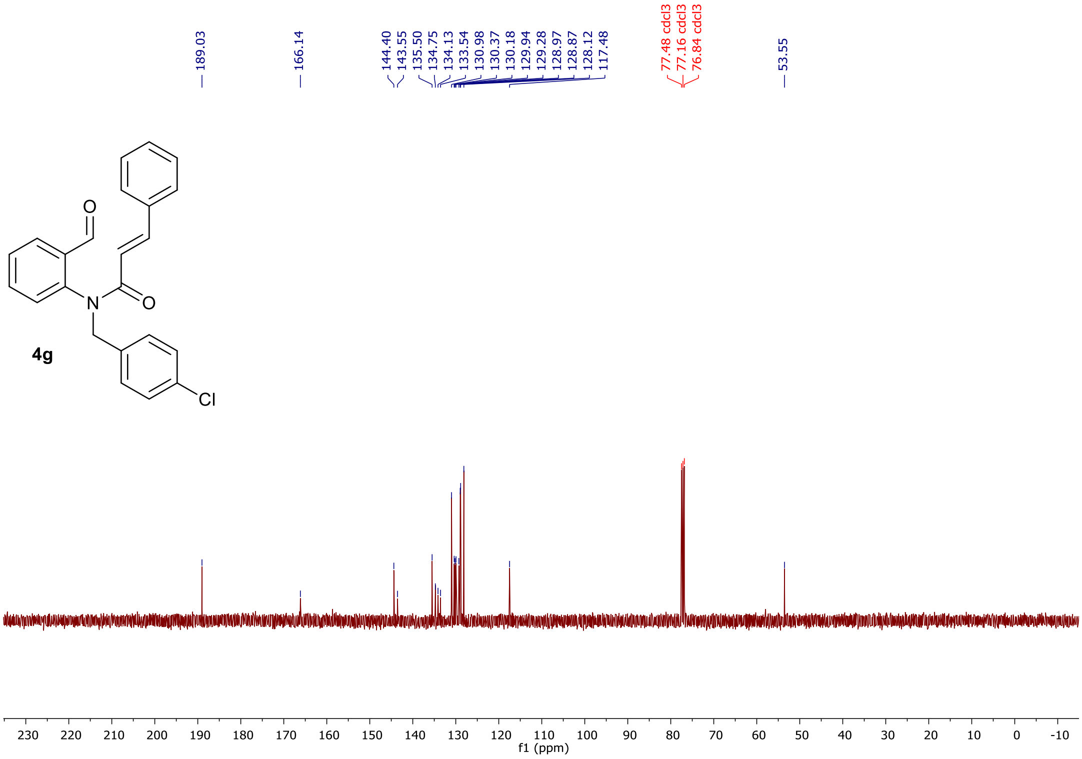

^{1}H NMR Spectrum of **4h** (400 MHz, $CDCl_3$)

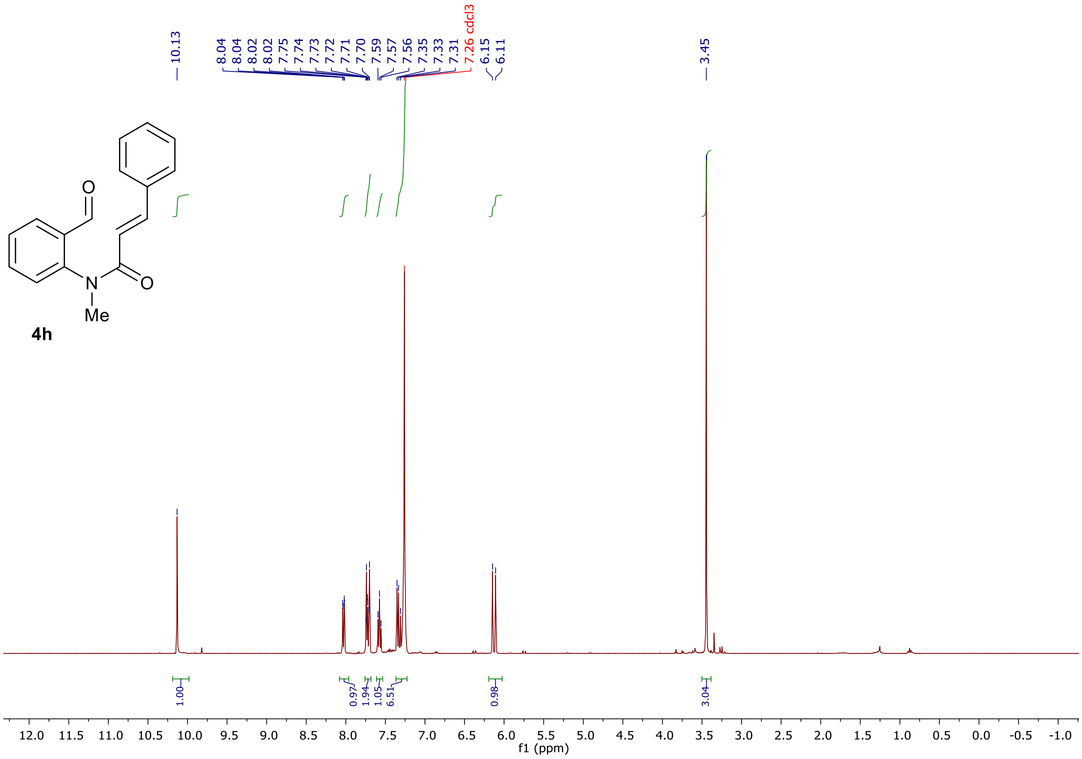

^{13}C NMR Spectrum of **4h** (101 MHz, $CDCl_3$)

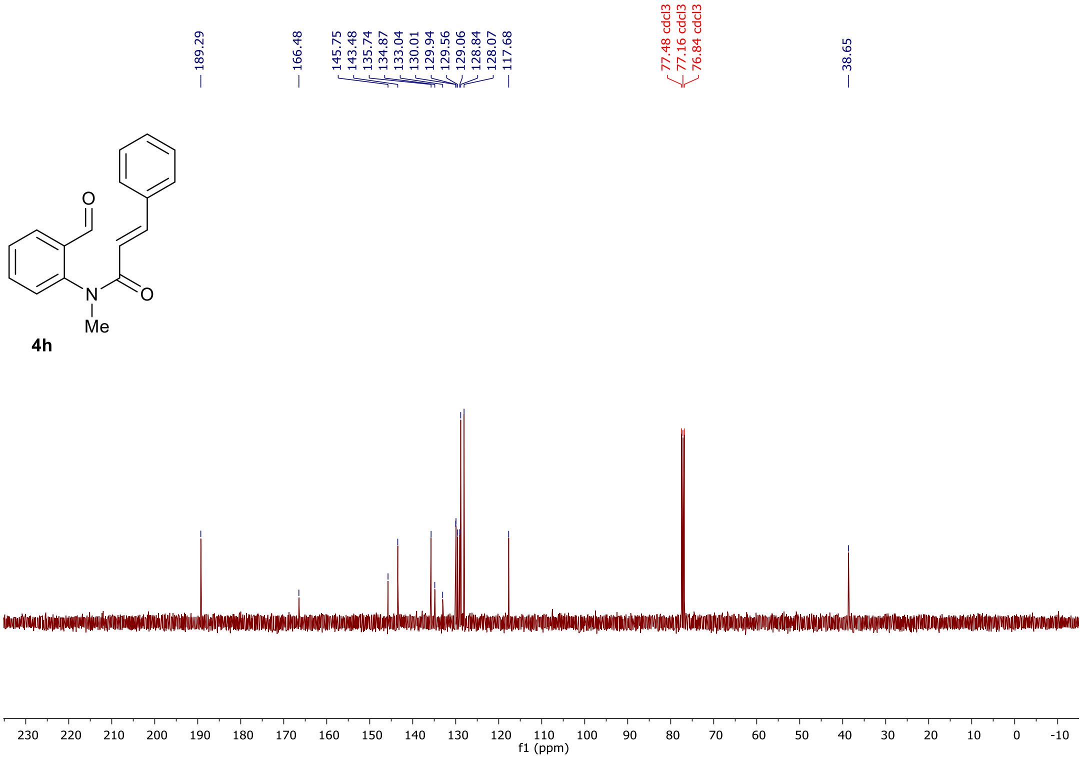

^{1}H NMR Spectrum of **4i** (400 MHz, $CDCl_3$)

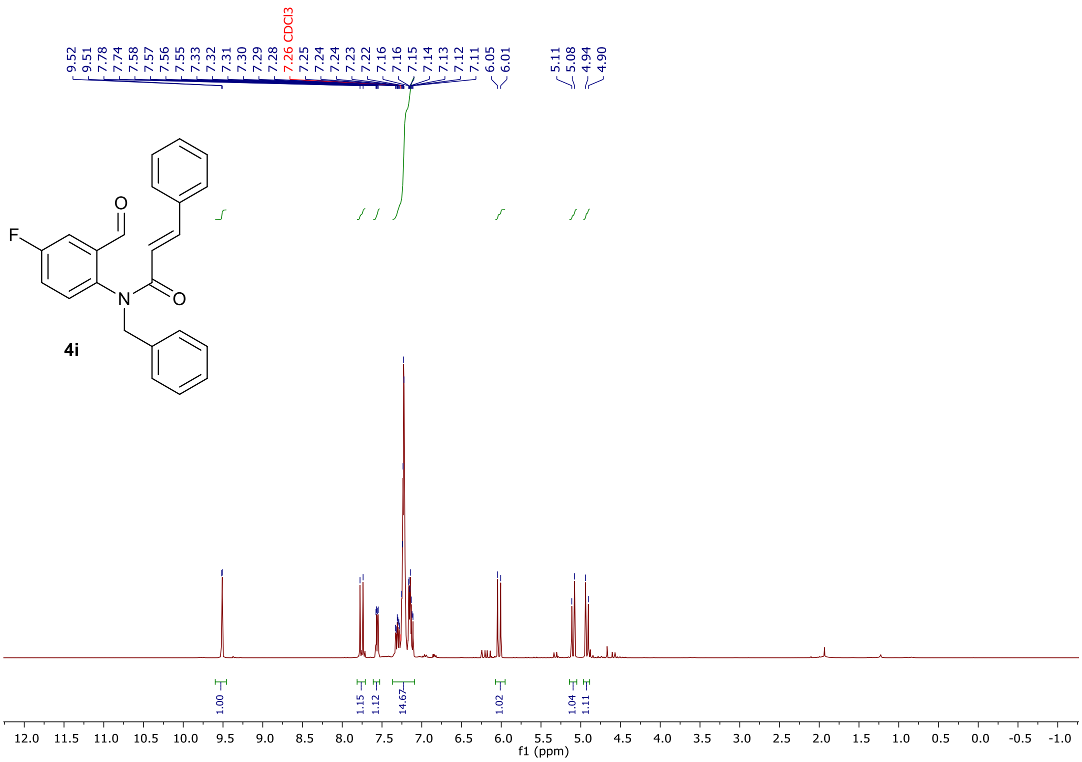

^{13}C NMR Spectrum of **4i** (101 MHz, $CDCl_3$)

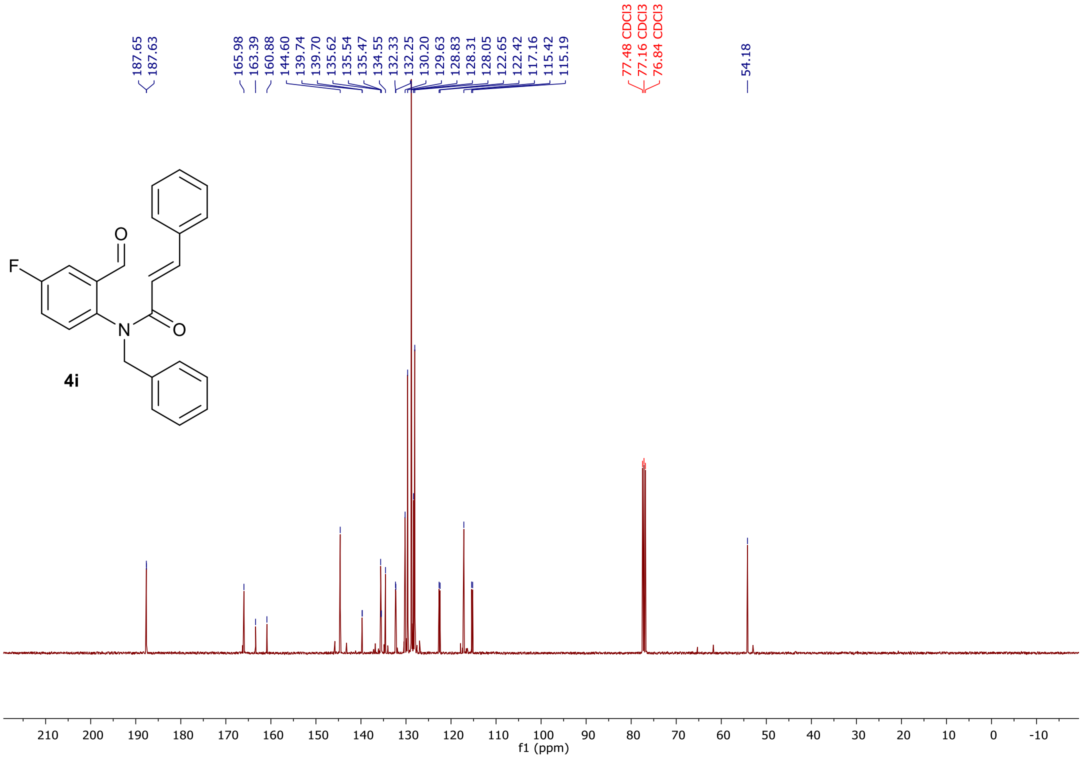

^{19}F NMR Spectrum of **4i** (377 MHz, $CDCl_3$)

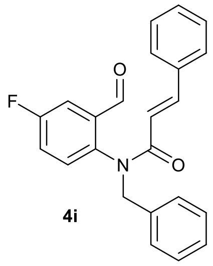

4i

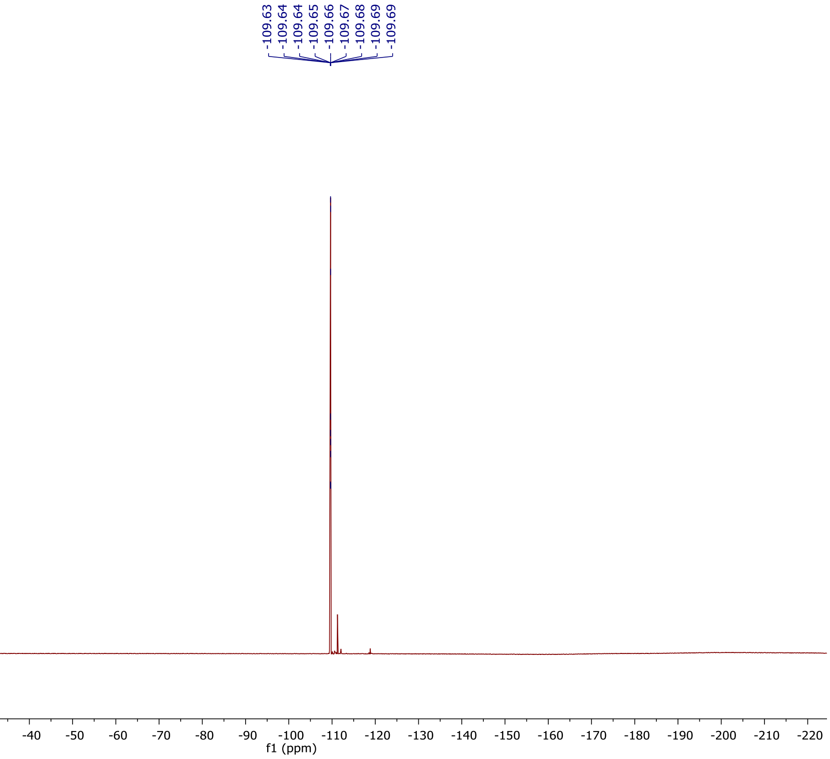

^{1}H NMR Spectrum of **4j** (400 MHz, $CDCl_3$)

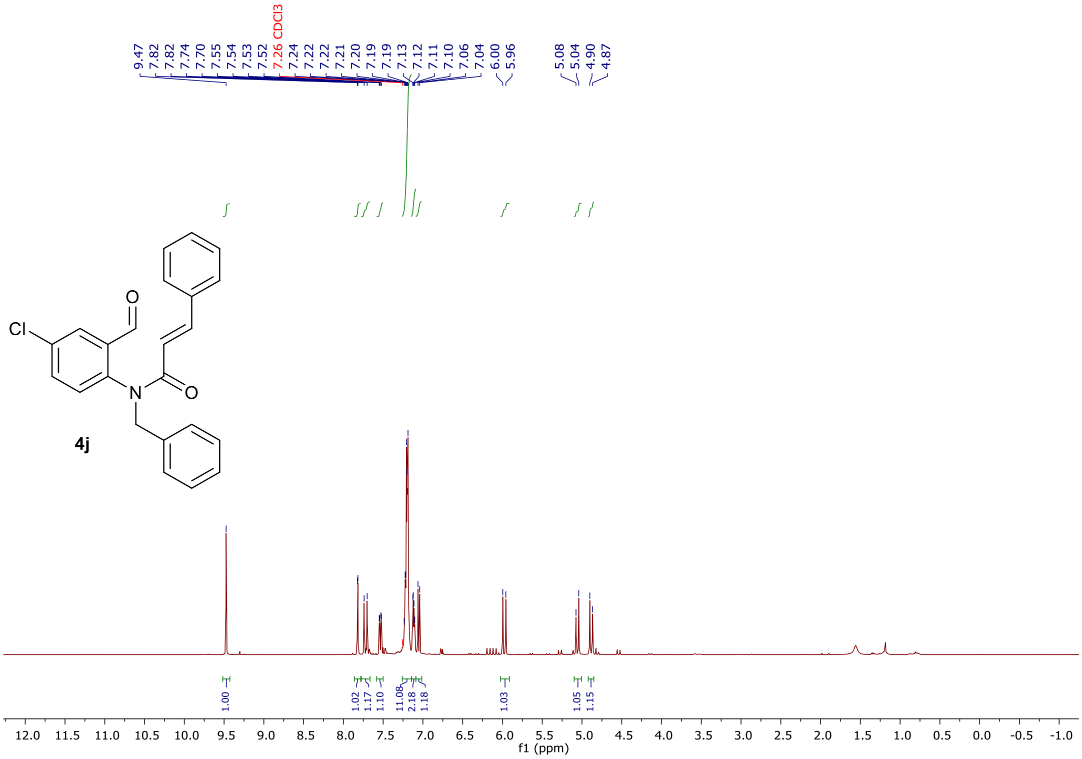

^{13}C NMR Spectrum of **4j** (126 MHz, $CDCl_3$)

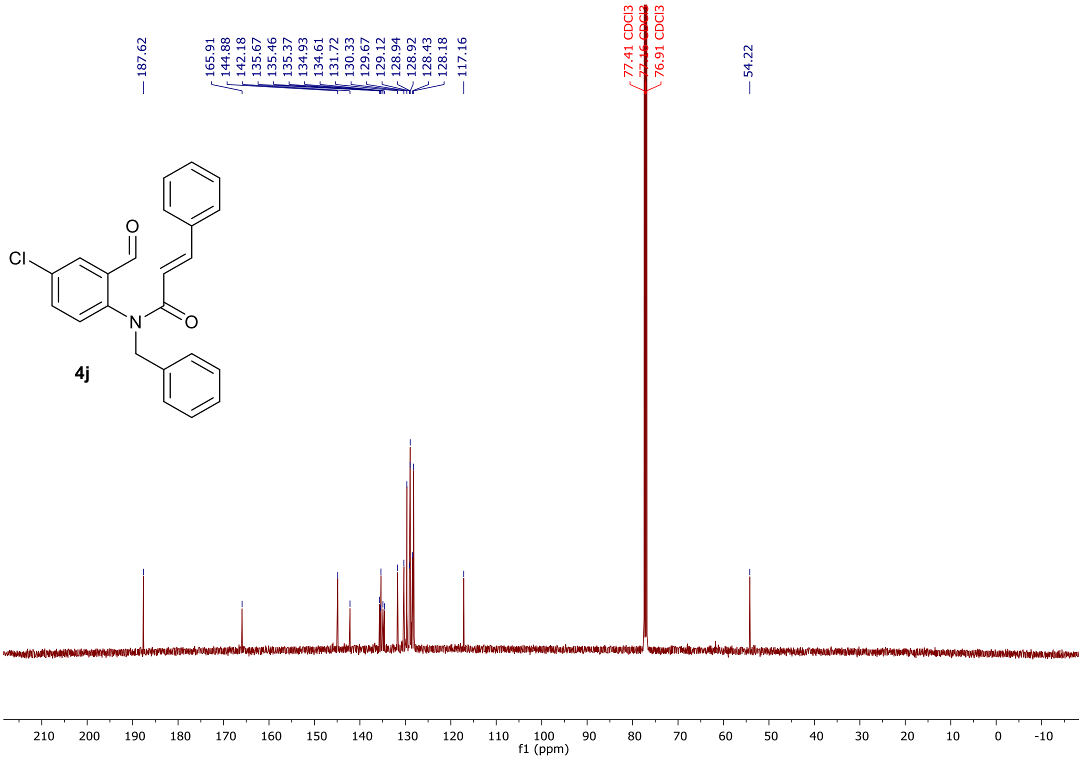

^{1}H NMR Spectrum of **4k** (400 MHz, $CDCl_3$)

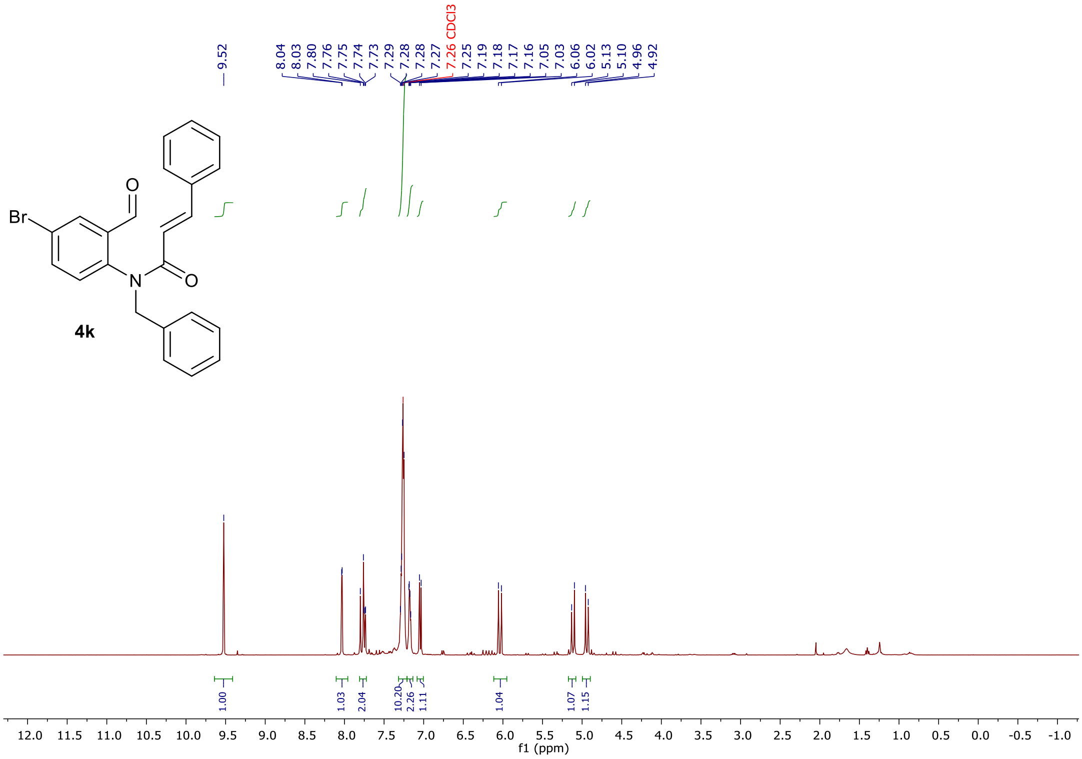

^{13}C NMR Spectrum of **4k** (101 MHz, $CDCl_3$)

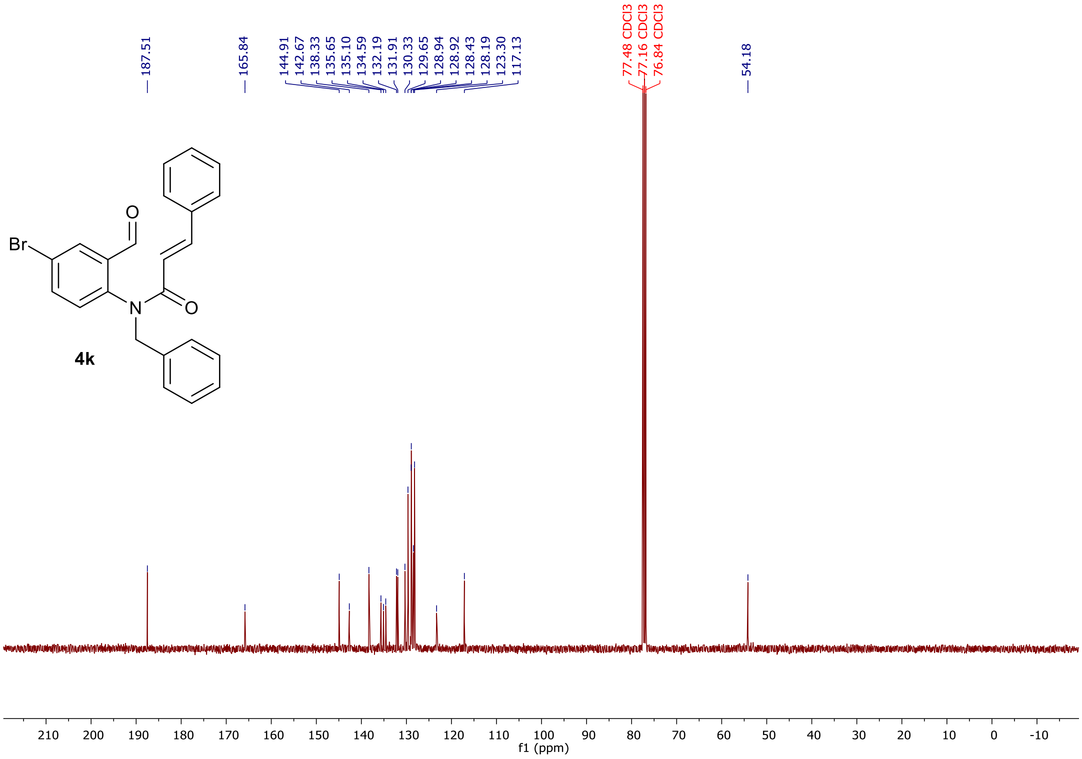

^{1}**H NMR** Spectrum of **4l** (400 MHz, $CDCl_3$)

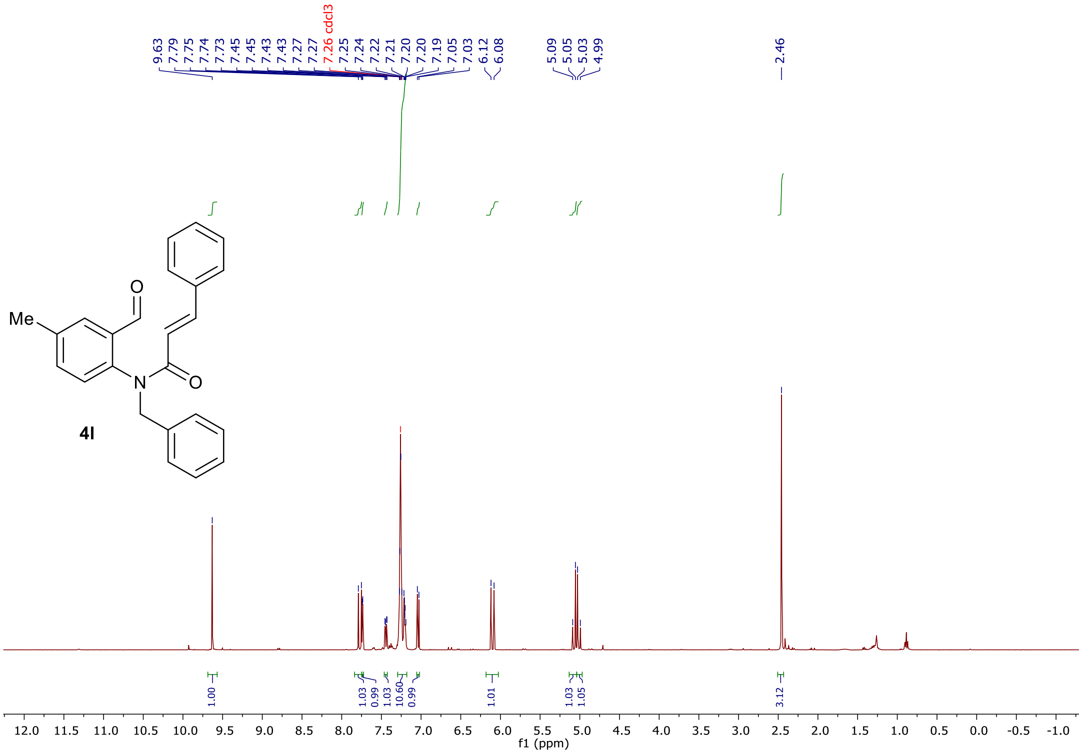

^{13}**C NMR** Spectrum of **4l** (101 MHz, $CDCl_3$)

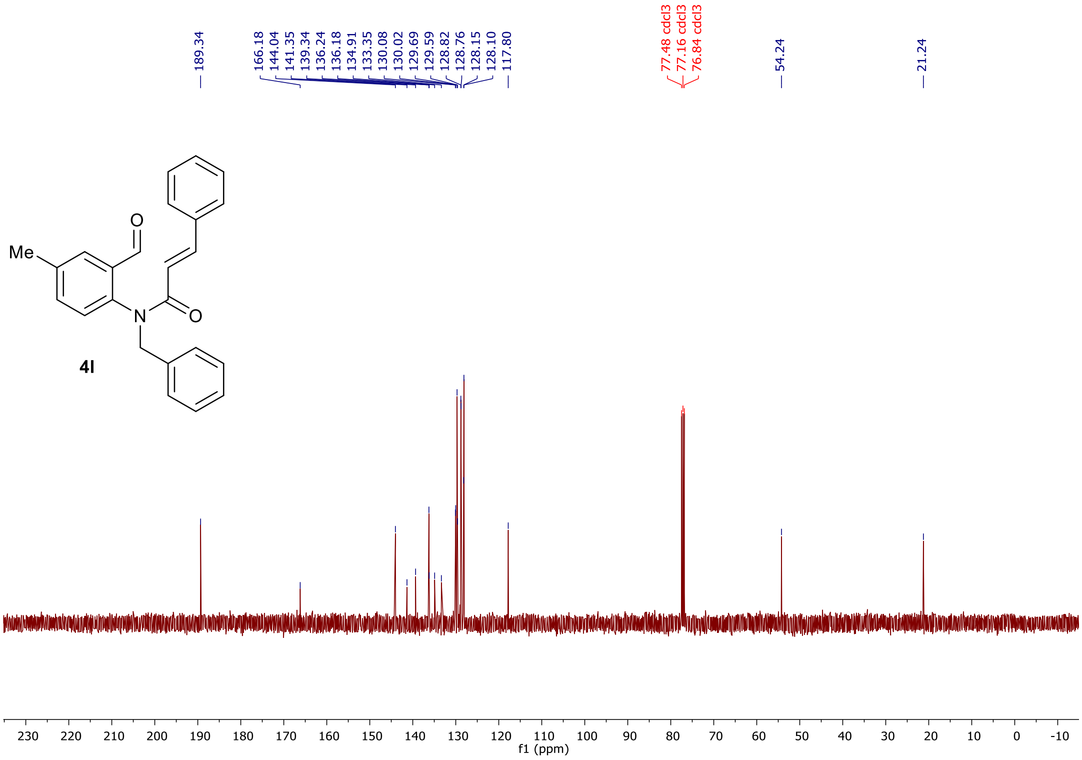

^{1}H NMR Spectrum of **4m** (500 MHz, $CDCl_3$)

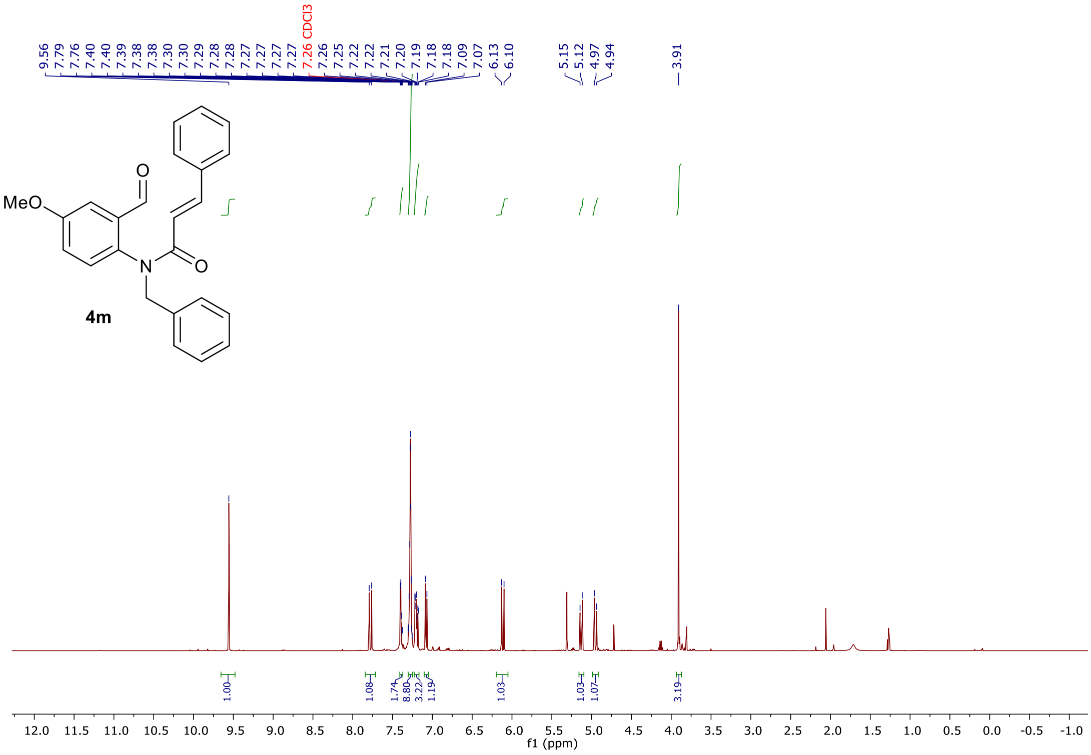

^{13}C NMR Spectrum of **4m** (126 MHz, $CDCl_3$)

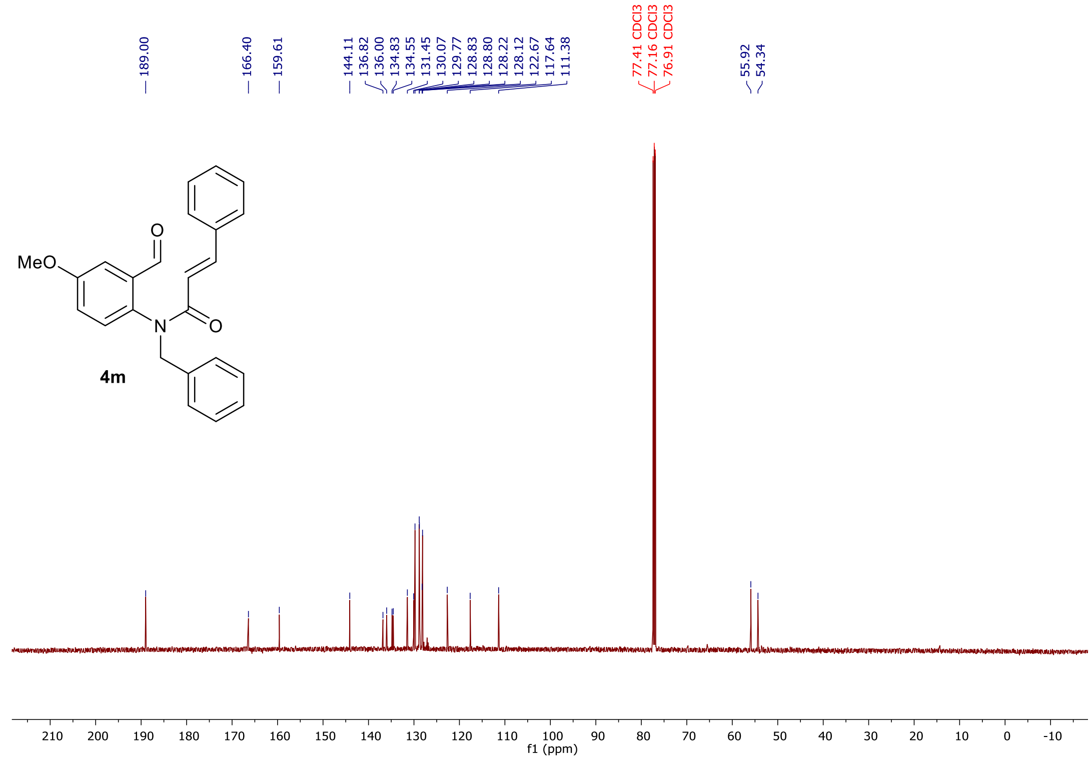

^{1}H NMR Spectrum of **4n** (500 MHz, $CDCl_3$)

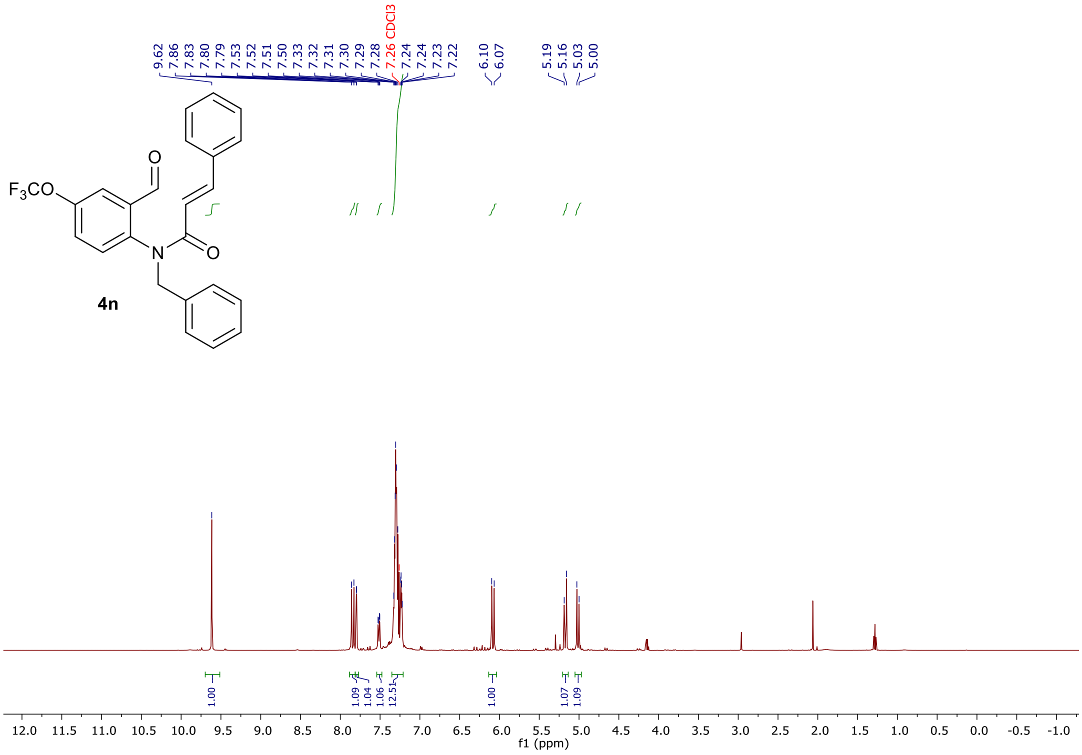

^{13}C NMR Spectrum of **4n** (126 MHz, $CDCl_3$)

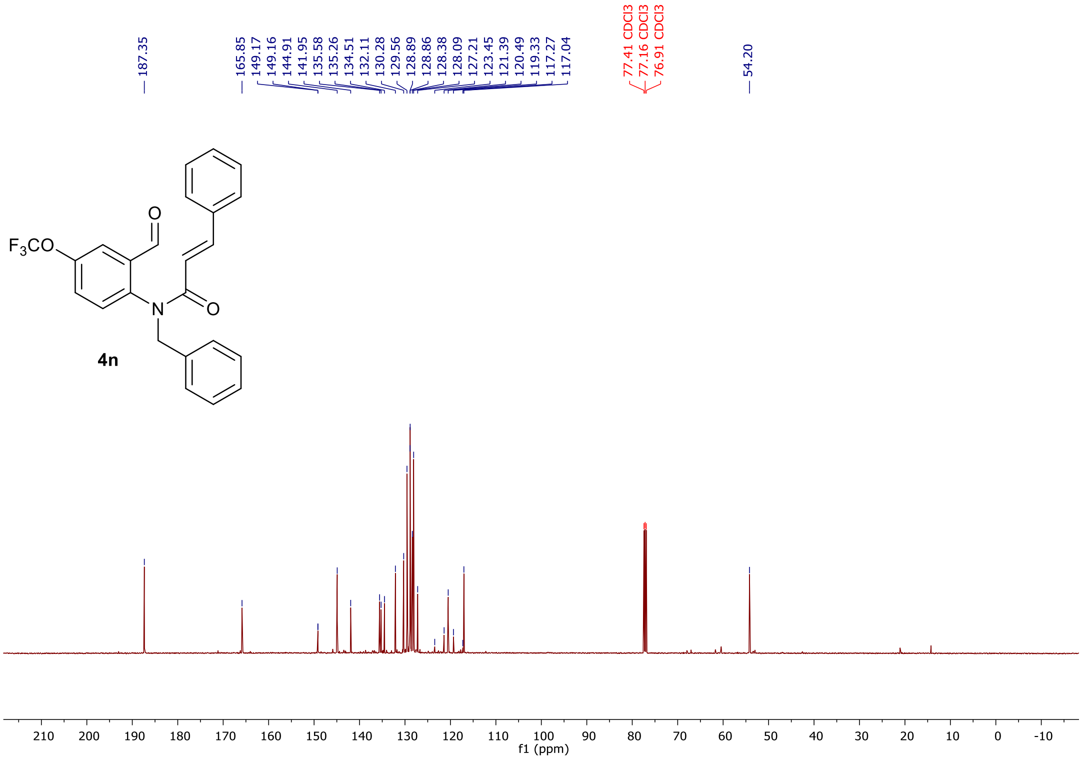

^{19}F NMR Spectrum of **4n** (470 MHz, $CDCl_3$)

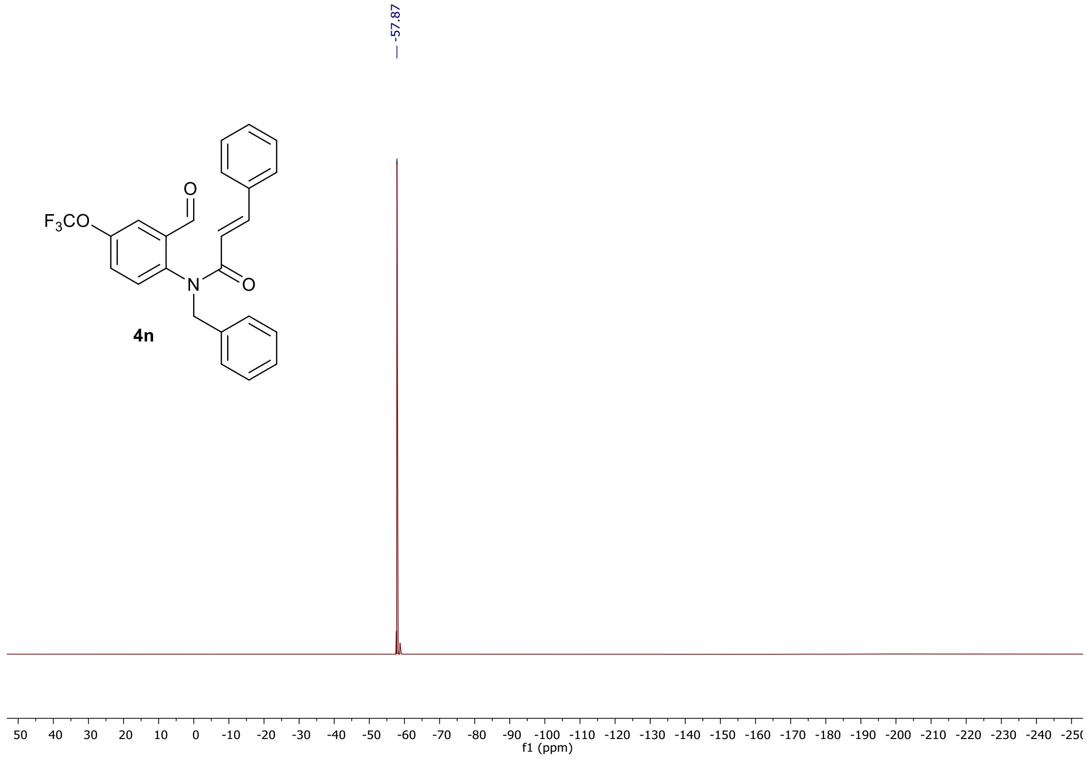

^{1}**H NMR** Spectrum of **4o** (500 MHz, $CDCl_3$)

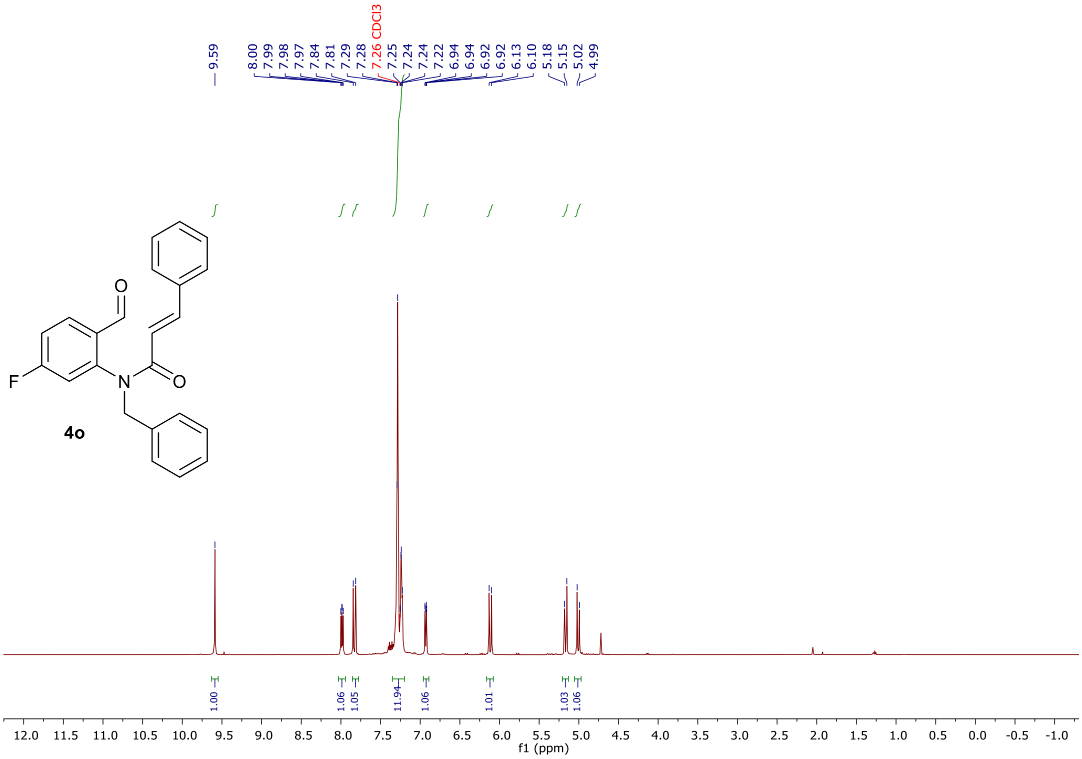

^{13}**C NMR** Spectrum of **4o** (126 MHz, $CDCl_3$)

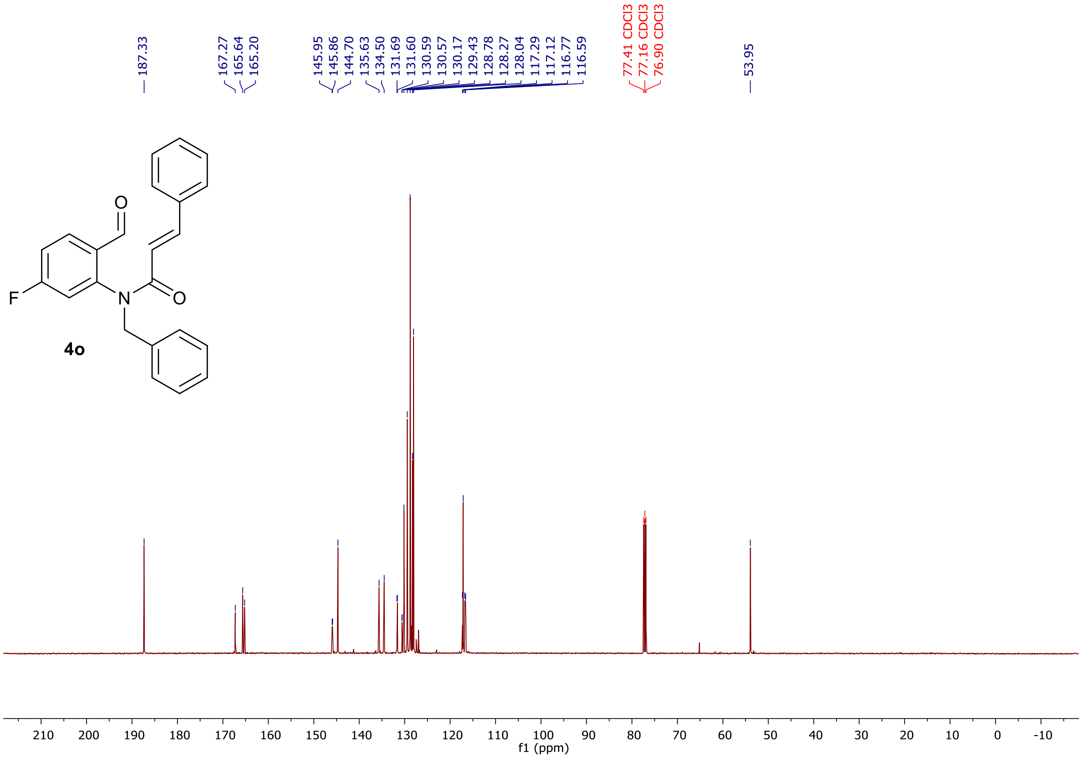

^{19}**F NMR** Spectrum of **4o** (470 MHz, $CDCl_3$)

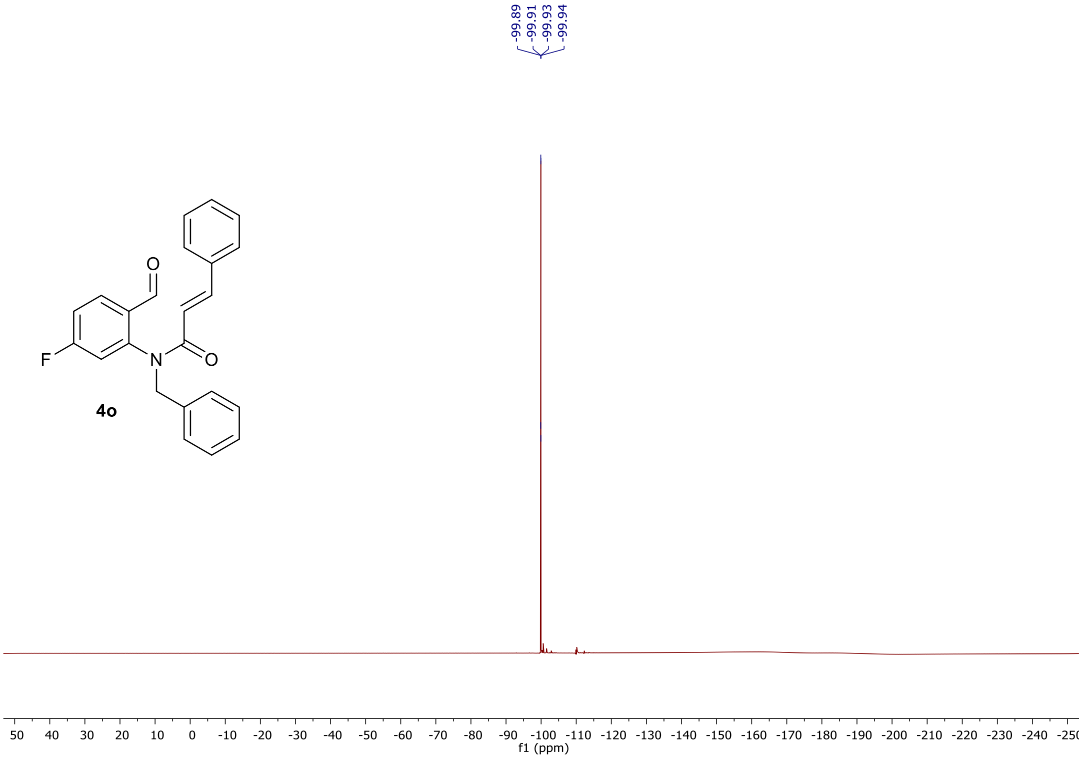

^{1}**H NMR** Spectrum of **4p** (400 MHz, $CDCl_3$)

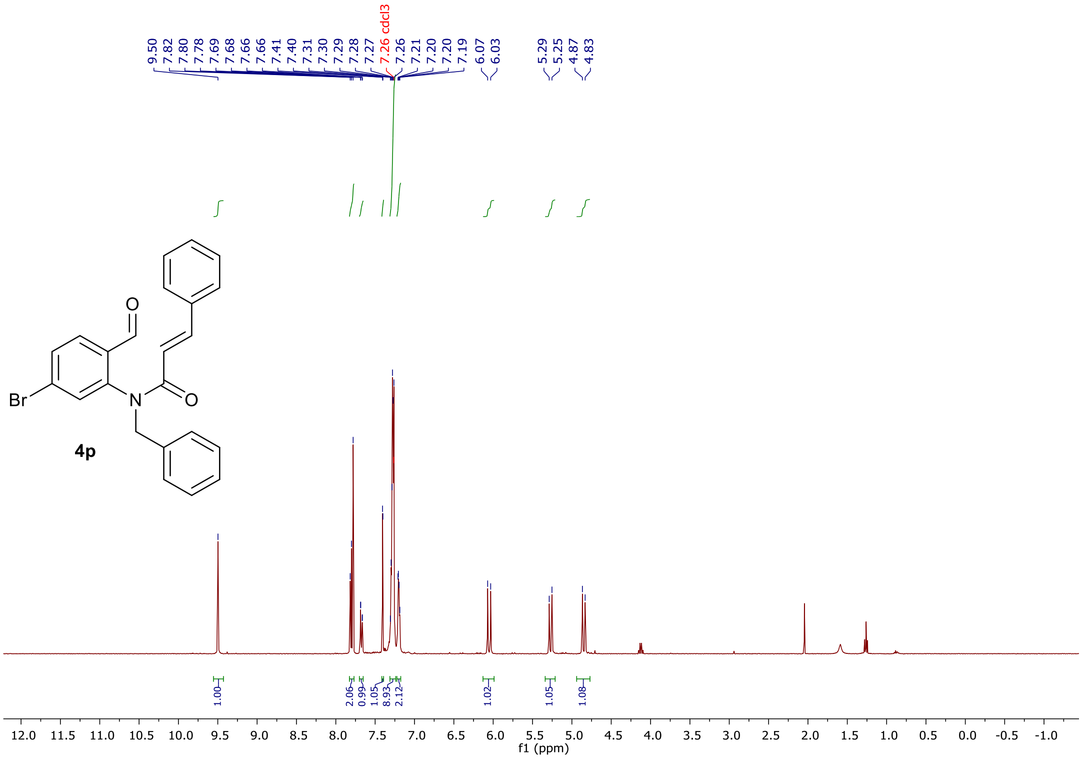

^{13}**C NMR** Spectrum of **4p** (126 MHz, $CDCl_3$)

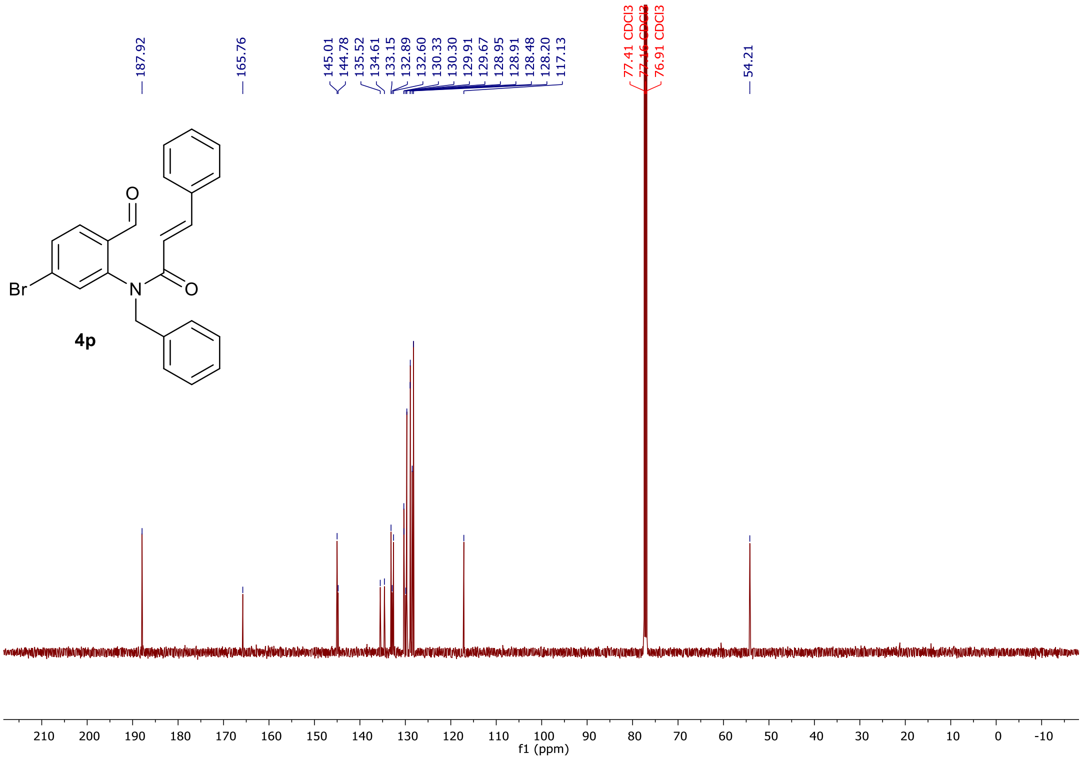

^{1}H NMR Spectrum of 4q (400 MHz, $CDCl_3$)

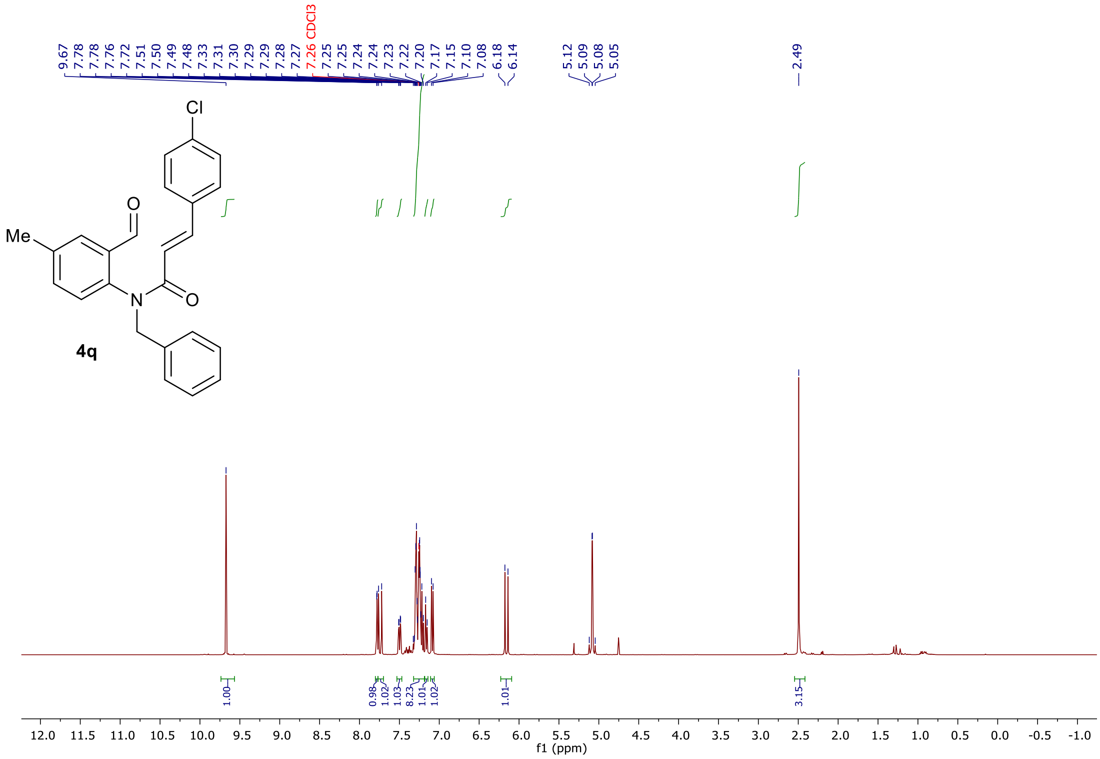

^{13}C NMR Spectrum of 4q (101 MHz, $CDCl_3$)

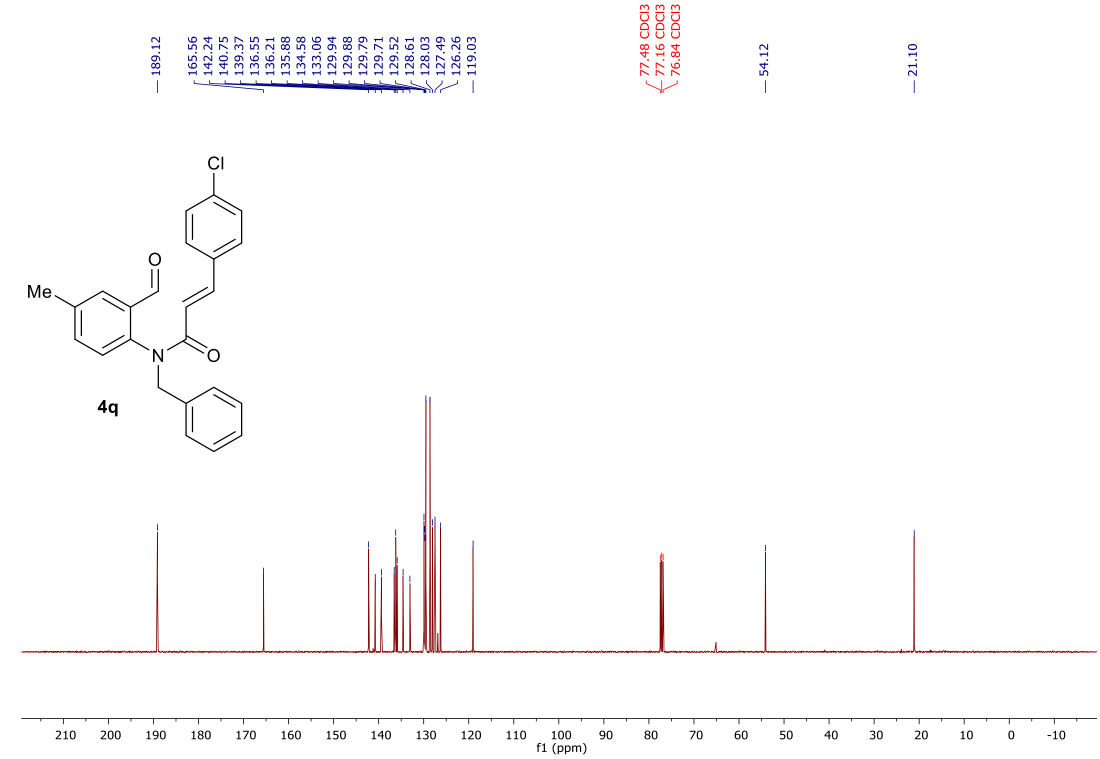

^{1}H NMR Spectrum of **4r** (400 MHz, $CDCl_3$)

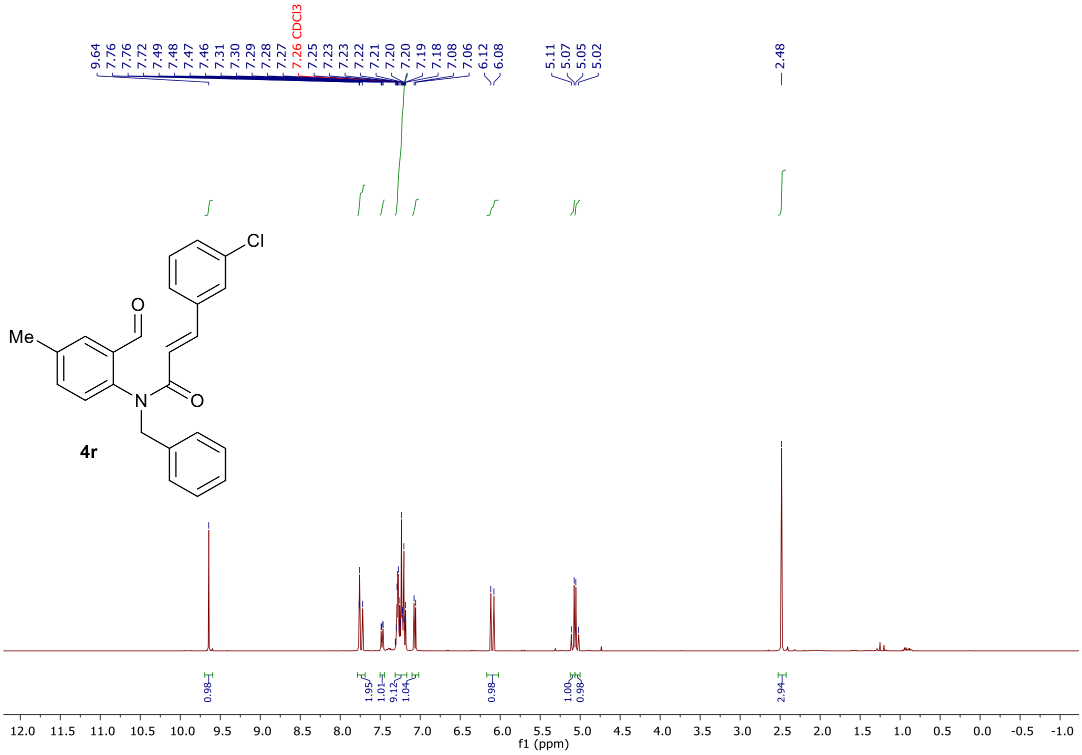

^{13}C NMR Spectrum of **4r** (101 MHz, $CDCl_3$)

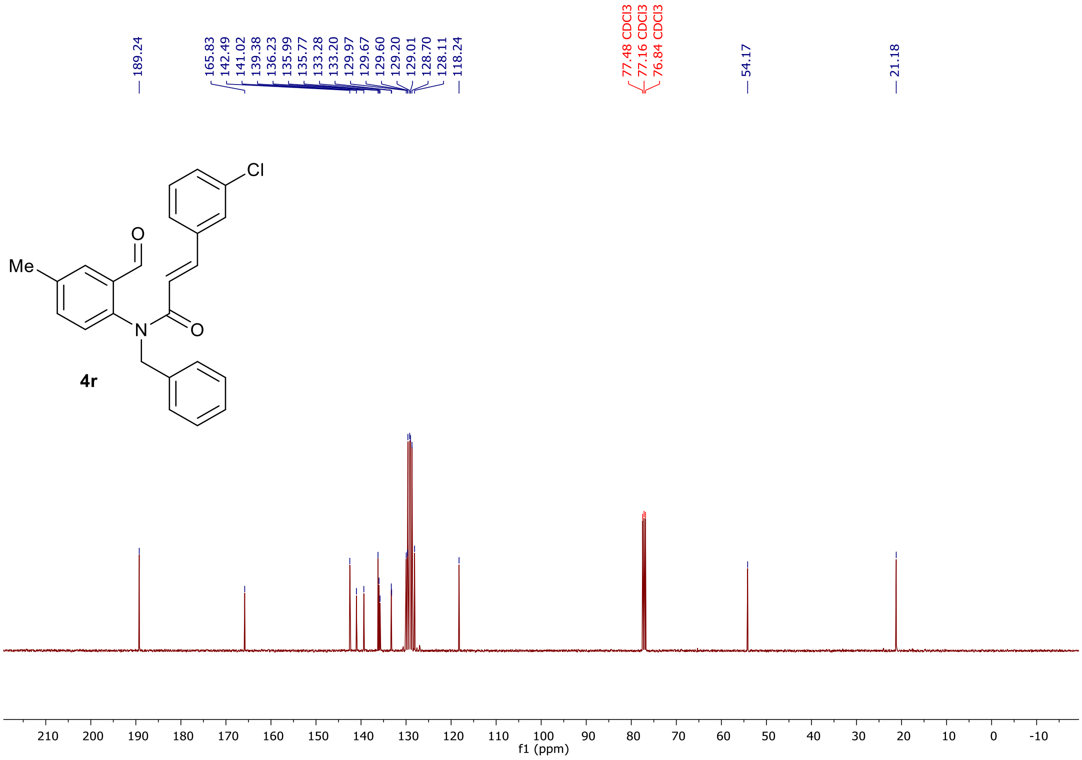

^{1}H NMR Spectrum of 4s (400 MHz, $CDCl_3$)

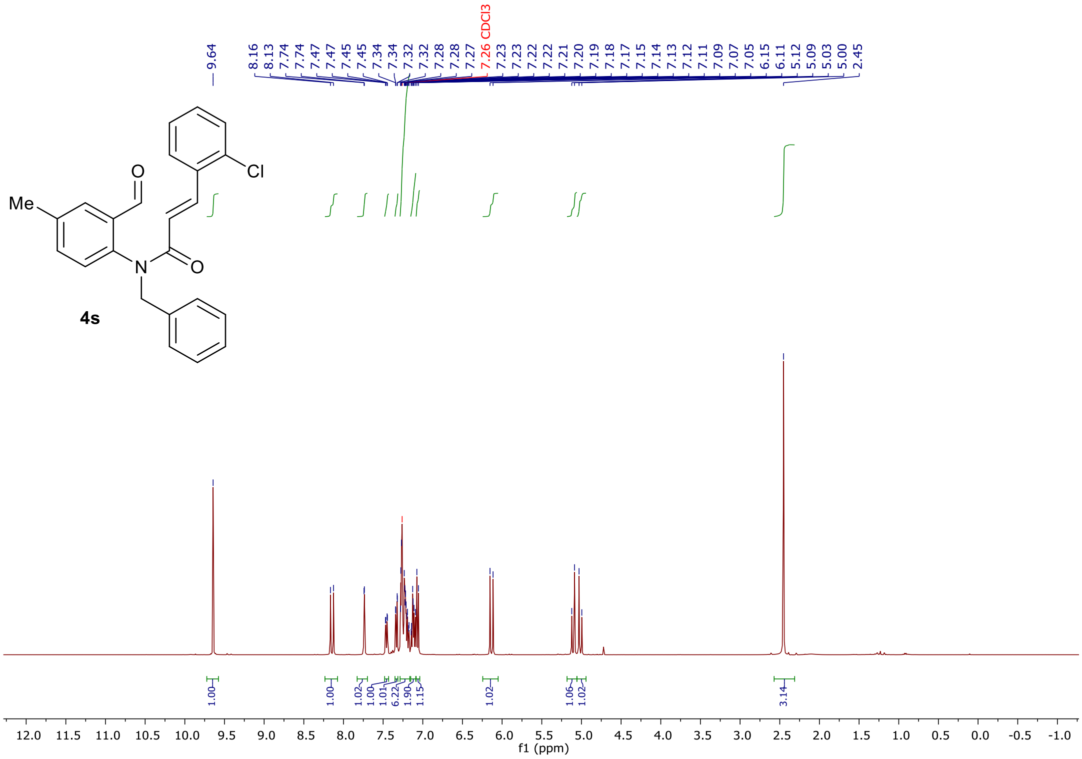

^{13}C NMR Spectrum of 4s (101 MHz, $CDCl_3$)

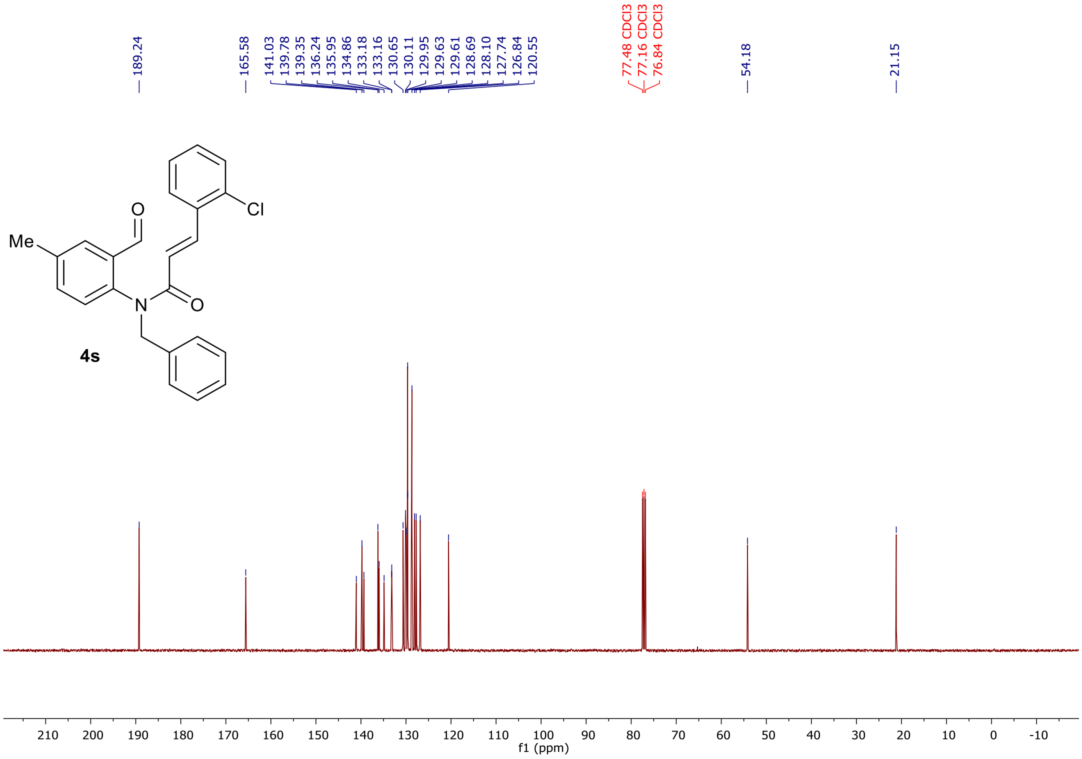

^{1}H NMR Spectrum of 4t (500 MHz, $CDCl_3$)

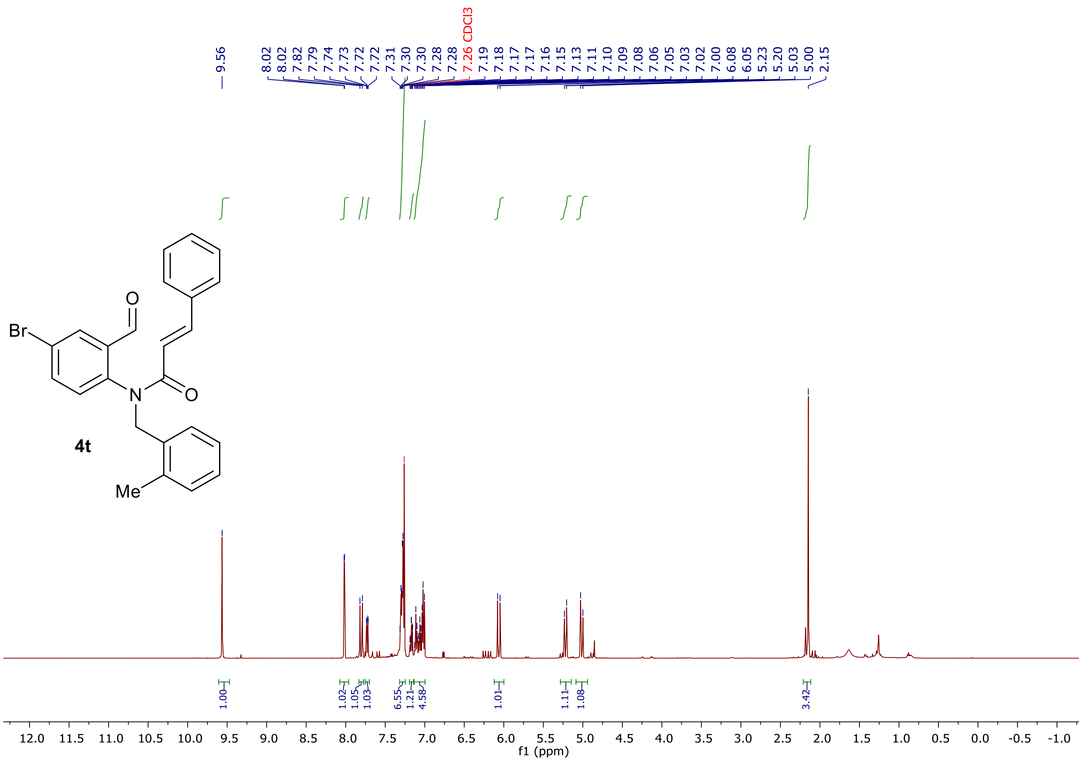

^{13}C NMR Spectrum of 4t (126 MHz, $CDCl_3$)

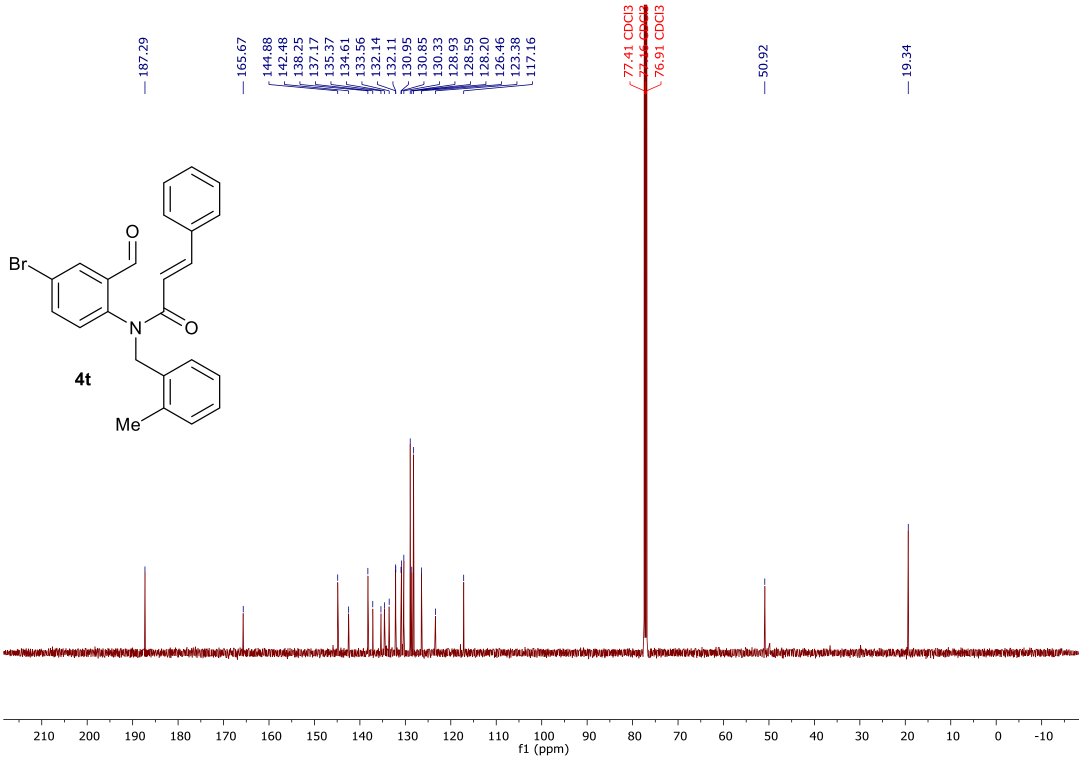

^{1}H NMR Spectrum of **4u** (400 MHz, $CDCl_3$)

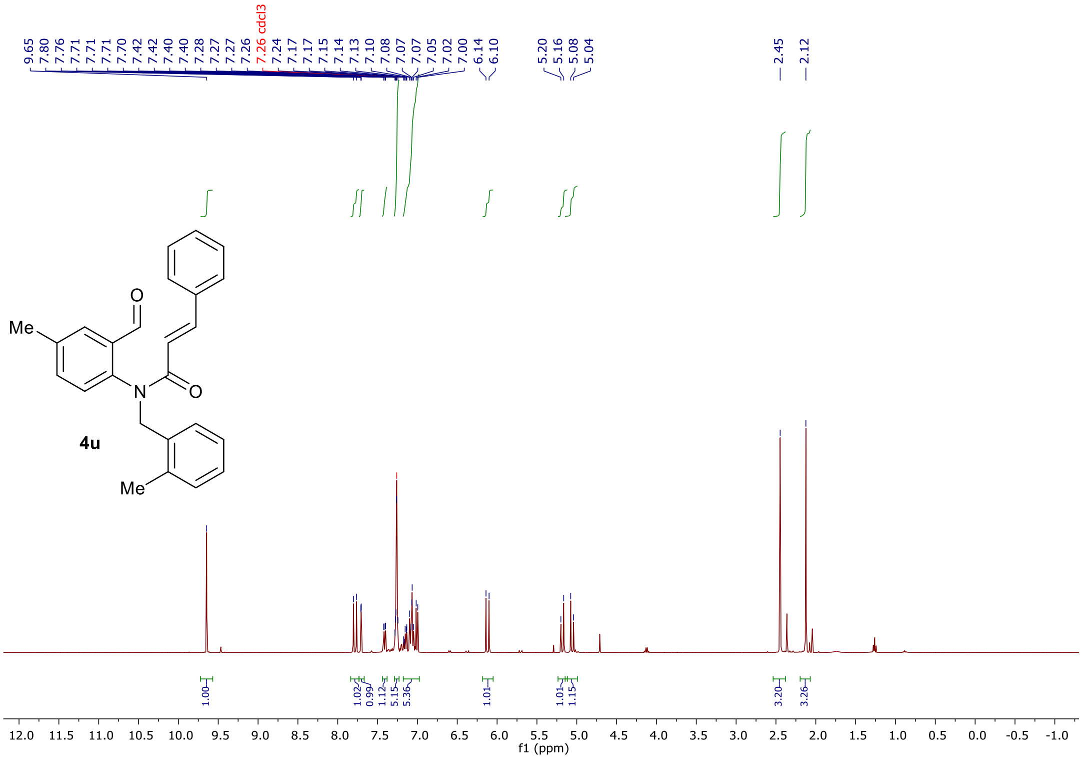

^{13}C NMR Spectrum of **4u** (101 MHz, $CDCl_3$)

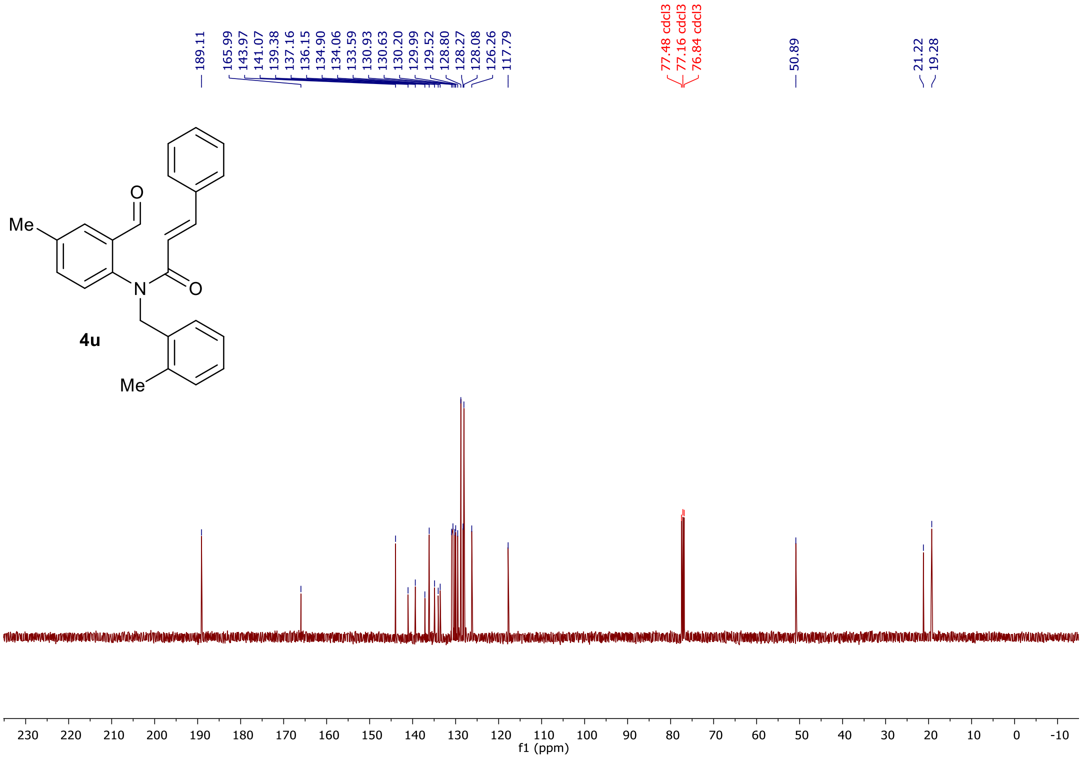

^{1}H NMR Spectrum of **4v** (400 MHz, $CDCl_3$)

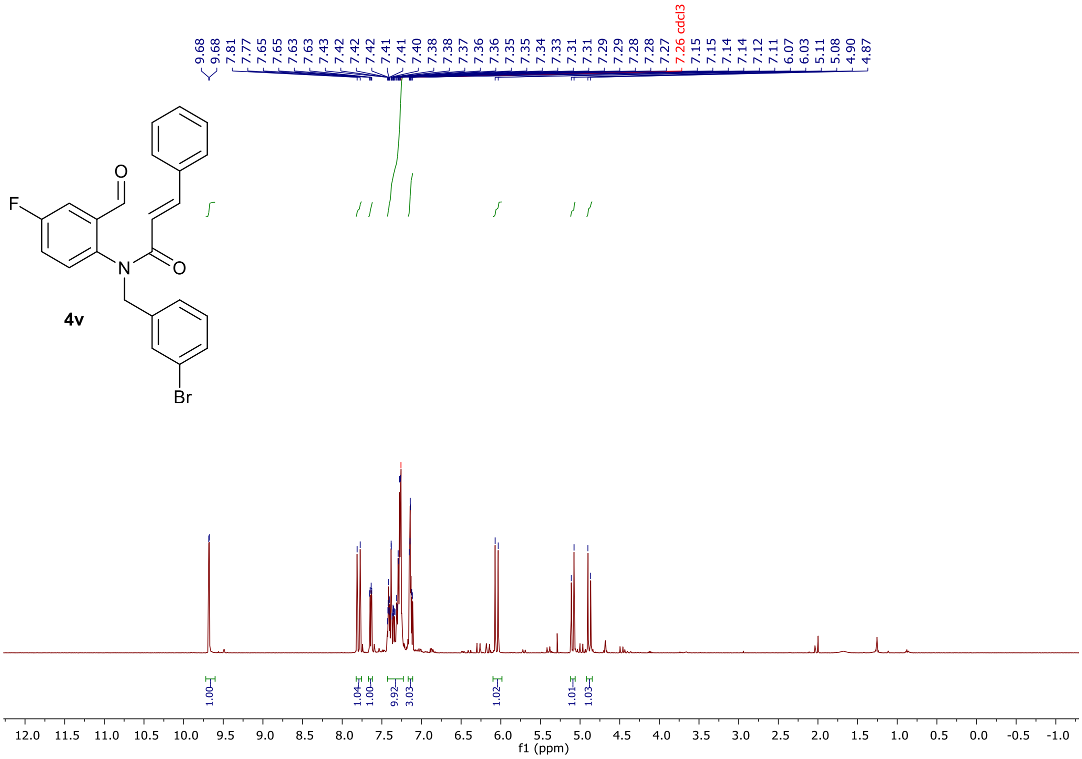

^{13}C NMR Spectrum of **4v** (101 MHz, $CDCl_3$)

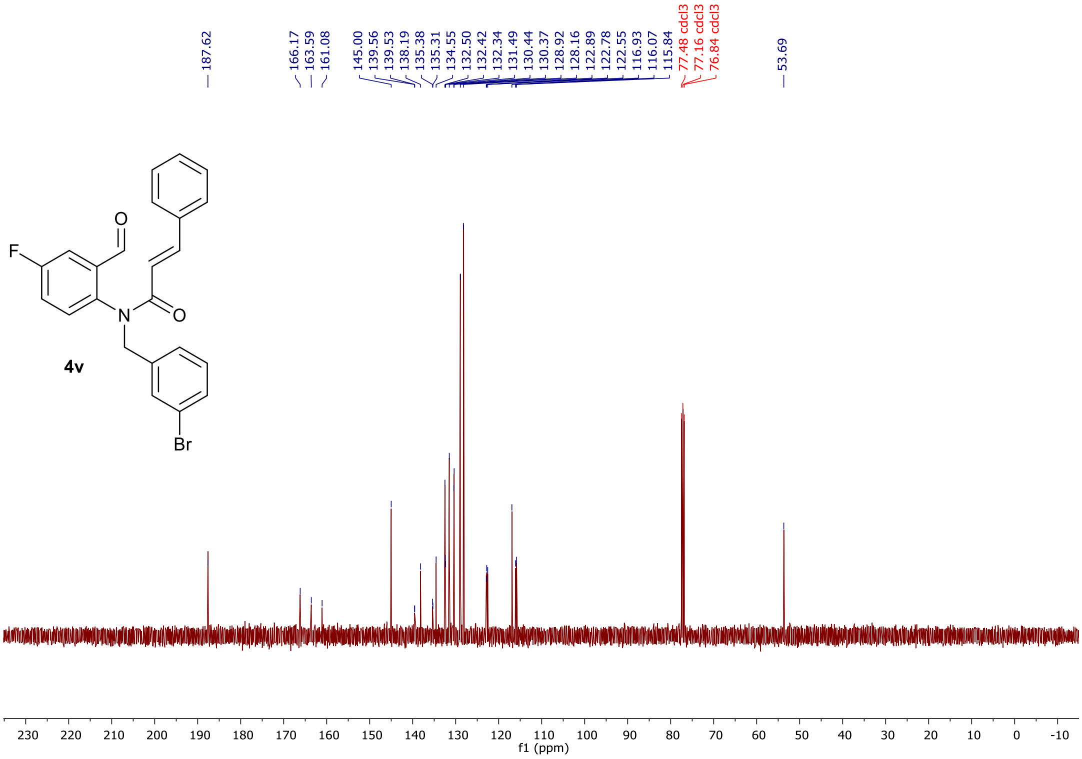

^{19}F NMR Spectrum of **4v** (470 MHz, $CDCl_3$)

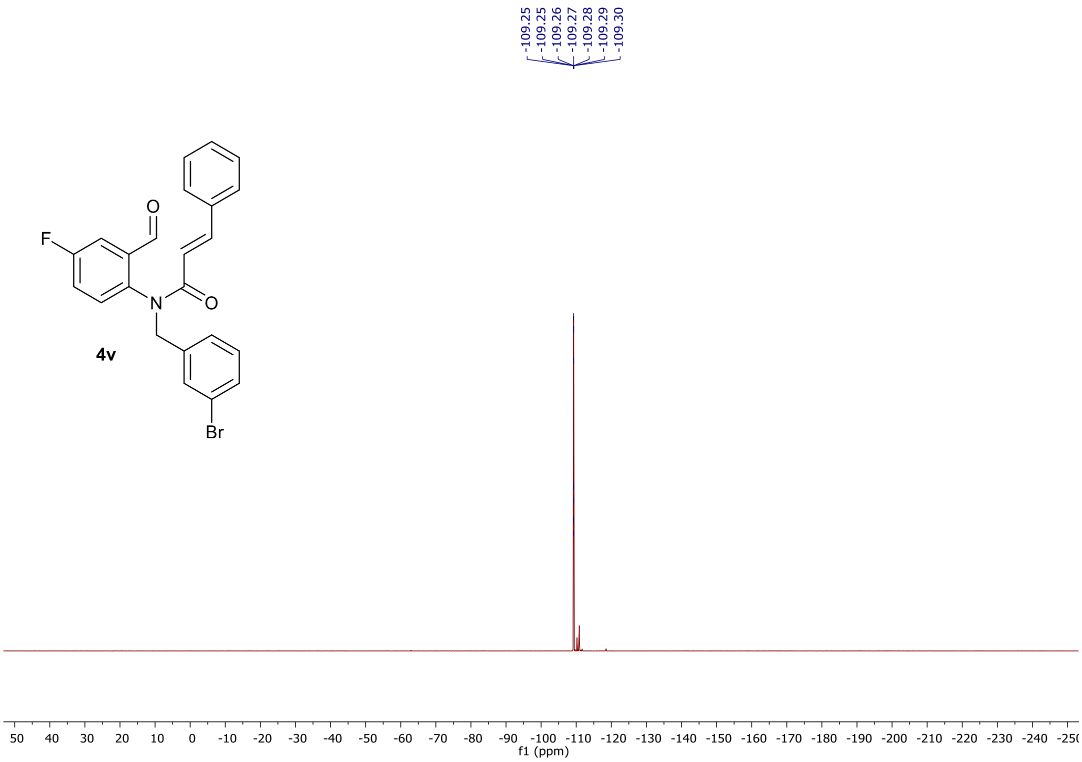

^{1}H NMR Spectrum of 4w (400 MHz, $CDCl_3$)

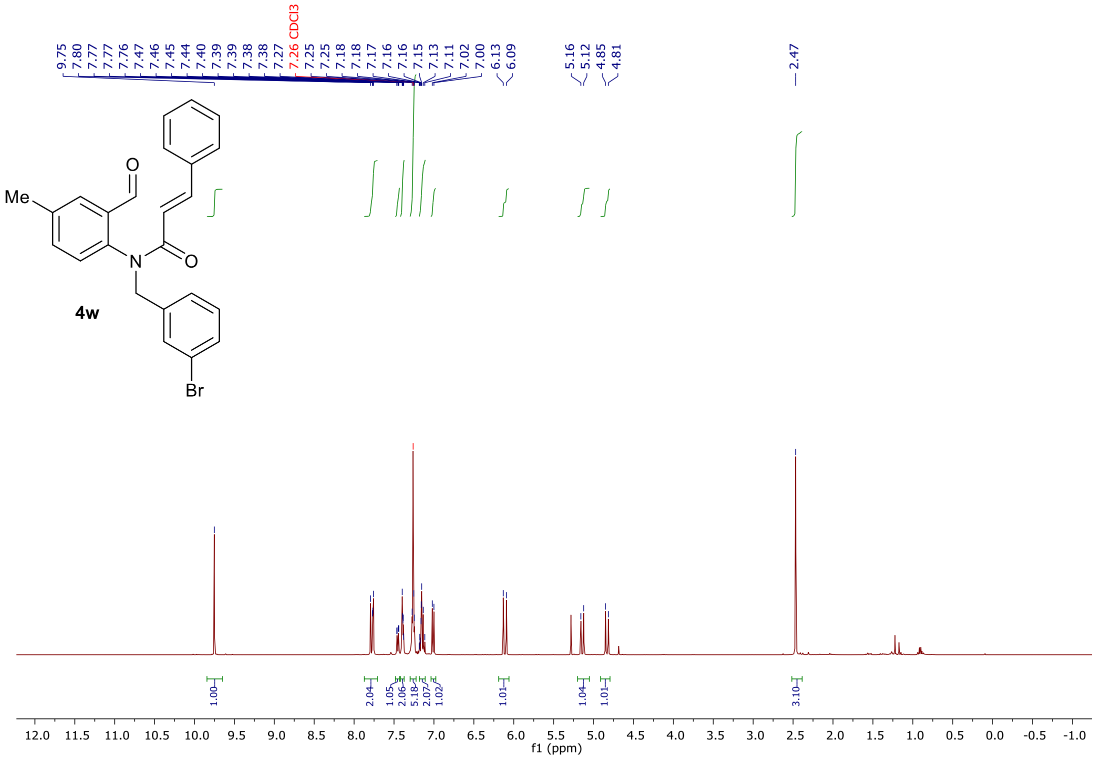

^{13}C NMR Spectrum of 4w (101 MHz, $CDCl_3$)

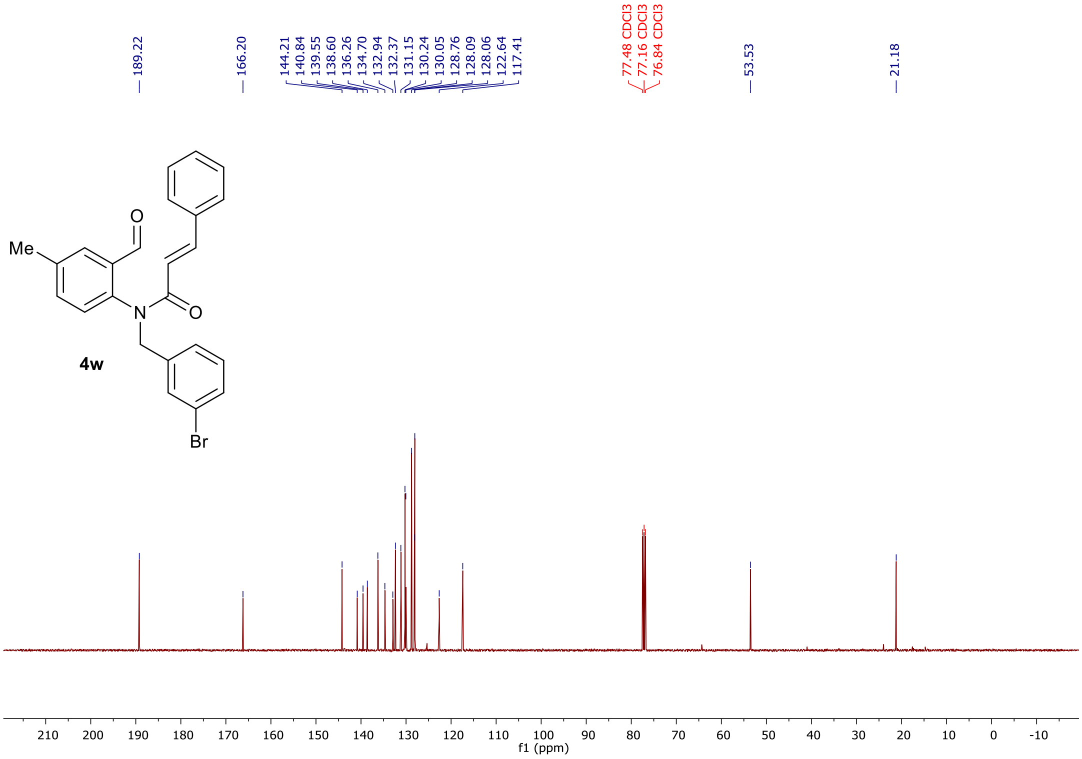

^{1}H NMR Spectrum of 4x (400 MHz, $CDCl_3$)

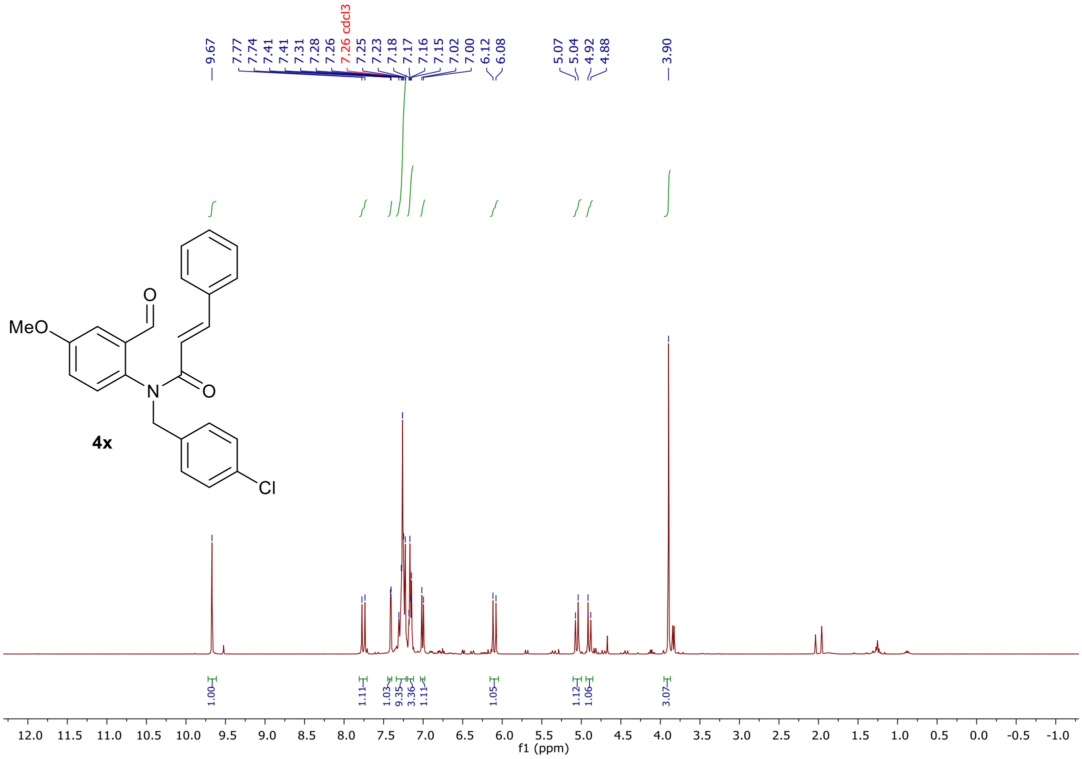

^{13}C NMR Spectrum of 4x (101 MHz, $CDCl_3$)

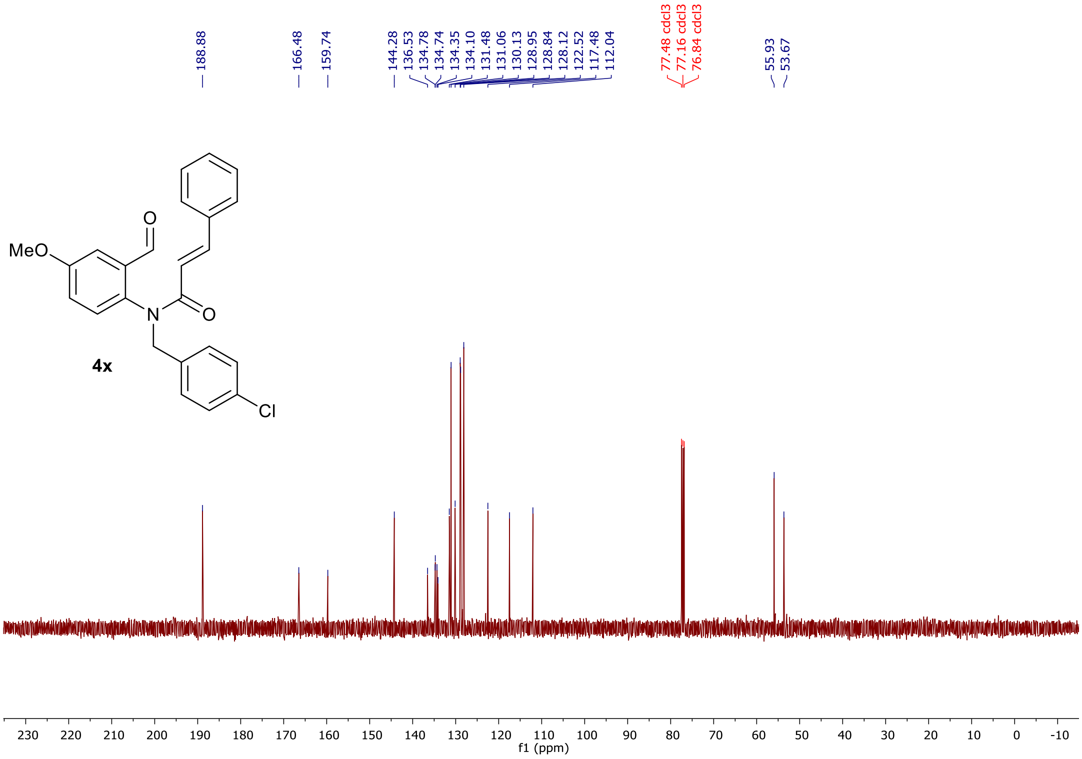

^{1}H NMR Spectrum of **4y** (400 MHz, $CDCl_3$)

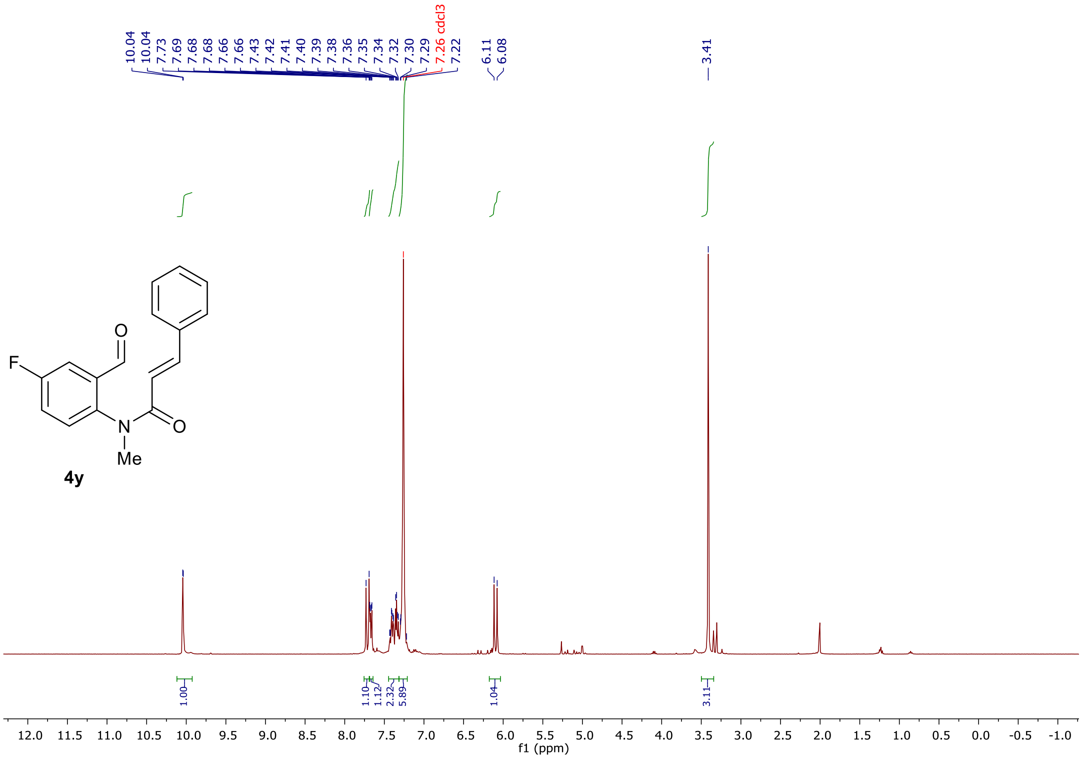

^{13}C NMR Spectrum of **4y** (101 MHz, $CDCl_3$)

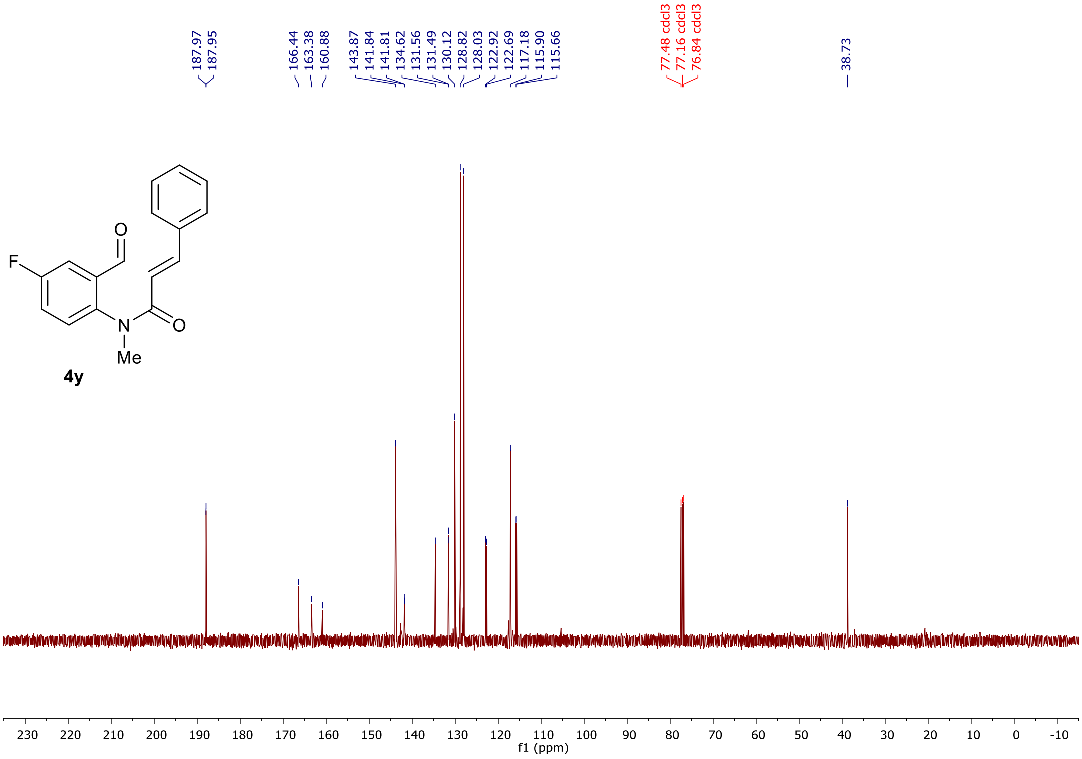

^{19}**F NMR** Spectrum of **4y** (470 MHz, $CDCl_3$)

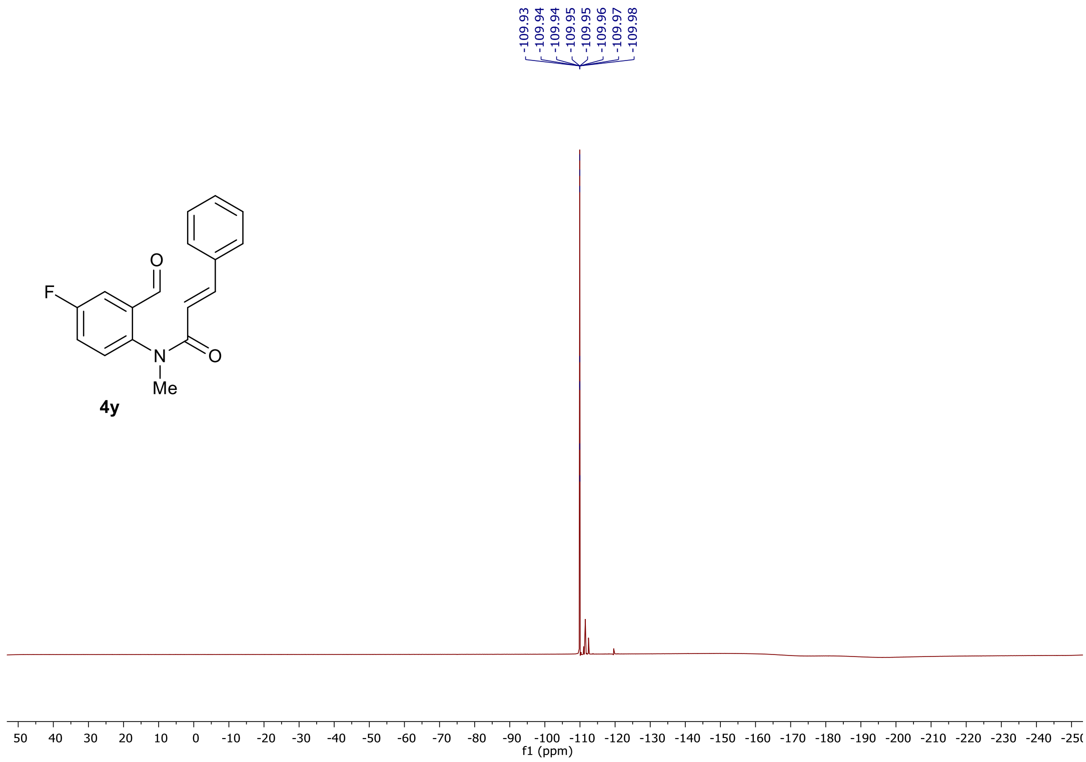

^{1}H NMR Spectrum of **4z** (400 MHz, $CDCl_3$)

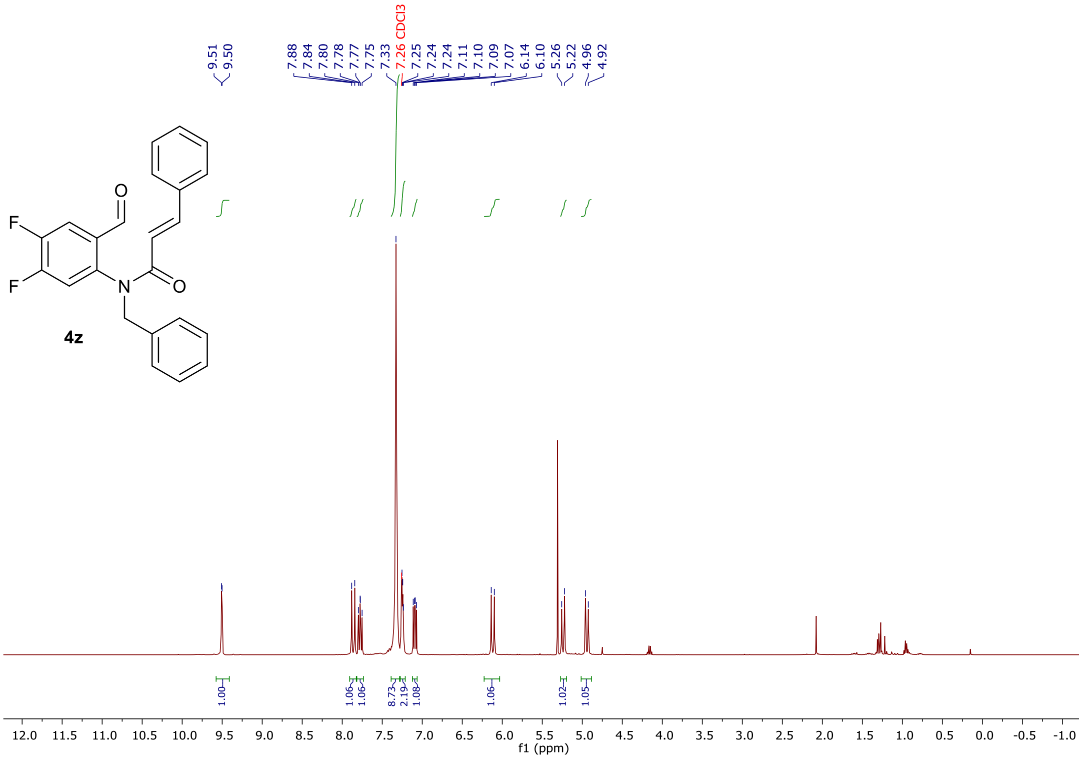

^{13}C NMR Spectrum of **4z** (101 MHz, $CDCl_3$)

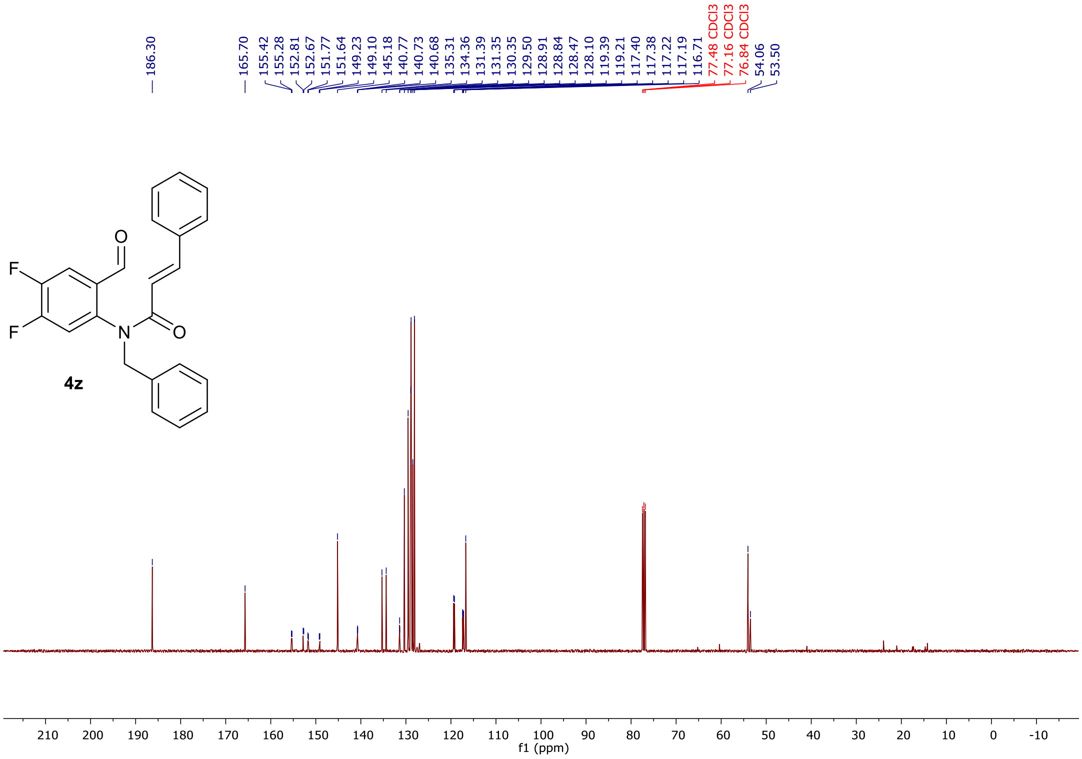

^{19}F NMR Spectrum of **4z** (377 MHz, $CDCl_3$)

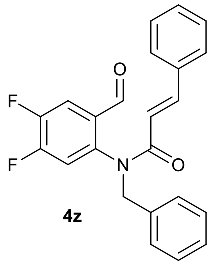

4z

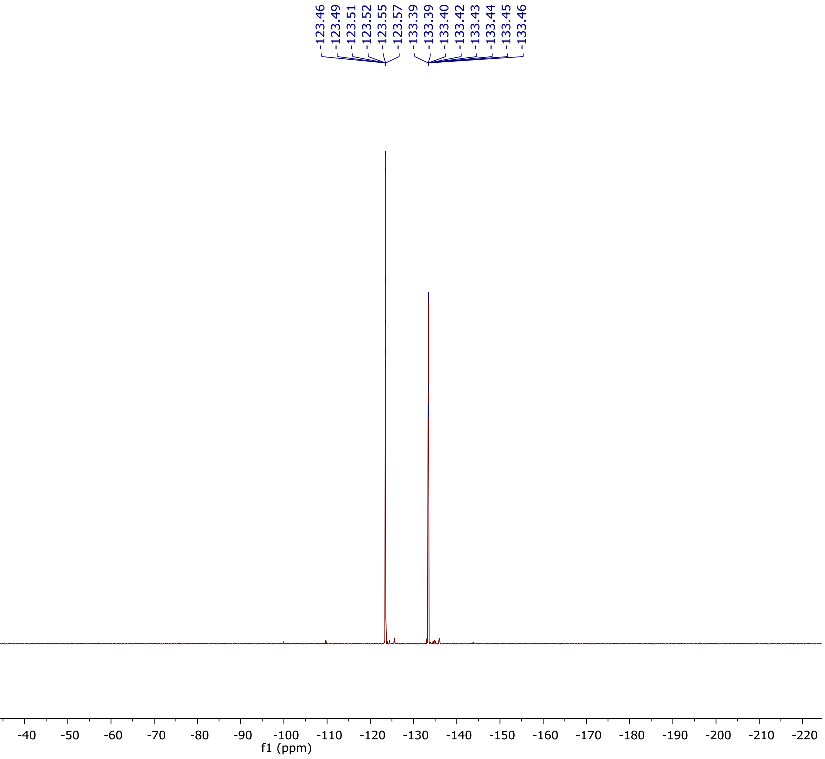

^{1}H NMR Spectrum of **4aa** (400 MHz, $CDCl_3$)

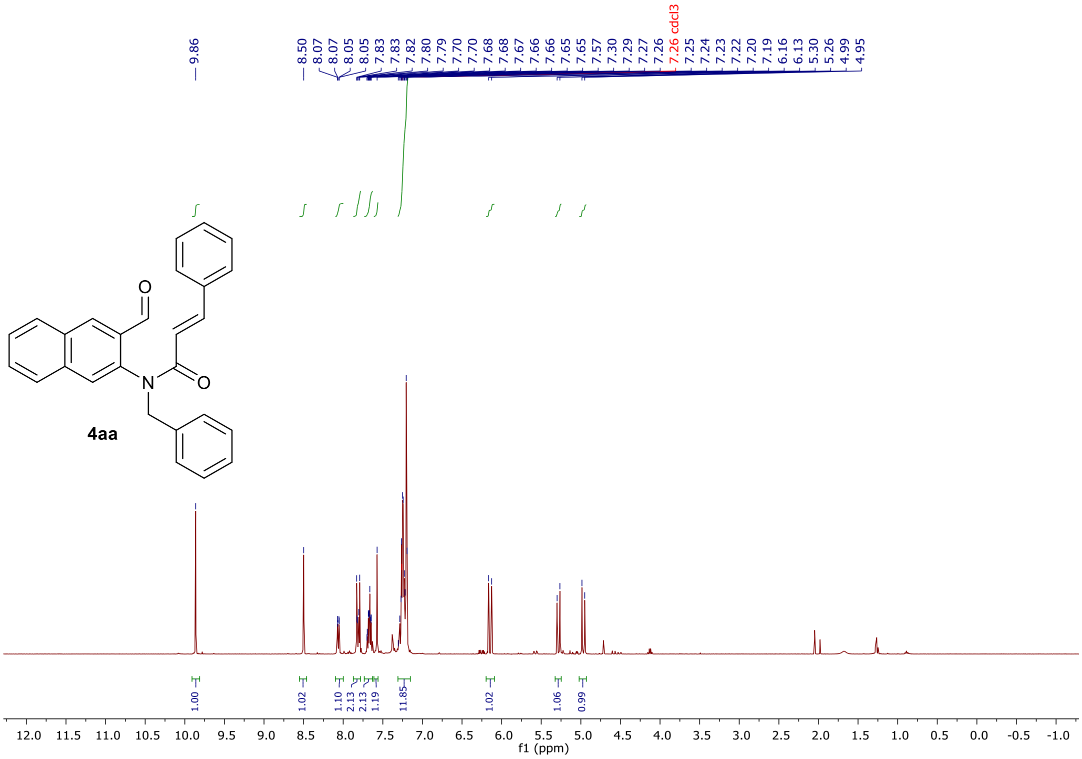

^{13}C NMR Spectrum of **4aa** (101 MHz, $CDCl_3$)

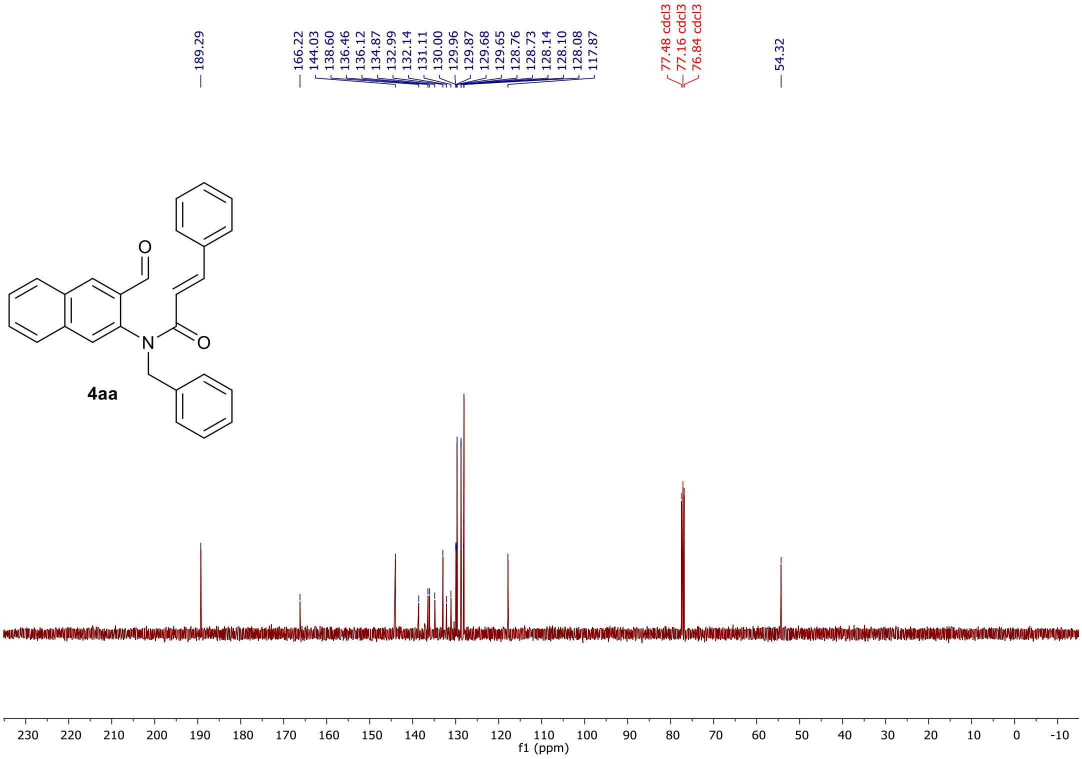

^{1}H NMR Spectrum of **4cc** (400 MHz, $CDCl_3$)

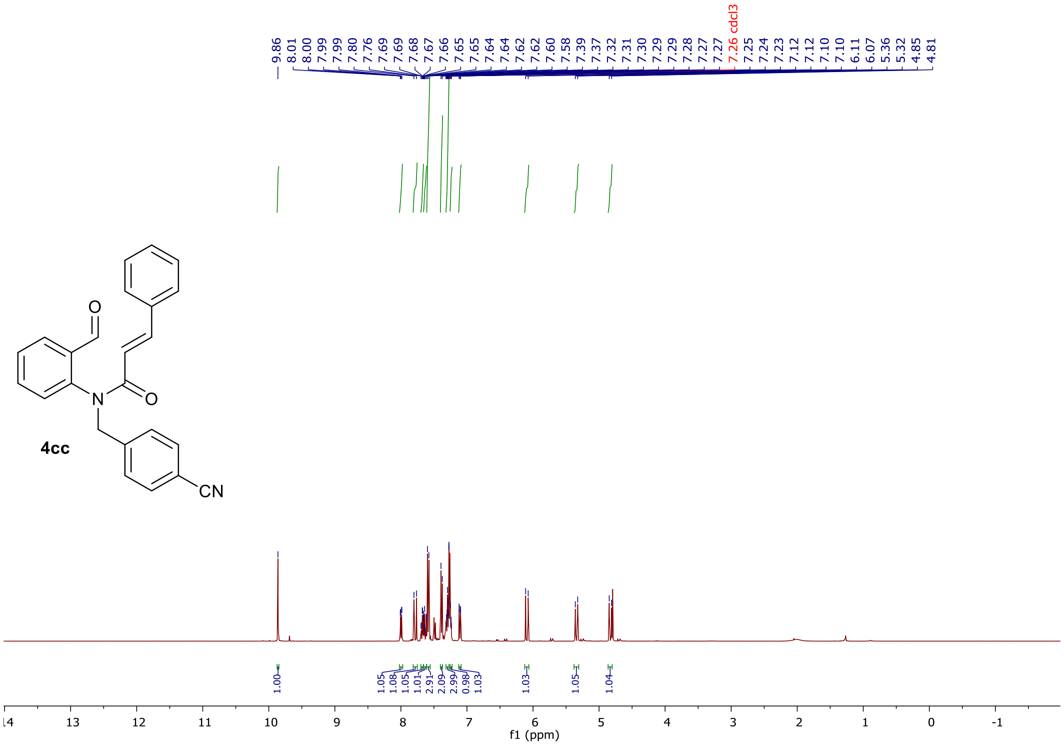

^{13}C NMR Spectrum of **4cc** (176 MHz, $CDCl_3$)

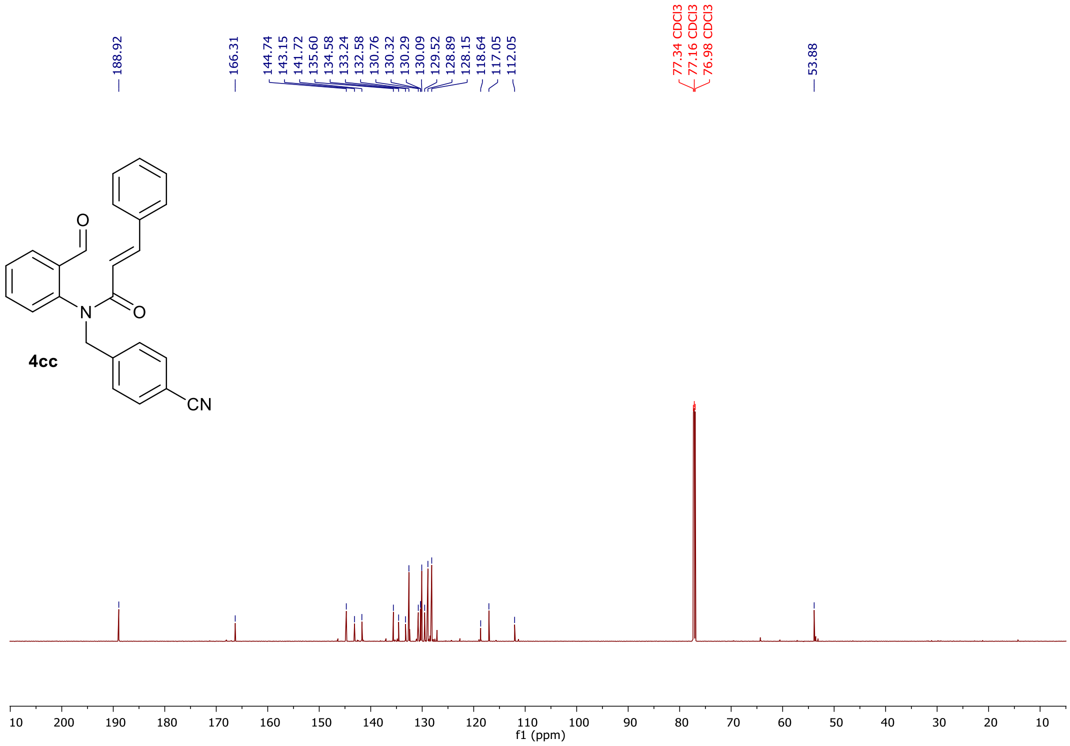

3a HPLC traces: racemate top, enantiomer bottom.

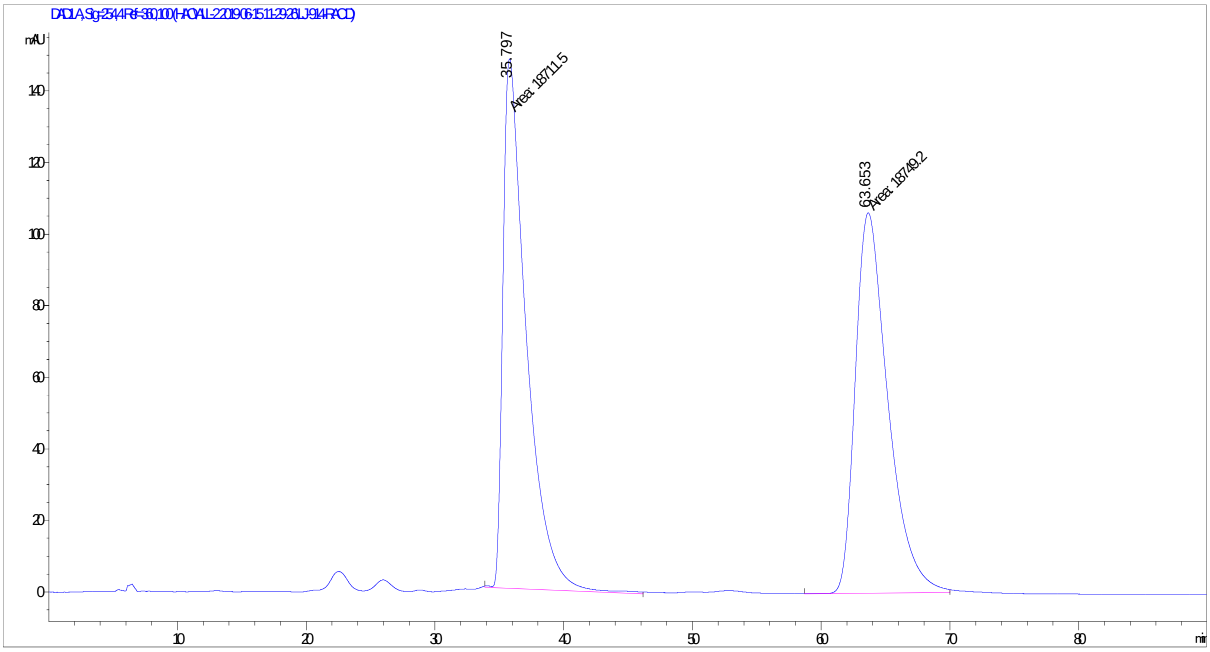

#	Time	Area	Height	Width	Area%	Symmetry
1	35.797	18711.5	148	2.1074	49.950	0.377
2	63.653	18749.2	106.3	2.9387	50.050	0.628

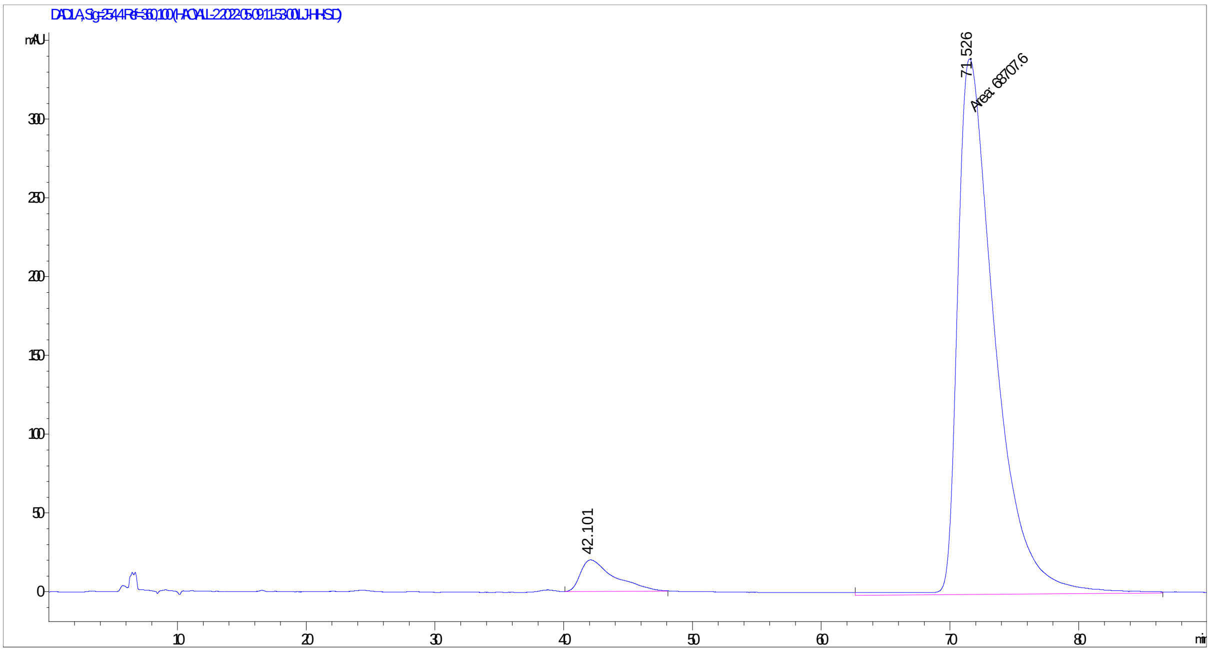

#	Time	Area	Height	Width	Area%	Symmetry
1	42.101	3625.6	20.1	2.1203	5.012	0.426
2	71.526	68707.6	340.1	3.367	94.988	0.458

Ent-**3a**

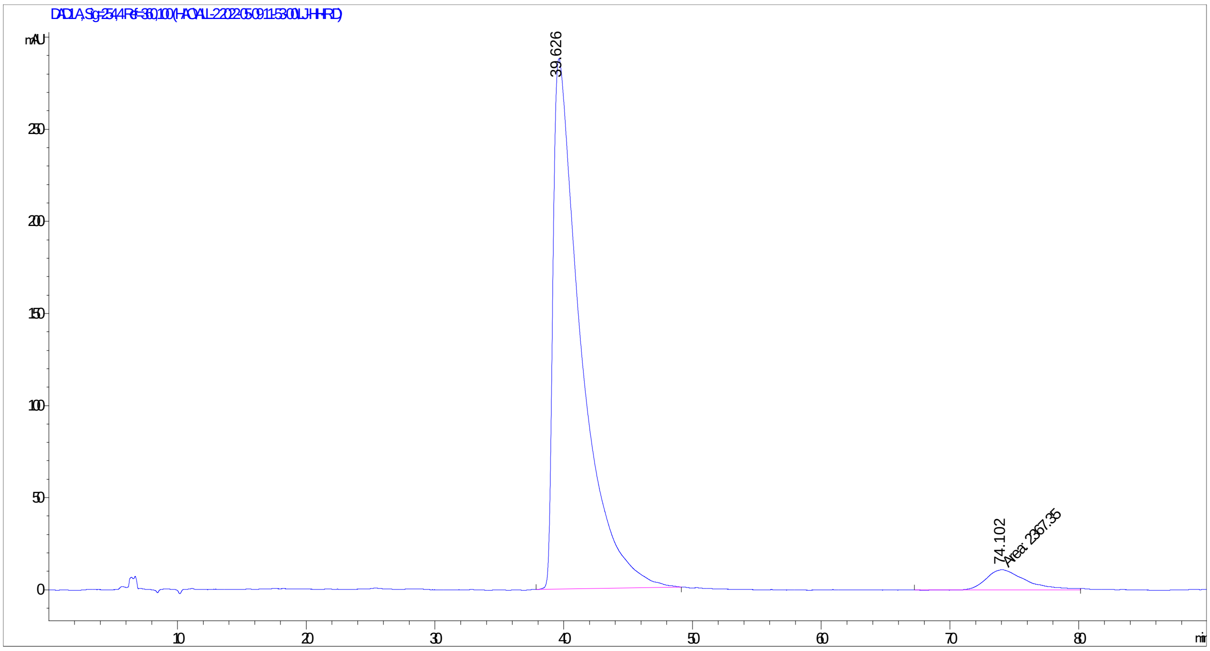

#	Time	Area	Height	Width	Area%	Symmetry
1	39.626	43343.6	288.1	1.9752	94.821	0.245
2	74.102	2367.4	10.9	3.6103	5.179	0.681

3b HPLC traces: racemate top, enantiomer bottom.

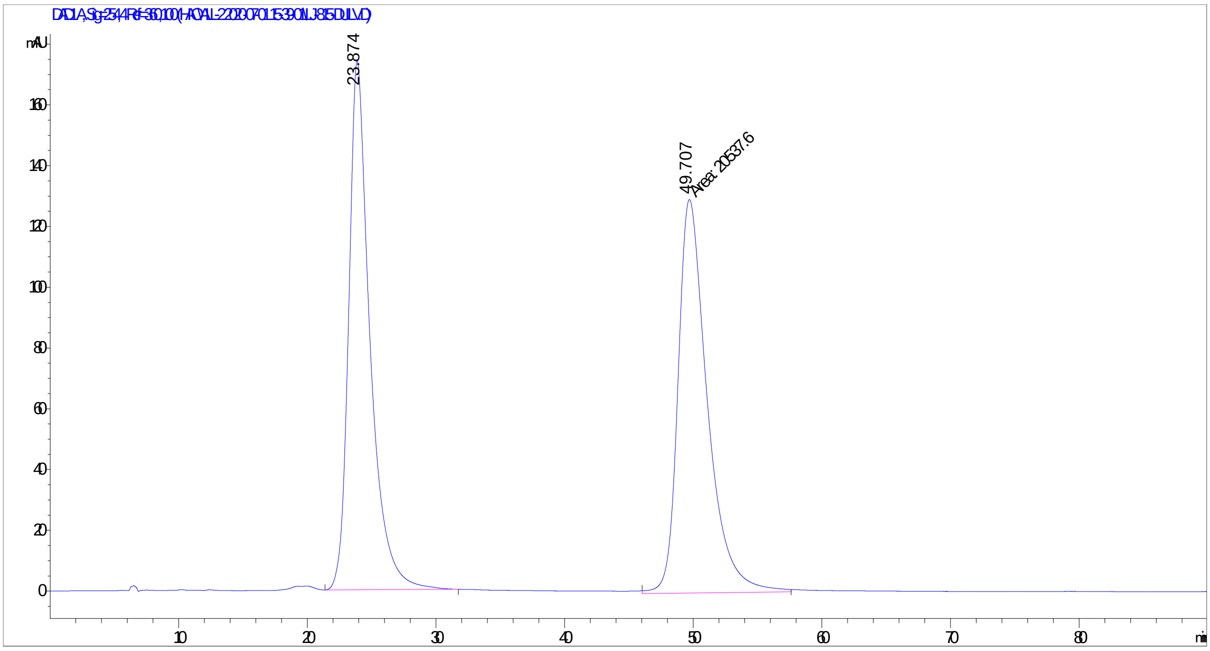

#	Time	Area	Height	Width	Area%	Symmetry
1	23.874	20030.8	174.2	1.5995	49.375	0.612
2	49.707	20537.6	129.5	2.6432	50.625	0.613

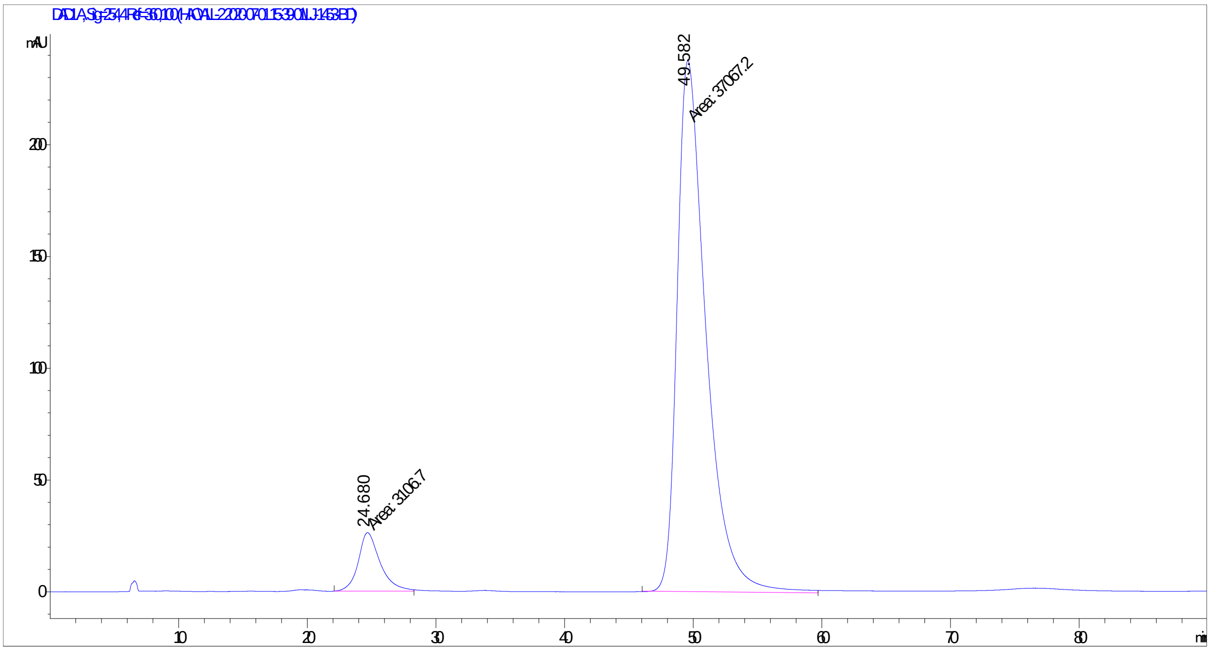

#	Time	Area	Height	Width	Area%	Symmetry
1	24.68	3106.7	26.2	1.9782	7.733	0.712
2	49.582	37067.2	237.6	2.6004	92.267	0.557

3c HPLC traces: racemate top, enantiomer bottom.

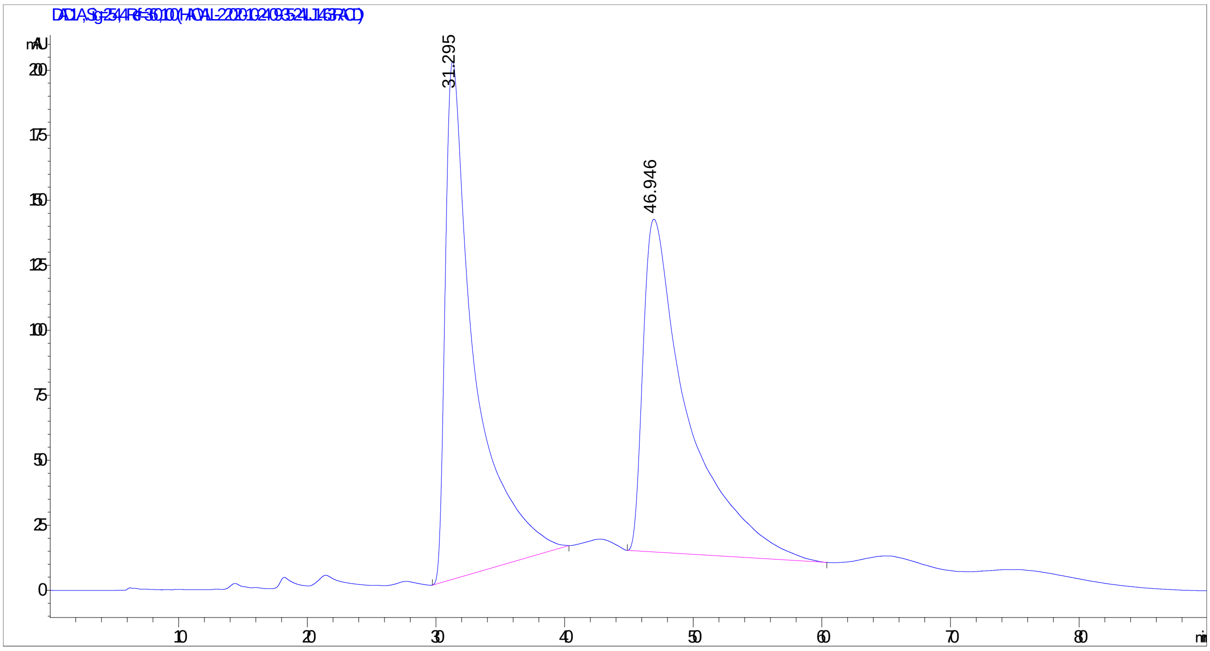

#	Time	Area	Height	Width	Area%	Symmetry
1	31.295	31687.6	199.2	2.2018	50.505	0.296
2	46.946	31054.1	128	3.1898	49.495	0.292

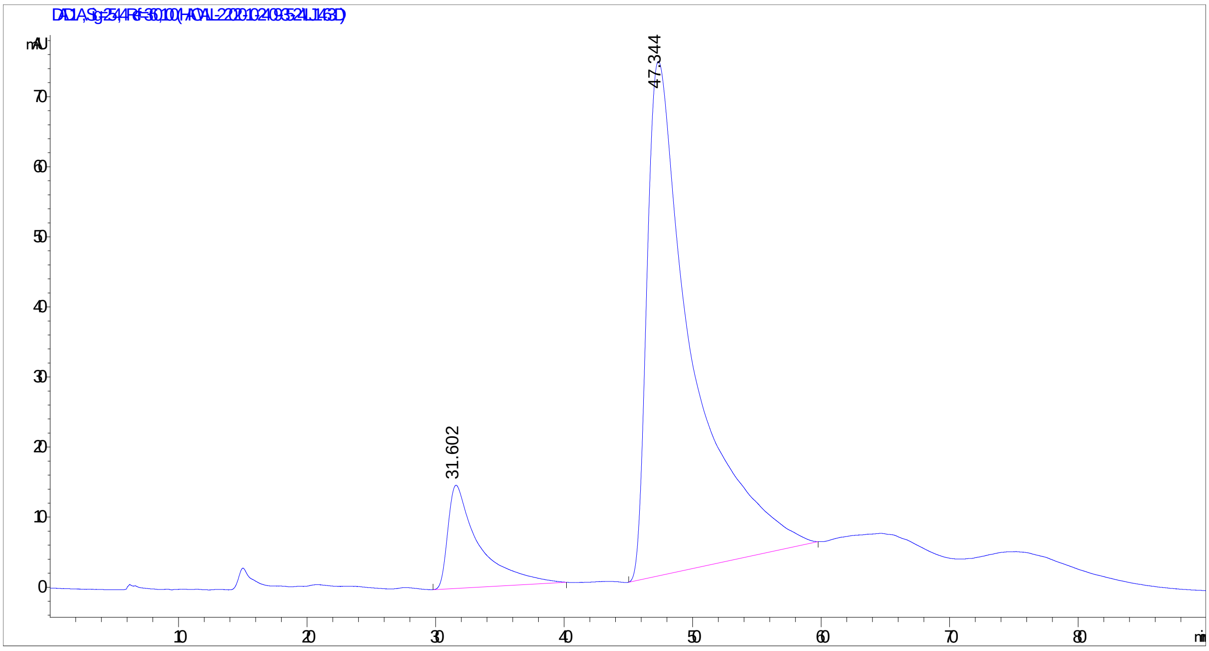

#	Time	Area	Height	Width	Area%	Symmetry
1	31.602	2491.5	14.7	1.9943	12.111	0.342
2	47.344	18080.2	73.4	3.1748	87.889	0.305

3d HPLC traces: racemate top, enantiomer bottom.

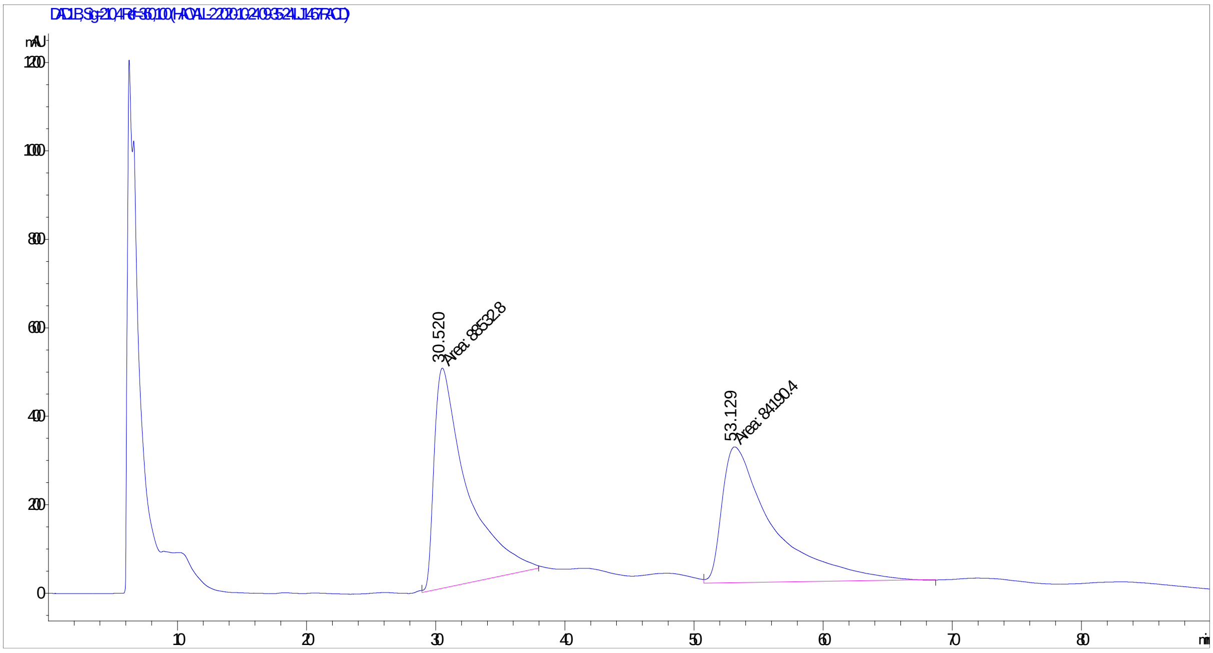

#	Time	Area	Height	Width	Area%	Symmetry
1	30.52	88532.8	498.2	2.962	51.257	0.322
2	53.129	84190.4	307.2	4.5673	48.743	0.315

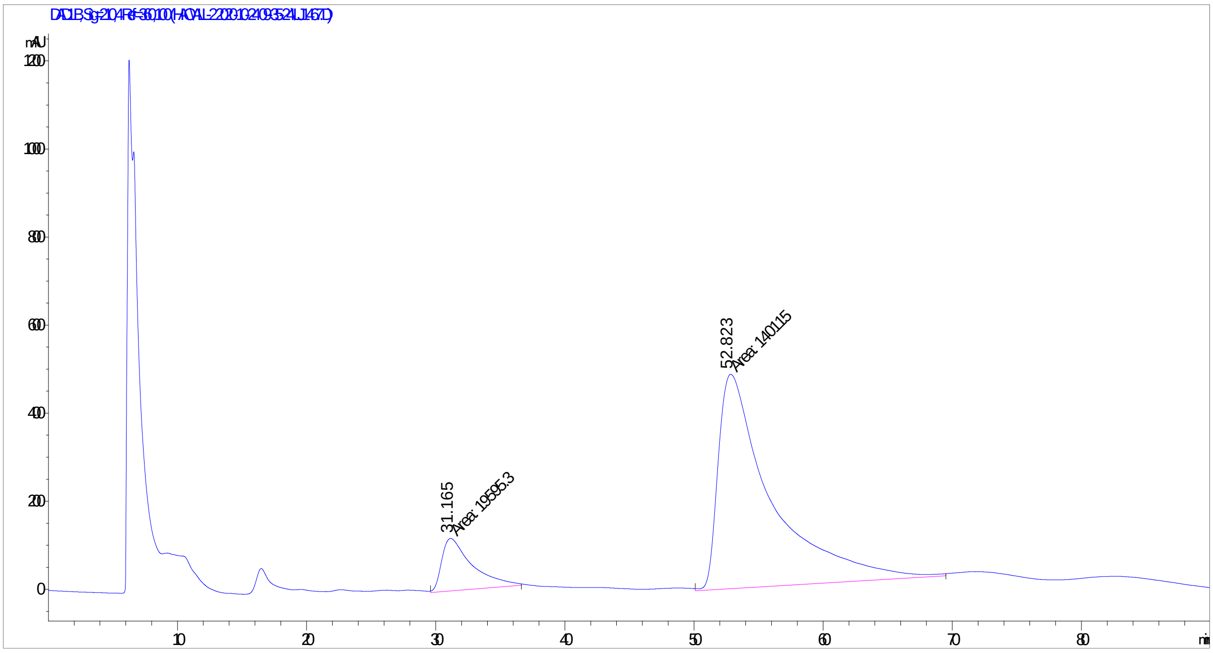

#	Time	Area	Height	Width	Area%	Symmetry
1	31.165	19595.3	119.5	2.7336	12.269	0.413
2	52.823	140115.2	487.1	4.7942	87.731	0.289

^{1}H NMR Spectrum of **3e** (500 MHz, $CDCl_3$)

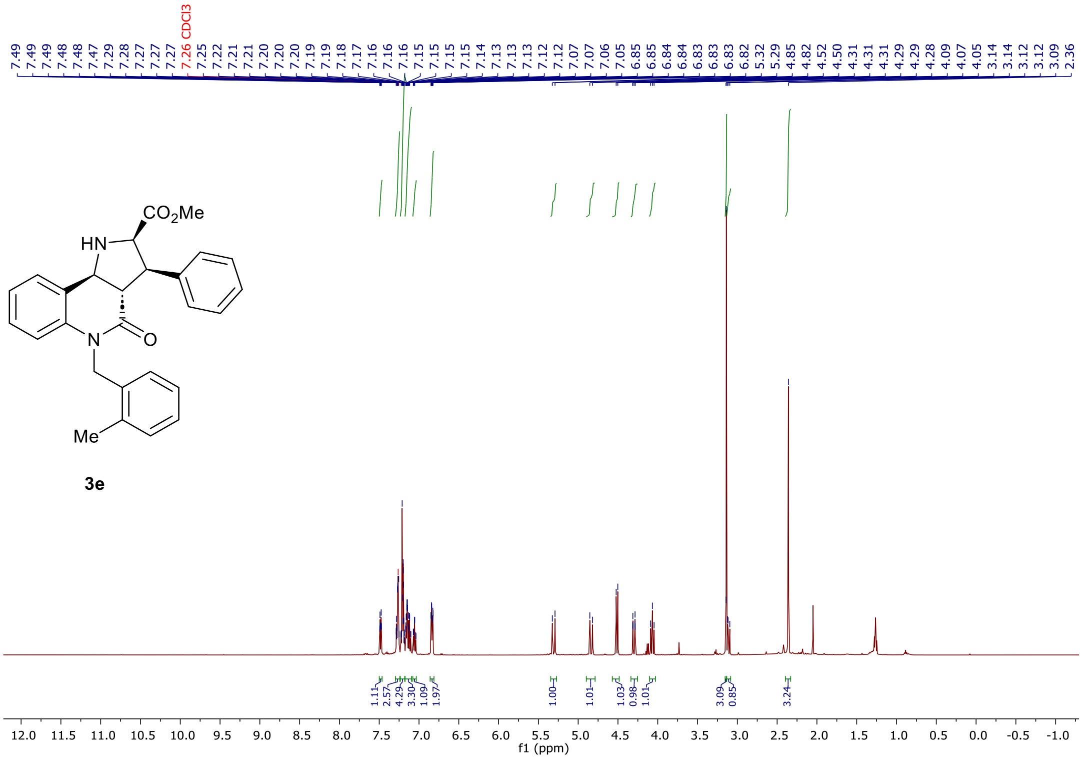

^{13}C NMR Spectrum of **3e** (126 MHz, $CDCl_3$)

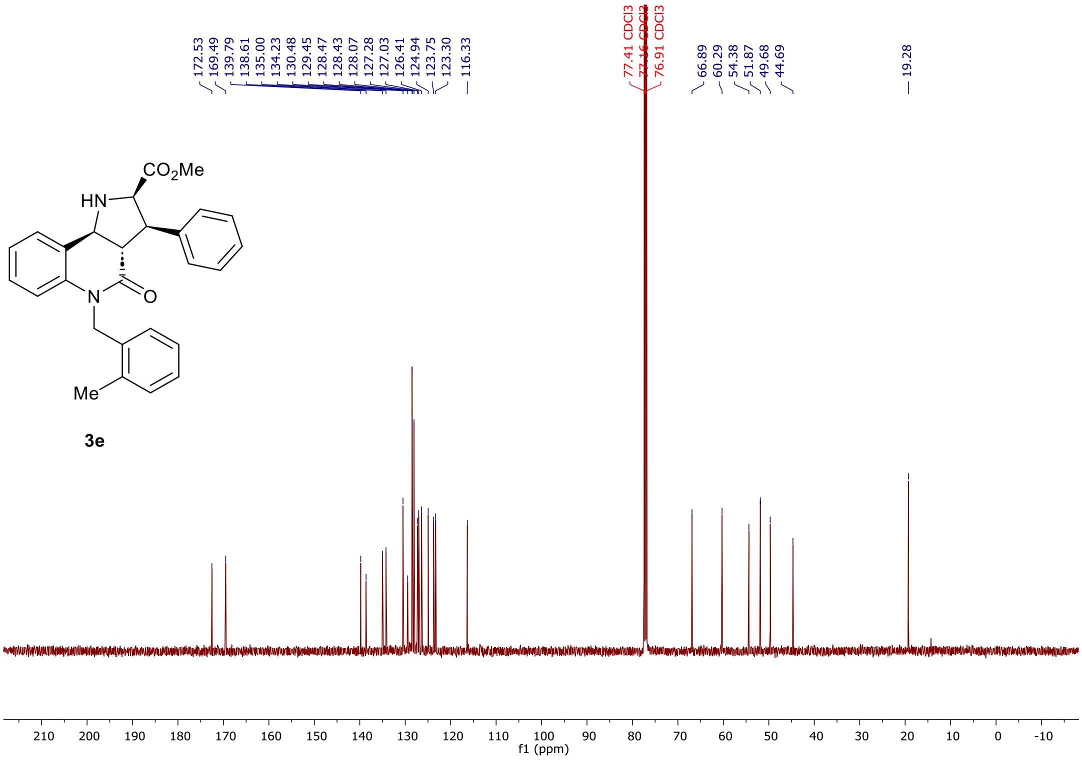

3e HPLC traces: racemate top, enantiomer bottom.

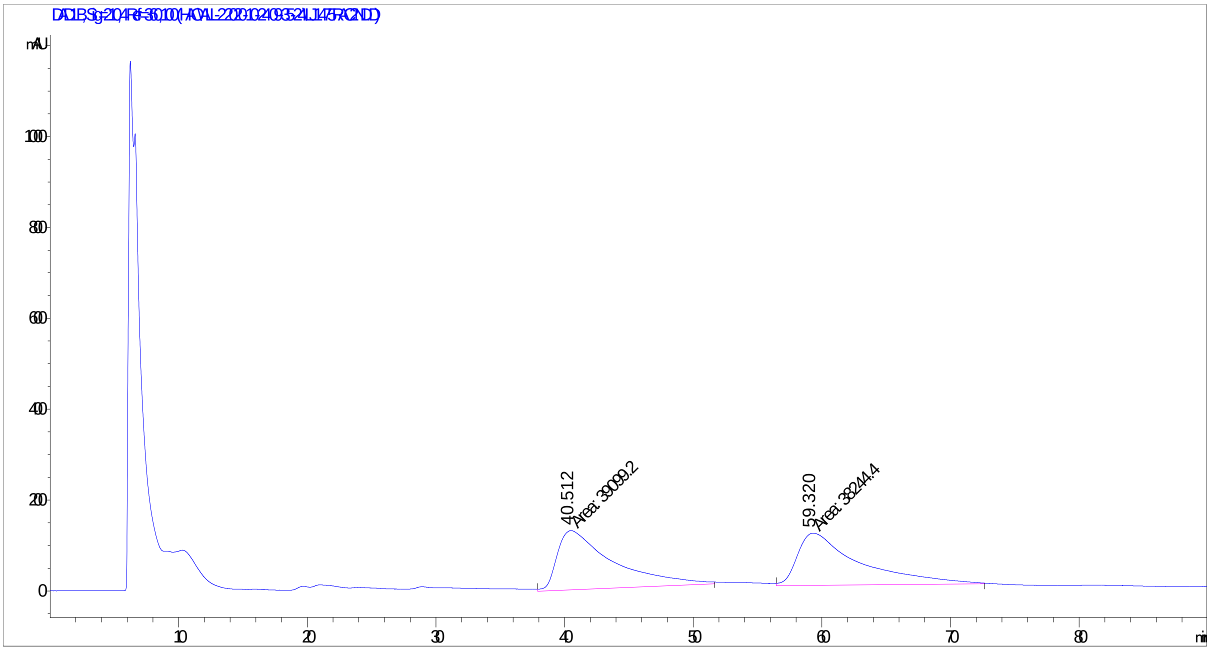

#	Time	Area	Height	Width	Area%	Symmetry
1	40.512	39099.2	130.5	4.9949	50.553	0.327
2	59.32	38244.4	114.8	5.5515	49.447	0.331

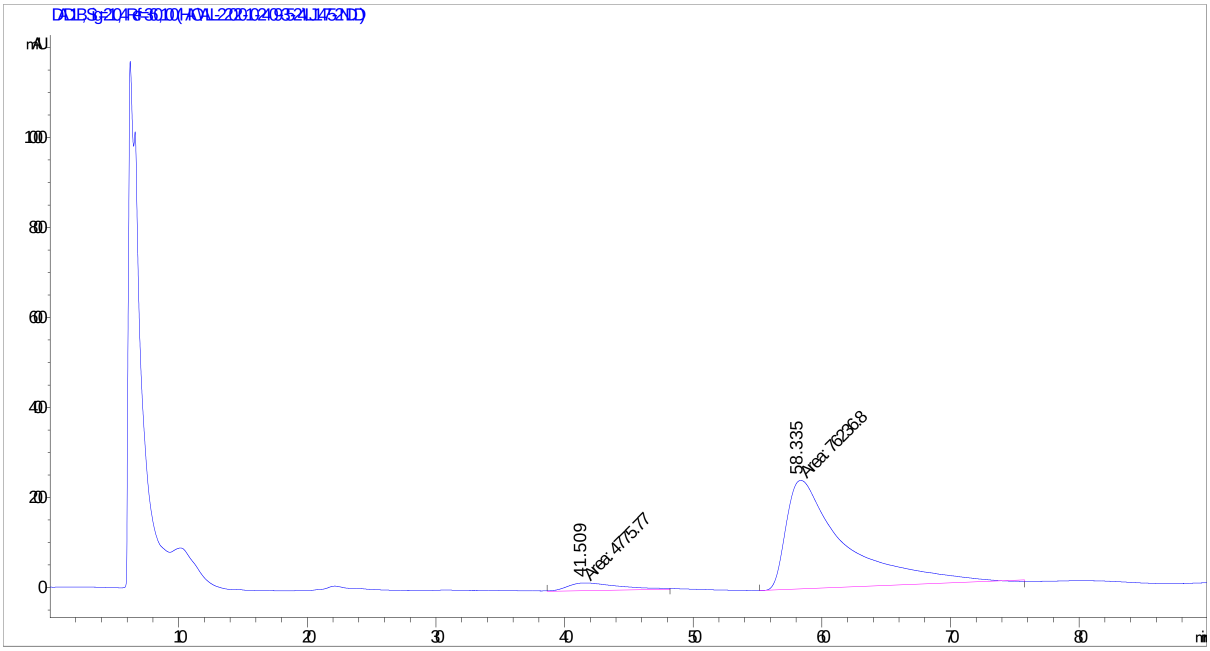

#	Time	Area	Height	Width	Area%	Symmetry
1	41.509	4775.8	17.3	4.5905	5.895	0.474
2	58.335	76236.8	241	5.2729	94.105	0.31

^{1}H NMR Spectrum of **3f** (500 MHz, $CDCl_3$)

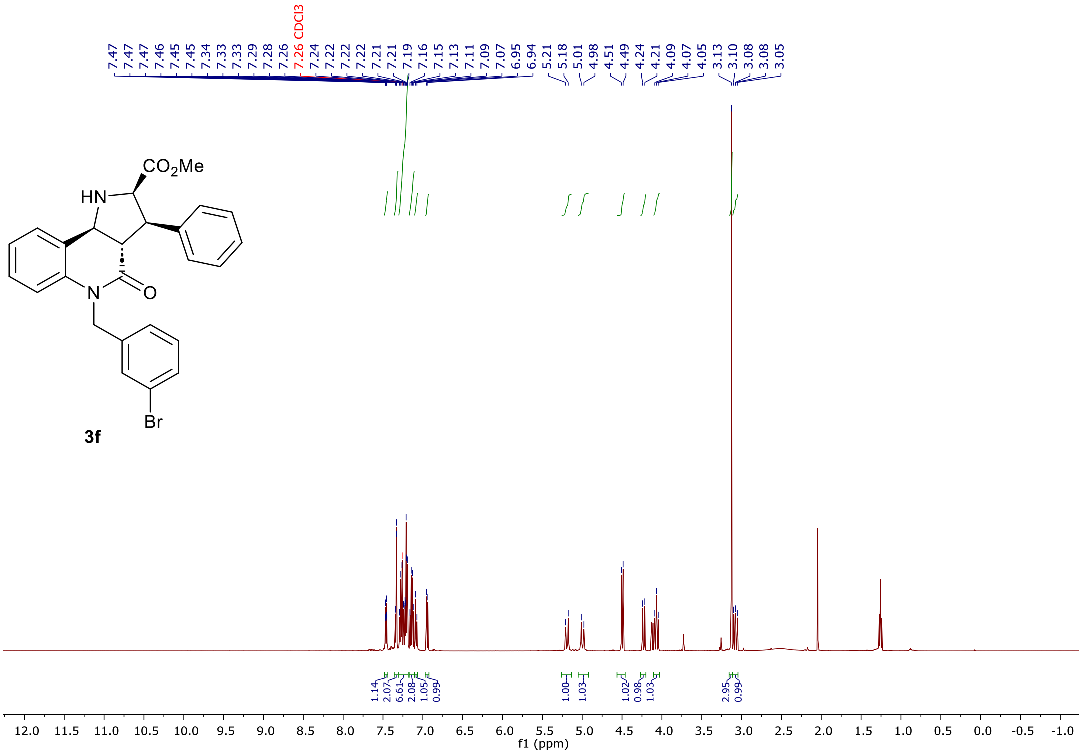

^{13}C NMR Spectrum of **3f** (126 MHz, $CDCl_3$)

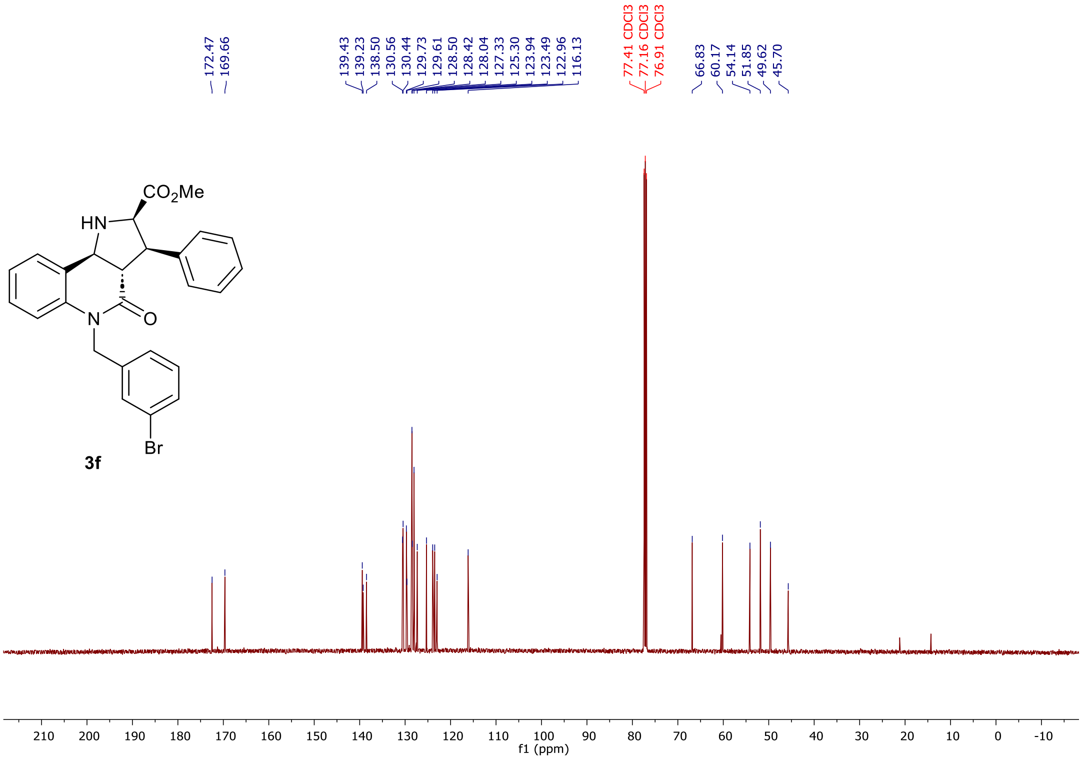

3f HPLC traces: racemate top, enantiomer bottom.

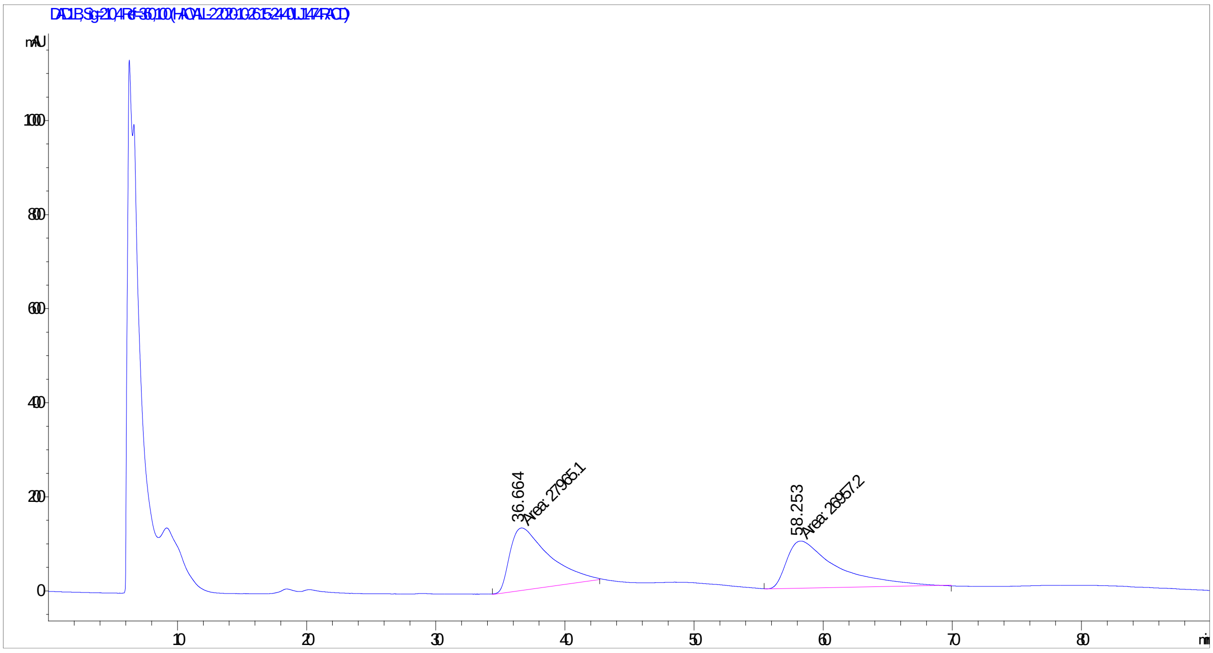

#	Time	Area	Height	Width	Area%	Symmetry
1	36.664	27965.1	132.8	2.4784	50.918	0.428
2	58.253	26957.2	100.1	4.4876	49.082	0.371

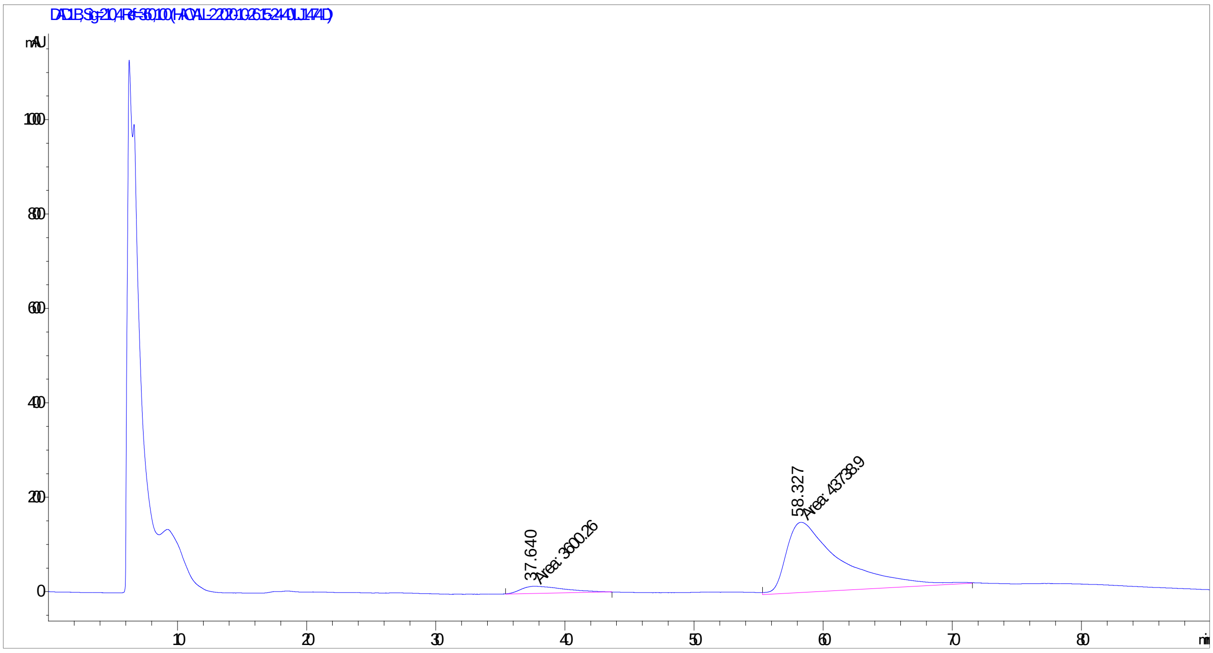

#	Time	Area	Height	Width	Area%	Symmetry
1	37.64	3600.3	15.5	3.8729	7.605	0.453
2	58.327	43738.9	148.7	4.903	92.395	0.373

^{1}H NMR Spectrum of **3g** (500 MHz, $CDCl_3$)

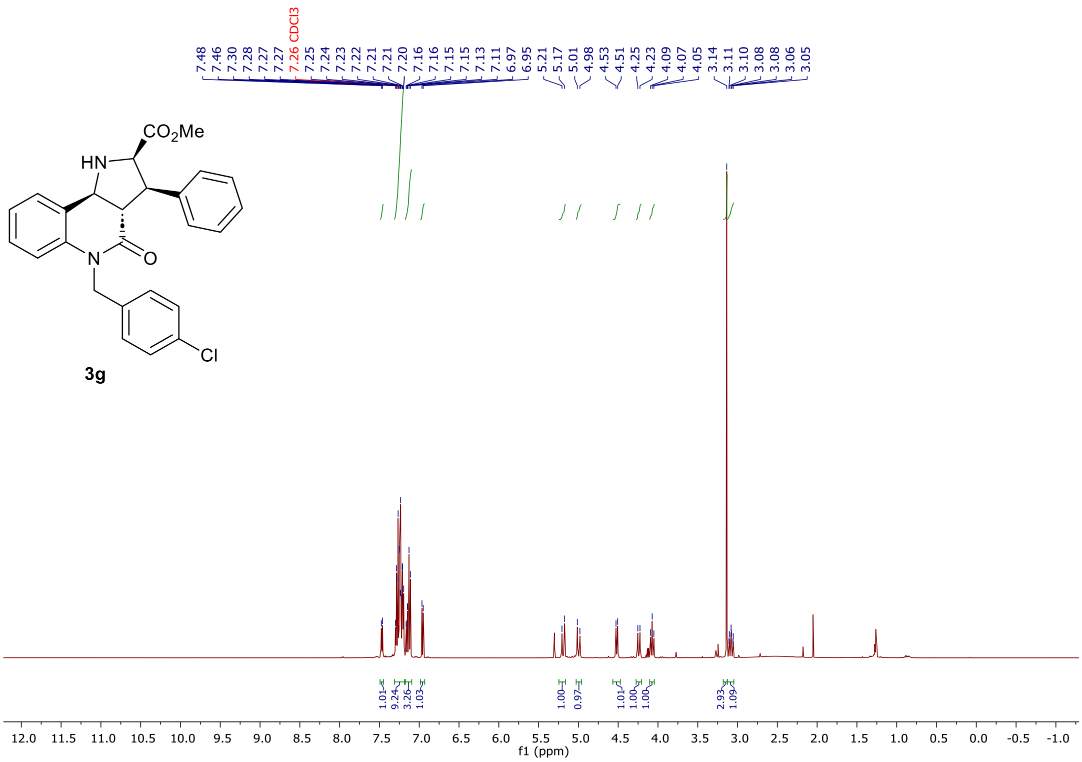

^{13}C NMR Spectrum of **3g** (126 MHz, $CDCl_3$)

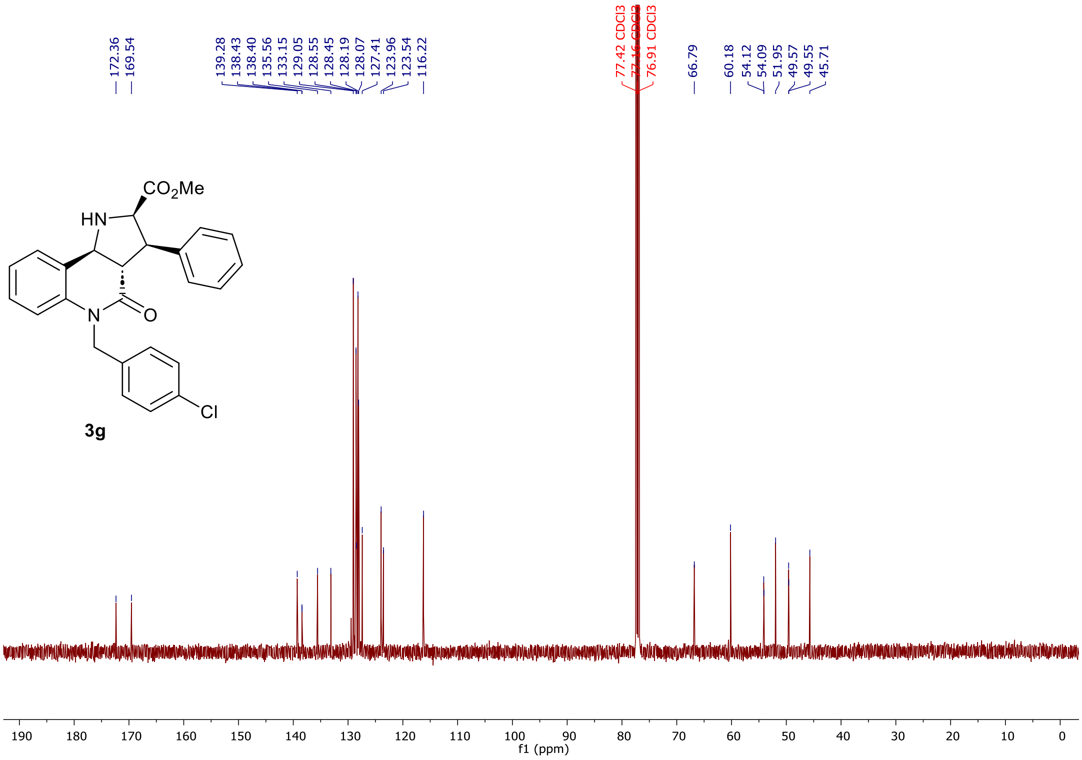

3g HPLC traces: racemate top, enantiomer bottom.

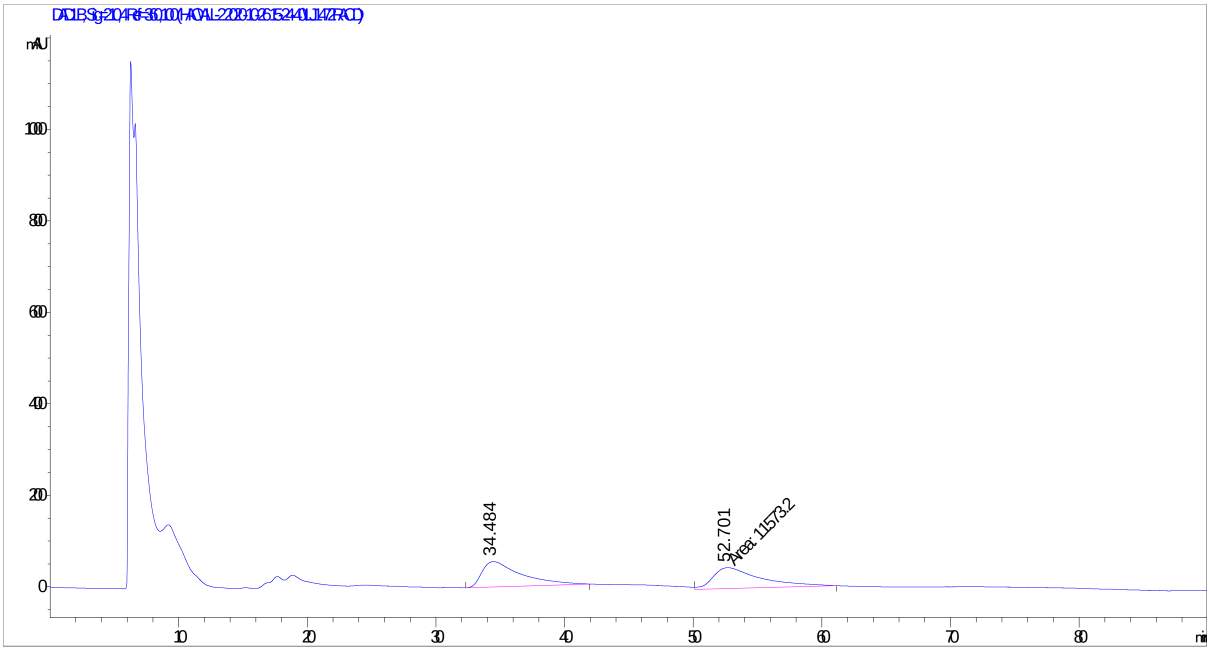

#	Time	Area	Height	Width	Area%	Symmetry
1	34.484	11743.4	55.5	2.4887	50.365	0.411
2	52.701	11573.2	45.4	4.2451	49.635	0.463

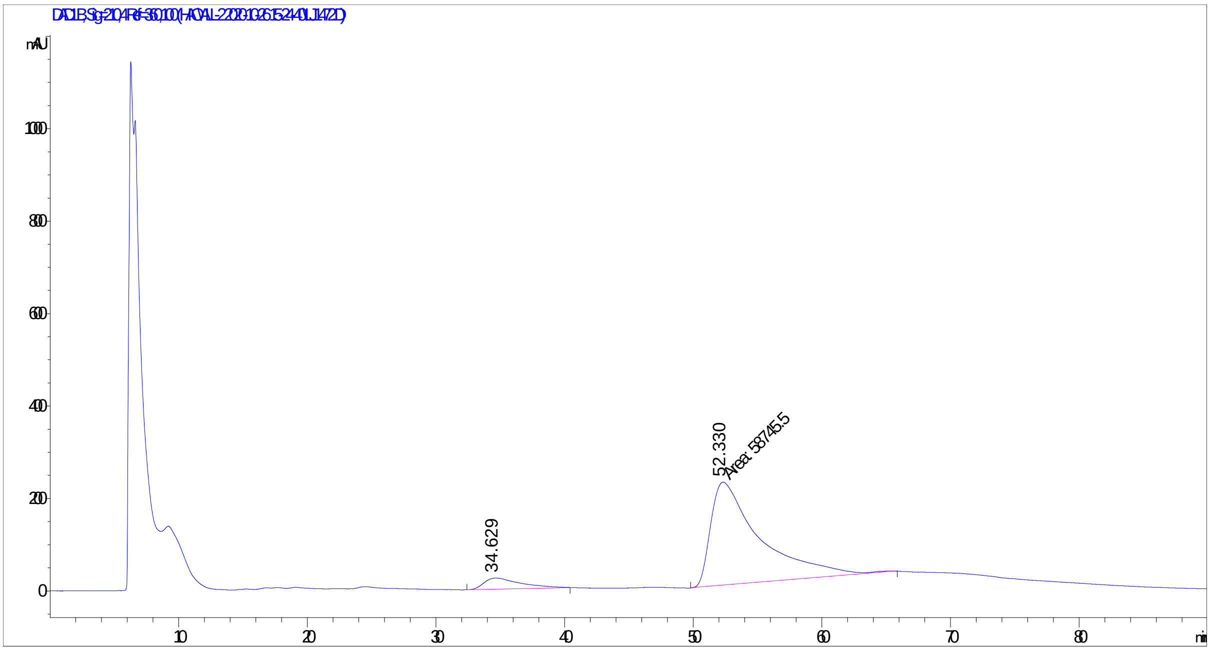

#	Time	Area	Height	Width	Area%	Symmetry
1	34.629	4717.6	24.3	2.2773	7.434	0.458
2	52.33	58745.5	222.4	4.4019	92.566	0.331

^{1}H NMR Spectrum of 3h (700 MHz, $CDCl_3$)

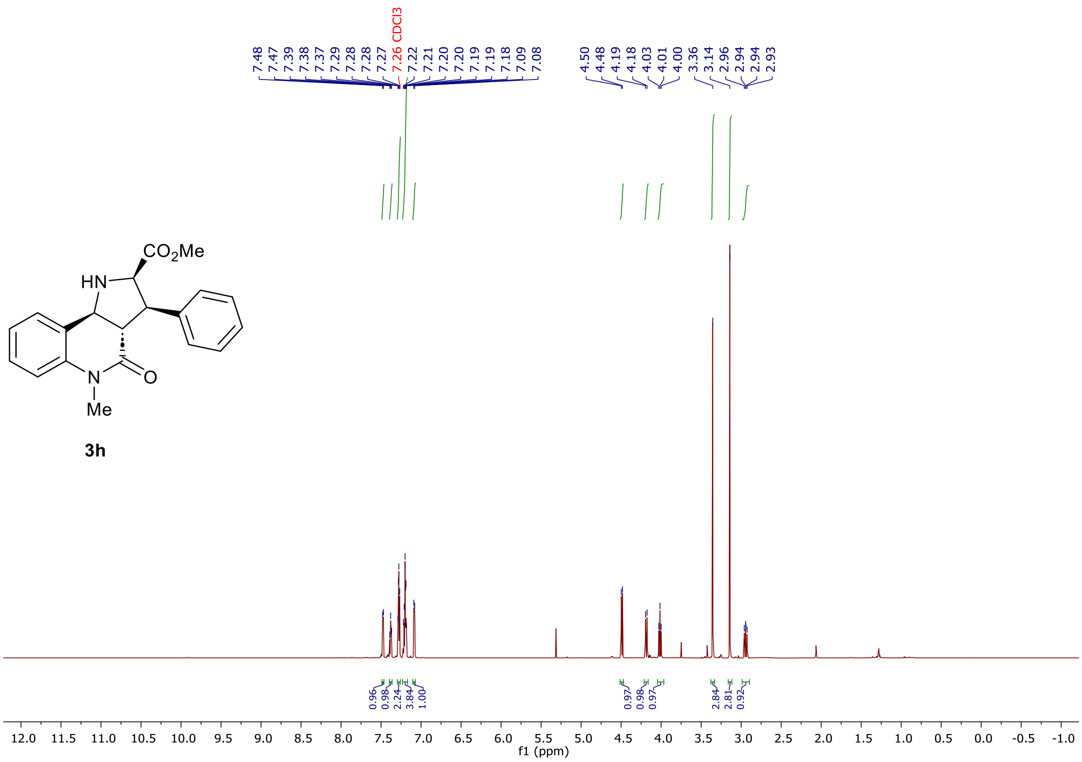

^{13}C NMR Spectrum of 3h (176 MHz, $CDCl_3$)

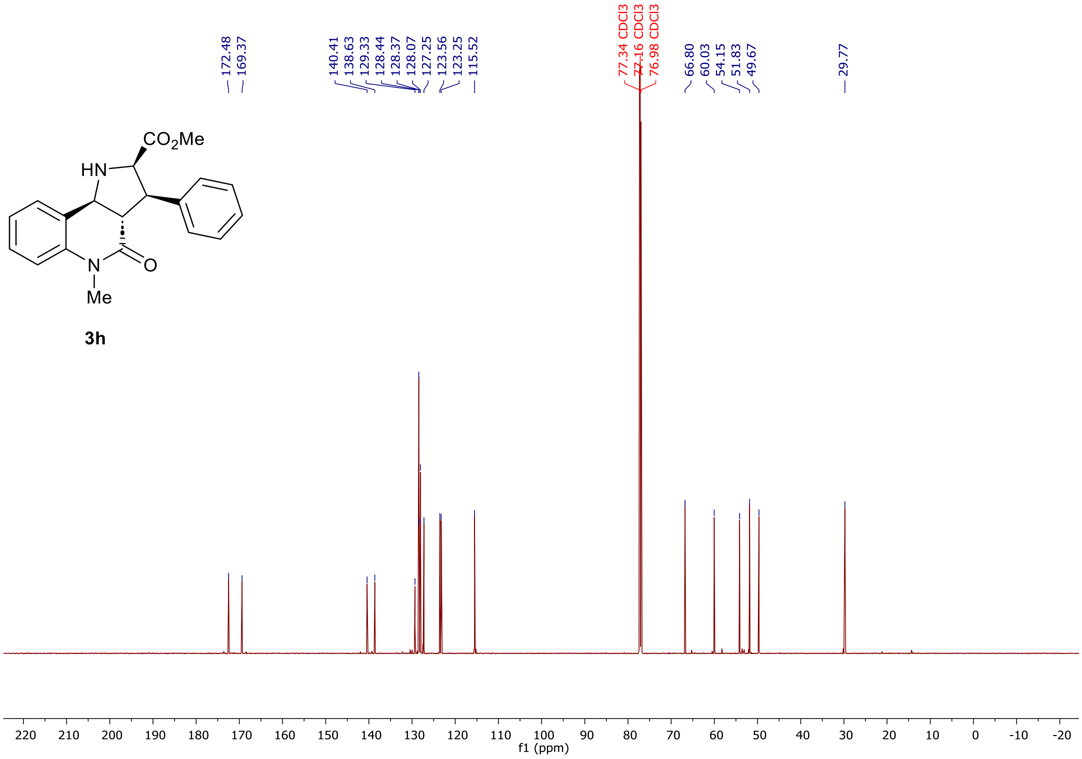

3h HPLC traces: racemate top, enantiomer bottom.

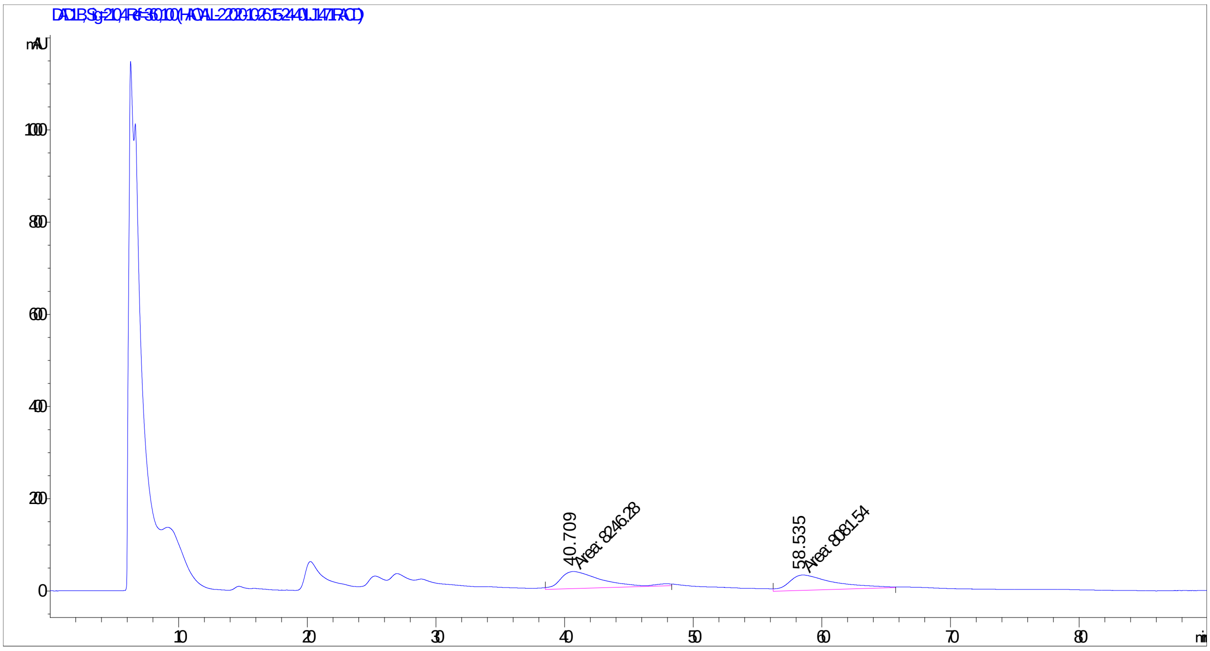

#	Time	Area	Height	Width	Area%	Symmetry
1	40.709	8246.3	36.9	3.7265	50.504	0.466
2	58.535	8081.5	33.3	4.0427	49.496	0.452

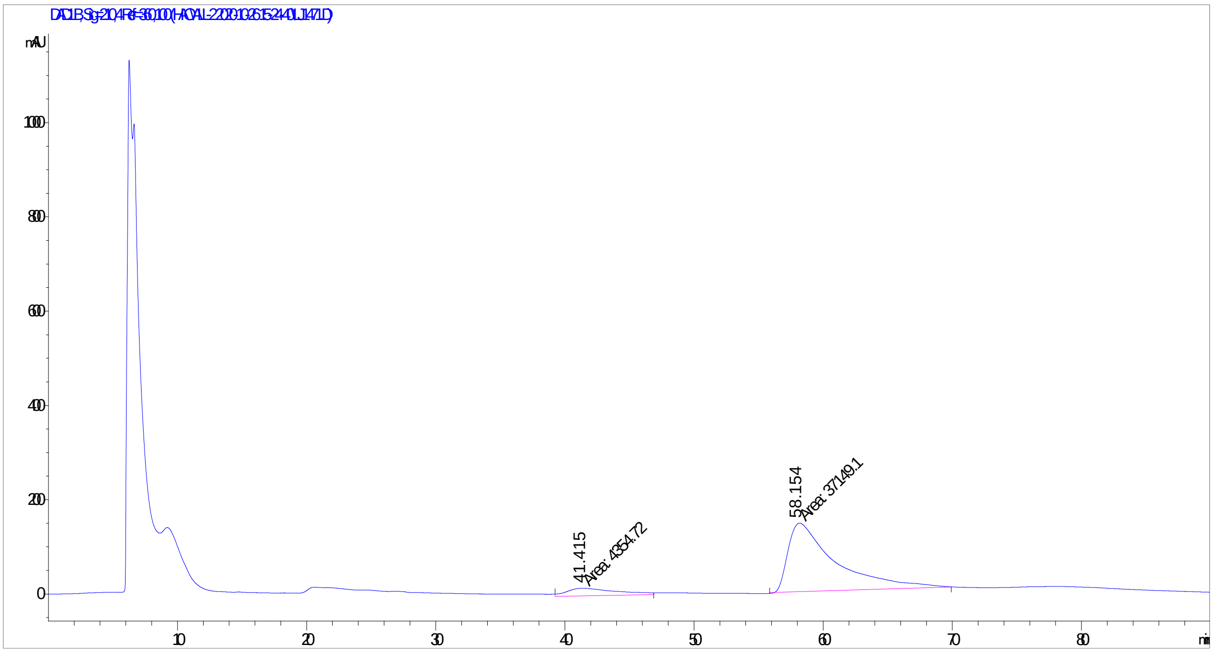

#	Time	Area	Height	Width	Area%	Symmetry
1	41.415	4354.7	16.3	4.4517	10.492	0.5
2	58.154	37149.1	145.7	4.2503	89.508	0.304

^{1}H NMR Spectrum of **3i** (500 MHz, $CDCl_3$)

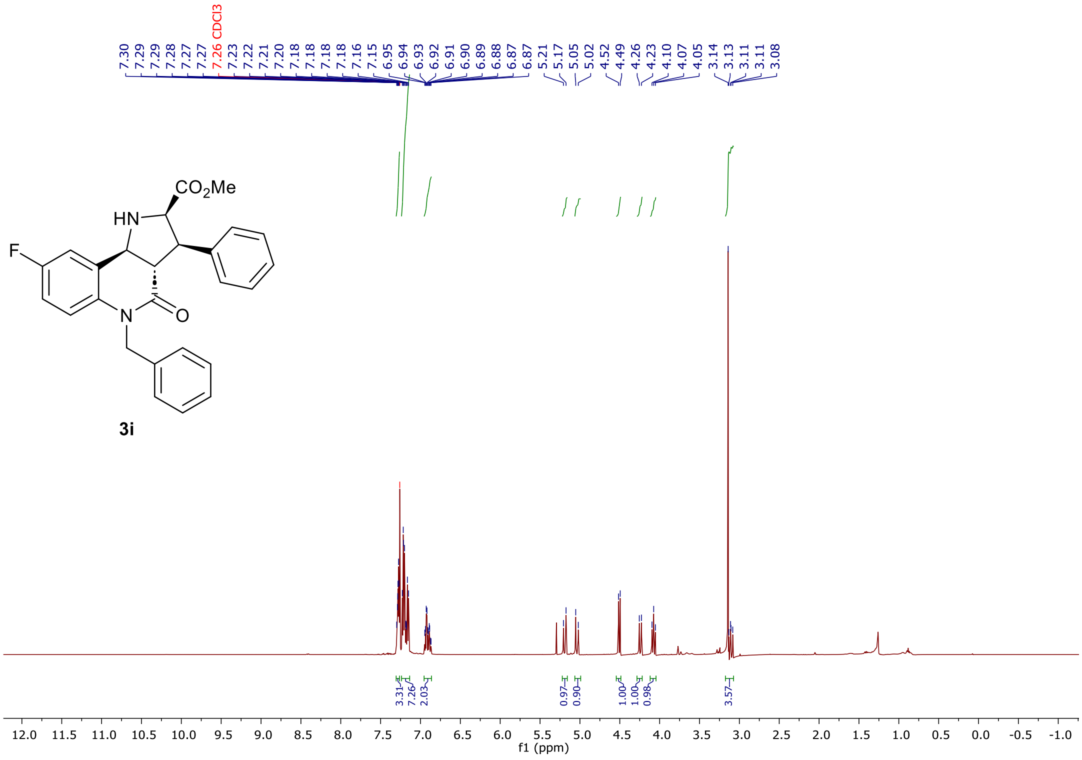

^{13}C NMR Spectrum of **3i** (126 MHz, $CDCl_3$)

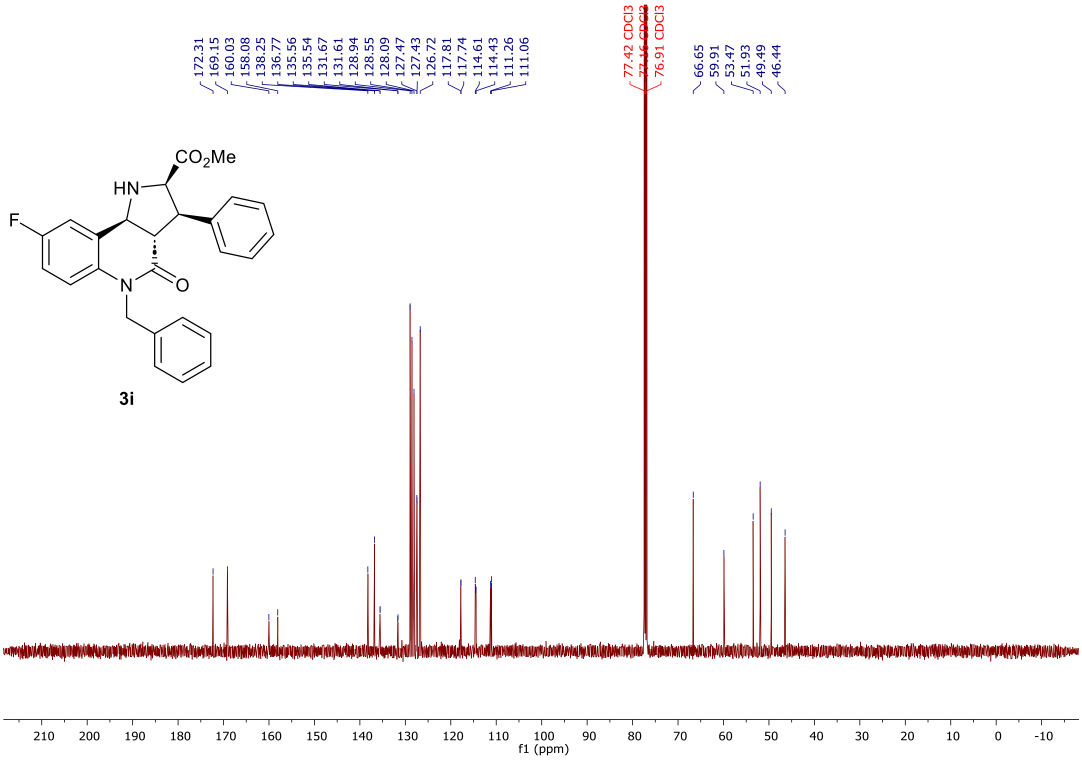

^{19}F NMR Spectrum of **3i** (470 MHz, $CDCl_3$)

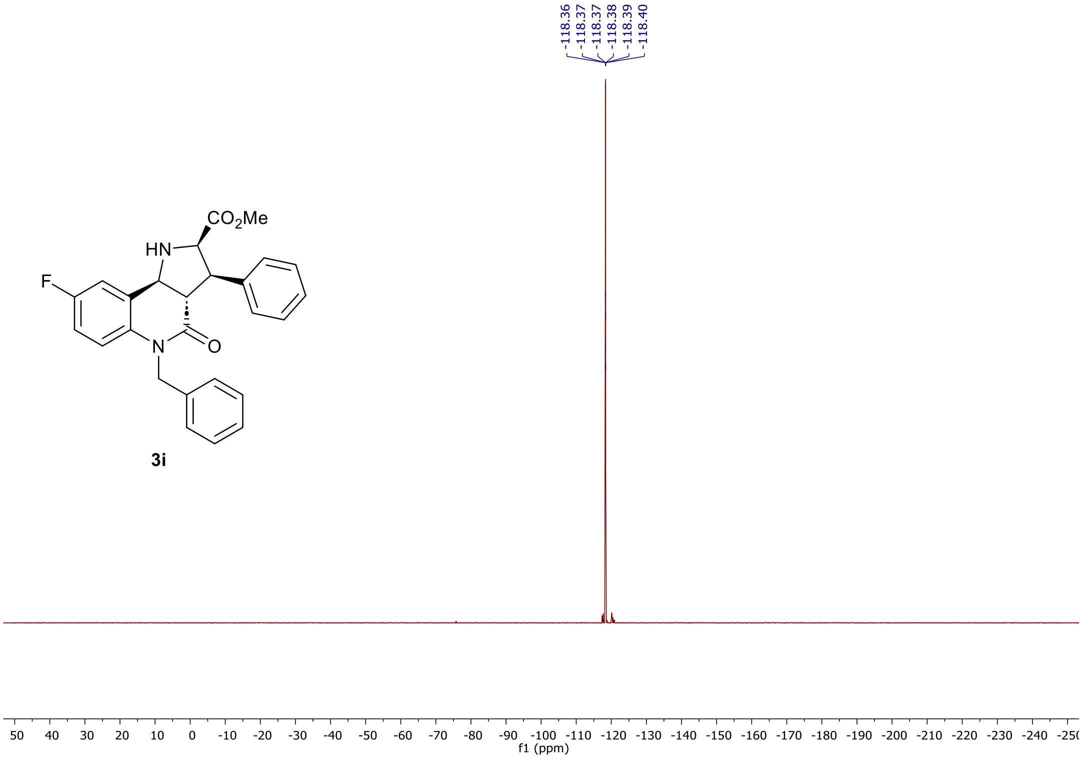

3i HPLC traces: racemate top, enantiomer bottom.

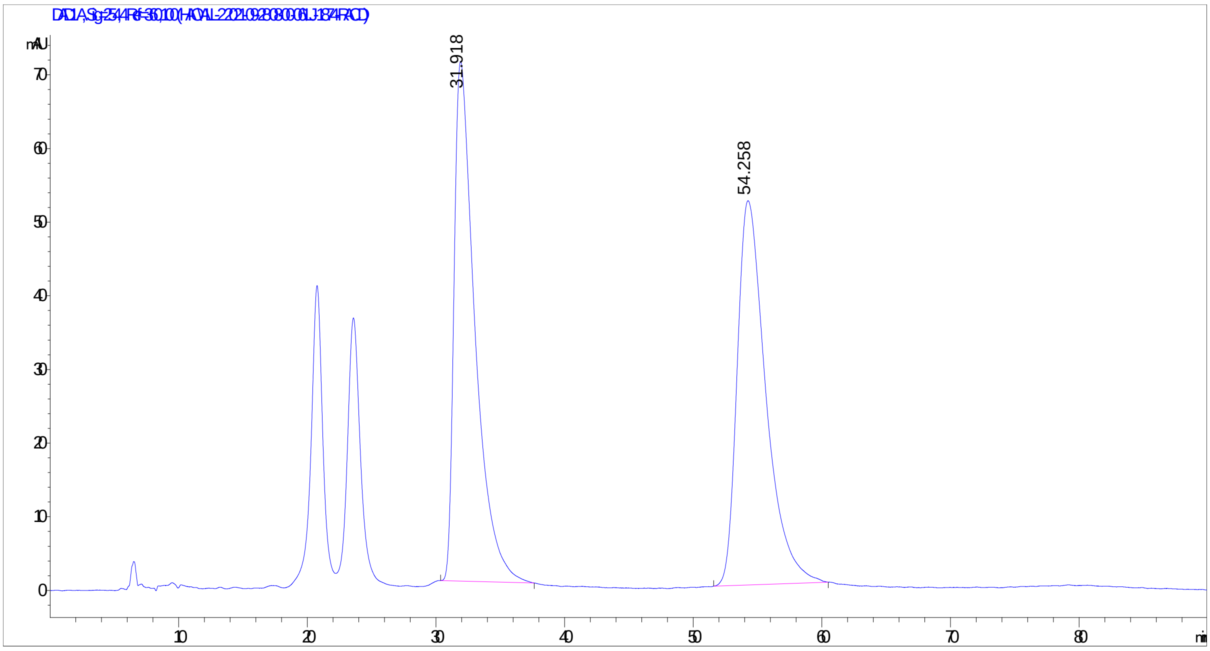

#	Time	Area	Height	Width	Area%	Symmetry
1	31.918	7966.4	70.6	1.5529	49.598	0.42
2	54.258	8095.4	52.2	1.9957	50.402	0.552

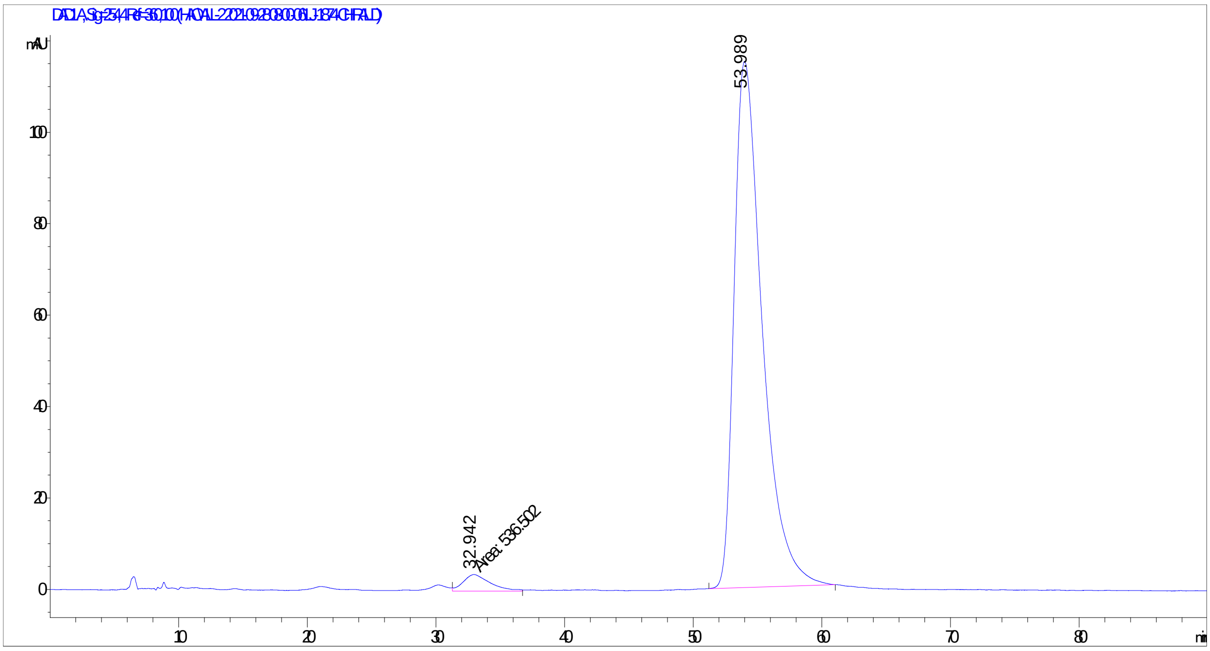

#	Time	Area	Height	Width	Area%	Symmetry
1	32.942	536.5	3.6	2.4633	2.978	0.625
2	53.989	17481.6	115.1	2.144	97.022	0.546

^{1}H NMR Spectrum of **3j** (700 MHz, $CDCl_3$)

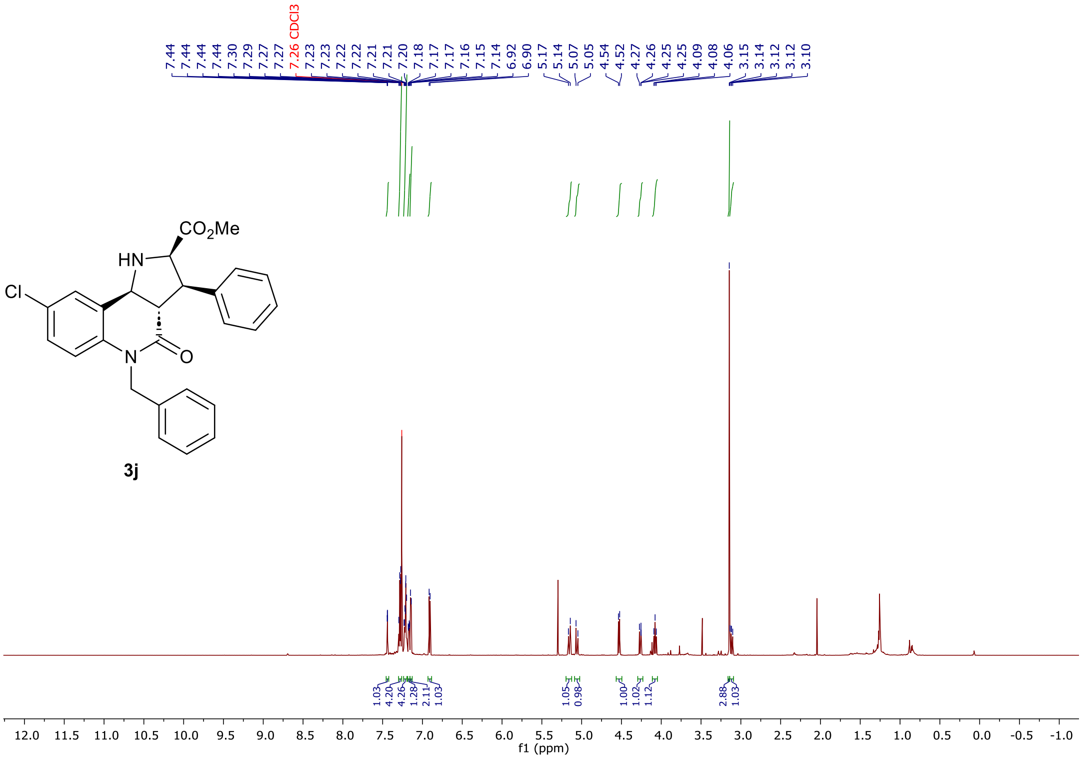

^{13}C NMR Spectrum of **3j** (176 MHz, $CDCl_3$)

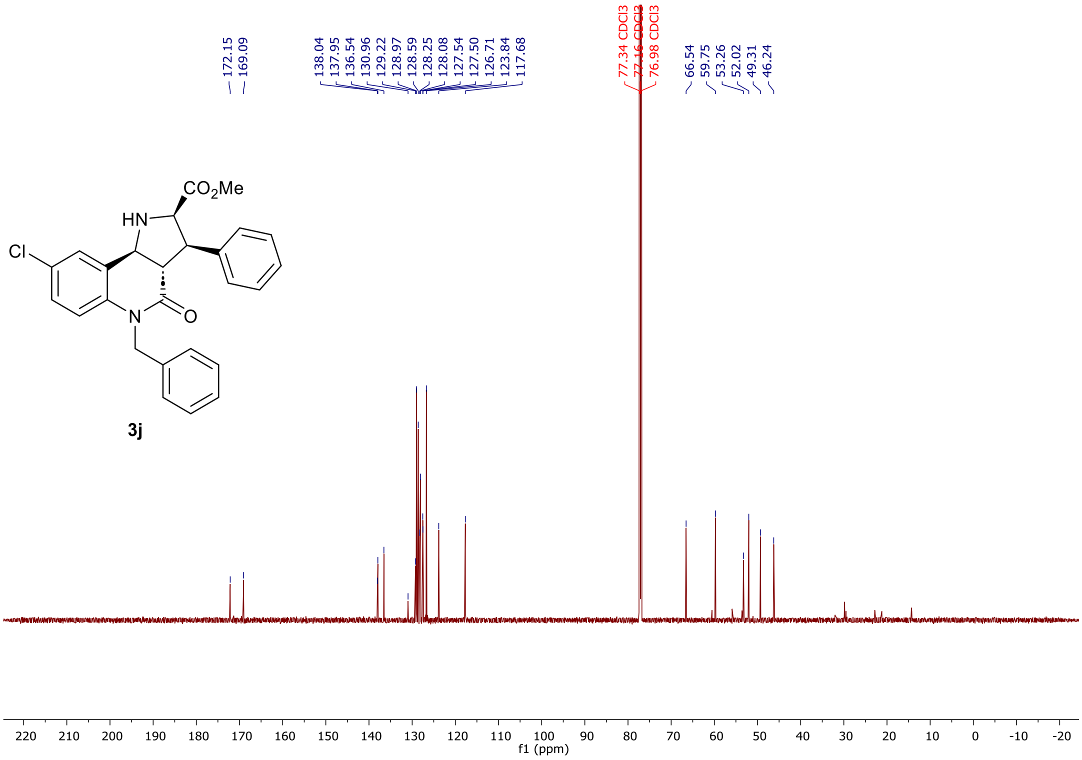

3j HPLC traces: racemate top, enantiomer bottom.

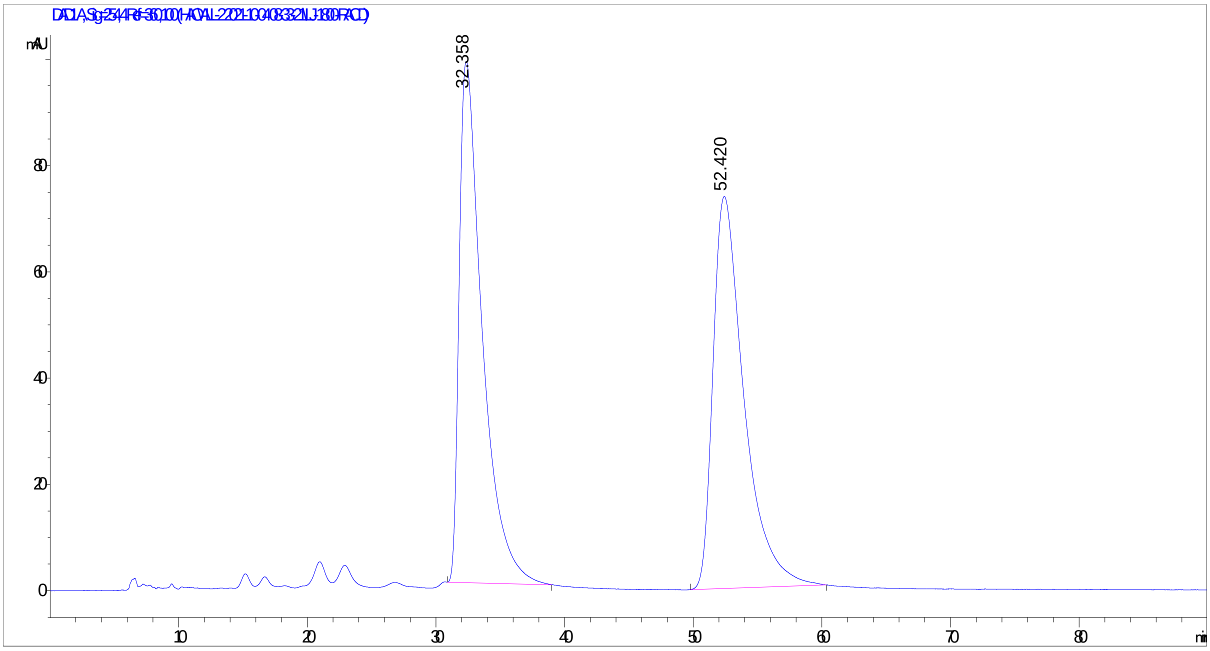

#	Time	Area	Height	Width	Area%	Symmetry
1	32.358	12233.2	98.1	1.7288	49.736	0.406
2	52.42	12362.9	73.8	2.0695	50.264	0.553

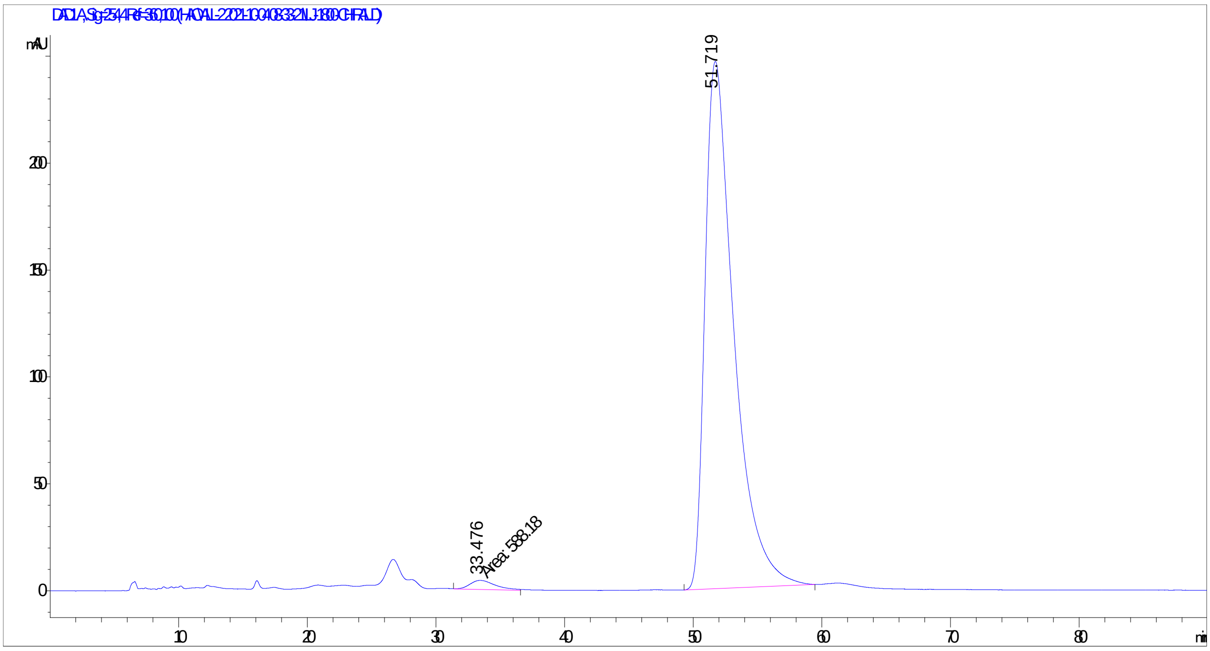

#	Time	Area	Height	Width	Area%	Symmetry
1	33.476	588.2	4.3	2.2965	1.507	0.704
2	51.719	38446.9	246.6	2.197	98.493	0.509

^{1}H NMR Spectrum of **3k** (500 MHz, $CDCl_3$)

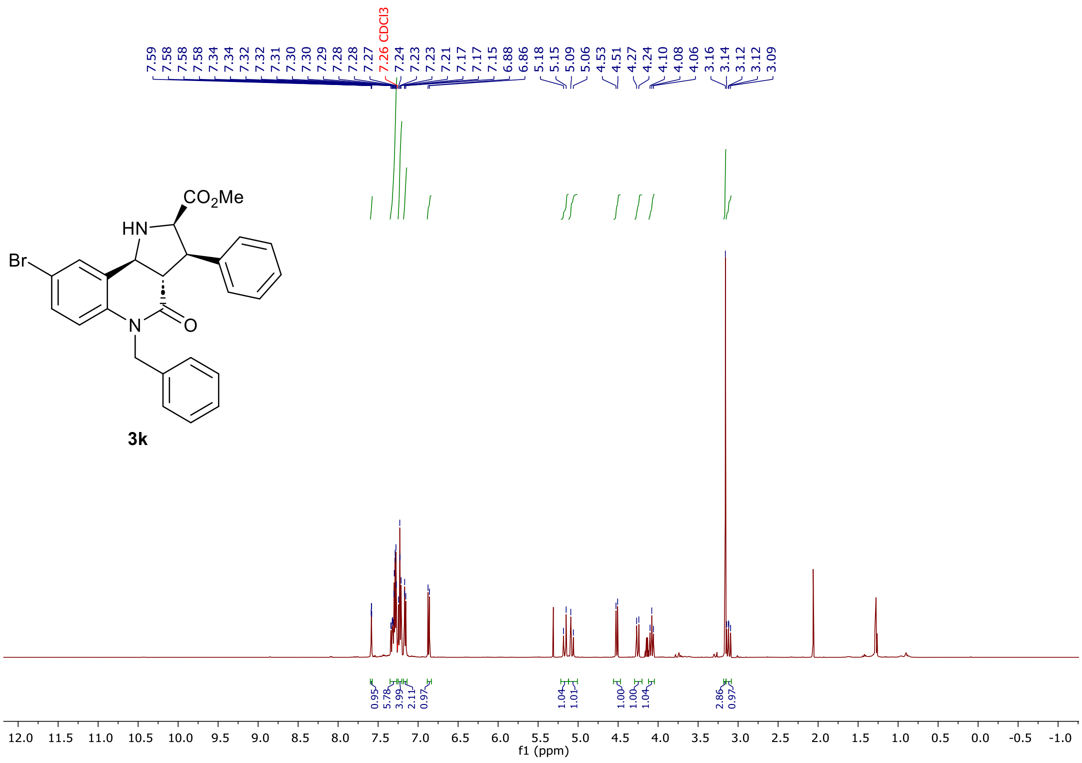

^{13}C NMR Spectrum of **3k** (126 MHz, $CDCl_3$)

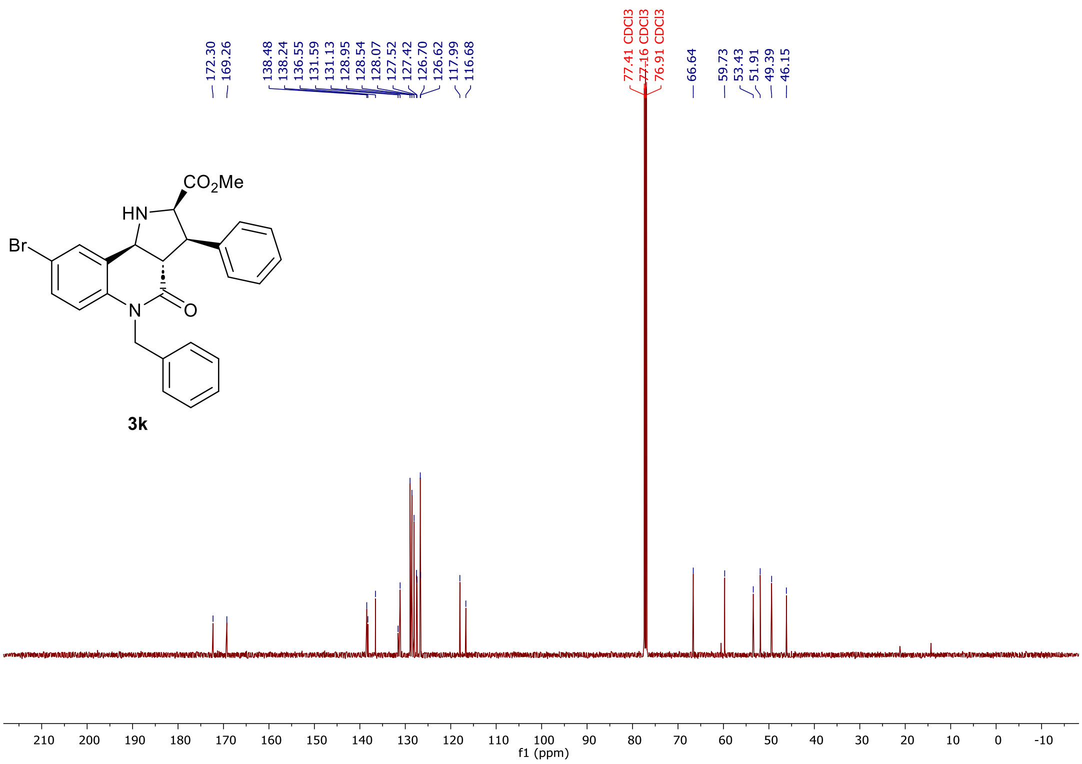

3k HPLC traces: racemate top, enantiomer bottom.

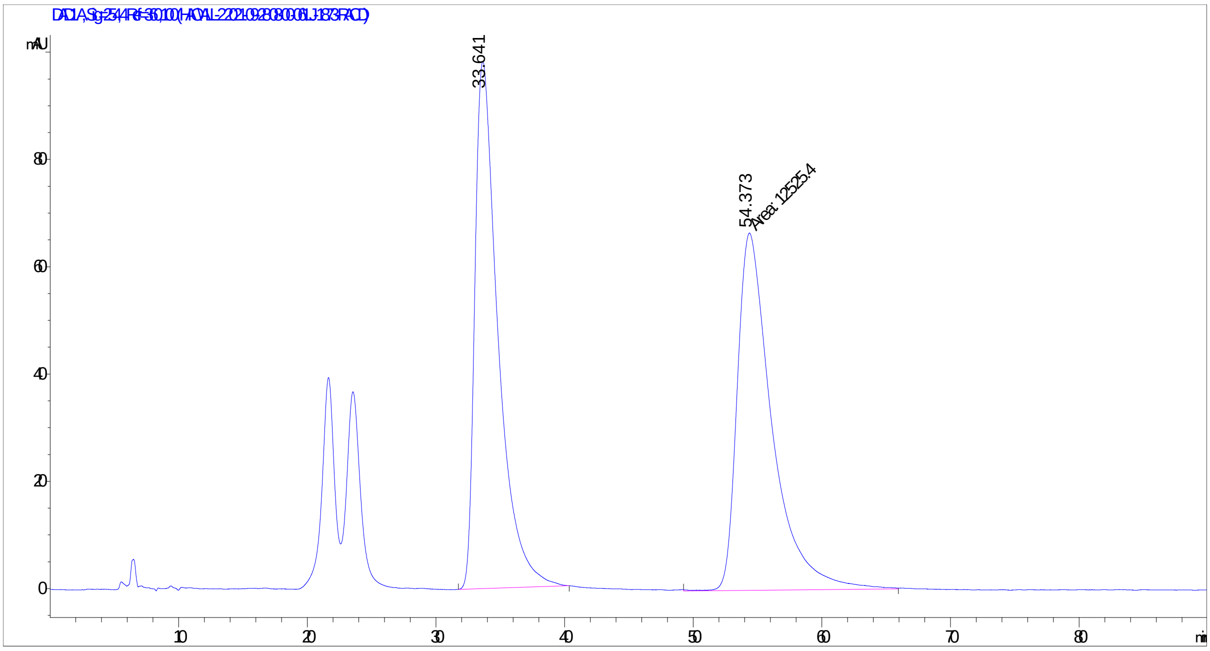

#	Time	Area	Height	Width	Area%	Symmetry
1	33.641	12329.3	98.3	1.7655	49.606	0.479
2	54.373	12525.4	66.6	3.1329	50.394	0.521

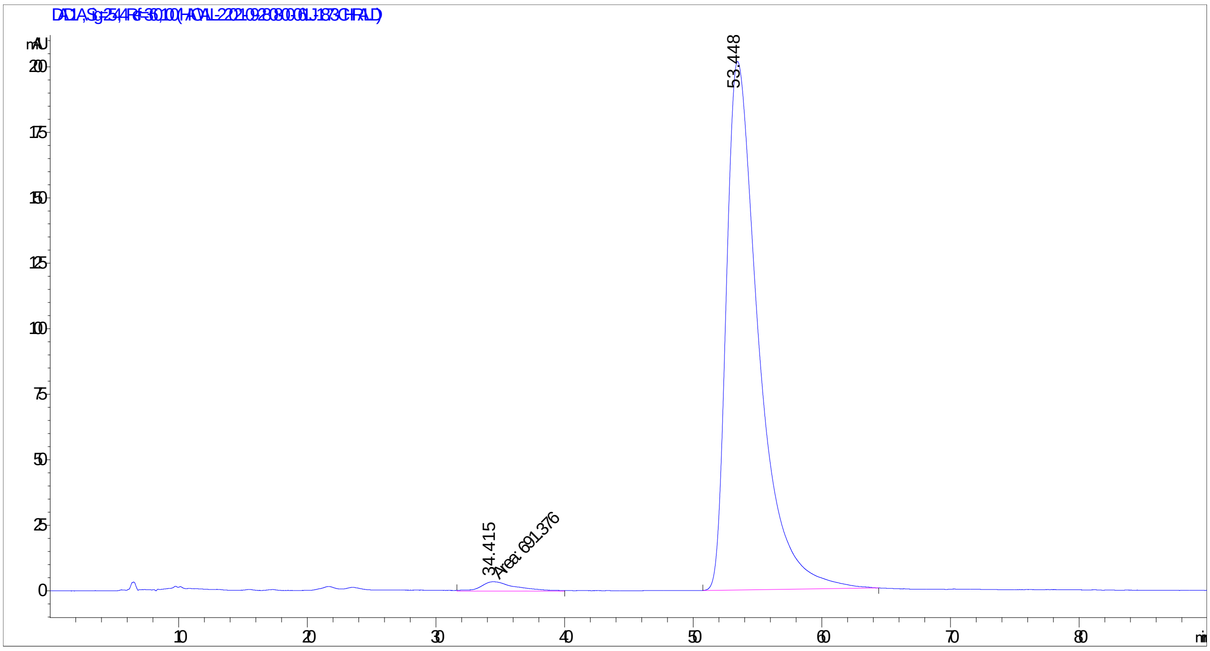

#	Time	Area	Height	Width	Area%	Symmetry
1	34.415	691.4	3.6	3.2105	1.928	0.517
2	53.448	35161.1	201.7	2.4514	98.072	0.488

^{1}H NMR Spectrum of 3l (500 MHz, $CDCl_3$)

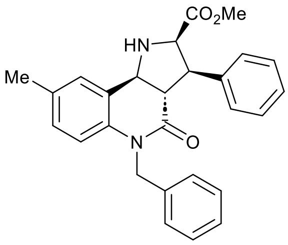

3l

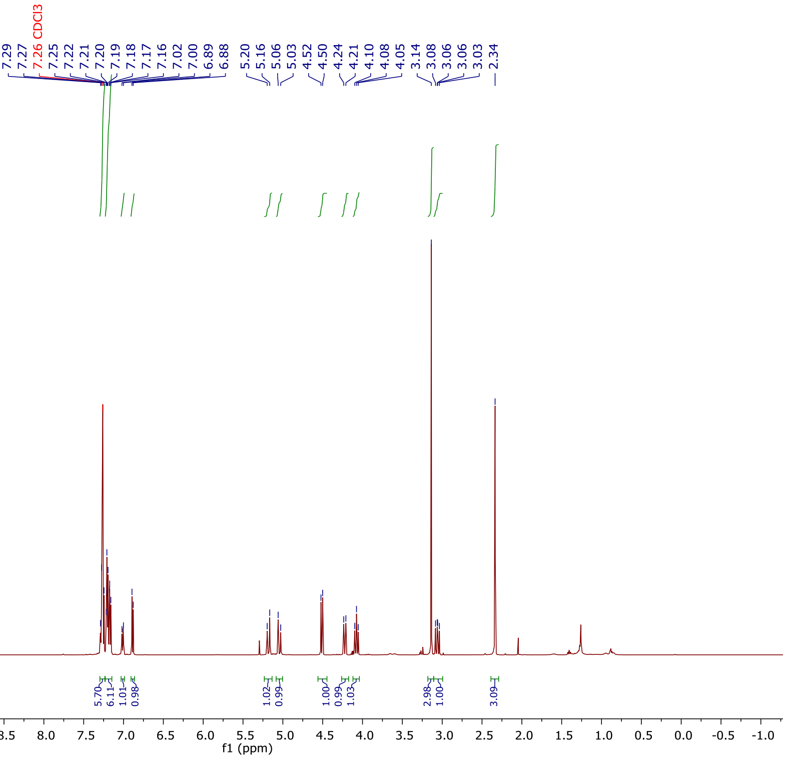

^{13}C NMR Spectrum of 3l (126 MHz, $CDCl_3$)

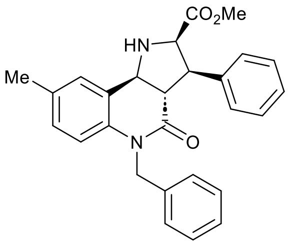

3l

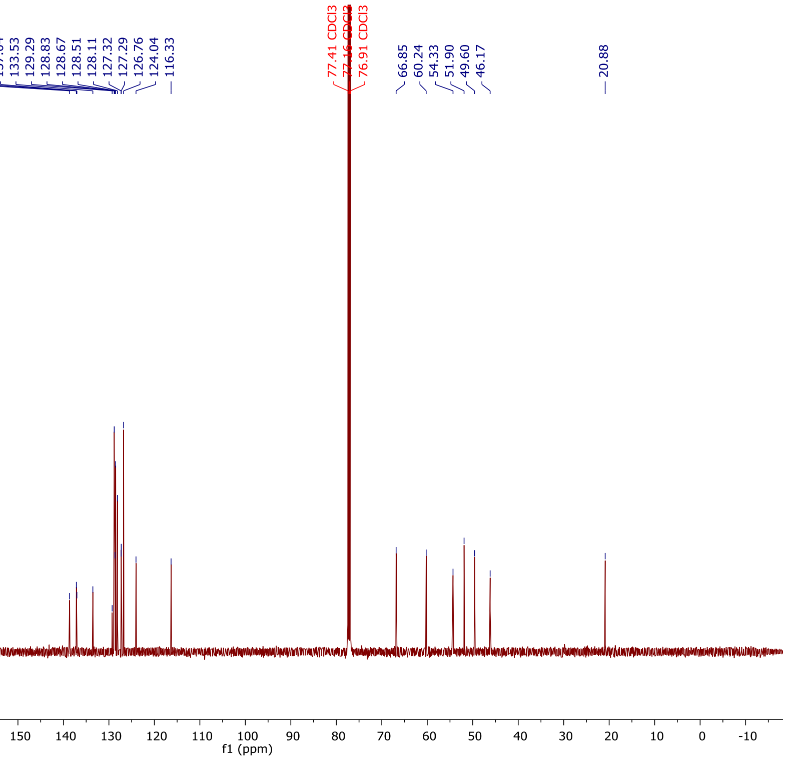

3l HPLC traces: racemate top, enantiomer bottom.

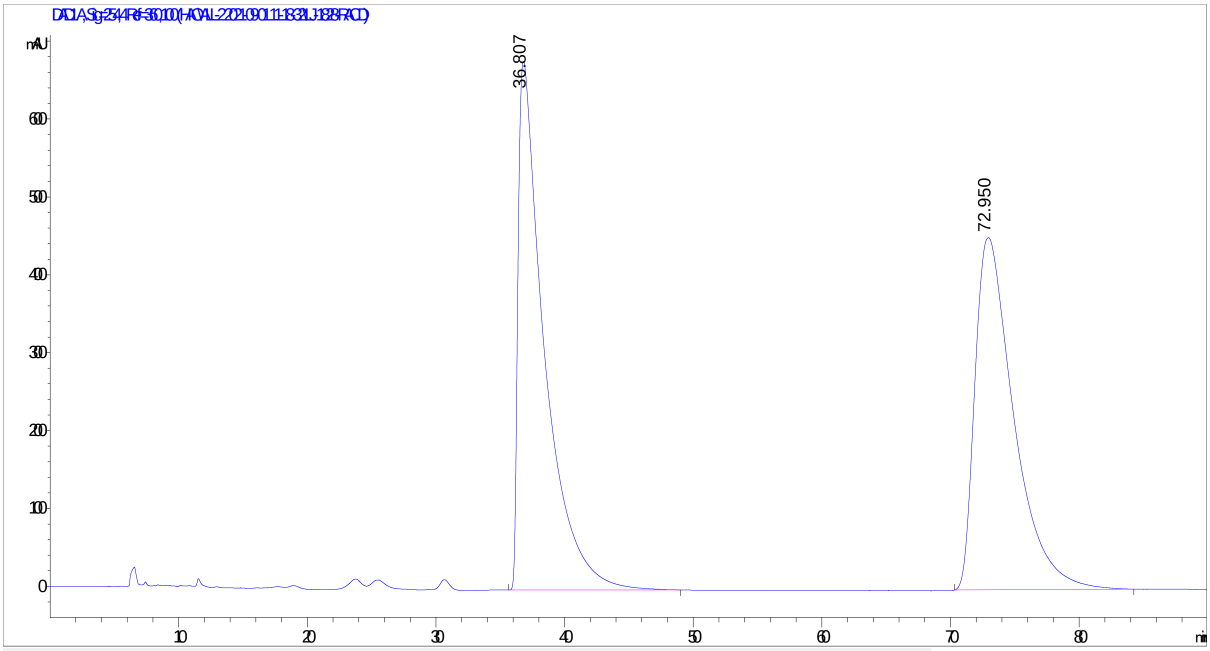

#	Time	Area	Height	Width	Area%	Symmetry
1	36.807	95941.4	678.7	1.9292	50.268	0.235
2	72.95	94916.9	452.1	2.7516	49.732	0.486

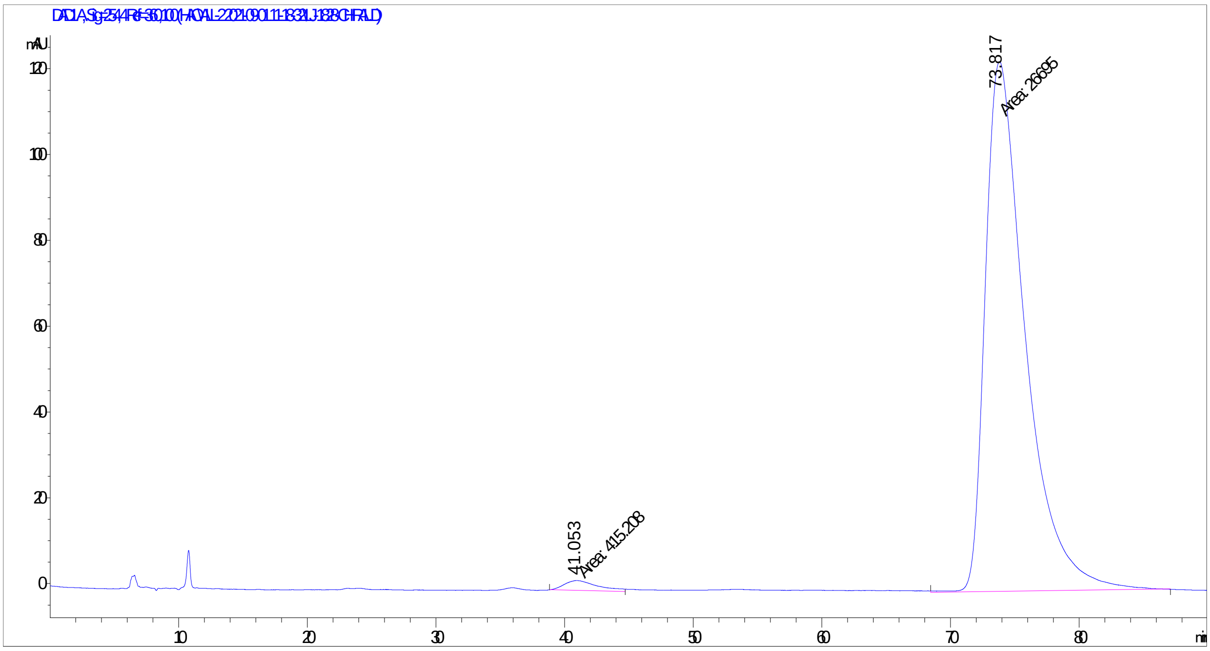

#	Time	Area	Height	Width	Area%	Symmetry
1	41.053	415.2	2.3	3.0541	1.532	0.605
2	73.817	26695	123.4	3.6041	98.468	0.53

^{1}H NMR Spectrum of **3m** (400 MHz, $CDCl_3$)

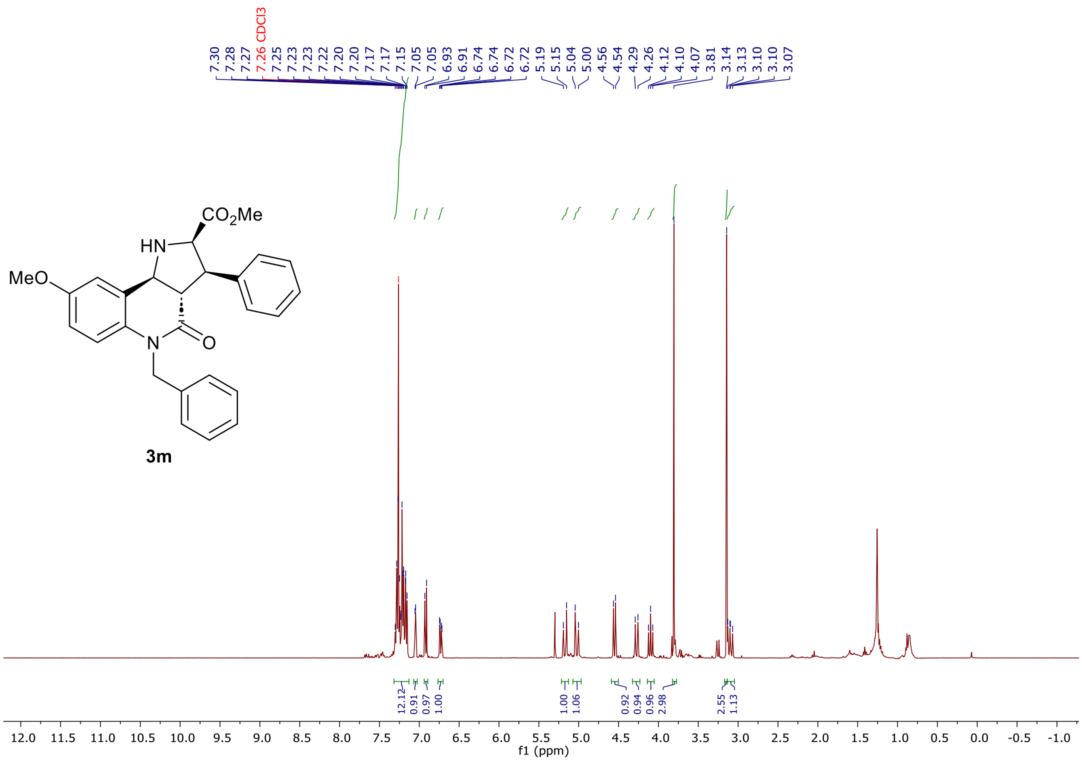

^{13}C NMR Spectrum of **3m** (101 MHz, $CDCl_3$)

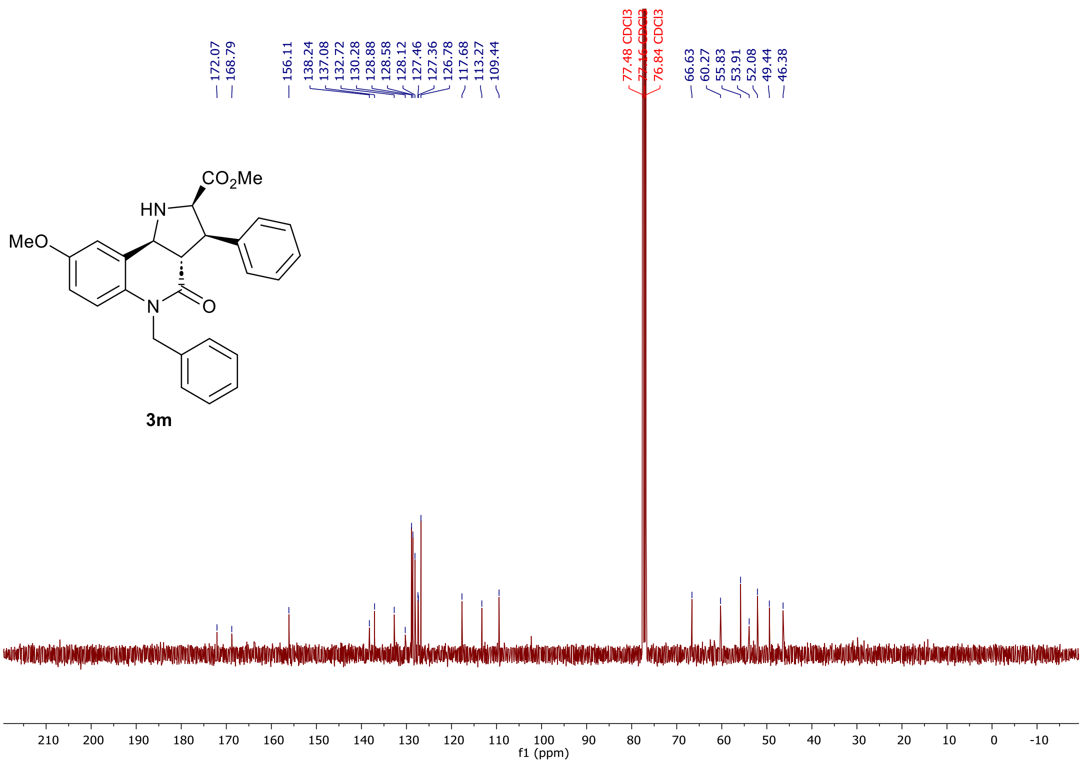

3m HPLC traces: racemate top, enantiomer bottom.

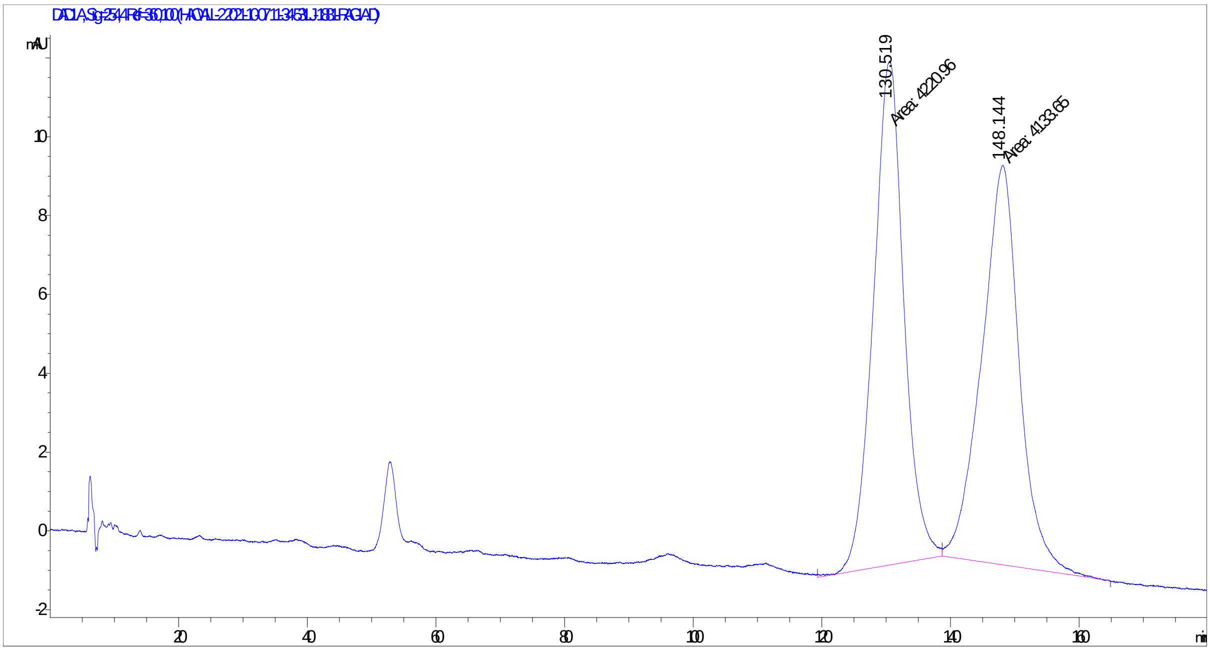

#	Time	Area	Height	Width	Area%	Symmetry
1	130.519	4221	12.8	5.5038	50.523	1.073
2	148.144	4133.6	10.1	6.7978	49.477	1.199

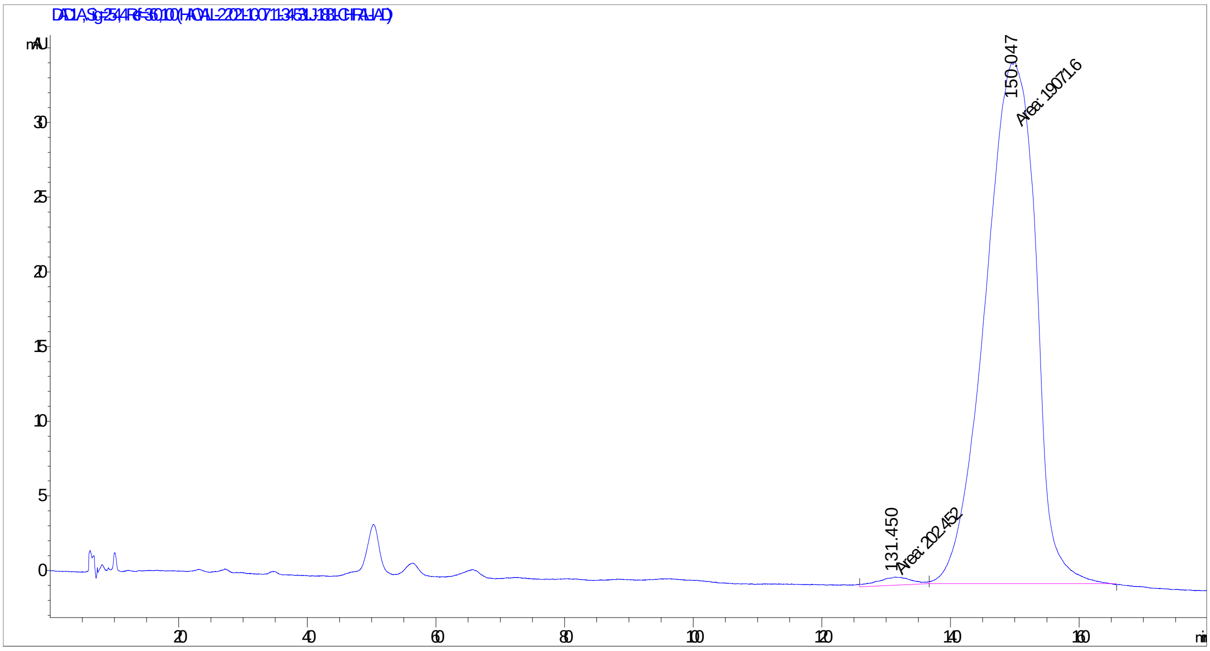

#	Time	Area	Height	Width	Area%	Symmetry
1	131.45	202.5	5.5E-1	6.1532	1.050	1.179
2	150.047	19071.6	35	9.0888	98.950	1.376

^{1}H NMR Spectrum of 3n (500 MHz, $CDCl_3$)

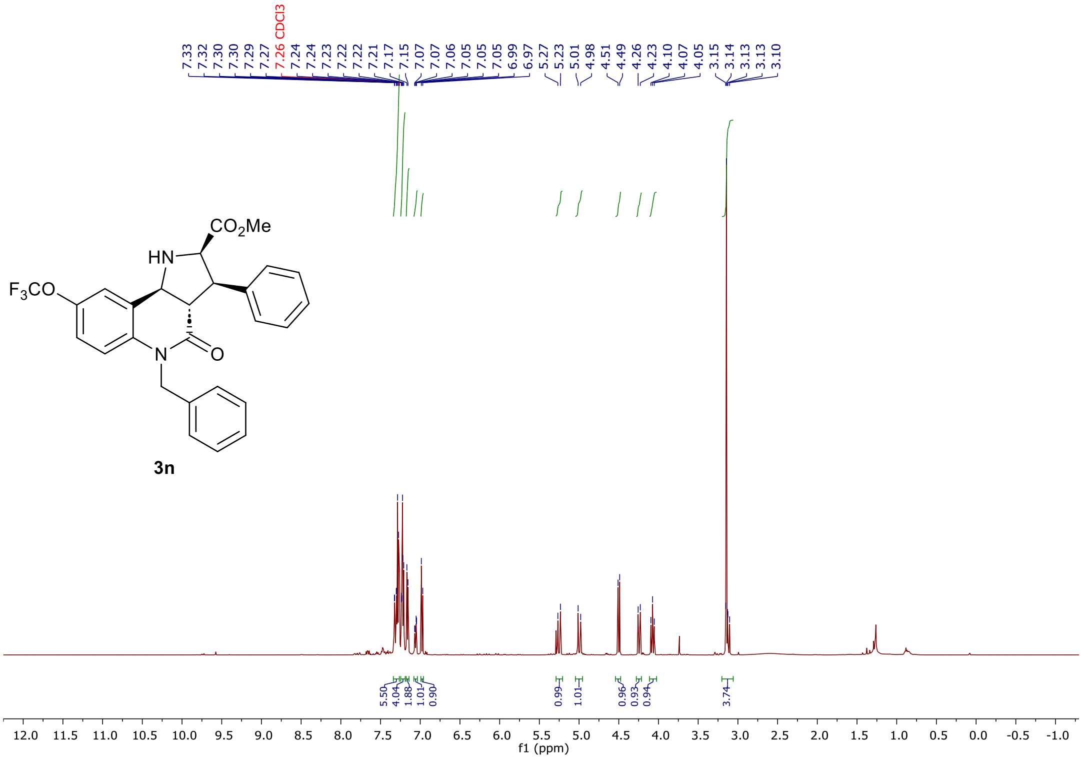

^{13}C NMR Spectrum of 3n (126 MHz, $CDCl_3$)

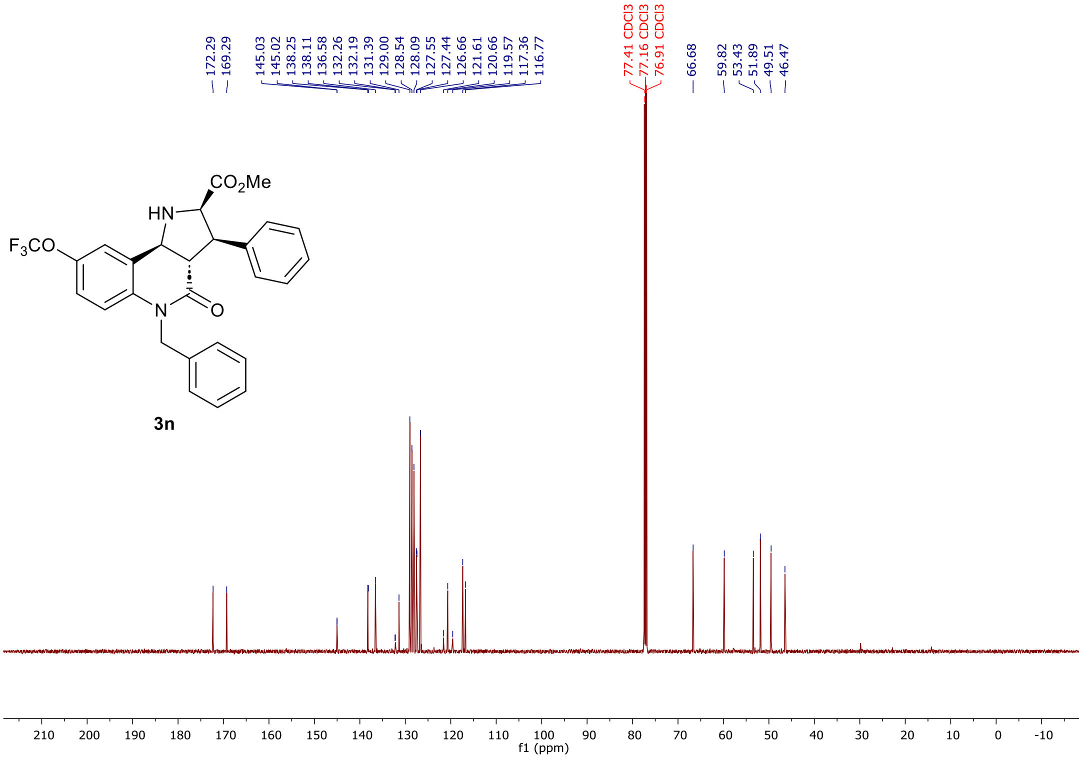

^{19}F NMR Spectrum of **3n** (470 MHz, $CDCl_3$)

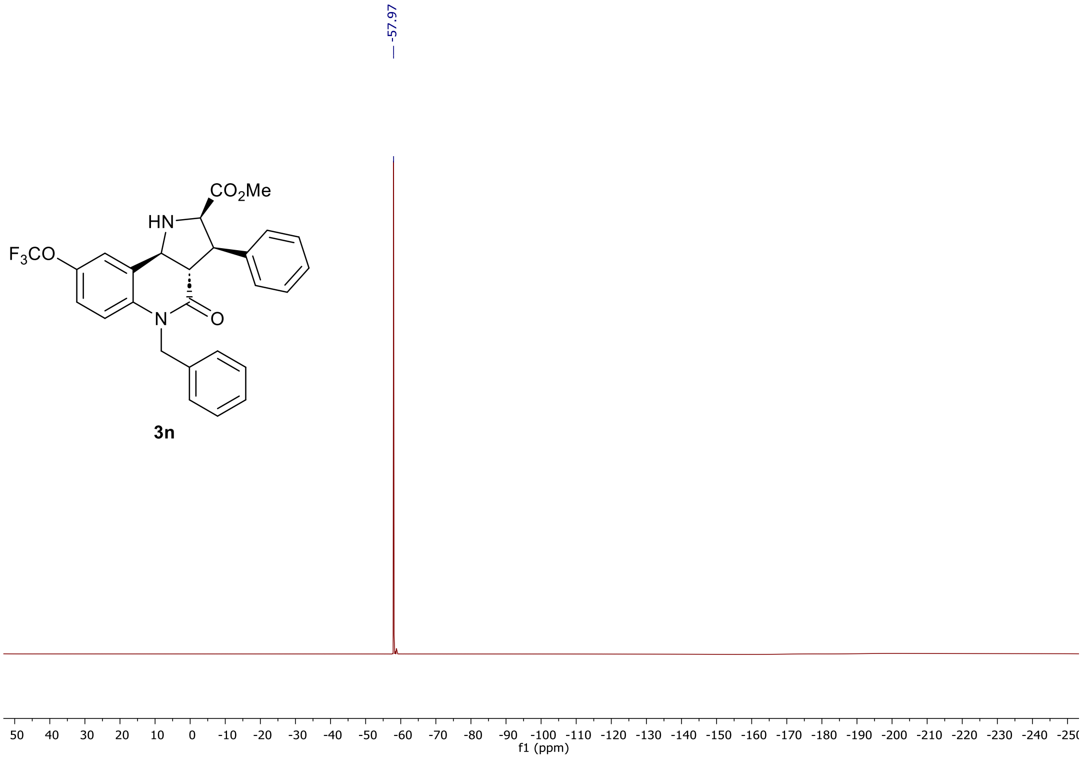

3n

3n HPLC traces: racemate top, enantiomer bottom.

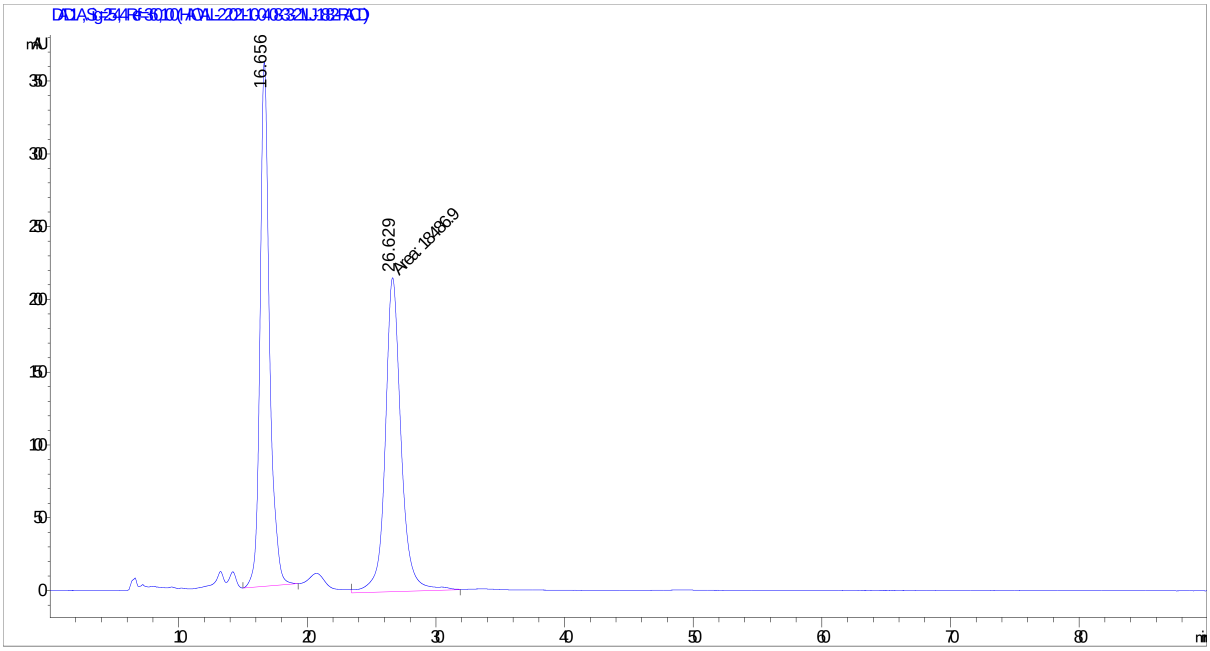

#	Time	Area	Height	Width	Area%	Symmetry
1	16.656	18408	360.6	0.7636	49.893	0.767
2	26.629	18486.9	215.7	1.4285	50.107	0.839

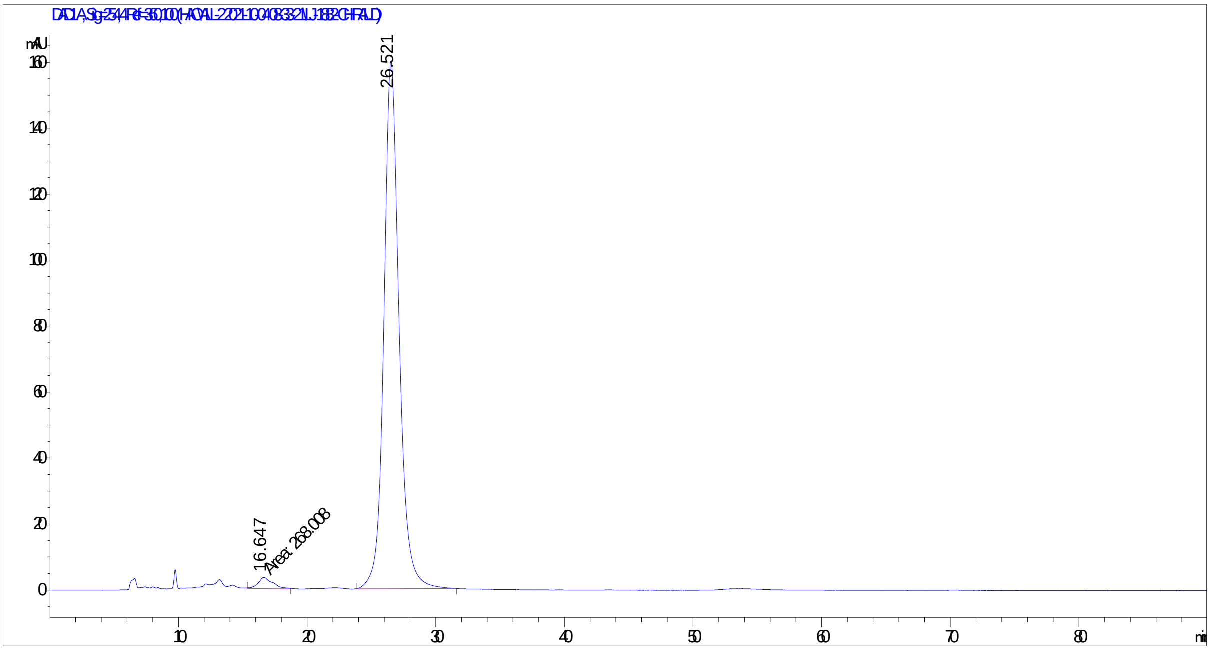

#	Time	Area	Height	Width	Area%	Symmetry
1	16.647	268	3.4	1.3095	1.996	0.647
2	26.521	13159.5	159.9	1.2221	98.004	0.83

^{1}H NMR Spectrum of **3o** (500 MHz, $CDCl_3$)

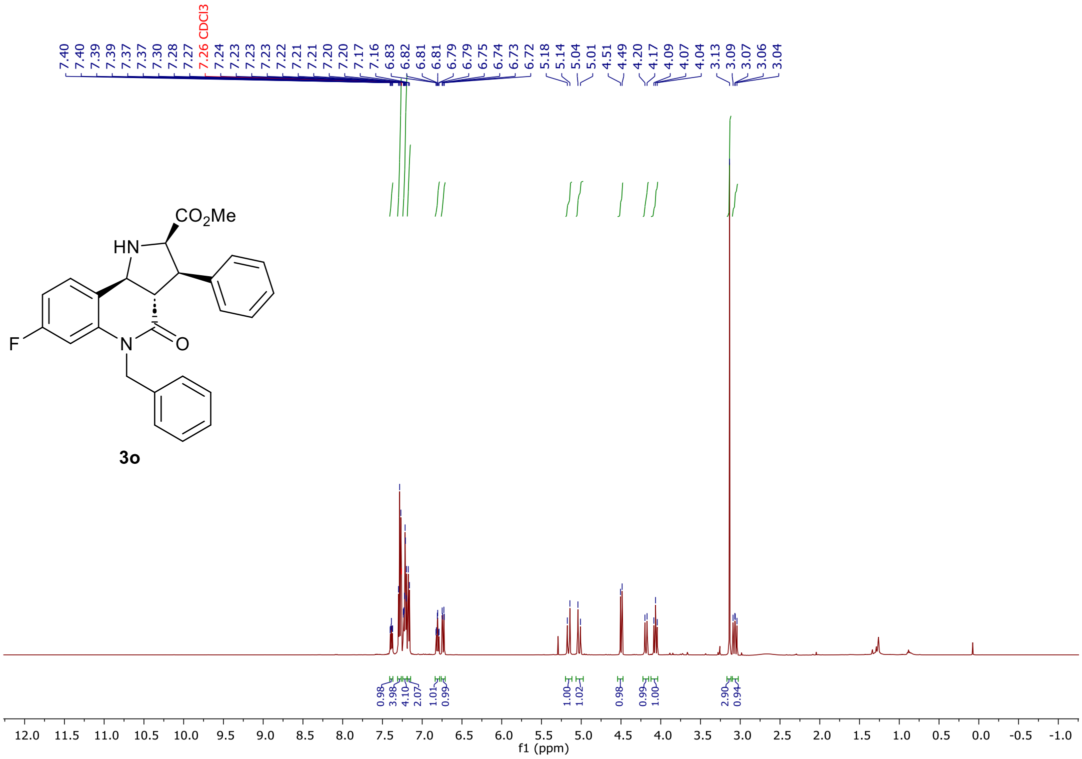

^{13}C NMR Spectrum of **3o** (126 MHz, $CDCl_3$)

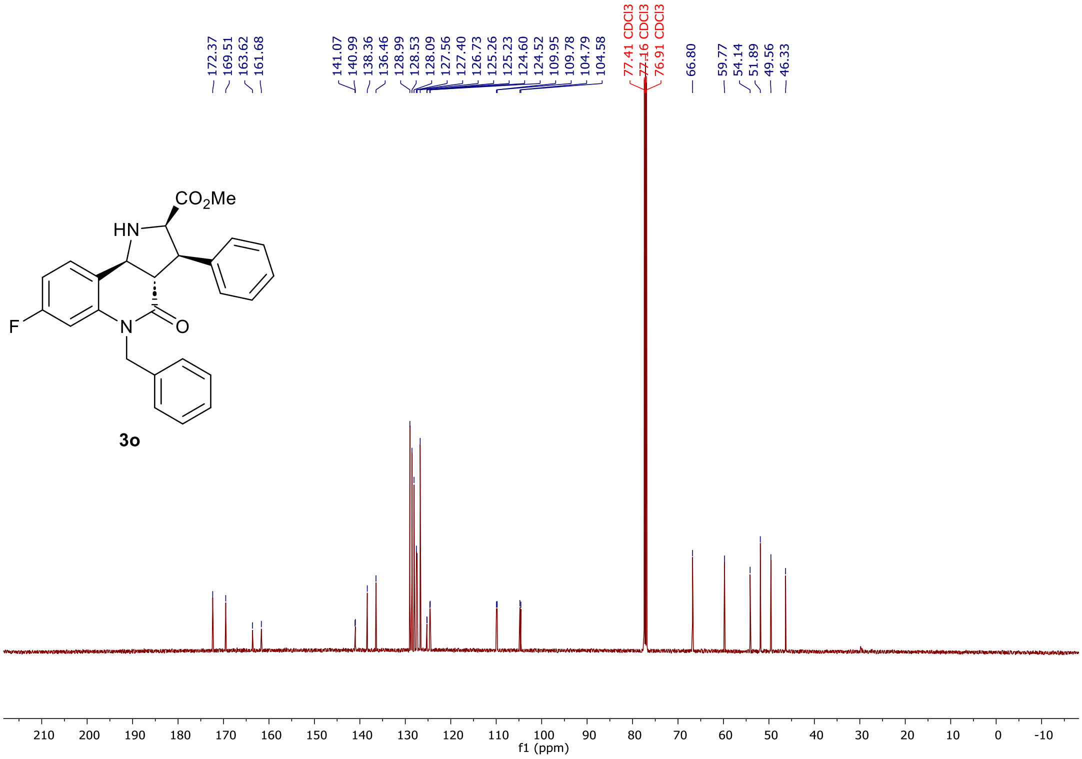

^{19}F NMR Spectrum of **3o** (470 MHz, $CDCl_3$)

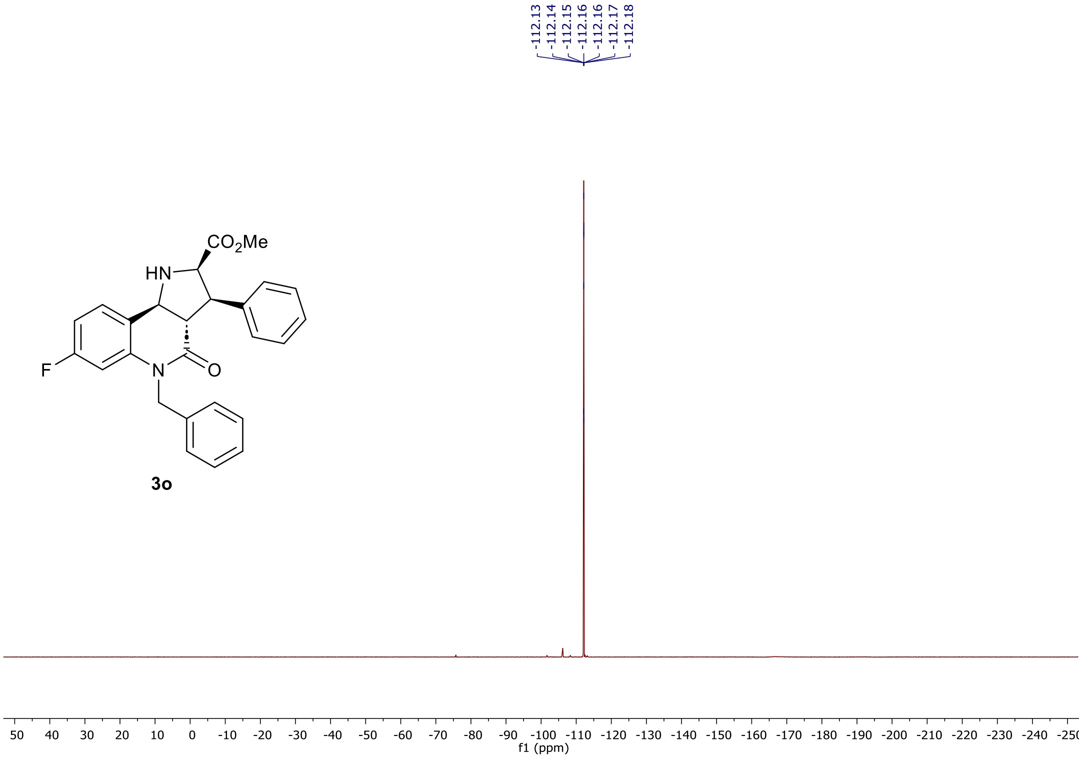

3o HPLC traces: racemate top, enantiomer bottom.

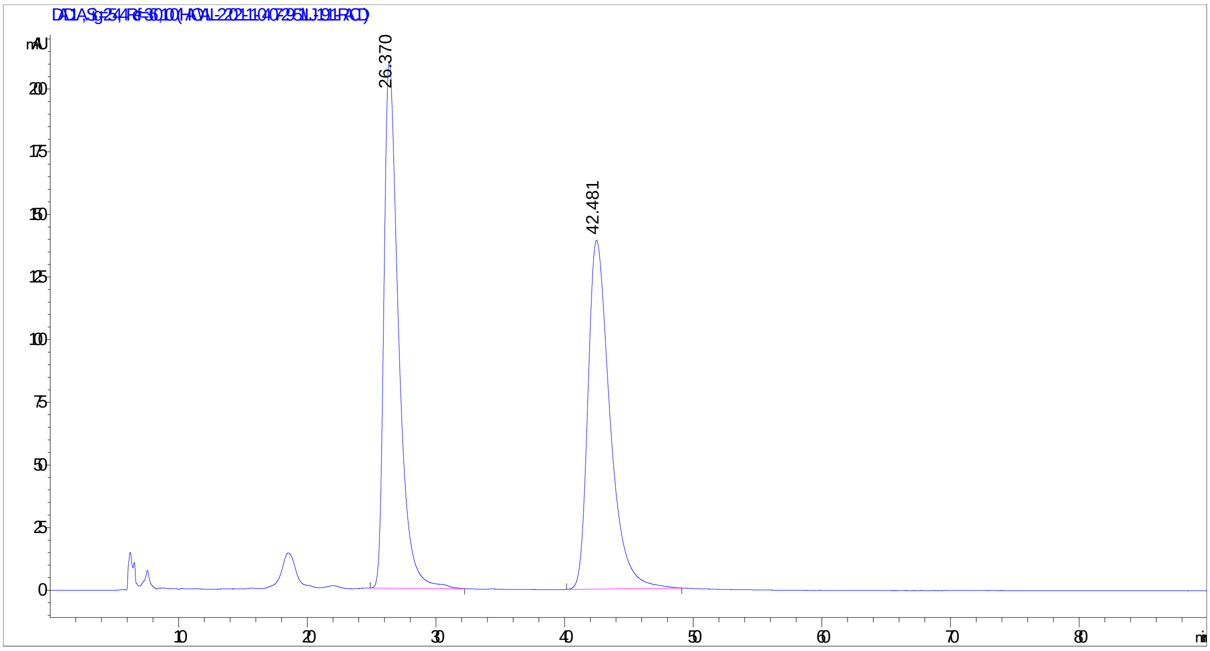

#	Time	Area	Height	Width	Area%	Symmetry
1	26.37	16615.6	210.3	1.172	50.123	0.528
2	42.481	16534.3	139.3	1.709	49.877	0.596

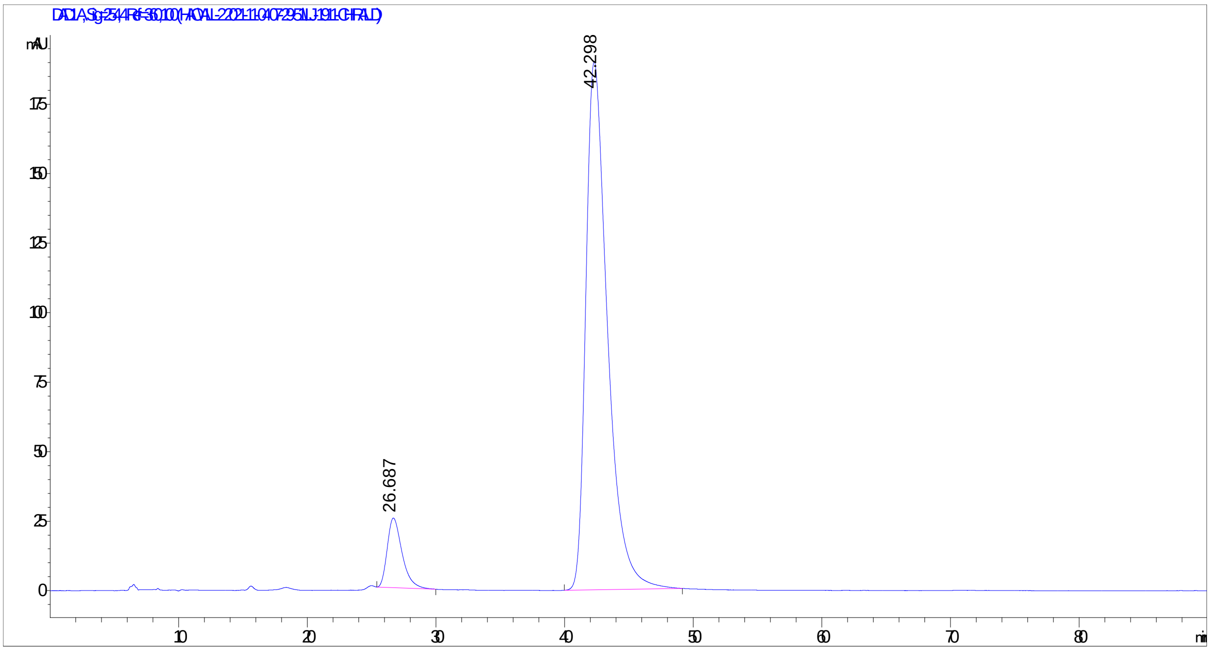

#	Time	Area	Height	Width	Area%	Symmetry
1	26.687	2006.2	25.1	1.1203	8.279	0.658
2	42.298	22227.3	190.1	1.716	91.721	0.58

^{1}H NMR Spectrum of **3p** (700 MHz, $CDCl_3$)

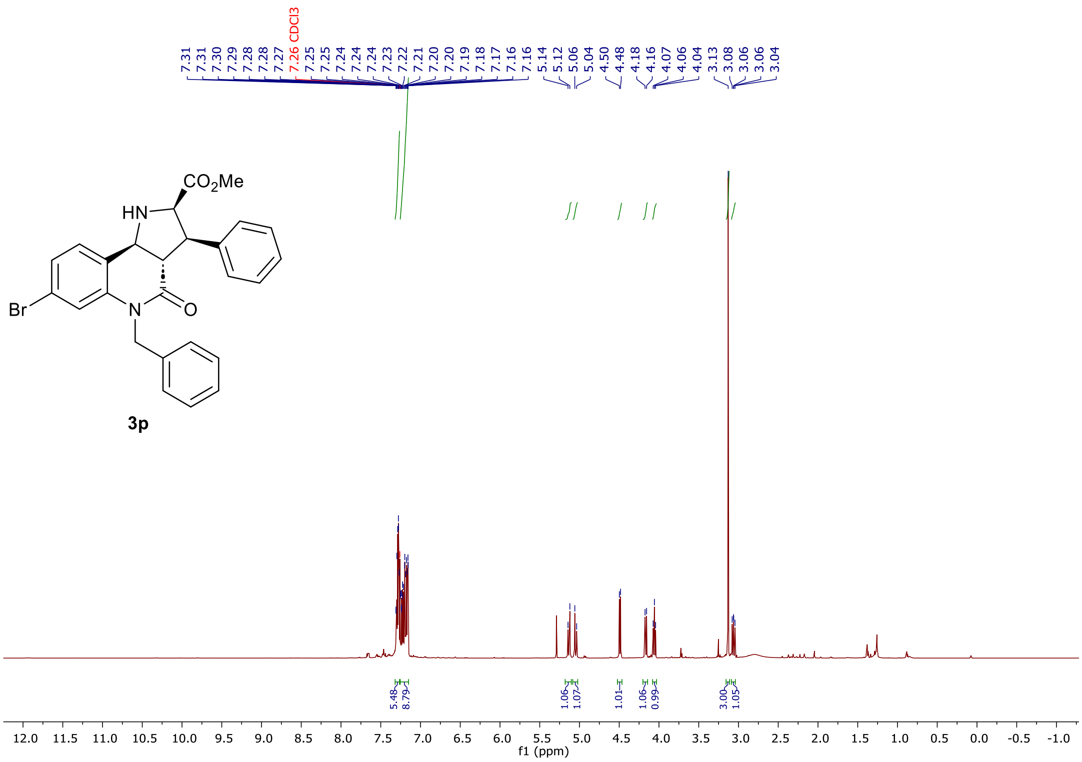

^{13}C NMR Spectrum of **3p** (176 MHz, $CDCl_3$)

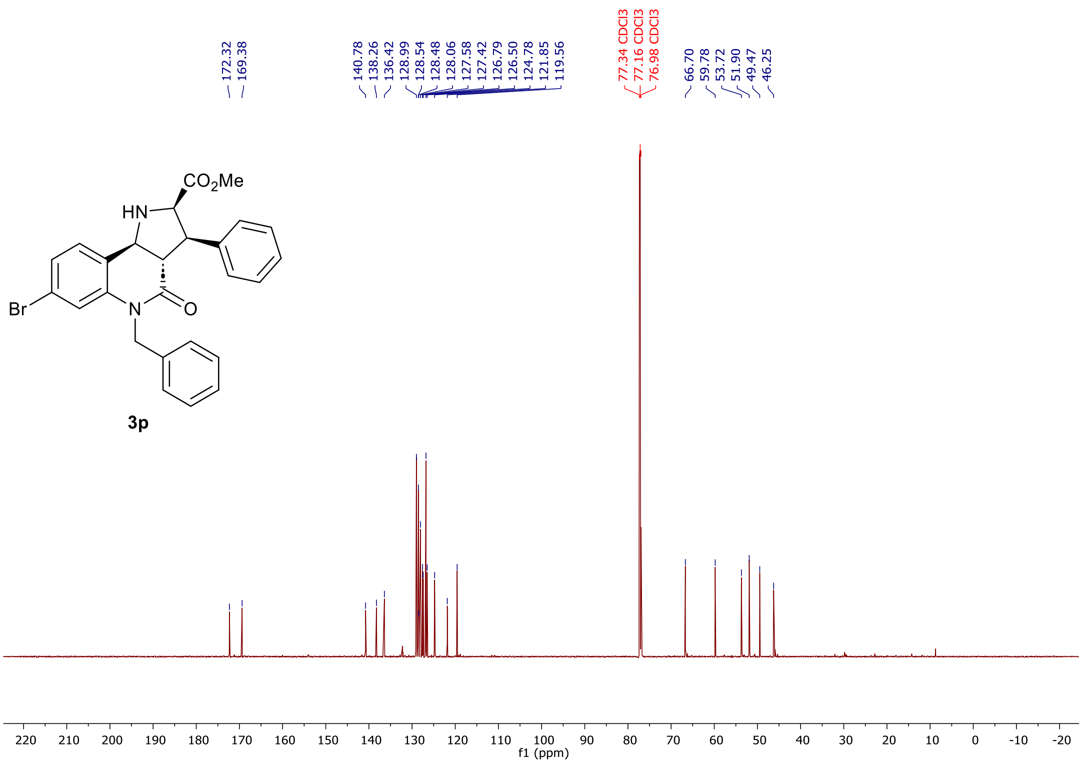

3p HPLC traces: racemate top, enantiomer bottom.

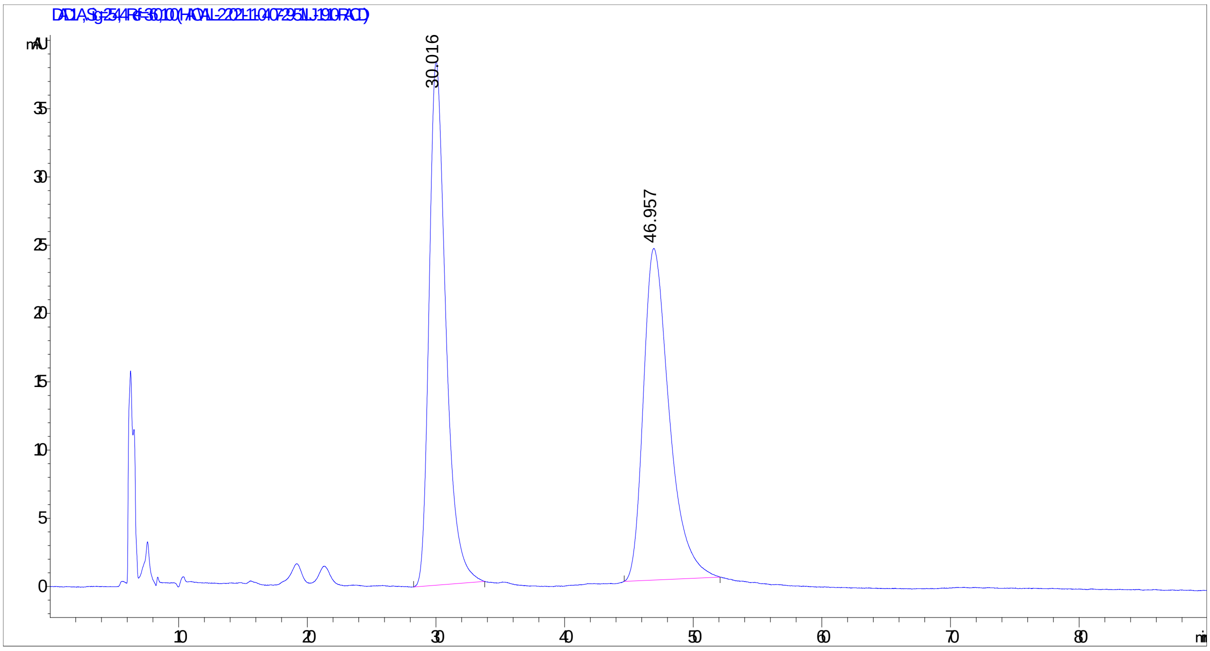

#	Time	Area	Height	Width	Area%	Symmetry
1	30.016	3478.1	38.4	1.2934	50.561	0.674
2	46.957	3400.9	24.3	1.6514	49.439	0.671

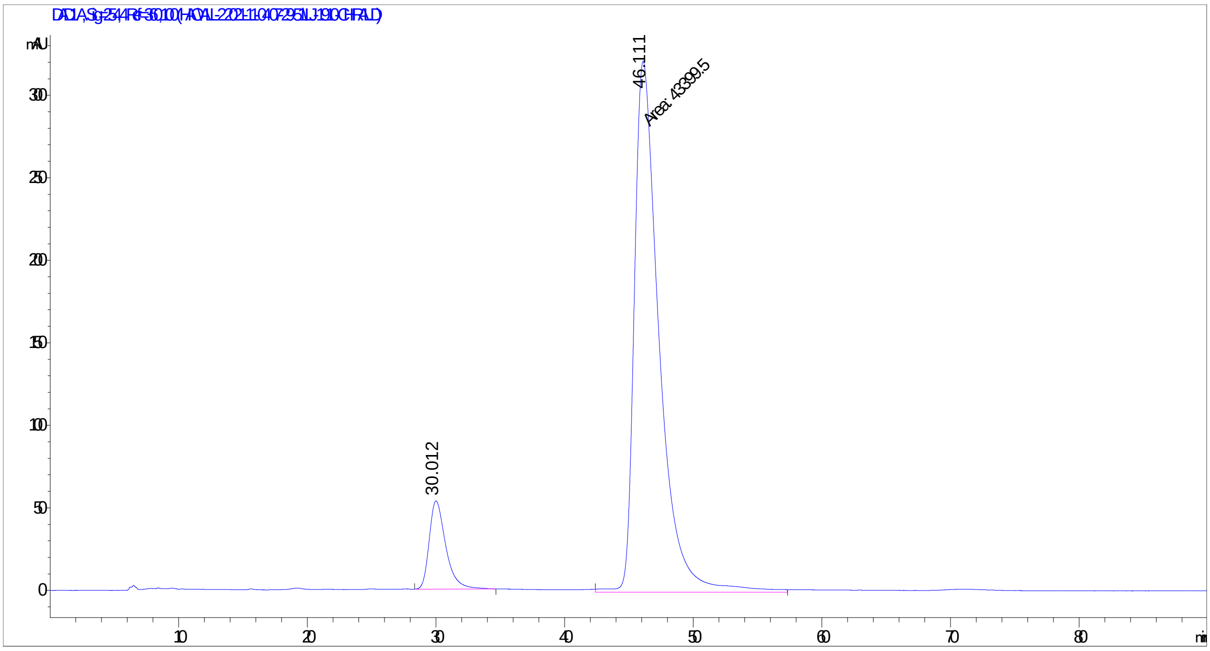

#	Time	Area	Height	Width	Area%	Symmetry
1	30.012	4776.3	53.5	1.3005	9.914	0.661
2	46.111	43399.5	321.7	2.2487	90.086	0.524

^{1}H NMR Spectrum of **3q** (700 MHz, $CDCl_3$)

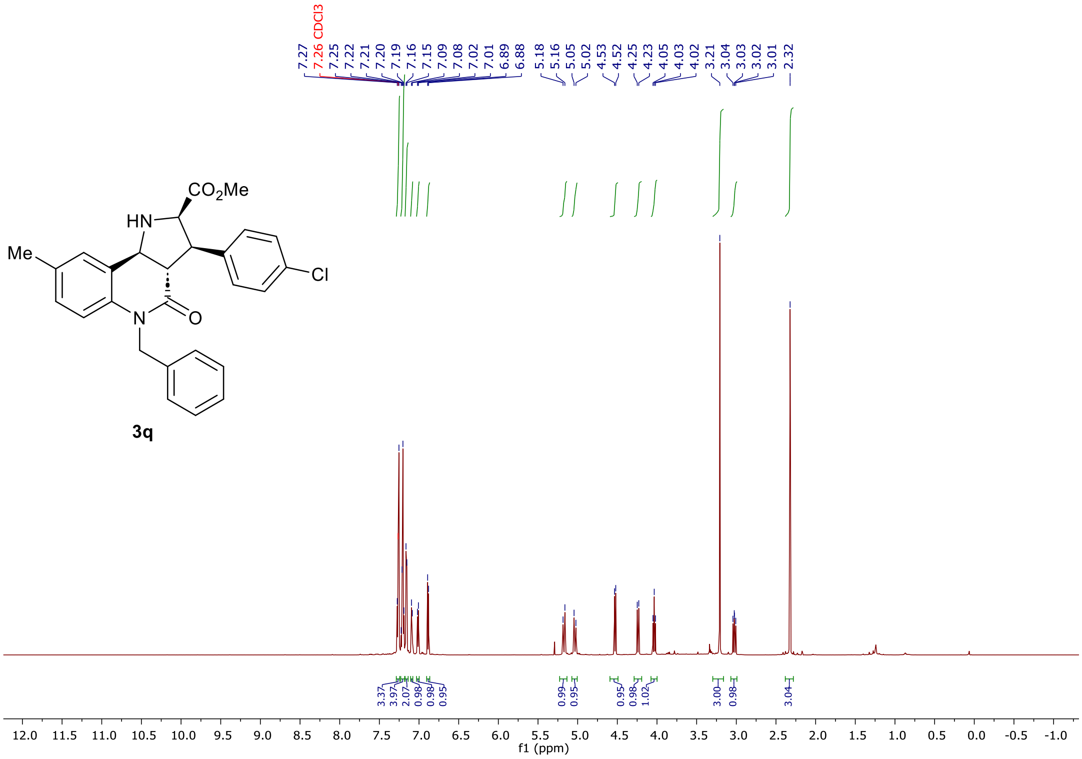

^{13}C NMR Spectrum of **3q** (176 MHz, $CDCl_3$)

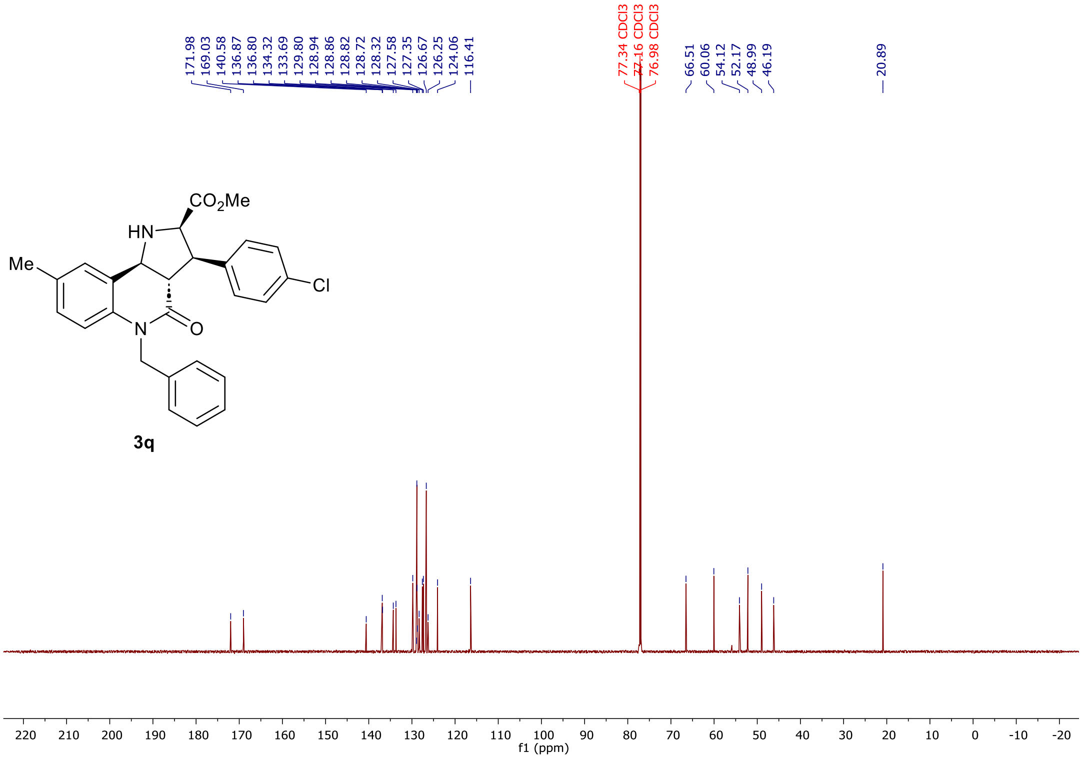

3q HPLC traces: racemate top, enantiomer bottom.

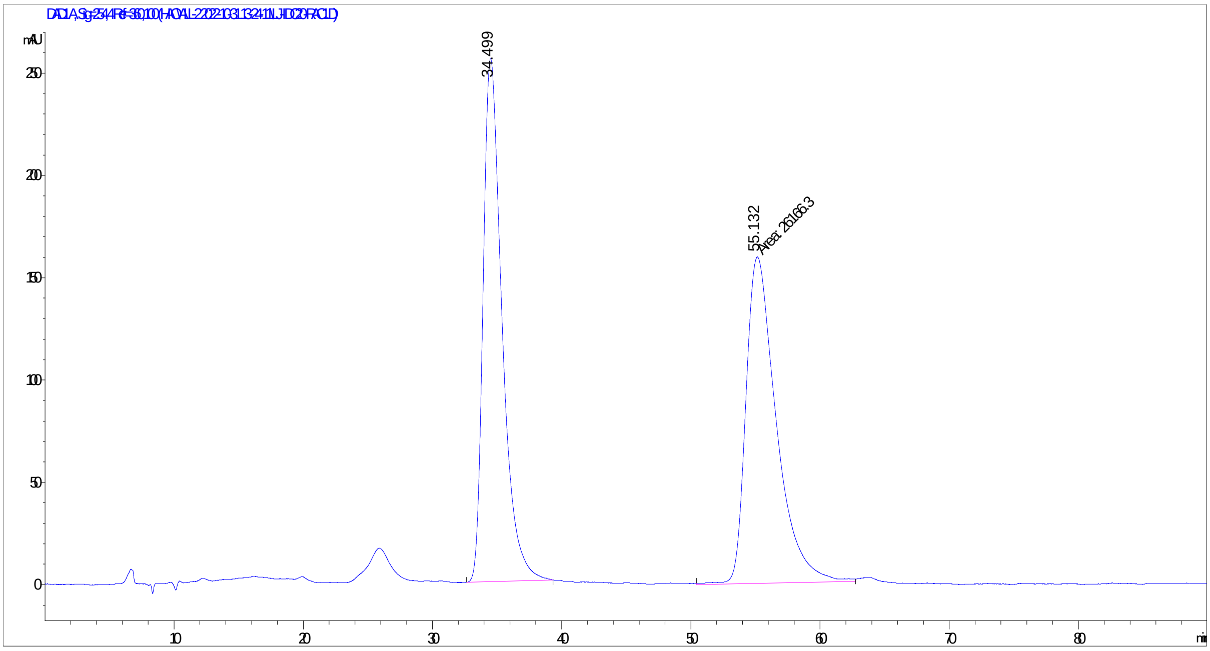

#	Time	Area	Height	Width	Area%	Symmetry
1	34.499	26015.5	255.8	1.4085	49.856	0.601
2	55.132	26166.3	159.6	2.7323	50.144	0.567

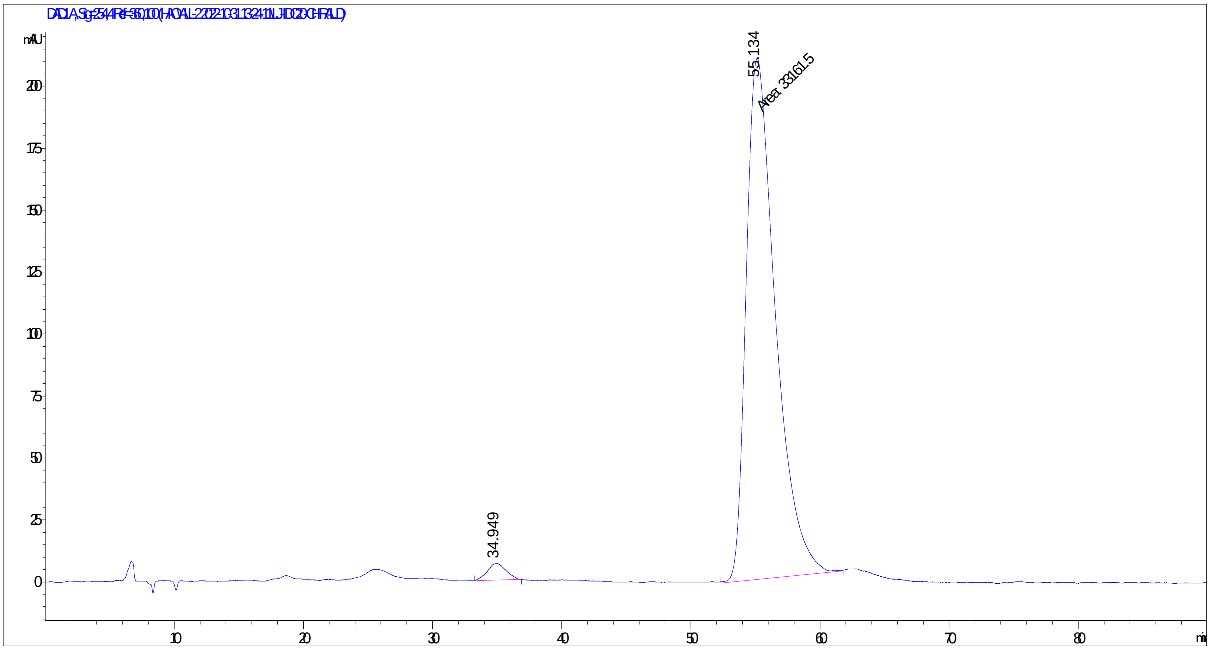

#	Time	Area	Height	Width	Area%	Symmetry
1	34.949	671.1	6.8	1.1767	1.984	0.941
2	55.134	33161.5	210.2	2.629	98.016	0.571

^{1}H NMR Spectrum of **3r** (700 MHz, $CDCl_3$)

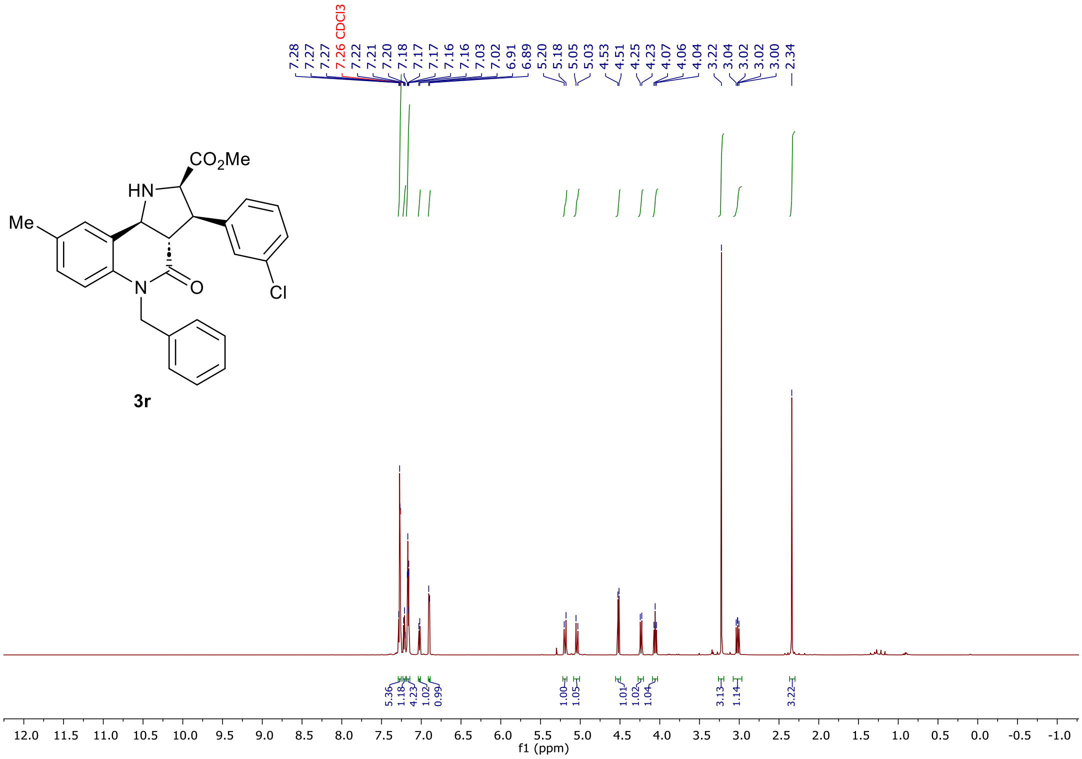

^{13}C NMR Spectrum of **3r** (176 MHz, $CDCl_3$)

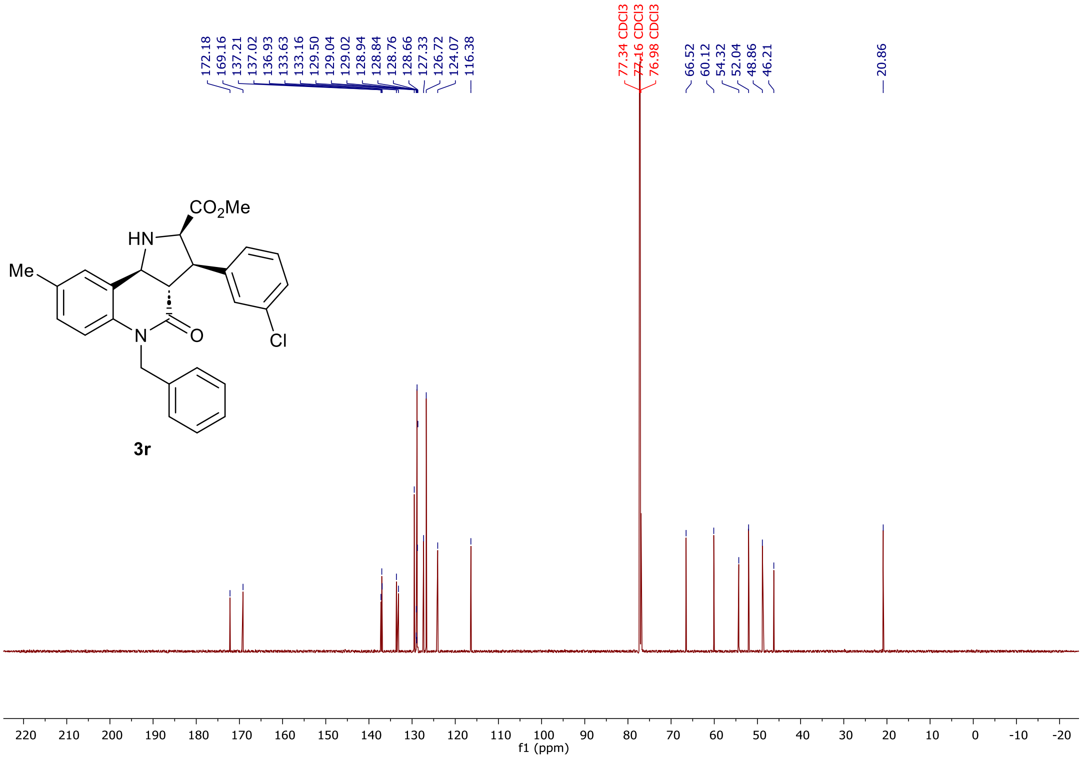

3r HPLC traces: racemate top, enantiomer bottom.

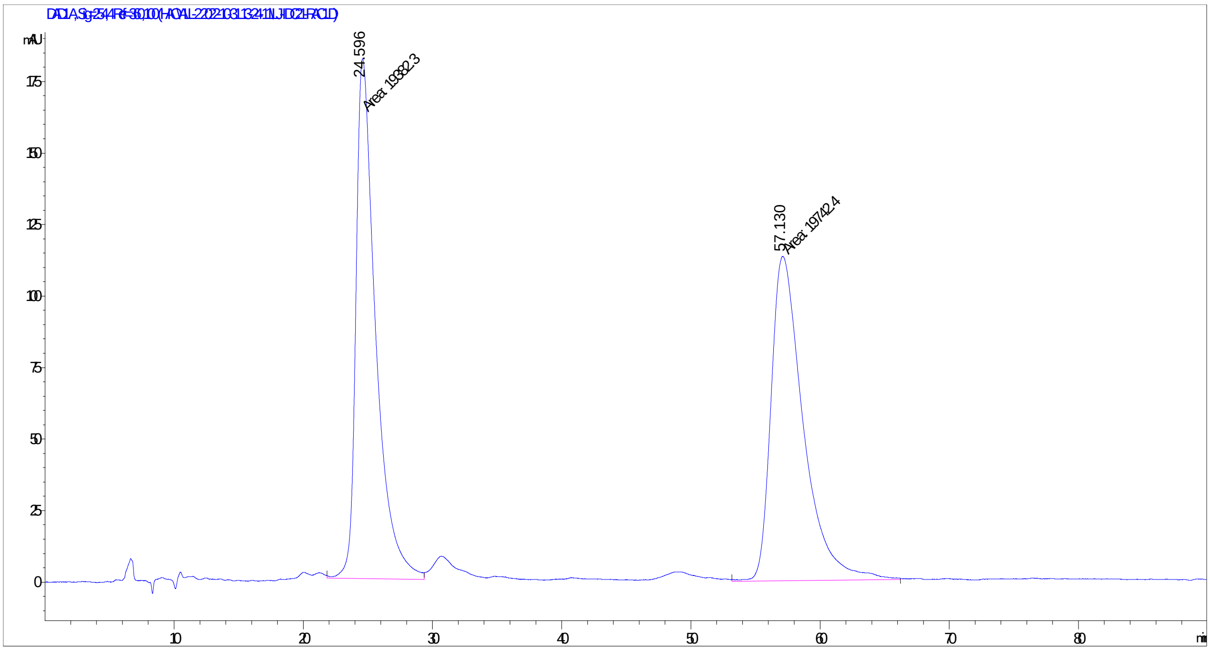

#	Time	Area	Height	Width	Area%	Symmetry
1	24.596	19382.3	181.8	1.7768	49.540	0.5
2	57.13	19742.4	113.4	2.9014	50.460	0.564

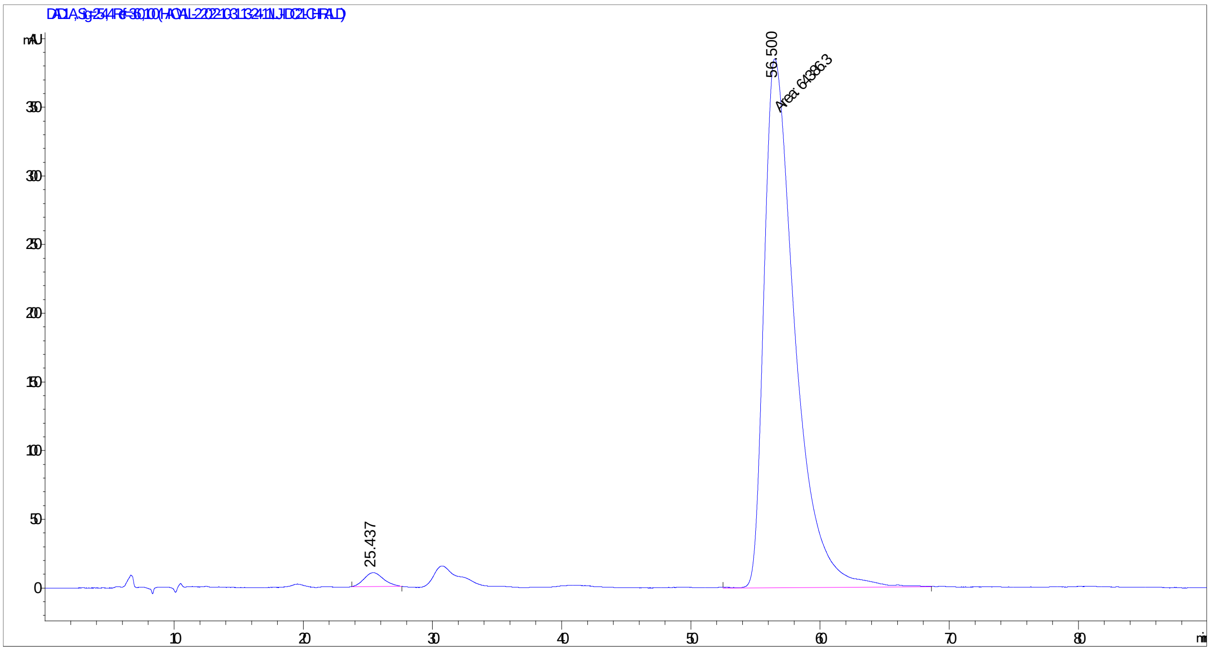

#	Time	Area	Height	Width	Area%	Symmetry
1	25.437	1052.6	10.1	1.2346	1.609	0.896
2	56.5	64386.3	385.3	2.7852	98.391	0.509

^{1}H NMR Spectrum of **3s** (700 MHz, $CDCl_3$)

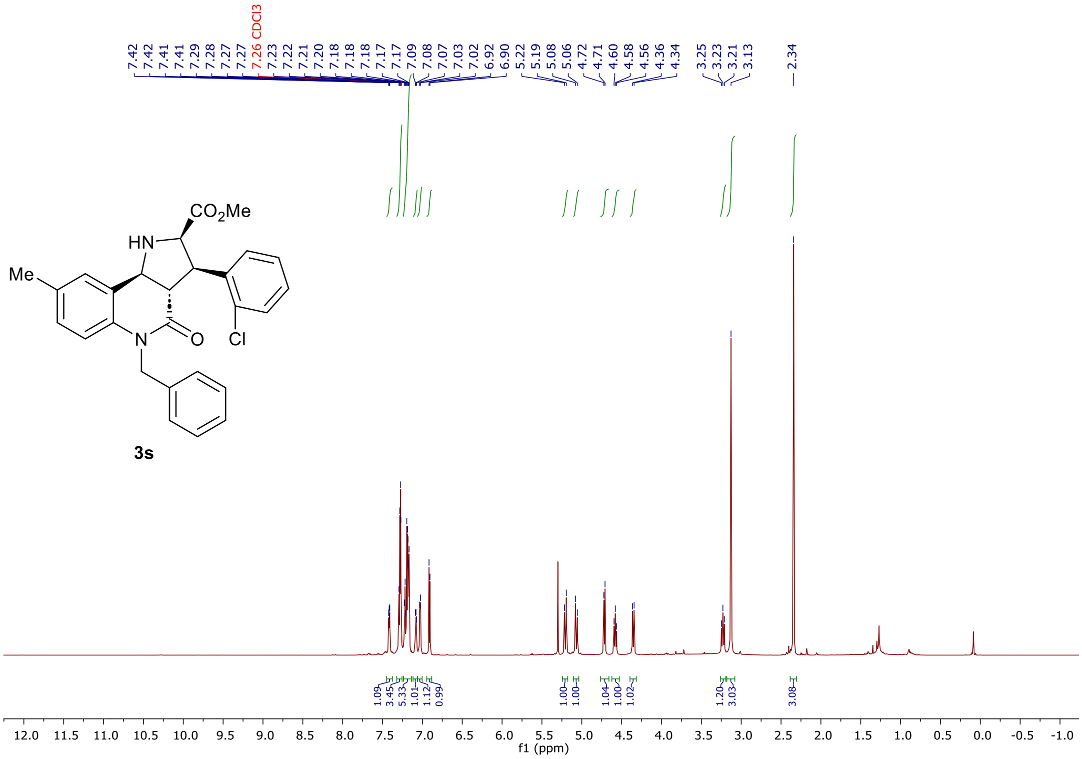

3s

^{13}C NMR Spectrum of **3s** (176 MHz, $CDCl_3$)

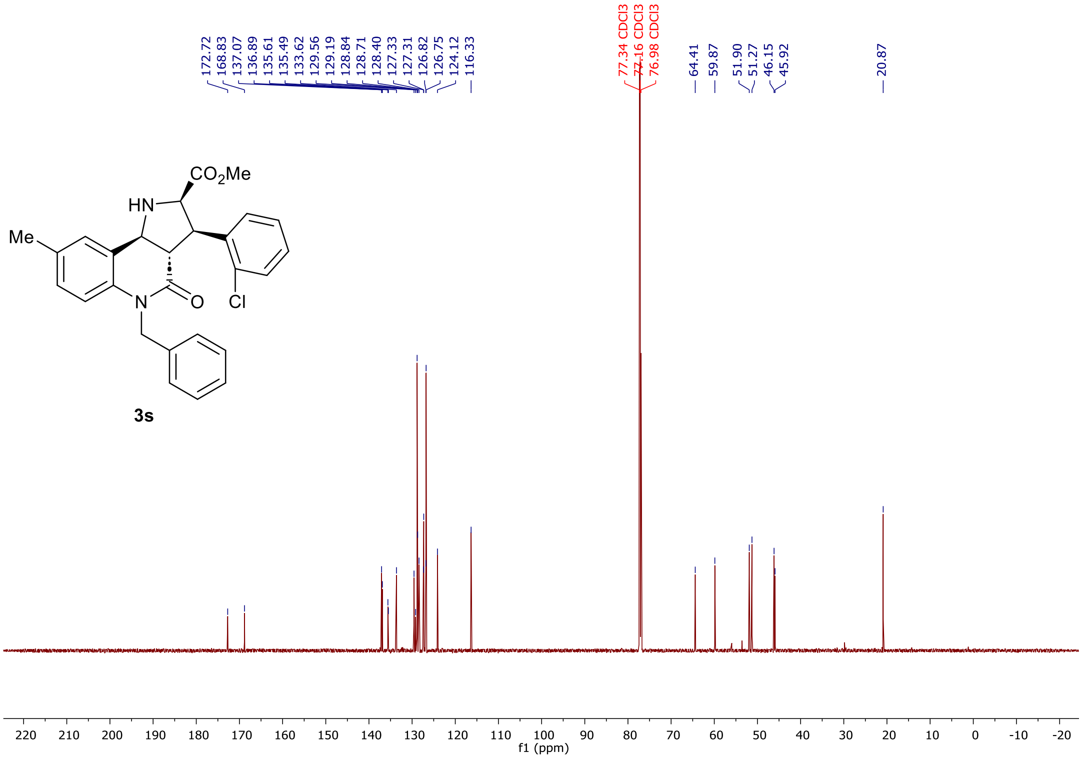

3s

3s HPLC traces: racemate top, enantiomer bottom.

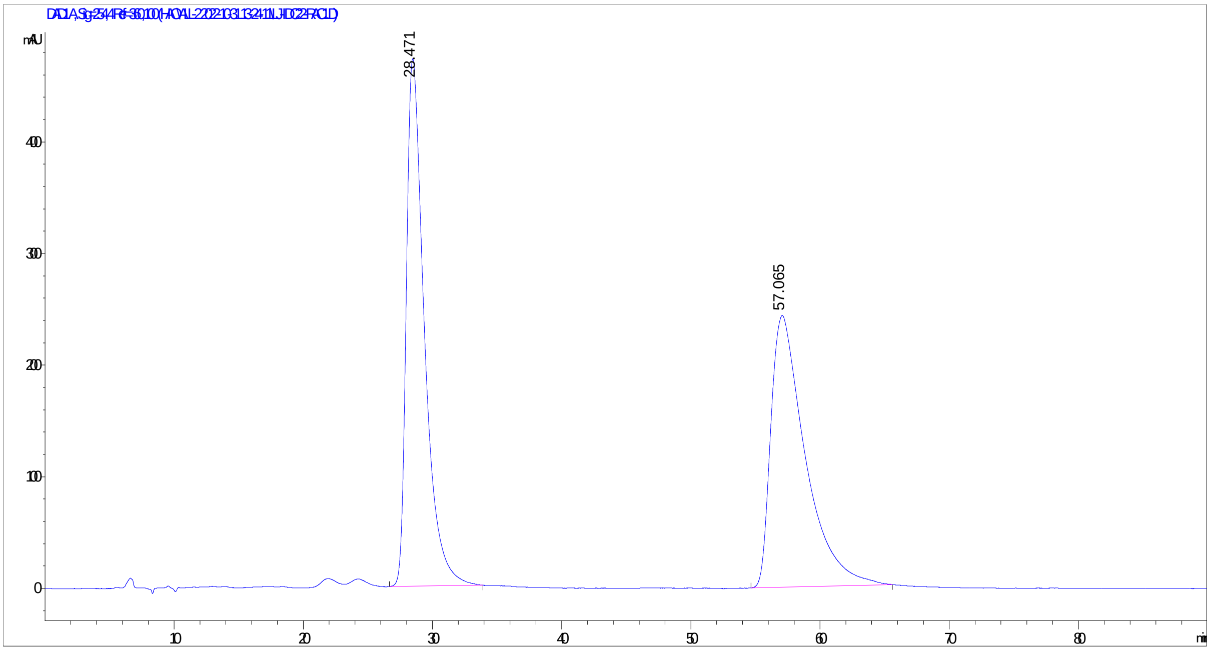

#	Time	Area	Height	Width	Area%	Symmetry
1	28.471	45801.4	472.9	1.324	50.435	0.507
2	57.065	45010.5	243.5	2.2287	49.565	0.466

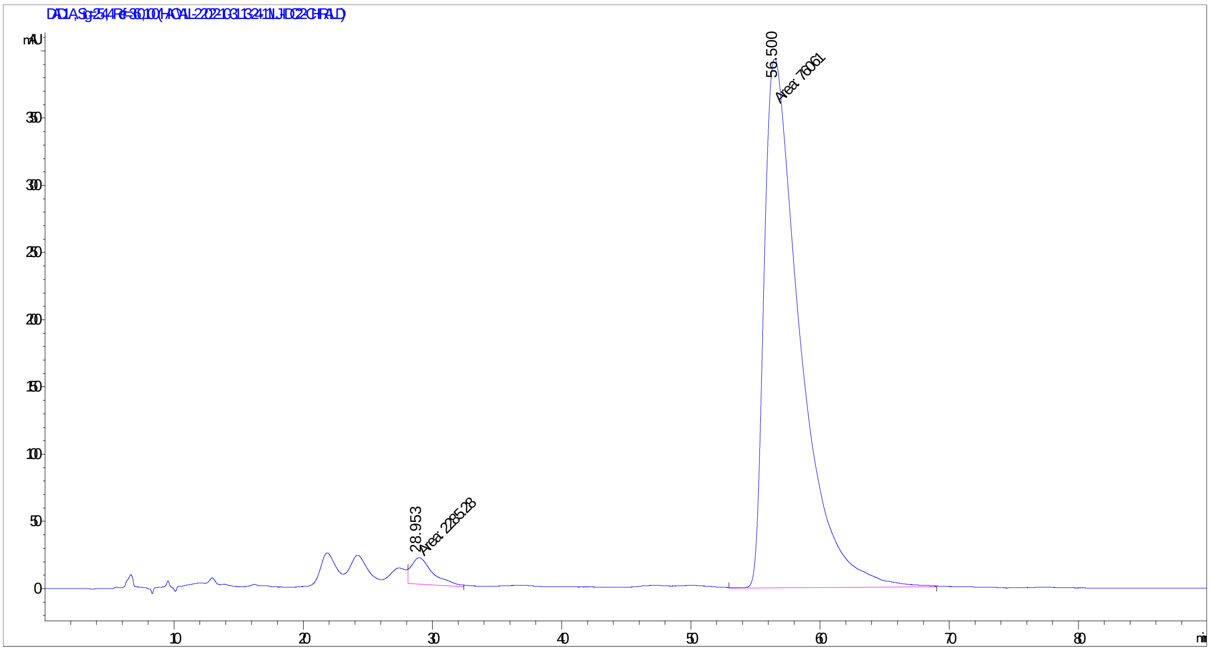

#	Time	Area	Height	Width	Area%	Symmetry
1	28.953	2285.3	19.5	1.9511	2.917	0.512
2	56.5	76061	393.7	3.2198	97.083	0.407

^{1}H NMR Spectrum of 3t (500 MHz, $CDCl_3$)

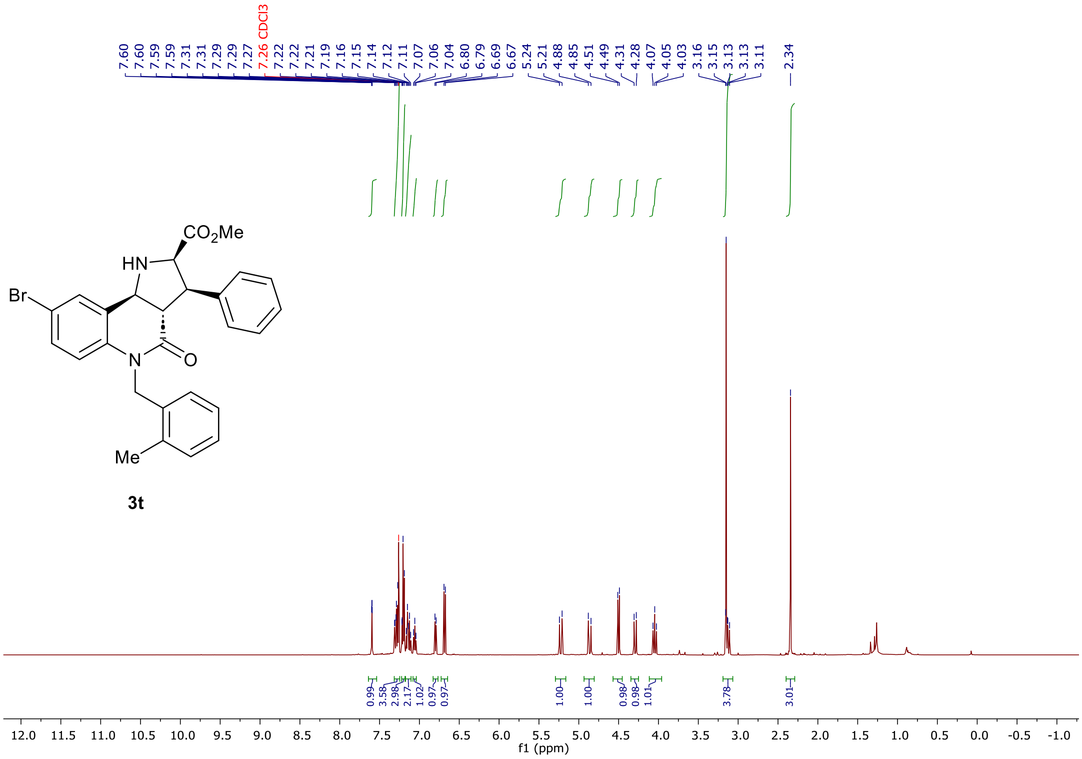

^{13}C NMR Spectrum of 3t (126 MHz, $CDCl_3$)

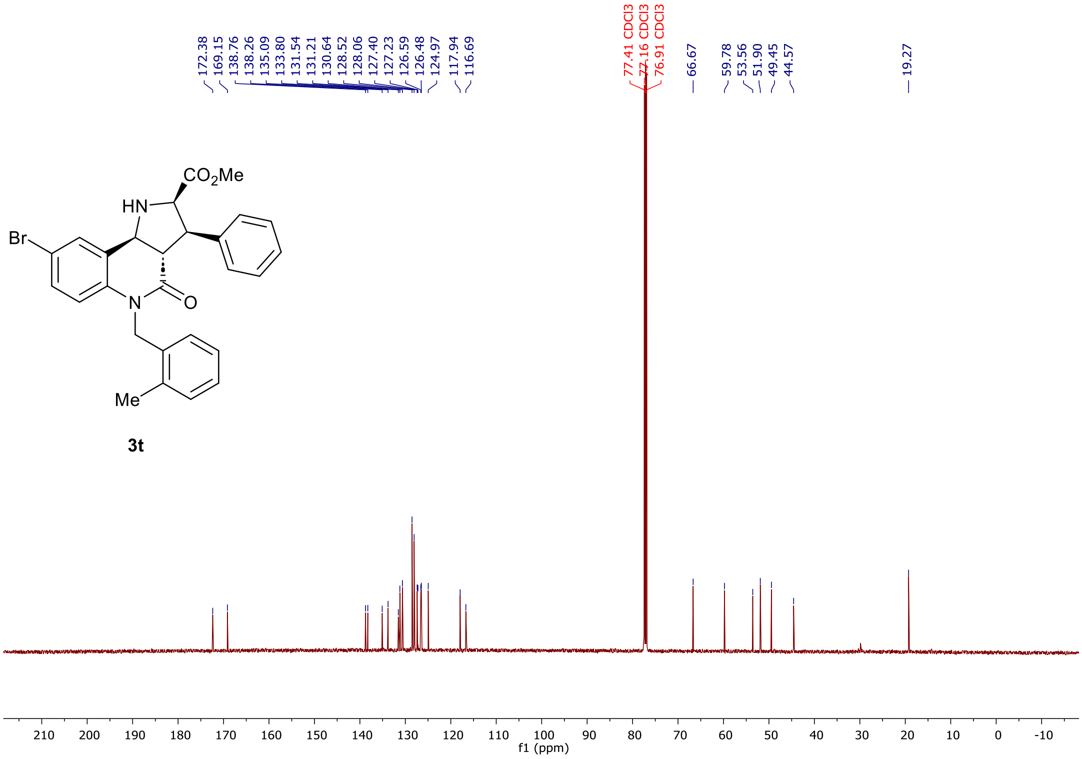

3t HPLC traces: racemate top, enantiomer bottom.

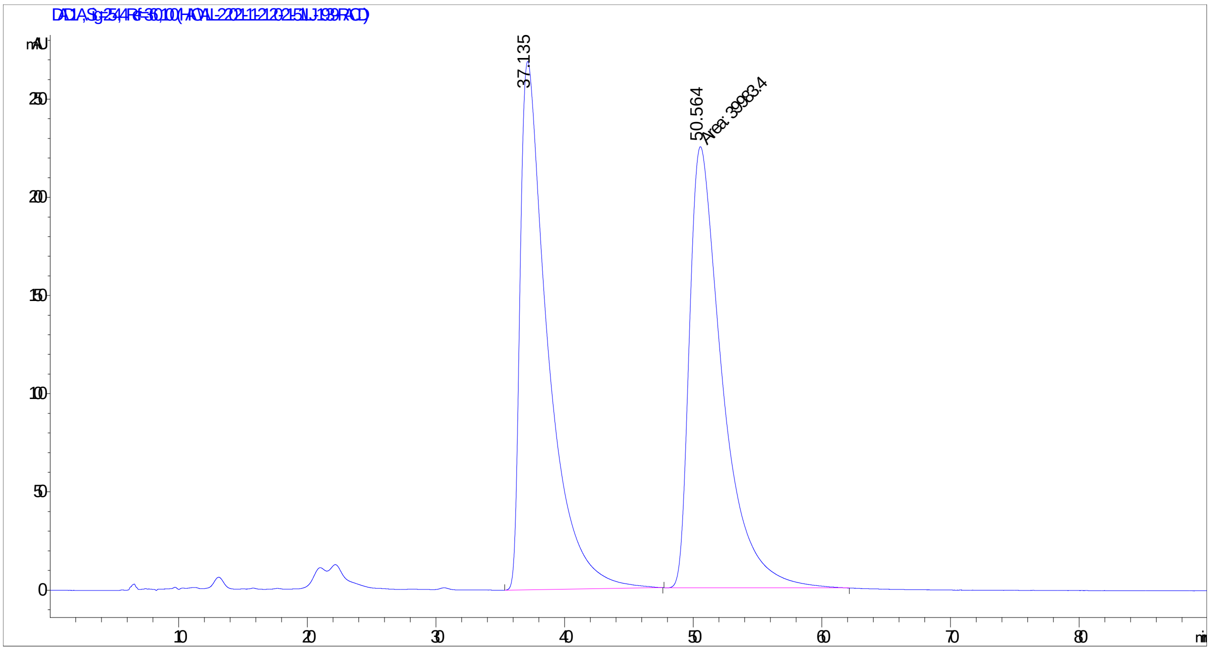

#	Time	Area	Height	Width	Area%	Symmetry
1	37.135	40313.7	269.2	2.0714	50.206	0.342
2	50.564	39983.4	224.7	2.9656	49.794	0.477

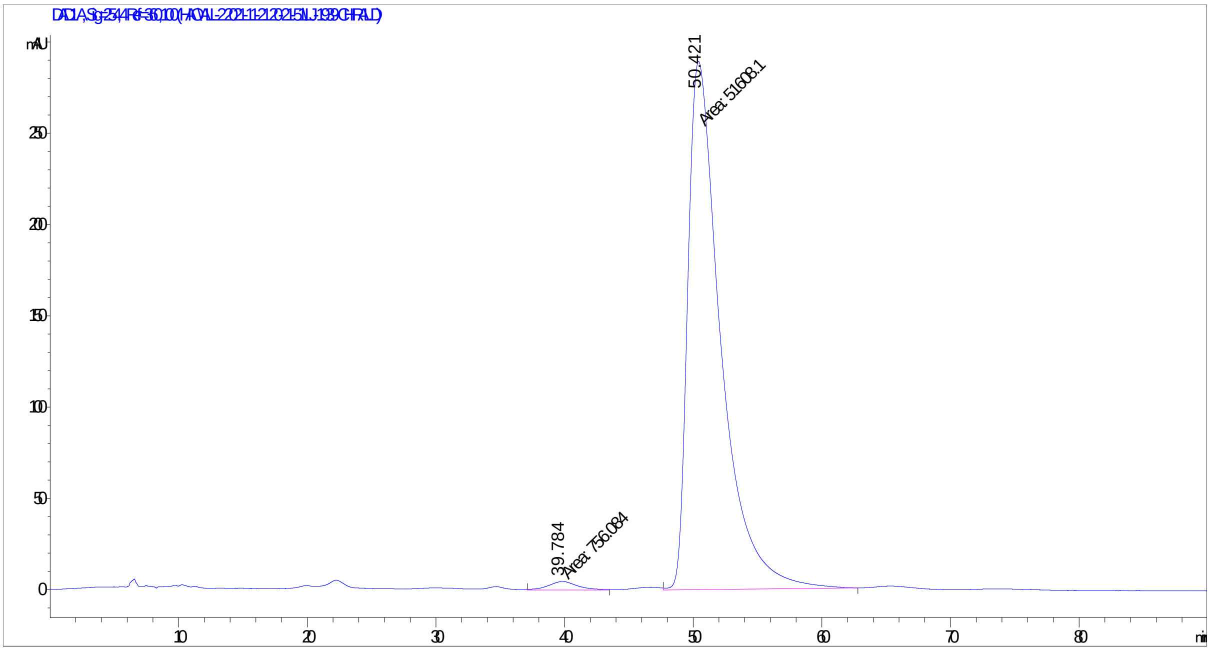

#	Time	Area	Height	Width	Area%	Symmetry
1	39.784	756.1	4.7	2.6611	1.444	0.828
2	50.421	51608.1	289.4	2.9725	98.556	0.461

^{1}H NMR Spectrum of **3u** (500 MHz, $CDCl_3$)

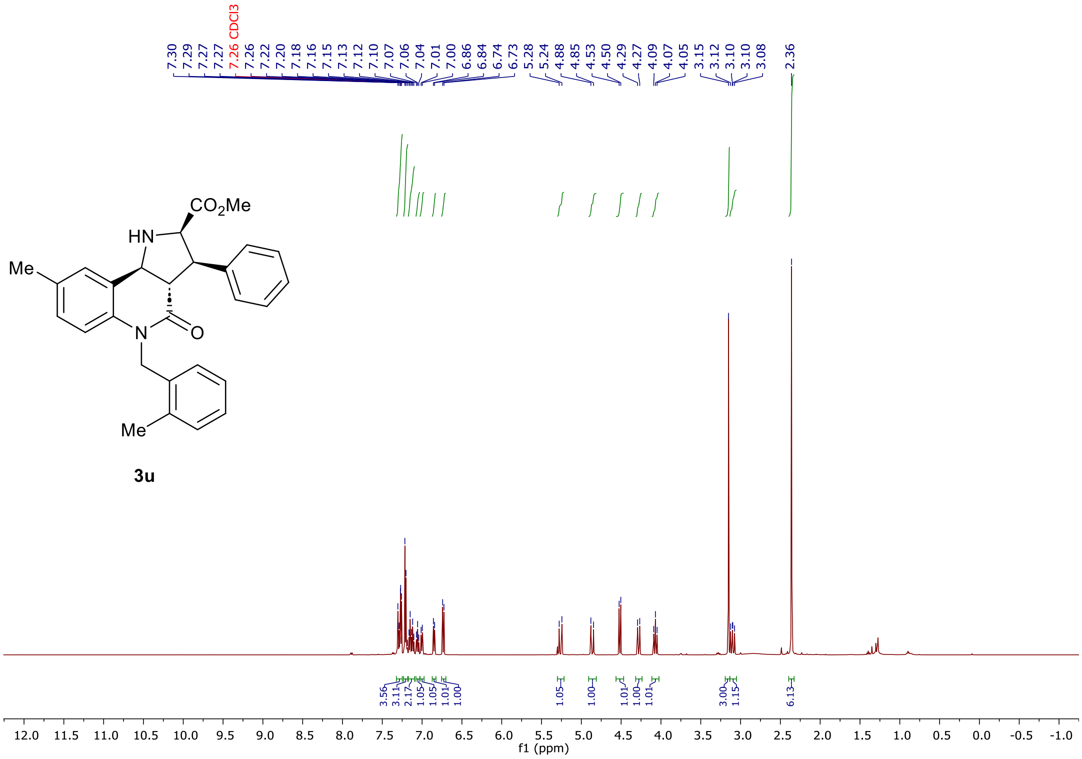

^{13}C NMR Spectrum of **3u** (126 MHz, $CDCl_3$)

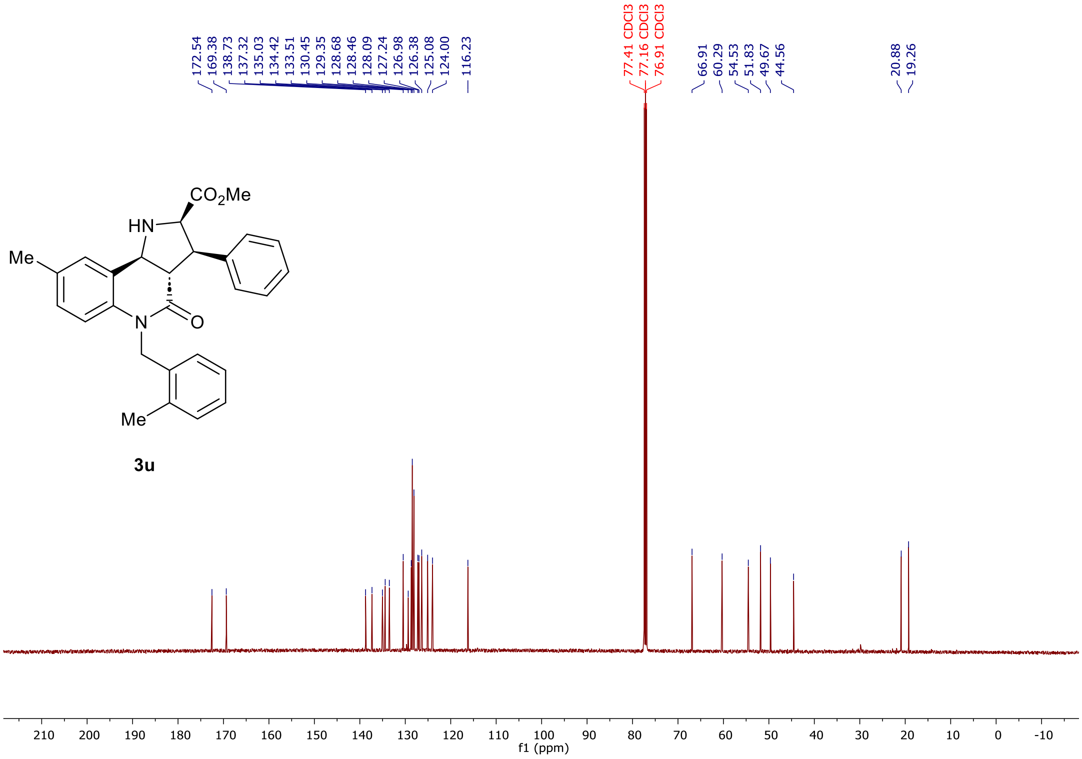

3u HPLC traces: racemate top, enantiomer bottom.

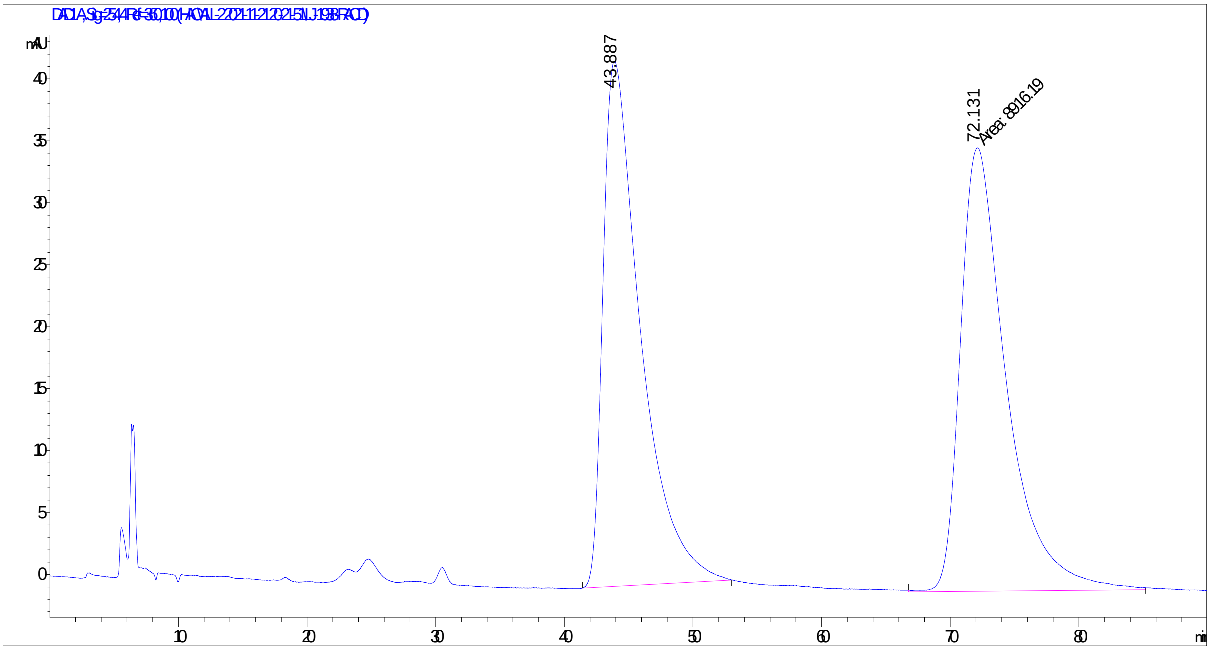

#	Time	Area	Height	Width	Area%	Symmetry
1	43.887	8601.9	42.4	2.3849	49.103	0.409
2	72.131	8916.2	35.8	4.1527	50.897	0.567

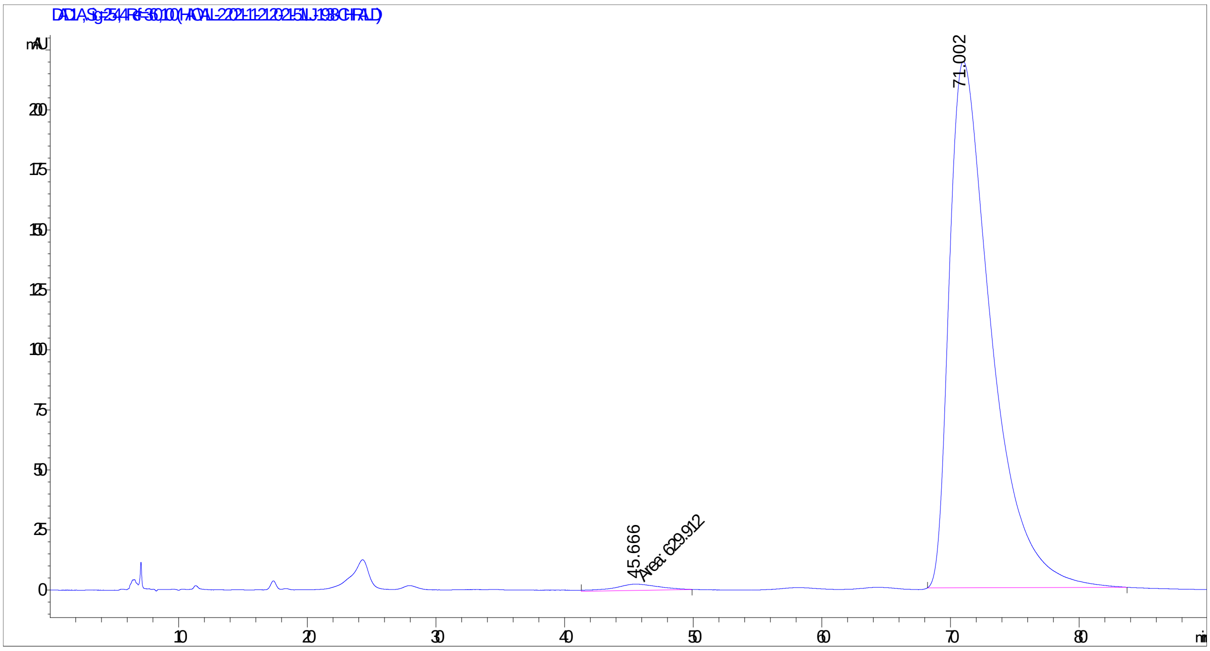

#	Time	Area	Height	Width	Area%	Symmetry
1	45.666	629.9	2.6	4.0049	1.247	1.142
2	71.002	49885.3	219.3	2.9638	98.753	0.461

^{1}H NMR Spectrum of **3v** (500 MHz, $CDCl_3$)

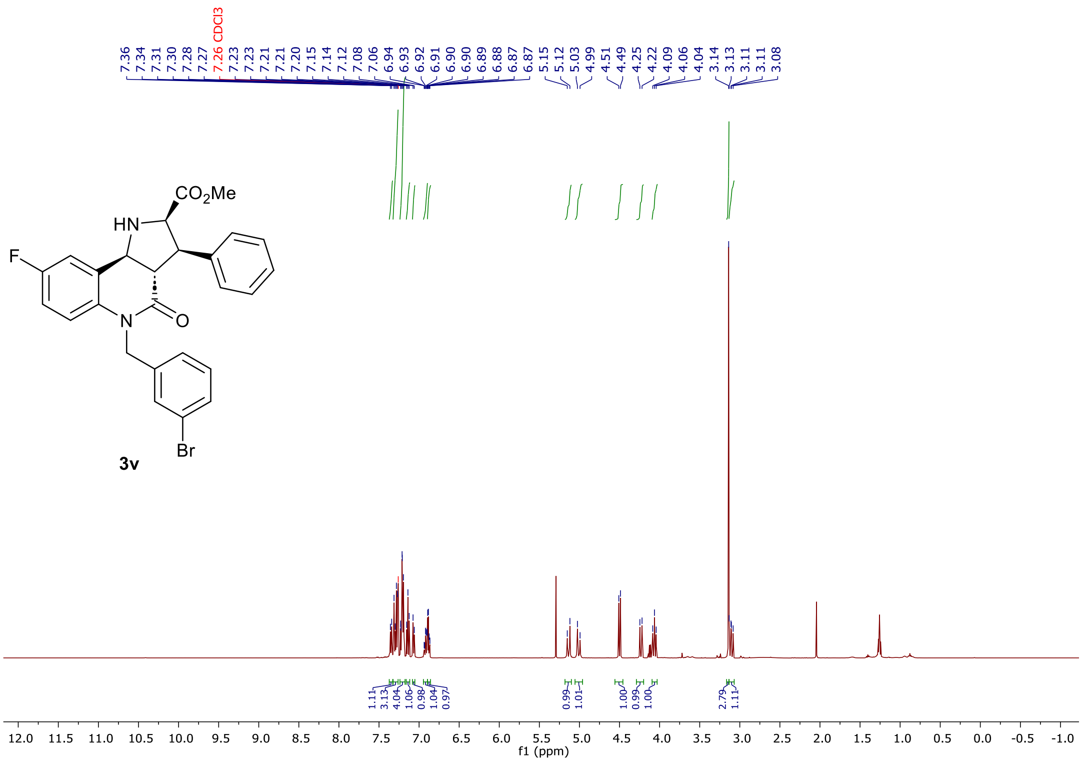

^{13}C NMR Spectrum of **3v** (126 MHz, $CDCl_3$)

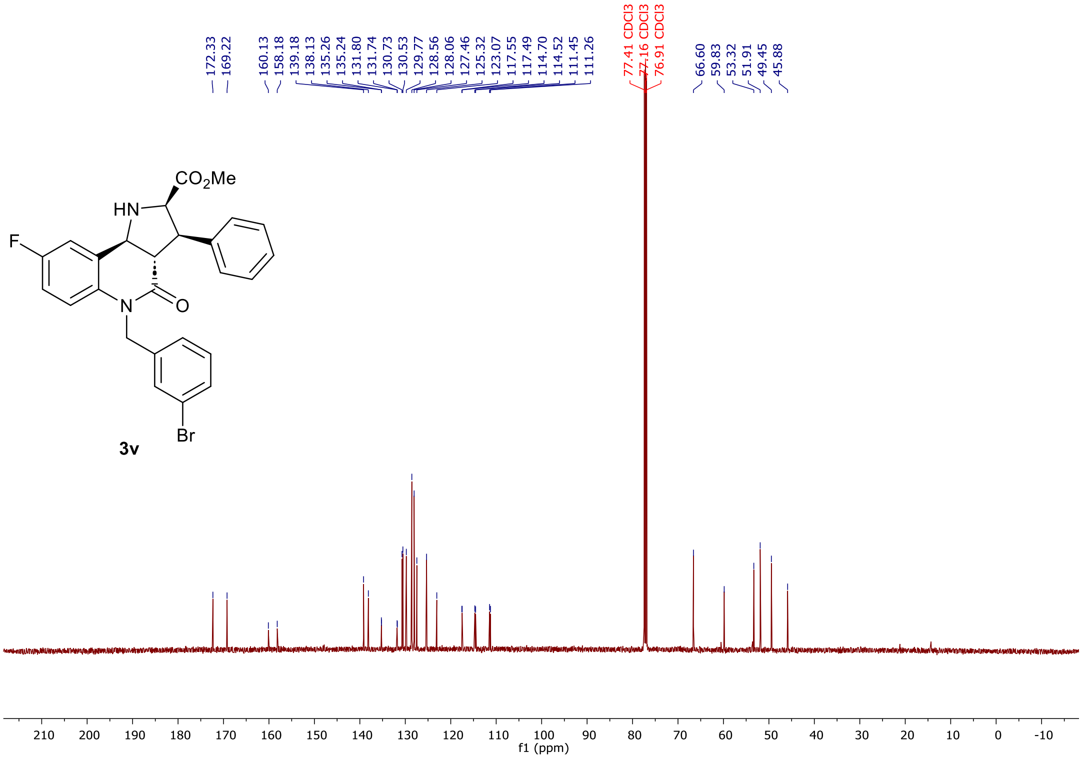

^{19}F NMR Spectrum of **3v** (470 MHz, $CDCl_3$)

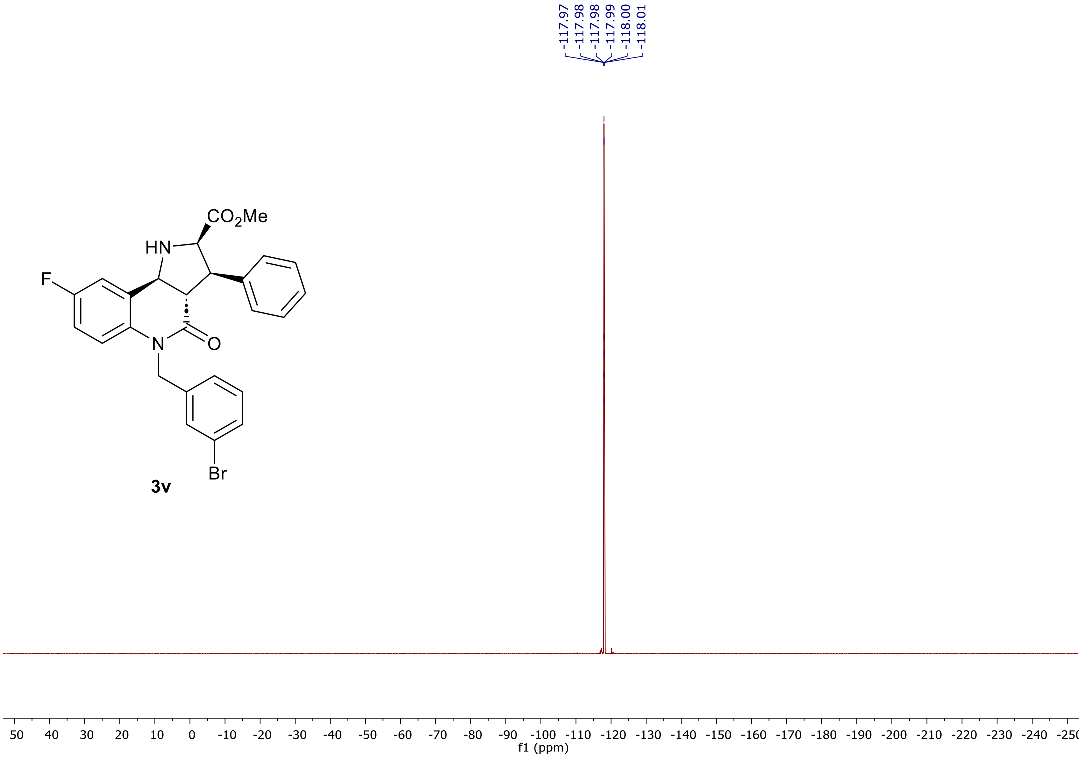

3v HPLC traces: racemate top, enantiomer bottom.

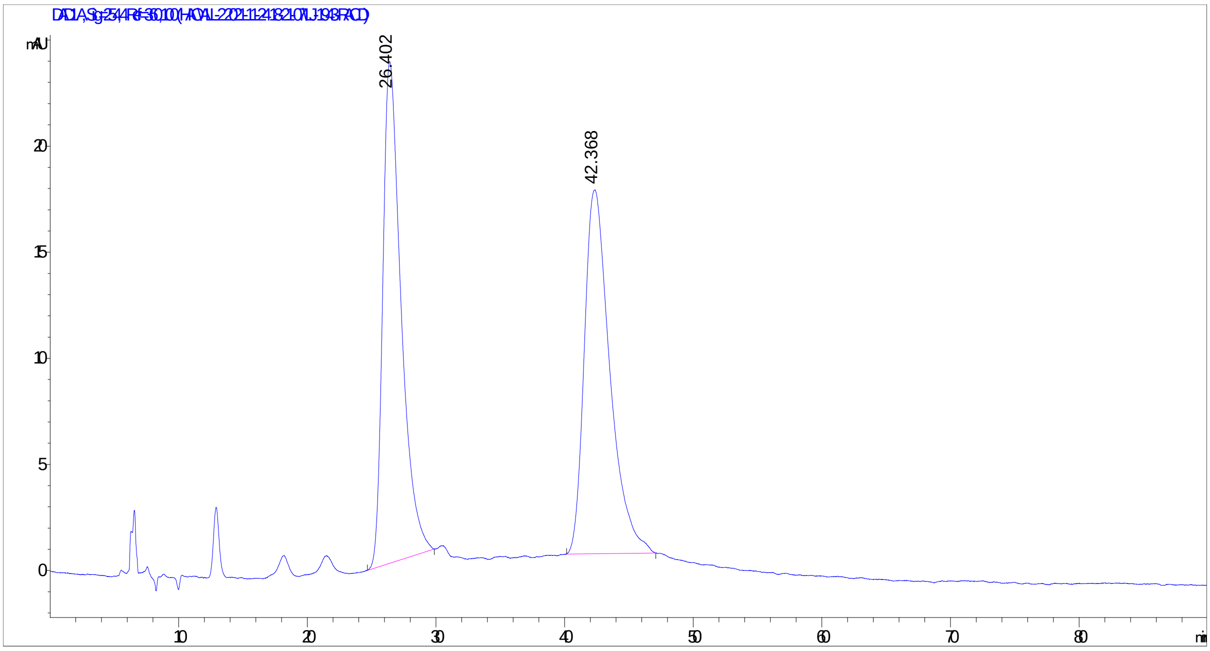

#	Time	Area	Height	Width	Area%	Symmetry
1	26.402	2344.2	23.7	1.2721	49.688	0.552
2	42.368	2373.7	17.1	1.6311	50.312	0.686

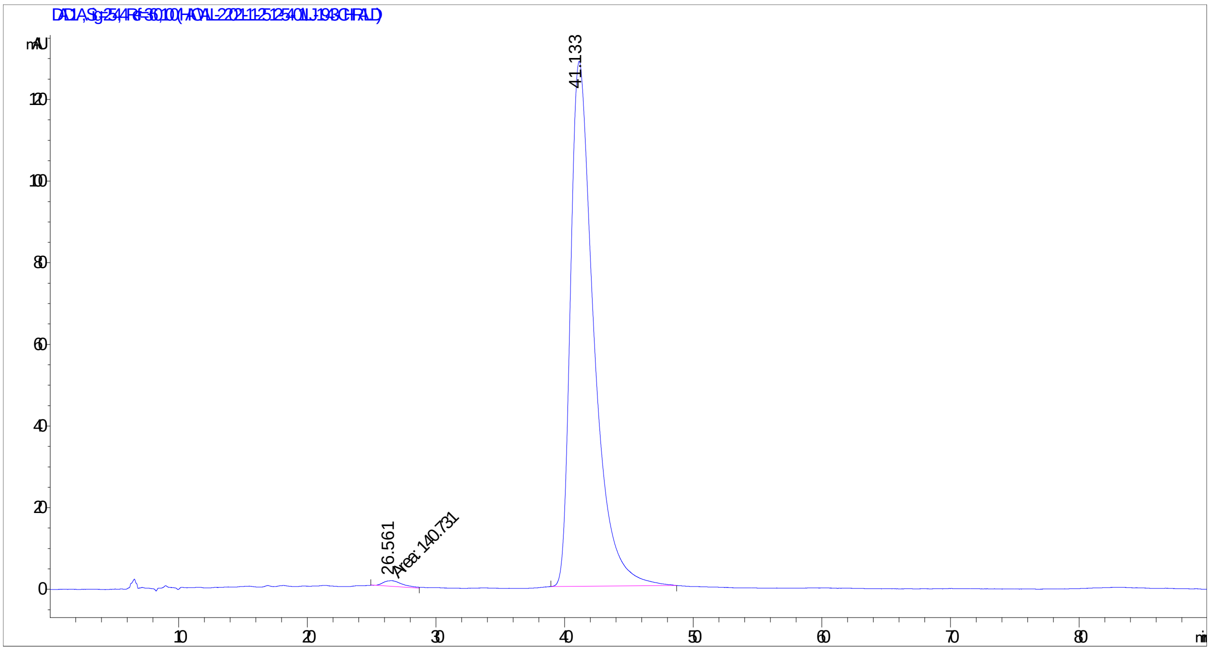

#	Time	Area	Height	Width	Area%	Symmetry
1	26.561	140.7	1.4	1.7062	0.858	0.684
2	41.133	16252	128.6	1.8543	99.142	0.541

^{1}H NMR Spectrum of 3w (500 MHz, $CDCl_3$)

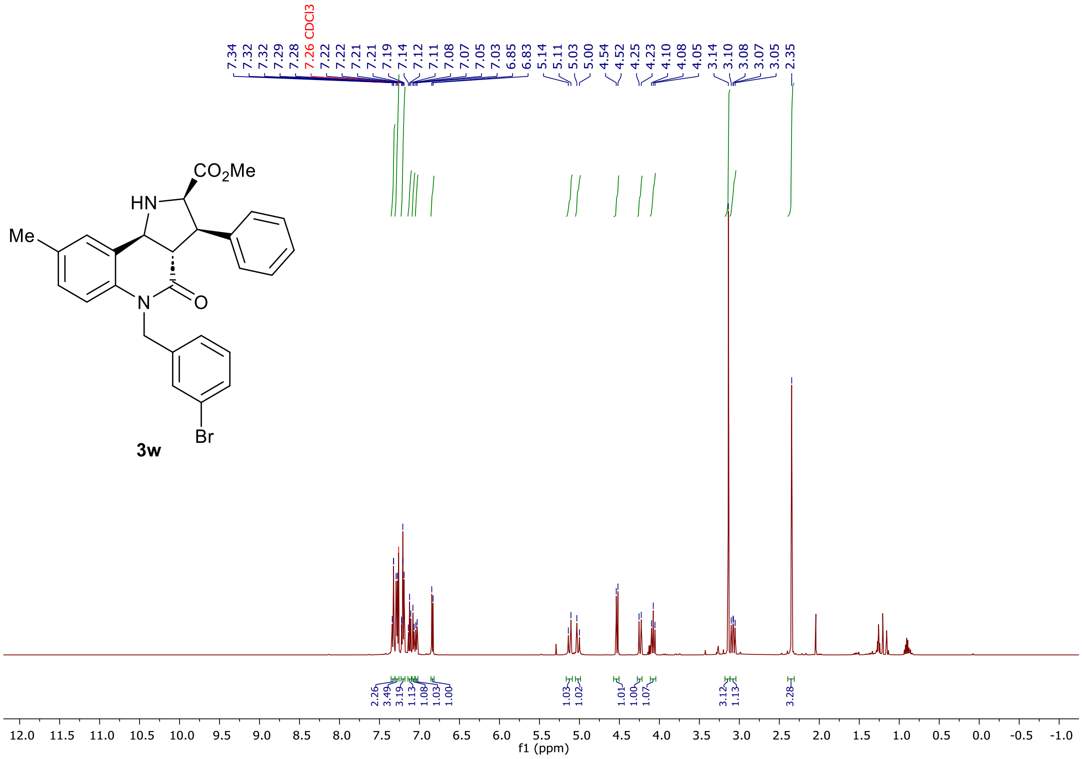

^{13}C NMR Spectrum of 3w (126 MHz, $CDCl_3$)

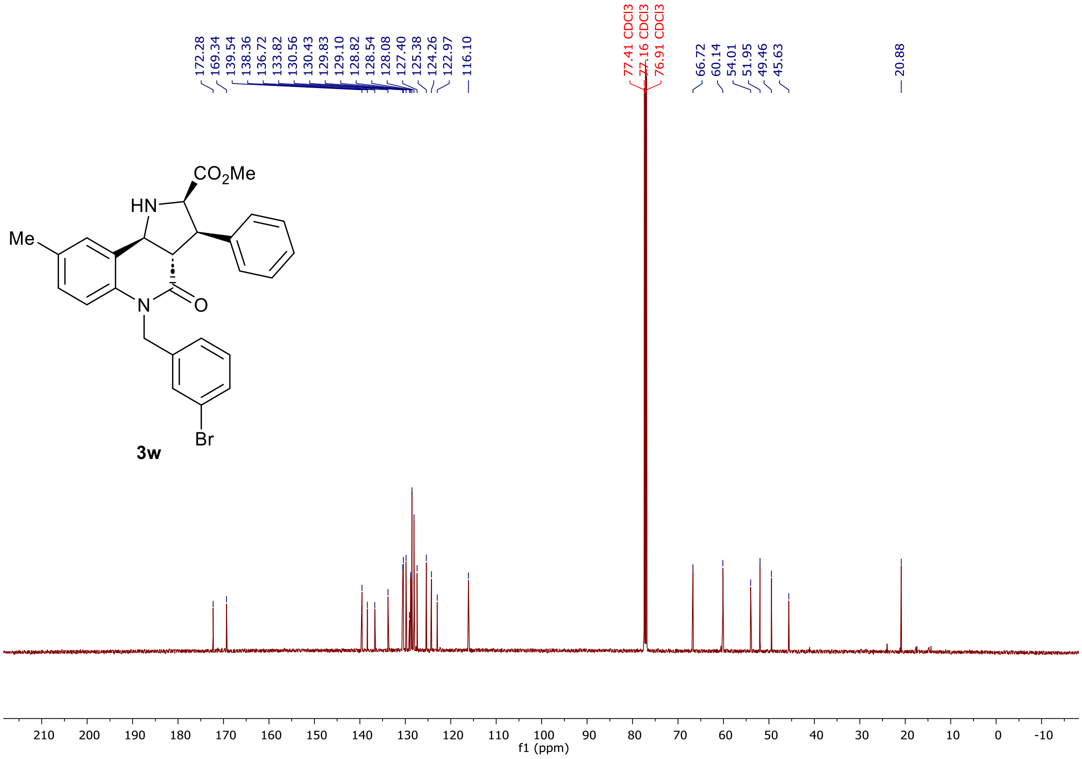

3w HPLC traces: racemate top, enantiomer bottom.

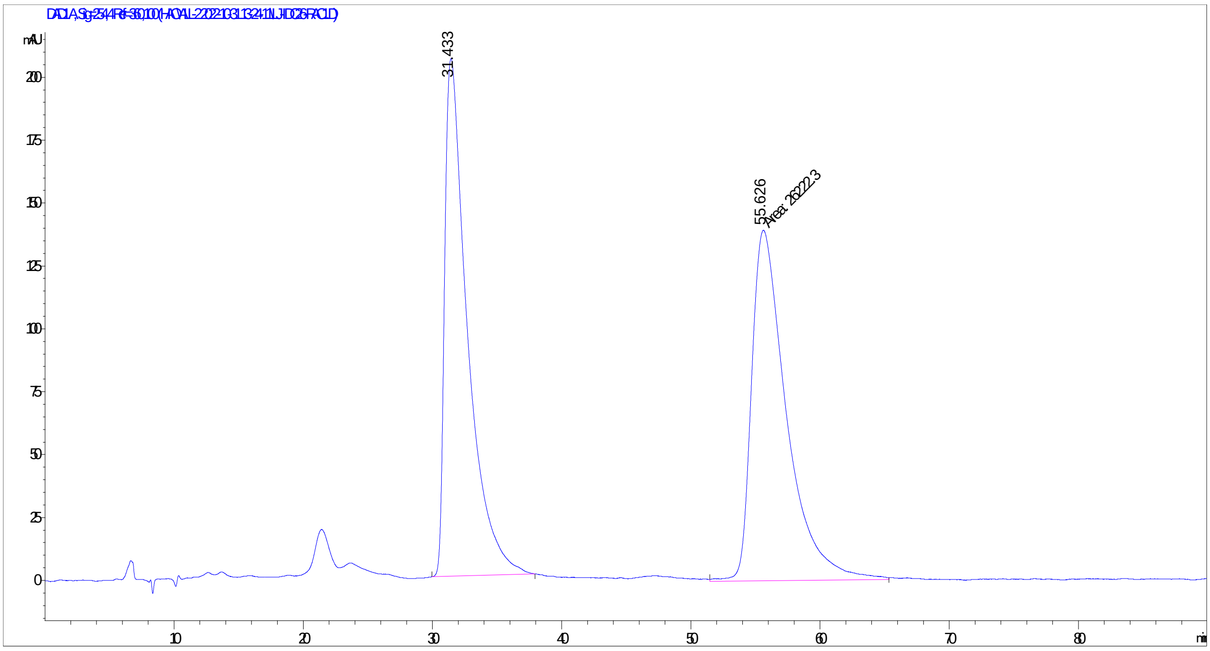

#	Time	Area	Height	Width	Area%	Symmetry
1	31.433	25198	205.8	1.6584	49.004	0.35
2	55.626	26222.3	139.4	3.1355	50.996	0.503

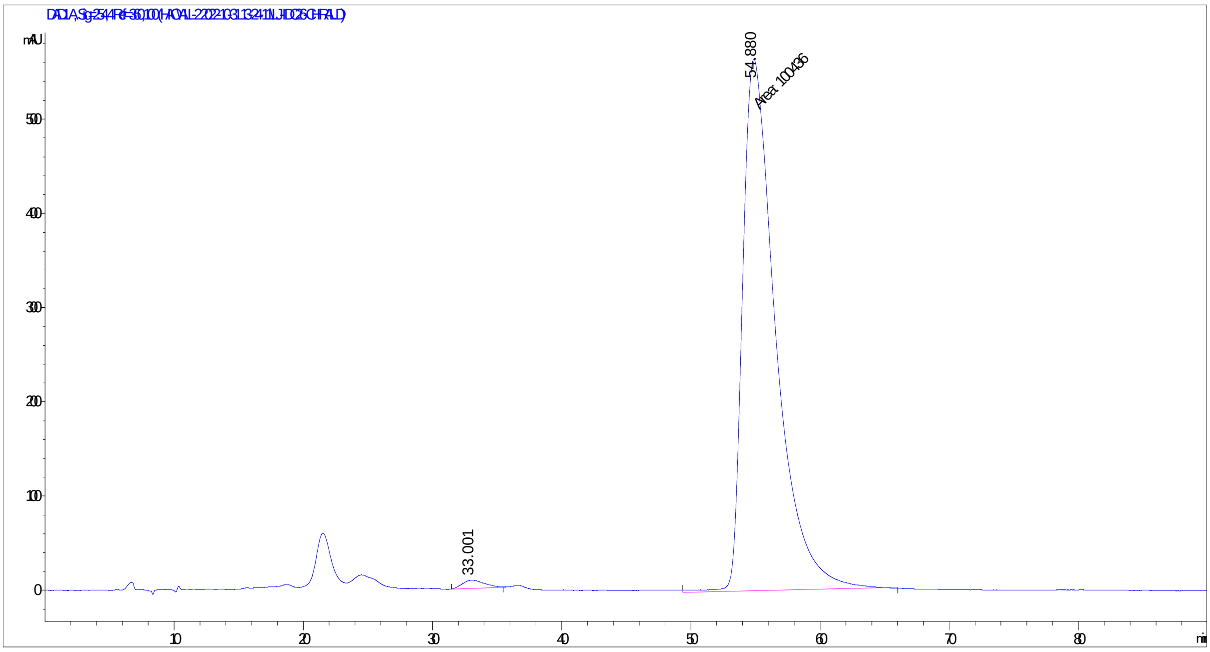

#	Time	Area	Height	Width	Area%	Symmetry
1	33.001	1013.1	8.7	1.3691	0.999	0.695
2	54.88	100436.5	564.3	2.9664	99.001	0.496

^{1}H NMR Spectrum of 3x (500 MHz, CDCl$_3$)

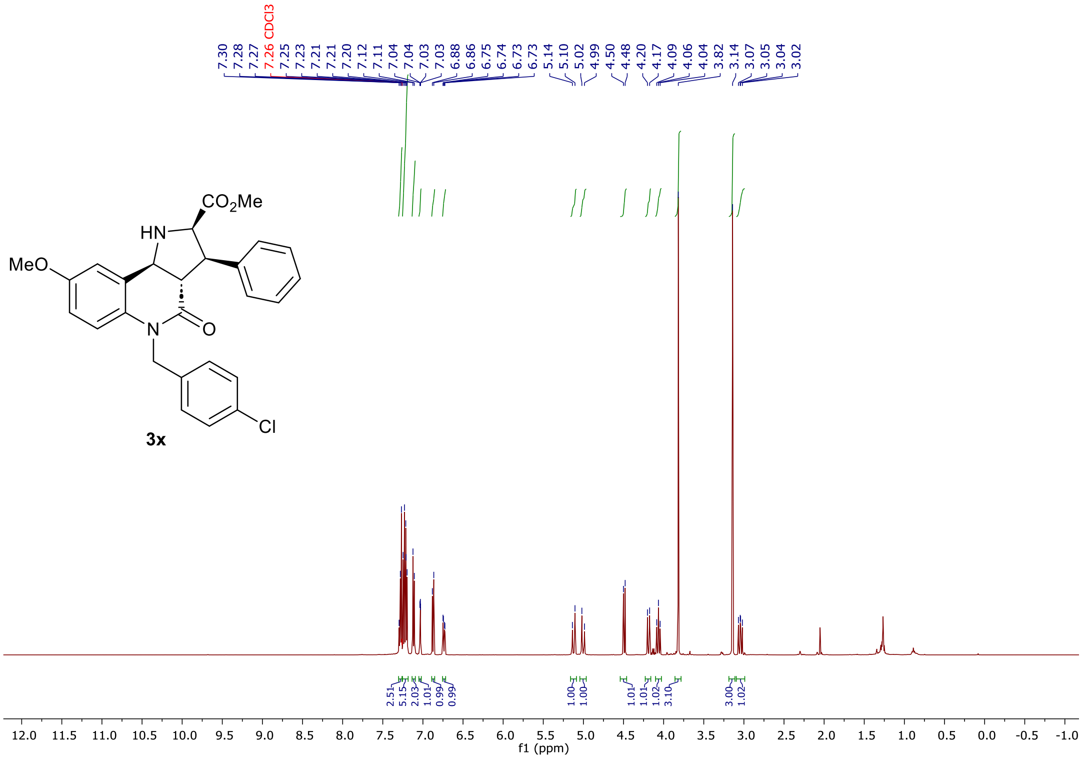

^{13}C NMR Spectrum of 3x (126 MHz, CDCl$_3$)

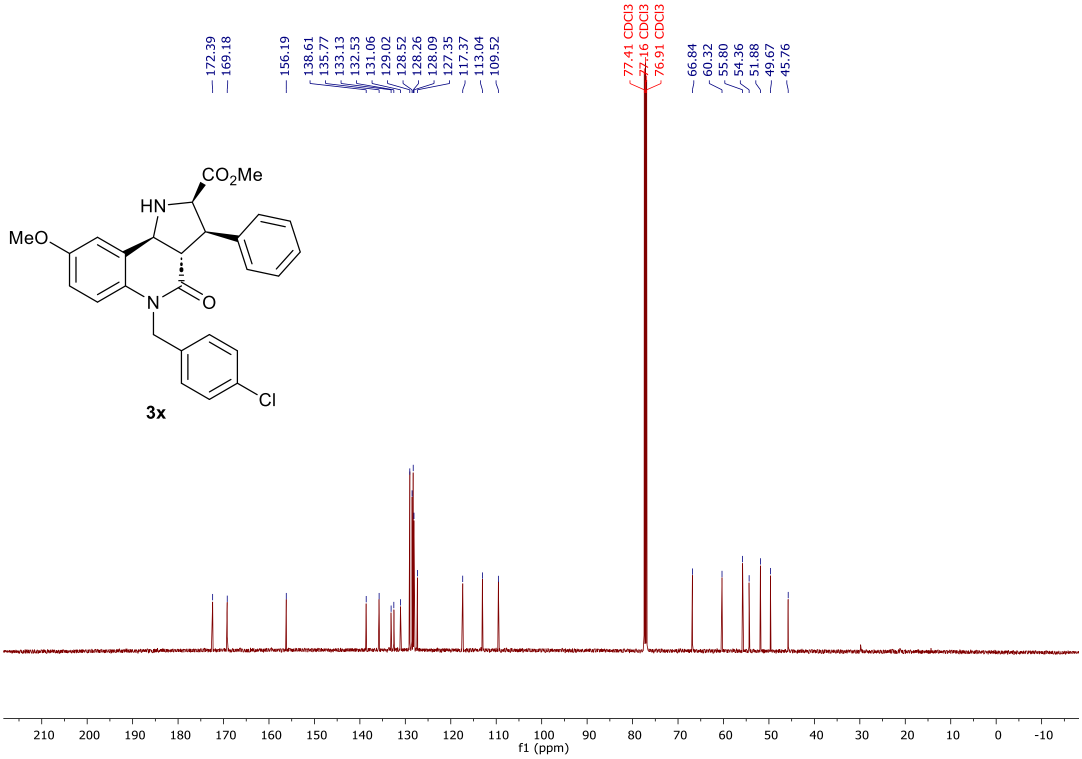

3x HPLC traces: racemate top, enantiomer bottom.

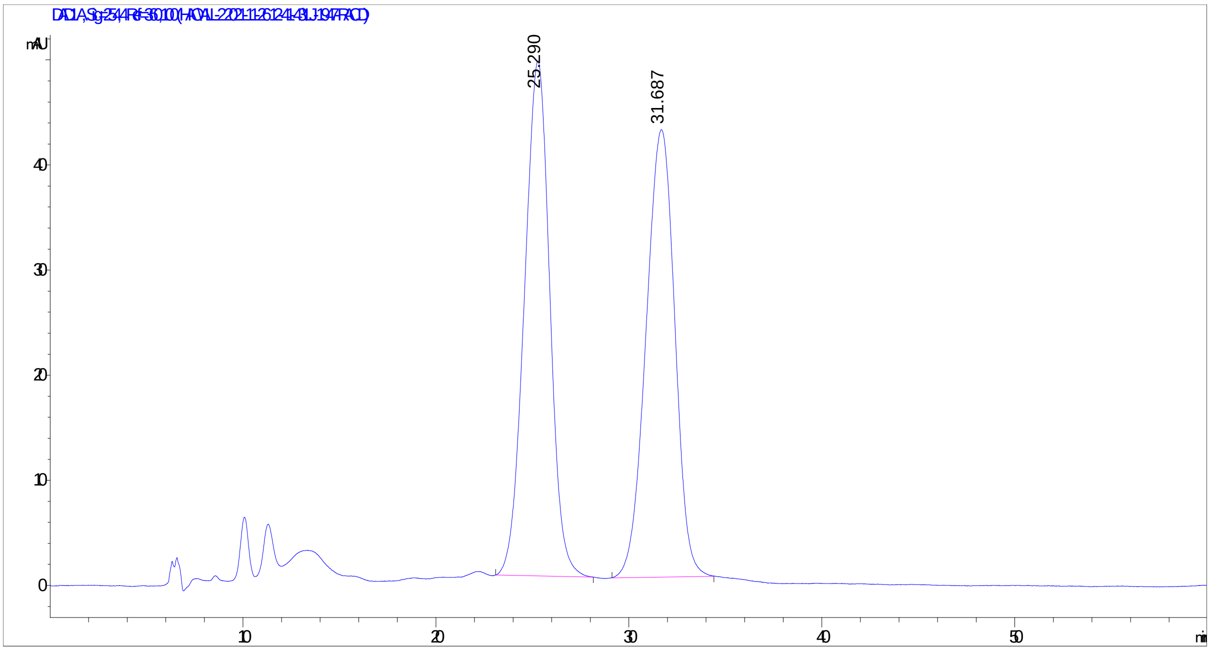

#	Time	Area	Height	Width	Area%	Symmetry
1	25.29	4476.3	49	1.3025	50.383	1.094
2	31.687	4408.2	42.6	1.3841	49.617	1.107

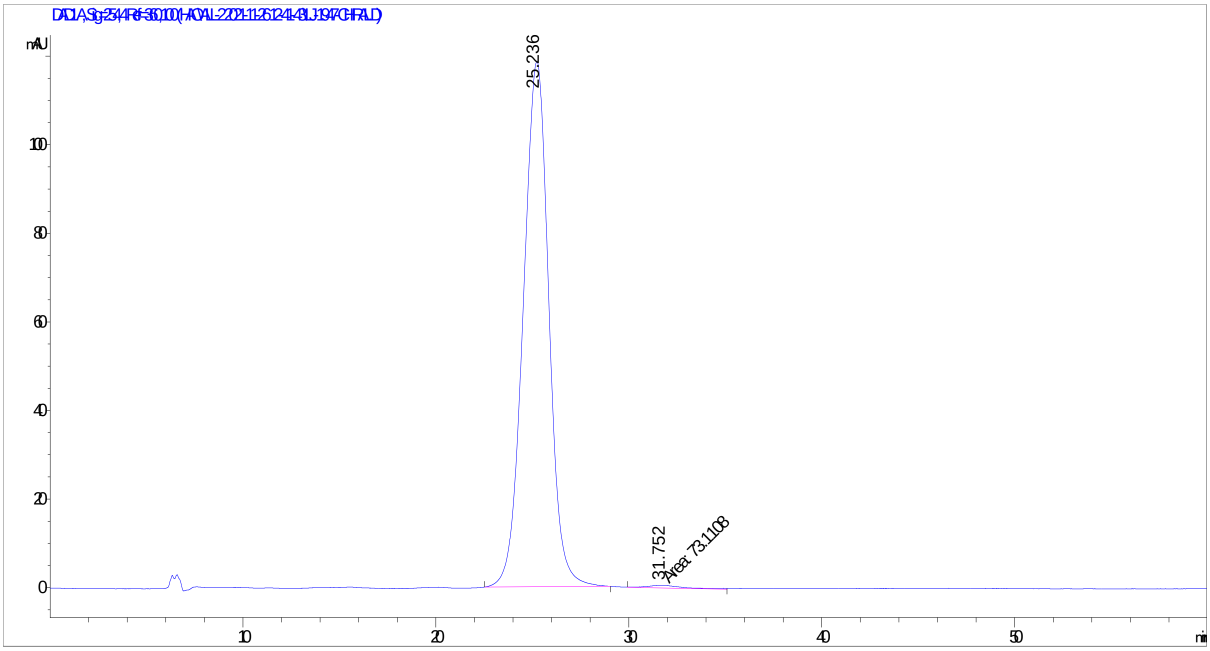

#	Time	Area	Height	Width	Area%	Symmetry
1	25.236	10879.5	118.6	1.388	99.332	1.075
2	31.752	73.1	5.8E-1	2.1165	0.668	0.708

^{1}H NMR Spectrum of **3y** (700 MHz, $CDCl_3$)

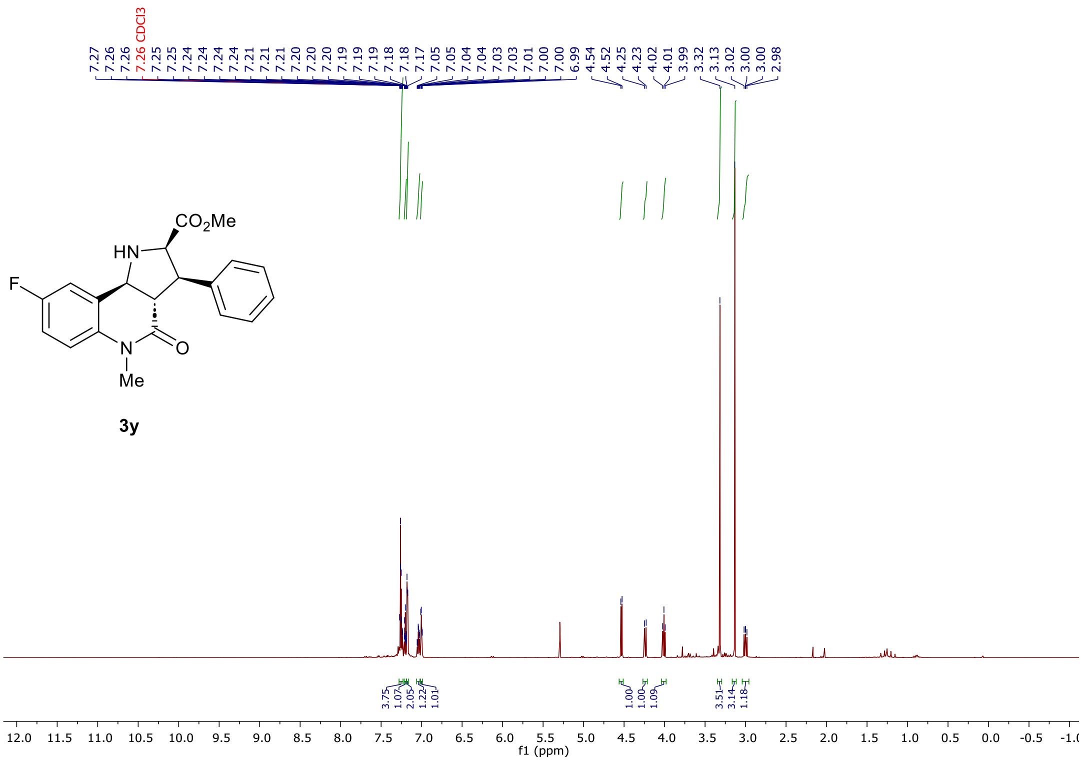

^{13}C NMR Spectrum of **3y** (176 MHz, $CDCl_3$)

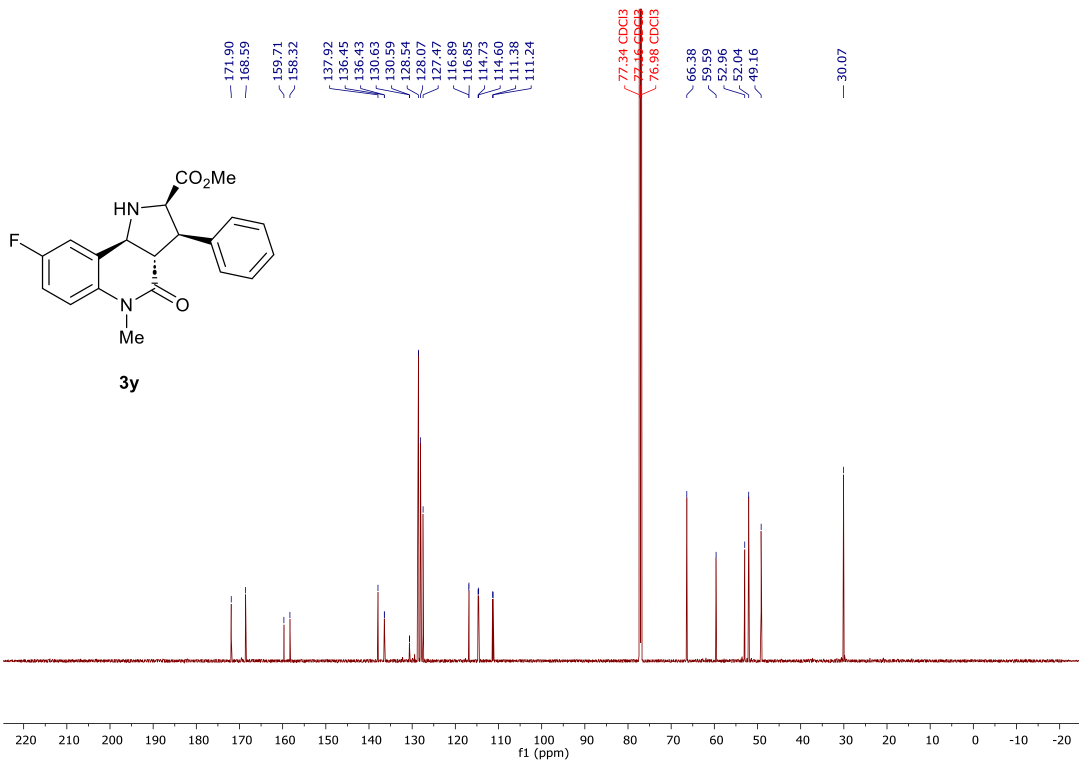

^{19}F NMR Spectrum of **3y** (470 MHz, $CDCl_3$)

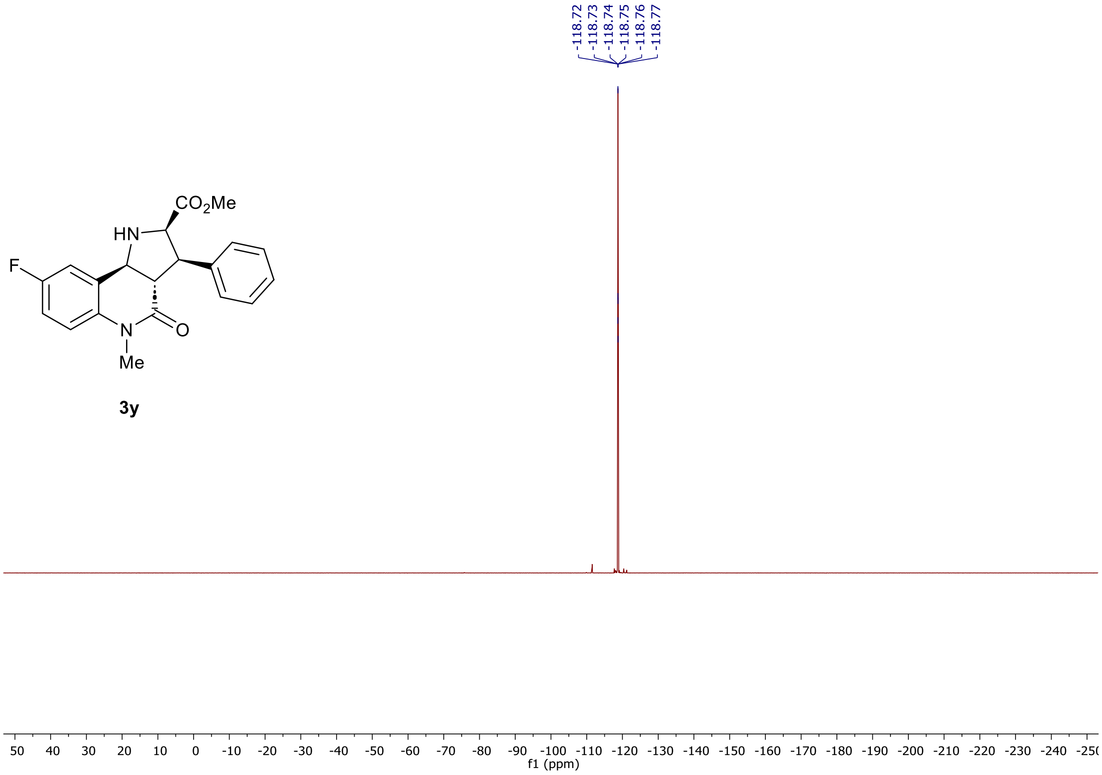

3y HPLC traces: racemate top, enantiomer bottom.

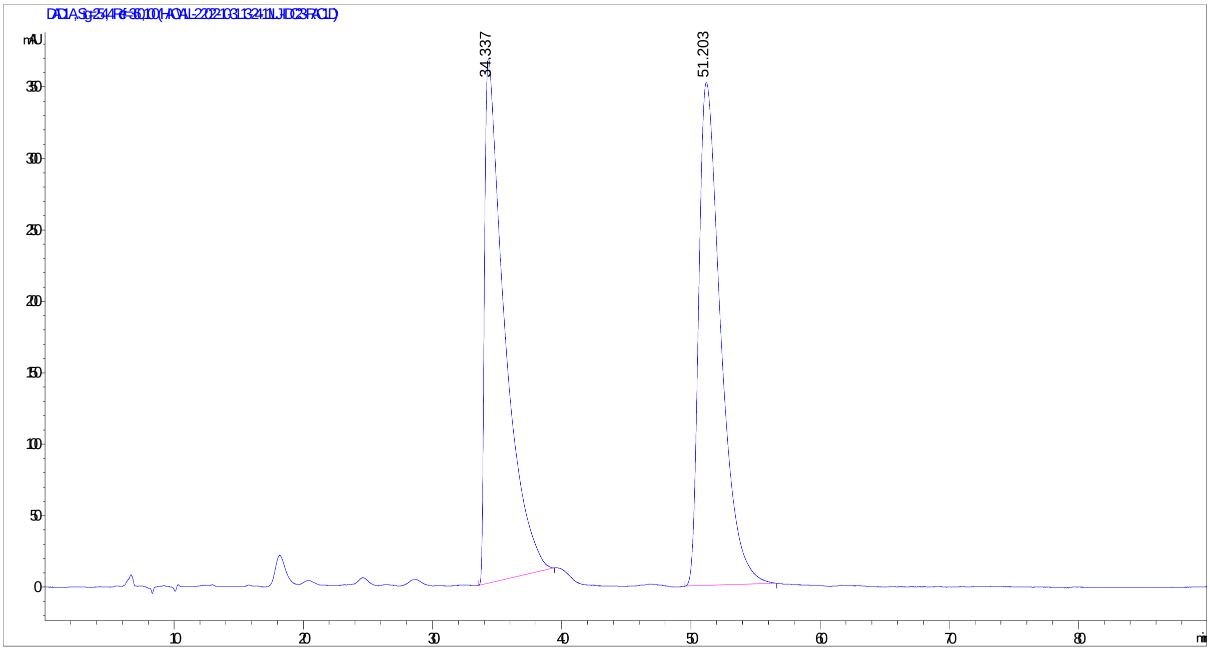

#	Time	Area	Height	Width	Area%	Symmetry
1	34.337	39680.9	367.1	1.429	48.801	0.224
2	51.203	41631	352.1	1.6295	51.199	0.491

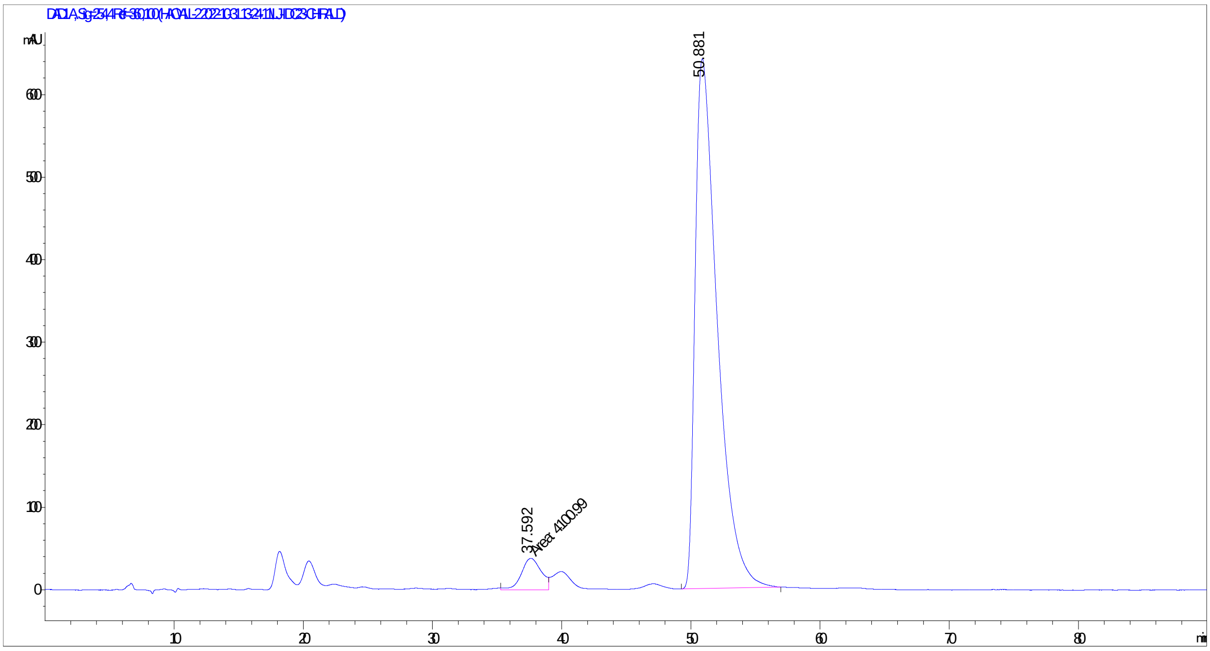

#	Time	Area	Height	Width	Area%	Symmetry
1	37.592	4101	38.3	1.7859	5.084	0.862
2	50.881	76556.4	642.3	1.5846	94.916	0.429

^{1}H NMR Spectrum of **3z** (500 MHz, $CDCl_3$)

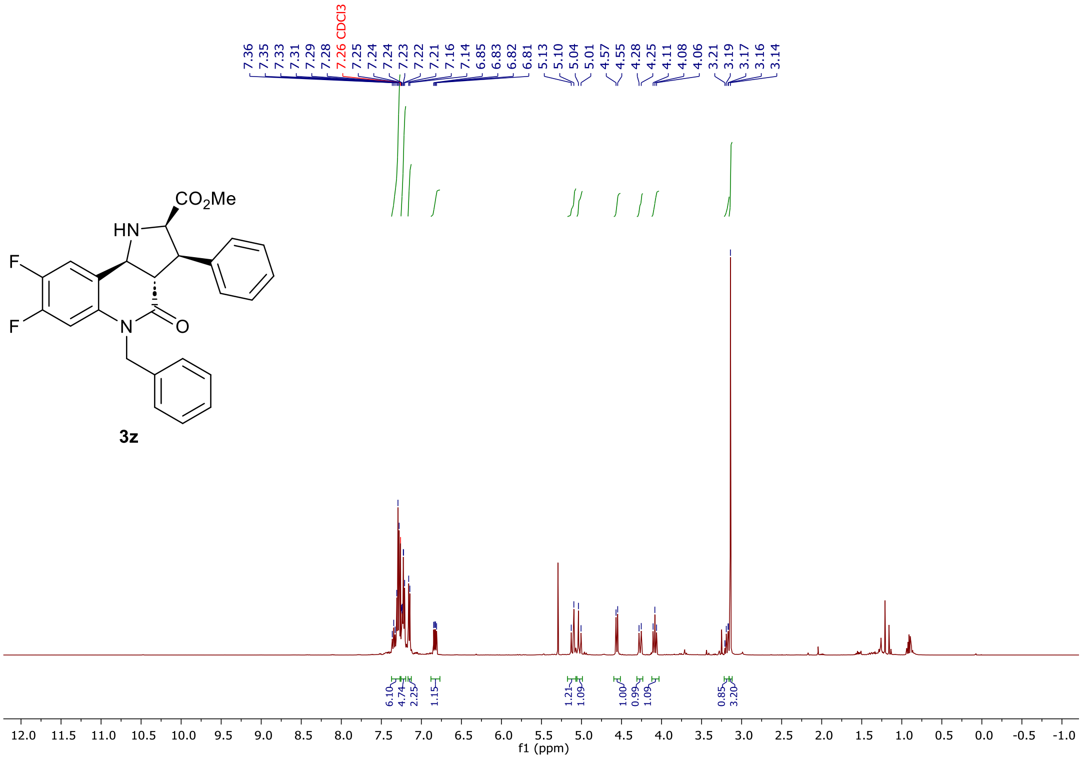

^{13}C NMR Spectrum of **3z** (176 MHz, $CDCl_3$)

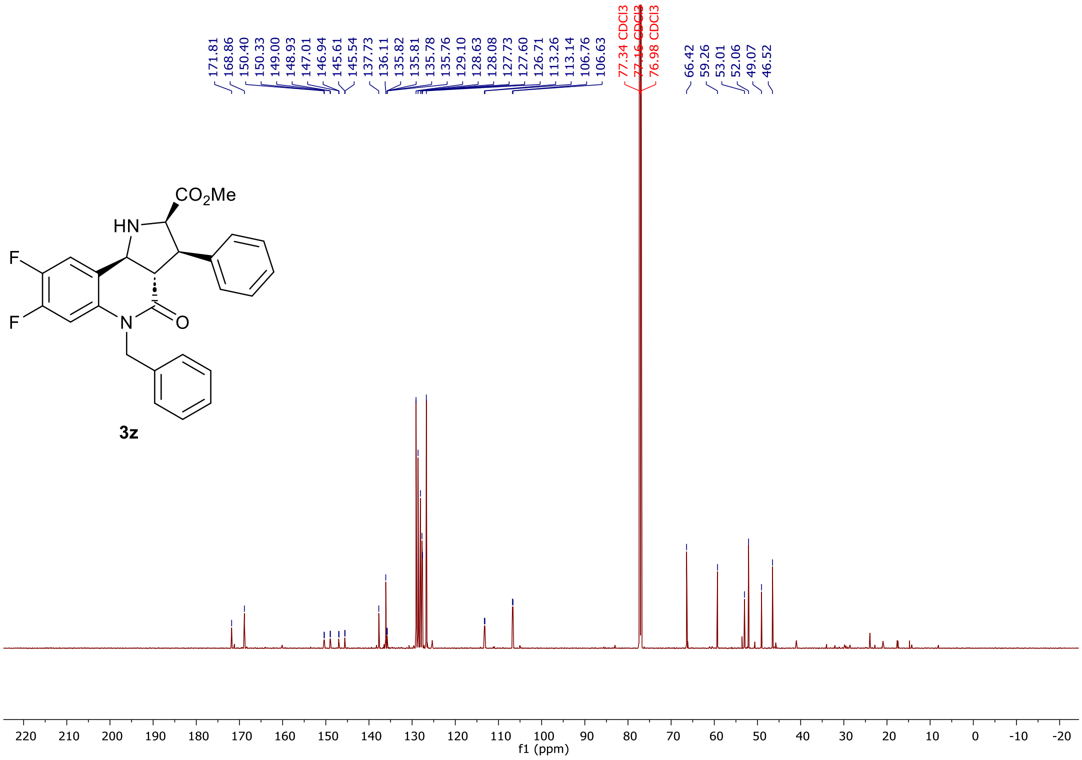

^{19}F NMR Spectrum of **3z** (470 MHz, $CDCl_3$)

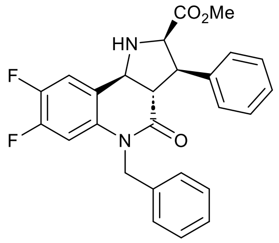

3z

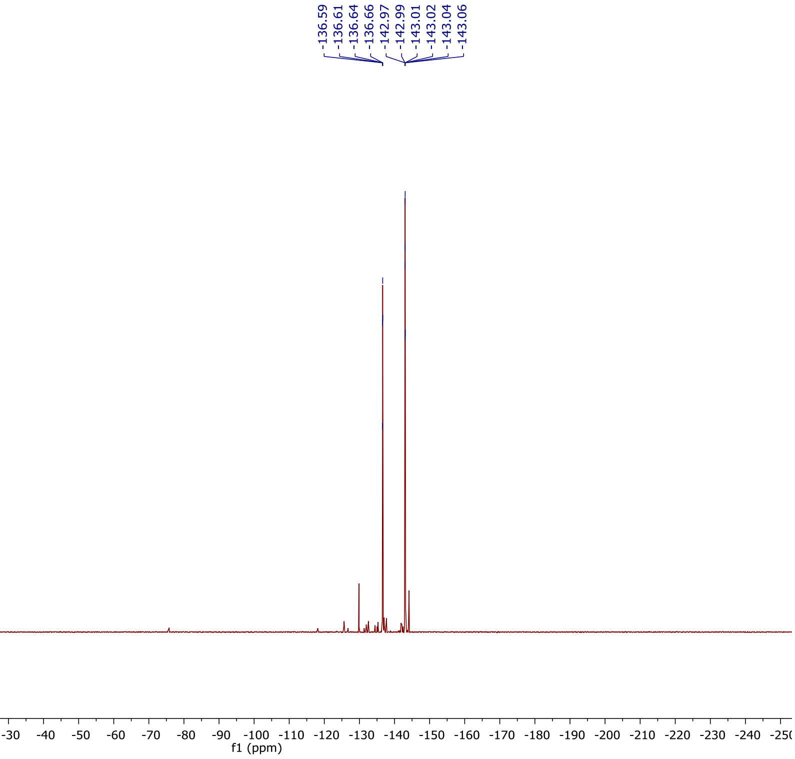

3z HPLC traces: racemate top, enantiomer bottom.

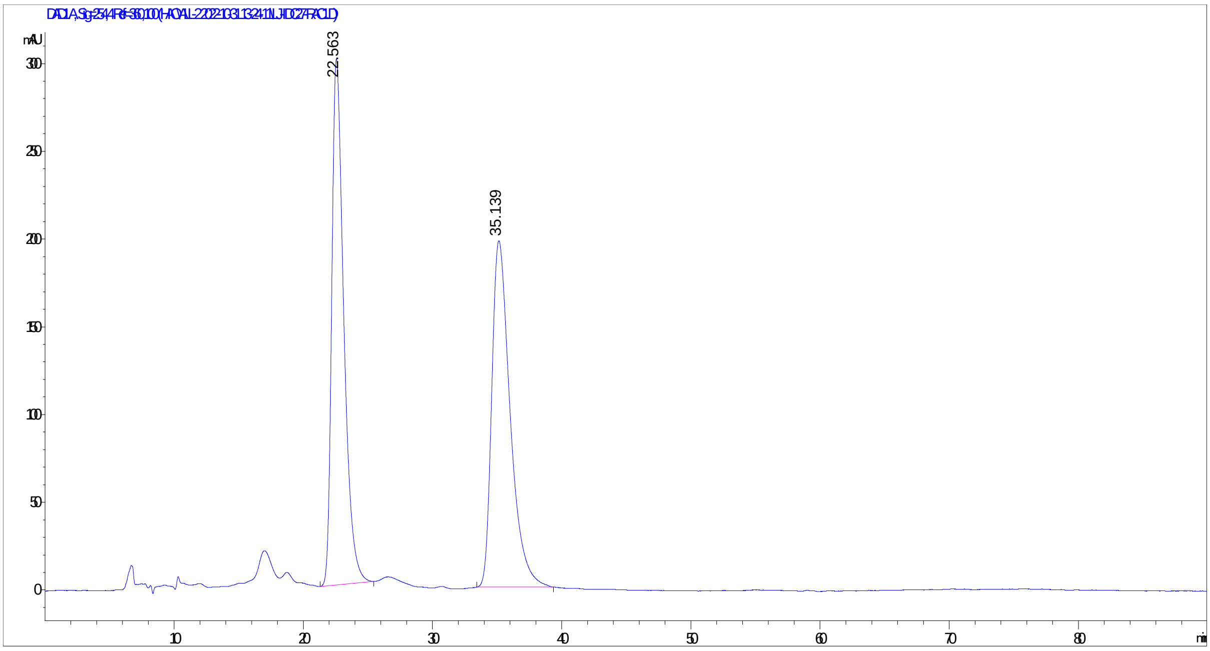

#	Time	Area	Height	Width	Area%	Symmetry
1	22.563	19386.2	300.1	0.9437	49.818	0.588
2	35.139	19527.6	197.4	1.31	50.182	0.587

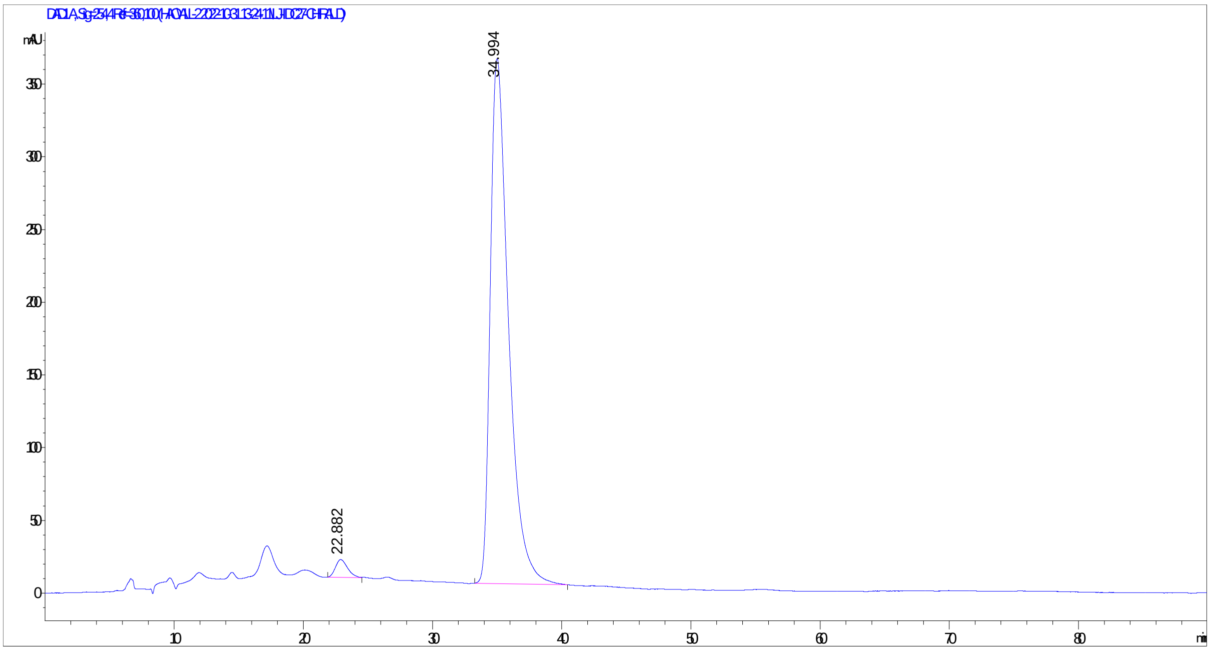

#	Time	Area	Height	Width	Area%	Symmetry
1	22.882	784.4	12.2	0.7673	2.170	0.71
2	34.994	35367.2	361.1	1.3707	97.830	0.547

^{1}H NMR Spectrum of 3aa (500 MHz, $CDCl_3$)

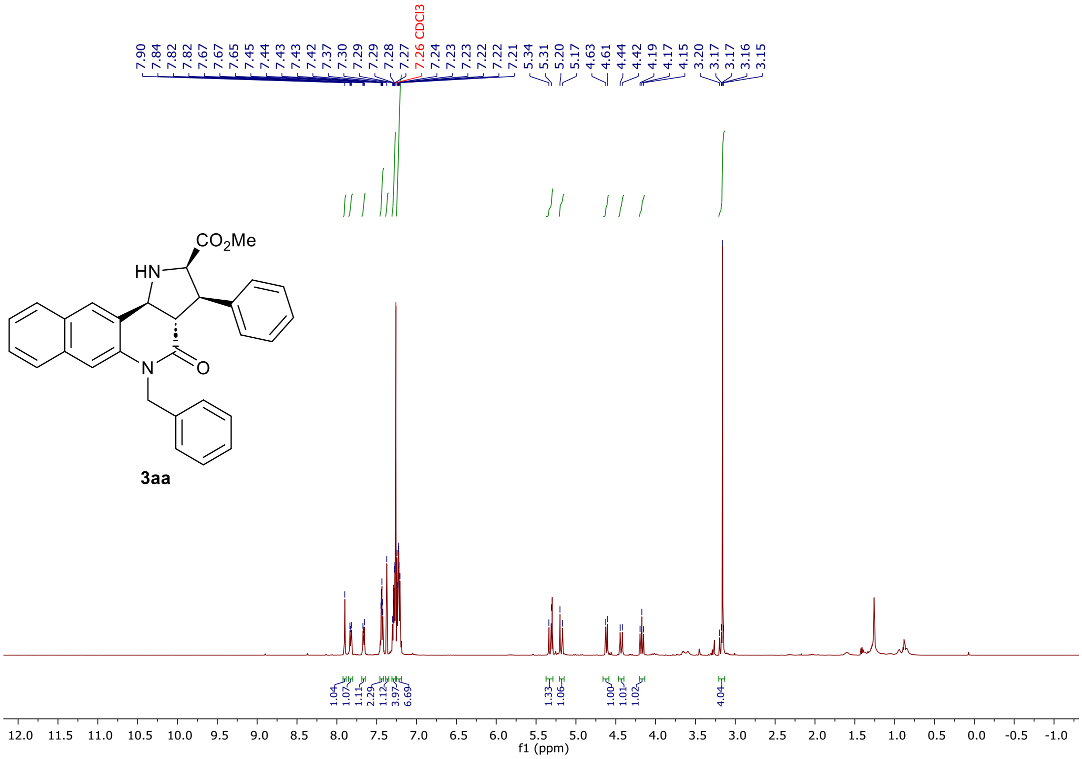

^{13}C NMR Spectrum of 3aa (176 MHz, $CDCl_3$)

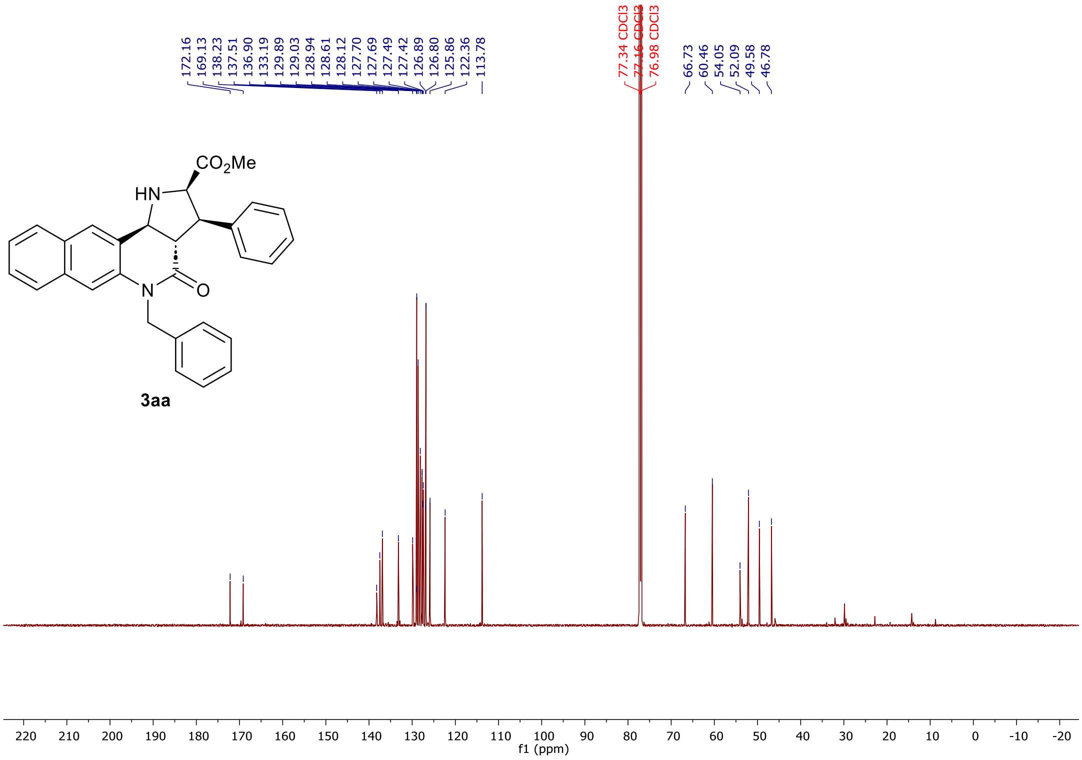

3aa HPLC traces: racemate top, enantiomer bottom.

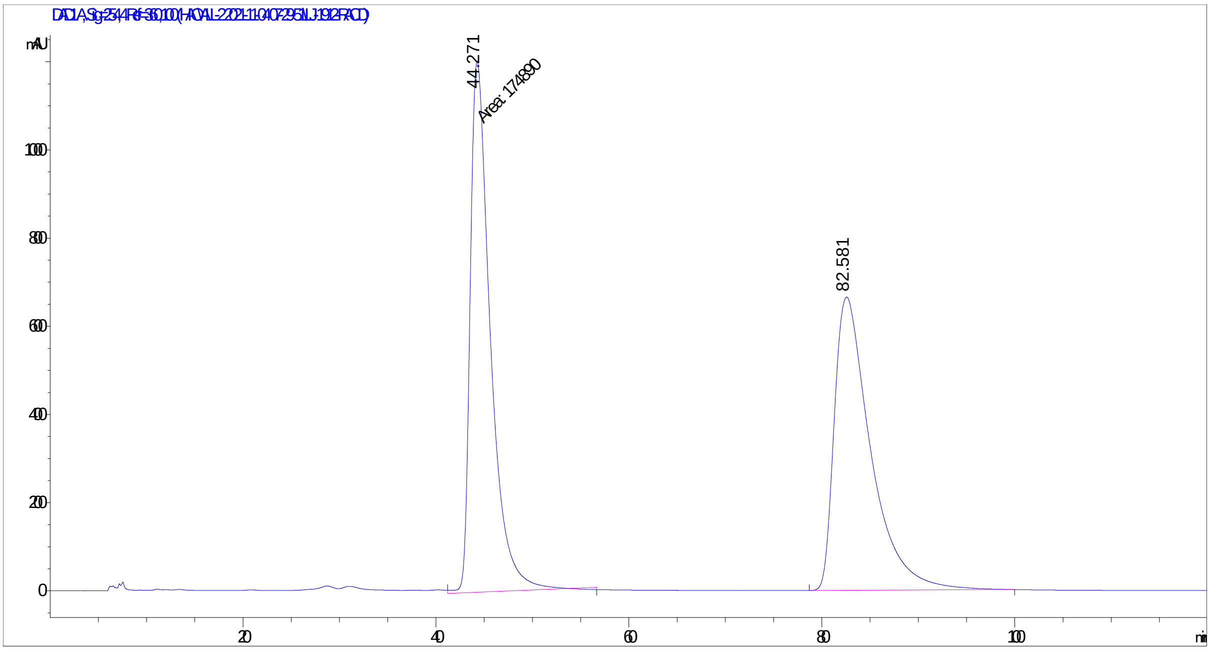

#	Time	Area	Height	Width	Area%	Symmetry
1	44.271	174890	1204	2.4209	50.107	0.507
2	82.581	174139.7	665.1	3.1804	49.893	0.5

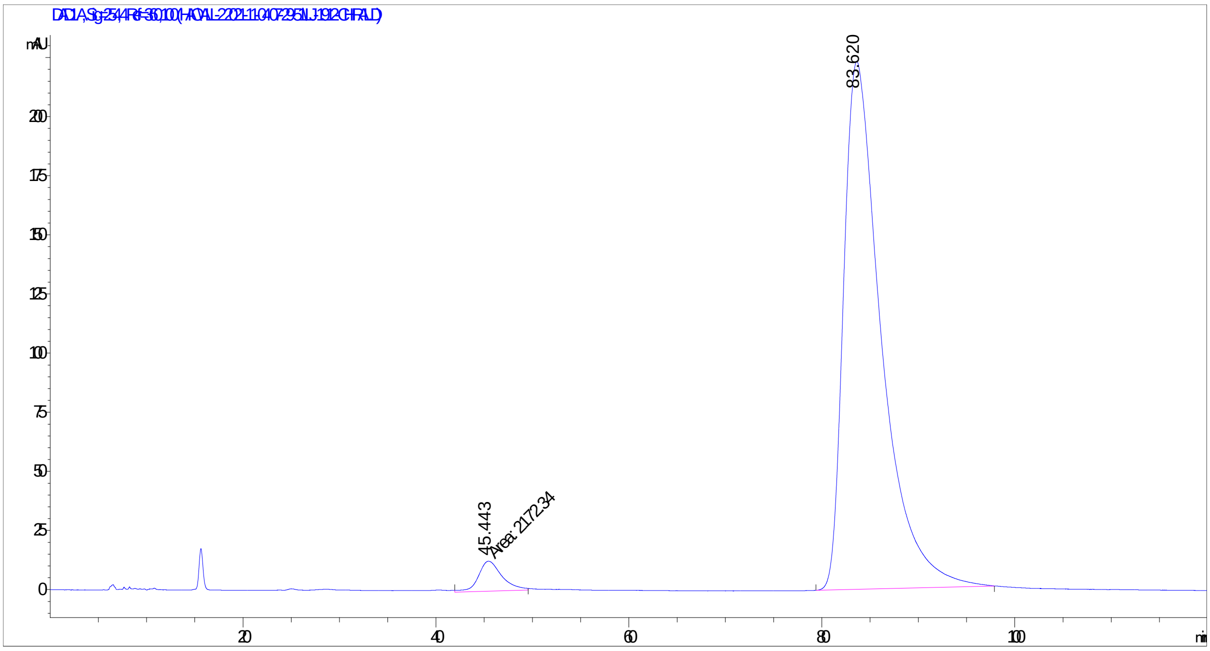

#	Time	Area	Height	Width	Area%	Symmetry
1	45.443	2172.3	12.7	2.8482	3.471	0.745
2	83.62	60413.2	223.3	3.331	96.529	0.492

3bb HPLC traces: racemate top, enantiomer bottom.

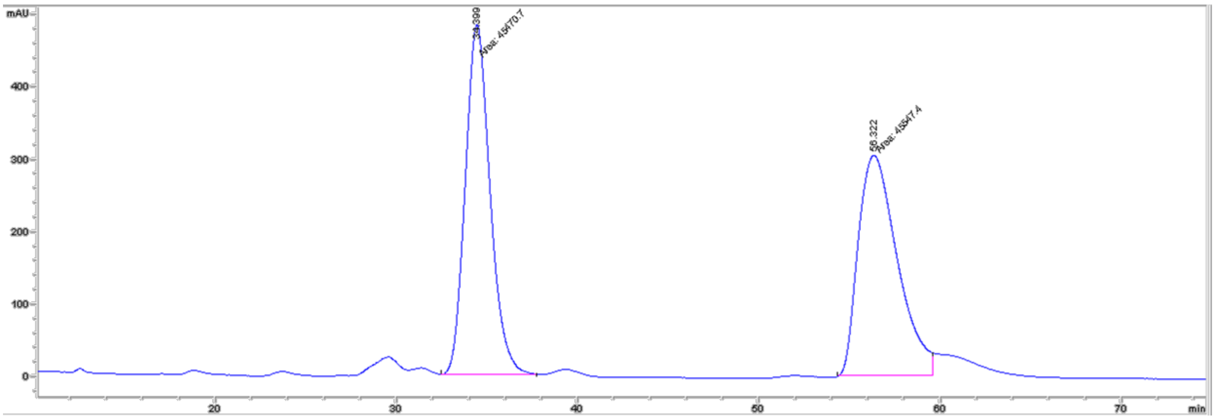

#	Time	Area	Height	Width	Area%	Symmetry
1	34.399	45470.7	482.3	1.5713	49.958	0.843
2	56.322	45547.4	304.5	2.4933	50.042	0.654

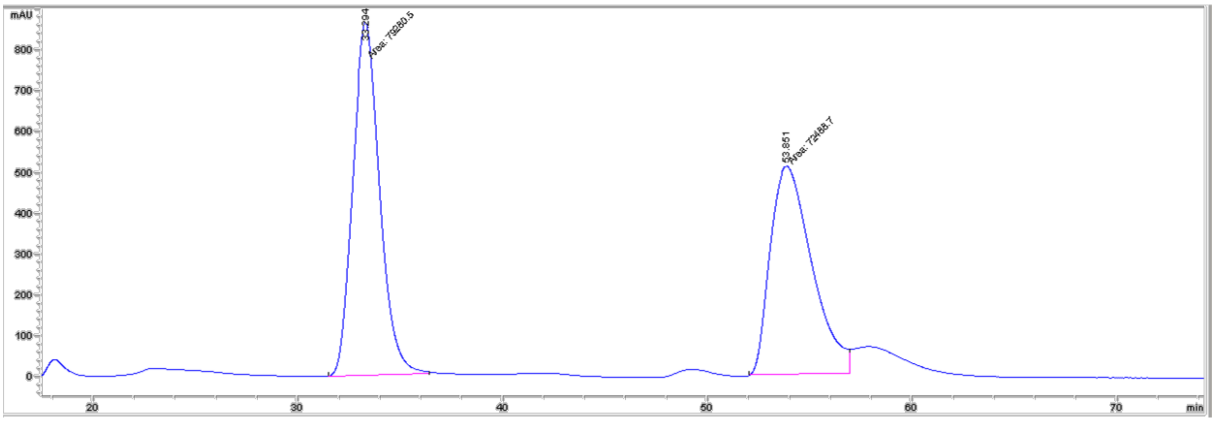

#	Time	Area	Height	Width	Area%	Symmetry
1	33.294	79280.5	862.4	1.5322	52.238	0.903
2	53.851	72488.7	509.9	2.3695	47.762	0.643

^{1}H NMR Spectrum of **3cc** (700 MHz, $CDCl_3$)

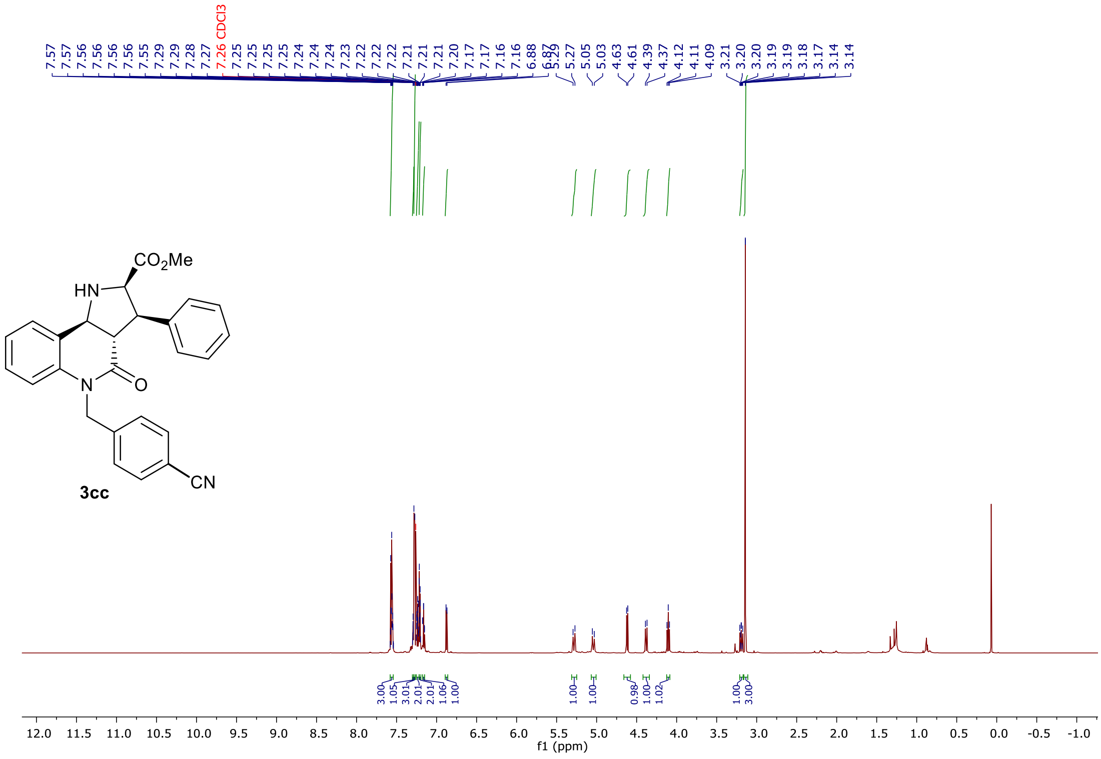

^{13}C NMR Spectrum of **3cc** (176 MHz, $CDCl_3$)

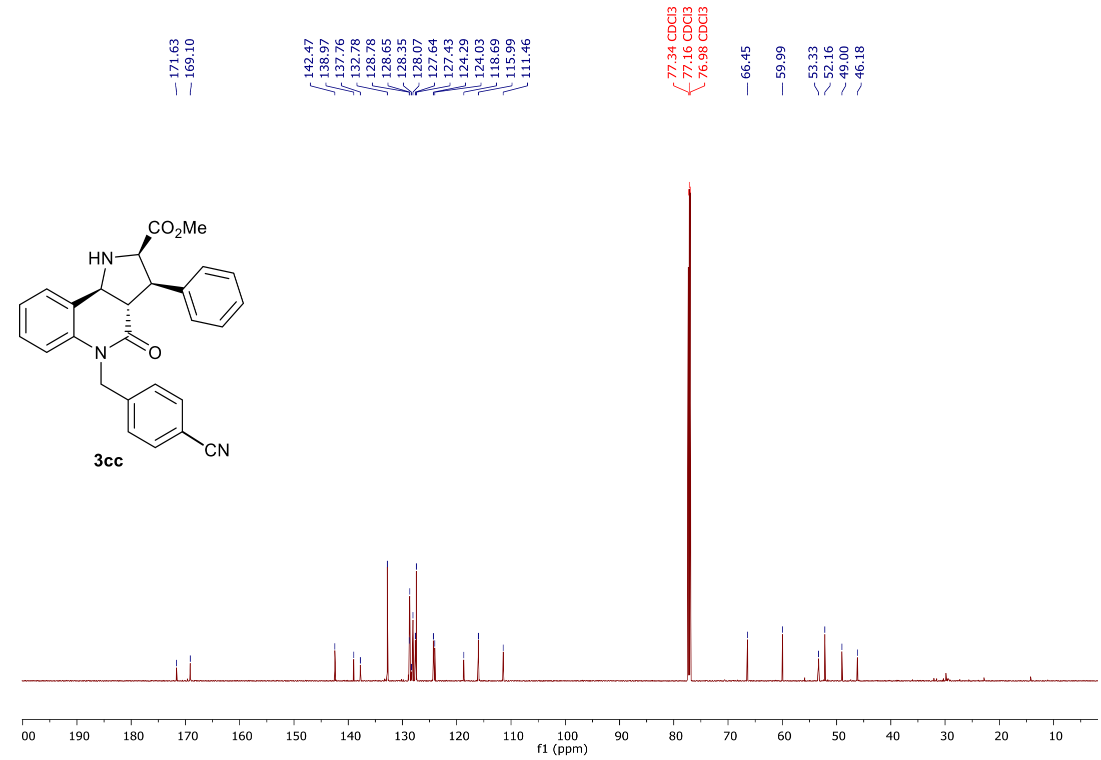

3cc HPLC traces: racemate top, enantiomer bottom.

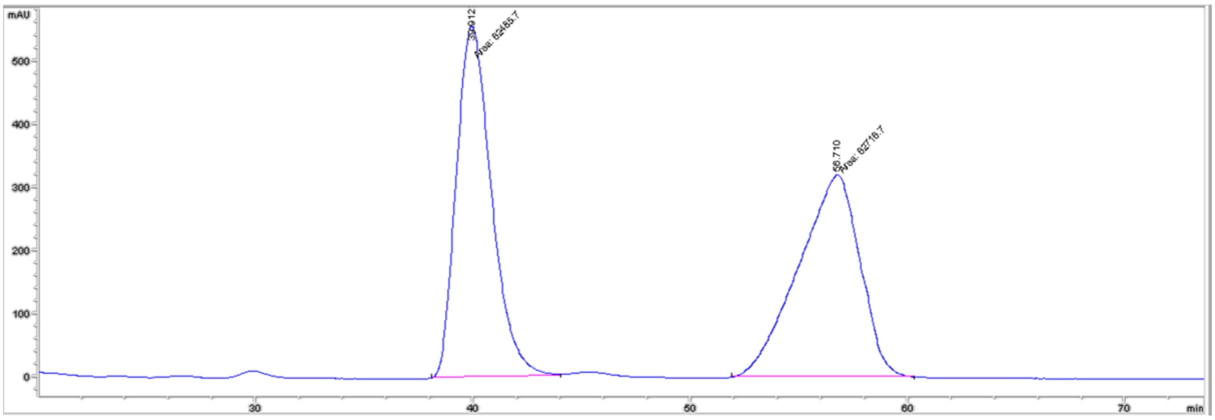

#	Time	Area	Height	Width	Area%	Symmetry
1	39.912	62485.7	554.4	1.8786	49.908	0.736
2	56.71	62716.7	319.1	3.2752	50.092	1.597

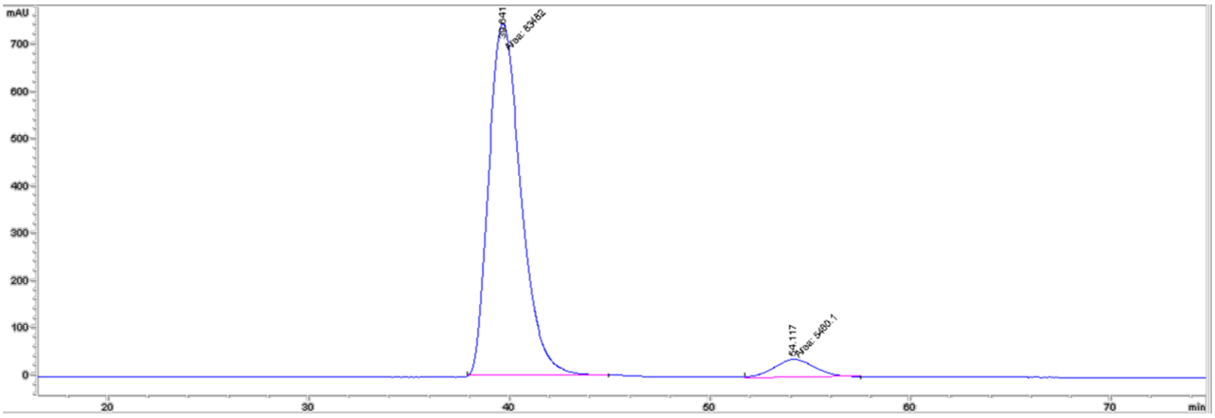

#	Time	Area	Height	Width	Area%	Symmetry
1	39.641	83482	744.6	1.8687	93.861	0.724
2	54.117	5460.1	38	2.3932	6.139	0.882

8. References

1. J. Taipale, J. K. Chen, M. K. Cooper, B. Wang, R. K. Mann, L. Milenkovic, M. P. Scott and P. A. Beachy, *Nature*, 2000, **406**, 1005-1009.
2. H. Sasaki, Y. Nishizaki, C. Hui, M. Nakafuku and H. Kondoh, *Development*, 1999, **126**, 3915-3924.
3. R. J. Lipinski, J. J. Gipp, J. Zhang, J. D. Doles and W. Bushman, *Exp. Cell Res.*, 2006, **312**, 1925-1938.
4. G. Regl, G. W. Neill, T. Eichberger, M. Kasper, M. S. Ikram, J. Koller, H. Hintner, A. G. Quinn, A.-M. Frischauf and F. Aberger, *Oncogene*, 2002, **21**, 5529-5539.
5. M. W. Pfaffl, *Nucleic Acids Res.*, 2001, **29**, e45-e45.
6. T. A. Halgren, *J. Comput. Chem.*, 1996, **17**, 490-519.
7. *Spartan 14, Wavefunction Inc., Irvine, CA, USA, **2014.***
8. *Gaussian 09, Rev E.01, M. J. Frisch, G. W. Trucks, H. B. Schlegel, G. E. Scuseria, M. A. Robb, J. R. Cheeseman, G. Scalmani, V. Barone, B. Mennucci, G. A. Petersson, H. Nakatsuji, M. Caricato, X. Li, H. P. Hratchian, A. F. Izmaylov, J. Bloino, G. Zheng, J. L. Sonnenberg, M. Hada, M. Ehara, K. Toyota, R. Fukuda, J. Hasegawa, M. Ishida, T. Nakajima, Y. Honda, O. Kitao, H. Nakai, T. Vreven, J. J. A. Montgomery, J. E. Peralta, F. Ogliaro, M. Bearpark, J. J. Heyd, E. Brothers, K. N. Kudin, V. N. Staroverov, T. Keith, R. Kobayashi, J. Normand, K. Raghavachari, A. Rendell, J. C. Burant, S. S. Iyengar, J. Tomasi, M. Cossi, N. Rega, J. M. Millam, M. Klene, J. E. Knox, J. B. Cross, V. Bakken, C. Adamo, J. Jaramillo, R. Gomperts, R. E. Stratmann, O. Yazyev, A. J. Austin, R. Cammi, C. Pomelli, J. W. Ochterski, R. L. Martin, K. Morokuma, V. G. Zakrzewski, G. A. Voth, P. Salvador, J. J. Dannenberg, S. Dapprich, A. D. Daniels, O. Farkas, J. B. Foresman, J. V. Ortiz, J. Cioslowski, D. J. Fox, Wallingford CT, USA, **2013.***
9. O. V. Dolomanov, L. J. Bourhis, R. J. Gildea, J. A. K. Howard and H. Puschmann, *J. Appl. Cryst.*, 2009, **42**, 339-341.
10. G. Sheldrick, *Acta Crystallogr. Sect. A*, 2015, **71**, 3-8.
11. G. Sheldrick, *Acta Crystallogr. Sect. A*, 2008, **64**, 112-122.
12. M. Lang and J. Wang, *Org. Chem. Front.*, 2019, **6**, 1367-1371.
13. J. Liu, G. S. Cremosnik, F. Otte, A. Pahl, S. Sievers, C. Strohmann and H. Waldmann, *Angew. Chem., Int. Ed.*, 2021, **60**, 4648-4656.
14. M. Grigalunas, S. Patil, A. Krzyzanowski, A. Pahl, J. Flegel, B. Schölermann, J. Xie, S. Sievers, S. Ziegler, H. Waldmann, *Chem. Eur. J.,* **2022**, *28*, e202202164.
